# A catalogue of Lithuanian beetles (Insecta, Coleoptera)

**DOI:** 10.3897/zookeys.121.732

**Published:** 2011-08-05

**Authors:** Vytautas Tamutis, Brigita Tamutė, Romas Ferenca

**Affiliations:** 1 Kaunas T. Ivanauskas Zoological Museum, Laisvės al. 106, LT-44253 Kaunas, Lithuania; 2 Department of Biology, Vytautas Magnus University, Vileikos 8, LT-44404 Kaunas, Lithuania; 3 Nature Research Centre, Institute of Ecology, Akademijos 2, LT-08412 Vilnius, Lithuania

**Keywords:** Coleoptera, Lithuania, catalogue, bibliography, fauna

## Abstract

This paper presents the first complete and updated list of all 3597 species of beetles (Insecta: Coleoptera) belonging to 92 familiesfound and published in Lithuania until 2011, with comments also provided on the main systematic and nomenclatural changes since the last monographic treatment in two volumes (Pileckis and Monsevičius 1995, 1997). The introductory section provides a general overview of the main features of the territory of Lithuania, the origins and formation of the beetle fauna and their conservation, the faunistic investigations in Lithuania to date revealing the most important stages of the faunistic research process with reference to the most prominent scientists, an overview of their work, and their contribution to Lithuanian coleopteran faunal research.

Species recorded in Lithuania by some authors without reliable evidence and requiring further confirmation with new data are presented in a separate list, consisting of 183 species. For the first time, analysis of errors in works of Lithuanian authors concerning data on coleopteran fauna has been conducted and these errors have been corrected. All available published and Internet sources on beetles found in Lithuania have been considered in the current study. Over 630 literature sources on species composition of beetles, their distribution in Lithuania and neighbouring countries, and taxonomic revisions and changes are reviewed and cited. An alphabetical list of these literature sources is presented. After revision of public beetle collections in Lithuania, the authors propose to remove 43 species from the beetle species list of the country on the grounds, that they have been wrongly identified or published by mistake. For reasons of clarity, 19 previously noted but later excluded species are included in the current checklist with comments. Based on faunal data from neighbouring countries, species expected to occur in Lithuania are matnioned. In total 1390 species are attributed to this category and data on their distribution in neighbouring countries is presented. Completion of this study provides evidence that the Lithuanian coleopteran fauna has yet to be completely investigated and it is estimated that approximately 28 % of beetle species remain undiscovered in Lithuania. More than 85% of beetle species expected for Lithuania have been found in the following families: Cerylonidae, Geotrupidae, Haliplidae, Kateridae, Lycidae, Lucanidae, Mycetophagidae, Scarabaeidae and Silphidae. In families with few species such as Alexiidae, Boridae, Byturidae, Dascilidae, Drilidae, Eucinetidae, Lampyridae, Lymexilidae, Megalopodidae, Nemonychidae, Nosodendridae, Noteridae, Orsodacnidae, Pyrochroidae, Pythidae, Psephenidae, Rhysodidae, Sphaeritidae, Sphaeriusidae, Sphindidae, Stenotrahelidae and Trogidae, all possible species have already been discovered. However in some beetle families such as Aderidae, Bothrideridae, Eucnemidae, Laemoploeidae, Mordellidae, Ptiliidae, Scraptidae and Throscidae less than 50% of all possible species are known. At present the beetle species recorded in Lithuania belong to 92 families, with species from 9 other families such as Agyrtidae, Biphylidae, Deradontidae, Mycteridae, Ochodaeidae, Phleophilidae, Phloeostichidae, Prostomidae, Trachypachidae are expected to be found.

A bibliography and a index of subfamily and genus levels are provided. The information published in the monograph will serve to further faunistic and distribution research of beetles and will help to avoid confusion in the identificatation of coleopteran fauna of Lithuania.

## Dedications

We dedicate the present work to Professor Simonas Pileckis, who laid the foundation of coleopterology in Lithuania and taught us to discover the wonders of the beetle’s world. I also dedicate this paper to my parents, Jurgiui and Janinai Tamučiams, for encouraging my childhood interests in natural history (VT).

## Introduction

The Order Coleoptera, or beetles, forms the most numerous group of insects throughout the animal kingdom. In ecological terms this is a very diverse group of insects occupying a wide variety of terrestrial and freshwater ecological niches. The value of these animals to terrestrial ecosystems and to humankind is enormous, and thus, knowledge of fauna is of particular importance.

Faunistic research is essential in assessing biodiversity in a given area and also for understanding processes at present occurring in nature related to the increasing anthropogenic impact and climate change. Faunistic research data are important not only to fundamental sciences, but it also can be widely used in many fields of applied sciences such as agriculture, forestry, ecology, environmental protection and many others. Inventories of such data and making a checklist are the most important steps to make the data accessible and suitable for use by the general public. Moreover, faunistic studies have both scientific and cultural-social importance. The above-mentioned aspects directly show the awareness of society about own country’s natural history and the necessity of its conservation.

The last 20 years has been a time of major changes in beetle classification. It can be summarized from the reviewed literature that over 600 beetle species’ names have been changed since their publication in the previous monograph of Lithuanian beetles (Pileckis and Monsevičius 1995, revised 1997) due to reidentification and spelling emendations. Such situation causes increasing discrepancies in nomenclature of beetles species used by various authors in the Lithuanian faunistic literature. This in turn leads to numerous misunderstandings and is highly inconvenient for anybody working with faunal lists, especially one who is not well acquainted with current beetle literature. Moreover, the necessity to summarize all the available literature on Lithuanian beetles was felt for a long time. A lot of information scattered in various publications was forgotten and unusable in the studies of Lithuanian Coleoptera. Therefore, the publication of a new, updated checklist became an urgent necessity. To fill this gap we decided not only to compile the list of Lithuanian beetles, but also to summarize all available literature sources on Lithuanian beetle fauna, to create the bibliographic list, and to present reference sources for each species separately. In addition, we predicted the expected species for Lithuania based on their presence in faunas of neighboring republics and considering conditions in these countries from both geographic and ecological aspects. Analyzing beetle fauna of such large countries as Sweden, Poland and Belarus, we considered the importance of assessing the diversity of natural systems in large territories. Species predicted for Lithuania are found in regions adjacent to our country, where natural conditions are similar to those in Lithuania; therefore, there is a high likelihood of detection in Lithuania. We believe that these forecastsprovide directions to faunistic research and facilitate the search of new species. Additionaly, we decided to comment on the main changes in beetle classification as well as mention the untrustworthy notifications of some species listed in the recently published multivolume Catalogue of Palaearctic Coleoptera, Web project “Fauna Europaea Web Service,” various regional catalogues and species lists. We hope this work will encourage researchers to look deeper into species distribution over the whole territory of the Republic. Considering distribution ranges of some species and their revised identifications, we suggest to remove 43 species from the list of Lithuanian beetles. We hope the list of beetles accomplished by us and presented in this paper will be useful for regional research in applied and conservation studies of beetles. Furthermore, it is our intention to help both Lithuanian and foreign entomologists to realize a truer picture of the current beetle diversity in Lithuania and to present a species list which is up to date as of 2010, with all doubtful entries indicated. We reference herein all new taxonomic placements and provide explanations for every nomenclatural change compared to those published in two volumes Lithuanian Fauna. The Beetles (Pileckis and Monsevičius 1995, 1997).

Unfortunately we failed to avoid some shortcomings in our paper. In our opinion one of the major disadvantages is that the information presented is based mostly only on published sources. Much of the faunistic data is accumulated in the database and collections of Kaunas T. Ivanauskas zoological museum, other collections stored in the Lithuanian universities or private collections, in the museums of foreign countries, but it is impossible physically for us to process the data and present it in this catalogue. In our view it will be much better to revise particular families or subfamilies and to use this data in the future and we sincerely hope that our catalogue will stimulate such process.

### Characterisation of territory

The main Lithuanian geomorphological features formed during the several glacial periods which started in the Pleistocene Epoch. The last glacier covered almost all Lithuania except the southeastern part. During this period large moraine structures formed from glacial drifts (Česnulevičius 1998). Morainae glacial lakes and the old alluvial plains, morainal hills, littoral plains, zandric plains and river valleys dominate in the Lithuanian landscape. Sand dunes stretch in the western country which is in the Curonian Spit and the continental seaside. Hilly and lake-rich landscape dominates in eastern, southern and western hills. There are various areas of agriculture, forests, lakes and river valleys. Marshes, swamps and other wetlands are common in lowlands and valleys. Wide and the homogeneous moraine and limnoglacial plains landscape is characteristic in central, northern and southeastern Lithuania. In these regions agriculture is developed because of rich soil. Firs, birches, alder or aspens dominate in small forests. Broad-leaved forests are common in the southwestern area. It is important to note that the common hornbeam (*Carpinus betulus* L.) grows naturally in this territory. This is northeastern habitat limit of the species (Navasaitis 2004).

The Lithuanian territory belongs to the Central European forest zone and mixed forest zone. The Lithuanian forests were forming after the last glacial period. Over the last 13 thousand years the forests have been changing from arctic tundra, northern taiga to broad-leaved forests. Pollen spectrum analysis has shown that the Lithuanian forests amounted to 50% of broad-leaved forests, especially oak woodlands, 7500 years ago. During the earlier historic periods the forests covered the major part of today‘s country. It has been estimated that in the 11th - 13th century the forests reached 55%, wetlands and water bodies 23%, and 22% belonged to the agrarian landscape (Kairiūkštis 2003). However, this proportion has changed drastically due to an intensified human economic activity during the last century. Recently the Lithuanian forests cover 19 677 square kilometers and provide 31.7 % of its area. Coniferous woods are 58.8%, the soft leafy forests 35.8%, whereas the hard ones amount to 4.5% of all forests area (Lithuanian Forest Statistics 2004). In southwestern and central Lithuanian Republic the dominating primeval broad-leaved forests have been almost destroyed. The major part has been planted or is "semi-natural". Agricultural lands, covering 53% of all country’s area, have been formed after cutting down the forests and draining the larger half of the marshes (Kurlavičius 2010). The Lithuanian grasslands and natural pastures provide 11% of the agricultural lands or cover more than 47 580 square kilometers. The wetlands cover 5-6% of the Lithuanian territory, whereas rivers, lakes and dams only 4%.

### Climate

The Lithuanian climate is often influenced by the Atlantic cyclones which bring much humidity and reduce the differences in temperatures. In the west of the country, especially at the Baltic Sea coast, the maritime climate is evident, but its features decrease in the east (Kaušyla 1981). The multi-year average temperature is 6.2°C, the absolute maximum temperature is 37.5°C and the minimum is -49°C. A vegetation period, when the average day temperature does not exceed 10°C, lasts 145 days averagely. According to the precipitation quantity, Lithuania is in a surplus drainage zone. The average annual precipitation is 661.5mm. The distribution of precipitation is uneven: in the west the quantity is the largest which reaches approximately 900 mm and it does not exceed 500mm of the annual precipitation quantity in the centre and north of the plains in the country. The coldest annual month is January and the average temperature of this month is -5 - 6°C. The warmest month is July when the average temperature reaches 16°C.

### Origin and formation of the fauna

Lithuanian beetle faunal structure and composition are closely related to the formative processes of the landscape which took place in this territory. The first, i.e. the oldest Lithuanian territory’s beetles have the characteristic features to exist in tundra and taiga, glacial relicts. They originated during the Subarctic Period, approximately 12-10 thousand years ago. The following betle species could be referred as glacial relicts: *Miscodera arctica*, *Blethisa multipunctata*, *Pycnoglypta lurida*, *Oxyporus mannerheimi*, *Ceruchus chrysomelinus*, *Ditylus laevis*, *Trogosoma desparium*, *Evodinus borealis*, and *Semanotus undatus* amongst others. These species moved to the north of the country when the climate became warmer, while mixed and leafy forests began to form in the Lithuanian territory. During that time eastern Europe and western Siberia species related to pine and small-leaved forests came into existence in the Lithuanian beetle fauna. Approximately 6 thousand years ago the Lithuanian climate became humid, broad-leaved tree species were proliferating as well as the following species belonging to the west European fauna, for instance *Calosoma sycopantha*, *Carabus intricatus*, *Carabus coriaceus*, *Dorcus paralelepipedus*, *Lucanus cervus*, *Osmoderma barnabita*, *Liocola marmorata*, *Gnorimus variabilis*, *Stenogostus rufus*, *Elater ferrugineus*, *Crepidophorus mutilatus*, *Cucujus cinnaberinus*, *Cucujus haematodes*, *Neatus picipes*, *Platydema violacea*, *Colydium elongatum*, and *Cerambyx cerdo*. Today there are only small fragments of natural and primeval forests in an agricultural and urban landscape or placesdifficult to approach; therefore, almost all species have become rare and are frequently referred as relicts of the Atlantic Period. The beetle species, ecologically related to open biotypes, can be considered as the youngest in Lithuania. When the area of forests decreased and open habitation expanded the species characteristic to forest steppe and steppe areas proliferated in Lithuania, such as *Diachromus germanus*, *Harpalus hirtipes*, *Harpalus froelichi*, *Copris lunaris*, *Othophagus* sp., *Hoplia graminicola*, *Agriotes lineatus*, *Tytthaspis sedecimpunctata*, *Phytoecia virgula*, *Chrysolina aurichalcea*, *Baris artemisia* and similar ones. Beetle species associated with nutritional relations to the increasing number of cultural plants variety found their way into Lithuania while its agriculture was developing. Such species are: *Leptinotarsa decemlineata*, *Phaedon armoraciae*, *Phyllotreta armoraciae*, *Psylliodes chlorosephala*, *Bruchus rufimanus*, *Tanymecus palliatus*, and *Ceutorhynchus pleurostigma*.

International tourism and trade has been developing, and thanks to it some species have spread in Lithuania: *Alphitobius diaperinus*, *Trogoderma angustum*, *Rhyzopertha dominica*, *Attagenus smirnovi*, *Lyctus africanus*, *Glischrochilus quadrisignatus*, *Bruchus pisorum*, *Acanthoscelides obtectus*, and *Sitophilus oryzae*.

There are no natural barriers preventing the beetle migration in the Lithuanian territory. Therefore, it is evident that the Lithuanian fauna is still in the process of formation. Over the last ten years a number of beetles specific to southern-middle Europe have been discovered, for example *Leistus rufomarginatus*, *Tachyura parvula*, *Acupalpus luteatus*, *Elater ferrugineus*, *Isoriphis melasoides*, *Melasis buprestoides*, *Xylotrechus pantherinus*, *Oulema tristis*, and *Magdalis caucasica*. However, the assumptions must be proved the origin of these species in Lithuania was determined by the climate or whether they are the relicts of the Atlantic Period.

### Conservation

The ways to prevent the extinction of plant and animals species, or at least to predict this process and control it, have been sought worldwide for long time. So far, most of the species have disappeared without our knowledge or even without our awareness of their impending departure. The beginning of animal and plant protection can be related to the first nature reserve establishment. In 1937 Žuvintas National Reserve was established on the initiative of a famous scientist, Professor T. Ivanauskas, in order to preserve the unique ecosystem complex of Žuvintas Lake and its surrounding marshes (Kirstukas 2004). Despite that, the list of rare and endangered species was first considered only in 1959 after the first Nature Protection Law had been enacted. In 1996 the Committee for Protection of Nature approved the list of protected plants species compiled by the Lithuanian Academy of Science’s Institute of Botany and Society of Botany. It includes 176 rare or endangered vascular plant species. Following the advice of the Committee for the Protection of Nature, the former Council of Ministers of the Lithuanian SSR established the Lithuanian SSR Red Book in 1976 , standardising the listing procedures. The Lithuanian Red Data Book was published in 1981. It lists 41 species of animals, the complete genus of bumblebees (*Bombus*), as well as 30 species of vascular plants (Jezerskas et al. 1981). However only one beetle species (*Lucanus cervus*) was included in the this Red Data Book as a very rare and relict species. Since 1990, Environmental Protection Department of the Republic of Lithuania has been responsible for the compilation and updating of the second edition of the Lithuanian Red Data Book. Overall 102 insect species have been listed in this edition, of which 17 were beetles (Balevičius 1992). Regarding the Lithuanian biodiversity research and its conservation, the Lithuanian situation changed after gaining its independence and signing the Rio de Janeiro Declaration, as well as after the ratification of the International Convention on Biological Diversity (CBD). In order to solve the problems of the protection of the most valuable and endangered species and their habitations, the Birds Directive (1979) was approved, the Network of Protected Areas (Natura 2000) was established, the Habitat Directive (1992), the European Community Strategy on Biological Diversity (1998) and other EU legislative acts and initiatives were implemented in Europe. That fact influenced the separation of valuable territories, regarding their biological diversity, which were given the status of protected areas. According to the High Conservation Values Forests (HCVF) concept, the Lithuanian forests, covering the area of 264.28 square kilometers, were acknowledged to have an exceptional conservation value when the Inventory Project of Woodland Key Habitat was under way in 2000-2005 (Andersson et al. 2005).

The Lithuanian protected territorial system consists of three nature and cultural reserves, one biosphere reserve, five national parks, 30 regional parks and 254 national reserves. The protected territorial system, including nature monuments and nationally protected nature heritage monuments, cover about 7.86 thousand square kilometres (approximately 12% of the country‘s territory) (Kirstukas 2004).

The list of protected insect species has been supplemented substantially in 2003 and a new edition of the Lithuanian Red Data Book has been published (Rašomavičius 2007). Altogether 767 species of animals, plants, fungi and lichens have been included in this book. From the 128 listed insect species, 32 belong to the order Coleoptera (Table 1); however these species have been included in latest Red Data Book on the basis of the status of the species in the list of protected animals of Natura 2000; some of them, such as *Ostoma ferruginea* *Graphoderus bilineatus*, *Dytiscus latissimus*, and *Agonum ericeti* are not very rare or endangered in Lithuania. At the same time this book does not include such species as *Calosoma auropunctatum*, *Velleius dilatatus*, *Emus hirtus*, *Hyleocoetus flabellicornis*, *Metoecus paradoxus*, *Tragosoma depsarium*, etc., which are rare in the territory of Lithuania and in need of protection.

**Table 1.** The species of beetles (Coleoptera) listed in the Red Data Book (RDB) in Lithuania. Categories: **Ex** extinct; **E** endangered; **V** vulnerable; **R** rare; **I** indeterminate; **Rs** restored.

**Name of species**	**Category of species**	
	*(RDB)1992*	*(RDB)2007*
*Agonum ericeti* (Panzer)	–	R
*Anostirus purpureus* (Poda)	–	R
*Boros schneideri* (Panzer)	E	R
*Calosoma inquisitor* (Linnaeus)	V	R
*Calosoma sycopantha* (Linnaeus)	V	Ex
*Carabus coriaceus* Linnaeus	V	R
*Carabus intricatus* Linnaeus	–	V
*Carabus menetriesi* Hummel	I	–
*Carabus nitens* Linnaeus	R	R
*Cassida margaritacea* (Schaller)	–	I
*Cerambyx cerdo* Linnaeus	E	Ex
*Ceruchus chrysomelinus* (Hochenwarth)	E	R
*Cicindela maritima* Dejean	V	R
*Cucujus cinnaberinus* (Scopoli)	–	V
*Cucujus haematodes* (Erichson)	–	V
*Dendroxena quadrimaculata* (Scopoli)	V	R
*Dytiscus latissimus* Linnaeus	–	I
*Ergates faber* (Linnaeus)	V	V
*Gnorimus variabilis* (Linnaeus)	V	R
*Graphoderus bilineatus* (DeGeer)	–	I
*Lamprodila rutilans* (Fabricius)	–	I
*Lucanus cervus* Linnaeus	E	Ex
*Melanophila acuminata* (DeGeer)	–	I
*Necydalis major* (Linnaeus)	–	I
*Osmoderma eremita* (Scopoli)	V	V
*Ostoma ferruginea* (Linnaeus)	–	I
*Peltis grossa* (Linnaeus)	E	R
*Polyphylla fullo* (Linnaeus)	R	R
*Prionus coriarius* (Linnaeus)	–	R
*Protaetia lugubris* (Herbst)	V	V
*Stenagostus rufus* (DeGeer)	–	I
*Uloma culinaris* (Linnaeus)	–	I
*Xestobium rufovillosum* (DeGeer)	–	I

### History of Coleoptera research

**Figure 1.**
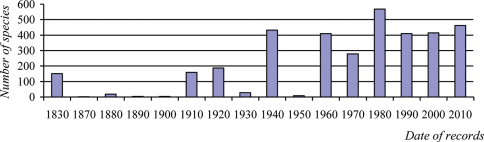
Rate of publication of first records of beetle species for Lithuania.

Research on Lithuanian Coleoptera does not have a long history. The first records of beetles found in Lithuania were noted by Eduard von Eichwald at the beginning of the 19th century (Eichwald 1830). In his book *Zoologia Specialis*, he mentioned approximately 150 species of beetles. Some beetle species were mentioned in other works of that century (Wankowicz 1869; Lindeman 1871; Lentz 1879; Osterloff 1889; Seidlitz 1891; Vozhev 1892), but some doubts concerning their authenticity exist since exact locations of the finds were not indicated and the territory of Lithuania in the 19th century and today differ. Some information about Lithuania’s insect fauna can be found in the works published at the beginning of the 20th century. In 1903, Lucas von Heyden released data regarding beetles collected in the environs of Kaunas - 205 species of beetles were reported for Lithuania, 158 of which were recorded for the first time. Ján Roubal (1910) published data on beetles collected in the environs of Vilnius in 1904-1907; 160 species of beetles were included on this list, 120 of which were noted for the first time in Lithuania. Some insect species were also attributed to the Lithuanian fauna in works published in neighbouring countries (Lomnicki 1913; Jakobson 1905-1915). Here, 67 species of insects were noted for Lithuania for the first time. More significant research work concerning the insect fauna of Lithuania was carried out in Vilnius University during the interwar period. However, particular attention was only paid to certain families of beetles such as Haliplidae, Dytiscidae, Carabidae, Rhysodidae, Hydrophilidae, Hydreanidae (Ogijewicz 1933), Silphidae (Kopyłówna 1935), Buprestidae, Elateridae (Ogijewicz 1939), Cerambycidae (Zawadzki 1936; Stanilisówna 1939) and Chrysomelidae (Kamiński 1936). Additionally, quite detailed results of faunistic research carried out in the eastern part of present-day Lithuania were published by Gabriela Mazurowa and Edward Mazur (1939). During this period, besides faunistic research into Coleopteran fauna, research in applied entomology related to agricultural and forest pests were also pursued (Ivanauskas and Vailionis 1922; Mastauskis 1925, 1927, 1936; Ogijewicz 1929, 1931, 1932, 1934, 1938). By 1940, in the current territory of Lithuania, 981 species of beetles had been recorded. During the post-war period from 1945 to 1955, little data concerning the Lithuania fauna was published. Records of 18 species of beetles were mentioned for Lithuania for the first time in the works of Prüffer (1948), (Horion (1949, 1955), (Richter (1949, 1952), and Medvedev (1952). Focused beetle research has been conducted by Stanislovas Mastauskis and Simonas Pileckis since 1955 in the Department of Plant Protection, Lithuanian Academy of Agriculture (currently the University). Lithuania’s beetle species list was considerably enlarged after the beetle collections of entomologist Alfonsas Palionis were analysed. Based on Palionis’ data, 351 beetle species new for Lithuania were registered. Moreover, important works concerning harmful beetles in Lithuanian forests were published; these including nine species of bark beetles (Curculionidae: Scolytinae) (Mastauskis and Pileckis 1959) and four species of jewel beetles (Buprestidae) (Pileckis 1958a) recorded for the first time in Lithuania. Pileckis (1960), who composed the first list of Lithuanian beetles, made a great contribution to the exploration of the fauna. The author presented a critical analysis of previous publications regarding Lithuanian beetles and summarised results of the audit of beetle collections held in natural history museums of Lithuania and in private collections. In the first list of Lithuanian beetles, 1446 beetle species were listed, 122 of which were for the first time. It should be noted that Pileckis (1960) not only notified new beetle species for Lithuania, but also proposed to remove some species from the list of misidentified beetles such as *Lethrus apterus*, *Aphodius alpinus*, *Anoplodera sequensi*, *Clytus rhamni*, *Isotomus speciosus* and *Acanthocinus reticulatus*. In the years after 1960, numerous works of faunistic and applied entomology were published (Pileckis 1962, 1963a, b, 1968a, b, 1970a, b, 1972; Lešinskas and Pileckis 1967; Pileckis et al. 1968; Zajančkauskas and Pileckis 1968; Kazlauskas and Pileckis 1968; Mastauskis 1970; Valenta and Jakaitis 1972; Jakaitis 1973; Sharova and Grüntal 1973; Mensonienė 1974) and these were summarised in the first monograph on Lithuanian beetles (Pileckis 1976a). In the monograph, the body composition of beetles, reproductive and developmental characteristics, environmental and distribution peculiarities, regularities of fauna formation, description of research process, as well as identification keys for some beetle groups and a systematic list of Lithuanian beetles are presented. 2203 beetle species were included on this list, 436 of which were mentioned for for the first time. It is worth noting that the 1970s were the most productive period in terms of beetle fauna research in Lithuania: 569 beetle species were noted the first time (Figure 1). It is also worth noting that besides the above-mentioned works, a Prussian beetle list was also published in the same decade. On this list, beetles found in Klaipėda region and near Juodkrantė in the Curonian Spit were noted (Bercio and Folwaczny 1979); 84 beetle species were new for Lithuania. In the 1980s, Vidmantas Monsevičius played a very important role in faunistic research, aiming to determine the species composition of Staphylinidae. During this period, 210 species of rove beetles new for Lithuania were recorded (Monsevičius 1982, 1883, 1985, 1986a, b, 1987, 1988a, b; Monsevičius and Jakaitis 1984). Beetles living in forest ecosystems were also actively studied; 108 new beetle species were found (Perkovskij and Monsevičius 1982; Pileckis and Monsevičius 1982; Pileckis and Jakaitis 1982, 1989; Slavinskas 1982; Pileckis 1984, 1988; Jakaitis 1985; Gaidienė and Ferenca 1988; Ferenca 1988). A significant contribution to the knowledge of the Lithuanian fauna was also made by the Estonian entomologist Georg Miländer; in his work, 42 beetle species new for Lithuania were mentioned (Miländer 1982; Miländer et al. 1984).

A more comprehensive review of faunistic records of beetles in Lithuania was provided by authors of subsequent volumes of the Lietuvos Fauna. Vabalai [Lithuanian Fauna. The beetles] (Pileckis and Monsevičius 1995 and 1997). This monograph consists of two parts, general and systematic. The first deals with the body structure and bionomics of beetles, the history of coleopteran research in Lithuania, stages of beetle fauna formation during the Postglacial Period, peculiarities of coleopteran distribution in the Republic as well as their significance. In the second part the descriptions of families of Coleoptera, genera and some species as well as distribution (in five regions) and occurrence rate (within 5 degrees) of each species are given. The monograph includes information on 2895 beetle species; 133 species are noted the first time for Lithuania. It is important to note that this is the first attempt to forecast expected beetle species in Lithuania: a number of species expected for each genus is given, totalling more than 990 additional species. Some valuable faunistic information about can be found in the Catalogue of Entomological Collections of Tadas Ivanauskas Zoological Museum. Therein 75 species of beetles have been recorded for the first time in Lithuania. Some information on new beetle species found in Lithuania can also be found in the catalogues of neighbouring countries (Silfverberg 1992, 2004; Lundberg and Gustafsson 1995; Althoff and Danilevsky 1997). Moreover, some species of beetles have been noted only in these catalogues and are not confirmed by more circumstantial information. The same can be said of later catalogues of faunistic works such as the Catalogue of Palaearctic Coleopteraand *Fauna Europaea Web Service*. In total, 122 species of beetles are noted the first time.

The publication of the most recent monograph (Pileckis and Monsevičius 1995 and 1997) induced an increase in faunistic investigations in Lithuania, which resulted in more than 50 publications containing new faunistic data on beetles the most important are arranged in the table 2.

**Table 2.** Most important publications containing new faunistic data on beetles after 1995 in Lithuania.

**Publications**	**Number of first recorded species for Lithuania**
Barševskis 2001, 2008, 2009	26
Ferenca and Tamutis 2009	15
Ferenca 1996, 2003, 2004	12
Ferenca et al. 2002, 2006	27
Ivinskis et al. 1999; 2008, 2009	20
Monsevičius 1997, 1998, 1999, 2000	25
Monsevičius and Pankevičius 2001	78
Šablevičius 2000a, 2003a, 2004	10
Šablevičius and Ferenca 1995	6
Tamutis 1999, 2002b, 2003, 2004	73
Tamutis and Ferenca 2006	13
Tamutis and Pankevičius 2001	27
Tamutis and Zolubas 2001	9

## Methods and conventions

In this check-list we use the classification of the order Coleoptera accepted by Bouchard et al. (2011), the genera and species names we use following accepted ones in the Catalogue of Palaearctic Coleoptera (Löbl and Smetana (eds.) 2003-2010) with some details accepted by other authors: Staphylinidae by Herman (2001), Buprestoidea by Kuban et al. (2006), Tenebrionoidea by Silfverberg (2004), Cerambycidae by Althoff and Danilevsky (1997), and Curculionoidea by Alonso-Zarazaga and Lyal (1999). The main taxonomic categories referred to in this check-list are the following: order, suborder, family, subfamily, tribe, genus and species. Higher taxonomic categories, like series, super- or sub-tribes, are omitted (for details see Bouchard et al. (2011)). Most common synonyms and subspecies names are included, especially the names used in the two volumes Lithuanian monograph (Pileckis and Monsevičius 1995, 1997). The list of Lithuanian beetles was compiled on the basis of published literature sources and revisions of public beetle collections. The catalogues, checklists and other faunistic literature of neighbouring countries have been used to compile the list of beetles species expected in Lithuanian fauna. The species are arranged alphabetically in their respective genera. Expected for Lithuania species names are placed in square brackets […]. The species excluded from the list of Lithuanian beetles are marked by asterisk (*). The officially protected species, included in the Red Data Book of Lithuania (2007) are marked by the abbreviation RDB. The comments (#) are presented after the list and referenced by sequential numbers.

## Results and discussion

All available published and interactive sources on beetles found in Lithuania have been summarized in the current study. In addition to verified records, 166 species noted for Lithuania only in several European catalogues (*Enumeratio Coleopterorum Fennscandiae, Daniae et Baltiae* (Silfverberg 1992, 2004), *Catalogue of Palaearctic Coleoptera* (Löbl and Smetana (eds.), 2003-2010), *Fauna Europaea Web Service* (2001-2011)) and other works of foreign scientists (Osterloff 1889; Jakobson 1905–1915; Łomnicki 1913; Lundberg and Gustafsson 1995; Althoff and Danilevsky 1997) are included in this new list of Lithuanian beetles (Table 3). The primary sources of these findings remains unknown to the authors. Another 17 species were noted for Lithuania by native authors, but this data is untrustworthy as the collected material is lost and confirmation remain absent.

For reasons of clarity, 19 previously noted but later excluded species are included in the current checklist with comments. Moreover, we recommend the removal of 43 species from the list as they were misidentified and erroneously reported in previous works or are species which have been noted by mistake and their distribution in Lithuania is untrustworthy (Table 4).

As a result, 3597 species of beetles are included in the new checklist. Based on the faunal data from neighbouring countries (Lundberg and Gustafsson 1995; Silfverberg 2004; Telnov 2004, 2010; Barševskis 2003; Barševskis et al. 2005; Alexandrovitch et al. 1996; Szujecki 1961, 1976; Stebnicka 1976, 1978; Smrezyński 1965, 1966, 1968, 1972, 1974, 1976; Burakowski et al. 1973, 1974, 1976, 1978, 1979, 1980, 1981, 1986a, b, c, 1987, 1989, 1990, 1991; Wanat and Mokrzycki 2005, Tarnawski and Buchholz 2008a, b; Süda 2006, 2009; *Fauna Europaea Web Service* (2009) and other faunistic works), a further 1390 species of beetles are included as expected species for Lithuanian fauna (Table 5).

The analysis of faunal data in Lithuania and neighbouring countries provides evidence that the Lithuanian coleopteran fauna has yet to be completely investigated and it is estimated that approximately 28% of beetle species remain undiscovered in Lithuania. More than 85% of expected beetle species have been found in the following families: Cerylonidae, Geotrupidae, Haliplidae, Kateridae, Lycidae, Lucanidae, Mycetophagidae, Scarabaeidae and Silphidae. In families with few species such as Alexiidae, Boridae, Byturidae, Dascilidae, Drilidae, Eucinetidae, Lampyridae, Lymexilidae, Megalopodidae, Nemonychidae, Nosodendridae, Noteridae, Orsodacnidae, Pyrochroidae, Pythidae, Psephenidae, Rhysodidae, Sphaeritidae, Sphaeriusidae, Sphindidae, Stenotrahelidae, and Trogidae all possible species have been already discovered. However in some beetle families such as Aderidae, Bothrideridae, Eucnemidae, Laemoploeidae, Mordellidae, Ptiliidae, Scraptidae and Throscidae less than 50% of all possible species are known. At present, the beetle species belonging to 92 familiesare reported for Lithuania and the species from the other 9 families such as Agyrtidae, Biphylidae, Deradontidae, Mycteridae, Ochodaeidae, Phleophilidae, Phloeostichidae, Prostomidae, Trachypachidae are expected.

**Table 3.** The list of beetles species for which Lithuanian notification is doubtful (**SV** Sweden; **EN** Estonia; **LA** Latvia; **BY** Belarus; **PL** Poland; **KR** Kaliningrad region (Russia); **DK** Denmark).

**Family and species name**	**Distribution in the neighbouring countries**
Bostrichidae	
*Stephanopachys substriatus* (Paykull, 1800)	SV, EN, LA, PL
*Stephanopachys linearis* (Kugelann, 1792)	SV, EN, LA, PL
Brentidae	
*Apion rubens* Stephens, 1839	SV, EN, LA, BY, PL, DK
*Diplapion stolidum* (Germar, 1817)	SV, EN, LA, BY, PL, DK
*Exapion elongatulum* (Desbrochers, 1891)	BY, PL
Bothrideridae	
*Teredus cylindricus* (Olivier, 1790)	SV, BY, PL, DK
Buprestidae	
*Habroloma nanum* (Paykull, 1799)	SV, EN, LA, BY, PL, DK
Cantharidae	
*Rhagonycha fugax* Mannerheim, 1843	EN, LA, BY, PL
Carabidae	
*Agostenus quadrisulcatus* (Paykull, 1790)	LA, BY, PL, KR
*Amara concinna* Zimmermann, 1832	BY, PL, KR
*Amara convexiuscula* (Marsham, 1802)	EN, LA, BY, PL, KR, DK
*Bembidion quadripustulatum* Audinet-Serville, 1821	SV, EN, LA, BY, PL, KR, DK
*Brachinus crepitans* (Linnaeus, 1758)	SV, EN, LA, BY, PL, KR
*Brachinus explodens* (Linnaeus, 1758)	EN, BY, PL, KR, DK
*Calathus mollis* (Marsham, 1802)	SV, EN, LA, PL, DK
*Chlaenielus tibialis* Dejean 1826	LA, BY, PL, KR
*Ocydromus fluviatilis* (Dejean, 1831)	BY, PL
*Paratachys bistriatus* (Duftschmid, 1812)	SV, EN, LA, BY, PL, KR, DK
*Pogonus chalceus* (Marsham, 1802)	PL, DK
*Porotachys bisulcatus* (Nicolai, 1822)	SV, LA, BY, PL, DK
*Zabrus tenebrioides* (Goeze, 1777)	PL
Cerambycidae	
*Acmaeops septentrionis* (Thomson, 1866)	SV, EN, BY, PL
*Anaglyptus mysticus* (Linnaeus, 1758)	SV, EN, LA, BY, PL, KR, DK
*Chlorophorus figuratus* (Scopoli, 1763)	LA, BY, PL
*Necydalis ulmi* Chevrolat, 1836	LA, BY, PL
*Echinocerus floralis* (Pallas, 1773)	LA, PL
*Oberea linearis* (Linnaeus, 1761)	LA, BY, PL
*Rhagium bifasciatum* (Hochhuth, 1849)	EN, LA, PL, KR, DK
*Ropalopus femoratus* (Linnaeus, 1758)	SV, LA, PL, KR
*Saperda punctata* (Linnaeus, 1767)	LA, BY, PL
*Tetrops starkii* Chevrolat, 1859	SV, PL, DK
Chrysomelidae	
*Altica brevicollis* Foudras, 1860	SV, EN, LA, BY, PL, DK
*Chaetocnema subcoerulea* (Kutschera, 1864)	SV, EN, LA, BY, PL, DK
*Chrysolina kuesteri* (Helliesen, 1912)	LA, BY, PL
*Cryptocephalus elegantulus* Gravenhorst, 1807	LA, BY, PL
*Cryptocephalus punctiger* Paykull, 1799	SV, EN, LA, BY, PL, KR, DK
*Donacia brevitarsis* Thomson, 1884	SV, LA, BY, PL, DK
*Donacia obscura* Gyllenhal, 1813	SV, EN, LA, BY, PL, KR, DK
*Galeruca jucunda* Faldermann, 1837	SV, PL
*Galerucella sagittariae* (Gyllenhal, 1713)	SV, EN, LA, BY, PL, DK
*Labidostomis cyanicornis* Germar, 1822	EN, LA, BY, PL
*Labidostomis lepida* Lefèvre, 1872	LA, BY
*Longitarsus longipennis* Kutschera, 1864	EN, BY, PL
*Timarcha goettingensis* (Linnaeus, 1758)	LA, BY, PL
Ciidae	
*Cis submicans* (Abeille de Perrin, 1874)	SV, EN, LA, PL, DK
*Cis rugulosus* Mellié, 1848	SV, LA, PL, DK
*Cis castaneus* Mellié, 1848	SV, LA, BY, PL, DK
Clambidae	
*Calyptomerus dubius* (Marsham, 1802)	SV, PL, DK
*Clambus pubescens* Redtenbacher, 1849	SV, EN, LA, PL, DK
*Clambus punctulum* (Beck, 1817)	SV, EN, LA, BY, PL, DK
*Clambus gibbulus*(LeConte, 1850)	SV, EN, LA, BY, PL, DK
Cleridae	
*Opilo mollis* (Linnaeus, 1758)	SV, EN, LA, PL, DK
Coccinellidae	
*Scymnus ater* Kugelann, 1794	SV, EN, LA, BY, PL
*Scymnus limbatus* Stephens, 1832	SV, EN, LA, BY, PL, DK
Corylophidae	
*Arthrolips obscura* (R.F. Sahlberg, 1833)	SV, LA, PL
Cryptophagidae	
*Atomaria alpina* Heer, 1841	SV, EN, LA, BY, PL, KR
*Atomaria apicalis* Erichson, 1846	SV, EN, LA, BY, PL, KR, DK
*Atomaria barani* Brisout, 1863	SV, EN, LA, BY, PL, KR, DK
*Atomaria bella* Reitter, 1875	SV, EN, LA, PL, DK
*Atomaria nigriventris* Stephens, 1830	SV, EN, LA, BY, PL, KR, DK
*Atomaria peltata* Kraatz, 1853	SV, EN, LA, BY, PL, KR, DK
*Atomaria rubida* Reitter, 1875	SV, LA, PL, KR, DK
*Atomaria zetterstedti* (Zetterstedt, 1838)	SV, EN, LA, PL, DK
*Caenoscelis subdeplanata* Brisout, 1882	SV, EN, LA, BY, PL, KR, DK
*Cryptophagus confertus* Casey, 1900	SV, EN, LA, PL, KR, DK
*Cryptophagus confusus* Bruce, 1934	SV, EN, LA, PL, KR, DK
*Cryptophagus cylindrus* Kiesenwetter, 1858	SV, EN, LA, BY, PL, KR, DK
*Cryptophagus lapponicus* Gyllenhal, 1827	SV, EN, LA, BY, PL, KR, DK
*Cryptophagus lysholmi* Munster, 1932	SV, EN, LA
*Cryptophagus schmidti* Sturm, 1845	EN, LA, PL
*Cryptophagus subdepressus* Gyllenhal, 1827	SV, EN, LA, BY, PL, KR, DK
*Curelius exiguus* (Erichson, 1846)	SV, EN, LA, BY, PL, DK
*Micrambe bimaculata* (Panzer, 1798)	SV, EN, LA, BY, PL, KR, DK
*Micrambe lindbergorum* Bruce, 1934	SV, EN, LA, KR, DK
*Micrambe longitarsis* J.R. Sahlberg, 1900	SV, EN, LA, KR, DK
*Micrambe villosus* (Heer, 1841)	SV, EN, LA, BY, PL, KR, DK
Curculionidae	
*Acalles misellus* Boheman, 1844	SV, EN, LA, PL, DK
*Anthonomus ulmi* (DeGeer, 1775)	SV, EN, LA, BY, PL, KR, DK
*Barypeithes araneiformis* (Schrank, 1781)	SV, EN, PL, DK
*Ceutorhynchus pyrrhorhynchus* (Marsham, 1802)	SV, EN, BY, PL, DK
*Hylurgops glabratus* (Zetterstedt, 1828)	SV, EN, LA, BY, PL, DK
*Kyklioacalles roboris* (Curtis, 1834)	SV, PL, DK
*Otiorhynchus coecus* Germar, 1824	SV, PL, DK
*Phyllobius thalassinus* Gyllenhal, 1834	BY, PL
*Phyllobius viridicollis* (Fabricius, 1792)	SV, EN, LA, BY, PL, DK
*Rhinusa thapsicola* (Germar, 1821)	SV, LA, PL, DK
*Scolytus laevis* Chapuis, 1869	SV, EN, LA, BY, PL, DK
*Sitona lateralis* Gyllenhal, 1834	SV, EN, LA, BY, PL, DK
*Trachyphloeus spinimanus* Germar, 1824	SV, EN, LA, BY, PL, DK
*Tychius aureolus* Kiesenwetter, 1851	EN, LA, BY, PL, DK
Dermestidae	
*Trinodes hirtus* (Fabricius, 1781)	SV, LA, BY, PL, KR, DK
*Trogoderma variabile* Ballion, 1878	SV, LA
Dytiscidae	
*Nebrioporus canaliculatus* (Lacordaire, 1835)	SV, PL, DK
*Oreodytes sanmarkii* (C.R. Sahlberg, 1826)	SV, BY, PL, KR, DK
*Stictotarsus duodecimpustulatus* (Fabricius, 1792)	SV, DK
Elateridae	
*Ampedus hjorti* (Rye, 1905)	SV, LA, PL, DK
*Cardiophorus ebeninus* (Germar, 1824)	EN, LA, BY, PL
*Melanotus crassicollis* (Erichson, 1841)	EN, LA, BY, PL
*Zorochros minimus* (Lacordaire, 1835)	SV, EN, LA, BY, PL, KR, DK
Histeridae	
*Plegaderus dissectus* Erichson, 1839	SV, PL, DK
Hydrophilidae	
*Enochrus fuscipennis* (Thomson, 1884)	SV, LA, DK
*Enochrus quadripunctus* (Herbst, 1797)	SV, EN, LA, BY, PL, DK
Kateretidae	
*Brachypterolus linariae* (Stephens, 1830)	SV, EN, LA, BY, PL, KR, DK
Latridiidae	
*Corticaria saginata* Mannerheim, 1844	SV, EN, LA, BY, PL, DK
*Enicmus brevicornis* (Mannerheim, 1844)	SV, EN, LA, PL
Leiodidae	
*Agathidium plagiatum* (Gyllenhal, 1810)	SV, LA, PL
*Leiodes ferruginea* (Fabricius, 1787)	SV, EN, LA, BY, PL, DK
Melandryidae	
*Abdera flexuosa* (Paykull, 1799	SV, EN, LA, BY, PL, KR, DK
*Zilora ferruginea* (Paykull, 1798)	SV, EN, LA, BY, PL
Meloidae	
*Apalus bimaculatus* (Linnaeus, 1761)	SV, EN, LA, PL, DK
Melyridae	
*Aplocnemus impressus* (Marsham, 1802)	SV, EN, LA, BY, PL, DK
*Dasytes obscurus* Gyllenhal, 1813	SV, EN, LA, BY, PL
Nitidulidae	
*Acanthogethes solidus* (Kugelann, 1794)	SV, LA, BY, PL, DK
*Brassicogethes coeruleoivirens* (Förster, 1849)	SV, EN, LA, BY, PL, KR, DK
*Brassicogethes czwalinai* (Reitter, 1871)	SV, EN, LA, PL
*Brassicogethes subaeneus* (Sturm, 1845)	SV, EN, LA, BY, PL, DK
*Carpophilus marginellus* Motschulsky, 1858	SV, EN, LA, BY, PL, KR, DK
*Epuraea fageticola* Audisio, 1991	EN, LA, BY, PL, KR, DK
*Epuraea longiclavis* Sjöberg, 1939	SV, EN, LA, PL, KR, DK
*Epuraea longula* Erichson, 1845	SV, EN, LA, BY, PL, KR, DK
*Epuraea muehli* Reitter, 1908	SV, EN, LA, BY, PL, KR, DK
*Epuraea placida* Mäklin, 1853	SV, EN, LA, BY, PL, KR, DK
*Epuraea silacea* (Herbst, 1784)	SV, EN, LA, BY, PL, KR, DK
*Lamiogethes serripes* (Gyllenhal, 1827)	SV, EN, BY, PL, KR, DK
*Meligethes atratus* (Olivier, 1790)	SV, LA, BY, PL, KR, DK
*Meligethes flavimanus* Stephens, 1830	SV, EN, LA, BY, PL, KR, DK
*Pocadius adustus* Reitter, 1888	SV, EN, LA, BY, PL, KR, DK
*Sagittogethes ovatus* (Sturm, 1845)	SV, EN, LA, BY, PL, KR, DK
*Sagittogethes umbrosus* (Sturm, 1845)	SV, EN, LA, BY, PL, KR, DK
*Thymogethes exilis* (Sturm, 1845)	SV, EN, LA, BY, PL, DK
*Thymogethes gagathinus* (Erichson, 1845)	SV, EN, LA, BY, PL, KR, DK
*Thymogethes lugubris* (Sturm, 1845)	SV, EN, LA, BY, PL, KR, DK
Phalacridae	
*Phalacrus corruscus* (Panzer, 1797)	SV, EN, LA, BY, PL, DK
Ptinidae	
*Anobium thomsoni* (Kraatz, 1881)	SV, EN, LA, BY, PL
*Ptinus dubius* Sturm, 1837	SV, LA, PL, DK
*Ptinus lichenum* Marsham, 1802	SV, PL, DK
*Ptinus podolicus* Iablokoff-Khnzorian & Karapetyan, 1991	SV, EN, LA, BY, PL, DK
*Ptinus tectus* Boieldieu, 1856	SV, PL, DK
Ptiliidae	
*Acrotrichis thoracica* (Waltl, 1838)	SV, LA, BY, PL, DK
*Acrotrichis brevipennis* (Erichson, 1845)	SV, EN, LA, BY, PL, DK
Pythidae	
*Pytho kolwensis* Sahlberg, 1833	SV, EN, LA, BY, PL
Rhysodidae	
*Rhysodes sulcatus* (Fabrcius, 1787)	SV, BY, PL
Scarabeidae	
*Rhysothorax rufus* (Fabricius, 1792)	SV, LA, PL, DK
Scirtidae	
*Elodes pseudominuta* Klausnitzer, 1971	LA, BY, PL
Scraptiidae	
*Anaspis melanostoma* Costa, 1854	PL, DK
Silvanidae	
*Oryzaephilus mercator* (Fauvel, 1889)	SV, EN, LA, BY, PL, DK
Staphylinidae	
*Acrulia inflata* (Gyllenhal, 1813)	SV, EN, LA, BY, PL, DK
*Arrhenopeplus tesserula* (Curtis, 1828)	SV, BY, PL, DK
*Batrisodes hubenthali* Reitter, 1913	SV, EN, PL
*Batrisus formicarius* Aubé, 1833	SV, PL
*Brachygluta sinuata* (Aubé, 1833	SV, PL, DK
*Bryaxis nodicornis* (Aubé, 1833)	BY, PL
*Bythinus securiger* (Reichenbach, 1816)	PL
*Carpelimus despectus* (Baudi, 1870)	SV, EN, LA, PL, DK
*Cephennium thoracicum* O.F. Müller & Kunze, 1822	LA, PL, DK
*Euplectus bonvouloiri narentinus* Reitter, 1881	SV, DK
*Euplectus kirbii revelierei* Reitter, 1884	EN, LA, PL
*Geodromicus plagiatus* (Fabricius, 1798)	SV, EN, LA, PL, DK
*Gyrophaena polita* Gravenhorst, 1802	PL
*Ischnoglossa elegantula* (Mannerheim, 1830)	SV, EN
*Leptophius flavocinctus* (Hochhuth, 1849)	LA, PL
*Nevraphes ruthenus* Machulka, 1925	SV, DK
*Nevraphes talparum* Lokay, 1920	SV, PL
*Nevraphes rubicundus* (Schaum, 1841)	PL
*Parocyusa rubicunda* (Erichson, 1837)	SV, EN, LA, BY, DK
*Phacophallus parumpunctatus* (Gyllenhal, 1827)	SV, EN, LA, BY, PL, DK
*Philhygra volans* (W.Scriba, 1859)	SV, EN, LA, DK
*Plectophloeus nitidus* (Fairmaire, 1857)	SV, EN, BY, PL, DK
*Plectophloeus nubigena* (Reitter, 1876)	SV, BY, PL, DK
*Pronomaea rostrata* Erichson, 1837	PL
*Saulcyella schmidtii* (Märkel, 1844)	SV, BY, PL, KR, DK
*Scaphisoma boreale* Lundbland, 1952	SV, LA, BY, PL
*Scaphisoma subalpinum* Reitter, 1881	SV, EN, LA, BY, PL
*Schistoglossa aubei* (Brisout, 1860)	SV, EN, LA, BY, PL, DK
*Sepedophilus wankowiczi* (Pandellé, 1869)	LA, BY
*Stenus incanus* Erichson, 1839	EN, LA, PL
*Stenus picipes* (Stephens, 1833)	SV, EN, PL, DK
*Xylodromus depressus* (Gravenhorst, 1802)	SV, EN, LA, PL, DK
Stenotrachelidae	
*Scotodes annulatus* Eschscholtz, 1818	EN, LA, BY, KR
Tenebrionidae	
*Gnatocerus cornutus* Fabricius, 1798	SV, EN, LA, PL, DK
Tetratomidae	
*Eustrophus dermestoides* (Fabricius, 1793)	EN, LA, BY, PL, KR
Zopheridae	
*Synchita variegata* Hellwig, 1792	SV, EN, LA, BY, PL, DK

**Table 4.** The list of beetle species excluded from the list of Lithuanian beetles.

**Family and species name**	**First notification**	**Disproved**
Anthribidae		
*Bruchela suturalis* (Fabricius, 1793)	Slifverberg 2004	In current paper
Cantharidae		
*Cratosilis denticollis* (Schummel, 1844)	Roubal 1910	In current paper
Carabidae		
*Anisodactylus signatus* (Panzer, 1797)	Gaidienė 1993	In current paper
*Asaphidion curtum* (Heyden, 1870)	Tamutis and Ferenca 2006	In current paper
*Bradycellus csikii* Laczó, 1912	Tamutis et al. 2008	In current paper
*Carabus scheidleri* Panzer, 1799	Pileckis 1960	In current paper
*Chlaenius festivus* (Panzer, 1796)	Kirschenhofer 2003	In current paper
*Chlaenius spoliatus* (P. Rossi, 1792)	Pileckis and Monsevičius 1995	Tamutis et al. 2008
*Cryptophonus melancholicus* (Dejean, 1829)	Tamutis and Ferenca 2006	Tamutis et al. 2008
*Cymindis axilliaris* (Fabrcius, 1794)	Pileckis 1960	Pileckis and Monsevičius 1995
*Harpalus atratus* Latreille, 1804	Pileckis 1960	In current paper
*Harpalus caspius* (Steven, 1806)	Pileckis and Monsevičius 1995	In current paper
*Harpalus dimidiatus* (P. Rossi, 1790)	Pileckis 1960	Pileckis and Monsevičius 1995
*Ocydromus coeruleus* (Audient-Serville, 1812)	Ferenca et al. 2002	Ferenca 2003
*Ocydromus decorus* (Panzer, 1799)	Marggi et al. 2003	In current paper
*Ocydromus modestus* (Fabricius, 1801)	Marggi et al. 2003	In current paper
*Ocydromus tibialis* (Duftschmid, 1812)	Marggi et al. 2003	In current paper
*Odontium foraminosum* (Sturm, 1825)	Marggi et al. 2003	In current paper
*Sinechostictus elongatus* (Dejean, 1831)	Pileckis 1960	In current paper
Cerambycidae		
*Acanthocinus reticulatus* (Razoumov, 1789)	Pileckis 1959	Pileckis 1960
*Anastrangalia sequensi* (Reitter, 1898)	Stanilisówna 1939	Pileckis 1960
*Clytus rhamni* (Germar, 1817)	Pileckis 1959	Pileckis 1960
*Isotomus comptus* (Mannerheim, 1825)	Gaidienė 1993	In current paper
*Isotomus speciosus* (Schneider, 1787)	Pileckis 1959	Pileckis 1960
*Leptura aurulenta* Fabricius, 1792	Pileckis 1963b	In current paper
*Leiopus femoratus* Fairmaire, 1859	Ferenca 2004	In current paper
*Paracorymbia fulva* (DeGeer, 1775)	Gaidienė 1993	In current paper
Chrysomelidae		
*Aphthona flaviceps* Allard, 1859	Šurkus and Gaurilčikienė 2002	In current paper
*Cassida azurea* Fabricius, 1801	Tamutis 2003	In current paper
*Cryptocephalus virens* Suffrian, 1847	Pileckis 1960	Pileckis and Monsevičius 1997
*Epitrix cucumeris* (Harris, 1851)	Pileckis and Vengeliauskaitė 1996	In current paper
*Longitarsus minusculus* (Foudras, 1860)	Putele 1972	Pileckis and Monsevičius 1997
*Pilemostoma fastuosa* (Schaller, 1783)	Šablevičius 2000a	In current paper
Coccinellidae		
*Henosepilachna vigintioctomaculata* (Motschulsky, 1857)	Šablevičius 2000b	In current paper
Curculionidae		
*Acalles parvulus* Boheman, 1837	Pileckis 1976a	Pileckis and Monsevičius 1997
*Cionus olivieri* Rosenschöld, 1838	Tamutis 2003	In current paper
*Hadroplonthus trimaculatus* (Germar, 1824)	Tamutis 1996	In current paper
*Larinus iaceae* (Fabricius, 1775)	Gaidienė 1993	In current paper
*Omiamima concinna* (Boheman, 1834)	Pileckis 1976a	In current paper
*Otiorhynchus gemmatus* (Scopoli, 1763)	Pileckis 1960	In current paper
*Psallidium maxillosum* (Fabricius, 1792)	Eichwald 1830	In current paper
*Pseudomyllocerus canescens* (Germar, 1824)	Pileckis 1960	In current paper
*Pseudorchestes cinereus* (Fåhraeus, 1843)	Pileckis 1976a	Pileckis and Monsevičius 1997
*Xyleborus dryographus* (Ratzeburg, 1837)	Ferenca 2006b	In current paper
Dytiscidae		
*Agabus biguttulus* (Thomson, 1867)	Gaidienė 1993	In current paper
*Ilybius wasastjernae* (C.R. Sahlberg, 1824)	Monsevičius 1998	In current paper
Elateridae		
*Ctenicera cuprea* (Fabricius, 1775)	Monsevičius 1988b	Tamutis et al. 2010
Geotrupidae		
*Lethrus apterus* (Laxmann, 1770)	Pileckis 1960	Pileckis 1960
Hydraenidae		
*Ochthebius metallescens* Rosenhauer, 1847	Ogijewicz 1933	In current paper
Hydrophilidae		
*Helochares lividus* (Forster, 1771)	Pileckis 1976a	In current paper
*Helophorus pumilio* Erichson, 1837	Lindeman 1871	In current paper
*Helophorus redtenbacheri* Kuwert, 1885	Lindeman 1871	In current paper
Leiodidae		
*Sogda ciliaris* (Thomson, 1874)	Perreau 2004	In current paper
Nitidulidae		
*Ipidia sexguttata* (R.F. Sahlberg, 1834)	Wankowicz 1867a	In current paper
Scarabaeidae		
*Anisoplia austriaca* (Herbst, 1783)	Pileckis 1968a	Pileckis and Monsevičius 1995
*Bodilus punctipennis* (Erichson, 1848)	Gaidienė 1993	In current paper
*Chilothorax paykulli* (Bedel, 1908)	Gaidienė 1993	In current paper
*Oromus alpinus* (Scopoli, 1763)	Mazurowa and Mazur 1939	Pileckis 1960
*Osmoderma eremita* (Scopoli, 1763)	Pileckis 1960	Audisio et al. 2007
Staphylinidae		
*Amauronyx maerkelii* (Aubé, 1844)	Pileckis 1976a	Pileckis and Monsevičius 1995
*Taxicera deplanata* Gravenhorst, 1802	Pileckis 1976a	In current paper
Tenebrionidae		
*Pedinus femoralis* (Linnaeus, 1767)	Pileckis and Monsevičius 1997	In current paper

**Table 5.** Reported and expected species number of beetles in Lithuania.

*Family name*	*Number of species*				*Family name*	*Number of species*			
	*Reported*		*Expected*			*Reported*		*Expected*	
	*sum*	*%*	*sum*	*%*		*sum*	*%*	*sum*	*%*
Aderidae	3	43	4	57	Latridiidae	42	66	22	34
Agyrtidae	0	0	3	100	Leiodidae	75	62	45	38
Alexiidae	1	100	0	0	Limnichidae	3	75	1	25
Anthicidae	12	67	6	33	Lucanidae	6	86	1	14
Anthribidae	10	67	5	33	Lymexilidae	3	100	0	0
Attelabidae	19	76	6	24	Megalophodidae	4	100	0	0
Biphylidae	0	0	2	100	Melandryidae	18	62	11	38
Boridae	1	100	0	0	Meloidae	7	70	3	30
Bostrichidae	4	80	1	20	Melyridae	26	68	12	32
Bothrideridae	2	29	5	71	Monotomidae	15	65	8	35
Brachyceridae	11	92	1	8	Mordellidae	16	47	18	53
Brentidae	67	66	35	34	Mycetophagidae	12	86	2	14
Buprestidae	45	80	11	20	Mycteridae	0	0	1	100
Byrrhidae	12	75	4	25	Nemonychidae	2	100	0	0
Byturidae	2	100	0	0	Nitidulidae	93	84	17	16
Cantharidae	39	68	18	32	Nosodendridae	1	100	0	0
Carabidae	323	82	69	18	Noteridae	2	100	0	0
Cerambycidae	136	83	27	17	Ochodaeidae	0	0	1	100
Cerylonidae	5	100	0	0	Oedemeridae	15	71	6	29
Chrysomelidae	317	84	60	16	Orsodacnidae	1	100	0	0
Cisidae (Ciidae)	27	71	11	29	Phalacridae	12	60	8	40
Clambidae	6	75	2	25	Phloeophilidae	0	0	1	100
Cleridae	10	71	4	29	Phloeostichidae	0	0	1	100
Coccinellidae	54	84	10	16	Pyrochroidae	3	100	0	0
Corylophidae	8	73	3	27	Pythidae	2	100	0	0
Cryptophagidae	83	75	28	25	Prostomidae	0	0	1	100
Cucujidae	3	60	2	40	Psephenidae	1	100	0	0
Curculionidae	456	75	150	25	Ptiliidae	22	32	48	68
Dascilidae	1	100	0	0	Ptinidae	48	58	35	42
Dermestidae	28	61	18	39	Rhipiphoridae	2	67	1	33
Derodontidae	0	0	1	100	Rhysodidae	1	100	0	0
Drilidae	1	100	0	0	Salphingidae	9	64	5	36
Dryophthoridae	3	75	1	25	Scarabaeidae	85	82	19	18
Dryopidae	8	80	2	20	Scirtidae	16	84	3	16
Dytiscidae	112	84	22	16	Scraptiidae	8	40	12	60
Elateridae	74	70	31	30	Silphidae	19	86	3	14
Elmidae	10	63	6	37	Silvanidae	9	82	2	18
Endomychidae	5	71	2	29	Sphaeritidae	1	100	0	0
Erotylidae	8	67	4	33	Sphaeriusidae	1	100	0	0
Eucinetidae	1	100	0	0	Sphindidae	2	100	0	0
Eucnemidae	8	42	11	58	Staphylinidae	812	66	418	34
Geotrupidae	6	86	1	14	Stenotrachelidae	1	100	0	0
Gyrinidae	12	80	3	20	Tenebrionidae	48	66	25	34
Haliplidae	18	90	2	10	Tetratomidae	5	83	1	17
Heteroceridae	7	58	5	42	Throscidae	2	22	7	78
Histeridae	56	78	16	28	Trachypachidae	0	0	1	100
Hydraenidae	17	57	13	43	Trogidae	3	100	0	0
Hydrophilidae	73	76	23	24	Trogossitidae	7	78	2	22
Kateretidae	10	91	1	9	Zopheridae	8	67	4	33
Laemoploeidae	5	31	11	69					
Lampyridae	2	100	0	0	Total	3597	72	1390	28

## List of species

**MYXOPHAGA Crowson, 1955**.

**SPHAERIOIDEA Erichson, 1845**.

**SPHAERIUSIDAE Erichson, 1845** = MICROSPORIDAE Crotch, 1873. (Sphaeriidae)

*Sphaerius* **Waltl, 1838** = *Microsporus* Kolenati, 1846.

*acaroides* **Waltl, 1838** = *obsidianus* (Kolenati, 1846). Pileckis and Monsevičius 1995; Silfverberg 2004; Löbl 2009.

**ADEPHAGA Crowson, 1955**.

**GYRINIDAE Latreille, 1810**.

**Gyrininae** **Latreille, 1810**.

**Gyrinini Latreille, 1810**.

*Aulongyrus* **Régimbart, 1883**.

[*concinnus* **(Klug, 1834)**]. Known in northeastern Poland (Burakowski et al. 1976).

*Gyrinus* **O.F. Müller, 1764**.

*aeratus* **Stephens, 1835**. Pileckis and Monsevičius 1995; Monsevičius 1998; Barševskis 2001a; Silfverberg 2004; Barševskis et al. 2005; Alekseev 2010a.

*caspius* **Ménétriés, 1832**. Barševskis 2001a; Mazzoldi 2003, 2009; Silfverberg 2004; Alekseev 2010a.

[*colymbus* **Erichson, 1837**]. Known in Kaliningrad region (Alekseev 2010a), eastern Belarus (Alexandrovitch et al. 1996) and Denmark (Mazzoldi 2009).

*distinctus* **Aubé, 1836**. Ogijewicz 1933; Pileckis 1960, 1976a, b, 1979; Bercio and Folwaczny 1979; Silfverberg 1992, 2004; Pileckis and Monsevičius 1995; Mazzoldi 2003; Alekseev 2010a.

*marinus* **Gyllenhal, 1808**. Ogijewicz 1933; Pileckis 1960, 1976a; Silfverberg 1992, 2004; Gaidienė 1993; Pileckis and Monsevičius 1995; Monsevičius 1997; Ferenca 2006b; Kovács et al. 2008; Alekseev 2010a.

*minutus* **Fabricius, 1798**. Ogijewicz 1933; Pileckis 1960, 1976a; Silfverberg 1992, 2004; Gaidienė 1993; Pileckis and Monsevičius 1995; Monsevičius 1997; Ferenca 2006b; Alekseev 2010a.

*natator* **(Linnaeus, 1758)**. Ogijewicz 1933; Pileckis 1960, 1976a; Lešinskas and Pileckis 1967; Zajančkauskas and Pileckis 1968; Silfverberg 1992, 2004; Gaidienė 1993; Pileckis and Monsevičius 1995; Monsevičius 1997; Šablevičius 2000b, 2011; Gliaudys 2001; Ferenca 2006b; Mazzoldi 2009; Alekseev 2010a.

*opacus* **C.R. Sahlberg, 1819**. Barševskis 2001a; Silfverberg 2004; Alekseev 2010a.

*paykulli* **G. Ochs, 1927**. Ogijewicz 1933; Pileckis 1960, 1976a; Silfverberg 1992, 2004; Pileckis and Monsevičius 1995; Šablevičius 2003a; Alekseev 2010a.

*pullatus* **Zaitzev, 1908**. Barševskis, 2001; Silfverberg 2004; Alekseev 2010a.

*substriatus* **Stephens, 1828**. Monsevičius 1986a, 1997; Silfverberg 1992, 2004; Pileckis and Monsevičius 1995; Alekseev 2010a.

[*suffriani* **W. Scriba, 1855**]. Known in Baltic Sea coast region in Poland (Burakowski et al. 1976), Kaliningrad region (Alekseev 2010a), southern Sweden (Lundberg and Gustafsson 1995), Denmark (Mazzoldi 2009), Estonia (Rutanen 2004).

*urinator* **Illiger, 1807**. Pileckis 1960, 1976a, b, 1979; Zajančkauskas and Pileckis 1968; Silfverberg 1992, 2004; Pileckis and Monsevičius 1995; Monsevičius 1997; Barševskis et al. 2005; Ferenca 2006b; Alekseev 2010a.

**Orectochilini Régimbart, 1882**.

*Orectochilus* **Dejean, 1833**.

*villosus* **(O.F. Müller, 1776)**. Ogijewicz 1933; Pileckis 1960, 1976a; Silfverberg 1992, 2004; Pileckis and Monsevičius 1995; Mazzoldi 2003, 2009; Kovács et al. 2008; Alekseev 2010a; Šablevičius 2011.

**TRACHYPACHIDAE Thomson, 1857**.

**Trachypachinae Thomson, 1857**.

*Trachypachus* **Motschulsky, 1844**.

[*zetterstedtii* **(Gyllenhal, 1827)**].Known in Latvia (Barševskis 2003).

**RHYSODIDAE Laporte, 1840**.

**Rhysodini Laporte, 1840**.

*Rhysodes* **Germar, 1822**.

*sulcatus* **(Fabricius, 1787)**.# 1. Ogijewicz 1933; Pileckis 1960.

**CARABIDAE Latreille, 1802**.

**Nebriinae Latreille, 1802**.

**Nebriini Laporte, 1834**.

*Leistus* **Frölich, 1799**.

*ferrugineus* **(Linnaeus, 1758)**. Ogijewicz 1933; Pileckis 1960, 1976a; Sharova and Grüntal 1973; Silfverberg 1992, 2004; Gaidienė 1993; Pileckis and Monsevičius 1995; Monsevičius 1997, 2001; Šablevičius 2000b; Gliaudys 2001; Farkač and Janata 2003; Tamutis 2005a; Ferenca 2006b; Tamutis et al. 2007; Alekseev 2008a, c; Vigna Taglianti 2009.

*piceus* **Frölich, 1799**. Šablevičius and Ferenca 1995; Silfverberg 1996, 2004; Monsevičius 1998; Šablevičius 2000a, b, 2003a, b; Farkač and Janata 2003; Vaivilavičius 2008; Alekseev 2008c; Vigna Taglianti 2009.

*rufomarginatus* **(Duftschmid, 1812)**. Barševskis 2001a, 2003; Ferenca et al. 2002; Silfverberg 2004; Vaivilavičius 2008; Alekseev 2008c; Vigna Taglianti 2009; Ivinskiset al. 2009.

*terminatus* **(Panzer, 1793)** = *rufescens* (Fabricius, 1775) nec (Ström, 1768).Ogijewicz 1933; Pileckis 1960, 1976a; Bercio and Folwaczny 1979; Silfverberg 1992, 2004; Gaidienė 1993; Pileckis and Monsevičius 1995; Monsevičius 1997, 2001; Farkač and Janata 2003; Žiogas and Zolubas 2005; Ivinskis et al. 2008; Alekseev 2008c; Vigna Taglianti 2009.

*Nebria* **Latreille, 1802**.

*brevicollis* **(Fabricius, 1792)**.Ogijewicz 1933; Pileckis 1960, 1963b, 1976a; Zajančkauskas and Pileckis 1968; Silfverberg 1992, 2004; Gaidienė 1993; Pileckis and Monsevičius 1995; Monsevičius 1997; Ferenca et al. 2002; Farkač and Janata 2003; Žiogas and Zolubas 2005; Ferenca 2006b; Žiogas and Vaičikauskas 2007a, b; Alekseev 2008c; Vigna Taglianti 2009.

*livida* **(Linnaeus, 1758)**. Jakobson 1905-1915; Ogijewicz 1933; Pileckis 1960, 1976a; Lešinskas and Pileckis 1967; Bercio and Folwaczny 1979; Silfverberg 1992, 2004; Gaidienė 1993; Pileckis and Monsevičius 1995; Monsevičius 1997; Gliaudys 2001; Ferenca 2006b; Alekseev 2008c.

*rufescens* **(Ström, 1768)** = *gyllenhali* (Schönherr, 1806). # 2. Pileckis 1968b, 1970a, 1976a, b, 1979; Silfverberg 1992, 2004; Pileckis and Monsevičius 1995; Alekseev 2008c.

[*salina* **Fairmaire & Laboulbène, 1854**]. Known in southern Sweden (Lundberg and Gustafsson 1995), Denmark (Silfverberg 1992, 2004).

**Notiophilini Motschulsky, 1850**.

*Notiophilus* **Duméril, 1806**.

*aesthuans* **Dejean, 1826** = pusillus Waterhouse, 1833 nec (Schreber, 1759).Ogijewicz 1933; Pileckis 1960, 1970a, 1976a; Silfverberg 1992, 2004; Pileckis and Monsevičius 1995; Barševskis 2001; Bousquet and Barševskis 2003; Alekseev 2005; Ferenca 2006b; Alekseev 2008c; Vigna Taglianti 2009.

*aquaticus* **(Linnaeus, 1758)**. Ogijewicz 1933; Pileckis 1960, 1976a; Sharova and Grüntal 1973; Silfverberg 1992, 2004; Gaidienė 1993; Pileckis and Monsevičius 1995; Monsevičius 1997; Gliaudys 2001; Bousquet and Barševskis 2003; Ferenca 2006b; Alekseev 2008c; Vigna Taglianti 2009.

*biguttatus* **(Fabricius, 1779)**. Roubal 1910; Ogijewicz 1933; Pileckis 1960, 1976a; Zajančkauskas and Pileckis 1968; Sharova and Grüntal 1973; Silfverberg 1992, 2004; Gaidienė 1993; Pileckis and Monsevičius 1995; Monsevičius 1997; Šablevičius 2000b; Bousquet and Barševskis 2003; Žiogas and Zolubas 2005; Ferenca 2006b; Lynikienė 2006; Vaivilavičius 2008; Alekseev 2008c; Vigna Taglianti 2009.

*germinyi* **Fauvel, 1863** = stipraisi Barševskis, 1993.Ogijewicz 1933; Mazurowa and Mazur 1939; Pileckis 1960, 1976a; Silfverberg 1992, 2004; Gaidienė 1993; Pileckis and Monsevičius 1995; Monsevičius 1997; Barševskis 2001a; Bousquet and Barševskis 2003; Alekseev 2008c; Vigna Taglianti 2009.

*palustris* **(Duftschmid, 1812)**. Ogijewicz 1933; Mazurowa and Mazur 1939; Pileckis 1960, 1976a; Sharova and Grüntal 1973; Silfverberg 1992, 2004; Gaidienė 1993; Pileckis and Monsevičius 1995; Monsevičius 1997; Šablevičius 2000b; Tamutis and Zolubas 2001; Bousquet and Barševskis 2003; Tamutis et al. 2004, 2007; Tamutis 2005a; Žiogas and Zolubas 2005; Ferenca 2006b; Lynikienė 2006; Dapkus and Tamutis 2007; Alekseev 2008c; Vigna Taglianti 2009.

[*rufipes* **Curtis, 1829**]. Known in southern Sweden (Lundberg and Gustafsson 1995), Denmark, Poland, Estonia, Kaliningrad region (Vigna Taglianti 2009).

**Pelophilini Kavanaugh, 1996**.

*Pelophila* **Dejean, 1821**.

[*borealis* **(Paykull, 1790)**]. Known in Estonia (Haberman 1968); Latvia (Barševskis 2003), central Sweden (Lundberg and Gustafsson 1995), northern Belarus (Alexandrovitch et al. 1996).

**Cicindelinae Latreille, 1802**.

**Cicindelini Latreille, 1802**.

*Cicindela* **Linnaeus, 1758**.

*campestris* **Linnaeus, 1758**. Eichwald 1830; Ogijewicz 1933; Mazurowa and Mazur 1939; Pileckis 1960, 1976a, 1982; Lešinskas and Pileckis 1967; Ivinskis et al. 1984; Silfverberg 1992, 2004; Gaidienė 1993; Pileckis and Monsevičius 1995; Monsevičius 1997; Šablevičius 2000b, 2011; Gliaudys 2001; Puchkov and Matalin 2003; Ferenca 2006b; Alekseev 2008c; Vigna Taglianti 2009.

*hybrida* **Linnaeus, 1758**. Ogijewicz 1933; Mazurowa and Mazur 1939; Pileckis 1960, 1976a, 1982; Lešinskas and Pileckis 1967; Ivinskis et al. 1984; Silfverberg 1992, 2004; Gaidienė 1993; Pileckis and Monsevičius 1995; Monsevičius 1997; Šablevičius 2000b, 2011; Gliaudys 2001; Puchkov and Matalin 2003; Ferenca 2006b; Alekseev 2008c; Vigna Taglianti 2009.

RDB*maritima* **Latreille & Dejean, 1822**. Pileckis 1963b, 1976a, b, 1979; Bercio and Folwaczny 1979; Balevičius 1992; Šablevičius 1995, 2011; Pileckis and Monsevičius 1995; Ferenca 2004; 2006a, b; Ivinskis and Rimšaitė 2005; Tamutis 2005b; Rašomavičius 2007; Alekseev 2008c, 2010b; Ivinskis et al. 2009; Vigna Taglianti 2009.

*sylvatica* **Linnaeus,** 1758. Eichwald 1830; Ogijewicz 1933; Pileckis 1960, 1976a, 1982; Lešinskas and Pileckis 1967; Ivinskis et al. 1984; Silfverberg 1992, 2004; Gaidienė 1993; Pileckis and Monsevičius 1995; Monsevičius 1997; Gliaudys 2001; Alekseev 2003, 2008c; Puchkov and Matalin 2003; Ferenca 2006b; Vigna Taglianti 2009; Šablevičius 2011.

*Cylindera* **Westwood 1831**.

*arenaria viennensis* **(Schrank, 1781)**. Ferenca et al. 2002; Barševskis 2003; Ivinskis et al. 2004a; Alekseev 2008c; Vigna Taglianti 2009.

*germanica* **(Linnaeus, 1758)**.Eichwald 1830 (*Cicindela*); Pileckis 1960, 1976a (*Cicindela*); Silfverberg 1992, 2004; Gaidienė 1993 (*Cicindela*); Pileckis and Monsevičius 1995 (*Cicindela*); Gliaudys 2001 (*Cicindela*); Inokaitis 2004; Tamutis 2005b, Tamutis et al. 2007; Alekseev 2008c; Vaivilavičius 2008; Vigna Taglianti 2009.

**Carabinae Latreille, 1802**.

**Carabini Latreille, 1802**.

*Calosoma* **Weber, 1801**.

*auropunctatum* **(Herbst, 1784)**. Pileckis 1968a, 1976a, 1982; Silfverberg 1992, 2004; Pileckis and Monsevičius 1995; Tamutis 1999, 2000, 2005a; Gliaudys 2001; Bousquet et al. 2003; Tamutis et al. 2007; Alekseev 2008c; Vigna Taglianti 2009.

RDB*inquisitor* **(Linnaeus, 1758)**. Pileckis 1968a, 1976a, b, 1979, 1982; Bercio and Folwaczny 1979; Balevičius 1992; Silfverberg 1992, 2004; Gaidienė 1993; Pileckis and Monsevičius 1995; Pileckis and Vengeliauskaitė 1996; Šablevičius 2000a, 2011; Gliaudys 2001; Ehnström et al. 2003; Bousquet et al. 2003; Ferenca 2004, 2006a; Ivinskis et al. 2004b, 2006, 2007; Tamutis 2005b; Rašomavičius 2007; Kriaučiūnienė and Zaplatkin 2007; Alekseev 2008c, 2010b; Vigna Taglianti 2009; Bačianskas 2009; Noreika 2009.

[*investigator* **(Illiger, 1798)**]. Known in southern Sweden (Lundberg and Gustafsson 1995), northeastern Poland (Burakowski et al. 1973), Kaliningrad region (Bercio and Folwaczny 1979, Vigna Taglianti 2009).

RDB*sycophanta* **(Linnaeus, 1758)**.# 3. Lentz 1879; Pileckis 1976a; Bercio and Folwaczny 1979; Pileckis and Monsevičius 1982, 1995; Balevičius 1992; Silfverberg 1992, 2004; Gaidienė 1993; Pileckis and Vengeliauskaitė 1996; Gliaudys 2001; Ehnström et al. 2003; Bousquet et al. 2003; Tamutis 2005b; Rašomavičius 2007; Alekseev 2008c, 2010b; Vigna Taglianti 2009; Šablevičius 2011.

*Callisthenes* **Fischer von Waldheim 1821**.

[*reticulatum* **(Fabricius, 1787)**]. # 4. Known in southern Sweden (Lundberg and Gustafsson 1995), northeastern Poland (Burakowski et al. 1973), western Belarus (Alexandrovitch et al. 1996), Kaliningrad region (Bercio and Folwaczny 1979).

*Carabus* **Linnaeus, 1758**.

*arcensis* **Herbst, 1784** = *arvensis* Herbst, 1784.Ogijewicz 1933; Mazurowa and Mazur 1939; Pileckis 1960, 1976a, 1982; Lešinskas and Pileckis 1967; Ivinskis et al. 1984; Silfverberg 1992, 2004; Gaidienė 1993; Pileckis and Monsevičius 1995; Monsevičius 1997; Šablevičius 2000b; Gliaudys 2001; Gedminas and Žiogas 2001, 2007; Bousquet et al. 2003; Ferenca 2006b; Lynikienė 2006; Žiogas et al. 2006; Žiogas and Vaičikauskas 2007b; Dapkus and Tamutis 2007, 2008a; Alekseev 2008c; Vigna Taglianti 2009.

[*auratus* **Linnaeus, 1761**]. Known in northeastern Poland (Burakowski et al. 1973), Denmark (Vigna Taglianti 2009).

*cancellatus tuberculatus* **Dejean, 1826**. Ogijewicz 1933; Mazurowa and Mazur 1939; Pileckis 1960, 1976a, 1982; Zajančkauskas and Pileckis 1968; Strazdienė 1976; Dvilevičius et al. 1988; Silfverberg 1992, 2004; Gaidienė 1993; Pileckis and Monsevičius 1995; Monsevičius 1997; Šablevičius 2000b; Tamutis 2000, 2005a; Gliaudys 2001; Bousquet et al. 2003; Tamutis et al. 2004, 2006, 2007; Ferenca 2006b; Lynikienė 2006; Žiogas and Vaičikauskas 2007b; Ivinskis et al. 2008; Alekseev 2008c; Vigna Taglianti 2009; Šablevičius 2011.

*clathratus* **Linnaeus, 1761**. Eichwald 1830; Ogijewicz 1933; Mazurowa and Mazur 1939; Pileckis 1960, 1976a, 1982; Lešinskas and Pileckis 1967; Zajančkauskas and Pileckis 1968; Sharova and Grüntal 1973; Ivinskis et al. 1984; Silfverberg 1992, 2004; Gaidienė 1993; Pileckis and Monsevičius 1995; Monsevičius 1997; Šablevičius 2000b; Gliaudys 2001; Bousquet et al. 2003; Ferenca 2003, 2006b; Ivinskis et al. 2004a; Alekseev 2008c; Vigna Taglianti 2009.

*convexus* **Fabricius, 1775**. Eichwald 1830; Ogijewicz 1933; Pileckis 1960, 1976a, 1982; Silfverberg 1992, 2004; Gaidienė 1993; Pileckis and Monsevičius 1995; Ferenca et al. 2002; Šablevičius 2003a, b; Bousquet et al. 2003; Žiogas and Zolubas 2005; Alekseev 2008c; Ivinskis et al. 2009; Vigna Taglianti 2009; Noreika 2009.

RDB*coriaceus* **Linnaeus, 1758**. Eichwald 1830; Jakobson 1905-1915; Pileckis 1960, 1976a, b, 1979, 1982; Lešinskas and Pileckis 1967; Strazdienė 1981, 1988; Balevičius 1992; Gaidienė and Ferenca 1992; Silfverberg 1992, 2004; Gaidienė 1993; Pileckis and Monsevičius 1995; Ivinskis et al. 1996b, 1997b, 1998b, 2000, 2004a, 2006, 2009; Ferenca 1996, 2006b; Auglys 1998; Gliaudys 2001; Bousquet et al. 2003; Tamutis 2005b; Rašomavičius 2007; Vaivilavičius 2008; Sruoga 2008; Alekseev 2008c, 2010b; Dapkus & Tamutis 2009; Vigna Taglianti 2009; Šablevičius 2011.

*glabratus* **Paykull, 1790**.Ogijewicz 1933; Mazurowa and Mazur 1939; Pileckis 1960, 1976a, 1982; Lešinskas and Pileckis 1967; Silfverberg 1992, 2004; Gaidienė 1993; Pileckis and Monsevičius 1995; Monsevičius 1997; Gliaudys 2001; Bousquet et al. 2003; Ferenca 2006b; Lynikienė 2006; Žiogas and Vaičikauskas 2007b; Ivinskis et al. 2008; Alekseev 2008c; Vigna Taglianti 2009.

*granulatus* **Linnaeus, 1758**. Eichwald 1830; Ogijewicz 1933; Pileckis 1960, 1976a, 1982; Zajančkauskas and Pileckis 1968; Strazdienė 1981; Dvilevičius et al. 1988; Silfverberg 1992, 2004; Gaidienė 1993; Pileckis and Monsevičius 1995; Monsevičius 1997; Šablevičius 2000b; Tamutis 2000, 2005a; Gliaudys 2001; Bousquet et al. 2003; Tamutis et al. 2004, 2006, 2007; Ferenca 2006b; Žiogas and Vaičikauskas 2007b; Vaivilavičius 2008; Ivinskis et al. 2008; Alekseev 2008c; Vigna Taglianti 2009.

*hortensis* **Linnaeus, 1758**. Eichwald 1830; Heyden 1903; Ogijewicz 1933; Mazurowa and Mazur 1939; Pileckis 1960, 1976a, 1982; Lešinskas and Pileckis 1967; Zajančkauskas and Pileckis 1968; Dvilevičius et al. 1988; Silfverberg 1992, 2004; Gaidienė 1993; Pileckis and Monsevičius 1995; Monsevičius 1997; Šablevičius 2000b; Gliaudys 2001; Bousquet et al. 2003; Žiogas and Zolubas 2005; Lynikienė 2006; Ferenca 2006b; Žiogas and Vaičikauskas 2007a, b; Dapkus and Tamutis 2007, 2008a; Vaivilavičius 2008; Ivinskis et al. 2008; Alekseev 2008c; Vigna Taglianti 2009.

RDB*intricatus* **Linnaeus, 1761**. Ferenca 1996; Ivinskis et al. 1996a, 1997b, 1998b, 2000, 2004a; Jonaitis et al. 2000; Šablevičius 2000b; Ferenca et al. 2002; Tamutis 2005b; Rašomavičius 2007; Alekseev 2008c, 2010b.

[*marginalis* **Fabricius, 1794**]. Known in northeastern Poland (Burakowski et al. 1973), eastern Belarus (Alexandrovitch et al. 1996), Kaliningrad region (Vigna Taglianti 2009).

*menetriesi* **(Faldermann in Hummel, 1827)**. Ogijewicz 1933; Mazurowa and Mazur 1939; Pileckis 1960, 1976a; Balevičius 1992; Silfverberg 1992, 2004; Pileckis and Monsevičius 1995; Monsevičius 1997; Bousquet et al. 2003; Tamutis 2005b; Ferenca 2006b; Vaivilavičius 2008; Ivinskis et al. 2008, 2009; Alekseev 2008c; Vigna Taglianti 2009.

*nemoralis* **O.F. Müller, 1764**. Ogijewicz 1933; Pileckis 1960, 1976a, 1982; Zajančkauskas and Pileckis 1968; Strazdienė 1981; Dvilevičius et al. 1988; Silfverberg 1992, 2004; Gaidienė 1993; Pileckis and Monsevičius 1995; Monsevičius 1997; Šablevičius 2000b; Tamutis 2000, 2005a; Bousquet et al. 2003; Tamutis et al. 2004, 2006, 2007; Ferenca 2006b; Žiogas and Vaičikauskas 2007b; Vaivilavičius 2008; Ivinskis et al. 2008; Alekseev 2008c; Vigna Taglianti 2009.

RDB*nitens* **Linnaeus, 1758**. Ogijewicz 1933; Pileckis 1960, 1976a, 1982; Zajančkauskas and Pileckis 1968; Balevičius 1992; Silfverberg 1992, 2004; Gaidienė 1993; Obelevičius 1994; Ostrauskas and Kubertavičius 1994; Šablevičius 1994, 2000b, 2003a, b, 2011; Pileckis and Monsevičius 1995; Ivinskis et al. 1996b, 2000, 2004a; Pankevičius 2000, 2007; Monsevičius 1997, 2001; Gliaudys 2001; Bousquet et al. 2003; Meržijevskis 2004; Dapkus 2004; Tamutis 2005b; Ferenca 2006b; Lopeta 2007a; (misprint as *Callisthenes coriaceus* L. in Lopeta 2007b); Rašomavičius 2007; Uselis et al. 2007; Butvila et al. 2007; Alekseev 2008c, 2010b; Vigna Taglianti 2009.

[*problematicus harcyniae* **Sturm, 1815**].Known in northeastern Poland (Burakowski et al. 1973), southern Sweden (Lundberg and Gustafsson 1995), Denmark (Vigna Taglianti 2009).

*scheidleri **Panzer, 1799**. # 5. Pileckis 1960.

*violaceus andrzejuscii* **Fischer von Waldheim, 1823**. Ogijewicz 1933; Mazurowa and Mazur 1939; Pileckis 1960, 1976a, 1982; Strazdienė 1988; Silfverberg 1992, 2004; Ferenca et al. 2002; Gaidienė 1993; Pileckis and Monsevičius 1995; Monsevičius 1997; Gliaudys 2001; Bousquet et al. 2003; Žiogas and Zolubas 2005; Ferenca 2006b; Lynikienė 2006; Dapkus and Tamutis 2007; Vaivilavičius 2008; Sruoga 2008; Alekseev 2008c; Vigna Taglianti 2009; Šablevičius 2011.

**Cychrini Laporte, 1834**.

*Cychrus* **Fabricius, 1794**.

*caraboides* **(Linnaeus, 1758)** *= rostratus* (Linnaeus, 1761). Heyden 1903; Ogijewicz 1933; Mazurowa and Mazur 1939; Pileckis 1960, 1976a; Lešinskas and Pileckis 1967; Sharova and Grüntal 1973; Dvilevičius et al. 1988; Silfverberg 1992, 2004; Gaidienė 1993; Pileckis and Monsevičius 1995; Monsevičius 1997; Šablevičius 2000b, 2011; Monsevičius 2001; Gliaudys 2001; Häckel 2003; Žiogas and Zolubas 2005; Lynikienė 2006; Ferenca 2006b; Dapkus and Tamutis 2007; Žiogas and Vaičikauskas 2007a, b; Ivinskis et al. 2008; Alekseev 2008c; Vigna Taglianti 2009.

**Loricerinae Bonelli, 1810**.

**Loricerini Bonelli, 1810**.

*Loricera* **Latreille, 1802**.

*pilicornis* **(Fabricius, 1775) =** *caerulescens* auct. nec (Linnaeus, 1758). Eichwald 1830; Ogijewicz 1933; Pileckis 1960, 1976a; Zajančkauskas and Pileckis 1968; Sharova and Grüntal 1973; Strazdienė 1976; Dvilevičius et al. 1988; Silfverberg 1992, 2004; Gaidienė 1993; Pileckis and Monsevičius 1995; Monsevičius 1997; Šablevičius 2000b; Tamutis 2000, 2005a; Tamutis and Zolubas 2001; Bousquet 2003a; Tamutis et al. 2004, 2006, 2007; Ferenca 2006b; Žiogas et al. 2006, 2009; Ivinskis et al. 2008; Alekseev 2008c; Vigna Taglianti 2009.

**Omophroninae Bonelli, 1810**.

**Omophronini Bonelli, 1810**.

*Omophron* **Latreille, 1802**.

*limbatum* **(Fabricius, 1776)**. Eichwald 1830; Pileckis 1960, 1976a; Gaidienė and Ferenca 1992; Silfverberg 1992, 2004; Gaidienė 1993; Pileckis and Monsevičius 1995; Hůrka 2003; Ferenca 2004, 2006b; Vaivilavičius 2008; Alekseev 2008a, c; Vigna Taglianti 2009; Ivinskis et al. 2009, Valainis 2010; Šablevičius 2011.

**Elaphrinae Latreille, 1802**.

**Elaphrini Latreille, 1802**.

*Blethisa* **Bonelli, 1810**.

*multipunctata* **(Linnaeus, 1758)**. Eichwald 1830; Jakobson 1905-1915; Pileckis 1960, 1970a, 1976a, b, 1979; Silfverberg 1992, 2004; Gaidienė 1993; Pileckis and Monsevičius 1995; Monsevičius 1997; Gliaudys 2001; Goulet 2003; Šablevičius 2003a; Ivinskis et al. 2004a; Ferenca 2006b; Alekseev 2008c; Vigna Taglianti 2009.

*Elaphrus* **Fabricius, 1775**.

[*angusticollis* **R.F. Sahlberg, 1844**]. Known in northwestern Belarus (Alexandrovitch et al. 1996), Latvia (Barševskis 2003).

*aureus* **O.F. Müller, 1821**. Pileckis 1968b, 1976a; Silfverberg 1992, 2004; Gaidienė 1993; Pileckis and Monsevičius 1995; Gedminas et al. 1996; Ferenca et al. 2002; Goulet 2003; Alekseev 2008c; Vaivilavičius 2008; Vigna Taglianti 2009.

*cupreus* **Duftschmid, 1812**. Ogijewicz 1933; Mazurowa and Mazur 1939; Pileckis 1960, 1976a; Zajančkauskas and Pileckis 1968; Sharova and Grüntal 1973; Silfverberg 1992, 2004; Gaidienė 1993; Pileckis and Monsevičius 1995; Monsevičius 1997; Šablevičius 2000b; Tamutis and Zolubas 2001; Goulet 2003; Ferenca 2006b; Vaivilavičius 2008; Ivinskis et al. 2008; Alekseev 2008c; Vigna Taglianti 2009.

*riparius* **(Linnaeus, 1758)**. Eichwald 1830; Heyden 1903; Ogijewicz 1933; Mazurowa and Mazur 1939; Pileckis 1960, 1968b, 1970a, 1976a, b, 1979; Lešinskas and Pileckis 1967; Sharova and Grüntal 1973; Silfverberg 1992, 2004; Gaidienė 1993; Pileckis and Monsevičius 1995; Monsevičius 1997; Šablevičius 2000b; Goulet 2003; Ferenca 2006b; Vaivilavičius 2008; Alekseev 2008c; Vigna Taglianti 2009.

*uliginosus* **Fabricius, 1792**. Pileckis 1960, 1976a; Bercio and Folwaczny 1979; Silfverberg 1992, 2004; Pileckis and Monsevičius 1995; Goulet 2003; Alekseev 2008c; Vigna Taglianti 2009.

**Scaritinae Bonelli, 1810**.

**Clivinini Rafinesque 1815**.

*Clivina* **Latreille, 1802**.

*collaris* **(Herbst, 1784)**. Eichwald 1830; Ogijewicz 1933; Pileckis 1960, 1976a; Sharova and Grüntal 1973; Silfverberg 1992, 2004; Gaidienė 1993; Pileckis and Monsevičius 1995; Monsevičius 1997; Balkenohl 2003; Ferenca 2006b; Alekseev 2008c.

*fossor* **(Linnaeus, 1758)**.Eichwald 1830; Ogijewicz 1933; Mazurowa and Mazur 1939; Pileckis 1960, 1976a; Zajančkauskas and Pileckis 1968; Sharova and Grüntal 1973; Strazdienė 1976; Silfverberg 1992, 2004; Gaidienė 1993; Pileckis and Monsevičius 1995; Monsevičius 1997; Šablevičius 2000b; Balkenohl 2003; Tamutis 2000, 2005a; Tamutis and Zolubas 2001; Tamutis et al. 2004, 2006, 2007; Ferenca 2006b; Alekseev 2008c; Vigna Taglianti 2009.

*Dyschirius* **Bonelli, 1810**.

*angustatus* **(Ahrens, 1830)**. Pileckis 1960, 1976a; Silfverberg 1992, 2004; Gaidienė 1993; Pileckis and Monsevičius 1995; Ferenca 2006b; Alekseev 2008c.

*digitatus* **(Dejean, 1825)**. Pileckis 1963b, 1976a; Silfverberg 1992, 2004; Pileckis and Monsevičius 1995; Alekseev 2008c.

*obscurus* **(Gyllenhal, 1827)**. Pileckis 1962, 1963b, 1970a, 1976a, b, 1979; Silfverberg 1992, 2004; Gaidienė 1993; Pileckis and Monsevičius 1995; Alekseev 2008a, c.

*thoracicus* **(P. Rossi, 1790) =** *arenosus* Stephens, 1827. Eichwald 1830; Jakobson 1905-1915; Roubal 1910; Pileckis 1960, 1976a; Sharova and Grüntal 1973; Silfverberg 1992, 2004; Gaidienė 1993; Pileckis and Monsevičius 1995; Monsevičius 1997; Ferenca 2006b; Alekseev 2008a, c.

*Dyschiriodes* **Jeannel 1941**.

*aeneus* **(Dejean, 1825)**.Ogijewicz 1933; Pileckis 1960, 1976a; Sharova and Grüntal 1973; Silfverberg 1992, 2004; Gaidienė 1993; Pileckis and Monsevičius 1995 (*Dyschirius*); Monsevičius 1997; Alekseev 2008c.

[*chalceus* **(Erichson, 1837)**]. Known in southern Sweden (Lundberg and Gustafsson 1995), Poland (Burakowski et al. 1973), Kaliningrad region, Estonia, Denmark (Vigna Taglianti 2009).

*globosus* **(Herbst, 1784)**. Eichwald 1830; Ogijewicz 1933; Mazurowa and Mazur 1939; Pileckis 1960, 1976a; Sharova and Grüntal 1973; Dvilevičius et al. 1988; Silfverberg 1992, 2004; Gaidienė 1993; Pileckis and Monsevičius 1995 (*Dyschirius*); Monsevičius 1997; Tamutis 2000; Tamutis et al. 2004; Ferenca 2006b; Alekseev 2008c.

*impunctipennis* **(Dawson, 1854)**. Pileckis 1976a, b, 1979; Bercio and Folwaczny 1979; Silfverberg 1992, 2004; Pileckis and Monsevičius 1995 (*Dyschirius*); Alekseev 2008c.

*intermedius* **(Putzeys, 1846)**. Lentz 1879; Pileckis 1976a; Bercio and Folwaczny 1979; Silfverberg 1992, 2004; Pileckis and Monsevičius 1995 (*Dyschirius*); Tamutis 2000, 2005a; Tamutis et al. 2004; Alekseev 2008c; Ivinskis et al. 2009.

*laeviusculus* **(Putzeys, 1846)**. Silfverberg 1992, 2004; Pileckis and Monsevičius 1995 (*Dyschirius*); Alekseev 2008c.

*neresheimeri* **(Wagner, 1915)**.Lentz 1879; Pileckis 1976a; Silfverberg 1992, 2004; Pileckis and Monsevičius 1995 (*Dyschirius*); Alekseev 2008c.

[*nigricornis* **(Motschulsky, 1844)** *= helleni* (P.W.J.Müller, 1922) *= septentrionum* (Munster, 1923)]. Known in northern Sweden (Lundberg and Gustafsson 1995), Latvia (Barševskis 2003), Kaliningrad Region, Estonia, (Vigna Taglianti 2009).

*nitidus* **(Dejean, 1825)**.Eichwald 1830; Sharova and Grüntal 1973; Pileckis 1976a; Silfverberg 1992, 2004; Pileckis and Monsevičius 1995 (*Dyschirius*); Monsevičius 1997; Ivinskis et al. 1998; Ferenca et al. 2002; Alekseev 2008c.

*politus* **(Dejean, 1825)**. Ogijewicz 1933; Pileckis 1960, 1976a; Silfverberg 1992, 2004; Gaidienė 1993; Pileckis and Monsevičius 1995 (*Dyschirius*); Tamutis 2000, 2005a; Tamutis et al. 2004; Ferenca 2006b; Alekseev 2008c.

[*salinus* **(Schaum, 1843)**]. Known in southern Sweden (Lundberg and Gustafsson 1995), coast of Baltic Sea in Poland (Burakowski et al. 1973), Latvia (Barševskis 2003), Kaliningrad Region, Estonia, Denmark (Vigna Taglianti 2009).

*tristis* **(Stephens, 1827) =** *luedersi* Wagner, 1915. Bercio and Folwaczny 1979; Pileckis and Monsevičius 1982, 1995 (*Dyschirius*); Silfverberg 1992, 2004; Alekseev 2008c.

**Broscinae Hope, 1838**.

**Broscini Hope, 1838**.

*Broscus* **Panzer, 1813**.

*cephalotes* **(Linnaeus, 1758)**. Heyden 1903; Roubal 1910; Ogijewicz 1933; Mazurowa and Mazur 1939; Pileckis 1960, 1976a; Lešinskas and Pileckis 1967; Zajančkauskas and Pileckis 1968; Sharova and Grüntal 1973; Silfverberg 1992, 2004; Gaidienė 1993; Pileckis and Monsevičius 1995; Monsevičius 1997; Šablevičius 2000b; Gliaudys 2001; Ferenca 2006b; Alekseev 2008c.

*Miscodera* **Eschscholtz, 1830**.

*arctica* **(Paykull, 1798)**. Pileckis 1968a, 1970a, 1976a, b, 1979, 1982; Ivinskis et al. 1984, 2004a; Silfverberg 1992, 2004; Gaidienė 1993; Pileckis and Monsevičius 1995; Monsevičius 1997; Ferenca et al. 2002; Alekseev 2008c.

**Trechinae Bonelli, 1810**.

**Trechini Bonelli, 1810**.

*Perileptus* **Schaum, 1860**.

[*aureolatus* **(Creutzer, 1799)**]. Known in Sweden (Lundberg and Gustafsson 1995), northeastern Poland (Burakowski et al. 1973), Latvia (Barševskis 2003), Belarus (Alexandrovitch et al. 1996).

*Trechus* **Clairville, 1806**.

*austriacus* **Dejean, 1831**. Pileckis and Monsevičius 1982, 1995; Silfverberg 1992, 2004; Moravec et al. 2003; Alekseev 2008c; Vigna Taglianti 2009.

*obtusus* **Erichson, 1837**.Ferenca et al. 2006, 2007; Ivinskis et al. 2009.

*quadristriatus* **(Schranck, 1781)**. Ogijewicz 1933; Pileckis 1960, 1976a; Zajančkauskas and Pileckis 1968; Sharova and Grüntal 1973; Dvilevičius et al. 1988; Silfverberg 1992, 2004; Gaidienė 1993; Pileckis and Monsevičius 1995; Šablevičius 2000b; Tamutis 2000, 2005a; Tamutis and Zolubas 2001; Moravec et al. 2003; Tamutis et al. 2004, 2006, 2007; Ferenca 2006b; Žiogas et al. 2006; Alekseev 2008c; Vigna Taglianti 2009; Ostrauskas and Ferenca 2010.

*rivularis* **(Gyllenhal, 1810)**. Pileckis and Monsevičius 1982, 1995 (*Epaphius*); Ivinskis et al. 1984; Silfverberg 1992, 2004; Monsevičius 1997; Moravec et al. 2003; Ivinskis et al. 2008; Alekseev 2008c; Vigna Taglianti 2009.

*rubens* **(Fabricius, 1792)**. Pileckis 1976a; Silfverberg 1992, 2004; Pileckis and Monsevičius 1995; Tamutis 1999, 2000, 2005a; Moravec et al. 2003; Alekseev 2008c; Vigna Taglianti 2009.

*secalis* **(Paykull, 1790)**. # 19. Roubal 1910; Ogijewicz 1933 (*Epaphius*); Pileckis 1960, 1976a (*Epaphius*); Sharova and Grüntal 1973; Dvilevičius et al. 1988; Silfverberg 1992, 2004; Gaidienė 1993; Pileckis and Monsevičius 1995 (*Epaphius*); Monsevičius 1997; Tamutis 2000, 2005a; Moravec et al. 2003; Tamutis et al. 2004; Vaivilavičius 2008; Ivinskis et al. 2008; Alekseev 2008c; Vigna Taglianti 2009.

*Trechoblemus* **Ganglbauer, 1891**.

*micros* **(Herbst, 1784)**. Ogijewicz 1933; Pileckis 1960, 1976a; Silfverberg 1992, 2004; Pileckis and Monsevičius 1995; Tamutis 2000, 2003, 2005a; Moravec et al. 2003; Alekseev 2008c; Vigna Taglianti 2009; Ivinskis et al. 2009.

*Blemus* **Dejean, 1821 =** *Lasiotrechus* Ganglbauer, 1892.

*discus* **(Fabricius, 1792)**. Ogijewicz 1933; Pileckis 1960, 1976a; Bercio and Folwaczny 1979; Silfverberg 1992, 2004; Gaidienė 1993; Pileckis and Monsevičius 1995; Monsevičius 1997; Tamutis 2000; Moravec et al. 2003; Tamutis et al. 2004; Alekseev 2008c; Vigna Taglianti 2009.

**Pagonini Laporte, 1834**.

*Pogonus* **Dejean, 1821**.

*chalceus* **(Marsham, 1802)**. # 6. Pileckis 1960, 1976a, b, 1979; Pileckis and Monsevičius 1995; Bousquet 2003b; Ivinskis and Rimšaitė 2005; Ferenca 2006b; Vigna Taglianti 2009.

[*luridipennis* **(Germar, 1822)**]. Known in Sweden (Lundberg and Gustafsson 1995), Poland (Burakowski et al. 1973), Latvia, Denmark, Kaliningrad region (Vigna Taglianti 2009).

**Bembidiini Stephens, 1827**.

*Asaphidion* **Gozis, 1886**.

[*austriacum* **Schweiger, 1975**]. Known in Latvia (Barševskis 2003), Belarus (Vigna Taglianti 2009).

*caraboides* **(Schrank, 1781)**. Pileckis 1960, 1976a; Silfverberg 1992, 2004; Pileckis and Monsevičius 1995; Barševskis 2003; Ferenca 2006b; Alekseev 2008c.

[*curtum **(Heyden, 1870)**].# 7. Tamutis and Ferenca 2006.

*flavipes* **(Linnaeus, 1761)**. Ogijewicz 1933; Pileckis 1960, 1976a; Sharova and Grüntal 1973; Silfverberg 1992, 2004; Gaidienė 1993; Pileckis and Monsevičius 1995; Monsevičius 1997; Tamutis 2000, 2005a; Marggi et al. 2003; Tamutis et al. 2004, 2006, 2007; Ferenca 2006b; Alekseev 2008c.

*pallipes* **(Duftschmid, 1812)**. Ogijewicz 1933; Mazurowa and Mazur 1939; Pileckis 1960, 1976a; Silfverberg 1992, 2004; Gaidienė 1993; Pileckis and Monsevičius 1995; Marggi et al. 2003; Tamutis 2005a; Tamutis et al. 2007; Alekseev 2008c.

*Odontium* **LeConte, 1848**.

*argenteolum* **(Ahrens, 1812)**. Jakobson 1905-1915; Ogijewicz 1933; Pileckis 1960, 1976a; Sharova and Grüntal 1973; Bercio and Folwaczny 1979; Silfverberg 1992, 2004; Pileckis and Monsevičius 1995 (*Bembidion*); Marggi et al. 2003; Ferenca 2006b; Alekseev 2008c; Vigna Taglianti 2009.

*foraminosum **(Sturm, 1825)**. Marggi et al. 2003; Vigna Taglianti 2009.# 8.

*litorale* **(Olivier, 1790)**. Heyden 1903; Jakobson 1905-1915; Ogijewicz 1933; Mazurowa and Mazur 1939; Pileckis 1960, 1976a; Sharova and Grüntal 1973; Silfverberg 1992, 2004; Gaidienė 1993; Pileckis and Monsevičius 1995 (*Bembidion*); Monsevičius 1997; Marggi et al. 2003; Ferenca 2006b; Vaivilavičius 2008; Alekseev 2008c; Vigna Taglianti 2009; Šablevičius 2011.

*striatum* **(Fabricius, 1792)**. Ogijewicz 1933; Pileckis 1960, 1976a; Silfverberg 1992, 2004; Gaidienė 1993; Pileckis and Monsevičius 1995 (*Bembidion*); Marggi et al. 2003; Ferenca 2006b; Alekseev 2008c; Vigna Taglianti 2009.

*velox* **(Linnaeus, 1761)**. Jakobson 1905-1915; Ogijewicz 1933; Pileckis 1960, 1963b, 1976a; Bercio and Folwaczny 1979; Silfverberg 1992, 2004; Pileckis and Monsevičius 1995 (*Bembidion*); Marggi et al. 2003; Ferenca 2004, 2006b; Alekseev 2008a, c; Vigna Taglianti 2009.

*Metallina* **Motschulsky, 1850**.

*lampros* **(Herbst, 1784)**. Heyden 1903; Ogijewicz 1933; Mazurowa and Mazur 1939; Pileckis 1960, 1976a; Zajančkauskas and Pileckis 1968; Sharova and Grüntal 1973; Silfverberg 1992, 2004; Gaidienė 1993; Pileckis and Monsevičius 1995 (*Bembidion*); Monsevičius 1997; Marggi et al. 2003; Tamutis 2005a; Ferenca 2006b; Alekseev 2008c; Vigna Taglianti 2009.

*nigricorne* **(Gyllenhal, 1827)**. Jakobson 1905-1915; Pileckis 1976a; Silfverberg 1992, 2004; Pileckis and Monsevičius 1995 (*Bembidion*); Alekseev 2008c.

*pygmaea* **(Fabricius, 1792)**. Jakobson 1905-1915; Ogijewicz 1933; Mazurowa and Mazur 1939; Pileckis 1960, 1976a; Dvilevičius et al. 1988; Silfverberg 1992, 2004; Gaidienė 1993; Pileckis and Monsevičius 1995 (*Bembidion*); Marggi et al. 2003; Ferenca 2006b; Alekseev 2008c; Vigna Taglianti 2009.

*properans* **(Stephens, 1828)**. Ogijewicz 1933; Pileckis 1960, 1976a; Zajančkauskas and Pileckis 1968; Sharova and Grüntal 1973; Silfverberg 1992, 2004; Pileckis and Monsevičius 1995 (*Bembidion*); Monsevičius 1997; Tamutis 1999, 2000, 2005a; Marggi et al. 2003; Tamutis et al. 2004, 2006, 2007; Ferenca 2006b; Vigna Taglianti 2009; Alekseev 2008c.

*Phyla* **Motschulsky, 1844**.

*bipunctatum* **(Linnaeus, 1761)**. Jakobson 1905-1915; Ogijewicz 1933; Pileckis 1960, 1976a; Silfverberg 1992, 2004; Pileckis and Monsevičius 1995 (*Bembidion*); Marggi et al. 2003; Alekseev 2008c; Vigna Taglianti 2009.

*obtusa* **(Audinet-Serville, 1821)**. Gaidienė 1993; Tamutis 2003; Marggi et al. 2003; Silfverberg 2004; Alekseev 2008c; Ivinskis et al. 2009; Vigna Taglianti 2009.

*pallidipenne* **(Illiger, 1802)**. Łomnicki 1913; Pileckis 1968b, 1976a, b, 1979; Silfverberg 1992, 2004; Pileckis and Monsevičius 1995 (*Bembidion*); Marggi et al. 2003; Ferenca 2004; Ivinskis and Rimšaitė 2005; Alekseev 2008c; Vigna Taglianti 2009.

*Princidium* **Motschulsky, 1864**.

*punctulatum* **(Drapiez, 1821)**. Roubal 1910; Ogijewicz 1933; Mazurowa and Mazur 1939; Pileckis 1960, 1970a, 1976a; Bercio and Folwaczny 1979; Silfverberg 1992, 2004; Gaidienė 1993; Pileckis and Monsevičius 1995 (*Bembidion*); Marggi et al. 2003; Ferenca 2006b; Alekseev 2008c; Vigna Taglianti 2009.

*ruficolle* **(Panzer, 1797)**. Heyden 1903; Pileckis 1960, 1976a; Silfverberg 1992, 2004; Pileckis and Monsevičius 1995 (*Bembidion*); Marggi et al. 2003; Alekseev 2008c; Vigna Taglianti 2009.

*Ocydromus* **Clairville, 1806**.

*bruxellensis* **(Westmaël, 1835) =** *rupestre* auct. nec (Linnaeus, 1758). Jakobson 1905-1915; Ogijewicz 1933; Pileckis 1960, 1976a; Silfverberg 1992, 2004; Gaidienė 1993; Pileckis and Monsevičius 1995 (*Bembidion*); Monsevičius 1997; Marggi et al. 2003; Alekseev 2008c; Vigna Taglianti 2009.

*coeruleus **(Audient-Serville, 1821)**. Ferenca et al. 2002; disproved by Ferenca (2003).

*cruciatus polonicum* **(P.W.J. Müller, 1930)**. Ogijewicz 1933; Mazurowa and Mazur 1939; Pileckis 1960, 1976a; Bercio and Folwaczny 1979; Silfverberg 1992, 2004; Gaidienė 1993; Pileckis and Monsevičius 1995 (*Bembidion*); Monsevičius 1997; Šablevičius 2000b; Marggi et al. 2003; Ferenca 2006b; Alekseev 2008c; Vigna Taglianti 2009.

[*dauricus* **(Motschulsky, 1844)**]. Known in Latvia and Estonia (Vigna Taglianti 2009).

*decorus **(Panzer, 1799)**. # 15. Marggi et al. 2003; Vigna Taglianti 2009.

*deletus* **(Audinet-Serville, 1821)** *= nitidulum* (Marsham, 1802) nec (Schrank, 1781). Pileckis 1976a; Silfverberg 1992, 2004; Pileckis and Monsevičius 1995; Monsevičius 1997; Marggi et al. 2003; Alekseev 2008c; Vigna Taglianti 2009.

*femoratus* **(Sturm, 1825)**. Roubal 1910; Ogijewicz 1933; Mazurowa and Mazur 1939; Pileckis 1960, 1976a; Silfverberg 1992, 2004; Pileckis and Monsevičius 1995 (*Bembidion*); Monsevičius 1997; Marggi et al. 2003; Vaivilavičius 2008; Alekseev 2008c; Vigna Taglianti 2009.

*fluviatilis* **(Dejean, 1831)**. # 15. Marggi et al. 2003; Vigna Taglianti 2009.

*lunatus* **(Duftschmid, 1812)**. Pileckis 1976a; Bercio and Folwaczny 1979; Silfverberg 1992, 2004; Gaidienė 1993; Pileckis and Monsevičius 1995 (*Bembidion*); Marggi et al. 2003; Alekseev 2008c; Vigna Taglianti 2009.

[*maritimus* **(Stephens, 1835)**]. Known in Denmark and Finland (Vigna Taglianti 2009).

*modestus **(Fabricius, 1801)**. # 15. Marggi et al. 2003; Vigna Taglianti 2009.

*monticola* **(Sturm, 1825)**. Miländer et al. 1984; Silfverberg 1992, 2004; Pileckis and Monsevičius 1995 (*Bembidion*); Marggi et al. 2003; Alekseev 2008c; Vigna Taglianti 2009.

[*obscurellus* **(Motschulsky, 1844)**]. Known in Latvia (Telnov et al. 2006), Estonia, Denmark (Vigna Taglianti 2009), southern Sweden (Lundberg and Gustafsson 1995).

*saxatilis* **(Gyllenhal, 1827)**.Heyden 1903; Mazurowa and Mazur 1939; Pileckis 1960, 1976a; Silfverberg 1992, 2004; Gaidienė 1993; Pileckis and Monsevičius 1995 (*Bembidion*); Marggi et al. 2003; Alekseev 2008c; Vigna Taglianti 2009.

*stephensii* **(Crotch, 1869)**. Ferenca 2003; Marggi et al. 2003 Vigna Taglianti 2009; Alekseev 2008c.

*tetracolus* **(Say, 1823) =** *ustulatus* (Linnaeus, 1758). Heyden 1903; Ogijewicz 1933; Mazurowa and Mazur 1939; Pileckis 1960, 1976a; Sharova and Grüntal 1973; Dvilevičius et al. 1988; Silfverberg 1992, 2004; Gaidienė 1993; Pileckis and Monsevičius 1995 (*Bembidion*); Monsevičius 1997; Tamutis 2000, 2005a; Tamutis and Zolubas 2001; Marggi et al. 2003; Tamutis et al. 2004, 2007; Ferenca 2006b; Alekseev 2008c.

*tetragrammus illigeri* **(Netolitzky, 1914) =** *quadriguttatus* (Illiger, 1798) = *genei* auct. nec (Küster, 1847). Ogijewicz 1933; Pileckis 1960, 1976a; Silfverberg 1992, 2004; Pileckis and Monsevičius 1995 (*Bembidion*); Monsevičius 1997; Marggi et al. 2003; Alekseev 2008c; Vigna Taglianti 2009.

*tibialis **(Duftschmid, 1812)**. # 15. Marggi et al. 2003; Vigna Taglianti 2009.

*Sinechostictus* **Motschulsky, 1864**.

*elongatus **(Dejean, 1831)**. # 9. Pileckis 1960 (*Bembidion*); Ferenca 2006b.

*Plataphus* **Motschulsky, 1864**.

[*crenulatus* **(R.F. Sahlberg, 1844)**]. Known in Estonia, Latvia (Vigna Taglianti 2009).

[*difficilis* **(Motschulsky, 1844)**]. Known in Estonia, Latvia (Vigna Taglianti 2009), central Sweden (Lundberg and Gustafsson 1995).

[*hastii* **(C.R. Sahlberg, 1827)**]. Known in Estonia, Latvia (Vigna Taglianti 2009), northern Sweden (Lundberg and Gustafsson 1995).

[*prasinus* **(Duftschmid, 1812)**]. Known in Estonia, Latvia (Vigna Taglianti 2009), central Sweden (Lundberg and Gustafsson 1995).

[*virens* **(Gyllenhal, 1827)**]. Known in Estonia, Latvia (Vigna Taglianti 2009), central Sweden (Lundberg and Gustafsson 1995).

*Notaphus* **Dejean, 1821**.

*dentellum* **(Thunberg, 1787)**. Ogijewicz 1933; Pileckis 1960, 1976a; Sharova and Grüntal 1973; Dvilevičius et al. 1988; Silfverberg 1992, 2004; Gaidienė 1993; Pileckis and Monsevičius 1995 (*Bembidion*); Monsevičius 1997; Marggi et al. 2003; Ferenca 2006b; Alekseev 2008c; Vigna Taglianti 2009.

*varium* **(Olivier, 1795)**.Heyden 1903; Ogijewicz 1933; Pileckis 1960, 1976a; Silfverberg 1992, 2004; Gaidienė 1993; Pileckis and Monsevičius 1995 (*Bembidion*); Šablevičius 2000b; Marggi et al. 2003; Ferenca 2006b; Alekseev 2008c; Vigna Taglianti 2009.

*semipunctatum* **(Donovan, 1806)**. Roubal 1910; Pileckis 1960, 1976a; Sharova and Grüntal 1973; Silfverberg 1992, 2004; Gaidienė 1993; Pileckis and Monsevičius 1995 (*Bembidion*); Monsevičius 1997; Marggi et al. 2003; Ferenca 2006b; Vaivilavičius 2008; Alekseev 2008c; Vigna Taglianti 2009.

*obliquum* **(Sturm, 1825)**. Ogijewicz 1933; Mazurowa and Mazur 1939; Pileckis 1960, 1976a; Sharova and Grüntal 1973; Dvilevičius et al. 1988; Silfverberg 1992, 2004; Gaidienė 1993; Pileckis and Monsevičius 1995 (*Bembidion*); Monsevičius 1997; Šablevičius 2000b; Marggi et al. 2003; Ferenca 2006b; Alekseev 2008c; Vigna Taglianti 2009.

[*ephippium* **(Marsham, 1802)**].Known in Denmark and Germany (Vigna Taglianti 2009).

*Emphanes* **Motschulsky, 1850**.

*azurescens* **(Dalla Torre**, **1877)**. Sharova and Grüntal 1973; Pileckis 1976a; Silfverberg 1992, 2004; Gaidienė 1993; Pileckis and Monsevičius 1995 (*Bembidion*); Marggi et al. 2003; Alekseev 2008c; Vigna Taglianti 2009.

*minimus* **(Fabricius, 1792)**. Pileckis 1960, 1976a; Silfverberg 1992, 2004; Pileckis and Monsevičius 1995 (*Bembidion*); Marggi et al. 2003; Ferenca 2006b; Alekseev 2008c; Vigna Taglianti 2009.

[*normannus* **(Dejean, 1831)**].Known in Denmark, Germany (Vigna Taglianti 2009), Poland (Burakowski et al. 1973).

*tenellus* **(Erichson, 1837)**. Roubal 1910; Ogijewicz 1933; Mazurowa and Mazur 1939; Pileckis 1960, 1976a; Silfverberg 1992, 2004; Gaidienė 1993; Pileckis and Monsevičius 1995 (*Bembidion*); Ferenca 2006b; Alekseev 2008c.

*Trepanes* **Motschulsky, 1864**.

*articulatus* **(Panzer, 1796)**. Heyden 1903; Ogijewicz 1933; Mazurowa and Mazur 1939; Pileckis 1960, 1976a; Sharova and Grüntal 1973; Dvilevičius et al. 1988; Silfverberg 1992, 2004; Gaidienė 1993; Pileckis and Monsevičius 1995 (*Bembidion*); Monsevičius 1997; Marggi et al. 2003; Ferenca 2006b 2009; Vaivilavičius 2008; Alekseev 2008c; Vigna Taglianti 2009.

*assimilis* **(Gyllenhal, 1810)**. Ogijewicz 1933; Sharova and Grüntal 1973; Pileckis 1976a; Dvilevičius et al. 1988; Silfverberg 1992, 2004; Gaidienė 1993; Pileckis and Monsevičius 1995 (*Bembidion*); Monsevičius 1997; Marggi et al. 2003; Ferenca 2006b; Alekseev 2008 a,c; Vigna Taglianti 2009.

[*clarkii* **(Dawson, 1849)**]. Known in southern Sweden (Lundberg and Gustafsson 1995), Denmark, Germany (Vigna Taglianti 2009), Poland (Burakowski et al. 1973).

*doris* **(Panzer, 1797)**. Jakobson 1905-1915; Mazurowa and Mazur 1939; Pileckis 1960, 1976a; Sharova and Grüntal 1973; Silfverberg 1992, 2004; Gaidienė 1993; Pileckis and Monsevičius 1995 (*Bembidion*); Monsevičius 1997; Marggi et al. 2003; Ferenca 2006b; Alekseev 2008c; Vigna Taglianti 2009.

*fumigatus* **(Duftschmid, 1812)**. Pileckis 1976a; Silfverberg 1992, 2004; Pileckis and Monsevičius 1995 (*Bembidion*); Alekseev 2008c.

*gilvipes* **(Sturm, 1825)**. Sharova and Grüntal 1973; Pileckis 1976a; Silfverberg 1992, 2004; Pileckis and Monsevičius 1995 (*Bembidion*); Monsevičius 1997; Tamutis 2000, 2005a; Tamutis and Zolubas 2001; Marggi et al. 2003; Tamutis et al. 2004; Alekseev 2008c; Vigna Taglianti 2009.

*octomaculatus* **(Goeze, 1777)**. Jakobson 1905-1915; Pileckis 1960, 1976a; Sharova and Grüntal 1973; Silfverberg 1992, 2004; Gaidienė 1993; Pileckis and Monsevičius 1995 (*Bembidion*); Monsevičius 1997; Marggi et al. 2003; Ferenca 2006b; Alekseev 2008c; Vigna Taglianti 2009.

*shuppelii* **(Dejean, 1831)**. Sharova and Grüntal 1973; Pileckis 1976a; Silfverberg 1992, 2004; Gaidienė 1993; Pileckis and Monsevičius 1995 (*Bembidion*); Monsevičius 1997; Alekseev 2008c.

*transparens* **(Gebler, 1829)**. Monsevičius and Pankevičius 2001 (*Bembidion*).

*Bembidion* **Latreille, 1802**.

*humerale* **Sturm, 1825**.Jakobson 1905-1915; Pileckis 1976a; Silfverberg 1992, 2004; Pileckis and Monsevičius 1995; Marggi et al. 2003; Alekseev 2008c; Vigna Taglianti 2009.

*quadrimaculatum* **(Linnaeus, 1761)**. Heyden 1903; Ogijewicz 1933; Mazurowa and Mazur 1939; Pileckis 1958b, 1960, 1976a; Pileckis et al. 1968; Sharova and Grüntal 1973; Dvilevičius et al. 1988; Silfverberg 1992, 2004; Gaidienė 1993; Pileckis and Monsevičius 1995; Monsevičius 1997; Tamutis 2000, 2005a; Marggi et al. 2003; Tamutis et al. 2004, 2007; Ferenca 2006b; Alekseev 2008c; Vigna Taglianti 2009.

*quadripustulatum* **Audinet-Serville, 1821 =** *quadriguttatus* Olivier, 1795. # 10. Pileckis 1960, 1976a; Silfverberg 1992, 2004; Pileckis and Monsevičius 1995; Barševskis 2003; Marggi et al. 2003; Alekseev 2008c; Vigna Taglianti 2009.

*Philochthus* **Stephens, 1828**.

*aeneus* **(Germar, 1824)**. Łomnicki 1913; Pileckis 1976a; Silfverberg 1992, 2004; Pileckis and Monsevičius 1995 (*Bembidion*); Alekseev 2008c; Ivinskis et al. 2009.

*biguttatus* **(Fabricius, 1779)**. Jakobson 1905-1915; Pileckis 1960, 1976a; Sharova and Grüntal 1973; Silfverberg 1992, 2004; Gaidienė 1993; Pileckis and Monsevičius 1995 (*Bembidion*); Monsevičius 1997; Tamutis 2000, 2005a; Marggi et al. 2003; Ferenca 2006b; Alekseev 2008c; Vigna Taglianti 2009.

*guttula* **(Fabricius, 1792)**. Jakobson 1905-1915; Ogijewicz 1933; Mazurowa and Mazur 1939; Pileckis 1960, 1976a; Sharova and Grüntal 1973; Dvilevičius et al. 1988; Silfverberg 1992, 2004; Gaidienė 1993; Pileckis and Monsevičius 1995 (*Bembidion*); Monsevičius 1997; Tamutis 2000; Marggi et al. 2003; Tamutis et al. 2004, 2007; Alekseev 2008c; Vigna Taglianti 2009.

[*inoptatus* **(Schaum, 1857)**].Known in Latvia (Telnov et al. 2006).

*lunulatus* **(Geoffroy, 1785)**. Pileckis 1960, 1976a; Silfverberg 1992, 2004; Gaidienė 1993; Pileckis and Monsevičius 1995 (*Bembidion*); Marggi et al. 2003; Alekseev 2008c; Vigna Taglianti 2009.

*mannerheimii* **C.R. Sahlberg, 1827 =** *unicolor* Chaudoir, 1850. Pileckis 1960, 1976a; Bercio and Folwaczny 1979; Silfverberg 1992, 2004; Gaidienė 1993; Pileckis and Monsevičius 1995 (*Bembidion*); Monsevičius 1997; Marggi et al. 2003; Alekseev 2008c; Vigna Taglianti 2009.

*neresheimeri* **P.W.J. Müller,** **1929**. Silfverberg 1992, 2004; Pileckis and Monsevičius 1995 (*Bembidion*); Marggi et al. 2003; Tamutis 2005a; Alekseev 2008c; Vigna Taglianti 2009.

*Ocys* **Stephens, 1828**.

*quinquestriatus* **(Gyllenhal, 1810)**. Bercio and Folwaczny 1979; Tamutis 2003; Alekseev 2008c.

*Paratachys* **Casey, 1918**.

*bistriatus* **(Duftschmid, 1812)**.# 11. Pileckis and Monsevičius 1995 (*Tachys*); Kopecký 2003; Silfverberg 2004; Alekseev 2008c; Vigna Taglianti 2009.

*micros* **(Fischer von Waldheim, 1828)**. Tamutis 2003; Barševskis 2003; Kopecký 2003; Vigna Taglianti 2009.

*Porotachys* **Netolitzky, 1914**.

*bisulcatus* **(Nicolai, 1822)**. Kopecký 2003; Vigna Taglianti 2009.

*Tachyura* **Motschulsky, 1862**.

*parvula* **(Dejean, 1831)**. Ferenca 2003 (*Elaphropus parvulus*).

*Tachyta* **Kirby, 1837**.

*nana* **(Gyllenhal, 1810)**. Ogijewicz 1933; Pileckis 1960, 1976a; Jakaitis and Valenta 1976; Silfverberg 1992, 2004; Gaidienė 1993; Pileckis and Monsevičius 1995; Monsevičius 1997; Šablevičius 2000b; Kopecký 2003; Ferenca 2003, 2004; Vaivilavičius 2008; Alekseev 2008a, c; Ivinskis et al. 1998, 2009; Vigna Taglianti 2009.

**Patrobinae Kirby, 1837**.

**Patrobini Kirby, 1837**.

*Patrobus* **Dejean, 1821**.

*assimilis* **Chaudoir, 1844**. Pileckis and Monsevičius 1982, 1995; Silfverberg 1992, 2004; Monsevičius 1997; Alekseev 2008c; Ivinskis et al. 2009.

*atrorufus* **Ström, 1768** **=** *excavatus* Paykull, 1790. Roubal 1910; Ogijewicz 1933; Pileckis 1960, 1976a; Silfverberg 1992, 2004; Gaidienė 1993; Pileckis and Monsevičius 1995; Monsevičius 1997; Zamotailov 2003; Lynikienė 2006; Žiogas and Vaičikauskas 2007b; Alekseev 2008c; Vigna Taglianti 2009.

*septentrionis* **Dejean, 1828 =** *australis* J.R. Sahlberg, 1875. Pileckis and Monsevičius 1982, 1995; Silfverberg 1992, 2004; Monsevičius 1997; Ferenca 2003; Alekseev 2008c.

**Brachininae Bonelli, 1810**.

**Brachinini Bonelli, 1810**.

*Brachinus* **Weber, 1801**.

*crepitans* **(Linnaeus, 1758)**. Hrdlička 2003; Vigna Taglianti 2009.

*explodens* **(Duftshmid, 1812)**. Hrdlička 2003; Vigna Taglianti 2009.

**Harpalinae Bonelli, 1810**.

**Pterostichini Bonelli, 1810**.

*Stomis* **Clairville, 1806**.

*pumicatus* **(Panzer, 1796)**. Roubal 1910; Ogijewicz 1933; Pileckis 1960, 1970a, 1976a; Silfverberg 1992, 2004; Gaidienė 1993; Pileckis and Monsevičius 1995; Monsevičius 1997; Šablevičius 2000b; Bousquet 2003c; Ferenca 2006b; Žiogas and Vaičikauskas 2007b; Alekseev 2008c; Ivinskis et al. 2008, 2009; Vigna Taglianti 2009.

*Poecilus* **Bonelli, 1810**.

*cupreus* **(Linnaeus, 1758)**. Heyden 1903; Ogijewicz 1933; Mazurowa and Mazur 1939; Pileckis 1960, 1976a; Zajančkauskas and Pileckis 1968; Sharova and Grüntal 1973; Strazdienė 1976; Dvilevičius et al. 1988; Pileckis and Šaluchaitė 1993; Silfverberg 1992, 2004; Gaidienė 1993 (*Pterostichus*); Pileckis and Monsevičius 1995 (*Pterostichus*); Monsevičius 1997, 2001; Tamutis 1999, 2000; Šablevičius 2000b; Gliaudys 2001; Bousquet 2003c; Tamutis et al. 2004, 2006, 2007; Ferenca 2006b; Žiogas and Vaičikauskas 2007b; Vaivilavičius 2008; Alekseev 2008c; Vigna Taglianti 2009.

*lepidus* **(Leske, 1785)** *= virens* (O.F. Müller, 1776). Heyden 1903; Ogijewicz 1933; Mazurowa and Mazur 1939; Pileckis 1960, 1976a; Strazdienė 1976, 1981, 1988; Silfverberg 1992, 2004; Gaidienė 1993 (*Pterostichus*); Pileckis and Monsevičius 1995 (*Pterostichus*); Monsevičius 1997; Šablevičius 2000b; Bousquet 2003c; Ferenca 2006b; Lynikienė 2006; Alekseev 2008c; Vigna Taglianti 2009.

*punctulatus* **(Schaller, 1783)**. Ogijewicz 1933; Pileckis 1960, 1976a; Strazdienė 1976; Bercio and Folwaczny 1979; Silfverberg 1992, 2004; Gaidienė 1993 (*Pterostichus*); Pileckis and Monsevičius 1995 (*Pterostichus*); Tamutis 1999, 2000; Bousquet 2003c; Ferenca 2006b; Alekseev 2008c; Vigna Taglianti 2009.

*versicolor* **(Sturm, 1824)** *= caerulescens* auct. nec (Linnaeus, 1758). Heyden 1903; Ogijewicz 1933; Mazurowa and Mazur 1939; Pileckis 1960, 1976a; Zajančkauskas and Pileckis 1968; Sharova and Grüntal 1973; Dvilevičius et al. 1988; Silfverberg 1992, 2004; Gaidienė 1993 (*Pterostichus*); Pileckis and Monsevičius 1995 (*Pterostichus*); Monsevičius 1997; Šablevičius 2000b; Tamutis 2000, 2005a; Gliaudys 2001; Bousquet 2003c; Tamutis et al. 2004, 2007; Žiogas and Zolubas 2005; Ferenca 2006b; Lynikienė 2006; Vaivilavičius 2008; Alekseev 2008c; Vigna Taglianti 2009.

*Pterostichus* **Bonelli, 1810**.

*aethiops* **(Panzer, 1796)**. Ogijewicz 1933; Mazurowa and Mazur 1939; Pileckis 1960, 1976a; Silfverberg 1992, 2004; Gaidienė 1993; Pileckis and Monsevičius 1995; Monsevičius 1997; Šablevičius 2000b; Bousquet 2003c; Žiogas and Zolubas 2005; Žiogas et al. 2006; Ferenca 2006b; Lynikienė 2006 (*P. atterrimus* Hbst.); Vaivilavičius 2008; Ivinskis et al. 2008; Alekseev 2008c; Vigna Taglianti 2009.

*anthracinus* **(Illiger, 1798)**. Ogijewicz 1933; Pileckis 1960, 1976a; Zajančkauskas and Pileckis 1968; Sharova and Grüntal 1973; Dvilevičius et al. 1988; Silfverberg 1992, 2004; Gaidienė 1993; Pileckis and Monsevičius 1995; Monsevičius 1997; Bousquet 2003c; Žiogas et al. 2006; Ferenca 2006b; Žiogas and Vaičikauskas 2007b; Alekseev 2008c; Vigna Taglianti 2009.

*atterimus* **(Herbst, 1784)**. Heyden 1903; Pileckis 1960, 1976a; Sharova and Grüntal 1973; Bercio and Folwaczny 1979; Silfverberg 1992, 2004; Gaidienė 1993; Pileckis and Monsevičius 1995; Monsevičius 1997; Bousquet 2003c; Ferenca 2006b; Ivinskis et al. 2008; Alekseev 2008c; Vigna Taglianti 2009.

*crenatus* **(Duftschmid, 1812)** *= vernalis* (Panzer, 1796) nec (O.F. Müller, 1776). Pileckis 1960, 1976a; Zajančkauskas and Pileckis 1968; Sharova and Grüntal 1973; Dvilevičius et al. 1988; Silfverberg 1992, 2004; Gaidienė 1993; Pileckis and Monsevičius 1995; Monsevičius 1997; Bousquet 2003c; Žiogas and Zolubas 2005; Ferenca 2006b; Lynikienė 2006; Alekseev 2008a, c; Ivinskis et al. 2008; Vigna Taglianti 2009.

*diligens* **(Sturm, 1824)**. Ogijewicz 1933; Pileckis 1960, 1976a; Sharova and Grüntal 1973; Ivinskis et al. 1984; Dvilevičius et al. 1988; Silfverberg 1992, 2004; Gaidienė 1993; Pileckis and Monsevičius 1995; Monsevičius 1997, 2001; Bousquet 2003c; Tamutis 2000, 2005a; Tamutis et al. 2004; Žiogas et al. 2006; Ferenca 2006b; Žiogas and Vaičikauskas 2007b; Dapkus and Tamutis 2007, 2008a; Ivinskis et al. 2008; Alekseev 2008c; Vigna Taglianti 2009.

*gracilis* **(Dejean, 1828) =** *guentheri* (Sturm, 1824). Ogijewicz 1933; Pileckis 1960, 1963b, 1976a; Zajančkauskas and Pileckis 1968; Silfverberg 1992, 2004; Gaidienė 1993; Pileckis and Monsevičius 1995; Monsevičius 1997; Bousquet 2003c; Ferenca 2006b; Alekseev 2008c; Vigna Taglianti 2009.

[*longicollis* **(Duftschmid, 1812)** *= inaequalis* (Marsham, 1802) nec (Panzer, 1796)]. Known in northern and central Poland (Burakowski et al. 1974), Denmark (Vigna Taglianti 2009).

*macer* **(Marsham, 1802)**. Sharova and Grüntal 1973; Pileckis 1976a; Silfverberg 1992, 2004; Gaidienė 1993; Pileckis and Monsevičius 1995; Monsevičius 1997; Tamutis 1999, 2000, 2005a; Bousquet 2003c; Tamutis et al. 2006, 2007; Vaivilavičius 2008; Alekseev 2008c; Vigna Taglianti 2009.

[*madidus* **(Fabricius, 1775)**]. Pervasive species to the European east. Known in Estonia, Poland, Denmark (Vigna Taglianti 2009).

*melanarius* **(Illiger, 1798)** *= vulgaris* auct. nec (Linnaeus, 1758). Heyden 1903; Ogijewicz 1933; Pileckis 1960, 1976a; Lešinskas and Pileckis 1967; Zajančkauskas and Pileckis 1968; Sharova and Grüntal 1973; Strazdienė 1976, 1988; Dvilevičius et al. 1988; Pileckis and Šaluchaitė 1993; Silfverberg 1992, 2004; Gaidienė 1993; Pileckis and Monsevičius 1995; Monsevičius 1997; Tamutis 1999, 2000, 2005a; Gliaudys 2001; Bousquet 2003c; Tamutis et al. 2004, 2006, 2007; Žiogas et al. 2006; Ferenca 2006b; Žiogas and Vaičikauskas 2007a, b; Vaivilavičius 2008; Ivinskis et al. 2008; Alekseev 2008c; Vigna Taglianti 2009.

*minor* **(Gyllenhal, 1827) =** *brunneus* (Sturm, 1824). Ogijewicz 1933; Pileckis 1960, 1976a; Sharova and Grüntal 1973; Dvilevičius et al. 1988; Silfverberg 1992, 2004; Gaidienė 1993; Pileckis and Monsevičius 1995; Monsevičius 1997; Bousquet 2003c; Žiogas and Zolubas 2005; Ferenca 2006b; Vaivilavičius 2008; Ivinskis et al. 2008; Alekseev 2008c; Vigna Taglianti 2009.

*niger* **(Schaller, 1783)**. Heyden 1903; Raubal 1910; Ogijewicz 1933; Mazurowa and Mazur 1939; Pileckis 1960, 1976a; Lešinskas and Pileckis 1967; Sharova and Grüntal 1973; Silfverberg 1992, 2004; Gaidienė 1993; Strazdienė 1976, 1988; Dvilevičius et al. 1988; Pileckis and Monsevičius 1995; Monsevičius 1997; Tamutis 2000, 2005a; Šablevičius 2000b; Gliaudys 2001; Tamutis and Zolubas 2001; Bousquet 2003c; Tamutis et al. 2004, 2007; Žiogas et al. 2006; Ferenca 2006b; Lynikienė 2003, 2006; Žiogas and Vaičikauskas 2007b; Dapkus and Tamutis 2008a; Vaivilavičius 2008; Ivinskis et al. 2008; Alekseev 2008c; Vigna Taglianti 2009.

*nigrita* **(Paykull, 1790)**. Heyden 1903; Ogijewicz 1933; Mazurowa and Mazur 1939; Pileckis 1960, 1976a; Zajančkauskas and Pileckis 1968; Sharova and Grüntal 1973; Strazdienė 1976, 1981, 1988; Ivinskis et al. 1984, 2008; Dvilevičius et al. 1988; Silfverberg 1992, 2004; Gaidienė 1993; Pileckis and Monsevičius 1995; Monsevičius 1997, 2001; Tamutis 2000, 2005a; Bousquet 2003c; Tamutis et al. 2004; Žiogas et al. 2006; Ferenca 2006b; Žiogas and Vaičikauskas 2007a, b; Alekseev 2008c; Vigna Taglianti 2009.

*oblongopunctatus* **(Fabricius, 1787)**. Heyden 1903; Raubal 1910; Ogijewicz 1933; Mazurowa and Mazur 1939; Pileckis 1960, 1976a; Sharova and Grüntal 1973; Strazdienė 1988; Dvilevičius et al. 1988; Silfverberg 1992, 2004; Gaidienė 1993; Pileckis and Monsevičius 1995; Monsevičius 1997; Tamutis 2000, 2005a; Šablevičius 2000b; Tamutis and Zolubas 2001; Bousquet 2003c; Tamutis et al. 2004; Žiogas et al. 2006; Ferenca 2006b; Lynikienė 2003, 2006; Žiogas and Vaičikauskas 2007a, b; Dapkus and Tamutis 2007, 2008a; Vaivilavičius 2008; Ivinskis et al. 2008; Alekseev 2008c; Vigna Taglianti 2009.

*ovoideus* **(Sturm, 1824)**. Ferenca 2004, 2006a.

*quadrifoveolatus* **Letzner, 1852 =** *angustatus* (Duftschmid, 1812) nec (Fabricius, 1787). Barševskis 2001a; Silfverberg 2004; Alekseev 2008c; Vigna Taglianti 2009; Ivinskis et al. 2009.

*rhaeticus* **Heer, 1837**. Monsevičius and Pankevičius 2001; Barševskis 2001a; Bousquet 2003c; Silfverberg 2004; Vaivilavičius 2008; Alekseev 2008c; Vigna Taglianti 2009.

*strenuus* **(Panzer, 1796)**. Ogijewicz 1933; Pileckis 1960, 1976a; Sharova and Grüntal 1973; Dvilevičius et al. 1988; Silfverberg 1992, 2004; Gaidienė 1993; Pileckis and Monsevičius 1995; Monsevičius 1997; Tamutis 2000, 2005a; Šablevičius 2000b; Bousquet 2003c; Ferenca 2006b; Žiogas and Vaičikauskas 2007b; Dapkus and Tamutis 2007; Vaivilavičius 2008; Alekseev 2008c; Vigna Taglianti 2009.

*Pedius* **Motschulsky, 1850**.

[*longicollis* **(Duftschmid, 1812)** = *inaequalis* (Marsham, 1802) nec (Panzer, 1796)]. Known in northern and central Poland (Burakowski et al. 1974), Denmark (Vigna Taglianti 2009).

*Abax* **Bonelli, 1810**.

[*parallelepipedus* **(Piller & Mitterpacher, 1783)**]. Known in northern Poland (Burakowski et al. 1974), Denmark, Estonia, Kaliningrad region (Vigna Taglianti 2009).

**Zabrini Bonelli, 1810**.

*Amara* **Bonelli, 1810**.

*aenea* **(DeGeer, 1774)**. Ogijewicz 1933; Mazurowa and Mazur 1939; Pileckis 1960, 1976a; Lešinskas and Pileckis 1967; Zajančkauskas and Pileckis 1968; Sharova and Grüntal 1973; Dvilevičius et al. 1988; Silfverberg 1992, 2004; Gaidienė 1993; Pileckis and Monsevičius 1995; Monsevičius 1997; Šablevičius 2000b; Gliaudys 2001; Hieke 2003; Tamutis 2000, 2005a; Tamutis and Zolubas 2001; Tamutis et al. 2004; Ferenca 2006b; Žiogas and Vaičikauskas 2007b; Vaivilavičius 2008; Alekseev 2008c; Vigna Taglianti 2009.

[*anthobia* **A. Villa & G.B. Villa, 1833**]. Known in Latvia (Barševskis 2003), southern Sweden (Lundberg and Gustafsson 1995), Denmark (Vigna Taglianti 2009).

*apricaria* **(Paykull, 1790)**. Ogijewicz 1933; Mazurowa and Mazur 1939; Pileckis 1960, 1976a; Strazdienė 1976; Silfverberg 1992, 2004; Gaidienė 1993; Pileckis and Monsevičius 1995; Monsevičius 1997; Tamutis 2000, 2005a; Hieke 2003; Ferenca 2006b; Alekseev 2008c; Vigna Taglianti 2009.

*aulica* **(Panzer, 1796)**. Ogijewicz 1933; Pileckis 1960, 1976a; Strazdienė 1988; Silfverberg 1992, 2004; Gaidienė 1993; Pileckis and Monsevičius 1995; Monsevičius 1997; Tamutis 2000, 2005a; Tamutis and Zolubas 2001; Hieke 2003; Ferenca 2006b; Tamutis et al. 2004, 2007 Alekseev 2008c; Vigna Taglianti 2009.

*bifrons* **(Gyllenhal, 1810)**. Ogijewicz 1933; Mazurowa and Mazur 1939; Pileckis 1960, 1976a; Sharova and Grüntal 1973; Dvilevičius et al. 1988; Strazdienė 1981, 1988; Silfverberg 1992, 2004; Gaidienė 1993; Pileckis and Monsevičius 1995; Monsevičius 1997; Tamutis 2000, 2005a; Hieke 2003; Tamutis et al. 2004, 2007; Ferenca 2006b; Alekseev 2008c; Vigna Taglianti 2009.

*brunnea* **(Gyllenhal, 1810)**. Jakobson 1905-1915; Ogijewicz 1933; Pileckis 1960, 1976a; Sharova and Grüntal 1973; Dvilevičius et al. 1988; Strazdienė 1981, 1988; Silfverberg 1992, 2004; Gaidienė 1993; Pileckis and Monsevičius 1995; Monsevičius 1997; Hieke 2003; Žiogas and Zolubas 2005; Ferenca 2006b; Dapkus and Tamutis 2007; Žiogas and Vaičikauskas 2007b; Alekseev 2008c; Vigna Taglianti 2009.

*communis* **(Panzer, 1797)** *= pulpani* Kult, 1949 = *pseudocommunis* Burakowski, 1957. Ogijewicz 1933; Pileckis 1960, 1976a; Sharova and Grüntal 1973; Dvilevičius et al. 1988; Silfverberg 1992, 2004; Gaidienė 1993; Pileckis and Monsevičius 1995; Monsevičius 1997; Tamutis 2000, 2005a; Tamutis and Zolubas 2001; Hieke 2003; Tamutis et al. 2004; Žiogas and Zolubas 2005; Lynikienė 2006; Ferenca 2006b; Vaivilavičius 2008; Alekseev 2008c; Vigna Taglianti 2009.

*concinna* **Zimmermann, 1832**. # 12. Pileckis 1960, 1976a; Pileckis and Monsevičius 1995; Silfverberg 2004; Alekseev 2008c.

*consularis* **(Duftschmid, 1812)**. Ogijewicz 1933; Mazurowa and Mazur 1939; Pileckis 1960, 1976a; Strazdienė 1976; Silfverberg 1992, 2004; Gaidienė 1993; Pileckis and Monsevičius 1995; Monsevičius 1997; Tamutis 2000, 2005a; Hieke 2003; Tamutis et al. 2004; Ferenca 2006b; Alekseev 2008c; Vigna Taglianti 2009.

*convexior* **Stephens, 1828**. Jakobson 1905-1915; Ogijewicz 1933; Pileckis 1960, 1976a; Silfverberg 1992, 2004; Pileckis and Monsevičius 1995; Hieke 2003; Alekseev 2008c; Vigna Taglianti 2009.

*convexiuscula* **(Marsham, 1802)**. Hieke 2003; Vigna Taglianti 2009.

*crenata* **(Dejean, 1828)**. Pileckis 1960, 1976a; Silfverberg 1992, 2004; Pileckis and Monsevičius 1995; Ferenca 2006b; Alekseev 2008c.

*cursitans* **Zimmermann, 1832**. Pileckis and Monsevičius 1982, 1995; Silfverberg 1992, 2004; Monsevičius 1997; Alekseev 2008c.

*curta* **Dejean, 1828**. Pileckis 1960, 1976a; Silfverberg 1992, 2004; Gaidienė 1993; Pileckis and Monsevičius 1995; Monsevičius 1997; Hieke 2003; Ferenca 2006b; Alekseev 2008c; Vigna Taglianti 2009.

*equestris* **(Duftschmid, 1812)**. Ogijewicz 1933; Pileckis 1960, 1976a; Silfverberg 1992, 2004; Gaidienė 1993; Pileckis and Monsevičius 1995; Hieke 2003; Žiogas and Zolubas 2005; Ferenca 2006b; Alekseev 2008c; Vigna Taglianti 2009.

*erratica* **(Duftschmid, 1812)**. Pileckis and Monsevičius 1982, 1995; Silfverberg 1992, 2004; Alekseev 2008c.

*eurynota* **(Panzer, 1796)**. Ogijewicz 1933; Pileckis 1960, 1976a; Silfverberg 1992, 2004; Gaidienė 1993; Pileckis and Monsevičius 1995; Tamutis 2000, 2005a; Hieke 2003; Tamutis et al. 2004; Alekseev 2008c; Vigna Taglianti 2009.

*famelica* **Zimmermann, 1832**. Ogijewicz 1933; Pileckis 1960, 1976a; Silfverberg 1992, 2004; Gaidienė 1993; Pileckis and Monsevičius 1995; Tamutis 2000, 2005a; Hieke 2003; Alekseev 2008c; Vigna Taglianti 2009.

*familiaris* **(Duftschmid, 1812)**. Heyden 1903; Roubal 1910; Ogijewicz 1933; Mazurowa and Mazur 1939; Pileckis 1960, 1976a; Sharova and Grüntal 1973; Dvilevičius et al. 1988; Silfverberg 1992, 2004; Gaidienė 1993; Pileckis and Monsevičius 1995; Monsevičius 1997; Tamutis 2000, 2005a; Tamutis and Zolubas 2001; Hieke 2003; Tamutis et al. 2004, 2007; Ferenca 2006b; Žiogas and Vaičikauskas 2007b; Vaivilavičius 2008; Ivinskis et al. 2008; Alekseev 2008c; Vigna Taglianti 2009.

*fulva* **(O.F. Müller, 1776)**. Heyden 1903; Ogijewicz 1933; Mazurowa and Mazur 1939; Pileckis 1960, 1976a; Silfverberg 1992, 2004; Gaidienė 1993; Pileckis and Monsevičius 1995; Monsevičius 1997; Šablevičius 2000b; Tamutis and Zolubas 2001; Hieke 2003; Tamutis 2005a; Ferenca 2006b; Alekseev 2008c; Vigna Taglianti 2009.

[*fusca* **Dejean, 1828**].Known in southern Sweden (Lundberg and Gustafsson 1995), Denmark, Belarus (Vigna Taglianti 2009).

*gebleri* **Dejean, 1831 =** *helleri* Gredler, 1868. Barševskis 2001a; Silfverberg 2004; Ferenca et al. 2006, 2007; Alekseev 2008c; Vigna Taglianti 2009.

*infima* **(Duftschmid, 1812)**. Mazurowa and Mazur 1939; Pileckis 1960, 1976a; Silfverberg 1992, 2004; Gaidienė 1993; Pileckis and Monsevičius 1995; Hieke 2003; Alekseev 2008c; Vigna Taglianti 2009.

*ingenua* **(Duftschmid, 1812)**. Miländer et al. 1984; Silfverberg 1992, 2004; Gaidienė 1993; Pileckis and Monsevičius 1995; Hieke 2003; Žiogas and Zolubas 2005; Alekseev 2008c; Vigna Taglianti 2009.

*littorea* **Thomson, 1857 =** *kodymi* Jedlička, 1936. Monsevičius 1986a; Dvilevičius et al. 1988; Silfverberg 1992, 2004; Gaidienė 1993; Pileckis and Monsevičius 1995; Hieke 2003; Alekseev 2008c; Vigna Taglianti 2009.

*lucida* **(Duftschmid, 1812)**. Ogijewicz 1933; Pileckis 1960, 1976a; Bercio and Folwaczny 1979; Silfverberg 1992, 2004; Pileckis and Monsevičius 1995; Hieke 2003; Alekseev 2008c; Vigna Taglianti 2009.

*lunicollis* **Schiödte, 1837 =** *fatrica* Roubal, 1922. Jakobson 1905-1915; Ogijewicz 1933; Pileckis 1960, 1976a; Dvilevičius et al. 1988; Silfverberg 1992, 2004; Gaidienė 1993; Pileckis and Monsevičius 1995; Monsevičius 1997; Tamutis 2000, 2005a; Hieke 2003; Tamutis et al. 2004; Žiogas and Zolubas 2005; Alekseev 2008c; Vigna Taglianti 2009.

*majuscula* **(Chaudoir, 1850)**. Pileckis1970a, 1976a; Silfverberg 1992, 2004; Gaidienė 1993; Pileckis and Monsevičius 1995; Monsevičius 1997; Hieke 2003; Tamutis 2000, 2005a; Alekseev 2008c; Vigna Taglianti 2009.

[*montivaga* **Sturm, 1825**]. Known in northern Poland (Burakowski et al. 1974), southern Sweden (Lundberg and Gustafsson 1995), northwestern Belarus (Alexandrovitch et al. 1996), Kaliningrad region (Vigna Taglianti 2009).

*municipalis* **(Duftschmid, 1812)**. Ogijewicz 1933; Pileckis 1960, 1976a; Dvilevičius et al. 1988; Silfverberg 1992, 2004; Gaidienė 1993; Pileckis and Monsevičius 1995; Hieke 2003; Ferenca 2006b; Alekseev 2008c; Vigna Taglianti 2009.

*nitida* **Sturm, 1825**. Ogijewicz 1933; Pileckis 1960, 1976a; Silfverberg 1992, 2004; Gaidienė 1993; Pileckis and Monsevičius 1995; Hieke 2003; Ferenca 2006b; Alekseev 2008c; Vigna Taglianti 2009.

*ovata* **(Fabricius, 1792)**. Ogijewicz 1933; Pileckis 1960, 1976a; Silfverberg 1992, 2004; Pileckis and Monsevičius 1995; Tamutis 2000, 2005a; Hieke 2003; Tamutis et al. 2004; Ferenca 2006b; Alekseev 2008c; Vigna Taglianti 2009.

*plebeja* **(Gyllenhal, 1810)**. Ogijewicz 1933; Pileckis 1960, 1976a; Lešinskas and Pileckis 1967; Dvilevičius et al. 1988; Silfverberg 1992, 2004; Gaidienė 1993; Pileckis and Monsevičius 1995; Monsevičius 1997; Šablevičius 2000b; Tamutis 2000, 2005a; Hieke 2003; Tamutis et al. 2004, 2007; Ferenca 2006b; Vaivilavičius 2008; Alekseev 2008c; Vigna Taglianti 2009.

*praetemissa* **(C.R. Sahlberg, 1827)**. Pileckis 1960, 1976a; Silfverberg 1992, 2004; Gaidienė 1993; Pileckis and Monsevičius 1995; Hieke 2003; Ferenca 2006b; Alekseev 2008c; Vigna Taglianti 2009.

*quenseli silvicola* **(Zimmermann, 1832)**. Pileckis 1976a; Bercio and Folwaczny 1979; Silfverberg 1992, 2004; Pileckis and Monsevičius 1995; Hieke 2003; Alekseev 2008c.

*similata* **(Gyllenhal, 1810)**. Jakobson 1905-1915; Ogijewicz 1933; Pileckis 1960, 1976a; Dvilevičius et al. 1988; Silfverberg 1992, 2004; Gaidienė 1993; Pileckis and Monsevičius 1995; Monsevičius 1997; Tamutis 1999, 2000, 2005a; Hieke 2003; Tamutis et al. 2004, 2007; Ferenca 2006b; Alekseev 2008c; Vigna Taglianti 2009.

*spreta* **Dejean, 1831**. Jakobson 1905-1915; Ogijewicz 1933; Mazurowa and Mazur 1939; Pileckis 1960, 1976a; Silfverberg 1992, 2004; Gaidienė 1993; Pileckis and Monsevičius 1995; Monsevičius 1997; Hieke 2003; Ferenca 2006b; Vaivilavičius 2008; Alekseev 2008c; Vigna Taglianti 2009.

[*strenua* **Zimmermann, 1832**]. Known in northern Poland (Burakowski et al. 1974), Kaliningrad region (Alekseev 2008c), Latvia (Barševskis 2003), Denmark (Vigna Taglianti 2009).

*tibialis* **(Paykull, 1798)**. Mazurowa and Mazur 1939; Pileckis 1960, 1976a; Sharova and Grüntal 1973; Silfverberg 1992, 2004; Gaidienė 1993; Pileckis and Monsevičius 1995; Monsevičius 1997; Hieke 2003; Ferenca 2006b; Alekseev 2008c; Vigna Taglianti 2009.

[*tricuspidata* **Dejean, 1831**]. Known in northern Poland (Burakowski et al. 1974), southern Sweden (Lundberg and Gustafsson 1995), northwestern Belarus (Alexandrovitch et al. 1996), Denmark (Vigna Taglianti 2009).

*Zabrus* **Clairville, 1806**.

*tenebrioides* **(Goeze, 1777)**. Serrano and Andújar 2003; Vigna Taglianti 2009.

**Sphodrini Laporte, 1834**.

*Calathus* **Bonelli, 1811**.

*ambiguus* **(Paykull, 1790)**. Ogijewicz 1933; Mazurowa and Mazur 1939; Pileckis 1960, 1976a; Sharova and Grüntal 1973; Silfverberg 1992, 2004; Gaidienė 1993; Pileckis and Monsevičius 1995; Monsevičius 1997; Tamutis 2000; Hovorka and Sciaky 2003a; Ferenca 2006b; Alekseev 2008c; Vigna Taglianti 2009.

[*cinctus* **Motschulsky, 1850**]. Known in Latvia (Barševskis 2003), southern Sweden (Lundberg and Gustafsson 1995), Estonia, Poland, Denmark (Vigna Taglianti 2009).

*erratus* **(C.R. Sahlberg, 1827) =** *fulvipes* (Gyllenhal, 1810). Heyden 1903; Roubal, 1910; Ogijewicz 1933; Mazurowa and Mazur 1939; Pileckis 1960, 1976a; Strazdienė 1976, 1981; Ivinskis et al. 1984; 1988; Dvilevičius et al. 1988; Silfverberg 1992, 2004; Gaidienė 1993; Žiogas and Gedminas 1994; Pileckis and Monsevičius 1995; Monsevičius 1997; Hovorka and Sciaky 2003a; Ferenca 2006b; Lynikienė 2006; Alekseev 2008c; Vigna Taglianti 2009.

*fuscipes* **(Goeze, 1777)**. Heyden 1903; Ogijewicz 1933; Mazurowa and Mazur 1939; Pileckis 1960, 1970a, 1976a; Silfverberg 1992, 2004; Gaidienė 1993; Pileckis and Monsevičius 1995; Monsevičius 1997; Tamutis 1999, 2000, 2005a; Šablevičius 2000b; Hovorka and Sciaky 2003a; Tamutis et al. 2004, 2006, 2007; Ferenca 2006b; Vaivilavičius 2008; Alekseev 2008c; Vigna Taglianti 2009.

*melanocephalus* **(Linnaeus, 1758)**. Ogijewicz 1933; Mazurowa and Mazur 1939; Pileckis 1960, 1976a; Lešinskas and Pileckis 1967; Strazdienė 1981, 1988; Silfverberg 1992, 2004; Gaidienė 1993; Pileckis and Monsevičius 1995; Monsevičius 1997; Tamutis 2000, 2005a; Šablevičius 2000b; Hovorka and Sciaky 2003a; Tamutis et al. 2004, 2007; Žiogas and Zolubas 2005; Ferenca 2006b; Lynikienė 2006; Alekseev 2008c; Vigna Taglianti 2009.

*micropterus* **(Duftschmid, 1812)**. Jakobson 1905-1915; Ogijewicz 1933; Pileckis 1960, 1976a; Sharova and Grüntal 1973; Dvilevičius et al. 1988; Silfverberg 1992, 2004; Gaidienė 1993; Žiogas and Gedminas 1994; Pileckis and Monsevičius 1995; Monsevičius 1997; Šablevičius 2000b; Hovorka and Sciaky 2003a; Žiogas and Zolubas 2005; Žiogas et al. 2006; Ferenca 2006b; Žiogas and Vaičikauskas 2007a, b; Dapkus and Tamutis 2007, 2008a; Vaivilavičius 2008; Ivinskis et al. 2008; Alekseev 2008c; Vigna Taglianti 2009.

*mollis* **(Marsham, 1802) =** *ochropterus* auct. nec (Duftschmid, 1812). # 13. Ogijewicz 1933; Pileckis 1960, 1976a; Silfverberg 1992, 2004; Pileckis and Monsevičius 1995; Hovorka and Sciaky 2003a; Alekseev 2008c; Vigna Taglianti 2009.

*Sphodrus* **Clairville, 1806**

*leucophtalmus* **(Linnaeus, 1758)**. Jakobson 1905-1915; Ogijewicz 1933; Pileckis 1960, 1970a, 1976a; Silfverberg 1992, 2004; Gaidienė 1993; Pileckis and Monsevičius 1995; Monsevičius 1997; Ferenca 2006b; Alekseev 2008c.

*Laemostenus* **Bonelli, 1810** **=** *Pristonychus* Dejean, 1828.

*terricola* **(Herbst, 1784)**. Ogijewicz 1933; Pileckis 1960, 1976a; Silfverberg 1992, 2004; Gaidienė 1993; Pileckis and Monsevičius 1995; Ferenca 2006b; Alekseev 2008c.

*Dolichus* **Bonelli, 1811**

*halensis* **(Schaller, 1783)**. Ogijewicz 1933; Pileckis 1960, 1976a; Silfverberg 1992, 2004; Gaidienė 1993; Pileckis and Monsevičius 1995; Tamutis 2000, 2005a; Barševskis 2003; Hovorka and Sciaky 2003b; Tamutis et al. 2004, 2006, 2007; Alekseev 2008c.

*Synuchus* **Gyllenhal, 1810**.

*vivalis* **(Illiger, 1798)** = *nivalis* (Panzer, 1797) nec (Paykull, 1790). Ogijewicz 1933; Pileckis 1960, 1976a; Strazdienė 1976; Silfverberg 1992, 2004; Gaidienė 1993; Pileckis and Monsevičius 1995; Tamutis 1999, 2000, 2005a; Šablevičius 2000b; Hovorka and Sciaky 2003c; Tamutis et al. 2006, 2007; Vaivilavičius 2008; Alekseev 2008c; Vigna Taglianti 2009.

**Platynini Bonelli, 1810**.

*Olisthopus* **Dejean, 1828=** *Odontonyx* Stephens, 1827.

*rotundatus* **(Paykull, 1790)**. Lentz 1879; Jakobson 1905-1915; Ogijewicz 1933; Pileckis 1960, 1970a, 1976a; Bercio and Folwaczny 1979; Silfverberg 1992, 2004; Pileckis and Monsevičius 1995; Bousquet 2003d; Alekseev 2008c; Vigna Taglianti 2009.

*Sericoda* **Kirby, 1837**.

*quadripunctata* **(DeGeer, 1774)**. Pileckis 1960, 1976a; Bercio and Folwaczny 1979; Silfverberg 1992, 2004; Pileckis and Monsevičius 1995 (*Agonum*); Bousquet 2003d; Alekseev 2008c; Vigna Taglianti 2009.

*Anchomenus* **Bonelli, 1810 =** *Idiochroma* Bedel, 1902.

*dorsalis* **(Pontoppidan, 1763)**. Ogijewicz 1933; Pileckis 1960, 1976a; Lešinskas and Pileckis 1967; Zajančkauskas and Pileckis 1968; Sharova and Grüntal 1973; Silfverberg 1992, 2004; Gaidienė 1993; Pileckis and Monsevičius 1995 (*Agonum*); Monsevičius 1997; Tamutis 1999, 2000, 2005a; Bousquet 2003d; Tamutis et al. 2004, 2006a, 2007; Ferenca 2006b; Vaivilavičius 2008; Alekseev 2008c; Vigna Taglianti 2009.

*Oxypselaphus* **Chaudoir, 1843**.

*obscurus* **(Herbst, 1784)**. Ogijewicz 1933; Pileckis 1960, 1976a; Sharova and Grüntal 1973; Silfverberg 1992, 2004; Gaidienė 1993; Pileckis and Monsevičius 1995 (*Agonum*); Monsevičius 1997; Gliaudys 2001; Bousquet 2003d; Žiogas and Zolubas 2005; Ferenca 2006b; Vaivilavičius 2008; Ivinskis et al. 2008; Alekseev 2008c; Vigna Taglianti 2009.

*Paranchus* **Lindroth, 1974**.

*albipes* **(Fabricius, 1796) =** *ruficornis* (Goeze, 1777) nec (DeGeer, 1774). Ogijewicz 1933; Mazurowa and Mazur 1939; Pileckis 1960, 1976a; Silfverberg 1992, 2004; Gaidienė 1993; Pileckis and Monsevičius 1995 (*Agonum*); Bousquet 2003d; Alekseev 2008c; Vigna Taglianti 2009.

*Platynus* **Bonelli, 1810**.

*assimilis* **(Paykull, 1790)**. Heyden 1903; Ogijewicz 1933; Pileckis 1960, 1976a; Zajančkauskas and Pileckis 1968; Dvilevičius et al. 1988; Silfverberg 1992, 2004; Gaidienė 1993; Pileckis and Monsevičius 1995 (*Agonum*); Monsevičius 1997; Šablevičius 2000b; Bousquet 2003d; Žiogas and Zolubas 2005; Žiogas et al. 2006; Ferenca 2006b; Žiogas and Vaičikauskas 2007b; Vaivilavičius 2008; Ivinskis et al. 2008; Alekseev 2008c; Vigna Taglianti 2009.

*krynickii* **(Sperk, 1835)**. Pileckis and Monsevičius 1982, 1995 (*Agonum*); Silfverberg 1992, 2004; Monsevičius 1997; Bousquet 2003d; Ivinskis et al. 2008; Alekseev 2008c; Vigna Taglianti 2009.

*livens* **(Gyllenhal, 1810)**.Pileckis 1968b, 1976a; Silfverberg 1992, 2004; Pileckis and Monsevičius 1995 (*Agonum*); Monsevičius 1997; Bousquet 2003d; Ferenca 2004; Žiogas and Zolubas 2005; Žiogas and Vaičikauskas 2007b; Alekseev 2008a; Ivinskis et al. 2008; Alekseev 2008c; Vigna Taglianti 2009.

*mannerheimii* **(Dejean, 1828)**. Pileckis 1976a; Silfverberg 1992, 2004; Pileckis and Monsevičius 1995 (*Agonum*); Bousquet 2003d; Alekseev 2008c; Vigna Taglianti 2009.

*Limodromus* **Motschulsky, 1864**.

*longiventris* **(Mannerheim, 1825)**. Pileckis 1976a; Silfverberg 1992, 2004; Pileckis and Monsevičius 1995 (*Agonum*); Bousquet 2003d; Alekseev 2008c; Vigna Taglianti 2009.

*Agonum* **Bonelli, 1810**.

*afrum* **(Duftschmid, 1812)** *= moestum* auct. nec (Duftschmid, 1812). Pileckis 1960, 1976a; Dvilevičius et al. 1988; Silfverberg 1992, 2004; Gaidienė 1993; Pileckis and Monsevičius 1995; Monsevičius 1997; Tamutis and Zolubas 2001; Barševskis 2001a; Bousquet 2003d; Ferenca 2006b; Ferenca et al. 2006, 2007; Vaivilavičius 2008; Ivinskis et al. 2008; Vigna Taglianti 2009.

[*chalconotum* **Ménétriés, 1832 =** *sahlbergii* (Chaudoir, 1850) *= archangelicum* (J.R. Sahlberg, 1875)]. Known in Latvia (Barševskis 2003).

*dolens* **(C.R. Sahlberg, 1827)**. Pileckis 1960, 1976a; Sharova and Grüntal 1973; Bercio and Folwaczny 1979; Silfverberg 1992, 2004; Gaidienė 1993; Pileckis and Monsevičius 1995; Tamutis and Zolubas 2001; Bousquet 2003d; Alekseev 2008c; Vigna Taglianti 2009.

*duftschmidi* **J. Schmidt, 1994** *= moestum* auct. nec (Duftschmid, 1812).# 14. Barševskis 2001a; Bousquet 2003d; Silfverberg 2004; Vaivilavičius 2008; Alekseev 2008c; Vigna Taglianti 2009.

RDB*ericeti* **(Panzer, 1809)**. Pileckis 1976a; Ivinskis et al. 1984, 2004a, 2009; Balevičius 1992; Silfverberg 1992, 2004; Gaidienė 1993; Pileckis and Monsevičius 1995; Monsevičius 1997, 2001; Bousquet 2003d; Dapkus 2004; Tamutis 2005b; Žiogas and Zolubas 2005; Žiogas and Vaičikauskas 2005; Žiogas et al. 2006; Uselis et al. 2007; Rašomavičius 2007; Žiogas and Vaičikauskas 2007a, b; Dapkus and Tamutis 2007, 2008a, b; Vaivilavičius 2008; Alekseev 2008c, 2010b; Vigna Taglianti 2009.

*fuliginosum* **(Panzer, 1809)**. Jakobson 1905-1915; Ogijewicz 1933; Pileckis 1960, 1976a; Sharova and Grüntal 1973; Silfverberg 1992, 2004; Gaidienė 1993; Pileckis and Monsevičius 1995; Monsevičius 1997; Tamutis and Zolubas 2001; Bousquet 2003d; Žiogas and Zolubas 2005; Ferenca 2006b; Lynikienė 2006; Žiogas et al. 2006; Vaivilavičius 2008; Alekseev 2008c; Vigna Taglianti 2009.

*gracile* **Sturm, 1824**. Jakobson 1905-1915; Ogijewicz 1933; Pileckis 1960, 1976a; Sharova and Grüntal 1973; Silfverberg 1992, 2004; Gaidienė 1993; Pileckis and Monsevičius 1995; Monsevičius 1997; Bousquet 2003d; Žiogas et al. 2006; Ferenca 2006b; Alekseev 2008c; Vigna Taglianti 2009.

*gracilipes* **(Duftschmid, 1812)**. Ogijewicz 1933; Pileckis 1960, 1976a; Bercio and Folwaczny 1979; Silfverberg 1992, 2004; Pileckis and Monsevičius 1995; Monsevičius 1997; Bousquet 2003d; Vaivilavičius 2008; Ivinskis et al. 2008; Alekseev 2008c; Vigna Taglianti 2009.

[*hypocrita* **(Apfelbeck, 1904)**].Known in northeastern Poland (Burakowski et al. 1974), Latvia (Barševskis 2003), northwestern Belarus (Alexandrovitch et al. 1996), Kaliningrad region (Vigna Taglianti 2009).

*impressum* **(Panzer, 1796)**. Heyden 1903; Ogijewicz 1933; Pileckis 1960, 1976a; Sharova and Grüntal 1973; Bercio and Folwaczny 1979; Silfverberg 1992, 2004; Pileckis and Monsevičius 1995; Tamutis 1999, 2000, 2005a; Bousquet 2003d; Ferenca 2006b; Alekseev 2008c; Vigna Taglianti 2009.

*lugens* **(Duftschmid, 1812)**.Pileckis 1960, 1976a; Sharova and Grüntal 1973; Silfverberg 1992, 2004; Pileckis and Monsevičius 1995; Bousquet 2003d; Ferenca 2006b; Alekseev 2008c; Vigna Taglianti 2009.

*marginatum* **(Linnaeus, 1758)**. Heyden 1903; Ogijewicz 1933; Pileckis 1960, 1976a; Sharova and Grüntal 1973; Strazdienė 1976; Silfverberg 1992, 2004; Gaidienė 1993; Pileckis and Monsevičius 1995; Bousquet 2003d; Ferenca 2006b; Tamutis et al. 2008; Alekseev 2008a, c; Vigna Taglianti 2009.

*micans* **Nicolai, 1822**. Jakobson 1905-1915; Pileckis 1960, 1976a; Sharova and Grüntal 1973; Silfverberg 1992, 2004; Gaidienė 1993; Pileckis and Monsevičius 1995; Monsevičius 1997; Šablevičius 2000b; Bousquet 2003d; Ferenca 2006b; Alekseev 2008c; Vigna Taglianti 2009.

*muelleri* **(Herbst, 1784)**. Heyden 1903; Ogijewicz 1933; Mazurowa and Mazur 1939; Pileckis 1960, 1976a; Zajančkauskas and Pileckis 1968; Silfverberg 1992, 2004; Gaidienė 1993; Pileckis and Monsevičius 1995; Monsevičius 1997; Tamutis 2000, 2005a; Šablevičius 2000b; Bousquet 2003d; Tamutis et al. 2004, 2007; Ferenca 2006b; Alekseev 2008c; Vigna Taglianti 2009.

[*munsteri* **(Hellén, 1935)**]. Known in Latvia (Barševskis 2003), northwestern Belarus (Alexandrovitch et al. 1996), Estonia, Denmark (Vigna Taglianti 2009).

*piceum* **(Linnaeus, 1758) =** *pelidnum* (Herbst, 1784). Jakobson 1905-1915; Pileckis 1960, 1976a; Zajančkauskas and Pileckis 1968; Bercio and Folwaczny 1979; Silfverberg 1992, 2004; Gaidienė 1993; Pileckis and Monsevičius 1995; Monsevičius 1997; Bousquet 2003d; Ferenca 2006b; Alekseev 2008c; Vigna Taglianti 2009.

[*scitulum* **Dejean, 1828**]. Known in Poland (Burakowski et al. 1974), Latvia (Barševskis 2003), northwestern Belarus (Alexandrovitch et al. 1996), Kaliningrad region, Estonia, (Vigna Taglianti 2009).

*sexpunctatum* **(Linnaeus, 1758)**. Heyden 1903; Ogijewicz 1933; Mazurowa and Mazur 1939; Pileckis 1960, 1976a; Lešinskas and Pileckis 1967; Zajančkauskas and Pileckis 1968; Sharova and Grüntal 1973; Dvilevičius et al. 1988; Silfverberg 1992, 2004; Gaidienė 1993; Pileckis and Monsevičius 1995; Monsevičius 1997; Šablevičius 2000b; Gliaudys 2001; Bousquet 2003d; Ferenca 2006b; Vaivilavičius 2008; Alekseev 2008c; Vigna Taglianti 2009.

*thoreyi* **Dejean, 1828**. Pileckis 1960, 1976a; Sharova and Grüntal 1973; Silfverberg 1992, 2004; Gaidienė 1993; Pileckis and Monsevičius 1995; Monsevičius 1997; Bousquet 2003d; Ferenca 2006b; Žiogas et al. 2006; Žiogas and Vaičikauskas 2007b; Alekseev 2008c; Ivinskis et al. 2009; Vigna Taglianti 2009.

*versutum* **Sturm, 1824**. Miländer et al. 1984; Silfverberg 1992, 2004; Pileckis and Monsevičius 1995; Bousquet 2003d; Alekseev 2008c; Vigna Taglianti 2009.

*viduum* **(Panzer, 1797)**. Ogijewicz 1933; Pileckis 1960, 1976a; Sharova and Grüntal 1973; Dvilevičius et al. 1988; Silfverberg 1992, 2004; Gaidienė 1993; Pileckis and Monsevičius 1995; Monsevičius 1997; Bousquet 2003d; Ferenca 2006b; Vaivilavičius 2008; Alekseev 2008c; Vigna Taglianti 2009.

[*viridicupreum* **(Goeze, 1777)**]. Known in Poland (Burakowski et al. 1974), Kaliningrad region, Estonia, (Vigna Taglianti 2009).

**Panagaenini Bonelli, 1810**.

*Panagaeus* **Latreille, 1802**.

*cruxmajor* **(Linnaeus, 1758)**. Eichwald 1830; Ogijewicz 1933; Pileckis 1960, 1976a; Lešinskas and Pileckis 1967; Sharova and Grüntal 1973; Silfverberg 1992, 2004; Gaidienė 1993; Pileckis and Monsevičius 1995; Monsevičius 1997; Šablevičius 2000b, 2011; Baehr 2003a; Inokaitis 2004; Ferenca 2006b; Alekseev 2008c; Vigna Taglianti 2009.

*bipustulatus* **(Fabricius, 1775)**. Bercio and Folwaczny 1979; Ferenca 2003; Baehr 2003a; Silfverberg 2004; Vaivilavičius 2008; Alekseev 2008c; Vigna Taglianti 2009.

**Chlaeniini Brulle, 1834**.

*Callistus* **Bonelli, 1810**.

[*lunatus* **(Fabricius, 1775)**]. Known in northern Belarus (Alexandrovitch et al. 1996), Kaliningrad region (Vigna Taglianti 2009).

*Chlaenius* **Bonelli, 1810**.

*festivus **(Panzer, 1796)**. # 15. Kirschenhofer 2003.

*spoliatus **(P. Rossi, 1792)**. Pileckis and Monsevičius 1995; Barševskis 2003; disproved by Tamutis et al. (2008).

*Chlaeniellus* **Reitter, 1908**.

[*kindermanni* **Chaudoir, 1856**].Known in Latvia (Barševskis 2003), Kaliningrad region (Vigna Taglianti 2009).

*nigricornis* **(Fabricius, 1787)**. Ogijewicz 1933; Pileckis 1960, 1976a; Zajančkauskas and Pileckis 1968; Sharova and Grüntal 1973; Dvilevičius et al. 1988; Silfverberg 1992, 2004; Gaidienė 1993; Pileckis and Monsevičius 1995 (*Chlaenius*); Monsevičius 1997; Kirschenhofer 2003; Ferenca 2006b; Alekseev 2008c; Vigna Taglianti 2009.

*nitidulus* **(Schrank, 1781)**. Ogijewicz 1933; Pileckis 1960, 1976a; Gaidienė 1993; Silfverberg 1992, 2004; Pileckis and Monsevičius 1995 (*Chlaenius*); Tamutis 2000, 2005a; Ferenca 2006b; Alekseev 2008c.

*tibialis* **Dejean, 1826**. Ferenca 2006b; Vigna Taglianti 2009.

*tristis* **(Schaller, 1783)**. Ogijewicz 1933; Pileckis 1960, 1976a; Zajančkauskas and Pileckis 1968; Sharova and Grüntal 1973; Bercio and Folwaczny 1979; Silfverberg 1992, 2004; Pileckis and Monsevičius 1995 (*Chlaenius*); Monsevičius 1997; Kirschenhofer 2003; Ferenca 2004, 2006b; Alekseev 2008c; Vigna Taglianti 2009.

*vestitus* **(Paykull, 1790)**. Ogijewicz 1933; Mazurowa and Mazur 1939; Pileckis 1960, 1976a; Silfverberg 1992, 2004; Gaidienė 1993; Pileckis and Monsevičius 1995 (*Chlaenius*); Ferenca 2006b; Alekseev 2008a, c; Vigna Taglianti 2009.

*Agostenus* **Fischer von Waldheim, 1829**.

*costulatus* **(Motschulsky, 1859)**. Pileckis 1960, 1976a; Silfverberg 1992, 2004; Pileckis and Monsevičius 1995 (*Chlaenius*); Kirschenhofer 2003; Ivinskis et al. 2008; Alekseev 2008c; Vigna Taglianti 2009.

*quadrisulcatus* **(Paykull, 1790)**. Ivinskis et al. 2008 (*Chlaenius*).

*sulcicollis* **(Paykull, 1798)**.Pileckis 1960, 1976a; Zajančkauskas and Pileckis 1968; Bercio and Folwaczny 1979; Silfverberg 1992, 2004; Gaidienė 1993; Pileckis and Monsevičius 1995 (*Chlaenius*); Monsevičius 1997; Kirschenhofer 2003; Ferenca 2006b; Alekseev 2008c; Vigna Taglianti 2009.

**Oodini LaFerté-Sénectère, 1851**.

*Oodes* **Bonelli, 1810**.

*helopioides* **(Fabricius, 1792)**. Jakobson 1905-1915; Roubal 1910; Ogijewicz 1933; Mazurowa and Mazur 1939; Pileckis 1960, 1976a; Sharova and Grüntal 1973; Dvilevičius et al. 1988; Silfverberg 1992, 2004; Gaidienė 1993; Pileckis and Monsevičius 1995; Monsevičius 1997; Bousquet 2003e; Ferenca 2006b; Vaivilavičius 2008; Ivinskis et al. 2008; Alekseev 2008c; Vigna Taglianti 2009.

*gracilis* **A. Villa & G.B. Villa, 1833**. Barševskis 2001a; Silfverberg 2004; Alekseev 2008c; Vigna Taglianti 2009.

**Licinini Bonelli, 1810**.

*Badister* **Clairville, 1806**.

*bullatus* **(Schrank, 1798) =** *bipustulatus* (Fabricius, 1792) nec (Fabricius, 1775). Heyden 1903; Roubal 1910; Ogijewicz 1933; Pileckis 1960, 1976a; Sharova and Grüntal 1973; Dvilevičius et al. 1988; Silfverberg 1992, 2004; Gaidienė 1993; Pileckis and Monsevičius 1995; Monsevičius 1997; Šablevičius 2000b; Baehr 2003b; Ferenca 2006b; Alekseev 2008c; Vigna Taglianti 2009.

*collaris* **Motschulsky, 1844 =** *anomalus* (Perris, 1866) *= striatulus* Hansen, 1944. Barševskis 2001; Silfverberg 2004; Vigna Taglianti 2009; Alekseev 2008c.

*dilatatus* **Chaudoir, 1837**. Pileckis and Monsevičius 1982, 1995; Silfverberg 1992, 2004; Monsevičius 1997; Baehr 2003b; Ferenca 2004; Vaivilavičius 2008; Alekseev 2008a, c; Vigna Taglianti 2009.

*dorsiger* **(Duftschmid, 1812)**.Tamutis et al. 2008; Vaivilavičius 2008.

*lacertosus* **Sturm, 1815**. Ogijewicz 1933; Pileckis 1960, 1976a; Miländer et al. 1984; Dvilevičius et al. 1988; Silfverberg 1992, 2004; Pileckis and Monsevičius 1995; Monsevičius 1997; Vaivilavičius 2008; Alekseev 2008c.

*meridionalis* **Puel, 1925**. Barševskis 2001a; Silfverberg 2004; Alekseev 2008c; Vigna Taglianti 2009.

*peltatus* **(Panzer, 1796)**. Ogijewicz 1933; Pileckis 1960, 1976a; Sharova and Grüntal 1973; Silfverberg 1992, 2004; Gaidienė 1993; Pileckis and Monsevičius 1995; Monsevičius 1997; Tamutis and Zolubas 2001; Ferenca 2006b; Vaivilavičius 2008; Ivinskis et al. 2008; Alekseev 2008c.

*sodalis* **(Duftschmid, 1812)**. Sharova and Grüntal 1973; Pileckis 1960, 1976a; Silfverberg 1992, 2004; Gaidienė 1993; Pileckis and Monsevičius 1995; Monsevičius 1997; Baehr 2003b; Ferenca 2006b; Ivinskis et al. 2008; Alekseev 2008c; Vigna Taglianti 2009.

*unipustulatus* **Bonelli, 1813**. Heyden 1903; Pileckis 1960, 1976a; Sharova and Grüntal 1973; Silfverberg 1992, 2004; Pileckis and Monsevičius 1995; Monsevičius 1997; Baehr 2003b; Žiogas and Zolubas 2005; Žiogas and Vaičikauskas 2007b; Vaivilavičius 2008 Alekseev 2008c; Vigna Taglianti 2009.

*Licinus* **Latreille, 1802**.

*depressus* **(Paykull, 1790)**. Ogijewicz 1933; Pileckis 1960, 1976a; Miländer et al. 1984; Dvilevičius et al. 1988; Silfverberg 1992, 2004; Gaidienė 1993; Pileckis and Monsevičius 1995; Ferenca et al. 2002; Baehr 2003b; Vaivilavičius 2008; Alekseev 2008c; Vigna Taglianti 2009.

**Harpalini Bonelli, 1810**.

*Ophonus* **Stephens, 1828**.

[*azureus* **(Fabricius, 1775)**]. Known in Latvia (Barševskis 2003), northwestern Poland (Burakowski et al. 1974), northern Belarus (Alexandrovitch et al. 1996), southern Sweden (Lundberg and Gustafsson 1995), Denmark, Kaliningrad region (Vigna Taglianti 2009).

*laticollis* **(Mannerheim, 1825) =** *nitidulus* Stephens, 1828 = *punctatulus* (Duftschmid, 1812) nec (Fabricius, 1792). Heyden 1903; Pileckis 1960, 1976a; Sharova and Grüntal 1973; Silfverberg 1992, 2004; Gaidienė 1993; Pileckis and Monsevičius 1995 (*Harpalus*); Monsevičius 1997; Kataev et al. 2003; Ferenca 2006b; Vaivilavičius 2008; Alekseev 2008c; Vigna Taglianti 2009.

[*melletii* **(Heer, 1837)**]. Known in northwestern Poland (Burakowski et al. 1974), southern Sweden (Lundberg and Gustafsson 1995), Denmark, Kaliningrad region (Vigna Taglianti 2009).

[*puncticeps* **Stephens, 1828**]. Known in Latvia (Barševskis 2003), northwestern Poland (Burakowski et al. 1974), southern Sweden (Lundberg and Gustafsson 1995), Denmark (Vigna Taglianti 2009).

*puncticollis* **(Paykull, 1798)**. Ogijewicz 1933; Pileckis 1960, 1976a; Silfverberg 1992, 2004; Gaidienė 1993; Pileckis and Monsevičius 1995 (*Harpalus*); Ferenca 2006b; Alekseev 2008c.

*rufibarbis* **(Fabricius, 1792) =** *seladon* (Schauberger, 1926).Heyden 1903; Pileckis 1960, 1976a; Silfverberg 1992, 2004; Gaidienė 1993; Pileckis and Monsevičius 1995 (*Harpalus*); Monsevičius 1997; Kataev et al. 2003; Ferenca 2006b; Vaivilavičius 2008; Alekseev 2008c; Vigna Taglianti 2009.

[*rupicola* **(Sturm, 1818)**].Known in Latvia (Barševskis 2003), northwestern Poland (Burakowski et al. 1974), southern Sweden (Lundberg and Gustafsson 1995), Kaliningrad region (Vigna Taglianti 2009).

*schaubergerianus* **(Puel, 1937)**. Tamutis et al. 2008.

[*stictus* **Stephens, 1828**]. Known in Latvia and Kaliningrad region (Vigna Taglianti 2009).

*Pseudoophonus* **Motschulsky, 1844**.

*calceatus* **(Duftschmid, 1812)**. Ogijewicz 1933; Mazurowa and Mazur 1939; Pileckis 1960, 1976a; Bercio and Folwaczny 1979; Silfverberg 1992, 2004; Gaidienė 1993; Pileckis and Monsevičius 1995 (*Harpalus*); Monsevičius 1997; Kataev et al. 2003; Ferenca 2006b; Tamutis et al. 2008; Alekseev 2008c; Vigna Taglianti 2009.

*griseus* **(Panzer, 1796)**. Ogijewicz 1933; Mazurowa and Mazur 1939; Pileckis 1960, 1976a; Gaidienė 1993; Silfverberg 1992, 2004; Pileckis and Monsevičius 1995 (*Harpalus*); Monsevičius 1997; Kataev et al. 2003; 2004; Tamutis 2000, 2005a; Ferenca 2006b; Alekseev 2008c; Vigna Taglianti 2009.

*rufipes* **(DeGeer, 1774) =** *pubescens* (O.F. Müller, 1776). Eichwald 1830; Ogijewicz 1933; Mazurowa and Mazur 1939; Pileckis 1960, 1976a; Lešinskas and Pileckis 1967; Zajančkauskas and Pileckis 1968; Pileckis et al. 1968; Sharova and Grüntal 1973; Pileckis and Vengeliauskaitė 1977, 1996; Dvilevičius et al. 1988; Silfverberg 1992, 2004; Gaidienė 1993; Pileckis and Monsevičius 1995 (*Harpalus*); Monsevičius 1997; Tamutis 1999, 2000, 2005a; Šablevičius 2000b; Gliaudys 2001; Kataev et al. 2003; Tamutis et al. 2004, 2006, 2007; Žiogas and Zolubas 2005; Ferenca 2006b; Lynikienė 2006; Žiogas and Vaičikauskas 2007b; Vaivilavičius 2008; Ivinskis et al. 2008; Alekseev 2008c; Vigna Taglianti 2009.

*Cryptophonus* **Brandmayr & Zetto- Brandmayr, 1882**.

[*melancholicus **(Dejean, 1829)**]. # 16. Tamutis and Ferenca 2006; Alekseev 2008c; disproved by Tamutis et al. (2008).

*Semiophonus* **Schauberger 1933**.

*signaticornis* **(Duftschmid, 1812)**. Tamutis 1999, 2000, 2005a; Silfverberg 2004; Tamutis et al. 2004; Alekseev 2008c.

*Harpalus* **Latreille, 1802**.

*affinis* **(Schrank, 1781) =** *aeneus* (Fabricius, 1775) nec (DeGeer, 1774). Eichwald 1830; Heyden 1903; Roubal 1910; Ogijewicz 1933; Mazurowa and Mazur 1939; Pileckis 1960, 1976a; Lešinskas and Pileckis 1967; Zajančkauskas and Pileckis 1968; Sharova and Grüntal 1973; Pileckis and Vengeliauskaitė 1977, 1996; Dvilevičius et al. 1988; Silfverberg 1992, 2004; Gaidienė 1993; Pileckis and Monsevičius 1995; Monsevičius 1997; Tamutis 1999, 2000, 2005a; Šablevičius 2000b; Gliaudys 2001; Kataev et al. 2003; Tamutis et al. 2004, 2006, 2007; Ferenca 2006b; Vaivilavičius 2008; Alekseev 2008c; Vigna Taglianti 2009.

*anxius* **(Duftschmid, 1812)**. Heyden 1903; Ogijewicz 1933; Pileckis 1960, 1976a; Bercio and Folwaczny 1979; Silfverberg 1992, 2004; Gaidienė 1993; Pileckis and Monsevičius 1995; Kataev et al. 2003; Ferenca 2006b; Alekseev 2008c; Vigna Taglianti 2009.

*atratus **Latreille, 1804**. # 17. Pileckis 1960; Gaidienė 1993; Silfverberg 2004.

*autumnalis* **(Duftschmid, 1812)**. Ogijewicz 1933; Pileckis 1960, 1976a Silfverberg 1992, 2004; Gaidienė 1993; Pileckis and Monsevičius 1995; Monsevičius 1997; Ferenca 2006b; Alekseev 2008c; Vigna Taglianti 2009.

*caspius **(Steven, 1806)** *= roubali* Schauberger, 1928. # 18. Pileckis and Monsevičius 1995; Barševskis 2003.

*dimidiatus **(P. Rossi, 1790)**. # 19. Pileckis 1960; Pileckis and Monsevičius 1995; Ferenca 2006b.

*distinguendus* **(Duftschmid, 1812)**. Pileckis 1960, 1976a; Sharova and Grüntal 1973; Silfverberg 1992, 2004; Pileckis and Monsevičius 1995; Tamutis 1999, 2000, 2005a; Kataev et al. 2003; Tamutis et al. 2004; Ferenca 2006b; Alekseev 2008c; Vigna Taglianti 2009; Ivinskis et al. 2009.

*flavescens* **(Piller & Mitterpacher, 1783) =** *rufus* Brüggemann, 1873. Heyden 1903; Ogijewicz 1933; Mazurowa and Mazur 1939; Pileckis 1960, 1976a; Silfverberg 1992, 2004; Gaidienė 1993; Pileckis and Monsevičius 1995; Kataev et al. 2003; Alekseev 2008a, c; Vigna Taglianti 2009.

*froelichii* **Sturm, 1818**. Ogijewicz 1933; Pileckis 1960, 1976a; Silfverberg 1992, 2004; Gaidienė 1993; Pileckis and Monsevičius 1995; Monsevičius 1997; Kataev et al. 2003; Alekseev 2008c; Vigna Taglianti 2009.

*hirtipes* **(Panzer, 1797)**. Eichwald 1830; Ogijewicz 1933; Pileckis 1960, 1976a; Silfverberg 1992, 2004; Gaidienė 1993; Pileckis and Monsevičius 1995; Ferenca et al. 2002; Kataev et al. 2003; Ferenca 2006b; Alekseev 2008c; Vigna Taglianti 2009.

[*honestus* **(Duftschmid, 1812)**]. Known in northwestern Poland (Burakowski et al. 1974), Belarus (Alexandrovitch et al. 1996).

*laevipes* **Zetterstedt, 1828 =** *quadripunctatus* Dejean, 1829. Ogijewicz 1933; Pileckis 1960, 1976a; Sharova and Grüntal 1973; Dvilevičius et al. 1988; Silfverberg 1992, 2004; Gaidienė 1993; Pileckis and Monsevičius 1995; Monsevičius 1997; Tamutis and Zolubas 2001; Kataev et al. 2003; Žiogas and Zolubas 2005; Žiogas et al. 2006; Ferenca 2006b; Lynikienė 2006; Žiogas and Vaičikauskas 2007b; Vaivilavičius 2008; Alekseev 2008c; Vigna Taglianti 2009.

*latus* **(Linnaeus, 1758)**. Ogijewicz 1933; Mazurowa and Mazur 1939; Pileckis 1960, 1976a; Sharova and Grüntal 1973; Dvilevičius et al. 1988; Silfverberg 1992, 2004; Gaidienė 1993; Pileckis and Monsevičius 1995; Monsevičius 1997; Šablevičius 2000b; Gliaudys 2001; Kataev et al. 2003; Ferenca 2006b; Alekseev 2008c; Vigna Taglianti 2009.

*luteicornis* **(Duftschmid, 1812)**. Heyden 1903; Pileckis 1960, 1976a; Dvilevičius et al. 1988; Silfverberg 1992, 2004; Gaidienė 1993; Pileckis and Monsevičius 1995; Monsevičius 1997; Kataev et al. 2003; Ferenca 2006b; Tamutis et al. 2007; Alekseev 2008c; Vigna Taglianti 2009.

*modestus* **(Dejean, 1829)**. Pileckis 1960, 1976a; Silfverberg 1992, 2004; Gaidienė 1993; Pileckis and Monsevičius 1995; Kataev et al. 2003; Ferenca 2006b; Vigna Taglianti 2009.

*neglectus* **Audinet-Serville, 1821**. Bercio and Folwaczny 1979; Pileckis and Monsevičius 1982, 1995; Silfverberg 1992, 2004; Alekseev 2008c.

*picipennis* **(Duftschmid, 1812)**. Pileckis and Monsevičius 1982, 1995; Silfverberg 1992, 2004; Gaidienė 1993; Barševskis 2001a; Alekseev 2008c.

*progrediens* **Schauberger, 1922**. Tamutis et al. 2008.

*pumilus* **(Sturm, 1818) =** *vernalis* (Fabricius, 1801) nec (Panzer, 1796). Monsevičius and Pankevičius 2001; Barševskis 2001a; Silfverberg 2004; Alekseev 2008c; Vigna Taglianti 2009.

*rubripes* **(Duftschmid, 1812)**. Ogijewicz 1933; Pileckis 1960, 1976a; Bercio and Folwaczny 1979; Silfverberg 1992, 2004; Gaidienė 1993; Pileckis and Monsevičius 1995; Kataev et al. 2003; Alekseev 2008c; Vigna Taglianti 2009.

*rufipalpis* **Sturm, 1818 =** *rufitarsis* (Duftschmid, 1812) nec (Illiger, 1802). Ogijewicz 1933; Pileckis 1960, 1976a; Silfverberg 1992, 2004; Gaidienė 1993; Pileckis and Monsevičius 1995; Monsevičius 1997; Kataev et al. 2003; Ferenca 2006b; Alekseev 2008c; Vigna Taglianti 2009.

*serripes* **(Quensel, 1806)**. Pileckis 1960, 1976a; Silfverberg 1992, 2004; Gaidienė 1993; Pileckis and Monsevičius 1995; Barševskis 2003; Kataev et al. 2003; Alekseev 2008c; Vigna Taglianti 2009.

*servus* **(Duftschmid, 1812)**. Pileckis 1976a; Bercio and Folwaczny 1979; Silfverberg 1992, 2004; Pileckis and Monsevičius 1995; Kataev et al. 2003; Alekseev 2008c; Vigna Taglianti 2009.

*smaragdinus* **(Duftschmid, 1812)**. Heyden 1903; Ogijewicz 1933; Mazurowa and Mazur 1939; Pileckis 1960, 1976a; Bercio and Folwaczny 1979; Silfverberg 1992, 2004; Gaidienė 1993; Pileckis and Monsevičius 1995; Monsevičius 1997; Kataev et al. 2003; Ferenca 2006b; Alekseev 2008c; Vigna Taglianti 2009.

*solitaris* **Dejean, 1829 =** *fuliginosus* (Duftschmid, 1812) nec (Panzer, 1809). Gaidienė 1993; Pileckis and Monsevičius 1995; Monsevičius 1997; Silfverberg 2004; Alekseev 2008c.

*tardus* **(Panzer, 1796)**. Heyden 1903; Ogijewicz 1933; Mazurowa and Mazur 1939; Pileckis 1960, 1976a; Silfverberg 1992, 2004; Gaidienė 1993; Pileckis and Monsevičius 1995; Monsevičius 1997; Kataev et al. 2003; Lynikienė 2006; Ferenca 2006b; Alekseev 2008c; Vigna Taglianti 2009.

*xanthopus winkleri* **Schauberger, 1923**. Gaidienė and Ferenca 1988; Silfverberg 1992, 2004; Gaidienė 1993; Pileckis and Monsevičius 1995; Tamutis et al. 2008; Alekseev 2008a, c.

[*zabroides* **Dejean, 1829**]. Known in northern Belarus (Alexandrovitch et al. 1996), Kaliningrad region (Vigna Taglianti 2009).

**A***nisodactylus* **Dejean, 1829**.

*binotatus* **(Fabricius, 1787)**. Heyden 1903; Ogijewicz 1933; Pileckis 1960, 1976a; Pileckis 1963b; Zajančkauskas and Pileckis 1968; Sharova and Grüntal 1973 Silfverberg 1992, 2004; Gaidienė 1993; Šablevičius and Ferenca 1995; Pileckis and Monsevičius 1995; Monsevičius 1997; Tamutis 2000, 2005a; Šablevičius 2000b; Tamutis and Zolubas 2001; Ito 2003; Tamutis et al. 2004, 2006, 2007; Ferenca 2006b; Vaivilavičius 2008; Alekseev 2008c; Vigna Taglianti 2009.

*nemorivagus* **(Duftschmid, 1812)**. Ogijewicz 1933; Pileckis 1960, 1976a; Silfverberg 1992, 2004; Gaidienė 1993; Pileckis and Monsevičius 1995; Alekseev 2008c.

[*poeciloides* **(Stephens, 1828)**]. Known in central Poland (Burakowski et al. 1974), southern Sweden (Lundberg and Gustafsson 1995), Denmark (Vigna Taglianti 2009).

[*signatus **(Panzer, 1797)**]. # 20. Gaidienė 1993; Silfverberg 2004; Alekseev 2008c.

*Diachromus* **Erichson, 1837**.

*germanus* **(Linnaeus, 1758)**. Pileckis 1960, 1963b, 1970a, 1976a, b, 1979; Sharova and Grüntal 1973; Silfverberg 1992, 2004; Gaidienė 1993; Pileckis and Monsevičius 1995; Monsevičius 1997; Tamutis 2000, 2005b; Ferenca et al. 2002, 2006; Ferenca 2006b; Vaivilavičius 2008; Alekseev 2008c.

*Stenolophus* **Dejean, 1821**.

[*discophorus* **(Fischer von Waldheim, 1823)**]. Known in northwestern Belarus (Alexandrovitch et al. 1996), Kaliningrad region (Vigna Taglianti 2009); central Poland (Burakowski et al. 1974).

*mixtus* **(Herbst, 1784)**. Sharova and Grüntal 1973; Pileckis 1976a; Silfverberg 1992, 2004; Gaidienė 1993; Pileckis and Monsevičius 1995 (*Acupalpus*); Monsevičius 1997; Ferenca 2004; Alekseev 2008a; Ivinskis et al. 2008; Alekseev 2008c.

[*skrimshiranus* **Stephens, 1828**]. Known in northeastern Belarus (Alexandrovitch et al. 1996), southern Sweden (Lundberg and Gustafsson 1995), Denmark (Vigna Taglianti 2009); central Poland (Burakowski et al. 1974).

*teutonus* **(Schrank, 1781)**. Ferenca 1988, 2004; Silfverberg 1992, 2004; Gaidienė 1993; Pileckis and Monsevičius 1995 (*Acupalpus*); Ferenca et al. 2002; Vaivilavičius 2008; Alekseev 2008c.

*Bradycellus* **Erichson, 1837**.

*caucasicus* **(Chaudoir, 1846)** *= collaris* (Paykull, 1798) nec (Herbst, 1784). Pileckis 1960, 1976a; Pileckis and Monsevičius 1982, 1995; Silfverberg 1992, 2004; Gaidienė 1993; Monsevičius 1997; Jaeger and Kataev 2003; Alekseev 2008c; Vigna Taglianti 2009.

[*csikii **Laczó, 1912**].# 21.Tamutis et al. 2008.

*harpalinus* **(Audinet-Serville, 1821)**. Silfverberg 1992, 2004; Pileckis and Monsevičius 1995; Alekseev 2008c; Ivinskis et al. 2009.

*ruficollis* **(Stephens, 1828) =** *similis* (Dejean, 1829). Pileckis 1960, 1976a; Bercio and Folwaczny 1979; Silfverberg 1992, 2004, Pileckis and Monsevičius 1995; Monsevičius 1997; Jaeger and Kataev 2003; Ferenca 2006b; Ivinskis et al. 2008; Alekseev 2008c.

[*verbasci* **(Duftschmid, 1812)**]. Known in northeastern Poland (Alexandrovitch et al. 2003), southern Sweden (Lundberg and Gustafsson 1995), Kaliningrad region (Alekseev and Bukejs 2010), Denmark (Vigna Taglianti 2009).

*Dicheirotrichus* **Jacquelin du Val, 1857**.

*cognatus* **(Gyllenhal 1827)**. Pileckis and Monsevičius 1982, 1995 (*Trichocellus*); Silfverberg 1992, 2004; Vaivilavičius 2008; Alekseev 2008c.

[*gustavii* **Crotch, 1871** *= pubescens* (Paykull, 1790) nec (O.F. Müller, 1776)]. Known in northwestern Poland (Burakowski et al. 1974), southern Sweden (Lundberg and Gustafsson 1995), Denmark (Vigna Taglianti 2009).

*placidus* **(Gyllenhal 1827)**. Pileckis 1976a; Gaidienė 1993; Silfverberg 1992, 2004; Pileckis and Monsevičius 1995 (*Trichocellus*); Monsevičius 1997; Jaeger and Kataev 2003; Alekseev 2008c; Vigna Taglianti 2009.

*rufithorax* **(C.R. Sahlberg, 1827)**. Pileckis 1960, 1976a, b, 1979; Pileckis and Monsevičius 1982, 1995; Silfverberg 1992, 2004; Gaidienė 1993; Jaeger and Kataev 2003; Ferenca 2006b; Vaivilavičius 2008; Alekseev 2008c; Vigna Taglianti 2009.

*Acupalpus* **Dejean, 1829**.

*brunnipes* **(Sturm, 1825)**. Silfverberg 1992, 2004; Pileckis and Monsevičius 1995; Jaeger and Kataev 2003; Alekseev 2008c; Vigna Taglianti 2009.

[*dubius* **Schilsky, 1888**]. Known in Latvia (Barševskis 2003), central and northwestern Poland (Burakowski et al. 1974), southern Sweden (Lundberg and Gustafsson 1995), Estonia, Denmark (Vigna Taglianti 2009).

[*elegans* **(Dejean, 1829)**]. Known in Denmark (Silfverberg 2004), Poland (Burakowski et al. 1974).

*exiguus* **Dejean, 1829**. Sharova and Grüntal 1973; Silfverberg 1992, 2004; Pileckis and Monsevičius 1995; Ferenca et al. 2006; Alekseev 2008c.

*flavicollis* **(Sturm, 1825)**.Pileckis 1976a; Silfverberg 1992, 2004; Gaidienė 1993; Pileckis and Monsevičius 1995; Monsevičius 1997; Tamutis and Zolubas 2001; Jaeger and Kataev 2003; Alekseev 2008c; Vigna Taglianti 2009.

[*interstitialis* **Reitter, 1884**]. Known in Latvia (Barševskis 2003), Estonia (Vigna Taglianti 2009).

*luteatus* **(Duftschmid, 1812)**. Ferenca et al. 2006; Tamutis and Ferenca 2006; Alekseev 2008c.

*meridianus* **(Linnaeus, 1761)**. Ogijewicz 1933; Pileckis 1960, 1976a; Sharova and Grüntal 1973; Silfverberg 1992, 2004; Gaidienė 1993; Pileckis and Monsevičius 1995; Monsevičius 1997; Tamutis 2000, 2005a; Tamutis and Zolubas 2001; Jaeger and Kataev 2003; Tamutis et al. 2004, 2006, 2007; Žiogas and Zolubas 2005; Ferenca 2006b; Vaivilavičius 2008; Alekseev 2008c; Vigna Taglianti 2009.

*parvulus* **(Sturm, 1825) =** *dorsalis* (Fabricius, 1787) nec (Pontoppidan, 1763). Ogijewicz 1933; Mazurowa and Mazur 1939; Pileckis 1960, 1976a; Silfverberg 1992, 2004; Gaidienė 1993; Pileckis and Monsevičius 1995; Monsevičius 1997; Tamutis 2000, 2005a; Jaeger and Kataev 2003; Tamutis et al. 2004; Ferenca 2006b; Alekseev 2008c; Vigna Taglianti 2009.

*suturalis* **Dejean, 1829**. Ferenca et al. 2006; Tamutis and Ferenca 2006; Alekseev 2008c.

*Anthracus* **Motschulsky, 1850**.

*consputus* **(Duftschmid, 1812)**. Sharova and Grüntal 1973; Pileckis 1976a; Silfverberg 1992, 2004; Gaidienė 1993; Pileckis and Monsevičius 1995 (*Acupalpus*); Monsevičius 1997; Jaeger and Kataev 2003; Alekseev 2008a, c; Ivinskis et al. 2009; Vigna Taglianti 2009.

**Perigonini Horn, 1881**.

*Trechicus* **LeConte, 1853** *=* *Perigona* Laporte, 1835.

*nigriceps* **(Dejean, 1831)**. Ferenca and Tamutis 2009.

**Cyclosomini Laporte, 1834**.

*Masoreus* **Dejean, 1821**.

*wetterhallii* **(Gyllenhal, 1813)**. Mazurowa and Mazur 1939; Pileckis 1960, 1970a, 1976a; Bercio and Folwaczny 1979; Silfverberg 1992, 2004; Pileckis and Monsevičius 1995; Ferenca et al. 2002, 2006; Bousquet 2003f; Žiogas and Zolubas 2005; Ivinskis and Rimšaitė 2005; Alekseev 2008c; Vigna Taglianti 2009.

**Odacanthini Laporte, 1834**.

*Odacantha* **Paykull, 1798**.

*melanura* **(Linnaeus, 1767)**. Pileckis 1960, 1963b, 1976a; Zajančkauskas and Pileckis 1968; Sharova and Grüntal 1973; Silfverberg 1992, 2004; Gaidienė 1993; Pileckis and Monsevičius 1995; Monsevičius 1997; Ivinskis et al. 1998; Bousquet and Ito 2003; Ferenca 2006b; Vaivilavičius 2008; Alekseev 2008c; Vigna Taglianti 2009.

**Lebiini Bonelli, 1810**.

*Lamprias* **Bonelli, 1810**.

*chlorocephalus* **(J.J. Hoffmann, 1803)**. Ogijewicz 1933; Pileckis 1960, 1976a; Silfverberg 1992, 2004; Gaidienė 1993; Pileckis and Monsevičius 1995 (*Lebia*); Ferenca et al. 2002, 2006; Šablevičius 2003a, 2011; Kabak 2003; Inokaitis 2004; Vaivilavičius 2008; Alekseev 2008c; Ivinskis et al. 2009.

*cyanocephala* **(Linnaeus, 1758)**. Ogijewicz 1933; Pileckis 1960, 1976a; Silfverberg 1992, 2004; Pileckis and Monsevičius 1995 (*Lebia*); Kabak 2003; Alekseev 2008c.

*Lebia* **Latreille, 1802**.

*cruxminor* **(Linnaeus, 1758)**. Ogijewicz 1933; Pileckis 1960, 1976a; Silfverberg 1992, 2004; Gaidienė 1993; Pileckis and Monsevičius 1995; Kabak 2003; Alekseev 2008c; Inokaitis 2009; Vigna Taglianti 2009.

**D***emetrias* **Bonelli, 1810**.

[*atricapillus* **(Linnaeus, 1758)**]. Known in Latvia (Barševskis 2003), northwestern Poland (Burakowski et al. 1974), southern Sweden (Lundberg and Gustafsson 1995), Kaliningrad region (Alekseev 2008c), Denmark, Estonia (Vigna Taglianti 2009).

*imperialis* **(Germar, 1824)**. Silfverberg 1992, 2004; Gaidienė 1993; Pileckis and Monsevičius 1995; Barševskis 2001; Ferenca 2004; Alekseev 2008c; Vigna Taglianti 2009.

*monostigma* **Samouelle, 1819**. Jakobson 1905-1915; Pileckis 1976a; Silfverberg 1992, 2004; Gaidienė 1993; Pileckis and Monsevičius 1995; Monsevičius 1997; Ferenca et al. 2002, 2006; Šablevičius 2003a, b; Kabak 2003; Ferenca 2004; Alekseev 2008c; Vigna Taglianti 2009.

**P***aradromius* **Fowler, 1887**.

*linearis* **(Olivier, 1795)**. Pileckis 1976a; Silfverberg 1992, 2004; Pileckis and Monsevičius 1995 (*Dromius*); Barševskis 2001; Šablevičius 2001, 2003a; Ferenca et al. 2002, 2006; Alekseev 2008c; Vigna Taglianti 2009.

*longiceps* **(Dejean, 1826)**. Kabak 2003; Tamutis et al. 2008; Vigna Taglianti 2009.

*Dromius* **Bonelli, 1810**.

*agilis* **(Fabricius, 1787)**. Ogijewicz 1933; Pileckis 1960, 1976a; Silfverberg 1992, 2004; Gaidienė 1993; Pileckis and Monsevičius 1995; Monsevičius 1997; Kabak 2003; Alekseev 2008c; Vigna Taglianti 2009; Ostrauskas and Ferenca 2010.

[*angusticollis* **J.R. Sahlberg, 1880**]. Known in northwestern Belarus (Alexandrovitch et al. 1996), Kaliningrad region (Vigna Taglianti 2009).

[*angustus* **Brullé, 1834**]. Known in central and northwestern Poland (Burakowski et al. 1974), southern Sweden (Lundberg and Gustafsson 1995), Kaliningrad region (Alekseev 2008c), Estonia, Denmark (Vigna Taglianti 2009).

*fenestratus* **(Fabricius, 1794)**. Jakobson 1905-1915; Pileckis 1976a; Bercio and Folwaczny 1979; Silfverberg 1992, 2004; Gaidienė 1993; Pileckis and Monsevičius 1995; Monsevičius 1997; Šablevičius 2001, 2003a; Alekseev 2008c.

[*laeviceps* **Motschulsky,** **1850**]. Recently discovered in Latvia (Telnov et al. 2008), known in Poland (Burakowski et al. 1974), Kaliningrad region, Belarus (Vigna Taglianti 2009).

*quadraticollis* **Morawitz, 1862**. Pileckis 1976a; Silfverberg 1992, 2004; Gaidienė 1993; Pileckis and Monsevičius 1995; Monsevičius 1997; Alekseev 2008c; Ostrauskas and Ferenca 2010.

*quadrimaculatus* **(Linnaeus, 1758)**. Ogijewicz 1933; Pileckis 1960, 1976a; Silfverberg 1992, 2004; Gaidienė 1993; Pileckis and Monsevičius 1995; Kabak 2003; Ferenca 2004; Žiogas and Zolubas 2005; Vaivilavičius 2008; Alekseev 2008c; Vigna Taglianti 2009.

*schneideri* **Crotch, 1871 =** *marginellus* (Fabricius, 1794) nec (Herbst, 1784). Ogijewicz 1933; Pileckis 1960, 1976a; Dvilevičius et al. 1988; Silfverberg 1992, 2004; Gaidienė 1993; Žiogas and Gedminas 1994; Pileckis and Monsevičius 1995; Kabak 2003; Ferenca 2006b; Alekseev 2008c; Vigna Taglianti 2009.

*Calodromius* **Reitter, 1905**.

*spilotus* **(Illiger, 1798) =** *quadrinotatus* (Panzer, 1800) nec (Fabricius, 1798). Bercio and Folwaczny 1979; Pileckis and Monsevičius1982, 1995 (*Dromius*); Ferenca 1988; Silfverberg 1992, 2004; Gaidienė 1993; Alekseev 2008c.

*Philorhizus* **Hope, 1838**.

[*melanocephalus* **(Dejean, 1825)**]. Known in northern Poland (Burakowski et al. 1974), southern Sweden (Lundberg and Gustafsson 1995), Estonia, Denmark (Vigna Taglianti 2009).

*notatus* **(Stephens, 1827) =** *nigriventris* (Thomson, 1857). Tamutis et al. 2008; Alekseev 2008c.

[*quadrisignatus* **(Dejean, 1825)**]. Known in Belarus, Denmark, northern Russia (Vigna Taglianti 2009), southern Sweden (Lundberg and Gustafsson 1995), Poland (Burakowski et al. 1974).

*sigma* **(P. Rossi, 1790)**. Pileckis 1976a; Silfverberg 1992, 2004; Gaidienė 1993; Pileckis and Monsevičius 1995 (*Dromius*); Monsevičius 1997; Ivinskis et al. 1998; Alekseev 2008c.

*Microlestes* **Schmidt-Goebel, 1846**.

*maurus* **(Sturm, 1827)**. Mazurowa and Mazur 1939; Pileckis 1960, 1976a; Silfverberg 1992, 2004; Pileckis and Monsevičius 1995; Žiogas and Zolubas 2005; Ferenca 2006b; Alekseev 2008c.

*minutulus* **(Goeze, 1777)**. Sharova and Grüntal 1973; Pileckis 1976a; Silfverberg 1992, 2004; Pileckis and Monsevičius 1995; Monsevičius 1997; Tamutis 2000, 2005a; Alekseev 2008c.

*Syntomus* **Hope, 1838**.

[*ai* **Barševskis, 1993**]. # 22. Known in Latvia (Barševskis 1993).

*foveatus* **(Geoffroy, 1785)**. Ogijewicz 1933; Pileckis 1960, 1976a; Silfverberg 1992, 2004; Gaidienė 1993; Pileckis and Monsevičius 1995; Monsevičius 1997; Kabak 2003; Ferenca 2006b; Alekseev 2008c; Vigna Taglianti 2009.

[*obscuroguttatus* **(Duftschmid, 1812)**]. Known in Kaliningrad region (Bercio and Folwaczny 1979), northeastern Poland (Alexandrovitch et al. 2003).

*truncatellus* **(Linnaeus, 1761)**. Ogijewicz 1933; Mazurowa and Mazur 1939; Pileckis 1960, 1976a; Dvilevičius et al. 1988; Gaidienė 1993; Silfverberg 1992, 2004; Pileckis and Monsevičius 1995; Monsevičius 1997; Ferenca et al. 2002; Kabak 2003; Alekseev 2008c; Vigna Taglianti 2009.

*Lionychus* **Wissmann, 1846**.

*quadrillum* **(Duftschmid, 1812)**. Ferenca et al. 2002, 2006; Ivinskis and Rimšaitė 2005; Alekseev 2008c.

*Cymindis* **Latreille, 1806**.

*angularis* **Gyllenhal, 1810**.Pileckis 1976a; Pileckis and Monsevičius 1995; Silfverberg 1992, 2004; Ferenca 2006b; Alekseev 2008c.

*axillaris **(Fabricius, 1794)**. Pileckis 1960, 1976a; disproved by Pileckis and Monsevičius (1995).

*humeralis* **(Geoffroy, 1785)**. Miländer et al. 1984; Pileckis and Monsevičius 1995; Silfverberg 1992, 2004; Alekseev 2008c.

*macularis* **Mannerheim 1824**. Pileckis 1976a; Silfverberg 1992, 2004; Pileckis and Monsevičius 1995; Monsevičius 1997; Alekseev 2008c.

*vaporariorum* **(Linnaeus, 1758)**. Pileckis 1976a; Bercio and Folwaczny 1979; Silfverberg 1992, 2004; Gaidienė 1993; Pileckis and Monsevičius 1995; Alekseev 2008c.

**HALIPLIDAE Brullé, 1835**.

*Brychius* **Thomson, 1859**.

*elevatus cristatus* **J.R. Sahlberg, 1875**.Ogijewicz 1933; Pileckis 1960, 1976a; Bercio and Folwaczny 1979; Silfverberg 1992, 2004; Gaidienė 1993; Pileckis and Monsevičius 1995; Kovács et al. 2008; Alekseev 2010a.

*Peltodytes* **Régimbart, 1879**.

*caesus* **(Duftschmid, 1805)**.Pileckis 1960, 1976a; Silfverberg 1992, 2004; Pileckis and Monsevičius 1995; Alekseev 2010a.

*Haliplus* **Latreille, 1802**.

*apicalis* **Thomson, 1868**.Bercio and Folwaczny 1979; Silfverberg 1992, 2004; Alekseev 2010a.

*confinis* **Stephens, 1828**.Ogijewicz 1933; Pileckis 1960, 1976a; Bercio and Folwaczny 1979; Silfverberg 1992, 2004; Gaidienė 1993; Pileckis and Monsevičius 1995; Monsevičius 1997; Šablevičius 2000b; Kovács et al. 2008; Alekseev 2010a.

*flavicollis* **Sturm, 1834**.Eichwald 1830;Ogijewicz 1933; Pileckis 1960, 1976a; Bercio and Folwaczny 1979; Silfverberg 1992, 2004; Gaidienė 1993; Pileckis and Monsevičius 1995; Šablevičius 2000b; Ferenca 2006b; Vaivilavičius 2008; Kovács et al. 2008; Alekseev 2010a.

*fluviatilis* **Aubé, 1836**.Ogijewicz 1933; Lešinskas and Pileckis 1967; Pileckis 1960, 1976a; Gaidienė 1993; Silfverberg 1992, 2004; Pileckis and Monsevičius 1995; Alekseev 2010a.

[*fulvicollis* **Erichson, 1837**]. Known in Kaliningrad region (Alekseev 2010a), northeastern Poland (Burakowski et al. 1976), Latvia (Barševskis et al. 2005), western Belarus (Alexandrovitch et al. 1996).

*fulvus* **(Fabricius, 1801) =** *lapponum* Thomson, 1856.Ogijewicz 1933; Pileckis 1960, 1976a; Silfverberg 1992, 2004; Gaidienė 1993; Pileckis and Monsevičius 1995; Alekseev 2010a.

*furcatus* **Seidlitz, 1887**.Silfverberg 1992, 2004; Pileckis and Monsevičius 1995; Šablevičius 2000b; Alekseev 2010a.

*heydeni* **Wehncke, 1875**. Ogijewicz 1933; Pileckis 1960, 1976a; Bercio and Folwaczny 1979; Silfverberg 1992, 2004; Gaidienė 1993; Pileckis and Monsevičius 1995; Alekseev 2010a.

*immaculatus* **Gerhardt, 1877**.Ogijewicz 1933; Pileckis 1960, 1976a; Silfverberg 1992, 2004; Pileckis and Monsevičius 1995; Alekseev 2010a.

[*interjectus* **H. Lindberg, 1937**].Known in Latvia (Barševskis et al. 2005).

*laminatus* **(Schaller, 1783)**. Pileckis 1960, 1970b, 1976a; Silfverberg 1992, 2004; Pileckis and Monsevičius 1995; Ferenca 2006b; Alekseev 2010a.

*lineatocollis* **(Marsham, 1802)**.Pileckis 1960, 1976a; Silfverberg 1992, 2004; Pileckis and Monsevičius 1995; Ferenca 2006b; Alekseev 2010a.

*lineolatus* **Mannerheim, 1844 =** *nomax* Balfour-Browne, 1911.# 23. Ogijewicz 1933; Pileckis 1960, 1976a; Pileckis and Monsevičius 1995; Monsevičius 1997.

*obliquus* **(Fabricius, 1787)**.Ogijewicz 1933; Mazurowa and Mazur 1939; Pileckis 1960, 1976a; Silfverberg 1992, 2004; Pileckis and Monsevičius 1995; Monsevičius 1997; Ferenca 2006b; Kovács et al. 2008; Alekseev 2010a.

*ruficollis* **(DeGeer, 1774)**.Ogijewicz 1933; Pileckis 1960, 1976a; Silfverberg 1992, 2004; Pileckis and Monsevičius 1995; Monsevičius 1997; Alekseev 2010a.

*sibiricus* **Motschulsky, 1860** = *wehnckei* Gerhardt, 1877 = *sahlbergi* Falkenström, 1940 = *lineolatus* auct. nec Mannerheim, 1844. # 1.Pileckis 1976a; Pileckis and Monsevičius 1982, 1995; Silfverberg 1992, 2004; Monsevičius 1997; Alekseev 2010a.

*variegatus* **Sturm, 1834**. Ogijewicz 1933; Pileckis 1960, 1976a; Silfverberg 1992, 2004; Pileckis and Monsevičius 1995; Alekseev 2010a.

*varius* **Nicolai, 1822**.Ogijewicz 1933; Pileckis 1960, 1976a; Silfverberg 1992, 2004; Pileckis and Monsevičius 1995; Alekseev 2010a.

**NOTERIDAE Thomson, 1860**.

**Noterinae Thomson, 1860**.

**Noterini Thomson, 1860**.

*Noterus* **Clairville, 1806**.

*clavicornis* **(DeGeer, 1774)**. Jakobson 1905-1915; Ogijewicz 1933; Pileckis 1960, 1976a; Silfverberg 1992, 2004; Gaidienė 1993; Pileckis and Monsevičius 1995; Monsevičius 1997; Nilsson 2003a, 2009; Ferenca 2006b; Alekseev 2010a.

*crassicornis* **(O.F. Müller, 1776)**. Eichwald 1830; Ogijewicz 1933; Zajančkauskas and Pileckis 1968; Pileckis 1960, 1976a; Silfverberg 1992, 2004; Gaidienė 1993; Pileckis and Monsevičius 1995; Monsevičius 1997; Nilsson 2003a, 2009; Ferenca 2006b; Alekseev 2010a.

**DYTISCIDAE Leach, 1815**.

**Agabinae Thomson, 1867**.

*Platambus* **Thomson, 1859**.

*maculatus* **(Linnaeus, 1758)**. Ogijewicz 1933; Pileckis 1960, 1976a; Silfverberg 1992, 2004; Gaidienė 1993; Pileckis and Monsevičius 1995; Šablevičius 2000b, 2011; Nilsson 2003b, 2009; Ferenca 2006b; Alekseev 2010a.

*Agabus* **Leach, 1817**.

*affinis* **(Paykull, 1798)**. Jakobson 1905-1915; Pileckis 1960, 1976a; Silfverberg 1992, 2004; Pileckis and Monsevičius 1995; Monsevičius 1997; Nilsson 2003b, 2009; Ferenca 2006b; Ivinskis et al. 2008; Alekseev 2010a.

[*biguttulus **(Thomson, 1867)**]. # 24. Gaidienė 1993; Silfverberg 2004; Alekseev 2010a.

*bipustulatus* **(Linnaeus, 1767)** = *solieri* Aubé, 1837.Ogijewicz 1933; Pileckis 1960, 1976a; Zajančkauskas and Pileckis 1968; Gaidienė 1993; Silfverberg 1992, 2004; Pileckis and Monsevičius 1995; Monsevičius 1997; Nilsson 2003b, 2009; Ferenca 2006b; Ivinskis et al. 2008; Alekseev 2010a.

[*clypealis* **(Thomson, 1867)**]. Known in Latvia (Barševskis et al. 2005), Kaliningrad region (Alekseev 2010a), southern Sweden (Lundberg and Gustafsson 1995), Poland (Burakowski et al. 1976), Belarus (Alexandrovitch et al. 1996).

[*confinis* **(Gyllenhal, 1808)**]. Known in Latvia (Barševskis et al. 2005).

*congener* **(Thunberg, 1794)**. Ogijewicz 1933; Pileckis 1960, 1970a, 1976a, b, 1979; Bercio and Folwaczny 1979; Silfverberg 1992, 2004; Gaidienė 1993; Pileckis and Monsevičius 1995; Monsevičius 1997; Nilsson 2003b, 2009; Ferenca 2006b; Alekseev 2010a.

[*conspersus* **(Marsham, 1802)**].Known in northern Poland (Burakowski et al. 1976), southern Sweden (Lundberg and Gustafsson 1995), Denmark (Nilsson 2003b, 2009).

*didymus* **(Olivier, 1795)**. Gaidienė 1993; Silfverberg 2004; Alekseev 2010a.

*fuscipennis* **(Paykull, 1798)**. Jakobson 1905-1915; Pileckis and Monsevičius 1982, 1995; Silfverberg 1992, 2004; Monsevičius 1997; Nilsson 2003b, 2009; Alekseev 2010a.

*guttatus* **(Paykull, 1798)**. Karalius and Monsevičius 1992; Pileckis and Monsevičius 1995; Silfverberg 1996, 2004; Alekseev 2010a.

*labiatus* **(Brahm, 1791)**. Monsevičius 1986a, 1997; Silfverberg 1992, 2004; Pileckis and Monsevičius 1995; Nilsson 2003b, 2009; Alekseev 2010a.

*melanarius* **Aubé, 1837** = *tarsatus* Zetterstedt, 1838. Silfverberg 1992, 2004; Pileckis and Monsevičius 1995; Nilsson 2003b, 2009; Alekseev 2010a.

*nebulosus* **(Forster, 1771)**. Silfverberg 1992, 2004; Pileckis and Monsevičius 1995; Monsevičius 1997; Nilsson 2003b, 2009; Alekseev 2010a.

*paludosus* **(Fabricius, 1801)**. Lindeman 1871; Jakobson 1905-1915; Pileckis 1968b, 1976a; Bercio and Folwaczny 1979; Silfverberg 1992, 2004; Pileckis and Monsevičius 1995; Monsevičius 1997; Nilsson 2003b, 2009; Alekseev 2010a.

[*pseudoclypealis* **Scholtz, 1933**]. Known in Latvia (Barševskis et al. 2005), Belarus (Ryndevich 2004).

[*setulosus* **(J.R. Sahlberg, 1895)**]. Known in Latvia (Barševskis et al. 2005).

*striolatus* **(Gyllenhal, 1808)**. Miländer et al. 1984; Silfverberg 1992, 2004; Pileckis and Monsevičius 1995; Monsevičius 1997; Nilsson 2003b, 2009; Alekseev 2010a.

*sturmii* **(Gyllenhal, 1808)**. Jakobson 1905-1915; Ogijewicz 1933; Pileckis 1960, 1976a; Silfverberg 1992, 2004; Gaidienė 1993; Pileckis and Monsevičius 1995; Monsevičius 1997; Nilsson 2003b, 2009; Alekseev 2010a.

*uliginosus* **(Linnaeus, 1761)**. Eichwald 1830; Lindeman 1871, Pileckis 1968b, 1976a; Silfverberg 1992, 2004; Gaidienė 1993; Pileckis and Monsevičius 1995; Monsevičius 1997; Nilsson 2003b, 2009; Alekseev 2010a.

*undulatus* **(Schrank, 1776)**. Ogijewicz 1933; Pileckis 1960, 1976a; Silfverberg 1992, 2004; Gaidienė 1993; Pileckis and Monsevičius 1995; Nilsson 2003b, 2009; Ferenca 2006b; Alekseev 2010a.

*unguicularis* **(Thomson, 1867)**. Tamutis et al. 2008; Ivinskis et al. 2008, 2009; Alekseev 2010a.

*Ilybius* **Erichson, 1832**.

*aenescens* **Thomson, 1870**. Jakobson 1905-1915; Pileckis 1963b, 1976a; Silfverberg 1992, 2004; Gaidienė 1993; Pileckis and Monsevičius 1995; Monsevičius 1997; Žiogas and Zolubas 2005; Ferenca 2006b; Nilsson 2009; Alekseev 2010a.

*angustior* **(Gyllenhal, 1808)**. Barševskis 2001a; Silfverberg 2004; Alekseev 2010a.

*ater* **(DeGeer, 1774)**. Ogijewicz 1933; Pileckis 1960, 1976a; Silfverberg 1992, 2004; Gaidienė 1993; Pileckis and Monsevičius 1995; Monsevičius 1997; Gliaudys 2001; Nilsson 2003b, 2009; Ferenca 2006b; Alekseev 2010a.

*chalconatus* **(Panzer, 1796)**.# 13. Miländer et al. 1984 (*Agabus*); Gaidienė 1993 (*Gaurodytes*); Pileckis and Monsevičius 1982, 1995 (*Agabus*); Silfverberg 1992, 2004; Monsevičius 1997; Nilsson 2003b, 2009; Alekseev 2010a.

*crassus* **Thomson, 1856**.Monsevičius 1986a, 1997; Silfverberg 1992, 2004; Pileckis and Monsevičius 1995; Nilsson 2003b; Alekseev 2010a.

*erichsoni* **(Gemminger & Harold, 1868) =** *nigroaeneus* (Erichson, 1837) nec (Marsham, 1802). Monsevičius 1986a (*Agabus*); Silfverberg 1992, 2004; Pileckis and Monsevičius 1995 (*Agabus*); Nilsson 2003b, 2009; Ivinskis et al. 2009; Alekseev 2010a.

*fenestratus* **(Fabricius, 1781)**. Ogijewicz 1933; Pileckis 1960, 1976a; Bercio and Folwaczny 1979; Silfverberg 1992, 2004; Gaidienė 1993; Pileckis and Monsevičius 1995; Nilsson 2003b, 2009; Ferenca 2006b; Alekseev 2010a.

*fuliginosus* **(Fabricius, 1792)**. Ogijewicz 1933; Pileckis 1960, 1976a; Silfverberg 1992, 2004; Gaidienė 1993; Pileckis and Monsevičius 1995; Monsevičius 1997; Šablevičius 2000b; Gliaudys 2001; Nilsson 2003b, 2009; Ferenca 2006b; Vaivilavičius 2008; Alekseev 2010a.

*guttiger* **(Gyllenhal, 1808)**. Jakobson 1905-1915; Ogijewicz 1933; Pileckis 1960, 1976a; Silfverberg 1992, 2004; Gaidienė 1993; Pileckis and Monsevičius 1995; Monsevičius 1997; Gliaudys 2001; Nilsson 2003b, 2009; Ivinskis et al. 2008, 2009; Alekseev 2010a.

*montanus* **(Stephens, 1828)** = *melanocornis* (A.Zimmermann, 1915). # 13. Silfverberg 1992, 2004; Pileckis and Monsevičius 1995; Nilsson 2003b, 2009; Alekseev 2010a.

*neglectus* **(Erichson, 1837)**. Monsevičius 1986a, 1998 (*Agabus*); Silfverberg 1992, 2004; Pileckis and Monsevičius 1995 (*Agabus*); Nilsson 2003b, 2009; Ivinskis et al. 2009; Alekseev 2010a.

*quadriguttatus* **(Lacordaire, 1835)** = *obscurus* (Marsham, 1802). Ogijewicz 1933; Pileckis 1960, 1976a; Silfverberg 1992, 2004; Gaidienė 1993; Pileckis and Monsevičius 1995; Monsevičius 1997; Nilsson 2003b, 2009; Ferenca 2006b; Ivinskis et al. 2009; Alekseev 2010a.

*similis* **Thomson, 1856**. Gaidienė 1993; Monsevičius 1997; Barševskis 2001; Silfverberg 2004; Alekseev 2010a.

*subaeneus* **Erichson, 1837**. Ogijewicz 1933; Pileckis 1960, 1976a; Silfverberg 1992, 2004; Gaidienė 1993; Pileckis and Monsevičius 1995; Monsevičius 1997; Nilsson 2003b; Ferenca 2006b; Alekseev 2010a.

*subtilis* **(Erichson, 1837)**.Pileckis and Monsevičius 1982, 1995 (*Agabus*); Silfverberg 1992, 2004; Monsevičius 1997 (*Agabus*); Nilsson 2003b, 2009; Ivinskis et al. 2009; Alekseev 2010a.

[*wasastjernae **(C.R. Sahlberg, 1824)**]. # 25. Monsevičius 1998.

**Colymbetinae Erichson, 1837**.

**Colymbetini Erichson, 1837**.

*Rhantus* **Dejean, 1833**.

*bistriatus* **(Bergsträsser, 1778)** = *adspersus* (Fabricius, 1801).Pileckis 1960, 1976a; Zajančkauskas and Pileckis 1968; Silfverberg 1992, 2004; Pileckis and Monsevičius 1995; Monsevičius 1997; Nilsson 2003b, 2009; Ferenca 2006b; Alekseev 2010a.

*consputus* **(Sturm, 1834)**. Pileckis and Monsevičius 1995; Alekseev 2010a.

*exsoletus* **(Forster, 1771)**. Ogijewicz 1933; Pileckis 1960, 1976a; Silfverberg 1992, 2004; Pileckis and Monsevičius 1995; Monsevičius 1997; Šablevičius 2003; Nilsson 2003b, 2009; Ivinskis et al. 2008; Alekseev 2010a.

*frontalis* **(Marsham, 1802)** = *notatus* (Fabricius, 1781) nec (Bergsträsser, 1778). Lentz 1879; Ogijewicz 1933; Pileckis 1960, 1976a; Silfverberg 1992, 2004; Gaidienė 1993; Pileckis and Monsevičius 1995; Monsevičius 1997; Nilsson 2003b, 2009; Alekseev 2010a.

*grapii* **(Gyllenhal, 1808)**.Jakobson 1905-1915; Pileckis 1960, 1976a; Silfverberg 1992, 2004; Gaidienė 1993; Pileckis and Monsevičius 1995; Monsevičius 1997; Nilsson 2003b, 2009; Ferenca 2006b; Ivinskis et al. 2008; Alekseev 2010a.

*incognitus* **(R. Scholz, 1927)**. Karalius and Monsevičius 1992; Pileckis and Monsevičius 1995; Silfverberg 1996, 2004; Alekseev 2010a.

*latitans* **Sharp, 1882**. Barševskis 2001a; Silfverberg 2004; Alekseev 2010a.

*notaticollis* **(Aubé, 1837)**. Ogijewicz 1933; Pileckis 1960, 1976a; Silfverberg 1992, 2004; Pileckis and Monsevičius 1995; Nilsson 2003b, 2009; Alekseev 2010a.

*suturalis* **(MacLeay, 1825)** = *pulverosus* (Stephens, 1828).Silfverberg 1992, 2004; Gaidienė 1993; Pileckis and Monsevičius 1995; Nilsson 2003b, 2009; Alekseev 2010a.

*suturellus* **(Harris, 1828)**. Ogijewicz 1933; Pileckis 1960, 1976a; Silfverberg 1992, 2004; Gaidienė 1993; Pileckis and Monsevičius 1995; Monsevičius 1997; Šablevičius 2000b, 2003a; Nilsson 2003b, 2009; Alekseev 2010a.

*Colymbetes* **Clairville, 1806**.

*fuscus* **(Linnaeus, 1758)**. Eichwald 1830; Pileckis 1960, 1976a; Zajančkauskas and Pileckis 1968; Silfverberg 1992, 2004; Gaidienė 1993; Pileckis and Monsevičius 1995; Monsevičius 1997; Nilsson 2003b, 2009; Ferenca 2006b; Alekseev 2010a.

*paykulli* **Erichson, 1837**. Ogijewicz 1933; Pileckis 1960, 1976a; Zajančkauskas and Pileckis 1968; Silfverberg 1992, 2004; Gaidienė 1993; Pileckis and Monsevičius 1995; Monsevičius 1997; Šablevičius 2000b; Gliaudys 2001; Nilsson 2003b, 2009; Ferenca 2006b; Ivinskis et al. 2008; Alekseev 2010a.

*striatus* **(Linnaeus, 1758)**. Ogijewicz 1933; Pileckis 1960, 1976a; Zajančkauskas and Pileckis 1968; Silfverberg 1992, 2004; Gaidienė 1993; Pileckis and Monsevičius 1995; Monsevičius 1997; Nilsson 2003b, 2009; Ferenca 2006b; Ivinskis et al. 2008; Alekseev 2010a.

**Copelatinae Van den Branden, 1885**.

**Copelatini Van den Branden, 1885**.

*Copelatus* **Erichson, 1832**.

*haemorrhoidalis* **(Fabricius, 1787)** = *ruficollis* (Schaller, 1783) nec (DeGeer, 1774). Pileckis 1960, 1976a; Pileckis and Monsevičius 1995; Silfverberg 1992, 2004; Nilsson 2003b, 2009; Ferenca 2006b; Alekseev 2010a.

**Dytiscinae Leach, 1815**.

**Hydaticini Sharp, 1880**.

*Hydaticus* **Leach, 1817**.

[*aruspex* **Clark, 1864** = *laevipennis* Thomson, 1867]. Known in Latvia (Barševskis et al. 2005), Kaliningrad region (Alekseev 2010a), southern Sweden (Lundberg and Gustafsson 1995), northern Poland (Burakowski et al. 1976), Belarus (Alexandrovitch et al. 1996).

*continentalis* **Balfour-Browne, 1944** = *stagnalis* (Fabricius, 1787) nec (Geoffroy, 1785).Pileckis 1960, 1976a; Silfverberg 1992, 2004; Gaidienė 1993; Pileckis and Monsevičius 1995; Šablevičius 2003a; Nilsson 2003b, 2009; Ferenca 2006b; Alekseev 2010a.

*seminiger* **(DeGeer, 1774)**. Eichwald 1830; Ogijewicz 1933; Pileckis 1960, 1976a; Zajančkauskas and Pileckis 1968; Silfverberg 1992, 2004; Gaidienė 1993; Pileckis and Monsevičius 1995; Monsevičius 1997; Šablevičius 2000b; Nilsson 2003b, 2009; Ferenca 2006b; Ivinskis et al. 2008; Alekseev 2010a.

*transversalis* **(Pontoppidan, 1763)**. Ogijewicz 1933; Pileckis 1960, 1976a; Silfverberg 1992, 2004; Gaidienė 1993; Pileckis and Monsevičius 1995; Monsevičius 1997; Šablevičius 2000b; Nilsson 2003b, 2009; Ferenca 2006b; Ivinskis et al. 2008; Alekseev 2010a.

**Aciliini Thomson, 1867**.

*Graphoderus* **Dejean, 1833**.

*austriacus* **(Sturm, 1834)**.Karalius and Monsevičius 1992; Pileckis and Monsevičius 1995; Silfverberg 1996, 2004; Alekseev 2010a.

RDB*bilineatus* **(DeGeer, 1774)**. Jakobson 1905-1915; Ogijewicz 1933; Pileckis 1960, 1976a; Silfverberg 1992, 2004; Gaidienė 1993; Pileckis and Monsevičius 1995; Nilsson 2003b, 2009; Ferenca 2004, 2006a; Tamutis 2005b; Kriaučiūnienė and Zaplatkin 2007; Rašomavičius 2007; Uselis et al. 2007; Ivinskis et al. 2008, 2009; Švitra and Aliukonis 2009; Alekseev 2010a, b; Šablevičius 2011.

*cinereus* **(Linnaeus, 1758)**. Ogijewicz 1933; Pileckis 1960, 1976a; Bercio and Folwaczny 1979; Silfverberg 1992, 2004; Pileckis and Monsevičius 1995; Nilsson 2003b, 2009; Ferenca 2006b; Ivinskis et al. 2009; Alekseev 2010a.

*zonatus zonatus* **(Hoppe, 1795)**. Ogijewicz 1933; Pileckis 1960, 1976a; Silfverberg 1992, 2004; Pileckis and Monsevičius 1995; Monsevičius 1997; Gliaudys 2001; Nilsson 2003b, 2009; Ivinskis et al. 2009; Alekseev 2010a.

*Acilius* **Leach, 1817**.

*canaliculatus* **(Nicolai, 1822)**. Ogijewicz 1933; Pileckis 1960, 1976a; Lešinskas and Pileckis 1967; Zajančkauskas and Pileckis 1968; Silfverberg 1992, 2004; Gaidienė 1993; Pileckis and Monsevičius 1995; Monsevičius 1997; Ferenca 2006b; Vaivilavičius 2008; Ivinskis et al. 2008; Alekseev 2010a; Šablevičius 2011.

*sulcatus* **(Linnaeus, 1758)**. Eichwald 1830; Ogijewicz 1933; Pileckis 1960, 1976a; Zajančkauskas and Pileckis 1968; Silfverberg 1992, 2004; Gaidienė 1993; Pileckis and Monsevičius 1995; Monsevičius 1997; Šablevičius 2000b; Gliaudys 2001; Nilsson 2003b, 2009; Ferenca 2006b; Ivinskis et al. 2008; Alekseev 2010a.

**Dytiscini Leach, 1815**.

*Dytiscus* **Linnaeus, 1758**.

*circumcinctus* **Ahrens, 1811**. Ogijewicz 1933; Pileckis 1960, 1976a; Bercio and Folwaczny 1979; Silfverberg 1992, 2004; Gaidienė 1993; Pileckis and Monsevičius 1995; Monsevičius 1997; Nilsson 2003b, 2009; Ferenca 2006b; Ivinskis et al. 2008; Alekseev 2010a.

*circumflexus* **Fabricius, 1801**. Pileckis 1960, 1970a, b, 1976a; Silfverberg 1992, 2004; Gaidienė 1993; Pileckis and Monsevičius 1995; Nilsson 2003b, 2009; Ferenca 2006b; Alekseev 2010a.

*dimidiatus* **Bergsträsser, 1778**. Ogijewicz 1933; Pileckis 1960, 1970a, 1976a; Silfverberg 1992, 2004; Gaidienė 1993; Pileckis and Monsevičius 1995; Monsevičius 1997; Gliaudys 2001; Nilsson 2003b, 2009; Ferenca 2006b; Ivinskis et al. 2008; Alekseev 2010a.

*lapponicus* **Gyllenhal, 1808**. Gaidienė and Ferenca 1988; Silfverberg 1992, 2004; Gaidienė 1993; Pileckis and Monsevičius 1995; Šablevičius 2000a, 2003a, b, 2011; Nilsson 2003b, 2009; Ivinskis et al. 2009; Alekseev 2010a.

RDB*latissimus* **Linnaeus, 1758**. Eichwald 1830; Ogijewicz 1933; Pileckis 1960, 1976a; Lešinskas and Pileckis 1967; Zajančkauskas and Pileckis 1968; Silfverberg 1992, 2004; Gaidienė 1993; Pileckis and Monsevičius 1995; Monsevičius 1997; Šablevičius 2000b, 2011; Gliaudys 2001; Nilsson 2003b, 2009; Tamutis 2005b; Ferenca 2006b; Rašomavičius 2007; Lopeta 2007a; Ivinskis et al. 2008; Aliukonis and Švitra 2009; Švitra and Aliukonis 2009; Alekseev 2010a, b.

*marginalis* **Linnaeus, 1758** = *conformis* Kunze, 1818.Eichwald 1830; Ogijewicz 1933; Pileckis 1960, 1976a; Silfverberg 1992, 2004; Gaidienė 1993; Pileckis and Monsevičius 1995; Šablevičius 2000b, 2011; Gliaudys 2001; Nilsson 2003b, 2009; Ferenca 2006b; Ivinskis et al. 2008; Alekseev 2010a.

*semisulcatus* **O.F. Müller, 1776** = *punctulatus* Fabricius, 1776. Ogijewicz 1993; Pileckis 1960, 1970a; Silfverberg 1992, 2004; 1976a; Gaidienė 1993; Pileckis and Monsevičius 1995; Nilsson 2003b, 2009; Alekseev 2010a.

**Cybisterini Sharp, 1880**.

*Cybister* **Curtis, 1827**.

*lateramarginalis* **(DeGeer, 1774)**. Ogijewicz 1933; Pileckis 1960, 1970a, b, 1976a; Lešinskas and Pileckis 1967; Bercio and Folwaczny 1979; Silfverberg 1992, 2004; Gaidienė 1993; Pileckis and Monsevičius 1995; Gliaudys 2001; Ferenca 2006b; Ivinskis et al. 2008; Alekseev 2010a; Šablevičius 2011.

**Hydroporinae Aubé, 1836**.

**Laccornini Wolfe & Roughley, 1990**.

L*accornis* **Gozis, 1914**.

*oblongus* **(Stephens, 1835)**.# 26. Monsevičius 1997.

**Hydrovatini Sharp, 1880**.

*Hydrovatus* **Motschulsky, 1853**.

[*cuspidatus* **(Kunze, 1818)**]. Known in northern Poland (Burakowski et al. 1976), Denmark (Nilsson 2003b, 2009).

**Bidessini Sharp, 1880**.

*Bidessus* **Sharp, 1880**.

*grossepunctatus* **Vorbringer, 1907**.Barševskis 2001a; Silfverberg 2004; Alekseev 2010a.

*unistriatus* **(Goeze, 1777)**.Ogijewicz 1933; Pileckis 1960, 1976a; Silfverberg 1992, 2004; Pileckis and Monsevičius 1995; Monsevičius 1997; Nilsson 2003b, 2009; Alekseev 2010a.

*Hydroglyphus* **Motschulsky, 1853** = *Guignotus* Houlbert, 1934.

*geminus* **(Fabricius, 1792)** = *pusillus* (Fabricius, 1781) nec (O.F. Müller, 1776). Miländer et al. 1984; Silfverberg 1992, 2004; Gaidienė 1993; Pileckis and Monsevičius 1995 (*Bidessus*); Šablevičius 2003a. Nilsson 2003b, 2009; Alekseev 2010a.

*hamulatus* **(Gyllenhal, 1813)**. Pileckis and Monsevičius 1995 (*Bidessus*); Silfverberg 2004; Alekseev 2010a.

**Hyphydrini Gistel, 1848**.

*Hyphydrus* **Illiger, 1802**.

*ovatus* **(Linnaeus, 1761)**.Ogijewicz 1933; Pileckis 1960, 1976a; Ivinskis et al. 1984; Silfverberg 1992, 2004; Gaidienė 1993; Pileckis and Monsevičius 1995; Monsevičius 1997; Nilsson 2003b, 2009; Ferenca 2006b; Alekseev 2010a; Šablevičius 2011.

**Hygrotini Portevin, 1929**.

*Hygrotus* **Stephens, 1828**.

[*confluens* **(Fabricius, 1787)**].Known in Kaliningrad region (Alekseev 2010a), northeastern Poland (Burakowski et al. 1976), eastern Belarus (Alexandrovitch et al. 1996); southern Sweden (Lundberg and Gustafsson 1995), Denmark (Nilsson 2003b, 2009).

*decoratus* **(Gyllenhal, 1810)**.Jakobson 1905-1915; Monsevičius 1986a; Silfverberg 1992, 2004; Gaidienė 1993; Pileckis and Monsevičius 1995; Monsevičius 1997; Šablevičius 2000b, 2003a; Nilsson 2003b, 2009; Alekseev 2010a.

*impressopunctatus* **(Schaller, 1783)**.Ogijewicz 1933 (*Coelambus*); Pileckis 1960, 1976a (*Coelambus*); Silfverberg 1992, 2004 (*Coelambus*);Gaidienė 1993 (*Coelambus*); Pileckis and Monsevičius 1995 (*Coelambus*); Monsevičius 1997 (*Coelambus*); Nilsson 2003b, 2009; Alekseev 2010a.

*inaequalis* **(Fabricius, 1776)**.Ogijewicz 1933; Pileckis 1960, 1976a; Silfverberg 1992, 2004; Gaidienė 1993; Pileckis and Monsevičius 1995; Monsevičius 1997; Šablevičius 2000b; Nilsson 2003b, 2009; Ferenca 2006b; Alekseev 2010a.

[*marklini* **(Gyllenhal, 1813)**].Known in Latvia (Barševskis et al. 2005); Kaliningrad region (Alekseev 2010a), northwestern Belarus (Alexandrovitch et al. 1996), northern Poland (Burakowski et al. 1976).

[*nigrolineatus* **(Steven, 1808) =** *lautus* (Schaum, 1843)].Known in southern Sweden (Lundberg and Gustafsson 1995), Denmark (Nilsson 2003b, 2009), Belarus (Ryndevich 2004).

*novemlineatus* **(Stephens, 1829)**. Tamutis et al. 2008 (*Coelambus*); Alekseev 2010a.

*parallelogrammus* **(Ahrens, 1812)**. Karalius and Monsevičius 1992 (*Coelambus*); Pileckis and Monsevičius 1995 (*Coelambus*); Silfverberg 1996, 2004 (*Coelambus*); Alekseev 2010a.

*polonicus* **(Aubé, 1842)**. Silfverberg 1992, 2004 (*Coelambus*); Gaidienė 1993 (*Coelambus*); Pileckis and Monsevičius 1995 (*Coelambus*); Ivinskis et al. 2009; Alekseev 2010a.

[*quinquelineatus* **(Zetterstedt, 1828)**]. Known in northern Belarus (Alexandrovitch et al. 1996), southern Sweden (Lundberg and Gustafsson 1995), Denmark (Nilsson 2003b, 2009).

*versicolor* **(Schaller, 1783)**. Pileckis 1976a; Silfverberg 1992, 2004; Pileckis and Monsevičius 1995; Monsevičius 1997; Gliaudys 2001; Nilsson 2003b, 2009; Vaivilavičius 2008; Alekseev 2010a.

**Hydroporini Aubé, 1836**.

*Hydroporus* **Clairville, 1806 =** *Hydrocolus* Roughley & Nilsson, 2000.

*angustatus* **Sturm, 1835**. Bercio and Folwaczny 1979; Silfverberg 1992, 2004; Pileckis and Monsevičius 1995; Monsevičius 1997; Nilsson 2003b, 2009; Alekseev 2010a.

[*brevis* **R.F. Sahlberg, 1834**]. Known in Kaliningrad region (Alekseev 2010a), northeastern Poland (Burakowski et al. 1976), northwestern Belarus (Alexandrovitch et al. 1996), southern Sweden (Lundberg and Gustafsson 1995), Latvia (Barševskis et al. 2005).

*elongatulus* **Sturm, 1835** = *eugeniae* Zaitzev, 1909.Pileckis 1960, 1976a; Bercio and Folwaczny 1979; Silfverberg 1992, 2004; Pileckis and Monsevičius 1995; Monsevičius 1997; Šablevičius 2000b; Nilsson 2003b, 2009; Ferenca 2006b; Alekseev 2010a.

*erythrocephalus* **(Linnaeus, 1758)**. Lentz 1879; Ogijewicz 1933; Pileckis 1960, 1976a; Silfverberg 1992, 2004; Gaidienė 1993; Pileckis and Monsevičius 1995; Monsevičius 1997; Nilsson 2003b, 2009; Alekseev 2010a.

*fuscipennis* **Schaum, 1868**. Pileckis 1960, 1970a, 1976a, b, 1979; Silfverberg 1992, 2004; Gaidienė 1993; Pileckis and Monsevičius 1995; Monsevičius 1997; Nilsson 2003b, 2009; Ferenca 2006b Alekseev 2010a.

*glabriusculus* **Aubé, 1838**.Pileckis and Monsevičius 1982, 1995; Silfverberg 1992, 2004; Monsevičius 1997; Nilsson 2003b, 2009; Alekseev 2010a.

[*gyllenhalii* **Schiödte, 1841** = *piceus* auct. nec Stephens, 1828].Known in northeastern Poland (Burakowski et al. 1976), southern Sweden (Lundberg and Gustafsson 1995), Belarus, Denmark (Nilsson 2003b, 2009).

*incognitus* **Sharp, 1869**. Karalius and Monsevičius 1992; Pileckis and Monsevičius 1995; Silfverberg 1996, 2004; Monsevičius 1997; Alekseev 2010a.

[*kraatzii* **Schaum, 1867**]. Known in Poland (Burakowski et al. 1976), Estonia (Nilsson 2003b, 2009).

*longicornis* **Sharp, 1871**. Miländer et al. 1984; Silfverberg 1992, 2004; Pileckis and Monsevičius 1995; Monsevičius 1997; Nilsson 2003b, 2009; Alekseev 2010a.

*melanarius* **Sturm, 1835**. Lentz 1879; Pileckis 1960, 1976a; Bercio and Folwaczny 1979; Silfverberg 1992, 2004; Pileckis and Monsevičius 1995; Monsevičius 1997; Nilsson 2003b, 2009; Alekseev 2010a.

*memnonius* **Nicolai, 1822**. Pileckis 1960, 1976a; Silfverberg 1992, 2004; Pileckis and Monsevičius 1995; Monsevičius 1997; Ferenca 2006b; Nilsson 2003b, 2009; Alekseev 2010a.

*morio* **Aubé, 1838** = *valliger* Hellén, 1929 = *melanocephalus* auct. nec (Marsham, 1802).Ogijewicz 1933; Pileckis 1960, 1976a; Silfverberg 1992, 2004; Pileckis and Monsevičius 1995; Monsevičius 1997; Nilsson 2003b, 2009; Alekseev 2010a.

*neglectus* **Schaum, 1845**. Monsevičius 1986a, 1997; Silfverberg 1992, 2004; Pileckis and Monsevičius 1995; Nilsson 2003b, 2009; Alekseev 2010a.

[*neuter* **Fairmaire & Laboulbène, 1854** = *discretus* Fairmaire, 1859].Known in Kaliningrad region (Alekseev 2010a), northern Poland (Burakowski et al. 1976), Latvia (Barševskis et al. 2005), southern Sweden (Lundberg and Gustafsson 1995), northwestern Belarus (Alexandrovitch et al. 1996).

[*nigellus* **Mannerheim, 1853** = *tartaricus* LeConte, 1850].Known in Latvia (Barševskis et al. 2005), southern Sweden (Lundberg and Gustafsson 1995).

*nigrita* **(Fabricius, 1792)**. Jakobson 1905-1915; Ogijewicz 1933; Pileckis 1960, 1976a; Silfverberg 1992, 2004; Pileckis and Monsevičius 1995; Nilsson 2003b, 2009; Alekseev 2010a.

*notatus* **Sturm, 1835**.Monsevičius 1986a, 1997; Silfverberg 1992, 2004; Pileckis and Monsevičius 1995; Ferenca et al. 2002; Alekseev 2010a.

*obscurus* **Sturm, 1835**.Pileckis and Monsevičius 1982, 1995; Silfverberg 1992, 2004; Monsevičius 1997; Nilsson 2003b, 2009; Alekseev 2010a.

[*obsoletus* **Aubé, 1838**]. Known in southern Sweden (Lundberg and Gustafsson 1995), Denmark (Nilsson 2003b, 2009).

*palustris* **(Linnaeus, 1761)**. Ogijewicz 1933; Pileckis 1960, 1976a; Silfverberg 1992, 2004; Pileckis and Monsevičius 1995; Monsevičius 1997; Šablevičius 2000b; Ferenca 2006b; Nilsson 2003b, 2009; Alekseev 2010a.

*planus* **(Fabricius, 1781)**. Silfverberg 1992, 2004; Gaidienė 1993; Šablevičius and Ferenca 1995; Pileckis and Monsevičius 1995; Monsevičius 1997; Vaivilavičius 2008; Nilsson 2003b, 2009; Ivinskis et al. 2009; Alekseev 2010a.

*pubescens* **(Gyllenhal, 1808)**. Tamutis 2003; Alekseev 2010a.

*rufifrons* **(O.F. Müller, 1776)** = *piceus* Stephens, 1828.# 27. Ogijewicz 1933; Pileckis 1960, 1976a; Bercio and Folwaczny 1979; Silfverberg 1992, 2004; Pileckis and Monsevičius 1995; Nilsson 2003b, 2009; Alekseev 2010a.

*scalesianus* **Stephens, 1828**. Ferenca and Tamutis 2009; Alekseev 2010a.

*striola* **(Gyllenhal, 1826)**. Jakobson 1905-1915; Ogijewicz 1933; Pileckis 1960, 1976a; Silfverberg 1992, 2004; Pileckis and Monsevičius 1995; Monsevičius 1997; Nilsson 2003b, 2009; Ivinskis et al. 2008; Alekseev 2010a.

[*tessellatus* **(Drapiez, 1819)**]. Known in Latvia (Barševskis et al. 2005).

*tristis* **(Paykull, 1798)**. Ogijewicz 1933; Pileckis 1960, 1970a, 1976a, b, 1979; Silfverberg 1992, 2004; Pileckis and Monsevičius 1995; Monsevičius 1997; Nilsson 2003b, 2009; Alekseev 2010a.

*umbrosus* **(Gyllenhal, 1808)**. Jakobson 1905-1915; Ogijewicz 1933; Pileckis 1960, 1976a; Silfverberg 1992, 2004; Pileckis and Monsevičius 1995; Monsevičius 1997; Nilsson 2003b, 2009; Alekseev 2010a.

*Porhydrus* **Guignot, 1945**.

*lineatus* **(Fabricius, 1775)**. Ogijewicz 1933; Pileckis 1960, 1976a; Silfverberg 1992, 2004; Gaidienė 1993; Pileckis and Monsevičius 1995; Monsevičius 1997; Nilsson 2003b, 2009; Alekseev 2010a.

[*obliquesignatus* **(Bielz, 1852)**]. Known in northwestern Belarus (Alexandrovitch et al. 1996).

*Graptodytes* **Seidlitz, 1887**.

*bilineatus* **(Sturm, 1835)**. Karalius and Monsevičius 1992; Pileckis and Monsevičius 1995; Silfverberg 1996, 2004; Šablevičius 2000b; Alekseev 2010a.

*granularis* **(Linnaeus, 1767)**. Jakobson 1905-1915; Pileckis 1960, 1976a; Silfverberg 1992, 2004; Pileckis and Monsevičius 1995; Monsevičius 1997; Nilsson 2003b, 2009; Ferenca 2006b; Alekseev 2010a.

*pictus* **(Fabricius, 1787)**. Pileckis 1960, 1976a; Silfverberg 1992, 2004; Gaidienė 1993; Pileckis and Monsevičius 1995; Monsevičius 1997; Šablevičius 2003a; Nilsson 2003b, 2009; Ferenca 2006b; Alekseev 2010a.

*Oreodytes* **Seidlitz, 1887**.

*sanmarkii* **(C.R. Sahlberg, 1826)** = *rivalis* (Gyllenhal, 1827). # 28. Pileckis 1976a; Silfverberg 1992, 2004; Pileckis and Monsevičius 1995; Nilsson 2003b, 2009; Alekseev 2010a.

*Suphrodytes* **DesGozis, 1914**.

*dorsalis* **(Fabricius, 1787)** = *eljasi* (Wirén, 1968).Ogijewicz 1933 (*Hydroporus*); Pileckis 1960, 1976a (*Hydroporus*); Silfverberg 1992, 2004; Pileckis and Monsevičius 1995 (*Hydroporus*); Monsevičius 1997 (*Hydroporus*); Šablevičius 2000b, 2003b (*Hydroporus*); Nilsson 2003b, 2009; Ferenca 2006b; Alekseev 2010a.

*Deronectes* **Sharp, 1880**.

*latus* **(Stephens, 1829)**. Ogijewicz 1933; Pileckis 1960, 1976a; Silfverberg 1992, 2004; Pileckis and Monsevičius 1995; Nilsson 2003b, 2009; Alekseev 2010a.

*Scarodytes* **DesGozis, 1914**.

*halensis* **(Fabricius, 1787)**. Pileckis and Monsevičius 1982, 1995; Silfverberg 1992, 2004; Gaidienė 1993; Monsevičius 1997; Šablevičius 2000b, 2011; Nilsson 2003b, 2009; Alekseev 2010a.

*Nebrioporus* **Régimbart, 1906** = *Potomonectes* A.Zimmermann, 1921.

*assimilis* **(Paykull, 1798)**. Pileckis and Monsevičius 1995; Silfverberg 2004; Alekseev 2010a.

*canaliculatus* **(Lacordaire, 1835)**.Nilsson 2003b.

*depressus* **(Fabricius, 1775)** = *elegans* (Panzer, 1794). Ogijewicz 1933; Pileckis 1960, 1976a; Bercio and Folwaczny 1979; Silfverberg 1992, 2004; Pileckis and Monsevičius 1995; Nilsson 2003b, 2009; Alekseev 2010a.

*Stictotarsus* **A. Zimmermann, 1919**.

*duodecimpustulatus* **(Fabricius, 1792)**. # 29. Pileckis 1960 (*Deronectes*); Ferenca 2006b.

[*griseostriatus* **(DeGeer, 1774)**]. Known in souhern Sweden, Estonia (Lundberg and Gustafsson 1995).

**Laccophilinae Gistel, 1848**.

**Laccophilini Gistel, 1848**.

**L***accophilus* **Leach, 1815**.

*hyalinus* **(DeGeer, 1774)**.Ogijewicz 1933; Pileckis 1960, 1976a; Zajančkauskas and Pileckis 1968; Bercio and Folwaczny 1979; Silfverberg 1992, 2004; Gaidienė 1993; Pileckis and Monsevičius 1995; Monsevičius 1997; Nilsson 2003b, 2009; Ferenca 2006b; Alekseev 2010a.

*minutus* **(Linnaeus, 1758)** = *obscurus* (Panzer, 1794). Ogijewicz 1933; Pileckis 1960, 1976a; Silfverberg 1992, 2004; Pileckis and Monsevičius 1995; Monsevičius 1997; Nilsson 2003b, 2009; Ferenca 2006b; Alekseev 2010a.

*poecilus* **Klug, 1834** = *ponticus* Sharp, 1882 = *variegatus* (Germar, 1812) nec (Geoffroy, 1785) = *obsoletus* auct. nec Westhoff, 1881. Ogijewicz 1933; Pileckis 1960, 1976a; Silfverberg 1992, 2004; Pileckis and Monsevičius 1995; Monsevičius 1997; Gliaudys 2001; Nilsson 2003b, 2009; Ferenca 2006b; Alekseev 2010a.

**POLYPHAGA Emery, 1886**.

**HYDROPHILOIDEA Latreille, 1802**.

**HYDROPHILIDAE Latreille, 1802**.

**Helophorinae Leach, 1815**.

*Helophorus* **Fabricius, 1775**.(Hydraenidae)

[*aequalis* **Thomson, 1868**]. Known in Poland, Denmark (Hansen 2009), southern Sweden (Lundberg and Gustafsson 1995), Latvia (Telnov 2004).

*aquaticus* **(Linnaeus, 1758)**. Eichwald 1830; Roubal 1910; Ogijewicz 1933; Pileckis 1960, 1963b, 1976a; Silfverberg 1992, 2004; Gaidienė 1993; Pileckis and Monsevičius 1995; Monsevičius 1997; Šablevičius 2000b; Löbl and Smetana 2004; Ferenca 2006b; Vaivilavičius 2008; Hansen 2004, 2009; Alekseev 2010a.

*arvenicus* **Mulsant, 1846**. Mazurowa and Mazur 1939; Pileckis 1960, 1976a; Silfverberg 1992, 2004; Pileckis and Monsevičius 1995; Hansen 2004a, 2009; Vorst et al. 2007; Alekseev 2010a.

[*asperatus* **Rey, 1885**]. Known in southern Sweden (Lundberg and Gustafsson 1995), Poland (Burakowski et al. 1976), Denmark (Hansen 2009).

*brevipalpis* **Bedel, 1881**. Ogijewicz 1933; Pileckis 1960, 1976a; Silfverberg 1992, 2004; Gaidienė 1993; Pileckis and Monsevičius 1995; Hansen 2004a, 2009; Ferenca 2006b; Alekseev 2010a.

[*croaticus* **Kuwert, 1886**]. Known in Belarus (Ryndevich 2004), Poland (Burakowski et al. 1976), recently discovered in Latvia (Telnov et al. 2006).

*flavipes* **Fabricius, 1792** *= viridicollis* Stephens, 1828. Ogijewicz 1933; Pileckis 1960, 1976a; Bercio and Folwaczny 1979; Silfverberg 1992, 2004; Gaidienė 1993; Pileckis and Monsevičius 1995; Monsevičius 1997; Hansen 2004a, 2009; Ferenca 2006b; Alekseev 2010a.

[*fulgidicollis* **Motschulsky, 1860**]. Known in southern Sweden (Lundberg and Gustafsson 1995), Denmark (Hansen 2009).

*grandis* **Illiger, 1798**. Karalius and Monsevičius 1992; Pileckis and Monsevičius 1995; Silfverberg 1996, 2004; Hansen 2004a; Hansen 2009; Alekseev 2010a.

*granularis* **(Linnaeus, 1761)**. Ogijewicz 1933; Mazurowa and Mazur 1939; Pileckis 1960, 1968b, 1976a; Silfverberg 1992, 2004; Gaidienė 1993; Pileckis and Monsevičius 1995; Monsevičius 1997; Šablevičius 2000b; Hansen 2004a, 2009; Ferenca 2006b; Vaivilavičius 2008; Alekseev 2010a.

*griseus* **Herbst, 1793** *= affinis* Marsham, 1802. Pileckis 1968b, 1976a; Silfverberg 1992, 2004; Gaidienė 1993; Šablevičius and Ferenca 1995; Pileckis and Monsevičius 1995; Monsevičius 1997; Hansen 2004a, 2009; Ferenca 2006b; Vaivilavičius 2008; Ivinskis et al. 2009; Alekseev 2010a.

[*lapponicus* **Thomson, 1853**]. Known in northwestern Belarus (Alexandrovitch et al. 1996; Ryndevich 2004), eastern Prusia (Schilsky 1909), Sweden (Lundberg and Gustafsson 1995), Estonia, Denmark (Hansen 2009).

[*laticollis* **Thomson, 1853**]. Known in Kaliningrad region (Alekseev 2010a), Belarus (Ryndevich 2004), northwestern Poland (Burakowski et al. 1976), Sweden (Lundberg and Gustafsson 1995), Estonia, Denmark (Hansen 2009).

*longitarsis* **Wollaston, 1864**. Monsevičius 1997.

*minutus* **Fabricius, 1775**. Pileckis 1960, 1976a; Silfverberg 1992, 2004; Pileckis and Monsevičius 1995; Hansen 2004a, 2009; Alekseev 2010a.

*nanus* **Sturm, 1836**. Pileckis 1976a; Bercio and Folwaczny 1979; Silfverberg 1992, 2004; Pileckis and Monsevičius 1995; Monsevičius 1997; Hansen 2004a, 2009; Alekseev 2010a.

*nubilus* **Fabricius, 1777**. Eichwald 1830; Lindeman 1871; Pileckis 1968b; Pileckis 1976a; Silfverberg 1992, 2004; Gaidienė 1993; Pileckis and Monsevičius 1995; Hansen 2004a, 2009; Alekseev 2010a.

[*obscurus* **Mulsant, 1844 =** *walkeri* Sharp, 1916]. Known in southern Sweden (Lundberg and Gustafsson 1995), northern Poland (Burakowski et al. 1976), Denmark (Hansen 2009).

[*paraminutus* **Angus, 1986**].Known in northwestern Belarus (Alexandrovitch et al. 1996; Ryndevich 2004).

[*pumilio **Erichson, 1837**]. # 30. Lindeman 1871; Pileckis 1968b (*Helophorus pumilio* Er. = *Helophorus redtenbacheri* Kuv., 1976a (*Helophorus pumilio* Er. = *Helophorus redtenbacheri* Kunc.); Alekseev 2010a.

[*redtenbacheri **Kuwert, 1885**]. # 30. Silfverberg 1992, 2004; Pileckis and Monsevičius 1995 (*Helophorus redtenbacheri* Kuwert = *Helophorus pumilio* auct. nec Er.); Hansen 2004a, 2009; Alekseev 2010a.

*strigifrons* **Thomson, 1868**. Silfverberg 1992, 2004; Pileckis and Monsevičius 1995; Monsevičius 1997; Alekseev 2010a.

*tuberculatus* **Gyllenhal, 1810**. Bercio and Folwaczny 1979; Monsevičius 1986a; Silfverberg 1992, 2004; Pileckis and Monsevičius 1995; Monsevičius 1997; Ferenca 2004; Hansen 2004a, 2009; Alekseev 2010a.

**Georissinae Laporte, 1840**.

*Georissus* **Latreille, 1809**.(Georissidae)

*crenulatus* **(P. Rossi, 1794)**. Pileckis 1976a; Bercio and Folwaczny 1979; Silfverberg 1992, 2004; Pileckis and Monsevičius 1995; Šablevičius 2000a, 2003a; Hansen 2004b, 2009.

**Hydrochinae Thomson, 1859**.

*Hydrochus* **Leach, 1817**.(Hydraenidae)

[*angustatus* **Germar, 1824**]. Known in Latvia (Telnov 2004), central Poland (Burakowski et al. 1976), Estonia (Silfverberg 1992, 2004).

*brevis* **(Herbst, 1793)**. Lindeman 1871; Pileckis 1968b, 1976a; Silfverberg 1992, 2004; Pileckis and Monsevičius 1995; Monsevičius 1997; Barševskis 2001a; Hansen 2004c; Vaivilavičius 2008; Alekseev 2010a.

*crenatus* **(Fabricius, 1792) =** *carinatus* Germar, 1824. Karalius and Monsevičius 1992; Pileckis and Monsevičius 1995; Silfverberg 1996, 2004; Barševskis 2001a; Ferenca et al. 2006; Alekseev 2010a.

*elongatus* **(Schaller, 1783)**. # 31. Ogijewicz 1933; Pileckis 1960, 1976a; Gaidienė 1993; Pileckis and Monsevičius 1995; Monsevičius 1997; Šablevičius 2000b; Ivinskis et al. 2008.

*ignicollis* **Motschulsky, 1860**. # 31. Silfverberg 1992, 2004; Hansen 2004c, 2009; Ferenca 2006b; Alekseev 2010a.

[*kirgisicus* **Motschulsky, 1860**]. Known in central Russia (Shatrovskij 1993), northwestern Belarus (Alexandrovitch et al. 1996; Ryndevich 2004).

[*megaphallus* **van Berge Henegouwen, 1988**]. Recently found in Latvia (Telnov et al. 2006), known in Belarus (Ryndevich 2004), Sweden (Lundberg and Gustafsson 1995), Poland, Denmark (Hansen 2009).

**Spercheinae Erichson, 1837**.

*Spercheus* **Illiger, 1798**.(Sphercheidae)

*emarginatus* **(Schaller, 1783)**. Pileckis 1976a; Silfverberg 1992, 2004; Pileckis and Monsevičius 1995; Šablevičius 2000b, 2003a; Hansen 2004d, 2009; Alekseev 2010a.

**Hydrophilinae Latreille, 1802**.

**Berosini Mulsant, 1844**

*Berosus* **Leach, 1817**.

[*fulvus* **Kuwert, 1888**]. Known in southern Sweden (Lundberg and Gustafsson 1995), Denmark (Hansen 2009).

*luridus* **(Linnaeus, 1761)**. Ogijewicz 1933; Pileckis 1960, 1976a; Silfverberg 1992, 2004; Pileckis and Monsevičius 1995; Monsevičius 1997; Hansen 2004e, 2009; Ferenca 2006b; Alekseev 2010a.

*signaticollis* **(Charpentier, 1825)**. Monsevičius 1986a, 1997; Silfverberg 1992, 2004; Pileckis and Monsevičius 1995; Hansen 2004e, 2009; Alekseev 2010a.

[*spinosus* **(Steven, 1808)**]. Known in southern Sweden (Lundberg and Gustafsson 1995), Latvia, Estonia (Hansen 2009), central Poland (Burakowski et al. 1976), Belarus (Ryndevich 2004).

**Chaetarthriini Bedel, 1881**

*Chaetarthria* **Stephens, 1832**.

*seminulum* **(Herbst, 1797)**. Pileckis 1960, 1976a; Silfverberg 1992, 2004; Pileckis and Monsevičius 1995; Monsevičius 1997; Hansen 2004e, 2009; Ferenca 2006b; Ivinskis et al. 2008; Šablevičius 2011.

**Anacaenini Hansen, 1991**

*Paracymus* **Thomson, 1867**.

[*aeneus* **(Germar, 1824)**]. Known in southern Sweden (Lundberg and Gustafsson 1995), Denmark (Hansen 2009).

*Anacaena* **Thomson, 1859**.

*globulus* **(Paykull, 1798)**.Ivinskis et al. 2008, 2009; Alekseev 2010a.

*lutescens* **(Stephens, 1832) =** *limbata* auct. nec (Fabricius, 1792). # 32. Ogijewicz 1933; Mazurowa and Mazur 1939; Pileckis 1960, 1976a; Silfverberg 1992, 2004; Gaidienė 1993; Pileckis and Monsevičius 1995; Monsevičius 1997; Šablevičius 2000b; Tamutis and Zolubas 2001; Hansen 2004e, 2009; Vaivilavičius 2008; Alekseev 2010a.

*limbata* **(Fabricius, 1792)**.# 32. Ferenca and Tamutis 2009; Alekseev 2010a.

**Laccobiini Houlbert, 1922**

*Laccobius* **Erichson, 1837**.

[*albipes* **Kuwert, 1890**]. Known in northern Poland (Burakowski et al. 1976), recently found in Latvia (Vorst et al. 2007).

*bipunctatus* **(Fabricius, 1775)**. Ogijewicz 1933; Pileckis 1960, 1976a; Silfverberg 1992, 2004; Gaidienė 1993; Pileckis and Monsevičius 1995; Monsevičius 1997; Hansen 2004e, 2009; Ferenca 2006b; Alekseev 2010a.

[*colon* **(Stephens, 1829) =** *biguttatus* Gerhardt, 1877]. Known in Kaliningrad region (Alekseev 2010a), southern Sweden (Lundberg and Gustafsson 1995), northern Poland (Burakowski et al. 1976), Latvia (Telnov 2004), northwestern Belarus (Alexandrovitch et al. 1996), Denmark (Hansen 2009).

[*decorus* **(Gyllenhal, 1827)**]. Known in southern Sweden (Lundberg and Gustafsson 1995), Estonia (Hansen 2009), recently found in Latvia (Vorst et al. 2007).

*minutus* **(Linnaeus, 1758)**.Ogijewicz 1933; Pileckis 1960, 1976a; Silfverberg 1992, 2004; Gaidienė 1993; Pileckis and Monsevičius 1995; Šablevičius and Ferenca 1995; Šablevičius 2000b, 2011; Hansen 2004e, 2009; Ferenca 2006b; Vaivilavičius 2008; Alekseev 2010a.

*sinuatus* **Motschulsky, 1849**. Pileckis and Monsevičius 1982, 1995; Silfverberg 1992, 2004; Hansen 2004e, 2009; Alekseev 2010a.

*striatulus* **(Fabricius, 1801)**. Karalius and Monsevičius 1992; Pileckis and Monsevičius 1995; Silfverberg 1996, 2004; Alekseev 2010a.

**Hydrophilini Latreille, 1802**.

*Helochares* **Mulsant, 1844**.

*obscurus* **(O.F. Müller, 1776) =** *erythrocephalus* (Fabrius 1792). # 33. Ogijewicz 1933; Pileckis 1976a; Silfverberg 1992, 2004; Gaidienė 1993; Pileckis and Monsevičius 1995; Monsevičius 1997; Šablevičius 2000b; Hansen 2004e, 2009; Ferenca 2006b; Alekseev 2010a.

*lividus **(Forster, 1771) =** *griseus* (Fabricius, 1787). # 33. Pileckis 1976a; Silfverberg 1992, 2004; Hansen 2004e; Pileckis and Monsevičius 1995.

*Enochrus* **Thomson, 1859**.

*affinis* **(Thunberg, 1794)**. Ogijewicz 1933; Pileckis 1960, 1976a; Silfverberg 1992, 2004; Pileckis and Monsevičius 1995; Monsevičius 1997; Hansen 2004e, 2009; Ferenca 2006b; Alekseev 2010a.

*bicolor* **(Fabricius, 1792)**. Bercio and Folwaczny 1979; Silfverberg 1992, 2004; Gaidienė 1993; Pileckis and Monsevičius 1995; Hansen 2004e, 2009; Ferenca 2006a; Alekseev 2010a.

*coarctatus* **(Gredler, 1863)**. Pileckis 1960, 1976a; Silfverberg 1992, 2004; Pileckis and Monsevičius 1995; Monsevičius 1997; Hansen 2004e, 2009; Ferenca 2006b; Alekseev 2010a.

*fuscipennis* **(Thomson, 1884) =** *sahlbergi* (Fauvel, 1887). # 34. Pileckis 1960; Pileckis 1976a; Gaidienė 1993; Pileckis and Monsevičius 1995; Silfverberg 2004; Ferenca 2006b; Alekseev 2010a.

[*halophilus* **(Bedel, 1878)**]. Known in southern Sweden (Lundberg and Gustafsson 1995), Denmark (Hansen 2009).

*melanocephalus* **(Olivier, 1792)**. Monsevičius 1986a, 1997; Silfverberg 1992, 2004; Gaidienė 1993; Pileckis and Monsevičius 1995; Hansen 2004e, 2009; Ivinskis et al. 2009; Alekseev 2010a.

*ochropterus* **(Marsham, 1802) =** *frontalis* (Erichson, 1837). Ogijewicz 1933; Pileckis 1960, 1976a; Silfverberg 1992, 2004; Gaidienė 1993; Pileckis and Monsevičius 1995; Monsevičius 1997; Šablevičius 2000b; Hansen 2004e, 2009; Alekseev 2010a.

*quadripunctus* **(Herbst, 1797)**. # 34.Ogijewicz 1933; Pileckis 1960, 1976a; Silfverberg 1992, 2004; Pileckis and Monsevičius 1995; Monsevičius 1997; Hansen 2004e, 2009; Ferenca 2006b; Alekseev 2010a.

*testaceus* **(Fabricius, 1801)**. Ogijewicz 1933; Pileckis 1960, 1963b, 1976a; Zajančkauskas and Pileckis 1968; Silfverberg 1992, 2004; Gaidienė 1993; Pileckis and Monsevičius 1995; Monsevičius 1997; Šablevičius 2000b; Hansen 2004e, 2009; Ferenca 2006b; Alekseev 2010a.

*Cymbiodyta* **Bedel, 1881**.

*marginella* **(Fabricius, 1792)**.Pileckis and Monsevičius 1982, 1995; Silfverberg 1992, 2004; Gaidienė 1993; Monsevičius 1997; Hansen 2004e, 2009; Vaivilavičius 2008; Alekseev 2010a.

*Hydrobius* **Leach, 1815**.

*fuscipes* **(Linnaeus, 1758) =** *subrotundus* Stephens, 1829 = *rottenbergi* Gerhardt, 1872. Ogijewicz 1933; Pileckis 1960, 1976a; Silfverberg 1992, 2004; Gaidienė 1993; Pileckis and Monsevičius 1995; Monsevičius 1997; Tamutis and Zolubas 2001; Ferenca 2006b; Ivinskis et al. 2008; Hansen 2004e, 2009; Alekseev 2010a.

*Limnoxenus* **Motschulsky, 1853**.

*niger* **(Gmelin, 1790) =** *picipes* (Fabricius, 1787). Pileckis and Monsevičius 1995.

*Hydrochara* **Berthold, 1827**.

*caraboides* **(Linnaeus, 1758)**. Eichwald 1830; Ogijewicz 1933; Pileckis 1960, 1963b, 1976a; Lešinskas and Pileckis 1967; Silfverberg 1992, 2004; Gaidienė 1993; Pileckis and Monsevičius 1995; Šablevičius 2000b, 2011; Gliaudys 2001; Hansen 2004e, 2009; Ferenca 2006b; Alekseev 2010a.

*Hydrophilus* **Geoffroy, 1762 =** *Hydrous* Linnaeus, 1775.

*aterrimus* **Eschscholtz, 1822**. Ogijewicz 1933; Pileckis 1960, 1963, 1976a; Silfverberg 1992, 2004; Gaidienė 1993; Pileckis and Monsevičius 1995; Hansen 2004e, 2009; Ferenca 2006b; Ivinskis et al. 2008; Alekseev 2010a; Šablevičius 2011.

*piceus* **(Linnaeus, 1758)**. Eichwald 1830; Ogijewicz 1933; Pileckis 1960, 1970a, b, 1976a; Lešinskas and Pileckis 1967; Zajančkauskas and Pileckis 1968; Silfverberg 1992, 2004; Gaidienė 1993; Pileckis and Monsevičius 1995; Monsevičius 1997; Šablevičius 2000b, 2011; Gliaudys 2001; Hansen 2004e, 2009; Ferenca 2006b; Ivinskis et al. 2008; Alekseev 2010a.

**Sphaeridiinae Latreille, 1802**.

**Coelostomatini Heyden, 1891 (1890)**

*Coelostoma* **Brullé, 1835**.

*orbiculare* **(Fabricius, 1775)**.Ogijewicz 1933; Mazurowa and Mazur 1939; Pileckis 1960, 1976a; Zajančkauskas and Pileckis 1968; Silfverberg 1992, 2004; Gaidienė 1993; Pileckis and Monsevičius 1995; Hansen 2004e, 2009; Ferenca 2006b; Monsevičius 1997; Vaivilavičius 2008; Ivinskis et al. 2008; Alekseev 2010a; Šablevičius 2011.

**Megasternini Mulsant, 1844.**

*Cercyon* **Leach, 1817**.

*analis* **(Paykull, 1798)**. Ogijewicz 1933; Pileckis 1960, 1976a; Bercio and Folwaczny 1979; Silfverberg 1992, 2004; Gaidienė 1993; Pileckis and Monsevičius 1995; Monsevičius 1997; Hansen 2004e, 2009; Šablevičius 2011.

*bifenestratus* **Küster, 1851**. Ogijewicz 1933; Pileckis 1960, 1976a; Bercio and Folwaczny 1979; Silfverberg 1992, 2004; Gaidienė 1993; Pileckis and Monsevičius 1995; Monsevičius 1997; Hansen 2004e, 2009; Ivinskis et al. 2009.

*castaneipenis* **Vorst, 2009.** Known in Latvia, Poland, Sweden (Vorst 2009).

*convexiusculus* **Stephens, 1829**. Roubal 1910; Ogijewicz 1933; Pileckis 1960, 1976a; Silfverberg 1992, 2004; Gaidienė 1993; Pileckis and Monsevičius 1995; Monsevičius 1997; Hansen 2004e, 2009; Ferenca 2006b.

[*depressus* **Stephens, 1829**]. Known in southern Sweden (Lundberg and Gustafsson 1995), Estonia, Denmark (Hansen 2009).

*granarius* **Erichson, 1837**. Pileckis 1960, 1976a; Silfverberg 1992, 2004; Gaidienė 1993; Pileckis and Monsevičius 1995; Monsevičius 1997; Hansen 2004e, 2009; Ferenca 2006b.

*haemorrhoidalis* **(Fabricius, 1775)**. Ogijewicz 1933; Mazurowa and Mazur 1939; Pileckis 1960, 1976a; Silfverberg 1992, 2004; Gaidienė 1993; Pileckis and Monsevičius 1995; Monsevičius 1997; Šablevičius 2000b; Tamutis and Zolubas 2001; Hansen 2004e, 2009; Ferenca 2006b.

*impressus* **(Sturm, 1807)**. Ogijewicz 1933; Pileckis 1960, 1976a; Silfverberg 1992, 2004; Gaidienė 1993; Pileckis and Monsevičius 1995; Monsevičius 1997; Hansen 2004e, 2009; Žiogas and Zolubas 2005.

*laminatus* **Sharp, 1873**. Monsevičius 1986a; Silfverberg 1992, 2004; Pileckis and Monsevičius 1995; Monsevičius 1997; Hansen 2004e, 2009; Ivinskis et al. 2009.

*lateralis* **(Marsham, 1802)**. Ogijewicz 1933; Pileckis 1960, 1976a; Silfverberg 1992, 2004; Gaidienė 1993; Pileckis and Monsevičius 1995; Monsevičius 1997; Ryndevich 2004; Hansen 2004e, 2009; Ferenca 2006b.

*littoralis* **(Gyllenhal, 1808)**. Pileckis 1960, 1976a, b, 1979; Bercio and Folwaczny 1979; Silfverberg 1992, 2004; Pileckis and Monsevičius 1995; Hansen 2004e, 2009; Ferenca 2006b.

*marinus* **Thomson, 1853**. Monsevičius 1986a, 1997; Silfverberg 1992, 2004; Pileckis and Monsevičius 1995; Hansen 2004e, 2009; Ivinskis et al. 2009.

*melanocephalus* **(Linnaeus, 1758)**. Ogijewicz 1933; Pileckis 1960, 1976a; Zajančkauskas and Pileckis 1968; Silfverberg 1992, 2004; Gaidienė 1993; Pileckis and Monsevičius 1995; Monsevičius 1997; Šablevičius 2000b, 2011; Hansen 2004e, 2009; Ferenca 2006b.

*nigriceps* **(Marsham, 1802) =** *atricapillus* (Marsham, 1802). Tamutis et al. 2008.

*obsoletus* **(Gyllenhal, 1808) =** *lugubris* auct. nec(Olivier, 1790). Gaidienė 1993; Silfverberg 2004.

*pygmaeus* **(Illiger, 1801)**. Ogijewicz 1933; Pileckis 1960, 1976a; Silfverberg 1992, 2004; Gaidienė 1993; Pileckis and Monsevičius 1995; Monsevičius 1997; Hansen 2004e, 2009; Ferenca 2006b; Šablevičius 2011.

*quisquilius* **(Linnaeus, 1761)**. Ogijewicz 1933; Pileckis 1960, 1976a; Zajančkauskas and Pileckis 1968; Silfverberg 1992, 2004; Gaidienė 1993; Pileckis and Monsevičius 1995; Monsevičius 1997; Hansen 2004e, 2009; Ferenca 2006b.

*sternalis* **Sharp, 1928**. Tamutis et al. 2008; Ivinskis et al. 2008.

*terminatus* **(Marsham, 1802)**. Ogijewicz 1933; Pileckis 1960, 1976a; Silfverberg 1992, 2004; Gaidienė 1993; Pileckis and Monsevičius 1995; Hansen 2004e, 2009.

*tristis* **(Illiger, 1801)**. Pileckis 1960, 1976a; Silfverberg 1992, 2004; Gaidienė 1993; Pileckis and Monsevičius 1995; Monsevičius 1997; Hansen 2004e, 2009; Ferenca 2006b.

*unipunctatus* **(Linnaeus, 1758)**.Ogijewicz 1933; Pileckis 1960, 1976a; Silfverberg 1992, 2004; Gaidienė 1993; Pileckis and Monsevičius 1995; Monsevičius 1997; Hansen 2004e, 2009; Šablevičius 2011.

*ustulatus* **(Preyssler, 1790)**. Ogijewicz 1933; Pileckis 1960, 1976a; Silfverberg 1992, 2004; Gaidienė 1993; Pileckis and Monsevičius 1995; Šablevičius 2000b; Hansen 2004e, 2009; Ferenca 2006b.

*Megasternum* **Mulsant, 1844**.

*concinnum* **(Marsham, 1802) =** *obscurum* (Marsham, 1802) = *boletophagum* (Marsham, 1802) nec Marsham, 1802. Pileckis 1976a; Silfverberg 1992, 2004; Gaidienė 1993; Pileckis and Monsevičius 1995; Monsevičius 1997; Hansen 2004e, 2009.

*Cryptopleurum* **Mulsant, 1844**.

*crenatum* **(Kugelann, 1794)**. Monsevičius 1986a, 1997; Silfverberg 1992, 2004; Pileckis and Monsevičius 1995; Hansen 2004e, 2009.

*minutum* **(Fabricius, 1775)**. Ogijewicz 1933; Pileckis 1960, 1976a; Silfverberg 1992, 2004; Gaidienė 1993; Pileckis and Monsevičius 1995; Monsevičius 1997; Šablevičius 2000b; Hansen 2004e, 2009; Ferenca 2006b.

[*subtile* **Sharp, 1884**]. Known in southern Sweden (Lundberg and Gustafsson 1995), Estonia (Süda 2009), northwestern Belarus (Alexandrovitch et al. 1996), Denmark (Hansen 2009).

**Sphaeridini Latreille, 1802. **

*Sphaeridium* **Fabricius, 1775**.

*bipustulatum* **Fabricius, 1781**. Eichwald 1830; Roubal 1910; Ogijewicz 1933; Pileckis 1960, 1976a; Silfverberg 1992, 2004; Gaidienė 1993; Pileckis and Monsevičius 1995; Monsevičius 1997; Šablevičius 2000b; Hansen 2004e, 2009; Ferenca 2006b.

*lunatum* **Fabricius, 1792**. Ogijewicz 1933; Mazurowa and Mazur 1939; Pileckis 1960, 1976a; Silfverberg 1992, 2004; Pileckis and Monsevičius 1995; Monsevičius 1997; Tamutis and Zolubas 2001; Hansen 2004e, 2009.

*marginatum* **Fabricius, 1787**. Monsevičius and Pankevičius 2001; Silfverberg 2004.

*scarabaeoides* **(Linnaeus, 1758)**. Eichwald 1830; Ogijewicz 1933; Mazurowa and Mazur 1939; Pileckis 1960, 1976a; Lešinskas and Pileckis 1967; Zajančkauskas and Pileckis 1968; Silfverberg 1992, 2004; Gaidienė 1993; Pileckis and Monsevičius 1995; Monsevičius 1997; Šablevičius 2000b, 2011; Hansen 2004e, 2009; Ferenca 2006b.

*substriatum* **Faldermann, 1838**. Mazurowa and Mazur 1939; Pileckis 1960, 1976a; Silfverberg 1992, 2004; Pileckis and Monsevičius 1995; Hansen 2004e, 2009.

**SPHAERITIDAE Shuckard, 1839**.

*Sphaerites* **Duftschmid, 1805**.

*glabratus* **(Fabricius, 1792)**. Pileckis 1968a, 1970a, 1976a, b, 1979; Pileckis and Monsevičius 1995; Silfverberg 1992, 2004; Ferenca 2004; Löbl 2004a, 2009.

**HISTERIDAE Gyllenhal, 1808**.

**Abraeinae MacLeay, 1819**.

**Abraeini MacLeay, 1819**.

*Chaetabraeus* **Portevin, 1929**.

*globulus* **(Creutzer, 1799)**. Mazurowa and Mazur 1939 (*Abraeus*); Pileckis 1960, 1976a (*Abraeus*); Pileckis and Monsevičius 1995; Silfverberg 2004; Mazur 2004.

*Abraeus* **Leach, 1817**.

[*granulum* **Erichson, 1839**]. Known in southern Sweden (Lundberg and Gustafsson 1995), northwestern Belarus (Alexandrovitch et al. 1996), Kaliningrad region (Alekseev and Bukejs 2010), northern Poland (Burakowski et al. 1978; Mazur 2004); Denmark (Lackner 2009).

[*parvulus* **Aubé, 1842**]. Known in southern Sweden (Lundberg and Gustafsson 1995), northeastern Poland (Burakowski et al. 1978; Mazur 2004), Denmark (Lackner 2009).

*perpusillus* **(Marsham, 1802) =** *globosus* (Hoffmann, 1803). Pileckis and Monsevičius 1995; Silfverberg 2004; Mazur 2004.

**Plegaderini Portevin, 1929**.

*Plegaderus* **Erichson, 1834**.

*caesus* **(Herbst, 1792)**. Pileckis 1968b; Pileckis 1976a; Bercio and Folwaczny 1979; Silfverberg 1992, 2004; Gaidienė 1993; Pileckis and Monsevičius 1995; Mazur 2004; Lackner 2009.

*dissectus* **Erichson, 1839**. # 35. Lentz 1879; Silfverberg 1992, 2004; Pileckis and Monsevičius 1995.

*saucius* **Erichson, 1834**. Pileckis 1976a; Silfverberg 1992, 2004; Gaidienė 1993; Pileckis and Monsevičius 1995; Šablevičius 2000a, 2001, 2003a, b, 2011; Mazur 2004; Lackner 2009.

*vulneratus* **(Panzer, 1797)**. Jakaitis 1973; Pileckis 1976a; Bercio and Folwaczny 1979; Silfverberg 1992, 2004; Gaidienė 1993; Pileckis and Monsevičius 1995; Monsevičius 1997; Šablevičius 2000b, 2003a, b; Mazur 2004; Lackner 2009.

**Acritini Wenzel, 1944**.

*Acritus* **LeConte, 1853**.

[*homoeopathicus* **Wollaston, 1857**]. Known in Latvia (Telnov 2004), Poland (Burakowski et al. 1978; Mazur 2004); Denmark (Lackner 2009).

*minutus* **(Herbst, 1792)**. Pileckis 1976a; Silfverberg 1992, 2004; Pileckis and Monsevičius 1995; Mazur 2004; Lackner 2009.

[*nigricornis* **(Hoffmann, 1803)**]. Known in southern Sweden (Lundberg and Gustafsson 1995), Latvia (Telnov 2004), northern Poland (Burakowski et al. 1978; Mazur 2004); Denmark (Lackner 2009).

*Aeletes* **Horn, 1873**.

[*atomarius* **(Aubé, 1842)**]. Known in southern Sweden (Lundberg and Gustafsson 1995), Poland (Burakowski et al. 1978; Mazur 2004); Denmark (Lackner 2009).

**Teretriini Bickhardt, 1914**.

*Teretrius* **Erichson, 1834**.

*fabricii* **Mazur, 1972 =** *picipes* (Fabricius, 1787) nec (Olivier, 1789). Pileckis 1976a; Silfverberg 1992, 2004; Pileckis and Monsevičius 1995; Tamutis and Zolubas 2001; Mazur 2004; Lackner 2009.

**Saprininae Blanchard, 1845**.

*Saprinus* **Erichson, 1834**.

*aeneus* **(Fabricius, 1775)**. Mazurowa and Mazur 1939; Pileckis 1960, 1976a; Bercio and Folwaczny 1979; Silfverberg 1992, 2004; Gaidienė 1993; Pileckis and Monsevičius 1995; Monsevičius 1997; Mazur 2004; Lackner 2009.

*immundus* **(Gyllenhal, 1827)**. Pileckis 1976a; Silfverberg 1992, 2004; Pileckis and Monsevičius 1995; Mazur 2004; Ferenca et al. 2006; Lackner 2009.

*planiusculus* **Motschulsky, 1849 =** *cuspidatus* Ihssen, 1949. Pileckis and Monsevičius 1982, 1995; Silfverberg 1992, 2004; Mazur 2004; Ferenca et al. 2006.

*rugifer* **(Paykull, 1809) =** *quadristriatus* auct. nec (Thunberg, 1794). Tenenbaum 1931; Pileckis 1960, 1963b, 1968b, 1976a; Silfverberg 1992, 2004; Pileckis and Monsevičius 1995; Ferenca 2006b; Lackner 2009.

*semistriatus* **(L.G. Scriba, 1790)**. Mazurowa and Mazur 1939; Pileckis 1960, 1976a; Silfverberg 1992, 2004; Gaidienė 1993; Pileckis and Monsevičius 1995; Monsevičius 1997; Mazur 2004; Ferenca 2006b; Lackner 2009.

[*subnitescens* **Bickhardt, 1909**]. Known in northeastern Poland (Burakowski et al. 1978; Mazur 2004), Latvia (Telnov 2004).

[*tenuistrius sparsutus* **Solskij, 1876**]. Known in Poland (Burakowski et al. 1978; Mazur 2004), northwestern Belarus (Alexandrovitch et al. 1996), Latvia (Telnov 2004).

*virescens* **(Paykull, 1798)**. Horion 1949; Pileckis 1968b, 1976a, 1982; Silfverberg 1992, 2004; Pileckis and Monsevičius 1995; Mazur 2004; Lackner 2009.

*Chalcionellus* **Reichardt, 1932**.

*decemstriatus* **(P. Rossi, 1792) =** *conjungens* (Paykull, 1798). Pileckis 1960, 1976a; Silfverberg 1992, 2004; Pileckis and Monsevičius 1995; Mazur 2004; Ferenca 2006b; Lackner 2009.

*Hypocacculus* **Bickhardt, 1916**.

*rufipes* **(Kugelann, 1792)**. Lentz 1879; Pileckis 1976a; Bercio and Folwaczny 1979; Silfverberg 1992, 2004; Pileckis and Monsevičius 1995; Mazur 2004.

*Hypocaccus* **Thomson, 1867 =** *Baeckmanniolus* Reichardt, 1926.

[*dimidiatus maritimus* **(Stephens, 1830)**]. Known in southern Sweden (Lundberg and Gustafsson 1995), Poland, Denmark (Lackner 2009).

*metallicus* **(Herbst, 1792)**. Mazurowa and Mazur 1939; Pileckis 1960, 1976a; Bercio and Folwaczny 1979; Silfverberg 1992, 2004; Pileckis and Monsevičius 1995; Mazur 2004; Ferenca 2006b; Lackner 2009.

*rugiceps* **(Duftschmid, 1805)**. Pileckis 1960, 1976a; Bercio and Folwaczny 1979; Silfverberg 1992, 2004; Gaidienė 1993; Pileckis and Monsevičius 1995; Ferenca 2004; Mazur 2004; Lackner 2009; Ivinskis et al. 2009.

*rugifrons* **(Paykull, 1798)**. Mazurowa and Mazur 1939; Pileckis 1960, 1963b, 1976a; Bercio and Folwaczny 1979; Silfverberg 1992, 2004; Pileckis and Monsevičius 1995; Ferenca 2004; Mazur 2004; Lackner 2009; Šablevičius 2011.

[*specularis* **(Marseul, 1855)**]. Known in northern Poland (Burakowski et al. 1978).

*Gnathoncus* **Jacquelin du Val, 1858**.

*buyssoni* **Auzat, 1917**. Pileckis and Monsevičius 1982, 1995; Silfverberg 1992, 2004; Monsevičius 1997, 1999; Tamutis and Zolubas 2001; Mazur 2004; Lackner 2009; Ivinskis et al. 2009.

*communis* **(Marseul, 1862) =** *schmidti* Reitter, 1894. Monsevičius 1999.

*nannetensis* **(Marseul, 1862)**. Monsevičius 1988b, 1997; Silfverberg 1992, 2004; Pileckis and Monsevičius 1995; Mazur 2004; Lackner 2009.

*nidorum* **Stockmann, 1957**. Monsevičius 1988b, 1997; Silfverberg 1992, 2004; Pileckis and Monsevičius 1995; Monsevičius 1999; Mazur 2004; Lackner 2009.

*rotundatus* **(Kugelann, 1792) =** *nanus* (L.G. Scriba, 1790) nec (Piller & Mitterpacher, 1783) *= punctulatus* Thomson, 1862. Tenenbaum 1923, 1931; Pileckis 1968b, 1976a; Jakaitis and Valenta 1976; Silfverberg 1992, 2004; Pileckis and Monsevičius 1995; Mazur 2004; Lackner 2009.

*Myrmetes* **Marseul, 1862**.

*paykulli* **Kanaar, 1979 =** *piceus* (Paykull, 1809) nec (Marsham, 1802). Łomnicki 1913; Pileckis 1976a; Silfverberg 1992, 2004; Pileckis and Monsevičius 1995; Mazur 2004; Lackner 2009.

**Dendrophilinae Reitter, 1909**.

**Dendrophilini Reitter, 1909**.

*Dendrophilus* **Leach, 1817**.

*corticalis* **(Paykull, 1798) =** *punctatus* (Herbst, 1792) nec (O.F. Müller, 1776). Monsevičius 1988b, 1997; Silfverberg 1992, 2004; Pileckis and Monsevičius 1995; Mazur 2004; Lackner 2009.

*pygmaeus* **(Linnaeus, 1758)**. Pileckis 1976a; Silfverberg 1992, 2004; Pileckis and Monsevičius 1995; Monsevičius 1997; Mazur 2004; Lackner 2009.

**Paromalini Reitter, 1909**.

*Carcinops* **Marseul, 1855**.

*pumilio* **(Erichson, 1834) =** *quatourdecimstriatus* (Staphens, 1835). Monsevičius 1988b, 1997; Silfverberg 1992, 2004; Pileckis and Monsevičius 1995; Mazur 2004; Lackner 2009.

*Platylomalus* **Cooman, 1948**.

*complanatus* **(Panzer, 1797)**. Mazur 2004; Inokaitis 2009.

*Paromalus* **Erichson, 1834**.

*flavicornis* **(Herbst, 1792)**. Tamutis 2003.

*parallelepipedus* **(Herbst, 1792)**. Pileckis 1968b, 1976a, 1982; Silfverberg 1992, 2004; Gaidienė 1993; Pileckis and Monsevičius 1995; Monsevičius 1997; Šablevičius 2000b, 2011; Mazur 2004; Lackner 2009.

**Onthophilinae MacLeay, 1819**.

*Onthophilus* **Leach, 1817**.

[*punctatus* **(O.F. Müller, 1776) =** *sulcatus* (Moll, 1784)]. Known in northern Poland (Burakowski et al. 1978; Mazur 2004), Denmark (Lackner 2009).

[*striatus* **(Forster, 1771)**]. Known in southern Sweden (Lundberg and Gustafsson 1995), northern Poland (Burakowski et al. 1978; Mazur 2004), Estonia, Denmark (Lackner 2009).

**Histerinae Gyllenhal, 1808**.

**Histerini Gyllenhal, 1808**.

*Margarinotus* **Marseul, 1853**.

*bipustulatus* **(Schrank, 1781)**. Pileckis 1960, 1976a, 1982; Silfverberg 1992, 2004; Pileckis and Monsevičius 1995; Šablevičius 2003a; Mazur 2004; Lackner 2009.

*brunneus* **(Fabricius, 1775) =** *impressus* (Fabricius, 1798) = *cadaverinus* (Hoffmann, 1803). Heyden 1903; Pileckis 1960, 1976a; Lešinskas and Pileckis 1967; Silfverberg 1992, 2004; Gaidienė 1993; Pileckis and Monsevičius 1995; Šablevičius 2000b, 2011; Gliaudys 2001; Mazur 2004; Lackner 2009.

*carbonarius* **(Hoffmann, 1803)**. Roubal 1910; Mazurowa and Mazur 1939; Pileckis 1960, 1976a; Silfverberg 1992, 2004; Gaidienė 1993; Pileckis and Monsevičius 1995; Mazur 2004; Ferenca 2006b; Lackner 2009.

[*marginatus* **(Erichson, 1834)**]. Known in southern Sweden (Lundberg and Gustafsson 1995), northwestern Belarus (Alexandrovitch et al. 1996), northern Poland (Burakowski et al. 1978; Mazur 2004), Latvia (Telnov 2004), Estonia, Denmark (Lackner 2009).

*merdarius* **(Hoffmann, 1803)**. Pileckis 1960, 1976a; Silfverberg 1992, 2004; Gaidienė 1993; Pileckis and Monsevičius 1995; Monsevičius 1997; Mazur 2004; Ferenca 2006b; Lackner 2009.

*neglectus* **(Germar, 1813)**. Pileckis 1960, 1976a; Pileckis and Monsevičius 1982, 1995; Silfverberg 1992, 2004; Tamutis and Zolubas 2001; Mazur 2004; Ferenca 2006b; Lackner 2009.

*obscurus* **(Kugelann, 1792) =** *stercorarius* (Hoffmann, 1803). Gaidienė 1993; Tamutis 2003; Silfverberg 2004.

*purpurascens* **(Herbst, 1792)**. Pileckis 1960, 1976a; Pileckis and Monsevičius 1982, 1995; Silfverberg 1992, 2004; Gaidienė 1993; Monsevičius 1997; Mazur 2004; Ferenca 2006b; Lackner 2009.

*striola* **(C.R. Sahlberg, 1819) =** *succicola* (Thomson, 1862). Heyden 1903; Pileckis 1960, 1976a (*H. striela*); Silfverberg 1992, 2004; Gaidienė 1993; Pileckis and Monsevičius 1995; Monsevičius 1997; Mazur 2004; Lackner 2009.

*terricola* **(Germar, 1824)**. Tenenbaum 1931; Pileckis 1960, 1968b, 1976a; Silfverberg 1992, 2004; Gaidienė 1993; Pileckis and Monsevičius 1995; Mazur 2004; Lackner 2009.

*ventralis* **(Marseul, 1854)**. Roubal 1910; Mazurowa and Mazur 1939; Pileckis 1960, 1976a; Silfverberg 1992, 2004; Gaidienė 1993; Pileckis and Monsevičius 1995; Šablevičius 2000b, 2003a; Mazur 2004; Lackner 2009.

*Hister* **Linnaeus, 1758**.

*bissexstriatus* **Fabricius, 1801**.Pileckis 1960, 1976a; Silfverberg 1992, 2004; Gaidienė 1993; Pileckis and Monsevičius 1995; Mazur 2004; Ferenca 2006b; Lackner 2009.

[*funestus* **Erichson, 1834**]. Recently found in Latvia (Telnov et al. 2006, 2008), known in southern Sweden (Lundberg and Gustafsson 1995), northeastern Poland (Burakowski et al. 1978; Mazur 2004), Kaliningrad region, Estonia, Denmark (Lackner 2009).

*helluo* **Truqui, 1852**. Pileckis 1976a; Silfverberg 1992, 2004; Pileckis and Monsevičius 1995; Mazur 2004; Lackner 2009.

[*illigeri* **Duftschmid, 1805**]. Known in Estonia (Lackner 2009), Belarus (Alexandrovitch et al. 1996), northern Poland (Burakowski et al. 1978; Mazur 2004).

[*quadrimaculatus* **Linnaeus, 1758**]. Known in northern Belarus (Alexandrovitch et al. 1996), Latvia (Telnov 2004), northern Poland (Burakowski et al. 1978; Mazur 2004), Estonia, Denmark (Lackner 2009).

*quadrinotatus* **L.G. Scriba, 1790**. Mazurowa and Mazur 1939; Pileckis 1960, 1976a; Lešinskas and Pileckis 1967; Silfverberg 1992, 2004; Gaidienė 1993; Pileckis and Monsevičius 1995; Gliaudys 2001; Mazur 2004; Ferenca 2006b; Lackner 2009; Šablevičius 2011.

*unicolor* **Linnaeus, 1758**. Eichwald 1830; Mazurowa and Mazur 1939; Pileckis 1960, 1976a; Silfverberg 1992, 2004; Gaidienė 1993; Pileckis and Monsevičius 1995; Monsevičius 1997; Šablevičius 2000b; Tamutis and Zolubas 2001; Mazur 2004.

*Atholus* **Thomson, 1859**.

*bimaculatus* **(Linnaeus, 1758)**. Mazurowa and Mazur 1939; Pileckis 1960, 1976a; Silfverberg 1992, 2004; Gaidienė 1993; Pileckis and Monsevičius 1995; Gliaudys 2001; Mazur 2004; Lackner 2009.

*corvinus* **(Germar, 1817)**.Pileckis 1960, 1976a; Silfverberg 1992, 2004; Pileckis and Monsevičius 1995; Mazur 2004; Ferenca 2006b; Lackner 2009.

*duodecimstriatus* **(Schrank, 1781)**. Pileckis 1976a; Silfverberg 1992, 2004; Gaidienė 1993; Pileckis and Monsevičius 1995; Monsevičius 1997; Mazur 2004; Lackner 2009.

[*praetermissus* **(Peyron, 1856)**]. Known in Estonia, Denmark (Lackner 2009), Belarus (Alexandrovitch et al. 1996), Poland (Burakowski et al. 1978; Mazur 2004).

**Platysomatini Bickhardt, 1914**.

*Cylister* **Cooman, 1941**.

*angustatum* **(Hoffmann, 1803) =** *ferrugineum* (Thunberg, 1794) nec (Olivier, 1789). Pileckis 1960, 1976a; Silfverberg 1992, 2004; Gaidienė 1993; Pileckis and Monsevičius 1995 (*Platysoma*); Mazur 2004; Lackner 2009.

*elongatus* **(Thunberg, 1787) =** *oblongus* (Fabricius, 1792). Pileckis 1960, 1976a; Silfverberg 1992, 2004; Pileckis and Monsevičius 1995 (*Platysoma*); Monsevičius 1997; Mazur 2004; Lackner 2009; Šablevičius 2011.

*lineare* **Erichson, 1834**. Pileckis 1968a, 1976a; Silfverberg 1992, 2004; Gaidienė 1993; Pileckis and Monsevičius 1995 (*Platysoma*); Mazur 2004.

*Platysoma* **Leach, 1817**.

*compressum* **(Herbst, 1783)**. Lešinskas and Pileckis 1967; Pileckis 1976a; Silfverberg 1992, 2004; Pileckis and Monsevičius 1995; Mazur 2004; Lackner 2009.

*deplanatum* **(Gyllenhal, 1808)**. Pileckis 1968a, 1976a; Silfverberg 1992, 2004; Pileckis and Monsevičius 1995; Šablevičius 2000a, 2003a, 2004; Mazur 2004; Lackner 2009.

*Emblisia* **Lewis, 1889**.

*minor* **(P. Rossi, 1792) =** *frontale* (Paykull, 1798). Pileckis 1960, 1976a; Silfverberg 1992, 2004; Gaidienė 1993; Pileckis and Monsevičius 1995 (*Platysoma*); Tamutis and Zolubas 2001; Šablevičius 2003a; Mazur 2004; Ferenca 2006b; Lackner 2009.

**Hololeptini Hope, 1840**.

*Hololepta* **Paykull, 1811**.

*plana* **(Sulzer, 1776)**. Pileckis and Monsevičius 1982, 1995; Silfverberg 1992, 2004; Gaidienė 1993; Šablevičius 2003a, 2011; Mazur 2004; Butvila et al. 2007; Lackner 2009; Inokaitis 2009.

**Haeteriinae Marseul, 1857**.

**Haeteriini Marseul, 1857**.

*Haeterius* **Erichson, 1834**.

*ferrugineus* **(Olivier, 1789)**. Pileckis 1988; Silfverberg 1992, 2004; Pileckis and Monsevičius 1995; Šablevičius 2003a, 2011; Mazur 2004; Lackner 2009.

**STAPHYLINOIDEA Latreille, 1802**.

**HYDRAENIDAE Mulsant, 1844**.

**Hydraeninae Mulsant, 1844**.

**Limnebiini Mulsant, 1844**.

*Limnebius* **Leach, 1815**.

[*aluta* **Bedel, 1881**]. Recently found in Latvia (Telnov et al. 2006), known in Kaliningrad region (Alekseev 2010a), southern Sweden (Lundberg and Gustafsson 1995), northern Poland (Burakowski et al. 1976), Denmark (Jäch 2004, 2009a).

*atomus* **(Duftshmid, 1805)**. Ferenca and Tamutis 2009; Alekseev 2010a.

*crinifer* **Rey, 1885**. Bercio and Folwaczny 1979; Miländer et al. 1984; Pileckis and Monsevičius 1995; Monsevičius 1997; Silfverberg 2004; Jäch 2004, 2009a; Alekseev 2010a.

*nitidus* **(Marsham, 1802)**. Pileckis and Monsevičius 1995; Silfverberg 2004; Alekseev 2010a.

*papposus* **Mulsant, 1844**. Pileckis and Monsevičius 1995; Silfverberg 2004; Alekseev 2010a.

*parvulus* **(Herbst, 1797) =** *truncatulus* Thomson, 1853. Karalius and Monsevičius 1992; Silfverberg 1996, 2004; Alekseev 2010a.

*truncatellus* **(Thunberg, 1794)**. Ogijewicz 1933; Pileckis 1960, 1976a; Silfverberg 1992, 2004; Gaidienė 1993; Pileckis and Monsevičius 1995; Jäch 2004, 2009a; Alekseev 2010a.

**Hydraenini Mulsant, 1844**.

*Hydraena* **Kugelann, 1794**.

*britteni* **Joy, 1907**. Pileckis and Monsevičius 1982, 1995; Silfverberg 1992, 2004; Monsevičius 1997; Jäch 2004, 2009a; Vorst et al. 2007; Alekseev 2010a.

[*excisa* **Kiesenwetter, 1849**]. Known in Latvia (Telnov 2004), northern Poland (Burakowski et al. 1976), Belarus (Jäch 2004, 2009a).

*gracilis* **Germar, 1824**. Lindeman 1871; Lentz 1879; Pileckis 1968b, 1976a; Bercio and Folwaczny 1979; Silfverberg 1992, 2004; Pileckis and Monsevičius 1995; Jäch 2004, 2009a; Alekseev 2010a.

[*melas* **Dalla Torre, 1877**]. Known in Estonia (Mahler 2004), Poland (Jäch 2004, 2009a).

*minutissima* **Stephens, 1829**. Buczyński et al. 2008; Alekseev 2010a.

[*nigrita* **Germar, 1824**]. Known in Sweden, Denmark, Estonia (Lundberg and Gustafsson 1995; Silfverberg 1992, 2004), Poland (Burakowski et al. 1976).

*palustris* **Erichson, 1837**. Ferenca and Tamutis 2009; Alekseev 2010a.

*pulchella* **Germar, 1824 = flavipes Sturm, 1836**. Buczyński et al. 2008; Alekseev 2010a.

*reyi* **Kuwert, 1888 = sternalis auct. nec Rey, 1893**. Buczyński et al. 2008; Alekseev 2010a.

*riparia* **Kugelann, 1794**. Ogijewicz 1933; Pileckis 1960, 1976a; Silfverberg 1992, 2004; Pileckis and Monsevičius 1995; Monsevičius 1997; Jäch 2004, 2009a; Alekseev 2010a.

[*testacea* **Curtis, 1830**]. Known in southern Sweden (Lundberg and Gustafsson 1995), Denmark (Jäch 2004, 2009a).

**Ochthebiinae Thomson, 1859**.

**Ochthebiini Thomson, 1859**.

*Ochthebius* **Leach, 1815**.

[*auriculatus* **Rey, 1886**]. Known in southern Sweden (Lundberg and Gustafsson 1995), Denmark (Jäch 2004, 2009a).

*bicolon* **Germar, 1824**. Monsevičius 1998.

[*dilatatus* **Stephens, 1829**]. Known in southern Sweden (Lundberg and Gustafsson 1995), Denmark (Jäch 2004, 2009a).

*exculptus* **Leach, 1829**.Ivinskis et al. 1999.

[*flavipes* **Dalla Torre, 1877**]. Recently found in Latvia (Telnov et al. 2006), known in Poland (Jäch 2004, 2009a).

[*gibbosus* **Germar 1824**]. Known in Kalingrad region (Alekseev 2010a), northern Poland (Burakowski et al. 1976).

[*hungaricus* **Endrödy-Younga, 1967**]. Recently found in Latvia (Vorst et al. 2007), known in Belarus, Estonia, Poland (Jäch 2004, 2009a).

[*marinus* **(Paykull, 1798)**]. Known in southern Sweden (Lundberg and Gustafsson 1995), Latvia (Telnov 2004), northern Poland (Burakowski et al. 1976), Estonia, Denmark (Jäch 2004, 2009a).

*metallescens **Rosenhauer, 1847**. # 36. Ogijewicz 1933; Pileckis 1960, 1970b, 1976a; Pileckis and Monsevičius 1995; Silfverberg 2004; Alekseev 2010a.

*minimus* **(Fabricius, 1792) =** *impressus* (Marsham, 1802). Ogijewicz 1933; Pileckis 1960, 1976a; Silfverberg 1992, 2004; Pileckis and Monsevičius 1995; Monsevičius 1997; Jäch 2004, 2009a; Alekseev 2010a.

*pusillus* **Stephens, 1835**. Pileckis 1976a; Silfverberg 1992, 2004; Gaidienė 1993; Pileckis and Monsevičius 1995; Ferenca et al. 2002; Jäch 2004, 2009a; Ivinskis et al. 2009; Alekseev 2010a.

[*remotus* **Reitter, 1887**]. Known in Latvia (Telnov 2004).

[*viridis* **Peyron, 1858**]. Known in southern Sweden (Lundberg and Gustafsson 1995), Denmark, Poland (Jäch 2004, 2009a).

**PTILIIDAE Erichson, 1845**.

**Ptiliinae Erichson, 1845**.

**Ptenidiini Flach. 1889**.

*Nossidium* **Erichson, 1845**.

[*pilosellum* **(Marsham, 1802)**]. Known in Belarus (Alexandrovitch et al. 1996); Denmark (Polilov 2009), Poland (Burakowski et al. 1978).

*Ptenidium* **Erichson, 1845**.

*formicetorum* **Kraatz, 1851** = *myrmecophilum* (Motschulsky, 1845) nec (Allibert, 1844). Monsevičius and Pankevičius 2001; Johnson 2004; Polilov 2009.

*fuscicorne* **Erichson, 1845**. Pileckis 1976a; Silfverberg 1992, 2004; Pileckis and Monsevičius 1995; Johnson 2004; Vorst et al. 2007.

*gressneri* **Erichson, 1845**. Łomnicki 1913; Pileckis 1968b, 1976a; Silfverberg 1992, 2004; Pileckis and Monsevičius 1995; Johnson 2004; Polilov 2009.

*intermedium* **Wankowicz, 1869**. Pileckis 1976a; Bercio and Folwaczny 1979; Silfverberg 1992, 2004; Pileckis and Monsevičius 1995; Johnson 2004; Polilov 2009; Ivinskis et al. 2009.

[*laevigatum* **Erichson, 1845**]. Known in Latvia (Telnov 2004), southern Sweden (Lundberg and Gustafsson 1995), Poland (Burakowski et al. 1978), Denmark, Belarus (Polilov 2009).

[*longicorne* **Fuss, 1868**]. Known in Latvia (Telnov 2004), Poland (Polilov 2009).

*nitidum* **(Heer, 1841)**. Ferenca et al. 2002, 2006.

[*punctatum* **(Gyllenhal, 1827)**]. Recently found in Latvia (Vorst et al. 2007), known in southern Sweden (Lundberg and Gustafsson 1995), northern Poland (Burakowski et al. 1978), Denmark (Polilov 2009).

*pusillum* **(Gyllenhal, 1808)**. Pileckis 1976a; Silfverberg 1992, 2004; Pileckis and Monsevičius 1995; Johnson 2004; Polilov 2009.

*turgidum* **Thomson, 1855**. Horion 1949; Pileckis 1968b, 1976a; Silfverberg 1992, 2004; Pileckis and Monsevičius 1995; Polilov 2009.

**Ptiliini Erichson, 1845**.

*Actidium* **Matthews, 1868**.

[*boudieri* **(Allibert, 1844)**]. Known in Latvia (Barševskis 2001b; Telnov 2004), northern Poland (Burakowski et al. 1978), Denmark, (Polilov 2009).

[*coarctatum* **(Haliday, 1855)**]. Known in southern Sweden (Lundberg and Gustafsson 1995), Denmark (Polilov 2009), Poland (Burakowski et al. 1978).

[*reticulatum* **Besuchet, 1971**]. Recently found in Latvia (Vorst et al. 2007).

*Oligella* **Motschulsky, 1869**.

[*foveolata* **(Allibert, 1844)**]. Known in Latvia (Barševskis 2001b; Telnov 2004), southern Sweden (Lundberg and Gustafsson 1995), Denmark (Polilov 2009), Poland (Burakowski et al. 1978).

[*intermedia* **Besuchet, 1971**]. Known in southern Sweden (Lundberg and Gustafsson 1995).

[*nana* **(Strand, 1946)**]. Known in southern Sweden (Lundberg and Gustafsson 1995).

*Micridium* **Motschulsky, 1869**.

[*angulicolle* **(Fairmaire, 1857)**]. Known in southern Sweden (Lundberg and Gustafsson 1995).

[*halidaii* **(Matthews, 1868)**]. Known in Latvia (Barševskis 2001b; Telnov 2004), southern Sweden (Lundberg and Gustafsson 1995), Belarus (Alexandrovitch et al. 1996), Poland (Burakowski et al. 1978), Denmark (Polilov 2009).

*Millidium* **Motschulsky, 1855**.

[*minutissimum* **(Ljungh, 1804)**]. Known in Latvia (Barševskis 2001b; Telnov 2004), southern Sweden (Lundberg and Gustafsson 1995), Belarus (Alexandrovitch et al. 1996), Poland (Burakowski et al. 1978), Denmark, Estonia (Polilov 2009).

*Ptilium* **Gyllenhal, 1827**.

[*affine* **Erichson, 1845**]. Known in southern Sweden (Lundberg and Gustafsson 1995), Poland (Burakowski et al. 1978), Denmark (Polilov 2009).

[*caesum* **Erichson, 1845**]. Known in southern Sweden (Lundberg and Gustafsson 1995), Poland (Burakowski et al. 1978).

[*exaratum* **(Allibert, 1844)**]. Known in Latvia (Barševskis 2001b; Telnov 2004), southern Sweden (Lundberg and Gustafsson 1995), Poland (Burakowski et al. 1978), Estonia, Denmark (Polilov 2009).

[*horioni* **Rosskothen, 1934**]. Known in southern Sweden (Lundberg and Gustafsson 1995), Denmark (Polilov 2009).

[*myrmecophilum* **(Allibert, 1844)**]. Known in Latvia (Telnov 2004), southern Sweden (Lundberg and Gustafsson 1995), northern Belarus (Alexandrovitch et al. 1996), Poland (Burakowski et al. 1978), Denmark (Polilov 2009).

*modestum* **Wankowicz, 1869**. Pileckis 1976a; Silfverberg 1992, 2004; Pileckis and Monsevičius 1995; Johnson 2004; Polilov 2009.

*Euryptilium* **Matthews, 1872**.

[*gillmeisteri* **Flach, 1889**]. Known in Latvia (Barševskis 2001b; Telnov 2004), Denmark, Poland (Polilov 2009).

[*saxonicum* **(Gillmeister, 1845) =** *marginatum* auct. nec (Aubé, 1850)]. Recently found in Latvia (Telnov et al. 2010), known in southern Sweden (Lundberg and Gustafsson 1995), Poland (Burakowski et al. 1978), Denmark (Polilov 2009).

*Ptiliola* **Haldeman, 1848 =** *Nanoptilium* Flach, 1889.

[*brevicollis* **(Matthews, 1860)**].Known in Latvia (Barševskis 2001b; Telnov 2004), southern Sweden (Lundberg and Gustafsson 1995), Denmark, Poland (Polilov 2009).

[*kunzei* **(Heer, 1841)**]. Known in Latvia (Barševskis 2001b; Telnov 2004), southern Sweden (Lundberg and Gustafsson 1995), Estonia, Denmark (Polilov 2009), Poland (Burakowski et al. 1978).

*Ptiliolum* **Flach, 1888**.

[*caledonicum* **(Sharp, 1871) =** *croaticum* (Matthews, 1872)]. Known in southern Sweden (Lundberg and Gustafsson 1995), Estonia (Polilov 2009).

[*fuscum* **(Erichson, 1845) =** *flachi* (Reitter, 1909)]. Known in southern Sweden (Lundberg and Gustafsson 1995), Denmark (Polilov 2009), Poland (Burakowski et al. 1978).

[*marginatum* **(Aubé, 1850) =** *lederi* (Flach, 1888)]. Known in southern Sweden (Lundberg and Gustafsson 1995), Denmark (Polilov 2009).

[*sahlbergi* **(Flach, 1888)**]. Known in Latvia (Telnov 2004), southern Sweden (Lundberg and Gustafsson 1995), Poland (Burakowski et al. 1978), Estonia (Polilov 2009).

[*schwarzi* **(Flach, 1887)**]. Known in southern Sweden (Lundberg and Gustafsson 1995), Poland (Burakowski et al. 1978, Estonia, Denmark (Polilov 2009).

[*spencei* **(Allibert, 1844)**]. Known in southern Sweden (Lundberg and Gustafsson 1995), Belarus (Alexandrovitch et al. 1996), northern Poland (Burakowski et al. 1978), Estonia, Denmark (Polilov 2009).

[*wuesthoffi* **Rosskothen, 1934**]. Known in southern Sweden (Lundberg and Gustafsson 1995), Denmark, Poland (Polilov 2009).

**Ptinellini Reitter, 1906 (1891)**.

*Ptinella* **Motschulsky, 1844**.

[*aptera* **(Guérin-Ménéville, 1839)**]. Known in southern Sweden (Lundberg and Gustafsson 1995), Belarus (Alexandrovitch et al. 1996), Poland (Burakowski et al. 1978), Denmark (Polilov 2009).

[*denticollis* **(Fairmaire, 1857)**]. Known in southern Sweden (Lundberg and Gustafsson 1995), Poland (Burakowski et al. 1978), Denmark (Polilov 2009).

*limbata* **(Heer, 1841) =** *testacea* (Heer, 1841). Horion 1949; Pileckis 1968b; 1976a; Pileckis and Monsevičius 1995; Silfverberg 1992, 2004; Johnson 2004; Polilov 2009.

[*tenella* **(Erichson, 1845)**]. Recently found in Latvia (Telnov et al. 2007), known in southern Sweden (Lundberg and Gustafsson 1995), Poland (Burakowski et al. 1978), Denmark (Polilov 2009).

*Pteryx* **Matthews, 1858**.

[*splendens* **Strand, 1960**]. Known in southern Sweden (Lundberg and Gustafsson 1995), Poland (Polilov 2009).

[*suturalis* **(Heer, 1841)**]. Known in Latvia (Barševskis 2001b; Telnov 2004), southern Sweden (Lundberg and Gustafsson 1995), Belarus (Alexandrovitch et al. 1996), northern Poland (Burakowski et al. 1978), Denmark (Polilov 2009).

**Acrotrichinae Reitter, 1909 (1856)**.

*Nephanes* **Thomson, 1859**.

*titan* **(Newman, 1834)**. Bercio and Folwaczny 1979; Silfverberg 1992, 2004; Pileckis and Monsevičius 1995; Johnson 2004; Ferenca et al. 2006; Polilov 2009.

*Smicrus* **Matthews, 1872**.

[*filicornis* **(Fairmaire & Laboulbène, 1855)**]. Known in Latvia (Barševskis 2001; Telnov 2004), southern Sweden (Lundberg and Gustafsson 1995), Poland (Burakowski et al. 1978), Denmark (Polilov 2009).

*Baeocrara* **Thomson, 1859**.

[*japonica* **(Matthews, 1884)**]. Known in Latvia (Barševskis 2001b; Telnov 2004), southern Sweden (Lundberg and Gustafsson 1995), Denmark (Polilov 2009).

*variolosa* **(Mulsant & Rey, 1873) =** *littoralis* (Thomson, 1855) nec (Motschulsky, 1845). Łomnicki 1913; Pileckis 1968b, 1976a; Silfverberg 1992, 2004; Pileckis and Monsevičius 1995; Johnson 2004; Polilov 2009.

*Acrotrichis* **Motschulsky, 1848**.

[*arnoldi* **Rosskothen, 1935**]. Known in southern Sweden (Lundberg and Gustafsson 1995), Denmark (Polilov 2009).

*atomaria* **(DeGeer, 1774)**. Pileckis 1962, 1963b, 1976a; Silfverberg 1992, 2004; Pileckis and Monsevičius 1995; Johnson 2004; Polilov 2009.

*brevipennis* **(Erichson, 1845)**. Polilov 2009.

[*cognata* **(Matthews, 1877) =** *platonoffi* Renkonen, 1945]. Known in southern Sweden (Lundberg and Gustafsson 1995), Estonia, Denmark (Polilov 2009).

[*danica* **Sundt, 1958**]. Known in southern Sweden (Lundberg and Gustafsson 1995), Denmark, Poland (Polilov 2009).

*dispar* **(Matthews, 1865)**. Mikutowicz 1905; Horion 1949; Pileckis 1976a, 1968b; Silfverberg 1992, 2004; Pileckis and Monsevičius 1995; Johnson 2004; Polilov 2009.

*fascicularis* **(Herbst, 1793)**. Pileckis 1976a; Silfverberg 1992, 2004; Pileckis and Monsevičius 1995; Johnson 2004; Ferenca et al. 2006; Polilov 2009.

*grandicollis* **(Mannerheim, 1844)**. Roubal 1910; Pileckis 1960, 1976a; Lešinskas and Pileckis 1967; Silfverberg 1992, 2004; Pileckis and Monsevičius 1995; Johnson 2004; Polilov 2009.

[*insularis* **(Mäklin, 1852)**]. Recently found in Latvia (Telnov et al. 2007), known in southern Sweden (Lundberg and Gustafsson 1995), Estonia, Denmark (Polilov 2009).

*intermedia* **(Gillmeister, 1845)**. Pileckis 1976a; Silfverberg 1992, 2004; Pileckis and Monsevičius 1995; Johnson 2004; Polilov 2009.

[*lucidula* **Rosskothen, 1935**]. Known in southern Sweden (Lundberg and Gustafsson 1995), Denmark, Poland (Polilov 2009).

*montandoni* **(Allibert, 1844)**. Pileckis 1976a; Silfverberg 1992, 2004; Pileckis and Monsevičius 1995; Johnson 2004; Polilov 2009.

[*norvegica* **Strand, 1941**]. Known in southern Sweden (Lundberg and Gustafsson 1995), northeastern Poland (Burakowski et al. 1978), Denmark (Polilov 2009).

[*parva* **Rosskothen, 1935**]. Known in Latvia (Barševskis 2001b; Telnov 2004), southern Sweden (Lundberg and Gustafsson 1995), Denmark (Polilov 2009).

[*pumila* **(Erichson, 1845) =** *longicornis* auct. nec (Mannerheim, 1844)]. Known in southern Sweden (Lundberg and Gustafsson 1995), northern Poland (Burakowski et al. 1978), Estonia, Denmark (Polilov 2009).

[*rosskotheni* **Sundt, 1971 =** *fraterna* Johnson, 1975]. Recently found in Latvia (Telnov et al. 2007), known in southern Sweden (Lundberg and Gustafsson 1995), Denmark (Polilov 2009).

[*rugulosa* **Rosskothen, 1935**]. Recently found in Latvia (Telnov et al. 2008), known in southern Sweden (Lundberg and Gustafsson 1995), Estonia, Denmark, Poland (Polilov 2009).

*sericans* **(Heer, 1841) =** *picicornis* auct. nec (Mannerheim, 1843) = *chevrolatii* (Allibert, 1844). Tamutis and Ferenca 2006; Ferenca et al. 2006.

[*silvatica* **Rosskothen, 1935**]. Recently found in Latvia (Telnov et al. 2008, 2010), known in southern Sweden (Lundberg and Gustafsson 1995), northeastern Poland (Burakowski et al. 1978), Denmark (Polilov 2009).

*sitkaensis* **(Motschulsky, 1845) =** *fratercula* auct. nec (Matthews, 1878). Tamutis and Ferenca 2006; Ferenca et al. 2006; Ferenca 2006a; Alekseev 2008a.

*strandi* **Sundt, 1958**. Ivinskis et al. 2009.

*thoracica* **(Waltl, 1838)**. Polilov 2009.

*Actinopteryx* **Matthews, 1872**.

[*fucicola* **(Allibert, 1844)**]. Known in southern Sweden (Lundberg and Gustafsson 1995), Denmark (Polilov 2009).

**AGYRTIDAE Thomson, 1859**.

**Agyrtinae Thomson, 1859**.

*Agyrtes* **Frölich, 1799)**.

[*bicolor* **Laporte, 1840**]. Known in southern Sweden (Lundberg and Gustafsson 1995), Poland (Růžička 2009).

[*castaneus* **(Fabricius, 1792)**]. Known in northern Poland (Burakowski et al. 1978).

**Pterolomatinae Thomson, 1862**.

*Pteroloma* **Gyllenhal, 1827**.

[*forsstromii* **(Gyllenhal, 1810)**]. Known in southern Sweden (Lundberg and Gustafsson 1995), Poland (Burakowski et al. 1978), Estonia (Růžička 2009).

**LEIODIDAE Fleming, 1821**.

**Leiodinae Fleming, 1821**.

**Sogdini Lopatin, 1961**.

*Triarthron* **Märkel, 1840**.

[*maerkelii* **Märkel, 1840**]. Known in southern Sweden (Lundberg and Gustafsson 1995), northern Poland (Burakowski et al. 1978), Denmark (Alonso-Zarazaga 2009a).

*Sogda* **Lopatin, 1961 =** *Trichohydnobius* Vogt, 1961.

*ciliaris **(Thomson, 1874) =** *hyperborea* (Strand, 1943). # 37. Perreau 2004.

*suturalis* **(Zetterstedt, 1828) =** *perrisii* (Fairmaire, 1855). Mazurowa and Mazur 1939; Pileckis 1960, 1976a; Silfverberg 1992, 2004; Pileckis and Monsevičius 1995 (*Hydnobius*); Perreau 2004; Alonso-Zarazaga 2009a.

*Hydnobius* **W.L.E. Schmidt, 1841**.

[*latifrons* **(Curtis, 1840) =** *strigosus* W.L.E. Schmidt, 1841]. Known in southern Sweden (Lundberg and Gustafsson 1995), northern Poland (Burakowski et al. 1978).

[*multistriatus* **(Gyllenhal, 1813)**]. Known in southern Sweden (Lundberg and Gustafsson 1995), northern Poland (Burakowski et al. 1978), Denmark, (Alonso-Zarazaga 2009a).

[*punctatus* **(Sturm, 1807)**]. Known in Latvia (Telnov 2004), northern Poland (Burakowski et al. 1978), Denmark (Alonso-Zarazaga 2009a).

[*spinipes* **(Gyllenhal, 1813)**]. Known in southern Sweden (Lundberg and Gustafsson 1995), Poland (Burakowski et al. 1978), Estonia (Alonso-Zarazaga 2009a).

**Leiodini Fleming, 1821**.

*Leiodes* **Latreille, 1796**.

*badius* **(Sturm, 1807)**. Pileckis 1976a; Silfverberg 1992, 2004; Pileckis and Monsevičius 1995; Perreau 2004; Alonso-Zarazaga 2009a.

*bicolor* **(W.L.E. Schmidt, 1841)**. Perkovskij and Monsevičius 1988; Silfverberg 1992, 2004; Pileckis and Monsevičius 1995; Monsevičius 1997; Ferenca 2003.

[*brunneus* **(Sturm, 1807)**]. Known in northern Poland (Burakowski et al. 1978).

*calcarata* **(Erichson, 1845)** **=** *polita* (Marsham, 1802).Roubal 1910; Pileckis 1960, 1976a; Bercio and Folwaczny 1979; Silfverberg 1992, 2004; Pileckis and Monsevičius 1995; Perreau 2004; Žiogas and Zolubas 2005; Alonso-Zarazaga 2009a.

*ciliaris* **(W.L.E. Schmidt, 1841)**. Perkovskij and Monsevičius 1988; Silfverberg 1992, 2004; Pileckis and Monsevičius 1995; Monsevičius 1997; Ferenca 2003; Ferenca et al. 2006.

[*cinnamomeus* **(Panzer, 1793)**]. Known in Sweden, Denmark (Lundberg and Gustafsson 1995; Alonso-Zarazaga 2009a), northern Poland (Burakowski et al. 1978).

*dubia* **(Kugellann, 1794)**. Pileckis 1976a; Silfverberg 1992, 2004; Gaidienė 1993; Pileckis and Monsevičius 1995; Perreau 2004.

*ferrugineus* **(Fabricius, 1787) =** *ovalis* (W.L.E. Schmidt, 1841). Perreau 2004.

*flavescens* **(W.L.E. Schmidt, 1841)**. Pileckis 1976a; Bercio and Folwaczny 1979; Silfverberg 1992, 2004; Pileckis and Monsevičius 1995; Perreau 2004; Alonso-Zarazaga 2009a.

[*fractus* **(Seidlitz, 1874) =** *rhaetica* auct. nec (Erichson, 1845)]. Known in Latvia (Telnov 2004), southern Sweden (Lundberg and Gustafsson 1995), Estonia (Alonso-Zarazaga 2009a).

[*furvus* **(Erichson, 1845)**]. Known in southern Sweden (Lundberg and Gustafsson 1995), Denmark, Estonia (Alonso-Zarazaga 2009a), northern Poland (Burakowski et al. 1978).

[*gallicus* **(Reitter, 1884)**]. Known in southern Sweden (Lundberg and Gustafsson 1995), Denmark (Alonso-Zarazaga 2009a).

*gyllenhalii* **Stephens, 1829 =** *parvula* (C.R. Sahlberg, 1833). Bercio and Folwaczny 1979; Silfverberg 1992, 2004; Perreau 2004; Alonso-Zarazaga 2009a.

[*litura* **Stephens, 1832**]. Known in southern Sweden (Lundberg and Gustafsson 1995), Denmark (Alonso-Zarazaga 2009a).

[*longipes* **(W.L.E. Schmidt, 1841) =** *curta* (Fairmaire & Laboulbène, 1854)]. Known in southern Sweden (Lundberg and Gustafsson 1995), Denmark, Poland (Alonso-Zarazaga 2009a).

[*lucens* **(Fairmaire, 1855)**]. Known in southern Sweden (Lundberg and Gustafsson 1995), southeastern Belarus (Alexandrovitch et al. 1996), Poland (Burakowski et al. 1978).

*obesus* **(W.L.E. Schmidt, 1841)**. Mazurowa and Mazur 1939; Pileckis 1960, 1976a; Silfverberg 1992, 2004; Pileckis and Monsevičius 1995; Perreau 2004; Alonso-Zarazaga 2009a.

*oblongus* **(Erichson, 1845)**. Karalius and Monsevičius 1992; Pileckis and Monsevičius 1995; Silfverberg 1996, 2004; Monsevičius 1997; Ferenca 2003.

[*pallens* **(Sturm, 1807)**]. Known in Latvia (Telnov et al. 2006), Sweden (Alonso-Zarazaga 2009a), northern Poland (Burakowski et al. 1978).

*piceus* **(Panzer, 1797)**. Pileckis 1976a; Silfverberg 1992, 2004; Gaidienė 1993; Pileckis and Monsevičius 1995; Perreau 2004; Alonso-Zarazaga 2009a.

[*rubiginosus* **(W.L.E. Schmidt, 1841)**]. Known in southern Sweden (Lundberg and Gustafsson 1995), Denmark (Alonso-Zarazaga 2009a), northern Poland (Burakowski et al. 1978).

[*ruficollis* **(J.R. Sahlberg, 1898) =** *nigrita* auct. nec (W.L.E. Schmidt, 1841)]. Known in southern Sweden (Lundberg and Gustafsson 1995), Denmark, Estonia, Poland (Alonso-Zarazaga 2009a).

*rufipennis* **(Paykull, 1798)**. Tamutis 2003; Žiogas and Zolubas 2005.

[*rugosus* **Stephens, 1829**]. Known in Latvia (Telnov 2004), southern Sweden (Lundberg and Gustafsson 1995), northwestern Belarus (Alexandrovitch et al. 1996), Denmark, Estonia (Alonso-Zarazaga 2009a), northern Poland (Burakowski et al. 1978).

*silesiacus* **(Kraatz, 1852)**. Pileckis and Monsevičius 1995; Silfverberg 2004.

[*triepkei* **(W.L.E. Schmidt, 1841)**]. Known in Latvia (Telnov 2004), southern Sweden (Lundberg and Gustafsson 1995), northwestern Belarus (Alexandrovitch et al. 1996), Denmark (Alonso-Zarazaga 2009a), northern Poland (Burakowski et al. 1978).

*Liocyrtusa* **Daffner, 1982**.

*minuta* **(Ahrens, 1812)**.Pileckis 1976a; Silfverberg 1992, 2004; Pileckis and Monsevičius 1995 (*Cyrtusa*); Perreau 2004; Alonso-Zarazaga 2009a.

[*vittata* **(Curtis, 1840) =** *pauxilla* (W.L.E. Schmidt, 1841)]. Known in southern Sweden (Lundberg and Gustafsson 1995), northwestern Belarus (Alexandrovitch et al. 1996), Denmark (Alonso-Zarazaga 2009a), northern Poland (Burakowski et al. 1978).

*Cyrtusa* **Erichson, 1842**.

*subtestacea* **(Gyllenhal, 1813)**. Silfverberg 1992, 2004; Pileckis and Monsevičius 1995; Perreau 2004; Alonso-Zarazaga 2009a.

**Pseudoliodini Portevin, 1926**.

*Colenis* **Erichson, 1842**.

*immunda* **(Sturm, 1807)**. Monsevičius and Pankevičius 2001; Ferenca 2003; Šablevičius 2004.

*Agaricophagus* **Schmidt, 1841**.

*cephalotes* **W.L.E. Schmidt, 1841**. Perkovskij and Monsevičius 1988; Silfverberg 1992, 2004; Pileckis and Monsevičius 1995; Monsevičius 1997; Perreau 2004; Alonso-Zarazaga 2009a.

**Agathidiini Westwood, 1838**.

*Anisotoma* **Panzer, 1797**.

*axillaris* **Gyllenhal, 1810**. Pileckis 1976a; Silfverberg 1992, 2004; Gaidienė 1993; Pileckis and Monsevičius 1995; Perreau 2004; Alonso-Zarazaga 2009a.

*castanea* **(Herbst, 1792)**. Slavinskas 1982; Perkovskij and Monsevičius 1988; Silfverberg 1992, 2004; Gaidienė 1993; Pileckis and Monsevičius 1995; Monsevičius 1997; Šablevičius 2003a; Perreau 2004; Vaivilavičius 2008; Alonso-Zarazaga 2009a; Ostrauskas and Ferenca 2010.

*glabra* **(Kugellan, 1794)**. Mazurowa and Mazur 1939; Pileckis 1960, 1976a; Bercio and Folwaczny 1979; Perkovskij and Monsevičius 1988; Silfverberg 1992, 2004; Gaidienė 1993; Pileckis and Monsevičius 1995; Monsevičius 1997; Barševskis 2001a; Perreau 2004; Alonso-Zarazaga 2009a.

*humeralis* **(Fabricius, 1792)**. Pileckis 1960, 1976a; Bercio and Folwaczny 1979; Perkovskij and Monsevičius 1988; Silfverberg 1992, 2004; Gaidienė 1993; Pileckis and Monsevičius 1995; Monsevičius 1997; Šablevičius 2000b; Barševskis 2001a; Tamutis and Zolubas 2001; Perreau 2004; Vaivilavičius 2008; Alonso-Zarazaga 2009a; Ostrauskas and Ferenca 2010.

*orbicularis* **(Herbst, 1792)**. Bercio and Folwaczny 1979; Perkovskij and Monsevičius 1988; Silfverberg 1992, 2004; Gaidienė 1993; Pileckis and Monsevičius 1995; Monsevičius 1997; Perreau 2004; Alonso-Zarazaga 2009a.

*Liodopria* **Reitter, 1909**.

*serricornis* **(Gyllenhal, 1813)**. Łomnicki 1913; Pileckis 1968b, 1976a; Pileckis and Monsevičius 1995; Silfverberg 1992, 2004; Perreau 2004; Alonso-Zarazaga 2009a.

*Amphicyllis* **Erichson 1845**.

*globiformis* **(C.R. Sahlberg, 1833)**. Perkovskij and Monsevičius 1988; Silfverberg 1992, 2004; Gaidienė 1993; Pileckis and Monsevičius 1995; Monsevičius 1997; Perreau 2004; Alonso-Zarazaga 2007a.

*globus* **(Fabricius, 1792)**. Bercio and Folwaczny 1979; Perkovskij and Monsevičius 1988; Silfverberg 1992, 2004; Pileckis and Monsevičius 1995; Monsevičius 1997; Perreau 2004; Žiogas and Zolubas 2005; Vaivilavičius 2008; Alonso-Zarazaga 2009a.

*Agathidium* **Panzer, 1797**.

*atrum* **(Paykull, 1798)**. Pileckis and Monsevičius 1982, 1995; Silfverberg 1992, 2004; Monsevičius 1997; Šablevičius 2000b; Perreau 2004; Alonso-Zarazaga 2009a; Dapkus and Tamutis 2007, 2008a.

*badium* **Erichson, 1845**. Perkovskij and Monsevičius 1988; Silfverberg 1992, 2004; Pileckis and Monsevičius 1995; Monsevičius 1997; Barševskis 2001a; Šablevičius 2001; Perreau 2004; Alonso-Zarazaga 2009a.

*confusum* **Brisout, 1863**. Silfverberg 1992, 2004; Pileckis and Monsevičius 1995; Perreau 2004; Žiogas and Zolubas 2005; Alonso-Zarazaga 2009a.

*convexum* **Sharp, 1866 =** *piceum* Erichson, 1845 nec Mannerheim, 1844. Pileckis 1976a; Silfverberg 1992, 2004; Pileckis and Monsevičius 1995; Perreau 2004; Alonso-Zarazaga 2009a.

[*discoideum* **Erichson, 1845**]. Known in Estonia (Süda 2009), Poland (Burakowski et al. 1978), Sweden (Lundberg and Gustafsson 1995), Belarus (Alexandrovitch et al. 1996).

[*haemorrhoum* **Erichson, 1845**]. Known in Latvia (Telnov 2004), southern Sweden (Lundberg and Gustafsson 1995), Denmark, Poland (Alonso-Zarazaga 2009a).

*laevigatum* **Erichson, 1845**. Bercio and Folwaczny 1979; Ivinskis et al. 1984; Perkovskij and Monsevičius 1988; Silfverberg 1992, 2004; Pileckis and Monsevičius 1995; Monsevičius 1997; Perreau 2004; Alonso-Zarazaga 2009a.

[*mandibulare* **Sturm, 1807**]. Known in Latvia (Telnov 2004), southern Sweden (Lundberg and Gustafsson 1995), Belarus (Alexandrovitch et al. 1996), Denmark, Estonia (Alonso-Zarazaga 2009a), northern Poland (Burakowski et al. 1978).

*marginatum* **Sturm, 1807**. Pileckis and Monsevičius 1982, 1995; Silfverberg 1992, 2004; Monsevičius 1997; Perreau 2004; Alonso-Zarazaga 2009a.

[*nigrinum* **Sturm, 1807**]. Known in southern Sweden (Lundberg and Gustafsson 1995), Estonia, Denmark (Alonso-Zarazaga 2009a), northern Poland (Burakowski et al. 1978).

*nigripenne* **(Fabricius, 1792)**. Lentz 1879; Pileckis 1968b, 1976a; Bercio and Folwaczny 1979; Silfverberg 1992, 2004; Gaidienė 1993; Pileckis and Monsevičius 1995; Šablevičius 2000a, 2001, 2003a, b; Perreau 2004; Alonso-Zarazaga 2009a.

*pisanum* **Brisout, 1872 =** *bicolor* J.R. Sahlberg, 1881. Perkovskij and Monsevičius 1988; Silfverberg 1992, 2004; Pileckis and Monsevičius 1995; Monsevičius 1997; Perreau 2004; Alonso-Zarazaga 2009a.

*plagiatum* **(Gyllenhal, 1810)**. Alonso-Zarazaga 2009a.

*pulchellum* **Wankowicz, 1869**. Hlisnikovský 1964; Pileckis 1976a; Bercio and Folwaczny 1979; Silfverberg 1992, 2004; Pileckis and Monsevičius 1995; Alonso-Zarazaga 2009a.

*rotundatum* **(Gyllenhal, 1827)=** *sphaerulum* Reitter, 1898. Pileckis 1976a; Silfverberg 1992, 2004; Pileckis and Monsevičius 1995; Perreau 2004; Alonso-Zarazaga 2009a; Ivinskis et al. 2009.

*seminulum* **(Linnaeus, 1758)**. Perkovskij and Monsevičius 1988; Silfverberg 1992, 2004; Gaidienė 1993; Pileckis and Monsevičius 1995; Monsevičius 1997; Tamutis and Zolubas 2001; Šablevičius 2000a, 2001, 2003a; Perreau 2004; Vaivilavičius 2008; Alonso-Zarazaga 2009a; Ostrauskas and Ferenca 2010.

*varians* **Beck, 1817**. Perkovskij and Monsevičius 1988; Silfverberg 1992, 2004; Pileckis and Monsevičius 1995; Monsevičius 1997; Perreau 2004; Alonso-Zarazaga 2009a.

**Coloninae Horn, 1880 (1859)**.(Colonidae)

*Colon* **Herbst, 1797**.

[*affine* **Surm, 1839**]. Known in northern Poland (Burakowski et al. 1978).

[*angulare* **Erichson, 1837**]. Known in southern Sweden (Lundberg and Gustafsson 1995), northern Belarus (Alexandrovitch et al. 1996), Denmark (Alonso-Zarazaga 2009a), northern Poland (Burakowski et al. 1978).

*appendiculatum* **(C.R. Sahlberg, 1822) =** *denticulatum* Kraatz, 1850. Monsevičius and Pankevičius 2001.

[*armipes* **Kraatz, 1854**]. Known in northern Poland (Burakowski et al. 1978).

[*barnevillei* **Kraatz, 1858** *= dubiosum* Ihssen, 1951]. Known in southern Sweden (Lundberg and Gustafsson 1995), Denmark (Alonso-Zarazaga 2009a), Poland (Burakowski et al. 1978).

[*bidentatum* **(C.R. Sahlberg, 1822)**] Known in Latvia (Telnov 2004), southern Sweden (Lundberg and Gustafsson 1995), Estonia, Denmark (Alonso-Zarazaga 2009a), Poland (Burakowski et al. 1978).

*brunneum* **(Latreille, 1807)**. Roubal 1910; Pileckis 1960, 1976a; Silfverberg 1992, 2004; Pileckis and Monsevičius 1995; Perreau 2004; Alonso-Zarazaga 2009a.

*calcaratum* **Erichson, 1837**. Pileckis 1976a; Silfverberg 1992, 2004; Pileckis and Monsevičius 1995; Perreau 2004; Alonso-Zarazaga 2009a.

[*claviger* **Herbst, 1797**]. Known in northern Poland (Burakowski et al. 1978).

[*dentipes* **(C.R. Sahlberg, 1822)**]. Known in southern Sweden (Lundberg and Gustafsson 1995), Denmark (Alonso-Zarazaga 2009a), northeastern Poland (Burakowski et al. 1978).

*latum* **Kraatz, 1850**. Monsevičius 1999.

[*puncticolle* **Kraatz, 1850**]. Recently found in Latvia (Telnov et al. 2008), known in southern Sweden (Lundberg and Gustafsson 1995), Estonia, Denmark (Alonso-Zarazaga 2009a), northern Poland (Burakowski et al. 1978).

[*rufescens* **Kraatz, 1850**]. Known in Latvia (Telnov 2004), southern Sweden (Lundberg and Gustafsson 1995), Denmark, Poland (Alonso-Zarazaga 2009a).

[*serripes* **(C.R. Sahlberg, 1822)**]. Known in Latvia (Telnov 2004), southern Sweden (Lundberg and Gustafsson 1995), Belarus (Alexandrovitch et al. 1996), Denmark (Alonso-Zarazaga 2009a), northern Poland (Burakowski et al. 1978).

*viennense* **Herbst, 1797**. Perkovskij and Monsevičius 1988; Silfverberg 1992, 2004; Pileckis and Monsevičius 1995; Monsevičius 1997; Perreau 2004; Alonso-Zarazaga 2009a.

**Cholevinae Kirby, 1837**.

**Ptomaphagini Jeannel, 1911**.

*Ptomaphagus* **Illiger, 1798**.(Catopidae)

*sericatus medius* **(Rey, 1889)**.# 38. Pileckis 1960, 1976a; Silfverberg 1992, 2004; Pileckis and Monsevičius 1995; Perreau 2004; Vorst et al. 2007; Ivinskis et al. 2009; Ostrauskas and Ferenca 2010.

*subvillosus* **(Goeze, 1777)**. Pileckis 1960, 1976a; Perkovskij and Monsevičius 1988; Silfverberg 1992, 2004; Pileckis and Monsevičius 1995; Monsevičius 1997; Perreau 2004.

*varicornis* **(Rosenhauer, 1847)**. Perkovskij and Monsevičius 1988; Silfverberg 1992, 2004; Pileckis and Monsevičius 1995; Monsevičius 1997; Perreau 2004.

**Anemadini Hatch, 1928**.

*Nemadus* **Thomson, 1867**. (Catopidae)

*colonoides* **(Kraatz, 1851)**. Perkovskij and Monsevičius 1988; Silfverberg 1992, 2004; Pileckis and Monsevičius 1995; Monsevičius 1997; Perreau 2004.

**Cholevini Kirby, 1937**.

*Nargus* **Thomson, 1867**.

*anisotomoides* **(Spence, 1815)**. Tamutis et al. 2008.

*velox* **(Spence, 1815)**. Tamutis et al. 2008.

[*wilkini* **(Spence, 1815)**]. Known in Latvia (Telnov 2004), southern Sweden (Lundberg and Gustafsson 1995), Poland (Szymczakowski 1961; Burakowski et al. 1978), Denmark, (Alonso-Zarazaga 2009a).

*Choleva* **Latreille, 1796**. (Catopidae)

*agilis* **(Illiger, 1798)**. Perkovskij and Monsevičius 1988; Silfverberg 1992, 2004; Pileckis and Monsevičius 1995; Monsevičius 1997, 1999.

*angustata* **(Fabricius, 1781)**. Pileckis 1976a; Silfverberg 1992, 2004; Pileckis and Monsevičius 1995; Perreau 2004.

*cisteloides* **(Frölich, 1799)**. Pileckis 1962, 1963b; Silfverberg 1992, 2004; Pileckis and Monsevičius 1995.

*elongata* **(Paykull, 1798)**. Monsevičius 1999; Perreau 2004; Alonso-Zarazaga 2009a.

[*fagniezi* **Jeannel, 1922**]. Known in southern Sweden (Lundberg and Gustafsson 1995), Poland (Szymczakowski 1961; Burakowski et al. 1978), Denmark (Silfverberg 2004).

[*glauca* **Britten, 1918**]. Known in southern Sweden (Lundberg and Gustafsson 1995), Poland (Szymczakowski 1961; Burakowski et al. 1978), western Belarus (Alexandrovitch et al. 1996), Estonia (Silfverberg 2004), Denmark (Alonso-Zarazaga 2009a).

[*jeanneli* **Britten, 1922**]. Known in southern Sweden (Lundberg and Gustafsson 1995), Poland (Szymczakowski 1961; Burakowski et al. 1978), Denmark (Silfverberg 1992, 2004; Alonso-Zarazaga 2009a).

[*lederiana* **Reitter, 1901 =** *aquilonia* Krogerus, 1926]. Known in southern Sweden (Lundberg and Gustafsson 1995), Estonia (Silfverberg 2004), Poland (Alonso-Zarazaga 2009a).

[*nivalis* **(Kraatz, 1856)**]. Known in Latvia (Telnov 2004), Poland (Szymczakowski 1961; Burakowski et al. 1978; Alonso-Zarazaga 2009a).

*oblonga* **Latreille, 1807**. Perkovskij and Monsevičius 1988; Silfverberg 1992, 2004; Pileckis and Monsevičius 1995; Monsevičius 1999; Ferenca 2004; Perreau 2004.

*paskoviensis* **Reitter, 1913**. Tamutis 2003.

[*reitteri* **Petri, 1915**]. Known in southern Sweden (Lundberg and Gustafsson 1995), Poland (Szymczakowski 1961; Burakowski et al. 1978), Denmark (Alonso-Zarazaga 2009a).

*spadicea* **(Sturm, 1839)**. Ferenca 2003.

*spinipennis* **Reitter, 1890**. Pileckis 1976a; Pileckis and Monsevičius 1995; Monsevičius 1997.

*sturmii* **Brisout, 1863**. Pileckis 1962, 1963b; Monsevičius 1999.

*Dreposcia* **Jeannel, 1922**.(Catopidae)

*umbrina* **(Erichson, 1837)**. Szymczakowski 1961; Pileckis 1968b, 1976a; Silfverberg 1992, 2004; Pileckis and Monsevičius 1995; Perreau 2004; Alonso-Zarazaga 2009a.

*Sciodrepoides* **Hatch, 1933 =** *Sciodrepa* auct. nec Thomson, 1895. (Catopidae)

[*alpestris* **Jeannel, 1934**]. Known in southwestern Belarus (Alexandrovitch et al. 1996), Poland (Szymczakowski 1961; Burakowski et al. 1978).

*fumatus* **(Spence, 1815)**. Pileckis 1976a; Bercio and Folwaczny 1979; Silfverberg 1992, 2004; Pileckis and Monsevičius 1995; Monsevičius 1997; Perreau 2004.

*watsoni* **(Spence, 1815)**. Pileckis 1976a; Silfverberg 1992, 2004; Pileckis and Monsevičius 1995; Monsevičius 1997; Perreau 2004; Dapkus and Tamutis 2008a; Ivinskis et al. 2008, 2009; Alonso-Zarazaga 2009a; Ostrauskas and Ferenca 2010.

*Catops* **Paykull, 1798**.(Catopidae)

[*chrysomeloides* **(Panzer, 1798)**]. Known in southern Sweden (Lundberg and Gustafsson 1995), northern Poland (Szymczakowski 1961; Burakowski et al. 1978), Denmark (Silfverberg 1992, 2004; Alonso-Zarazaga 2009a).

*coracinus* **Kellner, 1846**. Perkovskij and Monsevičius 1988; Silfverberg 1992, 2004; Pileckis and Monsevičius 1995; Monsevičius 1997; Perreau 2004; Vaivilavičius 2008; Ostrauskas and Ferenca 2010.

*fuliginosus* **Erichson, 1837**. Silfverberg 1992, 2004; Pileckis and Monsevičius 1995.

*fuscus* **(Panzer, 1794)**. Pileckis 1962, 1963b, 1976a; Silfverberg 1992, 2004; Pileckis and Monsevičius 1995; Perreau 2004; Ostrauskas and Ferenca 2010.

[*grandicollis* **Erichson, 1837**]. Known in Latvia (Telnov 2004), Poland (Szymczakowski 1961; Burakowski et al. 1978), Denmark, Sweden (Alonso-Zarazaga 2009a).

*kirbyi* **(Spence, 1815)**. Pileckis 1976a; Silfverberg 1992, 2004; Pileckis and Monsevičius 1995; Perreau 2004.

*morio* **(Fabricius, 1787)**. Pileckis 1976a; Silfverberg 1992, 2004; Pileckis and Monsevičius 1995; Monsevičius 1997; Perreau 2004; Ivinskis et al. 2009; Ostrauskas and Ferenca 2010.

[*neglectus* **Kraatz, 1852**]. Known in southern Sweden (Lundberg and Gustafsson 1995), Poland (Szymczakowski 1961; Burakowski et al. 1978), Denmark (Silfverberg 1992, 2004; Alonso-Zarazaga 2009a).

*nigricans* **(Spence, 1815)**. Perkovskij and Monsevičius 1988; Silfverberg 1992, 2004; Pileckis and Monsevičius 1995; Monsevičius 1997; Perreau 2004.

*nigriclavis* **Gerhardt, 1900 =** *dohrni* Reitter, 1913. Monsevičius 1999.

*nigrita* **Erichson, 1837**. Perkovskij and Monsevičius 1988; Silfverberg 1992, 2004; Pileckis and Monsevičius 1995; Monsevičius 1997; Perreau 2004; Vaivilavičius 2008.

*picipes* **(Fabricius, 1787)**. Szymczakowski 1961; Pileckis 1968b, 1976a; Silfverberg 1992, 2004; Pileckis and Monsevičius 1995; Perreau 2004.

*subfuscus* **Kellner, 1846**. Perkovskij and Monsevičius 1988; Silfverberg 1992, 2004; Pileckis and Monsevičius 1995; Monsevičius 1997; Perreau 2004.

*tristis* **(Panzer, 1794)**. Perkovskij and Monsevičius 1988; Silfverberg 1992, 2004; Pileckis and Monsevičius 1995; Monsevičius 1997; Perreau 2004.

*westi* **Krogerus, 1931**. Pileckis 1963; Perkovskij and Monsevičius 1988; Silfverberg 1992, 2004; Pileckis and Monsevičius 1995; Monsevičius 1997; Perreau 2004.

**Platypsyllinae Ritsema, 1869**.

*Leptinus* **O.F. Müller, 1817**.

[*testaceus* **O.F. Müller, 1817**]. Known in southern Sweden (Lundberg and Gustafsson 1995), southwestern Belarus (Alexandrovitch et al. 1996), Kaliningrad region (Alekseev and Bukejs 2010), northern Poland (Szymczakowski 1961; Burakowski et al. 1978), Denmark (Silfverberg 1992, 2004; Alonso-Zarazaga 2009a).

*Platypsyllus* **Ritsema, 1869**.

*castoris* **Ritsema, 1869**. Tamutis 2003.

**Silphidae Latreille, 1806**.

**Silphinae Latreille, 1806**.

*Necrodes* **Leach, 1815**.

*littoralis* **(Linnaeus, 1758)**. Heyden 1903; Kopyłówna 1935; Mazurowa and Mazur 1939; Pileckis 1960, 1976a; Strazdienė 1976; Silfverberg 1992, 2004; Gaidienė 1993; Pileckis and Monsevičius 1995; Monsevičius 1997; Gliaudys 2001; Alekseev 2003; Růžička and Schneider 2004; Ferenca 2006b; Růžička 2009; Šablevičius 2011.

*Thanatophilus* **Leach, 1815**.

*dispar* **(Herbst, 1793)**. Kopyłówna 1935; Pileckis 1960, 1973, 1976a; Silfverberg 1992, 2004; Pileckis and Monsevičius 1995; Růžička and Schneider 2004; Ferenca 2006b; Růžička 2009.

*rugosus* **(Linnaeus, 1758)**. Kopyłówna 1935; Pileckis 1960, 1973, 1976a; Lešinskas and Pileckis 1967; Silfverberg 1992, 2004; Gaidienė 1993; Pileckis and Monsevičius 1995; Monsevičius 1997; Šablevičius 2000b; Růžička and Schneider 2004; Ferenca 2006b; Tamutis et al. 2006, 2007; Růžička 2009.

*sinuatus* **(Fabricius, 1775)**. Kopyłówna 1935; Pileckis 1960, 1973, 1976a; Suždelis 1962; Pileckis and Šaluchaitė 1993; Silfverberg 1992, 2004; Gaidienė 1993; Pileckis and Monsevičius 1995; Monsevičius 1997; Tamutis and Zolubas 2001; Růžička and Schneider 2004; Ferenca 2006b; Tamutis et al. 2006, 2007; Růžička 2009.

*Oiceoptoma* **Leach, 1815**.

*thoracica* **(Linnaeus, 1758)**. Eichwald 1830; Heyden 1903; Kopyłówna 1935; Mazurowa and Mazur 1939; Lešinskas and Pileckis 1967; Zajančkauskas and Pileckis 1968; Pileckis 1960, 1973, 1976a; Suždelis 1962; Silfverberg 1992, 2004; Gaidienė 1993; Pileckis and Monsevičius 1995; Monsevičius 1997; Šablevičius 2000b, 2011; Gliaudys 2001; Růžička and Schneider 2004; Ferenca 2006b; Žiogas and Zolubas 2005; Růžička 2009.

*Aclypea* **Reitter, 1884 =** *Bolitophaga* Reitter, 1884.

*opaca* **(Linnaeus, 1758)**. Heyden 1903; Ogijewicz 1932; Kopyłówna 1935; Pileckis 1960, 1963b, 1973, 1976a; Suždelis 1962; Lešinskas and Pileckis 1967; Pileckis and Vengeliauskaitė 1977, 1996; Pileckis et al. 1983, 1994b; Silfverberg 1992, 2004; Gaidienė 1993; Pileckis and Monsevičius 1995; Gliaudys 2001; Šurkus and Gaurilčikienė 2002; Růžička and Schneider 2004; Růžička 2009.

*undata* **(O.F. Müller, 1776)**. Lešinskas and Pileckis 1967; Pileckis 1973, 1976a; Silfverberg 1992, 2004; Pileckis et al. 1994b; Pileckis and Monsevičius 1995; Šurkus and Gaurilčikienė 2002; Růžička and Schneider 2004; Růžička 2009.

*Dendroxena* **Motschulsky, 1858** = *Xylodrepa* Thomson, 1859.

RDB*quarimaculata* **(Scopoli, 1772) =** *quadripunctata* (Linnaeus, 1761) nec (Linnaeus, 1758). Lešinskas and Pileckis 1967; Pileckis 1968a, 1973, 1976a, b, 1979, 1982; Balevičius 1992; Silfverberg 1992, 2004; Gaidienė 1993; Pileckis and Monsevičius 1995; Ivinskis et al. 1996b, 2004b, 2006, 2007, 2009; Ostrauskas 2000; Jonaitis et al. 2000; Ferenca 2004; Růžička and Schneider 2004; Tamutis 2005b; Rašomavičius 2007; Vaivilavičius 2008; Dapkus and Tamutis 2008b; Růžička 2009; Inokaitis 2009; Bačianskas 2009; Noreika 2009; Alekseev 2010b; Šablevičius 2011.

*Silpha* **Linnaeus, 1758**.

*carinata* **Herbst, 1783**. Eichwald 1830; Heyden 1903; Kopyłówna 1935; Pileckis 1960, 1973, 1976a; Suždelis 1962; Lešinskas and Pileckis 1967; Bercio and Folwaczny 1979; Silfverberg 1992, 2004; Gaidienė 1993; Pileckis and Monsevičius 1995; Gliaudys 2001; Růžička and Schneider 2004; Ferenca 2006b; Tamutis et al. 2007; Růžička 2009.

*obscura* **Linnaeus, 1758**. Eichwald 1830; Heyden 1903; Ogijewicz 1932; Kopyłówna 1935; Pileckis 1960, 1973, 1976a; Suždelis 1962; Lešinskas and Pileckis 1967; Silfverberg 1992, 2004; Pileckis and Monsevičius 1995; Gliaudys 2001; Růžička and Schneider 2004; Žiogas and Zolubas 2005; Alekseev 2008a; Růžička 2009.

*tristis* **Illiger, 1798**. Lentz 1879; Kopyłówna 1935; Suždelis 1962; Pileckis 1968b, 1973, 1976a; Bercio and Folwaczny 1979; Silfverberg 1992, 2004; Gaidienė 1993; Pileckis and Monsevičius 1995; Růžička and Schneider 2004; Tamutis et al. 2007; Růžička 2009.

*Ablattaria* **Reitter, 1884**.

[*laevigata* **(Fabricius, 1775)**]. Known in Estonia (Lundberg and Gustafsson 1995), Poland (Mroczkowski 1955; Burakowski et al. 1978), Belarus (Růžička 2009).

*Phosphuga* **Leach, 1817**.

*atrata* **(Linnaeus, 1758)**. Eichwald 1830; Heyden 1903; Roubal 1910; Kopyłówna 1935; Pileckis 1960, 1973, 1976a; Suždelis 1962; Lešinskas and Pileckis 1967; Silfverberg 1992, 2004; Gaidienė 1993; Pileckis and Monsevičius 1995; Monsevičius 1997; Šablevičius 2000b; Gliaudys 2001; Růžička and Schneider 2004; Ferenca 2006b; Vaivilavičius 2008; Růžička 2009.

**Nicrophorinae Kirby, 1837**.

*Nicrophorus* **Fabricius, 1775**.

[*antennatus* **(Reitter, 1855)**]. Known in northern and northwestern Belarus (Alexandrovitch et al. 1996), Poland (Růžička 2009).

*fossor* **Erichson, 1837 =** *interruptus* Stephens, 1830, nec Brullé, 1822. Kopyłówna 1935; Pileckis 1963, 1973, 1976a; Silfverberg 1992, 2004; Pileckis and Monsevičius 1995; Monsevičius 1997; Růžička and Schneider 2004; Ivinskis et al. 2008; Růžička 2009.

[*germanicus* **(Linnaeus, 1758)**]. Known in Latvia (Telnov 2004), Denmark, southern Sweden (Lundberg and Gustafsson 1995), Poland (Mroczkowski 1955; Růžička 2009).

*humator* **(Gleditsch, 1767)**. Eichwald 1830; Kopyłówna 1935; Pileckis 1960, 1973, 1976a; Lešinskas and Pileckis 1967; Silfverberg 1992, 2004; Gaidienė 1993; Pileckis and Monsevičius 1995; Monsevičius 1997; Šablevičius 2000b, 2011; Gliaudys 2001; Tamutis and Zolubas 2001; Růžička and Schneider 2004; Ivinskis et al. 2008; Růžička 2009; Inokaitis 2009.

*investigator* **Zetterstedt, 1824**. Kopyłówna 1935; Mazurowa and Mazur 1939; Pileckis 1963b, 1973, 1976a; Silfverberg 1992, 2004; Gaidienė 1993; Pileckis and Monsevičius 1995; Šablevičius 2000b, 2011; Gliaudys 2001; Růžička and Schneider 2004; Žiogas and Zolubas 2005; Ferenca 2006b; Ivinskis et al. 2008; Růžička 2009.

*sepultor* **Charpentier,** 1825. Tamutis 1999; Silfverberg 2004; Růžička and Schneider 2004; Tamutis et al. 2006, 2007; Ivinskis et al. 2008, 2009; Růžička 2009.

*vespillo* **(Linnaeus, 1758)**. Heyden 1903; Kopyłówna 1935; Mazurowa and Mazur 1939; Pileckis 1960, 1973, 1976a; Zajančkauskas and Pileckis 1968; Pileckis and Šaluchaitė 1993; Silfverberg 1992, 2004; Gaidienė 1993; Pileckis and Monsevičius 1995; Monsevičius 1997; Šablevičius 2000b; Tamutis and Zolubas 2001; Růžička and Schneider 2004; Ferenca 2006b; Tamutis et al. 2007; Ivinskis et al. 2008; Růžička 2009.

*vespilloides* **Herbst, 1784**. Kopyłówna 1935; Pileckis 1960, 1973, 1976a; Zajančkauskas and Pileckis 1968; Silfverberg 1992, 2004; Gaidienė 1993; Pileckis and Monsevičius 1995; Monsevičius 1997; Šablevičius 2000b, 2011; Gliaudys 2001; Tamutis and Zolubas 2001; Růžička and Schneider 2004; Ferenca 2006b; Dapkus and Tamutis 2008a; Ivinskis et al. 2008; Růžička 2009.

*vestigator* **Herschel, 1807**. Kopyłówna 1935; Mazurowa and Mazur 1939; Pileckis 1960, 1973, 1976a; Silfverberg 1992, 2004; Gaidienė 1993; Pileckis and Monsevičius 1995; Růžička and Schneider 2004; Ferenca 2006b; Růžička 2009.

**STAPHYLINIDAE Latreille, 1802**.

**Omaliinae MacLeay, 1825**.

**Eusphalerini Hatch, 1957**.

*Eusphalerum* **Kraatz, 1857**.

*lapponicum* **(Mannerheim, 1830)**.Monsevičius 1987, 1988a, 1997; Silfverberg 1992, 2004; Pileckis and Monsevičius 1995; Smetana 2004a; Alonso-Zarazaga 2009a; Ivinskis et al. 2009.

[*limbatum* **(Erichson, 1840)**].Known in Estonia (Lundberg and Gustafsson 1995; Silfverberg 1992, 2004), Poland (Alonso-Zarazaga 2009a).

*longipenne* **(Erichson, 1839)**.Gaidienė 1993; Pileckis and Monsevičius 1995; Silfverberg 2004.

*luteum* **(Marsham, 1802)** = *ophthalmicum* (Paykull, 1800) nec (Scopoli, 1763). Gaidienė 1993; Silfverberg 2004.

*minutum* **(Fabricius, 1792)**.Pileckis 1960, 1976a; Monsevičius 1987, 1997; Silfverberg 1992, 2004; Gaidienė 1993; Pileckis and Monsevičius 1995; Smetana 2004a; Ferenca 2006b.

*primulae* **(Stephens, 1839)**.Pileckis 1960, 1976a; Gaidienė 1993; Silfverberg 1992, 2004; Pileckis and Monsevičius 1995; Smetana 2004a; Ferenca 2006b; Alonso-Zarazaga 2009a.

*sorbi* **(Gyllenhal, 1810)**.Tamutis 2003; Smetana 2004a; Alonso-Zarazaga 2009a.

[*sorbicola* **(Y. Kangas, 1941)**].Known in Estonia, central and northern Sweden, (Lundberg and Gustafsson 1995), Denmark (Silfverberg 2004; Alonso-Zarazaga 2009a).

*tenenbaumi* **(Bernhauer, 1932)** = *florale* auct. nec (Panzer, 1793). Gaidienė 1993; Silfverberg 2004; Ivinskis et al. 2009.

[*torquatum* **(Marsham, 1802)**].Known in Denmark, southern Sweden (Lundberg and Gustafsson 1995; Silfverberg 1992, 2004), Poland (Burakowski et al. 1979; Alonso-Zarazaga 2009a).

**Omaliini MacLeay, 1825**.

*Acrulia* **Thomson, 1858**.

*inflata* **(Gyllenhal, 1813)**. Smetana 2004a; Alonso-Zarazaga 2009a.

*Pycnoglypta* **Thomson, 1858**.

*lurida* **(Gyllenhal, 1813)**.Pileckis 1976a; Silfverberg 1992, 2004; Pileckis and Monsevičius 1995; Smetana 2004a; Alonso-Zarazaga 2009a.

*Acrolocha* **Thomson, 1858**.

[*minuta* **(Olivier, 1795)** = *striata* (Gravenhorst, 1802)].Known in Denmark, southern Sweden, Estonia (Lundberg and Gustafsson 1995; Silfverberg 1992, 2004), northern Poland (Burakowski et al. 1979).

[*pliginskii* **(Bernhauer, 1912)**].Known in Denmark, southern Sweden (Lundberg and Gustafsson 1995; Silfverberg 1992, 2004). 

*sulcula* **(Stephens, 1834)**.Gaidienė 1993; Tamutis 2003; Silfverberg 2004.

*Phyllodrepa* **Thomson, 1859**.# 93.

*floralis* **(Paykull, 1789)**.Pileckis 1976a; Silfverberg 1992, 2004; Gaidienė 1993; Pileckis and Monsevičius 1995 (*Hapalaraea*); Monsevičius 1997; Smetana 2004a; Alonso-Zarazaga 2009a; Ivinskis et al. 2009.

[*gracilicornis* **(Fairmaire & Laboulbène, 1856)**].Known in Denmark, southern Sweden (Lundberg and Gustafsson 1995; Silfverberg 1992, 2004), Poland (Alonso-Zarazaga 2009a).

*ioptera* **(Stephens, 1832)**.Monsevičius and Pankevičius 2001; Silfverberg 2004; Ivinskis et al. 2009.

[*linearis* **(Zetterstedt, 1828)**].Known in Latvia (Telnov 2004), throughout Sweden, Estonia (Lundberg and Gustafsson 1995), western Belarus (Alexandrovich et al. 1996), southern Poland (Burakowski et al. 1979).

[*melanocephala* **(Fabricius, 1787)**].Known in Latvia (Telnov. 2004), throughout Sweden, Estonia, Denmark (Lundberg and Gustafsson 1995), northwestern Belarus (Alexandrovich et al. 1996), throughout Poland (Burakowski et al. 1979).

*melis* **V. Hansen, 1940**.Monsevičius 1985, 1988a, 1997, 1999; Silfverberg 1992, 2004; Pileckis and Monsevičius 1995 (*Hapalaraea*); Alonso-Zarazaga 2009a.

*nigra* **(Gravenhorst, 1806)**.Pileckis 1976a; Monsevičius 1987, 1997; Silfverberg 1992, 2004; Pileckis and Monsevičius 1995 (*Hapalaraea*); Smetana 2004a; Alonso-Zarazaga 2009a.

*puberula* **Bernhauer, 1903**.Monsevičius 1985, 1997, 1999; Silfverberg 1992, 2004; Pileckis and Monsevičius 1995 (*Hapalaraea*); Smetana 2004a; Alonso-Zarazaga 2009a.

[*salicis* **(Gyllenhal, 1810)**].Known in Denmark, southern Sweden (Lundberg and Gustafsson 1995; Silfverberg 1992, 2004), northern Poland (Burakowski et al. 1979).

*vilis* **(Erichson, 1840)**.Ivinskis et al. 2009.

*Hapalaraea* **Thomson, 1858**.

[*pygmaea* **(Paykull, 1800)**]. Known in Latvia (Telnov 2004), Denmark, southern Sweden (Lundberg and Gustafsson 1995), Estonia (Silfverberg 2004), western Belarus (Alexandrovich et al. 1996), eastern Poland (Burakowski et al. 1979).

*Omalium* **Gravenhorst, 1802**.

[*allardii* **(Fairmaire & Brisout, 1859)**].Known in Denmark, southern Sweden (Lundberg and Gustafsson 1995; Silfverberg 1992, 2004).

*caesum* **Gravenhorst, 1806**.Silfverberg 1992, 2004; Pileckis and Monsevičius 1995; Monsevičius 1997; Tamutis et al. 2004; Smetana 2004a; Alonso-Zarazaga 2009a.

*excavatum* **Stephens, 1834**.Pileckis 1960, 1976a; Silfverberg 1992, 2004; Pileckis and Monsevičius 1995; Smetana 2004a; Ferenca 2006b; Alonso-Zarazaga 2009a.

[*exiguum* **Gyllenhal, 1810**].Known throughout Sweden, Denmark, Estonia (Lundberg and Gustafsson 1995), central Poland (Burakowski et al. 1979).

[*laeviusculum* **Gyllenhal, 1827**].Known in Denmark, southern Sweden (Lundberg and Gustafsson 1995; Silfverberg 1992, 2004).

[*laticolle* **Kraatz, 1858**].Known in Denmark, central and southern Sweden (Lundberg and Gustafsson 1995; Silfverberg 1992, 2004), Poland (Alonso-Zarazaga 2009a).

*littorale* **Kraatz, 1858**.Pileckis 1976a; Silfverberg 1992, 2004; Pileckis and Monsevičius 1995; Smetana 2004a; Alonso-Zarazaga 2009a.

*oxyacanthae* **Gravenhorst, 1806**.Monsevičius 1985; Silfverberg 1992, 2004; Pileckis and Monsevičius 1995; Smetana 2004a; Alonso-Zarazaga 2009a.

[*riparium* **Thomson, 1857**]. Known in Denmark, southern Sweden, Estonia (Lundberg and Gustafsson 1995; Silfverberg 1992, 2004).

*rivulare* **(Paykull, 1789)**.Pileckis 1976a; Monsevičius 1987, 1997; Silfverberg 1992, 2004; Gaidienė 1993; Pileckis and Monsevičius 1995; Smetana 2004a; Alonso-Zarazaga 2009a.

[*rugatum* **Rye, 1880**]. Known throughout Sweden, Denmark, Estonia (Lundberg and Gustafsson 1995), southern Poland (Burakowski et al. 1979).

*septentrionis* **Thomson, 1857**.Gaidienė 1993; Silfverberg 2004.

[*validum* **Kraatz, 1858**]. Known in southern Sweden, Estonia (Lundberg and Gustafsson 1995; Silfverberg 1992, 2004), Poland (Burakowski et al. 1979).

*Xylostiba* **Ganglbauer, 1895**.

*monilicornis* **(Gyllenhal, 1810)**.Monsevičius 1985, 1997; Pileckis and Monsevičius 1995 (*Phloenomus*); Silfverberg 1992, 2004; Smetana 2004a; Alonso-Zarazaga 2009a.

*Phloeostiba* **Thomson, 1858**.# 94.

*lapponica* **(Zetterstedt, 1838)**.Roubal 1910; Pileckis 1960, 1976a; Jakaitis and Valenta 1976; Monsevičius 1987, 1997; Silfverberg 1992, 2004; Pileckis and Monsevičius 1995 (*Phloenomus*); Smetana 2004a; Alonso-Zarazaga 2009a; Ivinskis et al. 2009.

*plana* **(Paykull, 1792)**. Pileckis 1976a; Silfverberg 1992, 2004; Pileckis and Monsevičius 1995 (*Phloenomus*); Smetana 2004a; Alonso-Zarazaga 2009a.

*Phloeonomus* **Heer, 1839**.

*minimus* **(Erichson, 1839)**.Ivinskis et al. 2009.

*punctipennis* **Thomson, 1867**.Monsevičius 1985, 1997; Silfverberg 1992, 2004; Pileckis and Monsevičius 1995; Smetana 2004a; Alonso-Zarazaga 2009a.

*pusillus* **(Gravenhorst, 1806)**.Jakaitis and Valenta 1976; Pileckis 1976a; Monsevičius 1987, 1997; Silfverberg 1992, 2004; Gaidienė 1993; Pileckis and Monsevičius 1995; Smetana 2004a; Alonso-Zarazaga 2009a.

[*sjoebergi* **Strand, 1937**]. Known in Latvia (Telnov 2004), Estonia, throughout the Sweden (Lundberg and Gustafsson 1995), Latvia (Telnov et al. 2005).

*Xylodromus* **Heer, 1839**.

[*affinis* **(Gerherdt, 1877)**].Known in Denmark, southern Sweden (Lundberg and Gustafsson 1995; Silfverberg 1992, 2004), southern Poland (Burakowski et al. 1979).

*brunnipennis* **(Stephens, 1832)** = *concinnus* (Marsham, 1802) nec (Gravenhorst, 1802).Pileckis 1976a; Silfverberg 1992, 2004; Pileckis and Monsevičius 1995; Smetana 2004a; Alonso-Zarazaga 2009a.

*depressus* **(Gravenhorst, 1802)**.Smetana 2004a; Alonso-Zarazaga 2009a.

[*testaceus* **(Erichson, 1840)**].Known in Denmark, southern Sweden (Lundberg and Gustafsson 1995), southern Belarus (Alexandrovich et al. 1996), northeastern Poland (Burakowski et al. 1979).

**Anthophagini Thomson, 1859**.

*Philorinum* **Kraatz, 1857**.

[*sordidum* **(Stephens, 1834)**].Known in Denmark (Silfverberg 1992, 2004; Alonso-Zarazaga 2009a).

*Orochares* **Kraatz, 1857**.

[*angustatus* **(Erichson, 1840)**].Known in Latvia (Telnov 2004), Denmark, southern Sweden (Lundberg and Gustafsson 1995), northern Poland (Burakowski et al. 1979).

*Phyllodrepoidea* **Ganglbauer, 1895**.

[*crenata* **Ganglbauer, 1895**]. Known in Sweden, Denmark (Lundberg and Gustafsson 1995), Poland (Burakowski et al. 1979).

*Deliphrum* **Erichson, 1839**.

*tectum* **(Paykull, 1789)**.Monsevičius 1985; Silfverberg 1992, 2004; Pileckis and Monsevičius 1995; Smetana 2004a; Alonso-Zarazaga 2009a.

*Anthobium* **Leach, 1819** = *Lathrimaeum* Erichson, 1839.

*atrocephalum* **(Gyllenhal, 1827)**.Bercio and Folwaczny 1979; Monsevičius 1986b, 1997; Silfverberg 1992, 2004; Pileckis and Monsevičius 1995; Smetana 2004a; Alonso-Zarazaga 2009a; Ivinskis et al. 2009.

[*fusculum* **(Erichson, 1839)**].Known in southern and central Sweden, Denmark (Lundberg and Gustafsson 1995), northern Poland (Burakowski et al. 1979).

[*melanocephalum* **(Illiger, 1794)**].Known in Latvia (Telnov 2004), southern and central Sweden, Estonia (Lundberg and Gustafsson 1995), throughout Poland (Burakowski et al. 1979).

[*unicolor* **(Marsham, 1802)**].Known in southern and central Sweden (Lundberg and Gustafsson 1995), northern Poland (Burakowski et al. 1979).

*Olophrum* **Erichson, 1839**.

*assimile* **(Paykull, 1800)**.Monsevičius 1985, 1986b, 1997; Dvilevičius et al. 1988; Silfverberg 1992, 2004; Gaidienė 1993; Pileckis and Monsevičius 1995; Smetana 2004a; Alonso-Zarazaga 2009a; Ivinskis et al. 2008.

*consimile* **(Gyllenhal, 1810)**. Pileckis 1976a; Silfverberg 1992, 2004; Pileckis and Monsevičius 1995; Monsevičius 1997; Smetana 2004a; Alonso-Zarazaga 2009a.

*fuscum* **(Gravenhorst, 1806)**. Monsevičius 1986b, 1997; Dvilevičius et al. 1988; Silfverberg 1992, 2004; Gaidienė 1993; Pileckis and Monsevičius 1995; Smetana 2004a; Alonso-Zarazaga 2009a; Ivinskis et al. 2009.

*piceum* **(Gyllenhal, 1810)**. Pileckis and Monsevičius 1995; Monsevičius 1997; Silfverberg 2004; Smetana 2004a; Alonso-Zarazaga 2009a; Ivinskis et al. 2009.

*rotundicolle* **(C.R. Sahlberg, 1830)**. Łomnicki 1913; Pileckis 1968b, 1976a; Silfverberg 1992, 2004; Pileckis and Monsevičius 1995; Smetana 2004a; Alonso-Zarazaga 2009a.

*Arpedium* **Erichson, 1839**.

*quadrum* **(Gravenhorst, 1806)**. Silfverberg 1992, 2004; Gaidienė 1993; Pileckis and Monsevičius 1995; Monsevičius 1997; Smetana 2004a; Alonso-Zarazaga 2009a; Ivinskis et al. 2009.

*Eucnecosum* **Reitter, 1909**.

*brachypterum* **(Gravenhorst, 1802)**. Silfverberg 1992, 2004; Pileckis and Monsevičius 1995; Monsevičius 1997; Smetana 2004a; Alonso-Zarazaga 2009a.

*Deliphrosoma* **Reitter, 1909**.

[*prolongatum* **(Rottenberg, 1873)**].Known in Estonia (Roosileht 2003), southern Poland (Burakowski et al. 1979).

*Acidota* **Stephens, 1829**.

*crenata* **(Fabricius, 1793)**. Bercio and Folwaczny 1979; Monsevičius 1986b, 1987, 1997; Silfverberg 1992, 2004; Gaidienė 1993; Pileckis and Monsevičius 1995; Smetana 2004a; Dapkus and Tamutis 2008a; Alonso-Zarazaga 2009a; Ivinskis et al. 2009.

*cruentata* **Mannerheim, 1830**. Pileckis 1976a; Silfverberg 1992, 2004; Pileckis and Monsevičius 1995; Monsevičius 1997; Smetana 2004a; Alonso-Zarazaga 2009a.

*Lesteva* **Latreille, 1797** = *Lesta* Backwelder, 1952.

[*hanseni* **Lohse, 1953 =** *frontinalis* auct. nec Kiesenwetter, 1850].Known in Denmark, southern Sweden (Lundberg and Gustafsson 1995).

*longoelytrata* **(Goeze, 1777)**. Pileckis 1976a; Monsevičius 1986b, 1997; Silfverberg 1992, 2004; Pileckis and Monsevičius 1995; Tamutis 2002b, c; Tamutis et al. 2004; Smetana 2004a; Alonso-Zarazaga 2009a; Ivinskis et al. 2009.

[*pubescens* **Mannerheim, 1830**].Known in Latvia (Telnov 2004), throughout the Sweden, Denmark (Lundberg and Gustafsson 1995), throughout the Poland (Burakowski et al. 1979).

[*punctata* **Erichson, 1839**]. Known in Denmark, southern Sweden, Estonia (Lundberg and Gustafsson 1995), northern Poland (Burakowski et al. 1979).

[*sicula heeri* **Fauvel, 1872**]. Known in Denmark, southern Sweden (Lundberg and Gustafsson 1995).

*Geodromicus* **Redtenbacher, 1857 =** *Psephidonus* Gistel, 1856.

*plagiatus* **(Fabricius, 1798)**. Smetana 2004a; Alonso-Zarazaga 2009a.

*Anthophagus* **Gravenhorst, 1802**.

*angusticollis* **(Mannerheim, 1830)=** *abreviatus* auct. nec (Fabricius, 1779).Monsevičius 1983; Silfverberg 1992, 2004; Gaidienė 1993; Pileckis and Monsevičius 1995; Smetana 2004a.

*caraboides* **(Linnaeus, 1758)**. Heyden 1903; Roubal 1910; Pileckis 1960, 1976a; Silfverberg 1992, 2004; Pileckis and Monsevičius 1995; Monsevičius 1997; Smetana 2004a.

[*omalinus* **Zetterstedt, 1828**].Known in throughout the Sweden, Latvia (Lundberg and Gustafsson 1995), Estonia (Silfverberg 2004), Poland (Burakowski et al. 1979).

**Coryphiini Jakobson, 1908**.

*Coryphium* **Stephens, 1834**.

*angusticolle* **Stephens, 1834**. Monsevičius 1983, 1997; Pileckis and Monsevičius 1995; Silfverberg 1992, 2004; Smetana 2004a; Alonso-Zarazaga 2009a.

**Proteininae Erichson, 1839**.

**Proteinini Erichson, 1839**.

*Metopsia* **Wollaston, 1854** *=* *Phloeobium* auct. nec Dejean, 1829.

[*clypeata* **(Müller, 1821)**]. Known in Denmark, southern Sweden (Lundberg and Gustafsson 1995), northern Poland (Burakowski et al. 1979).

[*similis* **Zerche, 1998**]. Known in Sweden, Denmark (Silfverberg 2004), Belarus, Poland (Alonso-Zarazaga 2009a).

*Megarthrus* **Curtis, 1829**.

*denticollis* **(Beck, 1817)**. Monsevičius 1983, 1997; Silfverberg 1992, 2004; Gaidienė 1993; Pileckis and Monsevičius 1995; Tamutis et al. 2004; Smetana 2004b; Alonso-Zarazaga 2009a; Vaivilavičius 2008.

*depressus* **(Paykull, 1789)** *= sinuaticollis* auct. nec (Lacordaire, 1835).Monsevičius 1983, 1997; Silfverberg 1992, 2004; Pileckis and Monsevičius 1995; Smetana 2004b; Vaivilavičius 2008; Alonso-Zarazaga 2009a; Ivinskis et al. 2009.

*hemipterus* **(Illiger, 1794)**. Pileckis 1976a; Silfverberg 1992, 2004; Gaidienė 1993; Pileckis and Monsevičius 1995; Smetana 2004b; Alonso-Zarazaga 2009a.

[*nitidulus* **Kraatz, 1857**].Known in Latvia (Telnov 2004), Sweden (Lundberg and Gustafsson 1995), Estonia (Roosileht 2003; Silfverberg 2004), northern Poland (Burakowski et al. 1979).

*proseni* **Schatzmayr, 1904** *= depressus* auct. nec (Paykull, 1789).Bercio and Folwaczny 1979; Silfverberg 1992, 2004; Smetana 2004b; Alonso-Zarazaga 2009a.

[*strandi* **Scheerpeltz, 1931**]. Known in Sweden (Lundberg and Gustafsson 1995), Estonia (Roosileht 2003; Silfverberg 2004).

*Proteinus* **Latreille, 1797** *=* *Pteronius* Backwelder, 1952.

*atomarius* **Erichson, 1840**. Pileckis 1976a; Silfverberg 1992, 2004; Pileckis and Monsevičius 1995; Smetana 2004b; Alonso-Zarazaga 2009a.

*brachypterus* **(Fabricius, 1792)**. Pileckis 1976a; Monsevičius 1986b, 1997; Silfverberg 1992, 2004; Pileckis and Monsevičius 1995; Smetana 2004b; Alonso-Zarazaga 2009a.

*laevigatus* **Hochhuth, 1872** *= macropterus* (Gyllenhal, 1810) nec (Gravenhorst, 1806). Monsevičius 1985, 1997; Silfverberg 1992, 2004; Pileckis and Monsevičius 1995; Tamutis 1999, 2002c; Tamutis et al. 2004; Smetana 2004b; Alonso-Zarazaga 2009a; Ivinskis et al. 2009.

[*ovalis* **Stephens, 1834**]. Known in northern Poland (Burakowski et al. 1979).

**Micropeplinae Leach, 1815**. (Micropeplidae)

*Arrhenopeplus* **Koch, 1937**.

*tesserula* **(Curtis, 1828)**. Pileckis 1976a (*Micropeplus*); Silfverberg 1992, 2004; Smetana 2004c; Alonso-Zarazaga 2009a.

*Micropeplus* **Latreille, 1809**.

*caelatus* **Erichson, 1839**. Pileckis 1976a; Silfverberg 1992, 2004; Pileckis and Monsevičius 1995; Smetana 2004c; Alonso-Zarazaga 2009a.

*fulvus* **Erichson, 1840**.Vaivilavičius 2008.

[*latus* **Hampe, 1861**].Known in southern Sweden (Lundberg and Gustafsson 1995).

[*longipennis* **Kraatz, 1859**]. Known in Ltavia (Vorst et al. 2007).

*porcatus* **(Paykull, 1789)**. Tamutis et al. 2004; Ferenca 2004.

[*staphylinoides* **(Marsham, 1802)**].Known in Latvia (Telnov, 2004), Estonia (Silfverberg 2004).

**Pselaphinae Latreille, 1802**.(Pselaphidae)

**Batrisini Reitter, 1882**.

*Batrisus* **Aubé, 1833**.

*formicarius* **Aubé, 1833**.# 39.Łomnicki 1913; Jakobson 1905-1915; Pileckis 1968b, 1976a; Silfverberg 1992, 2004; Pileckis and Monsevičius 1995; Smetana and Besuchet 2004.

*Batrisodes* **Reitter, 1882**.

[*adnexus* **(Hampe, 1863)**].Known in Denmark, southern Sweden (Lundberg and Gustafsson 1995), Poland (Burakowski et al. 1978), Estonia (Silfverberg 2004), recently found in Latvia (Vorst et al. 2007).

[*delaporti* **(Aubé, 1833)**].Known in southern Sweden (Lundberg and Gustafsson 1995), northwestern Belarus (Alexandrovich et al. 1996), norhern Poland (Burakowski et al. 1978).

*hubenthali* **Reitter, 1913**.Smetana and Besuchet 2004; Alonso-Zarazaga 2009a.

*venustus* **(Reichenbach, 1816)**. Pileckis 1976a; Silfverberg 1992, 2004; Pileckis and Monsevičius 1995; Smetana and Besuchet 2004.

**Clavigerini Leach, 1815**.

*Claviger* **Preyssler, 1790**.

[*longicornis* **O.F. Müller, 1818**]. Known in Denmark, southern Sweden (Lundberg and Gustafsson 1995), northeastern Poland (Burakowski et al. 1978).

*testaceus* **Preyssler, 1790**.Pileckis 1976a; Silfverberg 1992, 2004; Pileckis and Monsevičius 1995; Smetana and Besuchet 2004; Alonso-Zarazaga 2009a.

**Euplectini Streubel, 1839**.

*Meliceria* **Raffray, 1898**.

[*tragardhi* **Palm, 1938**]. Known in Latvia (Telnov, 2004), Sweden (Lundberg and Gustafsson 1995).

*Euplectus* **Leach, 1817**.

[*bescidicus* **Reitter, 1881** = *bohemicus* Machulka, 1930].Known in Denmark, southern and central Sweden (Lundberg and Gustafsson 1995), Estonia (Silfverberg 2004), northeastern Poland (Burakowski et al. 1978).

*bonvouloiri narentinus* **Reitter, 1881**. # 40.Jakobson 1905-1915.

[*bonvouloiri rosae* **Raffray, 1910**]. Known in Denmark, southern Sweden (Lundberg and Gustafsson 1995).

[*brunneus* **(Grimmer, 1841)**].Known in Denmark, southern Sweden (Lundberg and Gustafsson 1995), Belarus (Alexandrovich et al. 1996), Poland (Burakowski et al. 1978).

[*decipiens* **Raffray, 1910**]. Known throughout the Sweden (Lundberg and Gustafsson 1995), Estonia (Silfverberg 2004), Poland (Burakowski et al. 1978).

[*duponti* **Aubé, 1833**].Known in Latvia (Telnov, 2004), Denmark, southern Sweden (Lundberg and Gustafsson 1995), northern Poland (Gawroński and Oleksa 2006).

[*infirmus* **Raffray, 1910**]. Known in Denmark, southern Sweden (Lundberg and Gustafsson 1995).

*karstenii* **(Reichenbach, 1816)**. Pileckis 1976a; Silfverberg 1992, 2004; Pileckis and Monsevičius 1995; Smetana and Besuchet 2004; Alonso-Zarazaga 2009a.

*kirbii revelierei* **Reitter, 1884**.Smetana and Besuchet 2004; Alonso-Zarazaga 2009a.

[*mutator* **Fauvel, 1895 =** *fauveli* Guillebeau, 1888, nec Raffray, 1882 = *falsus* Bedel, 1906 = *tomlini* Joy, 1906].Known in Denmark, throughout Sweden (Lundberg and Gustafsson 1995), Poland (Burakowski et al. 1978), Estonia (Silfverberg 2004).

*nanus* **(Reichenbach, 1816)**. Pileckis 1976a; Silfverberg 1992, 2004; Pileckis and Monsevičius 1995; Smetana and Besuchet 2004.

*piceus* **Motschulsky, 1835**. Jakobson 1905-1915; Pileckis 1976a; Silfverberg 1992, 2004; Pileckis and Monsevičius 1995; Smetana and Besuchet 2004; Alonso-Zarazaga 2009a.

[*punctatus* **Mulsant, 1861**]. Known in Latvia (Telnov, 2004), Denmark, throughout Sweden (Lundberg and Gustafsson 1995), Poland (Burakowski et al. 1978).

*sanguineus* **Denny, 1825**. Łomnicki 1913; Jakobson 1905-1915; Pileckis 1968b, 1976a; Silfverberg 1992, 2004; Pileckis and Monsevičius 1995; Smetana and Besuchet 2004; Alonso-Zarazaga 2009a.

*signatus* **(Reichenbach, 1816)**. Pileckis 1976a; Silfverberg 1992, 2004; Pileckis and Monsevičius 1995; Smetana and Besuchet 2004; Alonso-Zarazaga 2009a.

[*tholini* **Guillebeau, 1888**]. Known in Denmark, southern Sweden (Lundberg and Gustafsson 1995), Poland (Alonso-Zarazaga 2009a).

*Plectophloeus* **Reitter, 1891**.

*fischeri* **(Aubé, 1833)**. Jakobson 1905-1915; Pileckis 1976a; Silfverberg 1992, 2004; Pileckis and Monsevičius 1995; Smetana and Besuchet 2004; Alonso-Zarazaga 2009a.

*nitidus* **(Fairmaire, 1857)**. # 41.Osterloff 1889; Łomnicki 1913; Jakobson 1905-1915; Pileckis and Monsevičius 1995.

*nubigena* **(Reitter, 1876)**.Smetana and Besuchet 2004; Alonso-Zarazaga 2009a.

*Saulcyella* **Reitter, 1901**.

*schmidtii* **(Märkel, 1844)**. Alonso-Zarazaga 2009a.

**Trichonychini Reitter, 1882**.

*Bibloporus* **Thomson, 1859**.

*bicolor* **(Denny, 1825)**. Pileckis 1976a; Silfverberg 1992, 2004; Pileckis and Monsevičius 1995; Alonso-Zarazaga 2009a.

[*minutus* **Raffray, 1914** = *hoglundi* Palm, 1948].Known in Latvia (Silfverberg 2004; Telnov et al. 2006), Denmark, throughout Sweden (Lundberg and Gustafsson 1995), western Poland (Burakowski et al. 1978).

[*ultimus* **Guillebeau, 1892**].Known in southern Sweden (Lundberg and Gustafsson 1995).

*Pseudoplectus* **Reitter, 1881**.

[*perplexus* **(Jacquelin du Val, 1854)**].Known in the central Sweden, Denmark (Lundberg and Gustafsson 1995).

*Bibloplectus* **Reitter, 1881**.

*ambiguus* **(Reichenbach, 1816)**. Pileckis 1976a; Silfverberg 1992, 2004; Pileckis and Monsevičius 1995; Monsevičius 1997; Smetana and Besuchet 2004; Alonso-Zarazaga 2009a.

[*minutissimus* **(Aubé, 1833)**]. Known in Denmark, southern Sweden (Lundberg and Gustafsson 1995), Latvia (Silfverberg 2004).

[*pusillus* **(Denny, 1825)**]. Known in Latvia (Telnov, 2004), Estonia, Denmark (Silfverberg 2004).

[*spinosus* **Raffray, 1914**]. Known in Latvia (Telnov, 2004), Estonia, Denmark, southern and central Sweden (Lundberg and Gustafsson 1995).

[*tenebrosus* **(Reitter, 1880)**].Known in southern and central Sweden, Denmark (Lundberg and Gustafsson 1995), Poland (Burakowski et al. 1978).

*Trimium* **Aubé, 1833**.

*brevicorne* **(Reichenbach, 1816)**. Pileckis 1976a; Silfverberg 1992, 2004; Pileckis and Monsevičius 1995; Monsevičius 1997; Smetana and Besuchet 2004; Alonso-Zarazaga 2009a.

*Amauronyx* **Reitter, 1881**.

[*maerkelii **(Aubé, 1844)**]. # 42. Pileckis 1976a; Silfverberg 1992; disproved by Pileckis and Monsevičius (1995); Smetana and Besuchet 2004; Alonso-Zarazaga 2009a.

*Trichonyx* **Chaudoir, 1845**.

*sulcicollis* **(Reichenbach, 1816)**. Pileckis 1976a; Silfverberg 1992, 2004; Pileckis and Monsevičius 1995; Smetana and Besuchet 2004; Alonso-Zarazaga 2009a.

**Brachyglutini Raffray, 1904**.

*Rybaxis* **Saulcy, 1876**.

*longicornis* **(Leach, 1817)**.Pileckis 1976a; Silfverberg 1992, 2004; Gaidienė 1993; Pileckis and Monsevičius 1995; Monsevičius 1997; Smetana and Besuchet 2004. Likely Mazurowa and Mazur (1939) and Pileckis (1960) this species reputed to be *Bryaxis sanguinea* Reichb.

*Brachygluta* **Thomson, 1859**.

*fossulata* **(Reichenbach, 1816)**.Eichwald 1830; Pileckis 1976a; Silfverberg 1992, 2004; Pileckis and Monsevičius 1995; Monsevičius 1997; Smetana and Besuchet 2004; Alonso-Zarazaga 2009a; Ivinskis et al. 2008.

[*haematica* **(Reichenbach, 1816)**]. # 43.Known in Latvia (Vorst et al. 2007), Denmark, Estonia, Poland, Sweden (Alonso-Zarazaga 2009a).

[*helferi* **(Schmidt-Goebel, 1836)**].Known in Denmark, southern Sweden (Lundberg and Gustafsson 1995), Poland (Burakowski et al. 1978).

*sinuata* **(Aubé, 1833)**. # 43.Smetana and Besuchet 2004; Alonso-Zarazaga 2009a.

*Reichenbachia* **Leach, 1826**.

*juncorum* **(Leach, 1817)**.Pileckis 1976a; Silfverberg 1992, 2004; Pileckis and Monsevičius 1995; Ivinskis et al. 1998; Smetana and Besuchet 2004; Alonso-Zarazaga 2009a.

*Fagniezia* **Jeannel, 1950**.

*impressa* **(Panzer, 1803)**.Pileckis 1976a (*Reichenbachia*); Silfverberg 1992, 2004; Pileckis and Monsevičius 1995 (*Trissemus*); Monsevičius 1997; Smetana and Besuchet 2004; Alonso-Zarazaga 2009a.

**Bythinini Raffray, 1890**.

*Bythinus* **Leach, 1817**.

[*burrellii* **Denny, 1825**].Known in Denmark, Estonia, southern Sweden (Lundberg and Gustafsson 1995), northern Poland (Burakowski et al. 1978).

*macropalpus* **Aubé, 1833** = *distinctus* Chaudoir, 1845.Pileckis 1976a; Silfverberg 1992, 2004; Pileckis and Monsevičius 1995; Smetana and Besuchet 2004; Alonso-Zarazaga 2009a.

*securiger* **(Reichenbach, 1816)**.# 44.Jakobson 1905-1915; Pileckis 1976a; Silfverberg 1992, 2004; Pileckis and Monsevičius 1995; Smetana and Besuchet 2004.

*Bryaxis* **Kugelann, 1794**.

*bulbifer* **(Reichenbach, 1816)**. Pileckis 1976a; Silfverberg 1992, 2004; Pileckis and Monsevičius 1995; Monsevičius 1997; Smetana and Besuchet 2004; Alonso-Zarazaga 2009a; Vaivilavičius 2008.

*clavicornis* **(Panzer, 1805)**.Monsevičius and Pankevičius 2001.

[*curtisii* **(Leach, 1817)**].Known in Latvia (Telnov 2004), northern Poland (Burakowski et al. 1978), Denmark, southern Sweden (Lundberg and Gustafsson 1995).

[*nigripennis* **(Aubé, 1844)**].Known in Latvia (Telnov 2004), Poland (Burakowski et al. 1978), Belarus (Alexandrovich et al. 1996).

*nodicornis* **(Aubé, 1833)**. # 45.Jakobson 1905-1915; Pileckis 1976a (*Euthia*).

*puncticollis* **(Denny, 1825)** = *validus* (Aubé, 1844).Jakobson 1905-1915; Pileckis 1976a; Silfverberg 1992, 2004; Pileckis and Monsevičius 1995; Monsevičius 1997; Smetana and Besuchet 2004; Alonso-Zarazaga 2009a.

**Tychini Raffray, 1904**.

*Tychus* **Leach, 1817**.

[*monilicornis* **Reitter, 1880**].Known in Denmark, southern Sweden (Lundberg and Gustafsson 1995).

*niger* **(Paykull, 1800)**.Jakobson 1905-1915; Pileckis 1976a; Silfverberg 1992, 2004; Pileckis and Monsevičius 1995; Smetana and Besuchet 2004; Alonso-Zarazaga 2009a.

[*normandi* **Jeannel, 1950**].Known in Denmark, southern Sweden (Lundberg and Gustafsson 1995).

**Ctenistini Blanchard, 1845**.

*Chennium* **Latreille, 1807**.

[*bituberculatum* **Latreille, 1807**]. Known in southern Sweden (Lundberg and Gustafsson 1995), Poland (Burakowski et al. 1978).

*Centrotoma* **Heyden, 1849**.

*lucifuga* **Heyden, 1849**.Pileckis and Monsevičius 1995; Silfverberg 2004.

**Pselaphini Latreille, 1802**.

*Pselaphaulax* **Reitter, 1909**.

*dresdensis* **(Herbst, 1792)**.Jakobson 1905-1915; Pileckis 1976a; Gaidienė 1993; Silfverberg 1992, 2004; Pileckis and Monsevičius 1995; Smetana and Besuchet 2004; Alonso-Zarazaga 2009a.

*Pselaphus* **Herbst, 1792**.

*heisei* **Herbst, 1792**.Lešinskas and Pileckis 1967; Pileckis 1976a; Silfverberg 1992, 2004; Pileckis and Monsevičius 1995; Monsevičius 1997; Smetana and Besuchet 2004; Alonso-Zarazaga 2009a.

**Tyrini Reitter, 1882**.

*Tyrus* **Aubé, 1833**.

*mucronatus* **(Panzer, 1803)**.Pileckis 1976a; Gaidienė 1993; Silfverberg 1992, 2004; Pileckis and Monsevičius 1995; Smetana and Besuchet 2004; Alonso-Zarazaga 2009a; Alekseev 2008a.

**Phloeocharinae Erichson, 1839**.

*Phloeocharis* **Mannerheim, 1830**.

*subtilissima* **Mannerheim, 1830**.Bercio and Folwaczny 1979; Miländer et al. 1984; Monsevičius 1986b, 1997; Silfverberg 1992, 2004; Pileckis and Monsevičius 1995; Smetana 2004d; Alonso-Zarazaga 2009a; Ivinskis et al. 2009.

**Olisthaerinae Thomson, 1859**.

*Olisthaerus* **Dejean, 1833**.

*megacephalus* **(Zetterstedt, 1828)**.Pileckis 1960, 1976a; Silfverberg 1992, 2004; Pileckis and Monsevičius 1995; Smetana 2004d; Ferenca 2006b; Alonso-Zarazaga 2009a.

*substriatus* **(Paykull, 1790)**.Pileckis 1976a; Silfverberg 1992, 2004; Pileckis and Monsevičius 1995; Smetana 2004d; Alonso-Zarazaga 2009a.

**Tachyporinae MacLeay 1825**.

**Mycetoporini Thomson, 1859** = Bolitobiini Horn, 1877.

*Mycetoporus* **Mannerheim, 1830**.

[*aequalis* **Thomson, 1868**]. Known in Denmark, Estonia, throughout Sweden (Lundberg and Gustafsson 1995).

*baudueri* **Mulsant & Rey, 1875** = *hellieseni* Strand, 1950.Monsevičius 1983; Silfverberg 1992, 2004; Pileckis and Monsevičius 1995; Smetana 2004d.

*bimaculatus* **Lacordaire, 1835** *= ruficornis* Kraatz, 1857.Monsevičius 1997.

*clavicornis* **(Stephens, 1832)**. Bercio and Folwaczny 1979; Monsevičius 1985, 1997; Dvilevičius et al. 1988; Silfverberg 1992, 2004; Pileckis and Monsevičius 1995; Smetana 2004d; Ivinskis et al. 2009.

[*debilis* **Mäklin, 1847**]. Known in northwestern Belarus (Alexandrovich et al. 1996), northern and central Russia (Alonso-Zarazaga 2009a).

[*eppelsheimianus* **Fagel, 1968**]. Known in Sweden, Denmark, Belarus, Kaliningrad region, Poland (Alonso-Zarazaga 2009a).

*erichsonanus* **Fagel, 1965** *= baudueri* auct. nec Mulsant & Rey, 1875.Monsevičius 1985, 1997; Dvilevičius et al. 1988; Silfverberg 1992, 2004; Pileckis and Monsevičius 1995.

[*forticornis* **Fauvel, 1875**]. Known in Denmark, Estonia, throughout Sweden (Lundberg and Gustafsson 1995), Poland (Alonso-Zarazaga 2009a).

*lepidus* **(Gravenhorst, 1806)** *= brunneus* (Marsham, 1802) nec (Fabricius, 1798).Pileckis 1960, 1976a; Monsevičius 1986b, 1987, 1997; Silfverberg 1992, 2004; Gaidienė 1993; Pileckis and Monsevičius 1995; Smetana 2004d; Ferenca 2006b.

*longulus* **Mannerheim, 1830**.Pileckis 1976a; Bercio and Folwaczny 1979; Silfverberg 1992, 2004; Pileckis and Monsevičius 1995; Smetana 2004d.

[*maeklini* **Bernhauer, 1906**].Known in Sweden (Lundberg and Gustafsson 1995), Estonia (Roosileht 2003).

[*niger* **Fairmaire & Laboulbène, 1856**]. Known in Sweden, Denmark (Lundberg and Gustafsson 1995), central Russia, Poland (Alonso-Zarazaga 2009a), northwestern Belarus (Alexandrovich et al. 1996).

*nigricollis* **Stephens, 1835** *= splendens* (Marsham, 1802) nec (Fabricius, 1793).Pileckis 1960, 1976a; Silfverberg 1992, 2004; Gaidienė 1993; Pileckis and Monsevičius 1995; Monsevičius 1997; Smetana 2004d; Ferenca 2006b.

[*piceolus* **Rey, 1882**]. Known in Estonia (Lundberg and Gustafsson 1995; Silfverberg 1992, 2004).

*punctus* **(Gravenhorst, 1806)**.Pileckis 1976a; Bercio and Folwaczny 1979; Silfverberg 1992, 2004; Pileckis and Monsevičius 1995; Monsevičius 1997; Smetana 2004d.

*rufescens* **(Stephens, 1832)**.Pileckis 1976a; Silfverberg 1992, 2004; Pileckis and Monsevičius 1995; Monsevičius 1997; Smetana 2004d; Ivinskis et al. 2009.

[*solidicornis reichei* **Pandellé, 1869**]. Known in northwestern Belarus (Alexandrovich et al. 1996), Estonia (Silfverberg 1992, 2004).

*tenuis* **Mulsant & Rey, 1853** *= mulsanti* Ganglbauer, 1895.Monsevičius 1983, 1986b, 1987, 1997; Silfverberg 1992, 2004; Pileckis and Monsevičius 1995; Smetana 2004d.

*Ischnosoma* **Stephens, 1829**.

*bergrothi* **(Hellén, 1925)**.Monsevičius 1983, 1997; Silfverberg 1992, 2004; Pileckis and Monsevičius 1995 (*Mycetoporus*); Alonso-Zarazaga 2009a.

*longicorne* **(Mäklin, 1847)**.Monsevičius and Pankevičius 2001; Tamutis et al. 2008.

*splendidum* **(Gravenhorst, 1806)**.Monsevičius 1982, 1997; Dvilevičius et al. 1988; Silfverberg 1992, 2004; Pileckis and Monsevičius 1995 (*Mycetoporus*); Tamutis et al. 2004; Smetana 2004d; Alekseev 2008a.

*Bryoporus* **Kraatz, 1857**.

*cernuus* **(Gravenhorst, 1806)** *= merdarius* (Olivier, 1794) nec (Fabricius, 1775).Monsevičius 1985; Silfverberg 1992, 2004; Pileckis and Monsevičius 1995; Smetana 2004d.

*Bryophacis* **Reitter 1909**.

*crassicornis* **(Mäklin, 1847)**.Monsevičius and Pankevičius 2001; Ivinskis et al. 2009.

*rufus* **(Erichson, 1839)**.Pileckis 1976a; Bercio and Folwaczny 1979; Silfverberg 1992, 2004; Pileckis and Monsevičius 1995 (*Bryoporus*); Smetana 2004d.

[*rugipennis* **(Pandellè, 1869)].** Known in Latvia (Telnov 2004), Sweden, Estonia (Lundberg and Gustafsson 1995).

*Carphacis* **DesGozis, 1886**

[*striatus* **(Olivier, 1795)** *= angularis* (Paykull, 1800)].Known in Denmark, southern Sweden (Lundberg and Gustafsson 1995), Belarus, Kaliningrad region, Poland (Alonso-Zarazaga 2009a).

*Lordithon* **Thomson, 1859** = *Bolitobius* auct. nec Leach, 1819.

*exoletus* **(Erichson, 1839)** *= bernhaueri* (Wanka, 1929).Monsevičius 1985, 1997; Silfverberg 1992, 2004; Pileckis and Monsevičius 1995; Smetana 2004d.

*lunulatus* **(Linnaeus, 1761)**.Pileckis 1960, 1976a; Silfverberg 1992, 2004; Gaidienė 1993; Pileckis and Monsevičius 1995; Monsevičius 1986b, 1997; Šablevičius 2000b; Smetana 2004d.

*pulchellus* **(Mannerheim, 1830)**.Pileckis 1976a; Silfverberg 1992, 2004; Pileckis and Monsevičius 1995; Monsevičius 1997; Smetana 2004d.

[*speciosus* **(Erichson, 1840)**].Known in Latvia (Telnov 2004), Sweden, Estonia (Lundberg and Gustafsson 1995), Poland (Alonso-Zarazaga 2009a).

*thoracicus* **(Fabricius, 1777)**. Monsevičius 1983, 1986b, 1987, 1997; Silfverberg 1992, 2004; Pileckis and Monsevičius 1995; Smetana 2004d; Alekseev 2008a.

*trimaculatus* **(Fabricius, 1793)**. Pileckis 1976a; Silfverberg 1992, 2004; Pileckis and Monsevičius 1995; Smetana 2004d.

*trinotatus* **(Erichson, 1839)**. Roubal 1910; Pileckis 1960, 1976a; Gaidienė 1993; Silfverberg 1992, 2004; Pileckis and Monsevičius 1995; Smetana 2004d.

*Bolitobius* **Leach, 1819** *=* *Bryocharis* Lacordaire, 1835.

*castaneus* **(Stephens, 1832)** *= analis* auct. nec (Fabricius, 1787).Monsevičius 1982, 1997; Dvilevičius et al. 1988; Silfverberg 1992, 2004; Gaidienė 1993; Pileckis and Monsevičius 1995; Smetana 2004d.

*cingulatus* **Mannerheim, 1830**. Pileckis 1976a; Silfverberg 1992, 2004; Gaidienė 1993; Pileckis and Monsevičius 1995; Monsevičius 1997; Smetana 2004d.

*Parabolitobius* **Li, Zhao & Sakai, 2000**.

*formosus* **(Gravenhorst, 1806)**. Monsevičius 1982, 1997; Dvilevičius et al. 1988; Silfverberg 1992, 2004; Gaidienė 1993; Pileckis and Monsevičius 1995; Smetana 2004d.

[*inclinans* **(Gravenhorst, 1806)**].Known in Denmark, southern Sweden (Lundberg and Gustafsson 1995), Poland, central Russia (Alonso-Zarazaga 2009a).

**Tachyporini MacLeay, 1825**.

*Sepedophilus* **Gistel, 1856** *=* *Conosoma* auct. nec Kraatz, 1857.

*bipunctatus* **(Gravenhorst, 1802)**. Monsevičius 1985; Silfverberg 1992, 2004; Pileckis and Monsevičius 1995; Smetana 2004d.

*bipustulatus* **(Gravenhorst, 1802)**. Pileckis 1976a; Silfverberg 1992, 2004; Pileckis and Monsevičius 1995; Monsevičius 1997; Smetana 2004d.

[*constans* **(Fowler, 1888)** *= strigosus* (J.R. Sahlberg, 1911) =*stoeckli* (Lokay, 1913)].Known in Latvia (Telnov 2004), Sweden (Lundberg and Gustafsson 1995), Poland (Alonso-Zarazaga 2009a).

*immaculatus* **(Stephens, 1832)**. Monsevičius 1982, 1997; Silfverberg 1992, 2004; Pileckis and Monsevičius 1995; Smetana 2004d.

*littoreus* **(Linnaeus, 1758)**. Monsevičius 1983; Silfverberg 1992, 2004; Pileckis and Monsevičius 1995; Smetana 2004d.

*marshami* **(Stephens, 1832)**. Monsevičius 1985, 1988a, 1997; Silfverberg 1992, 2004; Pileckis and Monsevičius 1995; Tamutis et al. 2004; Smetana 2004d; Vorst et al. 2007; Alekseev 2008a.

[*nigripennis* **(Stephens, 1832)** = *lividus* (Erichson, 1839)]. Known in northern Poland (Burakowski et al. 1980), Denmark (Silfverberg 2004), Sweden (Lundberg and Gustafsson 1995).

[*obtusus* **(Luze, 1902)**]. Known in Belarus (Smetana 2004d), Poland (Burakowski et al. 1980).

*pedicularius* **(Gravenhorst, 1802)**. Monsevičius 1983, 1986b, 1997; Silfverberg 1992, 2004; Pileckis and Monsevičius 1995; Smetana 2004d.

*testaceus* **(Fabricius, 1793)**. Pileckis 1976a (*S. pubescens*); Silfverberg 1992, 2004; Pileckis and Monsevičius 1995; Monsevičius 1997; Smetana 2004d.

*wankowiczi* **(Pandellé, 1869)**.Alonso-Zarazaga 2009a.

*Tachyporus* **Gravenhorst, 1802**.

*abdominalis* **(Fabricius, 1781)**. Pileckis 1960, 1976a; Dvilevičius et al. 1988; Silfverberg 1992, 2004; Pileckis and Monsevičius 1995; Smetana 2004d; Alonso-Zarazaga 2009a.

*atriceps* **Stephens, 1832**. Pileckis 1976a; Bercio and Folwaczny 1979; Silfverberg 1992, 2004; Pileckis and Monsevičius 1995; Smetana 2004d.

*chrysomelinus* **(Linnaeus, 1758)**. Pileckis 1960, 1976a; Monsevičius 1986b, 1987, 1997; Silfverberg 1992, 2004; Gaidienė 1993; Pileckis and Monsevičius 1995; Tamutis 1999, 2002b, c; Tamutis et al. 2004; Smetana 2004d; Ferenca 2006b; Vaivilavičius 2008.

*corpulentus* **J.R. Sahlberg, 1876**. Karalius and Monsevičius 1992; Pileckis and Monsevičius 1995; Silfverberg 1996, 2004.

[*dispar* **(Paykull, 1789)**].Known in Denmark, southern Sweden (Lundberg and Gustafsson 1995), Belarus, central Russia (Alonso-Zarazaga 2009a).

*formosus* **Matthews, 1838**.Monsevičius 1997; Monsevičius and Pankevičius 2001.

*hypnorum* **(Fabricius, 1775)**. Eichwald 1830; Pileckis 1960, 1976a; Monsevičius 1986b, 1997; Dvilevičius et al. 1988; Silfverberg 1992, 2004; Gaidienė 1993; Pileckis and Monsevičius 1995; Tamutis 1999, 2002b, c; Tamutis et al. 2004, 2007; Smetana 2004d; Ferenca 2006b.

*nitidulus* **(Fabricius, 1781)**. Eichwald 1830; Monsevičius 1982, 1986b, 1997; Silfverberg 1992, 2004; Pileckis and Monsevičius 1995; Smetana 2004d.

*obtusus* **(Linnaeus, 1767)**. Pileckis 1960, 1976a; Monsevičius 1986b, 1997; Silfverberg 1992, 2004; Gaidienė 1993; Pileckis and Monsevičius 1995; Tamutis and Zolubas 2001; Tamutis 1999, 2002b; Tamutis et al. 2004; Smetana 2004d; Ferenca 2006b.

*pallidus* **Sharp, 1871** *= scutellaris* Rye, 1871, nec Lacordaire, 1835.Monsevičius 1982, 1997; Pileckis and Monsevičius 1995; Silfverberg 1992, 2004.

*pulchellus* **Mannerheim, 1843**. Pileckis 1976a; Bercio and Folwaczny 1979; Silfverberg 1992, 2004; Pileckis and Monsevičius 1995; Monsevičius 1997; Smetana 2004d.

*pusillus* **Gravenhorst, 1806** *= macropterus* Stephens, 1832.Bercio and Folwaczny 1979; Monsevičius 1982, 1997; Silfverberg 1992, 2004; Gaidienė 1993; Pileckis and Monsevičius 1995; Tamutis 2002b; Smetana 2004d.

*quadriscopulatus* **Pandellé, 1869** = *signifer* Pandellé, 1872. Pileckis and Monsevičius 1995; Silfverberg 2004.

[*ruficollis* **Gravenhorst, 1802**].Known in Latvia (Telnov 2004), Denmark, Estonia (Lundberg and Gustafsson 1995), Poland (Alonso-Zarazaga 2009a).

*scitulus* **Erichson, 1839**. Karalius and Monsevičius 1992; Silfverberg 1996, 2004; Monsevičius 1997; Smetana 2004d.

*solutus* **Erichson, 1839**. Pileckis 1976a; Silfverberg 1992, 2004; Gaidienė 1993; Pileckis and Monsevičius 1995; Monsevičius 1997; Smetana 2004d.

*tersus* **Erichson, 1840**.Tamutis et al. 2008; Ivinskis et al. 2009.

*transversalis* **Gravenhorst, 1806**. Monsevičius 1982, 1986b, 1987, 1997; Dvilevičius et al. 1988; Silfverberg 1992, 2004; Gaidienė 1993; Pileckis and Monsevičius 1995; Smetana 2004d.

*Lamprinodes* **Luze, 1901**.

*saginatus* **(Gravenhorst, 1806)**.Pileckis 1960, 1976a; Silfverberg 1992, 2004; Pileckis and Monsevičius 1995; Smetana 2004d; Ferenca 2006b.

*Lamprinus* **Heer, 1839**.

[*erythropterus* **(Panzer, 1796)**]. Known in Latvia (Telnov 2004), Estonia (Lundberg and Gustafsson 1995), Poland (Alonso-Zarazaga 2009a).

*Tachinus* **Gravenhorst, 1802**.

*basalis* **Erichson, 1840**. Pileckis 1976a; Silfverberg 1992, 2004; Pileckis and Monsevičius 1995.

*bipustulatus* **(Fabricius, 1793)**. Gaidienė 1993; Silfverberg 2004.

*corticinus* **Gravenhorst, 1802**. Pileckis 1960, 1976a; Dvilevičius et al. 1988; Monsevičius 1987, 1997; Silfverberg 1992, 2004; Pileckis and Monsevičius 1995; Smetana 2004d; Ferenca 2006b; Vaivilavičius 2008.

*elongatus* **Gyllenhal, 1810**. Eichwald 1830; Pileckis 1976a; Silfverberg 1992, 2004; Pileckis and Monsevičius 1995; Smetana 2004d.

*fimetarius* **Gravenhorst, 1802**. Pileckis and Monsevičius 1995; Silfverberg 2004; Smetana 2004d.

*humeralis* **Gravenhorst, 1802**. Gaidienė 1993; Silfverberg 2004.

*laticollis* **Gravenhorst, 1802**. Roubal 1910; Pileckis 1960, 1976a; Monsevičius 1986b, 1987, 1997; Dvilevičius et al. 1988; Silfverberg 1992, 2004; Gaidienė 1993; Pileckis and Monsevičius 1995; Smetana 2004d; Ferenca 2006b.

*lignorum* **(Linnaeus, 1758)**. Pileckis 1960, 1976a; Bercio and Folwaczny 1979; Gaidienė 1993; Silfverberg 1992, 2004; Pileckis and Monsevičius 1995; Smetana 2004d; Alonso-Zarazaga 2009a; as *T. flavipes* (Steven, 1808) in Ferenca 2006b.

*marginatus* **(Fabricius, 1793)**. Monsevičius 1983, 1997; Silfverberg 1992, 2004; Gaidienė 1993; Pileckis and Monsevičius 1995; Smetana 2004d.

*marginellus* **(Fabricius, 1781)**. Bercio and Folwaczny 1979; Monsevičius 1985, 1997; Dvilevičius et al. 1988; Silfverberg 1992, 2004; Gaidienė 1993; Pileckis and Monsevičius 1995; Smetana 2004d.

*pallipes* **(Gravenhorst, 1806)**.Pileckis 1960, 1976a; Silfverberg 1992, 2004; Gaidienė 1993; Pileckis and Monsevičius 1995; Monsevičius 1997; Smetana 2004d; Ferenca 2006b.

*proximus* **Kraatz, 1855**. Pileckis 1976a; Silfverberg 1992, 2004; Pileckis and Monsevičius 1995; Smetana 2004d.

[*punctipennis* **(J. Sahlberg, 1876)**].Known in southern Estonia (Roosileht 2003).

*rufipennis* **Gyllenhal, 1810**. Pileckis 1976a; Monsevičius 1987, 1997; Silfverberg 1992, 2004; Pileckis and Monsevičius 1995; Smetana 2004d.

[*scapularis* **Stephens, 1832**]. Recently found in Latvia (Cibulskis et al. 2009), known in Poland (Alonso-Zarazaga 2009a).

[*schneideri* **Luze, 1900**]. Recently found in Latvia (Cibulskis et al. 2003), known in central Russia (Alonso-Zarazaga 2009a).

*signatus* **Gravenhorst, 1802** *= rufipes* auct. nec (Linnaeus, 1758). Monsevičius 1982, 1986b, 1987, 1997; Dvilevičius et al. 1988; Silfverberg 1992, 2004; Gaidienė 1993; Pileckis and Monsevičius 1995; Tamutis 2002b, c; Tamutis et al. 2004, 2007; Smetana 2004d; Vaivilavičius 2008.

*subterraneus* **(Linnaeus, 1758)**. Pileckis 1960, 1976a; Gaidienė 1993; Silfverberg 1992, 2004; Pileckis and Monsevičius 1995; Smetana 2004d; Ferenca 2006b.

*Coproporus* **Kraatz, 1857** = *Erchomus* Motschulsky, 1858.

*colchicus* **Kraatz, 1857** *= heterocerus* auct. nec (Fauvel, 1898).Tamutis and Ferenca 2006; Ferenca et al. 2006; Ferenca et al. 2007.

*Cilea* **Jacquelin du Val, 1856** = *Leucoparyphus* Kraatz, 1857.

*silphoides* **(Linnaeus, 1767)**. Pileckis 1960, 1976a; Silfverberg 1992, 2004; Pileckis and Monsevičius 1995; Smetana 2004d; Ferenca 2006b.

**Habrocerinae Mulsant & Rey, 1876**.

*Habrocerus* **Erichson, 1839**.

*capillaricornis* **(Gravenhorst, 1806)**. Monsevičius 1983, 1986b, 1987, 1997; Silfverberg 1992, 2004; Pileckis and Monsevičius 1995 .

**Trichophyinae Thomson, 1858**.

*Trichophya* **Mannerheim, 1830**.

*pilicornis* **(Gyllenhal, 1810)**. Monsevičius and Jakaitis 1984; Silfverberg 1992, 2004; Pileckis and Monsevičius 1995; Smetana 2004d.

**Aleocharinae Fleming, 1821**.

**Aleocharini Fleming, 1821**.

*Aleochara* **Gravenhorst, 1802**.

*bilineata* **Gyllenhal, 1810**. Pileckis 1976a; Silfverberg 1992, 2004; Pileckis and Monsevičius 1995; Smetana 2004d.

*binotata* **Kraatz, 1856** = *verna* auct. nec Say, 1836.Pileckis 1976a; Silfverberg 1992, 2004; Pileckis and Monsevičius 1995; Monsevičius 1997; Smetana 2004d.

*bipustulata* **(Linnaeus, 1761)**. Lešinskas and Pileckis 1967; Monsevičius 1985; Silfverberg 1992, 2004; Pileckis and Monsevičius 1995; Smetana 2004d.

*brevipennis* **Gravenhorst, 1806**. Bercio and Folwaczny 1979; Dvilevičius et al. 1988; Silfverberg 1992, 2004; Gaidienė 1993; Pileckis and Monsevičius 1995; Monsevičius 1997; Smetana 2004d.

[*cuniculorum* **Kraatz, 1858**].Known in Denmark, southern Sweden (Lundberg and Gustafsson 1995), Estonia (Roosileht 2003), northwestern Belarus (Alexandrovich et al. 1996), Poland (Smetana 2004d).

*curtula* **(Goeze, 1777)**. Pileckis 1976a; Silfverberg 1992, 2004; Gaidienė 1993; Pileckis and Monsevičius 1995; Monsevičius 1997; Smetana 2004d.

*erythroptera* **Gravenhorst, 1806**. Pileckis 1960, 1976a; Silfverberg 1992, 2004; Gaidienė 1993; Pileckis and Monsevičius 1995; Smetana 2004d.

*fumata* **Gravenhorst, 1802**. Monsevičius 1985, 1997; Silfverberg 1992, 2004; Pileckis and Monsevičius 1995; Smetana 2004d.

*funebris* **Wollaston, 1864** = *albovillosa* Bernhauer, 1901.Monsevičius 1985, 1997; Silfverberg 1992, 2004; Pileckis and Monsevičius 1995; Smetana 2004d.

*grisea* **Kraatz, 1856**. Pileckis 1976a; Bercio and Folwaczny 1979; Silfverberg 1992, 2004; Pileckis and Monsevičius 1995; Smetana 2004d; Ivinskis et al. 2009.

[*haemoptera ripicola* **Mulsant & Rey, 1874**]. Known in Estonia, Finland (Silfverberg 2004), Belarus (Alexandrovich et al. 1996).

[*inconspicua* **Aubé, 1850**]. Known in Denmark, Estonia, throughout Sweden (Lundberg and Gustafsson 1995), Latvia (Cibulskis et al. 2003, Telnov 2004), Poland (Smetana 2004d).

*intricata* **Mannerheim, 1830**. Roubal 1910; Pileckis 1960, 1976a; Bercio and Folwaczny 1979; Silfverberg 1992, 2004; Pileckis and Monsevičius 1995; Smetana 2004d; Ivinskis et al. 2009.

*kamila* **Likovský, 1984 =** *diversa* (J.R. Sahlberg, 1876) nec Mulsant & Rey, 1853.Pileckis 1976a; Silfverberg 1992, 2004; Pileckis and Monsevičius 1995; Smetana 2004d.

*laevigata* **Gyllenhal, 1810**. Pileckis 1976a; Silfverberg 1992, 2004; Pileckis and Monsevičius 1995; Smetana 2004d.

*lanuginosa* **Gravenhorst, 1802**. Roubal 1910; Pileckis 1960, 1976a; Silfverberg 1992, 2004; Gaidienė 1993; Pileckis and Monsevičius 1995; Monsevičius 1997; Smetana 2004d.

[*lygaea* **Kraatz, 1862**].Known in Denmark, southern Sweden (Lundberg and Gustafsson 1995), Estonia (Roosileht 2003).

*moerens* **Gyllenhal, 1827**.Monsevičius 1985, 1997; Silfverberg 1992, 2004; Pileckis and Monsevičius 1995; Smetana 2004d.

*moesta* **Gravenhorst, 1802**.Pileckis 1976a; Silfverberg 1992, 2004; Pileckis and Monsevičius 1995; Smetana 2004d.

[*obscurella* **Gravenhorst, 1806** = *algarum* Fauvel, 1862].Known in Denmark, southern Sweden (Lundberg and Gustafsson 1995).

[*punctatella* **Motschulsky, 1858** = *obscurella* auct. nec Gravenhorst, 1806].Known in Denmark, southern Sweden, Estonia (Lundberg and Gustafsson 1995).

[*ruficornis* **Gravenhorst, 1802**].Known in Denmark (Lundberg and Gustafsson 1995). 

*sanguinea* **(Linnaeus, 1758)**.Monsevičius and Pankevičius 2001.

[*spadicea* **(Erichson, 1837)**].Known in Denmark, Estonia, throughout Sweden (Lundberg and Gustafsson 1995), Poland (Smetana 2004d).

*sparsa* **Heer, 1839**. Pileckis 1976a; Monsevičius 1987, 1997, 1999; Silfverberg 1992, 2004; Pileckis and Monsevičius 1995; Smetana 2004d.

*stichai* **Likovský, 1965**. Monsevičius 1985, 1988; 1997, 1999; Silfverberg 1992, 2004; Pileckis and Monsevičius 1995; Smetana 2004d.

*tristis* **Gravenhorst, 1806**. Bercio and Folwaczny 1979; Monsevičius 1985; Silfverberg 1992, 2004; Pileckis and Monsevičius 1995; Smetana 2004d.

*verna* **Say, 1836**. Bercio and Folwaczny 1979; Gaidienė 1993; Silfverberg 2004.

*villosa* **Mannerheim, 1830**.Monsevičius and Pankevičius 2001.

*Tinotus* **Sharp, 1883**.

*morion* **(Gravenhorst, 1802)**. # 46.Roubal 1910; Pileckis 1960, 1976a; Bercio and Folwaczny 1979; Silfverberg 1992, 2004; Pileckis and Monsevičius 1995; Monsevičius 1997; Smetana 2004d.

**Oxypodini Thomson 1859**.

*Oxypoda* **Mannerheim, 1830**.

*abdominalis* **(Mannerheim, 1830)**. Monsevičius 1985, 1987, 1997; Silfverberg 1992, 2004; Pileckis and Monsevičius 1995; Smetana 2004d.

*acuminata* **(Stephens, 1832)** = *lividipennis* auct. nec Mannerheim, 1830.Pileckis 1976a; Bercio and Folwaczny 1979; Silfverberg 1992, 2004; Monsevičius 1987, 1997; Gaidienė 1993; Pileckis and Monsevičius 1995; Smetana 2004d.

*advena* **Mäklin, 1846**. Monsevičius 1985; Silfverberg 1992, 2004; Pileckis and Monsevičius 1995; Smetana 2004d.

*alternans* **(Gravenhorst, 1802)**. Monsevičius 1985, 1987, 1997; Silfverberg 1992, 2004; Pileckis and Monsevičius 1995; Smetana 2004d.

*annularis* **(Mannerheim, 1830)**. Monsevičius 1983, 1986b, 1997; Dvilevičius et al. 1988; Silfverberg 1992, 2004; Pileckis and Monsevičius 1995; Smetana 2004d.

*arborea* **Zerche, 1994** = *lucens* auct. nec Mulsant & Rey, 1853.Monsevičius and Pankevičius 2001

[*bicolor* **Mulsant & Rey, 1853**].Known in Sweden (Lundberg and Gustafsson 1995), Estonia (Roosileht 2003), recently found in Latvia (Telnov et al. 2006).

[*brachyptera* **(Stephens, 1832)**]. Known in Denmark, southern Sweden, Estonia (Lundberg and Gustafsson 1995), Belarus (Alexandrovich et al. 1996).

*brevicornis* **(Stephens, 1832)** = *umbrata* (Gyllenhal, 1810) nec (Gravenhorst, 1802).Monsevičius 1983, 1986b, 1997; Dvilevičius et al. 1988; Gaidienė 1993; Pileckis and Monsevičius 1995; Silfverberg 1992, 2004; Smetana 2004d.

[*carbonaria* **(Heer, 1841)**].Known in Denmark (Silfverberg 2004).

*elongatula* **Aubé, 1850**. Pileckis 1976a; Monsevičius 1986b, 1987, 1997; Silfverberg 1992, 2004; Pileckis and Monsevičius 1995; Smetana 2004d.

*exoleta* **Erichson, 1839**. Pileckis and Monsevičius 1995; Silfverberg 2004.

*ferruginea* **Erichson, 1839**. Pileckis 1976a; Silfverberg 1992, 2004; Pileckis and Monsevičius 1995; Smetana 2004d.

*filiformis* **Redtenbacher, 1849**. Horion 1967; Bercio and Folwaczny 1979; Silfverberg 1992, 2004; Pileckis and Monsevičius 1995; Smetana 2004d.

[*flavicornis* **Kraatz, 1856** = *amoena* Fairmaire & Laboulbène, 1856].Known in Denmark, throughout Sweden (Lundberg and Gustafsson 1995), Latvia (Telnov 2004), Belarus (Alexandrovich et al. 1996).

*formiceticola* **Märkel, 1841**. Monsevičius 1985, 1987, 1997; Silfverberg 1992, 2004; Pileckis and Monsevičius 1995; Smetana 2004d.

[*funebris* **Kraatz, 1856** = *laticollis* (Thomson, 1871)].Known in Denmark, Estonia, throughout Sweden (Lundberg and Gustafsson 1995), Latvia (Telnov 2004), Poland (Smetana 2004d).

*haemorrhoa* **(Mannerheim, 1830)**. Bercio and Folwaczny 1979; Monsevičius 1985, 1987, 1997; Silfverberg 1992, 2004; Pileckis and Monsevičius 1995; Smetana 2004d.

*induta* **Mulsant & Rey, 1861**. Monsevičius and Pankevičius 2001.

*lentula* **Erichson, 1837**. Silfverberg 1992, 2004; Pileckis and Monsevičius 1995; Smetana 2004d.

*longipes* **Mulsant & Rey, 1861**. Monsevičius 1985, 1987, 1997, 1999; Silfverberg 1992, 2004; Pileckis and Monsevičius 1995; Smetana 2004d.

*opaca* **(Gravenhorst, 1802)**. Pileckis 1976a; Silfverberg 1992, 2004; Pileckis and Monsevičius 1995; Smetana 2004d; Ivinskis et al. 2009.

*praecox* **Erichson, 1839**. Łomnicki 1913; Pileckis 1968b, 1976a; Monsevičius 1986b, 1997; Silfverberg 1992, 2004; Pileckis and Monsevičius 1995; Smetana 2004d.

[*pratensicola* **Lohse, 1970** = *scanica* Lundberg and Gustafsson, 1980].Known in southern Sweden (Lundberg and Gustafsson 1995).

*procerula* **Mannerheim, 1830**. Monsevičius 1983, 1997; Silfverberg 1992, 2004; Pileckis and Monsevičius 1995; Smetana 2004d.

*recondita* **Kraatz, 1856**. Pileckis and Monsevičius 1995; Monsevičius 1997; Silfverberg 2004.

*rufa* **Kraatz, 1856**. Monsevičius and Pankevičius 2001.

[*rugicollis* **Kraatz, 1856**]. Known in Sweden (Lundberg and Gustafsson 1995), Estonia (Roosileht 2003).

*skalitzyi* **Bernhauer, 1902**. Monsevičius 1983, 1986b, 1987, 1997; Silfverberg 1992, 2004; Pileckis and Monsevičius 1995; Smetana 2004d.

[*soror* **Thomson, 1855**]. Known in Denmark, Estonia, throughout Sweden (Lundberg and Gustafsson 1995), Latvia (Cibulskis et al. 2003).

*spectabilis* **Märkel, 1844**. Monsevičius 1985, 1987, 1997; Silfverberg 1992, 2004; Pileckis and Monsevičius 1995; Smetana 2004d.

[*strandi* **Scheerpeltz, 1957**].Known in Sweden, Denmark (Lundberg and Gustafsson 1995).

[*tarda* **Sharp, 1871**]. Known in Denmark, southern Sweden (Lundberg and Gustafsson 1995). 

*testacea* **Erichson, 1837**. Pileckis 1976a; Silfverberg 1992, 2004; Pileckis and Monsevičius 1995; Smetana 2004d.

*togata* **Erichson, 1837**. Bercio and Folwaczny 1979; Silfverberg 1992, 2004; Smetana 2004d.

[*vicina* **Kraatz, 1856**]. Known in Denmark, Estonia, throughout Sweden (Lundberg and Gustafsson 1995), Latvia (Cibulskis and Petrova 2002), Belarus (Alexandrovich et al. 1996), Poland (Smetana 2004d).

*vittata* **Märkel, 1842**. Monsevičius 1985, 1987, 1997; Silfverberg 1992, 2004; Pileckis and Monsevičius 1995; Smetana 2004d.

*Devia* **Blackwelder, 1952** *=* *Dasyglossa* Kraatz, 1856, nec Illiger, 1807.

*prospera* **(Erichson, 1839)**.Pileckis and Monsevičius 1995; Silfverberg 2004.

*Hygropora* **Kraatz, 1856**.

[*cunctans* **(Erichson, 1837)**]. Known in Denmark, throughout Sweden (Lundberg and Gustafsson 1995), Estonia (Roosileht 2003), Latvia (Telnov 2004), Belarus (Alexandrovich et al. 1996).

*Ocyusa* **Kraatz, 1856**.

[*maura* **(Erichson, 1837)**].Known in Denmark, throughout Sweden (Lundberg and Gustafsson 1995), Estonia (Roosileht 2003), Latvia (Telnov 2004), Belarus (Alexandrovich et al. 1996).

*Deubelia* **Bernhauer, 1899**.

*picina* **(Aubé, 1850)**. Monsevičius 1983, 1986b, 1997; Silfverberg 1992, 2004; Gaidienė 1993; Pileckis and Monsevičius 1995; Smetana 2004d.

*Chilomorpha* **Krása, 1914**.

*longitarsis* **(Thomson, 1867)** = *laticollis* auct. nec (Thomson, 1871) = *hibernica* (Rye, 1876).Ivinskis et al. 2009.

*Calodera* **Mannerheim, 1830** = *Ityocara* Thomson, 1867.

*aethiops* **(Gravenhorst, 1802)**. Pileckis 1976a; Monsevičius 1986b, 1997; Silfverberg 1992, 2004; Pileckis and Monsevičius 1995; Smetana 2004d.

[*nigrita* **Mannerheim, 1830**]. Known in Denmark, Estonia, throughout Sweden (Lundberg and Gustafsson 1995, Silfverberg 2004), Latvia (Cibulskis et al. 2003; Telnov 2004).

[*protensa* **Mannerheim, 1830**]. Known in Denmark, Estonia, southern Sweden (Lundberg and Gustafsson 1995).

*riparia* **Erichson, 1837**. Monsevičius 1985, 1997; Silfverberg 1992, 2004; Pileckis and Monsevičius 1995; Smetana 2004d.

[*rubens* **Erichson, 1837**].Known in Latvia (Telnov 2004), Denmark, southern Sweden (Lundberg and Gustafsson 1995), Estonia (Roosileht 2003), Belarus (Alexandrovich et al. 1996).

[*rufescens* **Kraatz, 1856**]. Known in Denmark, southern Sweden, Estonia (Lundberg and Gustafsson 1995).

*uliginosa* **Erichson, 1837**. Monsevičius 1983, 1997; Silfverberg 1992, 2004; Pileckis and Monsevičius 1995; Smetana 2004d.

*Parocyusa* **Bernhauer, 1902** = *Chilopora* Kraatz, 1867, nec Haime, 1854.

[*longitarsis* **(Erichson, 1839)**].Known in Denmark, Estonia (Silfverberg 2004).

*rubicunda* **(Erichson, 1837)**.Smetana 2004d.

*Stichoglossa* **Fairmaire & Laboulbène, 1856**.

[*semirufa* **(Erichson, 1839)**]. Known in Denmark (Lundberg and Gustafsson 1995; Silfverberg 2004).

*Ischnoglossa* **Kraatz, 1856**.

*elegantula* **(Mannerheim, 1830)**.Smetana 2004d.

[*obscura* **Wunderle, 1990**]. Known in Denmark, southern Sweden (Lundberg and Gustafsson 1995).

[*prolixa* **(Gravenhorst, 1802)**]. Known in Denmark, Estonia, throughout Sweden (Lundberg and Gustafsson 1995), Latvia (Telnov 2004).

*Dexiogyia* **Thomson, 1858**.

*corticina* **(Erichson, 1837)**. Pileckis 1976a; Silfverberg 1992, 2004; Pileckis and Monsevičius 1995; Smetana 2004d.

*Thiasophila* **Kraatz, 1856** = *Thyasophila* Fairmaire & Laboulbéne, 1856.

*angulata* **(Erichson, 1837)**. Monsevičius 1985, 1987, 1997; Silfverberg 1992, 2004; Pileckis and Monsevičius 1995; Smetana 2004d.

[*canaliculata* **Mulsant & Rey, 1874**].Known in Denmark, Estonia, throughout Sweden (Lundberg and Gustafsson 1995), Belarus (Alexandrovich et al. 1996).

[*inquilina* **(Märkel, 1842)**]. Known in Denmark, Estonia, southern Sweden (Lundberg and Gustafsson 1995), Belarus (Alexandrovich et al. 1996), recently found in Latvia (Telnov et al. 2008).

[*lohsei* **Zerche, 1987**]. Known in southern Sweden (Lundberg and Gustafsson 1995).

[*wockii* **(Schneider, 1862)** = *rotundicollis* Jansson, 1943].Known in Denmark, throughout Sweden (Lundberg and Gustafsson 1995).

*Crataraea* **Thomson, 1858**.

*suturalis* **(Mannerheim, 1830)**. Monsevičius 1985, 1987, 1997; Silfverberg 1992, 2004; Pileckis and Monsevičius 1995; Smetana 2004d.

*Haploglossa* **Kraatz, 1856** = *Microglotta* Kraatz, 1862.

*gentilis* **(Märkel, 1844)**. Pileckis 1976a; Silfverberg 1992, 2004; Pileckis and Monsevičius 1995; Smetana 2004d; Ostrauskas and Ferenca 2010.

*marginalis* **(Gravenhorst, 1806)**. Monsevičius 1985, 1997; Silfverberg 1992, 2004; Pileckis and Monsevičius 1995; Smetana 2004d.

*nidicola* **(Fairmaire, 1852)**. Pileckis 1976a; Silfverberg 1992, 2004; Pileckis and Monsevičius 1995; Monsevičius 1999; Smetana 2004d.

*picipennis* **(Gyllenhal, 1827)**. Seidlitz 1891; Bercio and Folwaczny 1979; Monsevičius 1985, 1987, 1997, 1999; Silfverberg 1992, 2004; Pileckis and Monsevičius 1995; Smetana 2004d.

*villosula* **(Stephens, 1832)** = *pulla* (Gyllenhal, 1827) nec (Gravenhorst, 1802).Pileckis 1976a; Silfverberg 1992, 2004; Pileckis and Monsevičius 1995; Monsevičius 1997; Monsevičius 1999; Smetana 2004d.

*Mniusa* **Mulsant & Rey, 1875**.

*incrassata* **(Mulsant & Rey, 1852)**. Monsevičius 1983, 1997; Silfverberg 1992, 2004; Pileckis and Monsevičius 1995; Smetana 2004d.

*Poromniusa* **Ganglbauer, 1895** = *Eurymniusa* Ganglbauer, 1895.

[*crassa* **(Eppelsheim, 1883)**]. Known in Denmark, Latvia (Silfverberg 2004).

*procidua* **(Erichson, 1837)**. Łomnicki 1913; Horion 1967; Silfverberg 1992, 2004; Pileckis and Monsevičius 1995.

*Ocalea* **Erichson, 1837**.

*badia* **Erichson, 1837**.Monsevičius 1983, 1997; Dvilevičius et al. 1988; Silfverberg 1992, 2004; Gaidienė 1993; Pileckis and Monsevičius 1995; Smetana 2004d.

[*concolor* **Kiesenwetter, 1847**]. Known in Denmark, Estonia (Lundberg and Gustafsson 1995; Silfverberg 1992, 2004).

[*latipennis* **Sharp, 1870**]. Known in Denmark, Sweden (Lundberg and Gustafsson 1995; Silfverberg 1992, 2004).

*picata* **(Stephens, 1832)**. Monsevičius 1985; Silfverberg 1992, 2004; Pileckis and Monsevičius 1995; Smetana 2004d; Ivinskis et al. 2009.

*rivularis* **Miller, 1851**. Pileckis 1976a; Silfverberg 1992, 2004; Pileckis and Monsevičius 1995; Smetana 2004d.

*Ilyobates* **Kraatz, 1856**.

*bennetti* **Donisthorpe, 1914** = *subopacus* Palm, 1953.Pileckis 1976a; Bercio and Folwaczny 1979; Silfverberg 1992, 2004; Pileckis and Monsevičius 1995; Smetana 2004d.

*nigricollis* **(Paykull, 1800)**. Monsevičius 1985, 1986b, 1997; Dvilevičius et al. 1988; Silfverberg 1992, 2004; Gaidienė 1993; Pileckis and Monsevičius 1995; Smetana 2004d; Ivinskis et al. 2008.

*Amarochara* **Thomson, 1858**.

[*bonnairii* **(Fauvel, 1865)**]. Known in Denmark, Sweden (Silfverberg 2004).

*forticornis* **(Lacordaire, 1835)**. Monsevičius 1999.

*umbrosa* **(Erichson, 1837)**. Monsevičius 1998.

*Phloeopora* **Erichson, 1837** = *Phloedroma* Kraatz, 1856.

[*bernhaueri* **Lohse, 1984**]. Known in Denmark (Lundberg and Gustafsson 1995; Silfverberg 2004).

*concolor* **(Kraatz, 1856)**. Pileckis 1976a; Silfverberg 1992, 2004; Pileckis and Monsevičius 1995; Smetana 2004d.

*corticallis* **(Gravenhorst, 1802)** = *teres* auct. nec (Gravenhorst, 1802) =*angustiformis* auct. nec Baudi, 1869.Lohse 1984; Pileckis and Monsevičius 1995; Monsevičius 1997; Silfverberg 1992, 2004; Smetana 2004d.

[*nitidiventris* **Fauvel, 1900**]. Known in northwestern Belarus (Alexandrovich et al. 1996), Sweden (Lundberg and Gustafsson 1995), Estonia (Roosileht 2003).

[*teres* **(Gravenhorst, 1802)**]. Known in Denmark (Lundberg and Gustafsson 1995; Silfverberg 2004).

*testacea* **(Mannerheim, 1830)**. Roubal 1910; Pileckis 1960, 1976a; Monsevičius 1987, 1997; Silfverberg 1992, 2004; Pileckis and Monsevičius 1995; Smetana 2004d.

*Dinarda* **Samouelle, 1819**.

[*dentata* **(Gravenhorst, 1806)**]. Known in Latvia (Telnov 2004), Sweden, Denmark (Lundberg and Gustafsson 1995; Silfverberg 2004), Belarus (Alexandrovich et al. 1996).

[*hagensii* **Wasmann, 1889**]. Known in Latvia (Telnov 2004), Sweden, Denmark (Lundberg and Gustafsson 1995; Silfverberg 2004).

*maerkelii* **Kiesenwetter, 1843**. Tamutis and Ferenca 2006; Ferenca et al. 2006.

*Meotica* **Mulsant & Rey, 1873**.

[*exilis* **(Knoch, 1806)** = *exiliformis* Joy, 1915].Known in Sweden, Denmark, Estonia (Lundberg and Gustafsson 1995; Silfverberg 2004), Belarus (Alexandrovich et al. 1996), recently found in Latvia (Telnov et al. 2008).

[*exillima* **Sharp, 1915**]. Known in Denmark (Lundberg and Gustafsson 1995; Silfverberg 2004).

[*filiformis* **(Motschulsky, 1860)** = *capitalis* Mulsant & Rey, 1873 = *apicalis* G. Benick, 1953 = *clavata* G. Benick, 1953]. Known in Latvia (Telnov 2004), Sweden, Denmark (Lundberg and Gustafsson 1995), Estonia (Silfverberg 2004), Belarus (Alexandrovich et al. 1996).

[*finnmarchica* **G. Benick, 1953**]. Kown in southern Sweden (Lundberg and Gustafsson 1995), Latvia (Vorst et al. 2007).

[*pallens* **(Redtenbacher, 1849)** = *lohsei* G. Benick, 1953 = *hanseni* Scheerpeltz, 1954 = *strandi* Scheerpeltz, 1958].Known in Sweden, Denmark (Lundberg and Gustafsson 1995; Silfverberg 2004), Belarus (Alexandrovich et al. 1996), recently found in Latvia (Telnov et al. 2008).

[*winkleri* **G. Benick, 1954**]. Known in Denmark (Silfverberg 2004).

*Dasygnypeta* **Lohse, 1974**.

*velata* **(Erichson, 1837)**.Pileckis 1976a; Silfverberg 1992, 2004; Pileckis and Monsevičius 1995; Monsevičius 1997; Smetana 2004d; Ivinskis et al. 2009.

*Gnypeta* **Thomson, 1858**.

[*caerulea* **(C.R. Sahlberg, 1831)**]. Known in Sweden, Estonia (Lundberg and Gustafsson 1995; Silfverberg 2004), Poland (Burakowski et al. 1980), Latvia (Telnov et al. 2006).

[*carbonaria* **(Mannerheim, 1830)**]. Known in Sweden, Denmark, Estonia (Lundberg and Gustafsson 1995; Silfverberg 2004), Poland (Burakowski et al. 1980), recently found in Latvia (Vorst et al. 2007).

[*ripicola* **(Kiesenwetter, 1844)**]. Known in Sweden, Denmark (Lundberg and Gustafsson 1995; Silfverberg 2004), Belarus (Alexandrovich et al. 1996), Poland (Burakowski et al. 1980).

*rubrior* **Tottenham, 1839**.Monsevičius 1997.

*Ischnopoda* **Stephens, 1832** = *Tachyusa* Erichson, 1837.

*atra* **(Gravenhorst, 1806)**. Monsevičius 1985, 1997; Gaidienė 1993; Pileckis and Monsevičius 1995; Silfverberg 1992, 2004; Smetana 2004d.

*coarctata* **(Erichson, 1837)**. Monsevičius 1985, 1997; Silfverberg 1992, 2004; Pileckis and Monsevičius 1995; Smetana 2004d.

*constricta* **(Erichson, 1837)**. Monsevičius 1985, 1997; Silfverberg 1992, 2004; Pileckis and Monsevičius 1995; Smetana 2004d.

*leucopus* **(Marsham, 1802)**. Bercio and Folwaczny 1979; Monsevičius 1983, 1987, 1997; Silfverberg 1992, 2004; Gaidienė 1993; Pileckis and Monsevičius 1995; Smetana 2004d.

*scitula* **(Erichson, 1837)**. Monsevičius 1985; Silfverberg 1992, 2004; Pileckis and Monsevičius 1995; Smetana 2004d.

*umbratica* **(Erichson, 1837)**. Pileckis 1976a; Gaidienė 1993; Silfverberg 1992, 2004; Pileckis and Monsevičius 1995; Smetana 2004d.

*Brachyusa* **Mulsant & Rey, 1874**.

*concolor* **(Erichson, 1839)**. Pileckis 1976a; Silfverberg 1992, 2004; Pileckis and Monsevičius 1995; Smetana 2004d.

**Athetini Casey, 1910**.

*Dochmonota* **Thomson, 1859**.

[*clancula* **(Erichson, 1837)**]. Recently found in Latvia (Vorst et al. 2007), known in Sweden, Denmark, Estonia (Lundberg and Gustafsson 1995; Silfverberg 2004), Poland (Burakowski et al. 1980).

[*rudiventris* **(Eppelsheim, 1886)**]. Known in Sweden (Lundberg and Gustafsson 1995; Silfverberg 2004) Poland (Burakowski et al. 1980).

*Dacrila* **Mulsant & Rey, 1874**.

[*fallax* **(Kraatz, 1856)**]. Known in Sweden, Denmark, Estonia (Lundberg and Gustafsson 1995; Silfverberg 2004), northwestern Belarus (Alexandrovich et al. 1996), northern Poland (Burakowski et al. 1980), recently found in Latvia (Telnov et al. 2008).

*Brundinia* **Tottenham, 1949**.

[*marina* **(Mulsant & Rey, 1853)**]. Known in Sweden, Denmark, Estonia (Lundberg and Gustafsson 1995; Silfverberg 2004).

[*meridionalis* **(Mulsant & Rey, 1853)**]. Known in Sweden, Denmark, Estonia (Lundberg and Gustafsson 1995; Silfverberg 2004).

*Thinoecia* **Mulsant & Rey, 1873**.

[*fragilicornis* **Kraatz, 1856**].Known in northern Poland (Burakowski et al. 1980).

*Hydrosmecta* **Thomson, 1858** = *Hydrosmectina* Ganglbauer, 1895.

[*longula* **(Heer, 1839)** = *thinobioides* (Kraatz, 1854)].Known in Sweden, Denmark, Estonia (Lundberg and Gustafsson 1995; Silfverberg 2004), Poland (Burakowski et al. 1980), recently found in Latvia (Vorst et al. 2007).

[*septentrionum* **(G. Benick, 1969)**].Known in Denmark (Silfverberg 2004).

[*subtilissima* **(Kraatz, 1854)** = *obscurior* (G. Benick, 1969)]. Known in Sweden, Estonia (Lundberg and Gustafsson 1995; Silfverberg 2004), Poland (Burakowski et al. 1980).

*Dilacra* **Thomson, 1858**.

[*luteipes* **(Erichson, 1837)**].Known in Sweden, Denmark, Estonia (Lundberg and Gustafsson 1995; Silfverberg 2004), northwestern Belarus (Alexandrovich et al. 1996).

*vilis* **(Erichson, 1837)**. Pileckis 1960, 1976a; Pileckis and Monsevičius 1995; Monsevičius 1997; Silfverberg 1992, 2004; Smetana 2004d; Ferenca 2006b; Ivinskis et al. 2009.

*Ousipalia* **Gozis, 1886**.

[*caesula* **(Erichson, 1839)**]. Known in Sweden, Denmark, Estonia (Lundberg and Gustafsson 1995; Silfverberg 2004), northern Poland (Burakowski et al. 1980).

*Tomoglossa* **Kraatz, 1856**.

[*luteicornis* **(Erichson, 1837)**]. Known in Sweden, Estonia (Lundberg and Gustafsson 1995), Denmark (Silfverberg 2004). 

*Schistoglossa* **Kraatz, 1856**.

*aubei* **(Brisout, 1860)**. Smetana 2004d.

[*bergvalli* **Palm, 1968** = *benicki* Lohse, 1981].Known in Sweden, Denmark (Lundberg and Gustafsson 1995; Silfverberg 2004).

*curtipennis* **(Sharp, 1869)**. Monsevičius 1985, 1986b, 1997; Silfverberg 1992, 2004; Pileckis and Monsevičius 1995; Smetana 2004d; Vorst et al. 2007.

[*drusilloides* **(J.R. Sahlberg, 1876)**]. Known in Sweden, Denmark (Lundberg and Gustafsson 1995; Silfverberg 2004).

*gemina* **(Erichson, 1837)**. Monsevičius 1983, 1997; Silfverberg 1992, 2004; Pileckis and Monsevičius 1995; Smetana 2004d.

[*pseudogemina* **G. Benick, 1981**].Known in Denmark (Silfverberg 2004).

*viduata* **(Erichson, 1837)**. Monsevičius 1983, 1997; Silfverberg 1992, 2004; Pileckis and Monsevičius 1995; Smetana 2004d.

*Aloconota* **Thomson, 1858**.

[*debilicornis* **(Erichson, 1839)**]. Known in Latvia (Telnov 2004).

*gregaria* **(Erichson, 1839)**. Pileckis 1960, 1976a; Monsevičius 1986b, 1987, 1997; Silfverberg 1992, 2004; Pileckis and Monsevičius 1995; Tamutis et al. 2004; Smetana 2004d; Ferenca 2006b; Ivinskis et al. 2009.

[*insecta* **(Thomson, 1856)**]. Known in Latvia (Vorst et al. 2007), Sweden, Denmark, Estonia (Lundberg and Gustafsson 1995; Silfverberg 2004), Poland (Burakowski et al. 1980).

[*planifrons* **(Waterhouse, 1864)**]. Known in Sweden, Denmark, Estonia (Lundberg and Gustafsson 1995; Silfverberg 2004), Poland (Burakowski et al. 1980).

[*subgrandis* **(Brundin, 1954)**]. Known Denmark, throughout Sweden (Lundberg and Gustafsson 1995; Silfverberg 2004).

*sulcifrons* **(Stephens, 1832)**. Monsevičius 1998; Silfverberg 2004; Vorst et al. 2007.

*Disopora* **Thomson, 1859**.

[*coulsoni* **(Last, 1952)**]. Known Denmark, southern Sweden (Lundberg and Gustafsson 1995; Silfverberg 2004).

*languida* **(Erichson, 1837)**.Monsevičius 1983, 1986b, 1997; Silfverberg 1992, 2004; Pileckis and Monsevičius 1995 (*Aloconota*); Smetana 2004d.

[*longicollis* **(Mulsant & Rey, 1852)**]. Known in Estonia (Lundberg and Gustafsson 1995; Silfverberg 2004), Poland (Burakowski et al. 1980).

[*ultima* **(G. Benick & Lohse, 1959)**]. Known in Estonia (Lundberg and Gustafsson 1995; Silfverberg 2004), Poland (Burakowski et al. 1980).

*Liogluta* **Thomson, 1858**.

*alpestris* **(Heer, 1839)** = *nitidula* (Kraatz, 1856).Monsevičius 1985, 1997; Silfverberg 1992, 2004; Pileckis and Monsevičius 1995.

*granigera* **(Kiesenwetter, 1850)**. Pileckis 1976a; Silfverberg 1992, 2004; Gaidienė 1993; Pileckis and Monsevičius 1995; Monsevičius 1997; Smetana 2004d; Ivinskis et al. 2009.

*longiuscula* **(Gravenhorst, 1802)**. Monsevičius 1983, 1986b, 1987, 1997; Silfverberg 1992, 2004; Pileckis and Monsevičius 1995; Smetana 2004d.

[*micans* **(Mulsant & Rey, 1852)** = *letzneri* (Eppelsheim, 1880)]. Known in Sweden, Denmark, Estonia (Lundberg and Gustafsson 1995; Silfverberg 2004), northwestern Belarus (Alexandrovich et al. 1996), northeastern Poland (Burakowski et al. 1980).

*microptera* **Thomson, 1867** = *oblongiuscula* (Sharp, 1869) = *oblonga* (Erichson, 1839) nec (Lacordaire, 1835).Monsevičius 1985, 1997; Dvilevičius et al. 1988; Silfverberg 1992, 2004; Gaidienė 1993; Pileckis and Monsevičius 1995; Smetana 2004d.

[*pagana* **(Erichson, 1839)**]. Known Denmark, southern Sweden, Estonia (Lundberg and Gustafsson 1995; Silfverberg 2004), northern Poland (Burakowski et al. 1980).

*Geostiba* **Thomson, 1858** = *Sipalia* auct. nec Mulsant & Rey, 1853 =*Evanystes* auct. nec Gistel, 1856.

*circellaris* **(Gravenhorst, 1802)**. # 47.Roubal 1910; Pileckis 1960; Pileckis 1976a; Jakaitis and Valenta 1976; Monsevičius 1986b, 1987, 1997; Silfverberg 1992, 2004; Pileckis and Monsevičius 1995.

*Taxicera* **Mulsant & Rey, 1873**.

*deplanata **Gravenhorst, 1802**.# 48.Pileckis 1976a; Pileckis and Monsevičius 1995.

*Callicerus* **Gravenhorst, 1802**.

*obscurus* **Gravenhorst, 1802**. Tamutis and Ferenca 2006; Ferenca et al. 2006, 2007.

[*rigidicornis* **(Erichson, 1839)**]. Known in Denmark (Silfverberg 2004), Poland (Burakowski et al. 1980).

*Dadobia* **Thomson, 1858**.

*immersa* **(Erichson, 1837)**. Monsevičius and Jakaitis 1984; Silfverberg 1992, 2004; Pileckis and Monsevičius 1995; Monsevičius 1997, 2000; Smetana 2004d.

*Paranopleta* **Brundin, 1954**.

[*inhabilis* **(Kraatz, 1856)**]. Known in Sweden, Denmark, Estonia (Lundberg and Gustafsson 1995; Silfverberg 2004), Poland (Burakowski et al. 1980).

*Cadaverota* **Yosii & Sawada, 1976**.

*cadaverina* **(Brisout, 1860)**.Pileckis 1976a; Bercio and Folwaczny 1979; Silfverberg 1992, 2004; Pileckis and Monsevičius 1995; Monsevičius 1987, 1997; Smetana 2004d.

[*hansseni* **(Strand, 1943)**]. Known in Sweden, Denmark (Lundberg and Gustafsson 1995; Silfverberg 2004).

*Philhygra* **Mulsant & Rey, 1873**.(*Atheta*)

*arctica* **(Thomson, 1856)**.Monsevičius 1983, 1986b, 1997; Silfverberg 1992, 2004; Pileckis and Monsevičius 1995; Smetana 2004d.

[*botildae* **(Brundin, 1954)**].Known in Sweden, Denmark, Estonia (Lundberg and Gustafsson 1995; Silfverberg 2004).

[*britteni* **(Joy, 1913)**]. Known in Latvia (Telnov 2004), Sweden, Estonia (Lundberg and Gustafsson 1995; Silfverberg 2004).

*debilis* **(Erichson, 1837)**. Monsevičius 1985, 1997; Silfverberg 1992, 2004; Pileckis and Monsevičius 1995; Smetana 2004d.

[*deformis* **(Kraatz, 1856)**]. Known in Denmark, southern Sweden (Lundberg and Gustafsson 1995; Silfverberg 2004).

*elongatula* **(Gravenhorst, 1802)**.Pileckis 1976a; Dvilevičius et al. 1988; Silfverberg 1992, 2004; Gaidienė 1993; Pileckis and Monsevičius 1995; Monsevičius 1997; Tamutis et al. 2004; Smetana 2004d.

*fallaciosa* **(Sharp, 1869)**. Łomnicki 1913; Pileckis 1968b, 1976; Monsevičius 1986b, 1997; Silfverberg 1992, 2004; Pileckis and Monsevičius 1995; Smetana 2004d.

*gyllenhalii* **(Thomson, 1856)**. Monsevičius 1985, 1986b, 1997; Silfverberg 1992, 2004; Pileckis and Monsevičius 1995; Smetana 2004d.

[*grisea* **(Thomson, 1852)**].Known in Latvia (Telnov 2004), Sweden, Denmark, Estonia (Lundberg and Gustafsson 1995; Silfverberg 2004).

*hygrobia* **(Thomson, 1856)** = *magniceps* (J.R. Sahlberg, 1876).Monsevičius 1983, 1987, 1997; Silfverberg 1992, 2004; Pileckis and Monsevičius 1995; Smetana 2004d.

[*hygrotopora* **(Kraatz, 1856)**]. Known in Latvia (Telnov et al. 2007), Sweden, Denmark, Estonia (Lundberg and Gustafsson 1995; Silfverberg 2004).

[*kaiseriana* **(Brundin, 1943)**]. Known in Denmark, southern Sweden (Lundberg and Gustafsson 1995; Silfverberg 2004).

*luridipennis* **(Mannerheim, 1830)**. Tenenbaum 1923; Pileckis 1968b, 1976a; Silfverberg 1992, 2004; Pileckis and Monsevičius 1995; Smetana 2004d.

[*mahleri* **Muona, 1995**]. Known Denmark (Lundberg and Gustafsson 1995; Silfverberg 2004).

*malleus* **(Joy, 1913)** = *hygrobia* auct. nec (Thomson, 1856).Monsevičius 1983, 1997; Silfverberg 1992, 2004; Gaidienė 1993; Pileckis and Monsevičius 1995; Smetana 2004d; Ivinskis et al. 2009.

*melanocera* **(Thomson, 1856)**. Pileckis 1976a; Monsevičius 1987, 1997; Silfverberg 1992, 2004; Gaidienė 1993; Pileckis and Monsevičius 1995; Smetana 2004d.

[*obtusangula* **(Joy, 1913)**].Known in Latvia (Telnov 2004), Sweden, Denmark (Lundberg and Gustafsson 1995; Silfverberg 2004).

*palustris* **(Kiesenwetter, 1844)**. Monsevičius 1985; Silfverberg 1992, 2004; Pileckis and Monsevičius 1995; Smetana 2004d.

[*parca* **(Mulsant & Rey, 1874)** = *nannion* (Joy, 1931)].Known in Latvia (Telnov 2004), Sweden, Denmark, Estonia (Lundberg and Gustafsson 1995; Silfverberg 2004).

*ripicola* **(Hanssen, 1932)** = *flavithorax* (G. Benick, 1976).Monsevičius 1985; Silfverberg 1992, 2004; Gaidienė 1993; Pileckis and Monsevičius 1995; Smetana 2004d.

[*scotica* **(Elliman, 1909)**].Known in Denmark, southern Sweden (Lundberg and Gustafsson 1995; Silfverberg 2004).

[*terminalis* **(Gravenhorst, 1806)**].Known in Latvia (Telnov 2004), Sweden, Denmark, Estonia (Lundberg and Gustafsson 1995; Silfverberg 2004), Belarus (Alexandrovich et al. 1996).

[*tmolosensis* **(Bernhauer, 1940)** = *denifera* (Brundin, 1943)].Known in Sweden, Denmark (Lundberg and Gustafsson 1995; Silfverberg 2004), Belarus (Alexandrovich et al. 1996).

*volans* **(W. Scriba, 1859)** = *halophila* (Thomson, 1861) = *tomlini* (Joy, 1913).Smetana 2004d.

*Atheta* **Thomson, 1858**.

[*aeneicollis* **(Sharp, 1869)** = *pertyi* auct. nec (Heer, 1839)].Known in Latvia (Telnov 2004), Denmark, southern Sweden (Lundberg and Gustafsson 1995; Silfverberg 2004).

*aeneipennis* **(Thomson, 1856)** = *picipennis* auct. nec (Mannerheim, 1843).Pileckis 1976a; Silfverberg 1992, 2004; Pileckis and Monsevičius 1995; Smetana 2004d.

*allocera* **Eppelsheim, 1893**.Monsevičius 2000.

*amicula* **(Stephens, 1832)**. Bercio and Folwaczny 1979; Monsevičius 1985; Silfverberg 1992, 2004; Pileckis and Monsevičius 1995; Tamutis et al. 2004; Smetana 2004d.

[*amplicollis* **(Mulsant & Rey, 1873)**]. Known in Sweden, Denmark (Lundberg and Gustafsson 1995; Silfverberg 2004), Belarus (Alexandrovich et al. 1996).

*aquatica* **(Thomson, 1852)**. Pileckis 1976a; Silfverberg 1992, 2004; Pileckis and Monsevičius 1995; Smetana 2004d.

[*aquatilis* **(Thomson, 1867)**].Known in Denmark, southern Sweden (Lundberg and Gustafsson 1995; Silfverberg 2004).

[*atomaria* **(Kraatz, 1856)**].Known in Denmark, southern Sweden (Lundberg and Gustafsson 1995; Silfverberg 2004).

*atramentaria* **(Gyllenhal, 1810)**. Pileckis 1976a; Monsevičius 1986b, 1997; Silfverberg 1992, 2004; Pileckis and Monsevičius 1995; Smetana 2004d.

*autumnalis* **(Erichson, 1839)**. Łomnicki 1913; Pileckis 1968b, 1976a; Silfverberg 1992, 2004; Pileckis and Monsevičius 1995; Smetana 2004d; Vorst et al. 2007.

*basicornis* **(Mulsant & Rey, 1852)**. Monsevičius 1983, 1987, 1997; Silfverberg 1992, 2004; Pileckis and Monsevičius 1995; Smetana 2004d.

*benickiella* **Brundin, 1948**]. Known in Sweden, Denmark (Lundberg and Gustafsson 1995), Estonia (Roosileht 2003), Belarus (Alexandrovich et al. 1996).

*boleticola* **J.R. Sahlberg, 1876**. Monsevičius 1985, 1987, 1988a, 1997, 2000; Silfverberg 1992, 2004; Pileckis and Monsevičius 1995.

*boletophila* **(Thomson, 1856).** Pileckis and Monsevičius 1995; Silfverberg 2004.

[*boreella* **Brundin, 1948**]. Known in Sweden, Denmark (Lundberg and Gustafsson 1995; Silfverberg 2004).

*britanniae* **Bernhauer & Scheerpeltz, 1926**. Monsevičius 1985, 1997; Silfverberg 1992, 2004; Pileckis and Monsevičius 1995; Smetana 2004d.

[*brunnea* **(Fabricius, 1798)**]. Known in Denmark, southern Sweden (Lundberg and Gustafsson 1995), Latvia (Telnov 2004), Estonia (Roosileht 2003; Silfverberg 2004), northern Poland (Burakowski et al. 1980).

*canescens* **(Sharp, 1869)**. Monsevičius 1985, 1997; Silfverberg 1992, 2004; Pileckis and Monsevičius 1995; Smetana 2004d.

[*castanoptera* **(Mannerheim, 1830)** = *pertyi* (Heer, 1839)].Known in Latvia (Telnov 2004), Sweden, Denmark, Estonia (Lundberg and Gustafsson 1995; Silfverberg 2004), northwestern Belarus (Alexandrovich et al. 1996).

*cauta* **(Erichson, 1837)**. Monsevičius 1985; Silfverberg 1992, 2004; Pileckis and Monsevičius 1995; Smetana 2004d; Ivinskis et al. 2009.

*celata* **(Erichson, 1837)** = *arenicola* Thomson, 1868.Roubal 1910; Pileckis 1960, 1976a; Monsevičius 1986b, 1997; Silfverberg 1992, 2004; Pileckis and Monsevičius 1995; Smetana 2004d.

*cinnamoptera* **(Thomson, 1856)**. Monsevičius 2000; Ivinskis et al. 2009.

[*clientula* **(Erichson, 1839)**]. Known in Sweden, Denmark (Lundberg and Gustafsson 1995; Silfverberg 2004).

*confusa* **(Märkel, 1844)**. Pileckis 1976a; Silfverberg 1992, 2004; Pileckis and Monsevičius 1995; Monsevičius 2000; Smetana 2004d.

*coriaria* **(Kraatz, 1856)**. Monsevičius 1983; Silfverberg 1992, 2004; Pileckis and Monsevičius 1995; Smetana 2004d.

*crassicornis* **(Fabricius, 1793)** = *repanda* Mulsant & Rey, 1873. Roubal 1910; Pileckis 1960, 1976a; Monsevičius 1987, 1997; Silfverberg 1992, 2004; Pileckis and Monsevičius 1995; Smetana 2004d.

*dadopora* **Thomson, 1867**. Monsevičius 1985, 1987, 1997; Silfverberg 1992, 2004; Pileckis and Monsevičius 1995; Smetana 2004d.

*diversa* **(Sharp, 1869)**. Monsevičius 1985, 1987, 1988a, 1997, 2000; Silfverberg 1992, 2004; Pileckis and Monsevičius 1995; Smetana 2004d.

*divisa* **(Märkel, 1844)**. Pileckis 1976a (*A. divasa*); Bercio and Folwaczny 1979; Silfverberg 1992, 2004; Pileckis and Monsevičius 1995; Monsevičius 1997; Smetana 2004d.

[*ebenina* **(Mulsant & Rey, 1873)**].Known in Sweden, Denmark (Lundberg and Gustafsson 1995), Estonia (Roosileht 2003).

[*eremita* **(Rye, 1866)** = *hercynica* Renkonen, 1936].Known in Sweden, Denmark (Lundberg and Gustafsson 1995), Estonia (Roosileht 2003).

*euryptera* **(Stephens, 1832)**. Roubal 1910; Pileckis 1960, 1976a; Bercio and Folwaczny 1979; Monsevičius 1987, 1997; Silfverberg 1992, 2004; Pileckis and Monsevičius 1995; Tamutis et al. 2004; Smetana 2004d.

[*excellens* **(Kraatz, 1856)**].Known in Denmark, throughout Sweden (Lundberg and Gustafsson 1995; Silfverberg 2004), recently found in Latvia (Cibulskis et al. 2009).

[*excelsa* **Bernhauer, 1911**]. Known in Denmark, southern Sweden (Lundberg and Gustafsson 1995; Silfverberg 2004).

*flavipes* **(Gravenhorst, 1806)**. Pileckis 1976a; Silfverberg 1992, 2004; Pileckis and Monsevičius 1995; Monsevičius 1997, 2000; Smetana 2004d.

*fungi* **(Gravenhorst, 1806)**. Roubal 1910; Pileckis 1960, 1976a; Monsevičius 1986b, 1987, 1997; Dvilevičius et al. 1988; Silfverberg 1992, 2004; Gaidienė 1993; Pileckis and Monsevičius 1995; Smetana 2004d; Ferenca 2006b.

[*fungicola* **(Thomson, 1852)**].Known in Denmark, Estonia, throughout Sweden (Lundberg and Gustafsson 1995; Silfverberg 2004), Latvia (Telnov et al. 2006).

[*fungivora* **(Thomson, 1867)**]. Known in Denmark, southern Sweden (Lundberg and Gustafsson 1995; Silfverberg 2004).

*gagatina* **(Baudi, 1848)**. Pileckis 1976a; Monsevičius 1987, 1997; Silfverberg 1992, 2004; Pileckis and Monsevičius 1995; Smetana 2004d.

[*ganglbaueri* **Brundin, 1948**]. Known in Estonia (Lundberg and Gustafsson 1995; Silfverberg 2004).

[*glabriuscula* **Thomson, 1867**]. Known in Denmark, southern Sweden (Lundberg and Gustafsson 1995; Silfverberg 2004).

[*glabriusculoides* **Strand, 1958**]. Known in Denmark, southern Sweden (Lundberg and Gustafsson 1995; Silfverberg 2004).

*graminicola* **(Gravenhorst, 1806)**. Pileckis 1976a; Monsevičius 1987, 1997; Dvilevičius et al. 1988; Silfverberg 1992, 2004; Gaidienė 1993; Pileckis and Monsevičius 1995; Smetana 2004d.

*harwoodi* **Williams, 1930**. Pileckis and Monsevičius 1995; Monsevičius 2000; Silfverberg 2004; Vorst et al. 2007.

*hepatica* **(Erichson, 1839)**. Ivinskis et al. 2009.

[*hybrida* **(Sharp, 1869)**]. Known in Sweden, Denmark (Lundberg and Gustafsson 1995; Silfverberg 2004).

*hypnorum* **(Kiesenwetter, 1850)**. Monsevičius and Pankevičius 2001; Smetana 2004d.

[*incognita* **(Sharp, 1869)**].Known in in Latvia (Cibulskis et al. 2005), Denmark, Estonia, throughout Sweden (Lundberg and Gustafsson 1995; Silfverberg 2004).

[*indubia* **(Sharp, 1869)**]. Known in in Latvia (Telnov et al. 2008), Denmark, southern Sweden (Lundberg and Gustafsson 1995; Silfverberg 2004).

*inquinula* **Gravenhorst, 1802**. Roubal 1910; Pileckis 1960, 1976a; Silfverberg 1992, 2004; Pileckis and Monsevičius 1995; Smetana 2004d; Ferenca 2006b.

*intermedia* **(Thomson, 1852)**. Monsevičius 1985; Silfverberg 1992, 2004; Pileckis and Monsevičius 1995; Smetana 2004d.

*ischnocera* **Thomson, 1870**. Monsevičius 1985; Silfverberg 1992, 2004; Pileckis and Monsevičius 1995.

[*kerstensi* **G. Benick, 1968**].Known in Denmark (Lundberg and Gustafsson 1995; Silfverberg 2004).

*laevana* **(Mulsant & Rey, 1852)**. Monsevičius 1983; Silfverberg 1992, 2004; Pileckis and Monsevičius 1995; Smetana 2004d.

*laevicauda* **J.R. Sahlberg, 1876**. Monsevičius 1985, 1987, 1997; Silfverberg 1992, 2004; Pileckis and Monsevičius 1995; Smetana 2004d.

[*laticeps* **(Thomson, 1856)**]. Known in Denmark, southern Sweden (Lundberg and Gustafsson 1995; Silfverberg 2004).

*laticollis* **(Stephens, 1832)**. Pileckis 1960, 1976a; Silfverberg 1992, 2004; Gaidienė 1993; Pileckis and Monsevičius 1995; Monsevičius 1997; Smetana 2004d; Ferenca 2006b.

[*liliputana* **(Brisout, 1860)** = *alpina* G. Benick, 1940].Known in Denmark, southern Sweden (Lundberg and Gustafsson 1995; Silfverberg 2004).

*liturata* **(Stephens, 1832)**. Pileckis 1976a; Silfverberg 1992, 2004; Pileckis and Monsevičius 1995; Vorst et al. 2007.

*longicornis* **(Gravenhorst, 1802)**. Roubal 1910; Pileckis 1960, 1976a; Monsevičius 1986b, 1997; Gaidienė 1993; Silfverberg 1992, 2004; Pileckis and Monsevičius 1995; Smetana 2004d.

*macrocera* **(Thomson, 1856)**. Monsevičius 1985, 1997; Silfverberg 1992, 2004; Pileckis and Monsevičius 1995; Smetana 2004d.

[*marcida* **(Erichson, 1837)**]. Known in Denmark, southern Sweden (Lundberg and Gustafsson 1995), Estonia (Silfverberg 2004).

[*minuscula* **(Brisout, 1859)** = *perexigua* (Sharp, 1869)].Known in Denmark, southern Sweden (Lundberg and Gustafsson 1995; Silfverberg 2004).

*myrmecobia* **(Kraatz, 1856)**. Pileckis 1976a; Monsevičius 1986b, 1987, 1997; Silfverberg 1992, 2004; Pileckis and Monsevičius 1995; Vorst et al. 2007.

*monticola* **(Thomson, 1852)**. Monsevičius 1985; Silfverberg 1992, 2004; Pileckis and Monsevičius 1995; Smetana 2004d.

[*negligens* **(Mulsant & Rey, 1873)**]. Known in Denmark, southern Sweden (Lundberg and Gustafsson 1995), Estonia (Silfverberg 2004), Latvia (Telnov et al. 2005).

[*nesslingi* **Bernhauer, 1928**]. Known in Sweden, Denmark (Lundberg and Gustafsson 1995; Silfverberg 2004).

*nidicola* **(Johansen, 1914)**. Silfverberg 1992, 2004; Pileckis and Monsevičius 1995; Monsevičius 1997, 1999, 2000; Smetana 2004d.

*nigra* **(Kraatz, 1856)**. Monsevičius 1985, 1987, 1997; Silfverberg 1992, 2004; Pileckis and Monsevičius 1995.

*nigricornis* **(Thomson, 1852)** = *vaga* (Heer, 1839).Monsevičius 1985, 1987, 1997, 1999, 2000; Silfverberg 1992, 2004; Pileckis and Monsevičius 1995; Smetana 2004d; Ferenca 2006b.

[*nigrifrons* **(Erichson, 1839)**]. Known in Denmark, southern Sweden (Lundberg and Gustafsson 1995; Silfverberg 2004), Poland (Burakowski et al. 1980).

*nigripes* **(Thomson, 1856)**. Pileckis and Monsevičius 1995; Silfverberg 2004.

*nigritula* **(Gravenhorst, 1802)**. Roubal 1910; Pileckis 1960, 1976a; Monsevičius 1986b, 1987, 1997; Silfverberg 1992, 2004; Pileckis and Monsevičius 1995; Tamutis et al. 2004.

[*oblita* **(Erichson, 1839)**]. Known in Denmark, southern Sweden, Estonia (Lundberg and Gustafsson 1995; Silfverberg 2004).

*occulta* **(Erichson, 1837)**. Bercio and Folwaczny 1979; Silfverberg 1992, 2004; Smetana 2004d.

*orbata* **(Erichson, 1837)**. Monsevičius 1985, 1987, 1997; Silfverberg 1992, 2004; Pileckis and Monsevičius 1995; Tamutis et al. 2004; Smetana 2004d.

[*orphana* **(Erichson, 1837)**]. Known in Latvia (Telnov 2004), Sweden, Denmark, Estonia (Lundberg and Gustafsson 1995; Silfverberg 2004), northwestern Belarus (Alexandrovich et al. 1996).

*pallidicornis* **(Thomson, 1856)**. Roubal 1910; Pileckis 1960, 1976a; Silfverberg 1992, 2004; Pileckis and Monsevičius 1995; Smetana 2004d; Vorst et al. 2007; Ivinskis et al. 2009.

*paracrassicornis* **Brundin, 1954**. Monsevičius 1985, 1986b, 1997; Silfverberg 1992, 2004; Pileckis and Monsevičius 1995; Smetana 2004d.

*picipes* **(Thomson, 1856)**. Pileckis 1976a; Silfverberg 1992, 2004; Pileckis and Monsevičius 1995; Monsevičius 1997; Smetana 2004d.

*pilicornis* **(Thomson, 1852)**. Pileckis 1976a; Silfverberg 1992, 2004; Pileckis and Monsevičius 1995; Smetana 2004d.

[*pittionii* **Scheerpeltz, 1950**].Known in northern Belarus (Alexandrovich et al. 1996).

*puncticollis* **G. Benick, 1938**. Ivinskis et al. 2009.

*putrida* **(Kraatz, 1856)**. Monsevičius 1985; Silfverberg 1992, 2004; Pileckis and Monsevičius 1995; Smetana 2004d.

*ravilla* **(Erichson, 1839)** = *angusticollis* (Thomson, 1856).Monsevičius 1985, 1987, 1997; Silfverberg 1992, 2004; Pileckis and Monsevičius 1995; Smetana 2004d.

[*scapularis* **(C.R. Sahlberg, 1831)**]. Known in Sweden, Denmark, Estonia (Lundberg and Gustafsson 1995; Silfverberg 2004), Belarus (Alexandrovich et al. 1996), Poland (Burakowski et al. 1980).

*setigera* **(Sharp, 1869)**. Pileckis 1976a; Silfverberg 1992, 2004; Pileckis and Monsevičius 1995; Monsevičius 2000; Smetana 2004d.

*sodalis* **(Erichson, 1837)**.Pileckis 1976a; Bercio and Folwaczny 1979; Monsevičius 1986b, 1987, 1997; Dvilevičius et al. 1988; Silfverberg 1992, 2004; Pileckis and Monsevičius 1995; Smetana 2004d.

*sordidula* **(Erichson, 1837)**. Bercio and Folwaczny 1979; Monsevičius 1985, 1997; Silfverberg 1992, 2004; Pileckis and Monsevičius 1995; Smetana 2004d.

*strandiella* **Brundin, 1954**. Monsevičius 1985, 1987, 1988a, 1997; Silfverberg 1992, 2004; Pileckis and Monsevičius 1995; Smetana 2004d.

*subglabra* **(Sharp, 1869)**. Monsevičius 1983; Silfverberg 1992, 2004; Pileckis and Monsevičius 1995; Smetana 2004d.

*subsinuata* **(Erichson, 1839)**. Monsevičius 1987; Silfverberg 1992, 2004; Pileckis and Monsevičius 1995; Smetana 2004d.

[*subterranea* **(Mulsant & Rey, 1853)**]. Known in Denmark, southern Sweden (Lundberg and Gustafsson 1995), Estonia (Silfverberg 2004), northeastern Poland (Burakowski et al. 1980).

*subtilis* **(W. Scriba, 1866)**. Pileckis 1976a; Monsevičius 1987; Silfverberg 1992, 2004; Pileckis and Monsevičius 1995; Smetana 2004d.

*talpa* **(Heer, 1841)**. Monsevičius 1987, 1997, 2000; Silfverberg 1992, 2004; Pileckis and Monsevičius 1995; Smetana 2004d.

[*triangulum* **(Kraatz, 1856)**]. Known in Latvia (Telnov 2004), Denmark, southern Sweden (Lundberg and Gustafsson 1995; Silfverberg 2004).

*trinotata* **(Kraatz, 1856)**. Monsevičius 1983, 1997; Silfverberg 1992, 2004; Pileckis and Monsevičius 1995; Smetana 2004d.

[*vestita* **(Gravenhorst, 1806)**]. Known in Denmark, Estonia, southern Sweden (Lundberg and Gustafsson 1995; Silfverberg 2004).

[*xanthopus* **(Thomson, 1856)**].Known in Denmark, Estonia, southern Sweden (Lundberg and Gustafsson 1995; Silfverberg 2004).

[*zosterae* **(Thomson, 1856)**]. Known in Latvia (Telnov 2004), Sweden, Denmark, Estonia (Lundberg and Gustafsson 1995; Silfverberg 2004).

*Anopleta* **Mulsant & Rey, 1874**.

*corvina* **(Thomson, 1856)**. Monsevičius 1985, 1987, 1997; Silfverberg 1992, 2004; Pileckis and Monsevičius 1995; Smetana 2004d.

*depressicollis* **(Fauvel, 1872)**. Monsevičius 1985, 1987, 1988a, 1997, 2000; Silfverberg 1992, 2004; Pileckis and Monsevičius 1995.

[*nitella* **(Brundin, 1948)**]. Known in Sweden, Denmark (Lundberg and Gustafsson 1995; Silfverberg 2004).

[*sodermani* **(Bernhauer, 1931)**]. Known in Denmark, throughout Sweden (Lundberg and Gustafsson 1995; Silfverberg 2004).

*Alevonota* **Thomson, 1858**.

*gracilenta* **(Erichson, 1839**). Pileckis 1976a (*A. splendens*); Pileckis and Monsevičius 1995; Silfverberg 2004.

[*rufotestacea* **(Kraatz, 1856)**].Known in Denmark, southern Sweden (Lundberg and Gustafsson 1995; Silfverberg 2004).

*Alianta* **Thomson, 1858**.

*incana* **(Erichson, 1837)**. Pileckis and Monsevičius 1995; Silfverberg 2004.

*Trichiusa* **Casey, 1893**.

[*immigrata* **Lohse, 1984**].Known in Denmark, southern Sweden (Lundberg and Gustafsson 1995; Silfverberg 2004).

*Pachnida* **Mulsant & Rey, 1874**.

[*nigella* **(Erichson, 1837)**].Recently found in Latvia (Vorst et al. 2007), known in Sweden, Denmark, Estonia (Lundberg and Gustafsson 1995; Silfverberg 2004), Belarus (Alexandrovich et al. 1996).

*Dinaraea* **Thomson, 1858**.

*aequata* **(Erichson, 1837)**. Pileckis 1976a; Silfverberg 1992, 2004; Pileckis and Monsevičius 1995; Monsevičius 1997; Smetana 2004d.

*angustula* **(Gyllenhal, 1810)**. Pileckis 1976a; Silfverberg 1992, 2004; Pileckis and Monsevičius 1995; Monsevičius 1997, 2000; Smetana 2004d.

*arcana* **(Erichson, 1839)**. Silfverberg 1992, 2004; Pileckis and Monsevičius 1995; Monsevičius 2000; Smetana 2004d.

*linearis* **(Gravenhorst, 1802)**. Monsevičius 1985, 1997, 2000; Silfverberg 1992, 2004; Pileckis and Monsevičius 1995.

*Lyprocorrhe* **Thomson, 1859**.

*anceps* **(Erichson, 1837)**. Monsevičius 1985, 1987, 1997, 2000; Silfverberg 1992, 2004; Pileckis and Monsevičius 1995; Smetana 2004d.

*Nehemitropia* **Lohse, 1971**.

*lividipennis* **(Mannerheim, 1830)** = *curvipes* (Stephens, 1832) = *sordida* (Marsham, 1802) nec (Gravenhorst, 1802).Monsevičius 1985, 1987, 1997; Silfverberg 1992, 2004; Pileckis and Monsevičius 1995; Tamutis et al. 2004; Smetana 2004d.

*Acrotona* **Thomson, 1859** = *Calpodota* Mulsant & Rey, 1873.

*aterrima* **(Gravenhorst, 1802)**. Monsevičius 1983, 1986b, 1997; Silfverberg 1992, 2004; Pileckis and Monsevičius 1995; Smetana 2004d.

*benicki* **(Allen, 1940**) = *pusilla* (Brundin, 1952).Monsevičius 1983; Silfverberg 1992, 2004; Pileckis and Monsevičius 1995; Smetana 2004d.

[*convergens* **(Strand, 1958)**]. Known in Denmark, throughout Sweden (Lundberg and Gustafsson 1995; Silfverberg 2004), Belarus (Alexandrovich et al. 1996), recently found in Latvia (Vorst et al. 2007).

*exigua* **(Erichson, 1837)**. Monsevičius 1985, 1997; Silfverberg 1992, 2004; Pileckis and Monsevičius 1995; Smetana 2004d.

*muscorum* **(Brisout, 1860)**. Monsevičius 1985, 1997; Silfverberg 1992, 2004; Pileckis and Monsevičius 1995; Smetana 2004d.

*obfuscata* **(Gravenhorst, 1802)**. Karalius and Monsevičius 1992; Silfverberg 1996, 2004; Pileckis and Monsevičius 1995; Vorst et al. 2007.

*parens* **(Mulsant & Rey, 1852)**. Pileckis 1960; Monsevičius 1985; Silfverberg 1992, 2004; Pileckis and Monsevičius 1995; Smetana 2004d; Ferenca 2006b.

*parvula* **(Mannerheim, 1830)**. Bercio and Folwaczny 1979; Silfverberg 1992, 2004; Smetana 2004d.

*pilosicollis* **(Brundin, 1952)**. Karalius and Monsevičius 1992; Pileckis and Monsevičius 1995; Monsevičius 1997, 2000; Silfverberg 1996, 2004.

*pygmaea* **(Gravenhorst, 1802)**. Pileckis 1976a; Silfverberg 1992, 2004; Pileckis and Monsevičius 1995; Monsevičius 1997; Smetana 2004d.

[*pseudotenera* **(Cameron, 1933)** = *rassii* Muona, 1993].Known in Sweden, Denmark (Lundberg and Gustafsson 1995; Silfverberg 2004).

*sylvicola* **(Kraatz, 1856)** = *planipennis* Thomson, 1855.Monsevičius 1983, 1987, 1997; Silfverberg 1992, 2004; Gaidienė 1993; Pileckis and Monsevičius 1995; Smetana 2004d.

*troglodytes* **(Motschulsky, 1858)** = *consanguinea* (Eppelsheim, 1875).Monsevičius 2000.

*Coprothassa* **Thomson, 1859**.

*melanaria* **(Mannerheim, 1830)**. Pileckis 1976a; Silfverberg 1992, 2004; Pileckis and Monsevičius 1995; Monsevičius 1997; Smetana 2004d.

*Halobrecta* **Thomson, 1858**.

[*flavipes* **Thomson, 1861**]. Known in Denmark, southern Sweden, Estonia (Lundberg and Gustafsson 1995; Silfverberg 2004).

[*puncticeps* **(Thomson, 1852)**]. Known in Denmark, southern Sweden, Estonia (Lundberg and Gustafsson 1995; Silfverberg 2004).

*Amischa* **Thomson, 1858**.

*analis* **(Gravenhorst, 1802)**. # 49.Pileckis 1976a; Monsevičius 1986b, 1987, 1997; Silfverberg 1992, 2004; Gaidienė 1993; Pileckis and Monsevičius 1995; Tamutis et al. 2004; Smetana 2004d.

[*bifoveolata* **(Mannerheim, 1830)** = *cavifrons* (Sharp, 1869)].Known in Denmark, throughout Sweden (Lundberg and Gustafsson 1995; Silfverberg 2004), Poland (Burakowski et al. 1980), northwestern Belarus (Alexandrovich et al. 1996).

*decipiens* **(Sharp, 1869)**. Monsevičius 1983, 1997; Silfverberg 1992, 2004; Pileckis and Monsevičius 1995; Smetana 2004d.

*nigrofusca* **(Stephens, 1832)** = *soror* (Kraatz, 1856) = *similima* (Sharp, 1869) = *sarsi* Munster, 1927.Pileckis 1976a (*Atheta*); Monsevičius 1986b, 1987, 1997; Silfverberg 1992, 2004; Pileckis and Monsevičius 1995; Smetana 2004d.

*Pycnota* **Mulsant & Rey, 1874**.

*paradoxa* **(Mulsant & Rey, 1861)** = *nidorum* (Thomson, 1868).Monsevičius 1997, 1999, 2000.

*Pachyatheta* **Munster, 1930**.

[*cribrata* **(Kraatz, 1856)**].Known in Denmark, throughout Sweden (Lundberg and Gustafsson 1995; Silfverberg 2004).

*Thamiaraea* **Thomson, 1858**.

*cinnamomea* **(Gravenhorst, 1802)**. Roubal 1910; Pileckis 1960, 1976a; Bercio and Folwaczny 1979; Silfverberg 1992, 2004; Pileckis and Monsevičius 1995; Smetana 2004d; Ivinskis et al. 2009.

*hospita* **(Märkel, 1844)**.Ivinskis et al. 2009.

**Falagriini Mulsant & Rey, 1873**.

*Falagria* **Samouelle, 1819**.

*caesa* **Erichson, 1837** = *sulcata* (Paykull, 1789) nec (O.F. Müller, 1776).Bercio and Folwaczny 1979; Monsevičius 1987; Silfverberg 1992, 2004; Pileckis and Monsevičius 1995; Smetana 2004d.

[*nigra* **Gravenhorst, 1802**]. Known in northern Poland (Burakowski et al. 1980).

*sulcatula* **(Gravenhorst, 1806)**. Monsevičius 1983, 1985, 1997; Silfverberg 1992, 2004; Pileckis and Monsevičius 1995; Smetana 2004d.

*Myrmecopora* **Saulcy, 1865**.

[*sulcata* **(Kiesenwetter, 1850)** = *similima* (Wollaston, 1864) = *lohmanderi* Bernhauer, 1927].Known in Sweden, Denmark (Lundberg and Gustafsson 1995; Silfverberg 2004).

*Borboropora* **Kraatz, 1862** = *Aneurota* Casey, 1893.

[*kraatzi* **Fuss, 1862**].Known in southern Sweden (Lundberg and Gustafsson 1995; Silfverberg 2004).

*Bohemiellina* **Machulka, 1941**.

[*flavipennis* **(Cameron, 1921)** = *paradoxa* Machulka, 1941]. Known in Sweden, Denmark (Lundberg and Gustafsson 1995; Silfverberg 2004).

*Cordalia* **Jacobs, 1925**.

*obscura* **(Gravenhorst, 1802)**. Monsevičius 1985; Silfverberg 1992, 2004; Gaidienė 1993; Pileckis and Monsevičius 1995; Smetana 2004d.

*Myrmecocephalus* **MacLeay, 1871**.

[*concinnus* **(Erichson, 1839**) = *longipes* (Wollaston, 1871).Known in Sweden, Denmark (Lundberg and Gustafsson 1995; Silfverberg 2004).

*Falagrioma* **Casey, 1906** = *Falagriusa* Ádám, 1987.

[*thoracica* **(Stephens, 1832)**].Known in Denmark, southern Sweden (Lundberg and Gustafsson 1995; Silfverberg 2004).

*Anaulacaspis* **Ganglbauer, 1895**.

*nigra* **(Gravenhorst, 1802)**. Monsevičius and Pankevičius 2001.

**Lomechusini Fleming, 1821** = Myrmedoniini Thomson, 1867.

*Drusilla* **Leach, 1819** = *Astilbus* Dillwyn, 1829.

*canaliculata* **(Fabricius, 1787)**. Mazurowa and Mazur 1939; Pileckis 1960, 1976a; Monsevičius 1986b, 1987, 1997; Silfverberg 1992, 2004; Gaidienė 1993; Pileckis and Monsevičius 1995; Smetana 2004d; Ferenca 2006b; Lynikienė and Gedminas 2006; Dapkus and Tamutis 2007, 2008a; Vaivilavičius 2008; Ivinskis et al. 2008.

*Zyras* **Stephens, 1835**.

*cognatus* **(Märkel, 1842)**. Pileckis 1976a; Silfverberg 1992, 2004; Pileckis and Monsevičius 1995; Monsevičius 1997; Smetana 2004d; Ivinskis et al. 2008; Ivinskis et al. 2009.

*collaris* **(Paykull, 1800)**. Monsevičius 1983, 1985, 1997; Silfverberg 1992, 2004; Gaidienė 1993; Pileckis and Monsevičius 1995; Smetana 2004d; Ivinskis et al. 2009.

*funestus* **(Gravenhorst, 1806)**.Tamutis et al. 2008; Ivinskis et al. 2008, 2009.

*humeralis* **(Gravenhorst, 1802)**. Pileckis 1976a; Dvilevičius et al. 1988; Silfverberg 1992, 2004; Pileckis and Monsevičius 1995; Monsevičius 1997; Smetana 2004d; Ivinskis et al. 2008, 2009.

*laticollis* **(Märkel, 1842)**. Karalius and Monsevičius 1992; Pileckis and Monsevičius 1995; Silfverberg 1996, 2004; Ivinskis et al. 2008, 2009.

*limbatus* **(Paykull, 1789)**. Roubal 1910; Pileckis 1960, 1976a; Monsevičius 1987, 1997; Dvilevičius et al. 1988; Silfverberg 1992, 2004; Gaidienė 1993; Pileckis and Monsevičius 1995; Smetana 2004d; Ferenca 2006b.

*lugens* **(Gravenhorst, 1802)**. Monsevičius and Jakaitis 1984; Silfverberg 1992, 2004; Pileckis and Monsevičius 1995; Smetana 2004d; Ivinskis et al. 2008, 2009.

*Lomechusoides* **Tottenham, 1939**.

*strumosus* **(Fabricius, 1793)**. Tamutis 2003.

*Lomechusa* **Gravenhorst, 1806** = *Atemeles* Dillwyn, 1829.

*emarginata* **(Paykull, 1789)**. Ferenca et al. 2002; Ferenca 2006a; Ivinskis et al. 2009.

[*paradoxa* **Gravenhorst, 1806**].Known in Denmark, southern Sweden (Lundberg and Gustafsson 1995), Latvia (Telnov 2004), Belarus (Alexandrovich et al. 1996).

**Homolotini Heer, 1839**.

*Gyrophaena* **Mannerheim, 1830**.

*affinis* **Mannerheim, 1830**. Monsevičius and Jakaitis 1984; Silfverberg 1992, 2004; Pileckis and Monsevičius 1995; Monsevičius 1997; Smetana 2004d.

*angustata* **(Stephens, 1832)** = *manca* Erichson, 1839.Monsevičius 1985, 1987, 1997; Silfverberg 1992, 2004; Pileckis and Monsevičius 1995; Smetana 2004d.

*bihamata* **Thomson, 1867**. Monsevičius 1983, 1997; Silfverberg 1992, 2004; Pileckis and Monsevičius 1995; Smetana 2004d.

*boleti* **(Linnaeus, 1758)**. Monsevičius 1985, 1987, 1997; Silfverberg 1992, 2004; Pileckis and Monsevičius 1995; Smetana 2004d.

[*congrua* **Erichson, 1837**]. Known in Latvia (Telnov 2004), Sweden, Denmark (Lundberg and Gustafsson 1995; Silfverberg 2004), Estonia (Roosileht 2003) northern Belarus (Alexandrovich et al. 1996), norteastern Poland (Burakowski et al. 1980).

*fasciata* **(Marsham, 1802)** = *laevipennis* Kraatz, 1856.Monsevičius 1985, 1997; Silfverberg 1992, 2004; Pileckis and Monsevičius 1995; Smetana 2004d.

*gentilis* **Erichson, 1839**. Pileckis 1976a; Silfverberg 1992, 2004; Pileckis and Monsevičius 1995; Monsevičius 1997; Smetana 2004d.

[*hanseni* **Strand, 1839**]. Known in Denmark (Lundberg and Gustafsson 1995; Silfverberg 2004).

*joyi* **Wendeler, 1924**. Karalius and Monsevičius 1992; Pileckis and Monsevičius 1995; Silfverberg 1996, 2004; Ferenca et al. 2006; Ivinskis et al. 2007a.

*joyioides* **Wüsthoff, 1937**. Monsevičius 1985, 1997; Silfverberg 1992, 2004; Pileckis and Monsevičius 1995; Smetana 2004d; Vorst et al. 2007.

*lucidula* **Erichson, 1837**. Ivinskis et al. 2009.

*minima* **Erichson, 1837**. Pileckis 1976a; Monsevičius 1987, 1997; Silfverberg 1992, 2004; Pileckis and Monsevičius 1995; Smetana 2004d.

*munsteri* **Strand, 1935**. Monsevičius 1985, 1997; Silfverberg 1992, 2004; Pileckis and Monsevičius 1995; Smetana 2004d; Vorst et al. 2007.

*nana* **(Paykull, 1800)**. Pileckis 1976a; Silfverberg 1992, 2004; Gaidienė 1993; Pileckis and Monsevičius 1995; Monsevičius 1997; Smetana 2004d.

*polita* **Gravenhorst, 1802**. Bercio and Folwaczny 1979.

*poweri* **Crotch, 1866**. Monsevičius 1985; Silfverberg 1992, 2004; Pileckis and Monsevičius 1995; Smetana 2004d.

[*pseudonana* **Strand, 1939**]. Known in southern Sweden (Lundberg and Gustafsson 1995).

*pulchella* **Heer, 1839**.Monsevičius and Pankevičius 2001; Ferenca et al. 2006, 2007; Ivinskis et al. 2009.

[*rousi* **Dvořák, 1966**].Known in southern Sweden (Lundberg and Gustafsson 1995).

*rugipennis* **Mulsant & Rey, 1861**.Tamutis and Ferenca 2006; Ferenca et al. 2006; Ivinskis et al. 2007a.

*strictula* **Erichson, 1839**. Roubal 1910; Pileckis 1960, 1976a; Silfverberg 1992, 2004; Pileckis and Monsevičius 1995; Smetana 2004d; Vorst et al. 2007.

*williamsi* **Strand, 1935**. Monsevičius and Pankevičius 2001.

*Agaricochara* **Kraatz 1856**.

[*latissima* **(Stephens, 1832)** = *laevicollis* (Kraatz, 1854)].Known in Sweden, Denmark (Lundberg and Gustafsson 1995; Silfverberg 2004), Poland (Burakowski et al. 1980).

*Encephalus* **Stephens, 1832**.

*complicans* **Stephens, 1832**. Barševskis 2001a; Tamutis 2003; Silfverberg 2004.

*Bolitochara* **Mannerheim, 1830**.

[*bella* **Märkel, 1844**]. Known in Denmark (Silfverberg 2004), Poland (Burakowski et al. 1980).

*lucida* **(Gravenhorst, 1802)**. Gaidienė 1993; Silfverberg 2004.

*mulsanti* **Sharp, 1875**. Monsevičius 1983, 1997; Silfverberg 1992, 2004; Pileckis and Monsevičius 1995; Smetana 2004d.

*obliqua* **Erichson, 1837**. Pileckis 1976a; Silfverberg 1992, 2004; Gaidienė 1993; Pileckis and Monsevičius 1995; Monsevičius 1997; Smetana 2004d.

*pulchra* **(Gravenhorst, 1806)** = *lunulata* (Paykull, 1789) nec (Linnaeus, 1761).Bercio and Folwaczny 1979; Monsevičius 1983, 1997; Silfverberg 1992, 2004; Pileckis and Monsevičius 1995; Smetana 2004d.

*Phymatura* **J.R. Sahlberg, 1876**.

*brevicollis* **(Kraatz, 1856)**. Tenenbaum 1923, 1931; Pileckis 1968b, 1976a; Monsevičius 1987, 1997; Silfverberg 1992, 2004; Pileckis and Monsevičius 1995.

*Leptusa* **Kraatz, 1856** = *Sipalia* auct. nec Mulsant & Rey, 1853; *Pachygluta* Thomson, 1858.

*fumida* **(Erichson, 1839)**. Monsevičius 1985, 1987, 1997; Silfverberg 1992, 2004; Pileckis and Monsevičius 1995; Smetana 2004d.

[*norvegica* **Strand, 1941**]. Known in Latvia (Telnov 2004), Sweden, Denmark (Lundberg and Gustafsson 1995; Silfverberg 2004).

*pulchella* **(Mannerheim, 1830)** = *saalasi* Kangas, 1952.Monsevičius and Jakaitis 1984; Monsevičius 1987, 1997; Silfverberg 1992, 2004; Gaidienė 1993; Pileckis and Monsevičius 1995.

[*ruficollis* **(Erichson, 1839)**].Known in Latvia (Telnov 2004), Sweden, Denmark (Lundberg and Gustafsson 1995; Silfverberg 2004), Estonia (Roosileht 2003), Belarus (Alexandrovich et al. 1996), northeastern Poland (Burakowski et al. 1980).

*Tachyusida* **Mulsant & Rey, 1872**.

*gracilis* **(Erichson, 1837)**. Pileckis 1976a; Silfverberg 1992, 2004; Pileckis and Monsevičius 1995.

*Euryusa* **Erichson, 1837**.

*castanoptera* **Kraatz, 1856**. Monsevičius 1985, 1997; Silfverberg 1992, 2004; Pileckis and Monsevičius 1995; Smetana 2004d.

[*coarctata* **Märkel, 1844**]. Known in southern Sweden (Lundberg and Gustafsson 1995).

[*optabilis* **Heer, 1839**]. Known in Sweden (Lundberg and Gustafsson 1995), Poland (Burakowski et al. 1980).

[*sinuata* **Erichson, 1837**]. Known in Denmark, southern Sweden (Lundberg and Gustafsson 1995), Belarus (Alexandrovich et al. 1996), Poland (Burakowski et al. 1980).

*Heterota* **Mulsant & Rey, 1874**.

[*plumbea* **(Waterhouse, 1858)**]. Known in southern Sweden (Lundberg and Gustafsson 1995).

*Silusa* **Erichson, 1837**.

*rubiginosa* **Erichson, 1837**. Pileckis 1976a; Silfverberg 1992, 2004; Pileckis and Monsevičius 1995; Smetana 2004d.

[*rubra* **Erichson , 1839**]. Known in Estonia (Silfverberg 2004), Poland (Burakowski et al. 1980).

*Anomognathus* **Solier, 1849** = *Theetura* Thomson, 1858.

*cuspidatus* **(Erichson, 1839)**. Monsevičius and Jakaitis 1984; Silfverberg 1992, 2004; Pileckis and Monsevičius 1995; Smetana 2004d.

*Thecturota* **Casey, 1893** = *Pragensiella* Machulka, 1941.

[*marchii* **(Dodero, 1922)**]. Known in Sweden, Denmark, Estonia (Lundberg and Gustafsson 1995; Silfverberg 2004).

*Homalota* **Mannerheim, 1830**.

*plana* **(Gyllenhal, 1810**). Pileckis 1976a (*H. cinnamomea*); Monsevičius and Jakaitis 1984; Silfverberg 1992, 2004; Pileckis and Monsevičius 1995; Monsevičius 1997; Smetana 2004d.

*Pseudomicrodota* **Machulka, 1935**.

[*jelineki* **(Krása, 1914)** = *flavicollis* (Brundin, 1948)]. Known in southern Sweden (Lundberg and Gustafsson 1995), Denmark (Silfverberg 2004), northern Belarus (Alexandrovich et al. 1996).

*Rhopalocerina* **Reitter, 1909**.

[*clavigera* **(W. Scriba, 1859)**]. Known in Poland (Burakowski et al. 1980), Sweden (Lundberg and Gustafsson 1995).

*Cyphaea* **Fauvel, 1863**.

[*curtula* **(Erichson, 1837)**]. Known in Poland (Burakowski et al. 1980), Latvia (Telnov et al. 2005), Estonia, Denmark, Sweden (Lundberg and Gustafsson 1995).

**Placusini Mulsant & Rey, 1871**.

*Placusa* **Erichson, 1837**.

*atrata* **(Mannerheim, 1830)**. Monsevičius 1985, 1987, 1997; Silfverberg 1992, 2004; Pileckis and Monsevičius 1995; Smetana 2004d; Ivinskis et al. 2009.

*complanata* **Erichson, 1839**. Monsevičius and Jakaitis 1984; Silfverberg 1992, 2004; Pileckis and Monsevičius 1995.

*depressa* **Mäklin, 1845**. Monsevičius and Jakaitis 1984; Monsevičius 1987, 1997; Silfverberg 1992, 2004; Pileckis and Monsevičius 1995; Smetana 2004d.

*incompleta* **Sjöberg, 1934**. Monsevičius and Jakaitis 1984; Silfverberg 1992, 2004; Pileckis and Monsevičius 1995; Monsevičius 1997; Smetana 2004d; Ivinskis et al. 2009.

[*pumilio* **(Gravenhorst, 1802)**].Known in northeastern Poland (Burakowski et al. 1980), Denmark, southern Sweden (Lundberg and Gustafsson 1995; Silfverberg 1992, 2004), Belarus (Alexandrovich et al. 1996).

*tachyporoides* **(Waltl, 1838)**. Monsevičius 1985, 1987, 1997; Silfverberg 1992, 2004; Pileckis and Monsevičius 1995; Smetana 2004d.

**Autaliini Thomson, 1859**.

*Autalia* **Leach, 1819**.

*impressa* **(Olivier, 1795)**.Monsevičius and Pankevičius 2001

*longicornis* **Scheerpeltz, 1947**.Ivinskis et al. 2009.

*puncticollis* **Sharp, 1864**. Pileckis 1976a; Silfverberg 1992, 2004; Pileckis and Monsevičius 1995; Smetana 2004d.

*rivularis* **(Gravenhorst, 1802)**. Monsevičius 1985, 1997; Silfverberg 1992, 2004; Pileckis and Monsevičius 1995.

**Phytosini Thomson, 1867**.

*Phytosus* **Curtis, 1838**.

*balticus* **Kraatz, 1859**. Pileckis 1976a; Silfverberg 1992, 2004; Pileckis and Monsevičius 1995; Smetana 2004d.

[*spinifer* **Curtis, 1838**].Known in Denmark, southern Sweden (Lundberg and Gustafsson 1995; Silfverberg 1992, 2004).

*Arena* **Fauvel, 1862**.

[*tabida* **(Kiesenwetter, 1850)**].Known in Denmark (Silfverberg 1992, 2004).

**Diglottini Jakobson, 1909**.

*Diglotta* **Champion, 1887**.

[*mersa* **(Haliday, 1837)** = *submarina* (Fairmaire & Laboulbène, 1856)].Known in Denmark (Lundberg and Gustafsson 1995; Silfverberg 1992, 2004).

[*sinuaticollis* **(Mulsant & Rey, 1870)** = *submarina* auct. nec (Fairmaire & Laboulbène, 1856)].Known in Sweden, Denmark (Lundberg and Gustafsson 1995; Silfverberg 1992, 2004).

**Hygronomini Thomson, 1859**.

*Hygronoma* **Erichson, 1837**.

*dimidiata* **(Gravenhorst, 1806)**. Monsevičius 1985, 1997; Silfverberg 1992, 2004; Pileckis and Monsevičius 1995; Smetana 2004d; Ferenca et al. 2006; Ivinskis et al. 2007a.

**Hypocyphtini Laporte, 1835** = Oligotini Thomson, 1859.

*Holobus* **Solier, 1849**.

[*apicatus* **(Erichson, 1837)**].Known in Latvia (Telnov et al. 2008), Poland (Burakowski et al. 1980), throughout Sweden (Lundberg and Gustafsson 1995), Denmark, Estonia (Silfverberg 1992, 2004).

*flavicornis* **Lacordaire, 1835**. Pileckis 1976a; Silfverberg 1992, 2004; Pileckis and Monsevičius 1995 (*Oligota*); Smetana 2004d.

*Oligota* **Mannerheim, 1830**.

*granaria* **Erichson, 1837**. Pileckis 1976a; Silfverberg 1992, 2004; Pileckis and Monsevičius 1995.

[*inflata* **(Mannerheim, 1830)**].Known in Latvia (Telnov 2004), throughout Sweden (Lundberg and Gustafsson, 1995), Denmark, Estonia (Silfverberg 1992, 2004), Belarus (Alexandrovich et al. 1996).

[*parva* **Kraatz, 1862**]. Known in northeastern Poland (Burakowski et al. 1980), throughout Sweden (Lundberg and Gustafsson 1995), Denmark, Estonia (Silfverberg 1992, 2004).

[*picipes* **(Stephens, 1832)**].Known in Denmark (Silfverberg 1992, 2004).

[*pumilio* **Kiesenwetter, 1858**].Known in Poland (Burakowski et al. 1980), Latvia (Cibulskis and Petrova 2002), Denmark, southern Sweden (Lundberg and Gustafsson 1995; Silfverberg 1992, 2004), Belarus (Alexandrovich et al. 1996).

[*punctulata* **Heer, 1839** = *ruficornis* Sharp, 1870].Known in Poland (Burakowski et al. 1980), Latvia (Cibulskis and Petrova 2002), Denmark, Sweden (Lundberg and Gustafsson 1995).

*pusillima* **(Gravenhorst, 1806)** = *atomaria* Erichson, 1837 = *intermedia* Kangas, 1938.Bercio and Folwaczny 1979; Silfverberg 1992, 2004; Smetana 2004d.

*Cypha* **Samouelle, 1819** = *Hypocyphtus* Gyllenhal, 1827.

[*discoidea* **(Erichson, 1839)**]. Known in Latvia (Telnov 2004), Denmark, southern Sweden (Lundberg and Gustafsson 1995; Silfverberg 1992, 2004).

[*hanseni* **(Palm, 1949)**].Known in Denmark, southern Sweden, (Lundberg and Gustafsson 1995; Silfverberg 1992, 2004).

[*imitator* **(Luze, 1902)**].Known in Latvia (Telnov 2004), Denmark, southern Sweden, (Lundberg and Gustafsson 1995; Silfverberg 1992, 2004).

*laeviuscula* **(Mannerheim, 1830)**. Pileckis 1976a; Silfverberg 1992, 2004; Pileckis and Monsevičius 1995; Smetana 2004d.

[*longicornis* **(Paykull, 1800)**].Known in Denmark, Estonia, southern Sweden (Lundberg and Gustafsson 1995; Silfverberg 1992, 2004), Belarus (Alexandrovich et al. 1996).

[*nitida* **(Palm, 1936)**].Known in Sweden, Denmark (Lundberg and Gustafsson 1995; Silfverberg 1992, 2004).

*pulicaria* **(Erichson, 1839)**. Pileckis 1976a; Silfverberg 1992, 2004; Pileckis and Monsevičius 1995; Smetana 2004d.

[*punctum* **(Motschulsky, 1857)**].Known in Sweden, Denmark (Lundberg and Gustafsson 1995; Silfverberg 1992, 2004).

*seminulum* **(Erichson, 1839)**. Pileckis 1976a; Silfverberg 1992, 2004; Pileckis and Monsevičius 1995; Smetana 2004d.

[*suecica* **(Palm, 1936)**].Recently found in Latvia (Vorst et al. 2007), known in Sweden, Denmark (Lundberg and Gustafsson 1995; Silfverberg 1992, 2004).

[*tarsalis* **(Luze, 1902)**].Known in throughout Sweden (Lundberg and Gustafsson 1995), Denmark, Estonia (Silfverberg 1992, 2004).

**Myllaenini Ganglbauer, 1895**.

*Myllaena* **Erichson, 1837**.

*brevicornis* **(Matthews, 1838)**. Pileckis 1976a; Silfverberg 1992, 2004; Pileckis and Monsevičius 1995; Smetana 2004d.

*dubia* **(Gravenhorst, 1806)**. Monsevičius 1985, 1986b, 1997; Silfverberg 1992, 2004; Pileckis and Monsevičius 1995; Smetana 2004d.

[*elongata* **(Matthews, 1838)**].Known in (Poland Burakowski et al. 1980), Estonia, Denmark (Lundberg and Gustafsson 1995; Silfverberg 1992, 2004).

[*gracilis* **(Matthews, 1838)**].Recently found in Latvia (Vorst et al. 2007), known in northern Poland (Burakowski et al. 1980), Sweden, Denmark, Estonia (Lundberg and Gustafsson 1995; Silfverberg 1992, 2004).

*infuscata* **Kraatz, 1853**. Monsevičius 1983, 1987, 1997; Silfverberg 1992, 2004; Pileckis and Monsevičius 1995; Smetana 2004d.

*intermedia* **Erichson, 1837**. Pileckis 1976a; Monsevičius 1986b, 1987, 1997; Silfverberg 1992, 2004; Gaidienė 1993; Pileckis and Monsevičius 1995; Smetana 2004d.

*kraatzi* **Sharp, 1871**. Monsevičius 1985, 1986b, 1997; Silfverberg 1992, 2004; Pileckis and Monsevičius 1995; Smetana 2004d.

[*masoni* **A. Matthews, 1883**].Known in Denmark (Silfverberg 2004).

*minuta* **(Gravenhorst, 1806)**. Monsevičius 1983, 1986b, 1987, 1997; Silfverberg 1992, 2004; Pileckis and Monsevičius 1995; Smetana 2004d.

*Pronomaea* **Erichson, 1837**.

*rostrata* **Erichson, 1837**. Bercio and Folwaczny 1979.

**Gymnusini Heer, 1839**.

*Gymnusa* **Gravenhorst, 1806**.

*brevicollis* **(Paykull, 1800)**. Pileckis 1976a; Bercio and Folwaczny 1979; Monsevičius 1986b, 1997; Silfverberg 1992, 2004; Pileckis and Monsevičius 1995; Smetana 2004d.

**Deinopsini Sharp, 1883**.

*Deinopsis* **Matthews, 1838**.

*erosa* **(Stephens, 1832)**. Monsevičius 1983, 1997; Silfverberg 1992, 2004; Pileckis and Monsevičius 1995; Smetana 2004d.

**Scaphidiinae Latreille, 1806**. (Scaphidiidae)

**Scaphidiini Latreille, 1806**.

*Scaphidium* **Olivier, 1790**.

*quadrimaculatum* **Olivier, 1790**. Pileckis 1962, 1963b, 1976a; Silfverberg 1992, 2004; Gaidienė 1993; Pileckis and Monsevičius 1995; Monsevičius 1997; Šablevičius 2000b, 2011; Tamutis and Zolubas 2001; Löbl 2004b; Vaivilavičius 2008; Alonso-Zarazaga 2009a.

**Scaphisomatini Casey, 1893**.

*Scaphisoma* **Leach, 1815**.

*agaricinum* **(Linnaeus, 1758)**. Roubal 1910; Pileckis 1960, 1976a; Silfverberg 1992, 2004; Gaidienė 1993; Pileckis and Monsevičius 1995; Monsevičius 1997; Šablevičius 2000b, 2011; Tamutis and Zolubas 2001; Löbl 2004b; Vaivilavičius 2008; Alonso-Zarazaga 2009a; Ostrauskas and Ferenca 2010.

*assimile* **Erichson, 1845**. Monsevičius 1988b, 1997; Silfverberg 1992, 2004; Pileckis and Monsevičius 1995; Löbl 2004b; Alonso-Zarazaga 2009a.

[*balcanicum* **Tamanini, 1954**].Known in southern Sweden (Lundberg and Gustafsson 195), Poland (Burakowski et al. 1978), southern Belarus (Alexandrovich et al. 1996).

*boleti* **(Panzer, 1793)**. Pileckis 1976a; Silfverberg 1992, 2004; Gaidienė 1993; Pileckis and Monsevičius 1995; Monsevičius 1997; Tamutis and Zolubas 2001; Šablevičius 2004; Alonso-Zarazaga 2009a.

*boreale* **Lundbland, 1952**. Löbl 2004b; Alonso-Zarazaga 2009a.

*inopinatum* **Löbl, 1967**. Monsevičius 1988b, 1997; Silfverberg 1992, 2004; Pileckis and Monsevičius 1995; Löbl 2004b; Alonso-Zarazaga 2009a.

[*obenbergeri* **Löbl, 1952**].Known in northwestern Belarus (Alexandrovich et al. 1996), Poland (Burakowski et al. 1978).

*subalpinum* **Reitter, 1881**.Löbl 2004b; Alonso-Zarazaga 2009a.

*Caryoscapha* **Ganglbauer, 1902**.

[*limbatum* **(Erichson, 1845)**]. Known in northwestern Belarus (Alexandrovich et al. 1996), Poland (Burakowski et al. 1978).

**Oxytelinae Fleming, 1821**.

**Euphaniini Reitter, 1909**.

*Syntomium* **Curtis, 1828**.

*aeneum* **(O.F. Müller, 1821)**. Monsevičius 1985, 1997; Silfverberg 1992, 2004; Pileckis and Monsevičius 1995; Smetana 2004f; Alonso-Zarazaga 2009a.

*Deleaster* **Erichson, 1839**.

*dichrous* **(Gravenhorst, 1802)**. Pileckis 1960, 1976a; Silfverberg 1992, 2004; Gaidienė 1993; Pileckis and Monsevičius 1995; Smetana 2004f; Ferenca 2006b; Alekseev 2008a; Alonso-Zarazaga 2009a.

**Coprophilini Heer, 1839**.

*Coprophilus* **Latreille, 1829** = *Elonium* Samouelle, 1819.

*striatulus* **(Fabricius, 1793)**. Gaidienė 1993; Monsevičius and Pankevičius 2001; Silfverberg 2004; Smetana 2004f; Vaivilavičius 2008; Alonso-Zarazaga 2009a.

**Planeustomini Jacquelin du Val, 1857**.

*Manda* **Blackwelder, 1952** = *Acrognathus* Erichson, 1839, nec Agassiz, 1826.

*mandibularis* **(Gyllenhal, 1827)**. Barševskis 2001; Silfverberg 2004.

*Planeustomus* **Jacquelin du Val, 1857**.

[*palpalis* **(Erichson, 1839)**].Known in Denmark, southern Sweden (Lundberg and Gustafsson 1995), northeastern Poland (Burakowski et al. 1979).

**Oxytelini Fleming, 1821**.

*Thinobius* **Kiesenwetter, 1844**.

[*brevipennis* **Kiesenwetter, 1850**].Recently found in Latvia (Cibulskis et al. 2009), known in Sweden, Denmark, Estonia (Lundberg and Gustafsson 1995; Silfverberg 1992, 2004), northern Poland (Burakowski et al. 1979).

[*ciliatus* **Kiesenwetter, 1844** = *praetor* Smetana, 1959].Known in Denmark, Sweden (Lundberg and Gustafsson 1995).

[*crinifer crinifer* **Smetana, 1959**]. Known in Denmark (Lundberg and Gustafsson 1995).

[*flagellatus* **Lohse, 1984**]. Known in Poland (Staniec 2002), Latvia (Vorst et al. 2007), Sweden (Lundberg and Gustafsson 1995).

[*longipennis* **Heer, 1841**]. Known in northeastern Poland (Burakowski et al. 1979).

*Thinodromus* **Kraatz, 1857**.

[*arcuatus* **(Stephens, 1834)**]. Recently found in Latvia (Vorst et al. 2007), known in northern Poland (Burakowski et al. 1979), southern Sweden, Denmark, Estonia (Lundberg and Gustafsson, 1995; Silfverberg 1992, 2004).

*Carpelimus* **Leach, 1819** = *Trogophloeus* Mannerheim, 1830.

*bilineatus* **Stephens, 1834**. Pileckis 1976a; Silfverberg 1992, 2004; Gaidienė 1993; Pileckis and Monsevičius 1995; Monsevičius 1998; Smetana 2004f; Alonso-Zarazaga 2009a.

*corticinus* **(Gravenhorst, 1806)**. Monsevičius 1983, 1986b, 1987, 1997; Silfverberg 1992, 2004; Gaidienė 1993; Pileckis and Monsevičius 1995; Smetana 2004f; Alonso-Zarazaga 2009a.

*despectus* **(Baudi, 1870)**.Smetana 2004f; Alonso-Zarazaga 2009a.

*elongatulus* **(Erichson, 1839)**. Monsevičius 1983, 1986b, 1997; Silfverberg 1992, 2004; Gaidienė 1993; Pileckis and Monsevičius 1995; Smetana 2004f; Alonso-Zarazaga 2009a.

[*erichsoni* **(Sharp, 1871)** = *augustae* (Bernhauer, 1901) = *bilineatus* (Erichson, 1839)].Known in Latvia (Telnov 2004).

[*exiguus* **Erichson, 1839**].Known in Latvia (Telnov 2004), northern Belarus (Alexandrovich et al. 1996), Poland (Burakowski et al. 1979).

*foveolatus* **C.R. Sahlberg, 1832**.Tamutis 2002b, c.

*fuliginosus* **(Gravenhorst, 1802)**. Pileckis 1976a; Silfverberg 1992, 2004; Pileckis and Monsevičius 1995; Smetana 2004f; Alonso-Zarazaga 2009a.

*gracilis* **(Mannerheim, 1830)** = *graciliformis* Konzelmann & Lohse, 1981.Pileckis 1976a; Silfverberg 1992, 2004; Pileckis and Monsevičius 1995; Smetana 2004f; Alonso-Zarazaga 2009a.

[*halophilus* **(Kiesenwetter, 1844)**].Known in Denmark, southern Sweden, Estonia (Lundberg and Gustafsson 1995; Silfverberg 1992, 2004), Poland (Burakowski et al. 1979).

[*heidenreichi* **(G. Benick, 1934)**].Known in Latvia (Telnov 2004); southern Sweden (Lundberg and Gustafsson 1995; Silfverberg 1992, 2004).

*impressus* **(Lacordaire, 1835)**.Monsevičius and Pankevičius 2001.

[*lindrothi* **(Palm, 1943)**].Known in Latvia (Telnov 2004), Sweden, Denmark, Estonia (Lundberg and Gustafsson 1995; Silfverberg 1992, 2004).

*nitidus* **(Baudi, 1848)**. Monsevičius 1985, 1997; Silfverberg 1992, 2004; Pileckis and Monsevičius 1995; Smetana 2004f; Alonso-Zarazaga 2009a.

*obesus* **(Kiesenwetter, 1844)**. Monsevičius 1985; Silfverberg 1992, 2004; Pileckis and Monsevičius 1995; Smetana 2004f; Alonso-Zarazaga 2009a; Ivinskis. 2009.

*pusillus* **(Gravenhorst, 1802)** = *lindbergi* (Scheerpeltz, 1937).Pileckis 1976a; Bercio and Folwaczny 1979; Silfverberg 1992, 2004; Pileckis and Monsevičius 1995; Tamutis 2002b; Tamutis et al. 2004; Smetana 2004f; Alonso-Zarazaga 2009a.

*rivularis* **(Motschulsky, 1860)**. Monsevičius 1983, 1987, 1997; Silfverberg 1992, 2004; Pileckis and Monsevičius 1995; Smetana 2004f; Alonso-Zarazaga 2009a.

[*schneideri* **(Ganglbauer, 1895)**].Known in southern Sweden (Lundberg and Gustafsson 1995), Denmark (Silfverberg 1992, 2004).

[*similis* **(Smetana, 1967)**]. Known in Latvia (Vorst et al. 2007), Poland (Staniec 2000).

[*subterraneus* **(Smetana, 1960)**].Known in Latvia (Telnov 2004). 

[*subtilicornis* **(Roubal, 1946)** = *strandi* (Scheerpeltz, 1950)].Known in Latvia (Cibulskis 2002; Silfverberg 2004); Estonia (Silfverberg 2004), Sweden (Lundberg and Gustafsson 1995).

*subtilis* **(Erichson, 1839)**. Silfverberg 1992, 2004; Smetana 2004f; Vorst et al. 2007; Alonso-Zarazaga 2009a.

*Aploderus* **Stephens, 1833**.

*caelatus* **(Gravenhorst, 1802)**. Pileckis 1960, 1976a; Silfverberg 1992, 2004; Pileckis and Monsevičius 1995; Monsevičius 1997; Tamutis 1999, 2002c, Smetana 2004f; Ferenca 2006b; Alonso-Zarazaga 2009a.

[*caesus* **(Erichson, 1839)**].Known in Sweden, Denmark, Estonia, Latvia (Silfverberg 2004), northern Poland (Burakowski et al. 1979).

*Oxytelus* **Gravenhorst, 1802**.

*fulvipes* **Erichson, 1839**.Pileckis 1976a; Monsevičius 1986b, 1987, 1997; Silfverberg 1992, 2004; Pileckis and Monsevičius 1995; Smetana 2004f; Alonso-Zarazaga 2009a.

*laqueatus* **(Marsham, 1802)**. Roubal 1910; Pileckis 1960, 1976a; Silfverberg 1992, 2004; Gaidienė 1993; Pileckis and Monsevičius 1995; Monsevičius 1997; Smetana 2004f; Alonso-Zarazaga 2009a.

*migrator* **Fauvel, 1904**. Monsevičius 1985, 1987, 1988a, 1997; Silfverberg 1992, 2004; Pileckis and Monsevičius 1995; Smetana 2004f; Alonso-Zarazaga 2009a.

*piceus* **(Linnaeus, 1767)**. Mazurowa and Mazur 1939; Pileckis 1960, 1976a; Bercio and Folwaczny 1979; Monsevičius 1987, 1997; Silfverberg 1992, 2004; Gaidienė 1993; Pileckis and Monsevičius 1995; Smetana 2004f; Alonso-Zarazaga 2009a.

*sculptus* **Gravenhorst, 1806**. Pileckis 1960, 1976a; Bercio and Folwaczny 1979; Silfverberg 1992, 2004; Gaidienė 1993; Pileckis and Monsevičius 1995; Smetana 2004f; Ferenca 2006b; Alonso-Zarazaga 2009a.

*Anotylus* **Thomson, 1859**.

[*affinis* **(Czwalina, 1870)**].Known in Latvia (Telnov 2004), Estonia (Silfverberg 1992, 2004).

[*clavatus* **(A. Strand, 1946)**].Known in Latvia (Telnov. 2006), throughout Sweden (Lundberg and Gustafsson 1995).

[*clypeonitens* **(Pandellé, 1867)**].Know in Latvia (Telnov 2004), Denmark, Estonia (Silfverberg 1992, 2004), Poland (Burakowski et al. 1979), northwestern Belarus (Alexandrovich et al. 1996).

*complanatus* **(Erichson, 1839)**. Pileckis 1976a; Silfverberg 1992, 2004; Pileckis and Monsevičius 1995; Smetana 2004f; Alonso-Zarazaga 2009a.

*fairmairei* **(Pandellé, 1867)**. Tenenbaum 1923, 1931; Pileckis 1968b, 1976a; Silfverberg 1992, 2004; Pileckis and Monsevičius 1995; Monsevičius 1997; Smetana 2004f; Ferenca et al. 2006; Alonso-Zarazaga 2009a.

[*hamatus* **(Fairmaire & Laboulbène, 1856)**].Know in Latvia (Telnov 2004), Denmark, Estonia (Lundberg and Gustafsson 1995; Silfverberg 1992, 2004), northern Poland (Burakowski et al. 1979).

*insecatus* **(Gravenhorst, 1806)**. Monsevičius and Pankevičius 2001.

[*intricatus* **(Erichson, 1840)**].Known in Latvia (Telnov 2004), Poland (Burakowski et al. 1979).

*inustus* **(Gravenhorst, 1806)**. Pileckis 1976a; Silfverberg 1992, 2004; Pileckis and Monsevičius 1995; Smetana 2004f; Alonso-Zarazaga 2009a.

[*maritimus* **Thomson, 1861** = *perrisii* (Fauvel, 1862)]. Known in Latvia (Telnov 2004), Denmark, southern Sweden (Lundberg and Gustafsson 1995).

[*mutator* **(Lohse, 1963)**].Known in Denmark, Sweden (Silfverberg 2004), Poland (Burakowski et al. 1979).

*nitidulus* **Gravenhorst, 1802**. Roubal 1910; Mazurowa and Mazur 1939; Pileckis 1960, 1976a; Silfverberg 1992, 2004; Gaidienė 1993; Pileckis and Monsevičius 1995; Monsevičius 1997; Tamutis 2002b; Smetana 2004f; Alonso-Zarazaga 2009a.

[*politus* **(Erichson, 1840)**].Known in Latvia (Telnov 2004), Poland (Alonso-Zarazaga 2009a).

*rugifrons* **(Hochhuth, 1849)**. Monsevičius 1985, 1997; Silfverberg 1992, 2004; Pileckis and Monsevičius 1995; Smetana 2004f; Alonso-Zarazaga 2009a.

*rugosus* **(Fabricius, 1775)**. Pileckis 1960, 1976a; Monsevičius 1986b, 1987, 1997; Dvilevičius et al. 1988; Silfverberg 1992, 2004; Gaidienė 1993; Pileckis and Monsevičius 1995; Tamutis and Zolubas 2001; Tamutis 2002b; Tamutis et al. 2004; Smetana 2004f; Ferenca 2006b; Dapkus and Tamutis 2008a; Alekseev 2008a; Alonso-Zarazaga 2009a.

[*saulcyi* **(Pandellé, 1867)**].Known in Denmark, southern Sweden (Lundberg and Gustafsson 1995), northern Poland (Burakowski et al. 1979).

*sculpturatus* **(Gravenhorst, 1806)**. Pileckis 1960, 1976a; Silfverberg 1992, 2004; Pileckis and Monsevičius 1995; Smetana 2004f; Ferenca 2006b; Alonso-Zarazaga 2009a.

*tetracarinatus* **(Block, 1799)**. Roubal 1910; Pileckis 1960, 1976a; Monsevičius 1986b, 1987, 1997; Dvilevičius et al. 1988; Silfverberg 1992, 2004; Gaidienė 1993; Pileckis and Monsevičius 1995; Ferenca 2006b.

[*tetratoma* **(Czwalina, 1870)**].Known in Sweden (Silfverberg 1992, 2004), Poland (Burakowski et al. 1979), western Belarus (Alexandrovich et al. 1996).

*Platystethus* **Mannerheim, 1830**.

*alutaceus* **Thomson, 1861**. Monsevičius and Jakaitis 1984; Silfverberg 1992, 2004; Pileckis and Monsevičius 1995; Smetana 2004f; Alonso-Zarazaga 2009a.

*arenarius* **(Geoffroy, 1785)**. Roubal 1910; Mazurowa and Mazur 1939; Pileckis 1960, 1976a; Monsevičius 1987, 1997; Silfverberg 1992, 2004; Gaidienė 1993; Pileckis and Monsevičius 1995; Smetana 2004f; Ferenca 2006b; Alonso-Zarazaga 2009a.

*cornutus* **(Gravenhorst, 1802)**. Pileckis 1976a; Gaidienė 1993; Silfverberg 1992, 2004; Pileckis and Monsevičius 1995; Smetana 2004f; Alonso-Zarazaga 2009a.

*nitens* **(C.R. Sahlberg, 1832)**. Karalius and Monsevičius 1992; Silfverberg 1996, 2004.

*nodifrons* **Mannerheim, 1830**. Pileckis 1960, 1976a; Silfverberg 1992, 2004; Gaidienė 1993; Pileckis and Monsevičius 1995; Smetana 2004f; Alonso-Zarazaga 2009a.

**Blediini Ádám, 2001**.

*Bledius* **Leach, 1819**.

[*annae* **Sharp, 1911**].Known in northwestern Belarus (Alexandrovich et al. 1996).

[*atricapillus* **(Germar, 1825)** = *praetermissus* Williams, 1929].Known in Latvia (Telnov 2004), Denmark, southern Sweden (Lundberg and Gustafsson 1995; Silfverberg 1992, 2004), Belarus (Alexandrovich et al. 1996), Poland (Burakowski et al. 1979).

[*baudii* **Fauvel, 1872**].Known in Denmark, southern Sweden, (Lundberg and Gustafsson 1995; Silfverberg 1992, 2004), Poland (Burakowski et al. 1979).

[*bicornis jutllandensis* **Herman, 1986** = *dama* Motschulsky, 1857].Known in Latvia (Telnov 2004), Denmark (Lundberg and Gustafsson 1995; Silfverberg 1992, 2004).

[*cribricollis* **Heer, 1839**].Known in Latvia (Telnov 2004), Denmark (Lundberg and Gustafsson 1995; Silfverberg 1992, 2004), northern Poland (Burakowski et al. 1979).

*defensus* **Fauvel, 1872**. Monsevičius 1985; Silfverberg 1992, 2004; Pileckis and Monsevičius 1995; Smetana 2004f; Alonso-Zarazaga 2009a.

[*denticollis* **Fauvel, 1872**].Known in Latvia (Telnov 2004), Sweden, Estonia (Lundberg and Gustafsson 1995; Silfverberg 1992, 2004), northwestern Belarus (Alexandrovich et al. 1996), Poland (Burakowski et al. 1979).

[*diota* **Schiödte, 1866**].Known in Sweden, Denmark, Estonia (Lundberg and Gustafsson 1995; Silfverberg 1992, 2004).

*disimilis* **Erichson, 1840**. Monsevičius 1983; Silfverberg 1992, 2004; Pileckis and Monsevičius 1995; Tamutis and Zolubas 2001; Smetana 2004f; Alonso-Zarazaga 2009a.

*erraticus* **Erichson, 1839** = *bosnicus* auct. nec Bernhauer, 1902 = *fontinalis* auct. nec Bernhauer, 1929.Pileckis 1976a; Silfverberg 1992, 2004; Pileckis and Monsevičius 1995; Smetana 2004f; Alonso-Zarazaga 2009a.

*femoralis* **(Gyllenhal, 1827)**. Pileckis 1976a; Silfverberg 1992, 2004; Pileckis and Monsevičius 1995; Smetana 2004f; Alonso-Zarazaga 2009a.

*fergussoni* **Joy, 1912 =** *arenarius* (Paykull, 1800) nec (Geoffroy, 1785).Pileckis 1976a; Silfverberg 1992, 2004; Pileckis and Monsevičius 1995; Monsevičius 1987, Smetana 2004f; Alonso-Zarazaga 2009a.

*filipes* **Sharp, 1911**.Monsevičius and Pankevičius 2001.

[*frater* **Kraatz, 1857**]. Known in Latvia (Telnov 2004), Poland (Alonso-Zarazaga 2009a).

[*furcatus* **(Olivier, 1811)**].Known in Sweden, Denmark (Lundberg and Gustafsson 1995; Silfverberg 1992, 2004),

[*fuscipes* **Rye, 1865**].Known in Denmark, throughout Sweden (Lundberg and Gustafsson, 1995), Estonia (Silfverberg 2004), northwestern Belarus (Alexandrovich et al. 1996).

*gallicus* **(Gravenhorst, 1806)** = *fracticornis* (Paykull, 1790) nec (O.F. Müller, 1776).Monsevičius 1983, 1987, 1997; Silfverberg 1992, 2004; Gaidienė 1993; Pileckis and Monsevičius 1995; Tamutis 1999, 2002b; Tamutis et al. 2004; Smetana 2004f; Alonso-Zarazaga 2009a.

[*limicola* **Tottenham, 1940** = *germanicus* Wagner, 1935, nec (Gravenhorst, 1806)].Known in Sweden, Denmark (Lundberg and Gustafsson 1995), Estonia (Silfverberg 2004), Poland (Burakowski et al. 1979), northwestern Belarus (Alexandrovich et al. 1996).

[*littoralis* **Heer, 1839**].Known in Latvia (Telnov 2004), Sweden, Denmark, Estonia (Lundberg and Gustafsson 1995; Silfverberg 1992, 2004), northwestern Belarus (Alexandrovich et al. 1996), Poland (Burakowski et al. 1979).

*longulus* **Erichson, 1839**. Roubal 1910; Pileckis 1960, 1976a; Silfverberg 1992, 2004; Gaidienė 1993; Pileckis and Monsevičius 1995; Smetana 2004f; Alonso-Zarazaga 2009a.

[*nanus* **Erichson, 1840**].Known in Latvia (Telnov 2004), Denmark, southern Sweden (Lundberg and Gustafsson 1995; Silfverberg 1992, 2004), southern Belarus (Alexandrovich et al. 1996), northern Poland (Burakowski et al. 1979).

*occidentalis* **Bondroit, 1907** = *crassicollis* auct. nec Lacordaire, 1835.Pileckis 1960, 1976a; Silfverberg 1992, 2004; Gaidienė 1993; Pileckis and Monsevičius 1995; Smetana 2004f.

*opacus* **(Block, 1799)** = *subsinuatus* Mulsant & Rey, 1878.Pileckis 1960, 1976a; Silfverberg 1992, 2004; Gaidienė 1993; Pileckis and Monsevičius 1995; Smetana 2004f; Alonso-Zarazaga 2009a; Ivinskis et al. 2009.

*pallipes* **(Gravenhorst, 1806)** = *larseni* Hansen, 1940.Monsevičius 1985; Silfverberg 1992, 2004; Gaidienė 1993; Pileckis and Monsevičius 1995; Smetana 2004f; Alonso-Zarazaga 2009a.

[*pygmaeus* **Erichson, 1839** = *agricultor* Heer, 1841].Known in Denmark (Lundberg and Gustafsson 1995; Silfverberg 1992, 2004), Poland (Burakowski et al. 1979).

*procerulus* **Erichson, 1840**. Pileckis 1976a; Silfverberg 1992, 2004; Gaidienė 1993; Pileckis and Monsevičius 1995; Smetana 2004f; Alonso-Zarazaga 2009a.

[*pusillus* **Erichson, 1839**].Known in Denmark, (Silfverberg 1992, 2004), northern Poland (Burakowski et al. 1979).

*rossicus* **Bernhauer & Schubert, 1911**. Gaidienė 1993; Silfverberg 2004.

[*spectabilis* **Kraatz, 1857**].Known in Denmark (Lundberg and Gustafsson 1995; Silfverberg 1992, 2004), Sweden (Alonso-Zarazaga 2009a).

[*subniger* **Schneider, 1898**].Known in Denmark (Lundberg and Gustafsson 1995; Silfverberg 1992, 2004). 

*subterraneus* **Erichson, 1839**. Monsevičius 1985, 1987, 1997; Silfverberg 1992, 2004; Gaidienė 1993; Pileckis and Monsevičius 1995; Smetana 2004f; Alonso-Zarazaga 2009a.

*talpa* **(Gyllenhal, 1810)**. Mazurowa and Mazur 1939; Pileckis 1960, 1976a; Monsevičius 1987, 1997; Silfverberg 1992, 2004; Gaidienė 1993; Pileckis and Monsevičius 1995; Smetana 2004f; Alonso-Zarazaga 2009a.

*terebrans* **(Schiödte, 1866)**.Monsevičius and Pankevičius 2001.

[*tibialis* **Heer, 1839**].Known in Latvia (Telnov 2004), Sweden, Denmark (Lundberg and Gustafsson 1995; Silfverberg 1992, 2004), Poland (Burakowski et al. 1979).

*tricornis* **(Herbst, 1784)**. Pileckis 1960, 1976a; Silfverberg 1992, 2004; Pileckis and Monsevičius 1995; Smetana 2004f; Ferenca 2006b; Alonso-Zarazaga 2009a.

*vilis* **Mäklin, 1876**. Monsevičius 1985, 1988a, 1997; Silfverberg 1992, 2004; Gaidienė 1993; Pileckis and Monsevičius 1995.

**Oxyporinae Fleming, 1821**.

*Oxyporus* **Fabricius, 1775**.

*mannerheimii* **Gyllenhal, 1827**. Łomnicki 1913; Pileckis 1968b, 1976a; Silfverberg 1992, 2004; Pileckis and Monsevičius 1995; Ferenca et al. 2002; Smetana 2004f; Alonso-Zarazaga 2009a.

*maxillosus* **Fabricius, 1793**. Wankowicz 1867b; Pileckis 1962, 1963b, 1976a; Silfverberg 1992, 2004; Gaidienė 1993; Pileckis and Monsevičius 1995; Smetana 2004f; Alonso-Zarazaga 2009a.

*rufus* **(Linnaeus, 1758)**. Eichwald 1830; Pileckis 1962, 1963b, 1976a; Gaidienė 1993; Silfverberg 1992, 2004; Pileckis and Monsevičius 1995; Smetana 2004f; Alonso-Zarazaga 2009a; Šablevičius 2011.

**Scydmaeninae Leach, 1815**.

**Eutheiini Casey, 1897**.

*Euthiconus* **Reitter, 1882**.

[*conicicollis* **(Fairmaire & Laboulbène, 1855)**]. Known in southern Sweden (Lundberg and Gustafsson 1995), Poland (Burakowski et al. 1978), Denmark (Sánchez-Terrón 2009).

*Eutheia* **Stephens, 1830**

[*linearis* **Mulsant, 1861**]. Known in Latvia (Telnov 2004), southern Sweden (Lundberg and Gustafsson 1995), Poland (Burakowski et al. 1978), Denmark (Sánchez-Terrón 2009).

*plicata* **(Gyllenhal, 1813)**. Pileckis 1976a; Silfverberg 1992, 2004; Pileckis and Monsevičius 1995; Vít 2004; Sánchez-Terrón 2009.

[*schaumii* **(Kiesenwetter, 1858)**]. Known in southern Sweden (Lundberg and Gustafsson 1995), Estonia (Süda 2009), Poland, Denmark (Sánchez-Terrón 2009).

*scydmaenoides* **Stephens, 1830**. Pileckis 1976a; Silfverberg 1992, 2004; Pileckis and Monsevičius 1995; Vít 2004; Sánchez-Terrón 2009.

**Cephenniini Reitter, 1882**.

*Cephennium* **O.F. Müller & Kunze, 1822**.

[*gallicum* **Ganglbauer, 1899**]. Known in southern Sweden (Lundberg and Gustafsson 1995), Denmark (Sánchez-Terrón 2009).

*majus* **Reitter, 1882**. Pileckis and Monsevičius 1982, 1995; Silfverberg 1992, 2004; Monsevičius 1997; Vít & Besuchet 2004; Sánchez-Terrón 2009.

*thoracicum* **O.F. Müller & Kunze, 1822**.# 50. Osterloff 1889; Pileckis 1976a; Silfverberg 1992, Pileckis and Monsevičius 1995; Sánchez-Terrón 2009.

**Cyrtoscydmini Schaufuss, 1889** = Stenichnini Ganglbauer, 1899.

*Nevraphes* **Thomson, 1859 =** *Neuraphes* Thomson, 1862.

*angulatus* **(O.F. Müller & Kunze, 1822)**. Pileckis 1976a; Silfverberg 1992, 2004; Pileckis and Monsevičius 1995; Vít 2004; Sánchez-Terrón 2009.

[*carinatus* **(Mulsant, 1861)**]. Known in Poland (Burakowski et al. 1978), Estonia, (Sánchez-Terrón 2009).

[*coronatus* **J.R. Sahlberg, 1881**]. Known in southern Sweden, Estonia (Lundberg and Gustafsson 1995), Poland (Burakowski et al. 1978), Denmark, (Sánchez-Terrón 2009).

*elongatulus* **(O.F. Müller & Kunze, 1822)**. Pileckis 1976a; Silfverberg 1992, 2004; Pileckis and Monsevičius 1995; Monsevičius 1997; Vít 2004; Sánchez-Terrón 2009.

[*plicicollis* **Reitter, 1879**]. Known in southern Sweden, Denmark (Lundberg and Gustafsson 1995; Sánchez-Terrón 2009), Belarus (Alexandrovitch et al. 1996).

[*praeteritus* **Rye, 1872**]. Known in Estonia (Lundberg and Gustafsson 1995; Sánchez-Terrón 2009).

*rubicundus* **(Schaum, 1841)**.# 51. Osterloff 1889; Pileckis 1976a; Pileckis and Monsevičius 1995.

*ruthenus* **Machulka, 1925**. Vít 2004.

*talparum* **Lokay, 1920**. # 51. Silfverberg 1992, 2004; Pileckis and Monsevičius 1995; Sánchez-Terrón 2009.

*Scydmoraphes* **Reitter, 1891**.

*helvolus* **(Schaum, 1844) =** *nigrescens* Reitter 1881. Jakaitis 1973; Pileckis 1976a; Silfverberg 1992, 2004; Pileckis and Monsevičius 1995; Vít 2004; Sánchez-Terrón 2009.

*minutus* **(Chaudoir, 1845)**. Pileckis 1976a; Silfverberg 1992, 2004; Pileckis and Monsevičius 1995; Vít 2004; Sánchez-Terrón 2009.

[*sparshalli* **(Denny, 1825)**]. Known in Latvia (Telnov 2004), southern Sweden (Lundberg and Gustafsson 1995), Denmark (Sánchez-Terrón 2009).

*Stenichnus* **Thomson, 1859**.

*bicolor* **(Denny, 1825) =** *exilis* (Erichson, 1837). Pileckis 1976a; Silfverberg 1992, 2004; Pileckis and Monsevičius 1995; Vít 2004.

*collaris* **(O.F. Müller & Kunze, 1822)**.Roubal 1910; Pileckis 1960, 1976a; Silfverberg 1992, 2004; Pileckis and Monsevičius 1995; Monsevičius 1997; Vít 2004; Sánchez-Terrón 2009.

*godarti* **(Latreille, 1806)**. Pileckis 1976a; Silfverberg 1992, 2004; Pileckis and Monsevičius 1995; Vít 2004.

[*poweri* **(Fowler, 1884) =** *harwoodianus* Williams, 1927]. Known in southern Sweden, Denmark (Lundberg and Gustafsson 1995; Sánchez-Terrón 2009).

[*pusillus* **(O.F. Müller & Kunze, 1822)**]. Known in southern Sweden (Lundberg and Gustafsson 1995), northeastern Poland (Burakowski et al. 1978; Sánchez-Terrón 2009).

*scutellaris* **(O.F. Müller & Kunze, 1822)**. Pileckis 1976a; Silfverberg 1992, 2004; Pileckis and Monsevičius 1995; Vít 2004; Sánchez-Terrón 2009.

*Microscydmus* **Saulcy & Croissandeau, 1893**.

[*minimus* **(Chaudoir, 1845)**]. Known in Latvia (Telnov 2004), Estonia, Denmark, southern Sweden (Lundberg and Gustafsson 1995) Poland (Sánchez-Terrón 2009).

*nanus* **(Schaum, 1844)**. Pileckis 1976a (*Euconnus*); Silfverberg 1992, 2004; Pileckis and Monsevičius 1995; Vít 2004; Sánchez-Terrón 2009.

*Euconnus* **Thomson, 1859**.

*claviger* **(O.F. Müller & Kunze, 1822)**. Pileckis 1976a; Silfverberg 1992, 2004; Pileckis and Monsevičius 1995; Sánchez-Terrón 2009.

*denticornis* **(O.F. Müller & Kunze, 1822)**.Pileckis 1976a; Silfverberg 1992, 2004; Pileckis and Monsevičius 1995; Vít 2004; Sánchez-Terrón 2009.

*fimetarius* **(Chaudoir, 1845)**. Pileckis 1976a; Silfverberg 1992, 2004; Pileckis and Monsevičius 1995; Monsevičius 1997; Vít 2004; Sánchez-Terrón 2009.

*hirticollis* **(Illiger, 1798)**. Pileckis and Monsevičius 1995.

*maklinii* **(Mannerheim, 1844)**. Pileckis 1976a; Silfverberg 1992, 2004; Pileckis and Monsevičius 1995; Vít 2004.

*pubicollis* **(O.F. Müller & Kunze, 1822)**. Pileckis and Monsevičius 1982, 1995; Silfverberg 1992, 2004; Monsevičius 1997; Vít 2004; Sánchez-Terrón 2009.

*rutilipennis* **(O.F. Müller & Kunze, 1822)**. Pileckis 1976a; Silfverberg 1992, 2004; Pileckis and Monsevičius 1995; Vít 2004; Sánchez-Terrón 2009.

*wetterhallii* **(Gyllenhal, 1813)**. Pileckis 1976a; Silfverberg 1992, 2004; Pileckis and Monsevičius 1995; Vít 2004; Sánchez-Terrón 2009.

**Scydmaenini Leach, 1815**.

*Scydmaenus* **Latreille, 1802**.

*hellwigii* **(Herbst, 1792)**. Monsevičius and Pankevičius 2001.

[*perrisii* **(Reitter, 1882)**]. Known in southern Sweden (Lundberg and Gustafsson 1995), Poland (Burakowski et al. 1978; Sánchez-Terrón 2009).

*rufus* **O.F. Müller & Kunze, 1822**. Gaidienė 1993; Silfverberg 2004.

*tarsatus* **O.F. Müller & Kunze, 1822**. Pileckis 1976a (*S. tarsalis*); Pileckis and Monsevičius 1995; Silfverberg 1992, 2004; Vít 2004; Ferenca et al. 2006; Sánchez-Terrón 2009.

**Steninae MacLeay, 1825**.

*Stenus* **Latreille, 1797**.

[*ageus* **Casey, 1884** = *gerhardti* L. Benick, 1915].Known in Latvia (Telnov 2004), Sweden, Estonia (Lundberg and Gustafsson 1995; Silfverberg 1992, 2004), northwestern Belarus (Alexandrovich et al. 1996), Poland (Alonso-Zarazaga 2009a).

[*ampliventris* **J.R. Sahlberg, 1890**].Known in northern Belarus (Alexandrovich et al. 1996).

*argus* **Gravenhorst, 1806**. Pileckis 1960, 1976a; Silfverberg 1992, 2004; Pileckis and Monsevičius 1995; Monsevičius 1997; Smetana 2004f; Ferenca 2006b; Alonso-Zarazaga 2009a.

*assequens* **Rey, 1884** = *pusio* Casey, 1884 = *simillimus* L. Benick, 1949.Tamutis 2002b, c, 2003.

*ater* **Mannerheim, 1830**. Pileckis 1976a; Bercio and Folwaczny 1979; Silfverberg 1992, 2004; Pileckis and Monsevičius 1995; Smetana 2004f; Alonso-Zarazaga 2009a.

*aterrimus* **Erichson, 1839**. Pileckis 1976a; Silfverberg 1992, 2004; Pileckis and Monsevičius 1995; Smetana 2004f; Alonso-Zarazaga 2009a.

*atratulus* **Erichson, 1839**. Monsevičius 1985; Silfverberg 1992, 2004; Pileckis and Monsevičius 1995; Smetana 2004f; Alonso-Zarazaga 2009a.

*bifoveolatus* **Gyllenhal, 1827**. Pileckis 1976a; Monsevičius 1986b, 1997; Silfverberg 1992, 2004; Pileckis and Monsevičius 1995; Smetana 2004f; Alonso-Zarazaga 2009a.

*biguttatus* **(Linnaeus, 1758)**. Pileckis 1960, 1976a; Silfverberg 1992, 2004; Gaidienė 1993; Pileckis and Monsevičius 1995; Monsevičius 1997; Tamutis 1999, 2002b; Tamutis and Zolubas 2001; Tamutis et al. 2004; Smetana 2004f; Ferenca 2006b; Vaivilavičius 2008; Alonso-Zarazaga 2009a.

*bimaculatus* **Gyllenhal, 1810**. Pileckis 1960, 1976a; Monsevičius 1987, 1997; Silfverberg 1992, 2004; Gaidienė 1993; Pileckis and Monsevičius 1995; Smetana 2004f; Ferenca 2006b; Alonso-Zarazaga 2009a.

*binotatus* **Ljungh, 1804**. Mazurowa and Mazur 1939; Pileckis 1960, 1976a; Bercio and Folwaczny 1979; Silfverberg 1992, 2004; Gaidienė 1993; Pileckis and Monsevičius 1995; Monsevičius 1997; Smetana 2004f; Alonso-Zarazaga 2009a.

*bohemicus* **Machulka, 1947**. Monsevičius 1982, 1997; Silfverberg 1992, 2004; Gaidienė 1993; Pileckis and Monsevičius 1995; Smetana 2004f; Alonso-Zarazaga 2009a.

*boops* **Ljungh, 1810**. Mazurowa and Mazur 1939; Pileckis 1960, 1976a; Monsevičius 1987, 1997; Silfverberg 1992, 2004; Gaidienė 1993; Pileckis and Monsevičius 1995; Smetana 2004f; Alonso-Zarazaga 2009a.

*brunnipes* **Stephens, 1833**. Pileckis 1976a; Silfverberg 1992, 2004; Gaidienė 1993; Pileckis and Monsevičius 1995; Smetana 2004f; Alonso-Zarazaga 2009a.

*calcaratus* **W. Scriba, 1864**. Pileckis 1960, 1976a; Bercio and Folwaczny 1979; Silfverberg 1992, 2004; Pileckis and Monsevičius 1995; Smetana 2004f; Ferenca 2006b; Alonso-Zarazaga 2009a.

*canaliculatus* **Gyllenhal, 1827**. Pileckis 1960, 1976a; Bercio and Folwaczny 1979; Silfverberg 1992, 2004; Pileckis and Monsevičius 1995; Tamutis et al. 2004; Smetana 2004f; Ferenca 2006b; Alonso-Zarazaga 2009a.

*carbonarius* **Gyllenhal, 1827**. Pileckis 1976a; Silfverberg 1992, 2004; Pileckis and Monsevičius 1995; Monsevičius 1997; Smetana 2004f; Alonso-Zarazaga 2009a; Alekseev 2008a.

*cautus* **Erichson, 1839** = *vafellus* Erichson, 1839.Pileckis 1976a; Silfverberg 1992, 2004; Pileckis and Monsevičius 1995; Monsevičius 1997; Smetana 2004f; Alonso-Zarazaga 2009a.

*cicindeloides* **Schaller, 1783**. Pileckis 1976a; Silfverberg 1992, 2004; Gaidienė 1993; Pileckis and Monsevičius 1995; Monsevičius 1997; Smetana 2004f; Alonso-Zarazaga 2009a.

*circularis* **Gravenhorst, 1802**. Pileckis 1976a; Silfverberg 1992, 2004; Pileckis and Monsevičius 1995; Monsevičius 1997; Smetana 2004f; Alonso-Zarazaga 2009a.

*clavicornis* **(Scopoli, 1763)**. Pileckis 1960, 1976a; Monsevičius 1987, 1997; Dvilevičius et al. 1988; Silfverberg 1992, 2004; Gaidienė 1993; Pileckis and Monsevičius 1995; Tamutis 2002b; Tamutis et al. 2004; Smetana 2004f; Ferenca 2006b; Dapkus and Tamutis 2007; Alonso-Zarazaga 2009a.

*comma* **(LeConte, 1863)** = *bipunctatus* Erichson, 1839, nec Ljungh, 1804.Roubal 1910; Mazurowa and Mazur 1939; Pileckis 1960, 1976a; Lešinskas and Pileckis 1967; Monsevičius 1987, 1997; Silfverberg 1992, 2004; Gaidienė 1993; Pileckis and Monsevičius 1995; Gliaudys 2001; Smetana 2004f; Ferenca 2006b; Alonso-Zarazaga 2009a; Alekseev 2008a.

*crassus* **Stephens, 1833**. Pileckis 1976a; Bercio and Folwaczny 1979; Silfverberg 1992, 2004; Gaidienė 1993; Pileckis and Monsevičius 1995; Monsevičius 1997; Smetana 2004f; Alonso-Zarazaga 2009a.

[*eumerus* **Kiesenwetter, 1850**]. Known in northern Belarus (Alexandrovich et al. 1996), Poland (Burakowski et al. 1979).

*europaeus* **Puthz, 1966**. Monsevičius 1986b, 1997; Silfverberg 1992, 2004; Pileckis and Monsevičius 1995; Smetana 2004f; Alonso-Zarazaga 2009a.

[*excubitor* **Erichson, 1839** = *rossicus* Bernhauer, 1903]. # 52.Known in Latvia (Telnov et al. 2005), Denmark, Estonia (Silfverberg 1992, 2004), southern Sweden (Lundberg and Gustafsson 1995), northern Poland (Burakowski et al. 1979), northwestern Belarus (Alexandrovich et al. 1996).

*flavipalpis* **Thomson, 1860**. Bercio and Folwaczny 1979; Monsevičius 1982, 1986b, 1997; Silfverberg 1992, 2004; Pileckis and Monsevičius 1995; Smetana 2004f.

*flavipes* **Stephens, 1833**. Gaidienė 1993; Pileckis and Monsevičius 1995; Monsevičius 1997; Silfverberg 2004; Smetana 2004f; Alonso-Zarazaga 2009a; Ivinskis et al. 2009.

*formicetorum* **Mannerheim, 1843**. Pileckis 1976a; Silfverberg 1992, 2004; Pileckis and Monsevičius 1995; Monsevičius 1997; Smetana 2004f; Vorst et al. 2007; Alonso-Zarazaga 2009a; Ivinskis et al. 2009.

*fornicatus* **Stephens, 1833**. Gaidienė 1993; Silfverberg 2004.

[*fossulatus* **Erichson, 1840**].Known in Latvia (Telnov et al. 2006), Denmark, Estonia, throughout Sweden (Lundberg and Gustafsson 1995), northeastern Poland (Burakowski et al. 1979).

*fulvicornis* **Stephens, 1833**. Pileckis 1976a; Silfverberg 1992, 2004; Pileckis and Monsevičius 1995; Monsevičius 1997; Tamutis et al. 2004; Smetana 2004f.

*fuscicornis* **Erichson, 1840**. Monsevičius 1982, 1997; Silfverberg 1992, 2004; Pileckis and Monsevičius 1995.

*fuscipes* **Gravenhorst, 1802**. Pileckis 1976a; Monsevičius 1987, 1997; Silfverberg 1992, 2004; Pileckis and Monsevičius 1995; Smetana 2004f; Alonso-Zarazaga 2009a.

*gallicus* **Fauvel, 1873**. # 52.Monsevičius 1997.

*geniculatus* **Gravenhorst, 1806**. Bercio and Folwaczny 1979; Monsevičius 1983, 1987, 1997; Silfverberg 1992, 2004; Pileckis and Monsevičius 1995; Smetana 2004f; Alonso-Zarazaga 2009a.

*glabellus* **Thomson, 1870**. Monsevičius 1982, 1997; Silfverberg 1992, 2004; Pileckis and Monsevičius 1995; Smetana 2004f; Vorst et al. 2007; Alonso-Zarazaga 2009a; Ivinskis et al. 2009.

[*guttula* **O.F. Müller, 1821**].Know in Latvia (Telnov 2004), Denmark, southern Sweden (Lundberg and Gustafsson 1995), Poland (Burakowski et al. 1979).

*humilis* **Erichson, 1839**. Monsevičius 1982, 1987, 1997; Dvilevičius et al. 1988; Silfverberg 1992, 2004; Gaidienė 1993; Pileckis and Monsevičius 1995; Smetana 2004f; Alonso-Zarazaga 2009a.

*impressus* **Germar, 1824**.Monsevičius 1983, 1986b, 1997; Silfverberg 1992, 2004; Pileckis and Monsevičius 1995; Smetana 2004f; Alonso-Zarazaga 2009a; Ivinskis et al. 2009.

*incanus* **Erichson, 1839**..Pileckis 1960.

*incrassatus* **Erichson, 1839**. Pileckis 1976a; Silfverberg 1992, 2004; Pileckis and Monsevičius 1995; Monsevičius 1997; Smetana 2004f; Alonso-Zarazaga 2009a.

*intermedius* **Rey, 1884** = *problematicus* Kevan &Allen, 1962.Monsevičius 1983, 1997; Silfverberg 1992, 2004; Pileckis and Monsevičius 1995; Smetana 2004f.

*juno* **(Paykull, 1789)**. Pileckis 1976a; Monsevičius 1986b, 1987, 1997; Dvilevičius et al. 1988; Silfverberg 1992, 2004; Gaidienė 1993; Pileckis and Monsevičius 1995; Smetana 2004f; Alonso-Zarazaga 2009a.

*kiesenwetteri* **Rosenhauer, 1856**. Tamutis et al. 2008.

*kolbei* **Gerhardt, 1893**. Łomnicki 1913; Pileckis 1968b, 1976a; Silfverberg 1992, 2004; Pileckis and Monsevičius 1995; Monsevičius 1997; Alonso-Zarazaga 2009a.

*latifrons* **Erichson, 1839**. Pileckis 1976a; Silfverberg 1992, 2004; Gaidienė 1993; Pileckis and Monsevičius 1995; Monsevičius 1997; Smetana 2004f; Alonso-Zarazaga 2009a.

*longipes* **Heer, 1839**. Silfverberg 1992, 2004; Smetana 2004f; Alonso-Zarazaga 2009a; Ivinskis et al. 2009.

[*longitarsis* **Thomson, 1851**].Know in Latvia (Telnov 2004), Denmark, southern Sweden (Lundberg and Gustafsson 1995), Poland (Burakowski et al. 1979).

[*ludyi* **Fauvel, 1886**].Known in Latvia (Telnov et al. 2007), Denmark, Estonia, throughout Sweden (Lundberg and Gustafsson 1995), northern Poland (Burakowski et al. 1979), northwestern Belarus (Alexandrovich et al. 1996).

*lustrator* **Erichson, 1839**.Pileckis 1976a; Monsevičius 1987, 1997; Silfverberg 1992, 2004; Gaidienė 1993; Pileckis and Monsevičius 1995; Smetana 2004f; Alonso-Zarazaga 2009a.

*melanarius* **Stephens, 1833**. Pileckis 1976a; Bercio and Folwaczny 1979; Silfverberg 1992, 2004; Pileckis and Monsevičius 1995; Smetana 2004f; Alonso-Zarazaga 2009a.

*melanopus* **(Marsham, 1802)**.Monsevičius and Pankevičius 2001; Alonso-Zarazaga 2009a.

*morio* **Gravenhorst, 1806**. Monsevičius 1985, 1997; Silfverberg 1992, 2004; Gaidienė 1993; Pileckis and Monsevičius 1995; Smetana 2004f; Alonso-Zarazaga 2009a.

*nanus* **Stephens, 1833**. Pileckis 1976a; Monsevičius 1986b, 1997; Silfverberg 1992, 2004; Pileckis and Monsevičius 1995; Smetana 2004f; Alonso-Zarazaga 2009a; Ivinskis et al. 2009.

*nigritulus* **Gyllenhal, 1827**. Pileckis 1976a; Silfverberg 1992, 2004; Pileckis and Monsevičius 1995; Monsevičius 1997; Smetana 2004f; Alonso-Zarazaga 2009a.

*nitens* **Stephens, 1833**.Monsevičius and Pankevičius 2001.

[*nitidiusculus* **Stephens, 1833**]. Known in Denmark, southern Sweden (Lundberg and Gustafsson 1995), northern Poland (Burakowski et al. 1979).

[*niveus* **Fauvel, 1865**]. Known in Denmark, Estonia, northern Sweden (Lundberg and Gustafsson 1995), Poland (Burakowski et al. 1979).

*ochropus* **Kiesenwetter, 1858**. Monsevičius 1982, 1997; Silfverberg 1992, 2004; Pileckis and Monsevičius 1995.

*opticus* **Gravenhorst, 1806**. Monsevičius 1982, 1997; Dvilevičius et al. 1988; Silfverberg 1992, 2004; Gaidienė 1993; Pileckis and Monsevičius 1995; Smetana 2004f.

[*oscillator* **Rye, 1870**]. Known in Denmark, southern Sweden (Lundberg and Gustafsson 1995), northern Poland (Burakowski et al. 1979).

*pallipes* **Gravenhorst, 1802**. Pileckis 1960, 1976a; Silfverberg 1992, 2004; Gaidienė 1993; Pileckis and Monsevičius 1995; Monsevičius 1997; Smetana 2004f; Ferenca 2006b; Alonso-Zarazaga 2009a.

*pallitarsis* **Stephens, 1833**. Monsevičius 1988; Silfverberg 1992, 2004; Pileckis and Monsevičius 1995; Smetana 2004f; Alonso-Zarazaga 2009a.

*palposus* **Zetterstedt, 1828**. Mazurowa and Mazur 1939; Pileckis 1960, 1976a; Bercio and Folwaczny 1979; Monsevičius 1987, 1997; Silfverberg 1992, 2004; Pileckis and Monsevičius 1995; Smetana 2004f; Ferenca 2006b; Alekseev 2008a; Alonso-Zarazaga 2009a.

*palustris* **Erichson, 1839**. Monsevičius 1982, 1986b, 1997; Silfverberg 1992, 2004; Pileckis and Monsevičius 1995; Smetana 2004f; Alonso-Zarazaga 2009a.

[*picipennis* **Erichson, 1840**]. Known in Denmark, Estonia (Lundberg and Gustafsson 1995; Silfverberg 1992, 2004), northern Poland (Burakowski et al. 1979).

*picipes* **Stephens, 1833** = *brevipennis* Thomson, 1851 = *foveicollis* Kraatz, 1857. Silfverberg 2004.

*proditor* **Erichson, 1839**. Monsevičius 1983, 1997; Silfverberg 1992, 2004; Pileckis and Monsevičius 1995; Smetana 2004f; Alonso-Zarazaga 2009a; Ivinskis et al. 2009

*providus* **Erichson, 1839** = *rogeri* Kraatz, 1857.Pileckis 1960, 1976a; Bercio and Folwaczny 1979; Silfverberg 1992, 2004; Pileckis and Monsevičius 1995; Smetana 2004f; Ferenca 2006b; Alonso-Zarazaga 2009a.

*pubescens* **Stephens, 1833**. Pileckis 1976a; Bercio and Folwaczny 1979; Silfverberg 1992, 2004; Pileckis and Monsevičius 1995; Smetana 2004f; Alonso-Zarazaga 2009a.

[*pumilio* **Erichson, 1839**]. Known in Latvia (Telnov 2004), Denmark, throughout Sweden (Lundberg and Gustafsson 1995), northern Poland (Burakowski et al. 1979), northwestern Belarus (Alexandrovich et al. 1996).

*pusillus* **Stephens, 1833** = *exiguus* Erichson, 1840]. Pileckis 1976a; Monsevičius 1986b, 1997; Dvilevičius et al. 1988; Silfverberg 1992, 2004; Gaidienė 1993; Pileckis and Monsevičius 1995; Smetana 2004f; Alonso-Zarazaga 2009a.

*ruralis* **Erichson, 1840**.Monsevičius and Pankevičius 2001; Smetana 2004f; Alonso-Zarazaga 2009a.

[*scabriculus* **J.R. Sahlberg, 1876**]. Known in Latvia (Telnov 2004), Sweden (Lundberg and Gustafsson 1995), Estonia (Alonso-Zarazaga 2009a).

*scrutator* **Erichson, 1840**. Horion 1963; Bercio and Folwaczny 1979; Silfverberg 1992, 2004; Pileckis and Monsevičius 1995; Monsevičius 1997; Smetana 2004f; Alonso-Zarazaga 2009a.

*similis* **(Herbst, 1784)**. Monsevičius 1982; Dvilevičius et al. 1988; Silfverberg 1992, 2004; Gaidienė 1993; Pileckis and Monsevičius 1995; Tamutis 1999; Alonso-Zarazaga 2009a.

*sylvester* **Erichson, 1839**. Pileckis 1976a; Dvilevičius et al. 1988; Silfverberg 1992, 2004; Gaidienė 1993; Pileckis and Monsevičius 1995; Monsevičius 1997; Smetana 2004f; Ivinskis et al. 2009.

*solutus* **Erichson, 1840**. Pileckis and Monsevičius 1995; Silfverberg 2004.

*stigmula* **Erichson, 1840**.Ferenca and Tamutis 2009.

[*subdepressus* **Mulsant & Rey, 1861**].Known in Denmark (Lundberg and Gustafsson 1995; Silfverberg 1992, 2004), Poland (Burakowski et al. 1979).

*tarsalis* **Ljungh, 1804**. Roubal 1910; Pileckis 1960, 1976a; Bercio and Folwaczny 1979; Silfverberg 1992, 2004; Pileckis and Monsevičius 1995; Monsevičius 1997; Smetana 2004f; Alonso-Zarazaga 2009a; Ivinskis et al. 2009.

[*umbratilis* **Casey, 1884** = *pseudopubescens* Strand, 1940]. Known in Latvia (Telnov 2004), Denmark, throughout Sweden (Lundberg and Gustafsson 1995), Estonia (Silfverberg 2004), Poland (Alonso-Zarazaga 2009a), northern Belarus (Alexandrovich et al. 1996).

*Dianous* **Samouelle, 1819**.

*coerulescens* **(Gyllenhal, 1810)**. Ivinskis et al. 2009.

**Euaesthetinae Thomson, 1859**.

**Euaesthetini Thomson, 1859**.

*Edaphus* **Motschulsky, 1857**.

[*beszedesi* **Reitter, 1913** = *bluehweissi* Scheerpeltz, 1936].Known in Latvia (Telnov et al. 2005), Estonia (Lundberg and Gustafsson 1995).

*Euaesthetus* **Gravenhorst, 1806**.

*bipunctatus* **(Ljungh, 1804)**. Pileckis 1976a; Silfverberg 1992, 2004; Pileckis and Monsevičius 1995; Monsevičius 1997; Smetana 2004f; Alonso-Zarazaga 2009a.

*laeviusculus* **Mannerheim, 1844**. Monsevičius 1983, 1986b, 1997; Silfverberg 1992, 2004; Pileckis and Monsevičius 1995; Smetana 2004f; Vorst et al. 2007; Alonso-Zarazaga 2009a.

*ruficapillius* **Lacordaire, 1835**. Monsevičius 1983, 1986b, 1987, 1997; Silfverberg 1992, 2004; Pileckis and Monsevičius 1995; Smetana 2004f; Alonso-Zarazaga 2009a.

**Pseudopsinae Ganglbauer, 1895**.

*Pseudopsis* **Newman, 1834**.

[*sulcata* **Newman, 1834**]. Known in northern Poland (Burakowski et al. 1979).

**Paederinae Fleming, 1821**.

**Paederini Fleming, 1821**.

*Paederidus* **Mulsant & Rey, 1877**.

[*ruficollis* **(Fabricius, 1781)**]. Known in Denmark, southern Sweden (Lundberg and Gustafsson 1995), northeastern Poland (Burakowski et al. 1979).

*Paederus* **Fabricius, 1775**.

*brevipennis* **Lacordaire, 1835**. Monsevičius 1999.

[*caligatus* **Erichson, 1840**]. Known in northern Poland (Burakowski et al. 1979).

*fuscipes* **Curtis, 1826**. Heyden 1903; Mazurowa and Mazur 1939; Pileckis 1960, 1976a; Zajančkauskas and Pileckis 1968; Silfverberg 1992, 2004; Gaidienė 1993; Pileckis and Monsevičius 1995; Monsevičius 1997; Smetana 2004g; Ferenca 2006b; Alonso-Zarazaga 2009a.

[*limnophilus* **Erichson, 1840**]. Recently re-found in Latvia (Cibulskis et al. 2009), known in Poland (Burakowski et al. 1979).

*littoralis* **Gravenhorst, 1802**. Pileckis 1976a; Silfverberg 1992, 2004; Gaidienė 1993; Pileckis and Monsevičius 1995; Smetana 2004g; Alonso-Zarazaga 2009a.

*riparius* **(Linnaeus, 1758)**. Zajančkauskas and Pileckis 1968; Pileckis 1968b, 1976a; Bercio and Folwaczny 1979; Monsevičius 1987, 1997; Silfverberg 1992, 2004; Gaidienė 1993; Pileckis and Monsevičius 1995; Tamutis et al. 2004, 2007; Smetana 2004g; Alekseev 2008a; Alonso-Zarazaga 2009a; Šablevičius 2011.

*Astenus* **Stephens, 1833**.

[*bimaculatus* **(Erichson, 1839)**].Known in Estonia (Roosileht 2004).

*gracilis* **(Paykull, 1789)** = *angustatus* (Paykull, 1789) nec (Schrank, 1781).Bercio and Folwaczny 1979; Gaidienė 1993; Pileckis and Monsevičius 1995; Silfverberg 2004; Smetana 2004g; Ferenca et al. 2006, 2007; Alonso-Zarazaga 2009a.

[*immaculatus* **Stephens, 1833**]. Known in northern Poland (Burakowski et al. 1979), Denmark, southern Sweden (Lundberg and Gustafsson 1995).

*lyonessius* **(Joy, 1908)** = *longelytratus* Palm, 1936.Pileckis 1976a; Silfverberg 1992, 2004; Pileckis and Monsevičius 1995; Smetana 2004g; Alonso-Zarazaga 2009a.

*procerus* **(Gravenhorst, 1806)** = *filiformis* (Latreille, 1806) nec (Fabricius, 1793).Bercio and Folwaczny 1979; Silfverberg 1992, 2004; Smetana 2004g; Alonso-Zarazaga 2009a.

*pulchellus* **(Heer, 1839)**. Pileckis and Monsevičius 1995; Silfverberg 2004.

*Rugilus* **Leach, 1819** = *Stilicus* Berthold, 1827.

*angustatus* **(Geoffroy, 1785)** = *fragilis* (Gravenhorst, 1806) = *scutellatus* (Motschulsky, 1858).Tamutis and Zolubas 2001; Ferenca et al. 2006, 2007; Ivinskis et al. 2009.

*erichsonii* **(Fauvel, 1867)**. Pileckis 1976a; Silfverberg 1992, 2004; Gaidienė 1993; Pileckis and Monsevičius 1995; Monsevičius 1997; Smetana 2004g; Alonso-Zarazaga 2009a.

*geniculatus* **(Erichson, 1839)**. Monsevičius 1982; Silfverberg 1992, 2004; Pileckis and Monsevičius 1995.

*orbiculatus* **(Paykull, 1789)**. Monsevičius 1985, 1997; Silfverberg 1992, 2004; Pileckis and Monsevičius 1995; Smetana 2004g; Alonso-Zarazaga 2009a.

*rufipes* **Germar, 1836**. Pileckis 1960, 1976a; Monsevičius 1986b, 1987, 1997; Dvilevičius et al. 1988; Silfverberg 1992, 2004; Gaidienė 1993; Pileckis and Monsevičius 1995; Smetana 2004g; Ferenca 2006b; Alonso-Zarazaga 2009a.

*similis* **(Erichson, 1839)**. Silfverberg 1992, 2004; Gaidienė 1993; Pileckis and Monsevičius 1995; Smetana 2004g; Alonso-Zarazaga 2009a.

*Medon* **Stephens, 1833**.

[*apicalis* **(Kraatz, 1857)**]. Known in Denmark, throughout Sweden (Lundberg and Gustafsson 1995).

[*brunneus* **(Erichson, 1839)**]. Known in Denmark, southern Sweden (Lundberg and Gustafsson 1995), northern Poland (Burakowski et al. 1979).

*castaneus* **(Gravenhorst, 1802)**. Pileckis 1976a; Silfverberg 1992, 2004; Pileckis and Monsevičius 1995; Monsevičius 1997, 1999; Smetana 2004g; Alonso-Zarazaga 2009a.

[*dilutus* **(Erichson, 1839)**]. Known in southern Sweden (Lundberg and Gustafsson 1995), Poland (Burakowski et al. 1979).

*fusculus* **(Mannerheim, 1830)**. Pileckis 1976a; Silfverberg 1992, 2004; Pileckis and Monsevičius 1995; Smetana 2004g; Alonso-Zarazaga 2009a.

*ripicola* **(Kraatz, 1854)**. Pileckis 1976a; Silfverberg 1992, 2004; Pileckis and Monsevičius 1995.

[*rufiventris* **(Nordmann, 1837)**]. Known in southern Sweden (Lundberg and Gustafsson 1995), Poland (Burakowski et al. 1979).

*Sunius* **Stephens, 1829** = *Hypomedon* Mulsant & Rey, 1877.

[*bicolor* **(Olivier, 1795)**]. Known in Denmark, southern Sweden (Lundberg and Gustafsson 1995), northern Poland (Burakowski et al. 1979).

*melanocephalus* **Fabricius, 1793**. Tamutis and Černiauskaitė-Kedienė 2005; Tamutis and Ferenca 2006; Ferenca et al. 2006, 2007.

*Pseudomedon* **Mulsant & Rey, 1877**.

[*obscurellus* **(Erichson, 1840)**]. Known in Latvia (Telnov 2004), northern Poland (Burakowski et al. 1979), Denmark, throughout Sweden (Lundberg and Gustafsson 1995).

[*obsoletus* **(Nordmann, 1837)**]. Known in Latvia (Telnov 2004), northern Poland (Burakowski et al. 1979), Denmark, southern Sweden (Lundberg and Gustafsson 1995).

*Lithocharis* **Dejean, 1833**.

*nigriceps* **Kraatz, 1859**. Monsevičius 1997.

*ochracea* **(Gravenhorst, 1802)** = *tricolor* auct. nec (Fabricius, 1787).Tamutis 2003; Ferenca et al. 2006, 2007.

*Hypomedon* **Mulsant & Rey, 1878**.

[*debilicornis* **(Wollaston, 1857)**]. Known in Sweden (Lundberg and Gustafsson 1995).

*Scopaeus* **Erichson, 1840**.

*laevigatus* **(Gyllenhal, 1827)**. Pileckis 1960, 1976a; Silfverberg 1992, 2004; Pileckis and Monsevičius 1995; Monsevičius 1997; Smetana 2004g; Žiogas and Zolubas 2005; Alonso-Zarazaga 2009a.

*minutus* **Erichson, 1840**. Ivinskis et al. 2009.

*pusillus* **Kiesenwetter, 1843**. Ferenca 2003.

*ryei* **Wollaston, 1872** = *minimus* auct. nec (Erichson, 1839).Tamutis 2003; Vaivilavičius 2008.

[*sulcicollis* **(Stephens, 1833)** = *cognatus* Mulsant & Rey, 1855].Known in Denmark, Estonia, southern Sweden (Lundberg and Gustafsson 1995), northern Poland (Burakowski et al. 1979).

*Lobrathium* **Mulsant & Rey, 1878**.

*multipunctum* **(Gravenhorst, 1802)**. Pileckis and Monsevičius 1995; Silfverberg 2004.

*Tetartopeus* **Czwalina, 1888**.

*quadratus* **(Paykull, 1789)**. Pileckis 1976a; Bercio and Folwaczny 1979; Silfverberg 1992, 2004; Gaidienė 1993; Pileckis and Monsevičius 1995; Smetana 2004g; Alonso-Zarazaga 2009a.

[*rufonitidus* **(Reitter, 1909)** = *fennicum* (Renkonen, 1938)]. Known in Latvia (Vorst et al. 2007), Denmark, Estonia, throughout Sweden (Lundberg and Gustafsson 1995), Poland (Burakowski et al. 1979), Belarus (Alexandrovich et al. 1996).

*scutellaris* **(Nordmann, 1837)**. Pileckis 1976a; Silfverberg 1992, 2004; Pileckis and Monsevičius 1995; Tamutis et al. 2004.

[*sphagnetorum* **(Muona, 1977)** = *gracile* Hampe, 1866].Known in Sweden (Lundberg and Gustafsson 1995), northeastern Poland (Burakowski et al. 1979), Denmark (Alonso-Zarazaga 2009a).

*terminatus* **(Gravenhorst, 1802)**. Pileckis 1976a; Monsevičius 1986b, 1997; Silfverberg 1992, 2004; Gaidienė 1993; Pileckis and Monsevičius 1995; Smetana 2004g; Alonso-Zarazaga 2009a.

[*zetterstedti* **(Rye, 1872)** = *punctatum* (Zetterstedt, 1828), nec (Goeze, 1785)].Known in Latvia (Telnov 2004), Estonia (Silfverberg 2004), throughout Sweden (Lundberg and Gustafsson 1995), Denmark, Poland (Alonso-Zarazaga 2009a).

*Lathrobium* **Gravenhorst, 1802**.

*brunnipes* **(Fabricius, 1793)**. Pileckis 1960, 1976a; Monsevičius 1986b, 1987, 1997; Dvilevičius et al. 1988; Silfverberg 1992, 2004; Gaidienė 1993; Pileckis and Monsevičius 1995; Smetana 2004g; Ferenca 2006b; Alonso-Zarazaga 2009a.

[*castaneipenne* **Kolenati, 1846**]. Known in Latvia (Silfverberg 2004; Cibulskis et al. 2009), Estonia, Sweden (Lundberg and Gustafsson 1995), northern Poland Burakowski et al. 1979), Belarus (Alexandrovich et al. 1996), Denmark (Silfverberg 2004).

*dilutum* **Erichson, 1839**. Silfverberg 1992, 2004; Pileckis and Monsevičius 1995; Tamutis 2002b, c; Smetana 2004g; Alonso-Zarazaga 2009a.

*elongatum* **(Linnaeus, 1767)**. Pileckis 1976a; Monsevičius1986b, 1997; Strazdienė 1988; Silfverberg 1992, 2004; Gaidienė 1993; Pileckis and Monsevičius 1995; Smetana 2004g; Alonso-Zarazaga 2009a.

*fovulum* **Stephens, 1833**. Monsevičius 1982, 1987, 1997; Silfverberg 1992, 2004; Gaidienė 1993; Pileckis and Monsevičius 1995; Smetana 2004g; Alonso-Zarazaga 2009a.

*fulvipenne* **Gravenhorst, 1806**. Pileckis 1960, 1976a; Silfverberg 1992, 2004; Gaidienė 1993; Pileckis and Monsevičius 1995; Monsevičius 1997; Tamutis et al. 2004; Smetana 2004g; Ferenca 2006b; Alonso-Zarazaga 2009a.

*geminum* **Kraatz, 1857** = *boreale* Hochhuth, 1851 = *volgense* Hochhuth, 1851.Pileckis 1960, 1976a; Dvilevičius et al. 1988; Silfverberg 1992, 2004; Gaidienė 1993; Pileckis and Monsevičius 1995; Monsevičius 1997; Tamutis and Zolubas 2001; Tamutis 2002b; Smetana 2004g; Tamutis et al. 2004, 2006, 2007; Ferenca 2006b; Alonso-Zarazaga 2009a.

*impressum* **Heer, 1841** = *filiforme* Gravenhorst, 1806, nec (Fabricius, 1793).Pileckis 1976a; Monsevičius 1987, 1997; Silfverberg 1992, 2004; Pileckis and Monsevičius 1995; Smetana 2004g; Alonso-Zarazaga 2009a.

*laevipenne* **Heer, 1839**.Tamutis 2003.

*longulum* **Gravenhorst, 1802** = *patris* G. Benick, 1950.Pileckis 1976a; Monsevičius 1986b, 1997; Silfverberg 1992, 2004; Pileckis and Monsevičius 1995; Smetana 2004g; Alonso-Zarazaga 2009a.

*pallidipenne* **Hochhuth, 1851** = *ripicola* Czwalina, 1888.Monsevičius and Pankevičius 2001; Tamutis 2002c.

[*pallidum* **Nordmann, 1837**]. Known in Denmark, Estonia, southern Sweden (Lundberg and Gustafsson 1995), northern Poland (Burakowski et al. 1979).

*rufipenne* **Gyllenhal, 1813**. Monsevičius 1982, 1986b, 1987, 1997; Silfverberg 1992, 2004; Pileckis and Monsevičius 1995; Smetana 2004g; Alonso-Zarazaga 2009a.

*Achenium* **Samouelle, 1819**.

[*humile* **(Nicolai, 1822)**]. Known in Denmark, southern Sweden (Lundberg and Gustafsson 1995), Estonia (Silfverberg 2004), northern Poland (Burakowski et al. 1979).

*Ochthephilum* **Stephens, 1829** = *Cryptobium* Mannerheim, 1830.

[*collare* **(Reitter, 1884)**]. Known in Denmark, southern Sweden (Lundberg and Gustafsson 1995).

*fracticorne* **(Paykull, 1800)**. Pileckis 1960, 1976a; Monsevičius 1986b, 1987, 1997; Dvilevičius et al. 1988; Silfverberg 1992, 2004; Gaidienė 1993; Pileckis and Monsevičius 1995; Smetana 2004g; Alonso-Zarazaga 2009a.

**Staphylininae Latreille, 1802**.

**Xantholinini Erichson, 1839**.

*Leptacinus* **Erichson, 1839**.

*batychrus* **(Gyllenhal, 1827)**. Roubal 1910; Pileckis 1960, 1976a; Silfverberg 1992, 2004; Gaidienė 1993; Pileckis and Monsevičius 1995; Smetana 2004g; Alonso-Zarazaga 2009a.

*formicetorum* **Märkel, 1841**. Monsevičius 1985, 1997; Silfverberg 1992, 2004; Pileckis and Monsevičius 1995.

*intermedius* **Donisthorpe, 1936**. Monsevičius 1985; Silfverberg 1992, 2004; Pileckis and Monsevičius 1995.

*pusillus* **(Stephens, 1833)** = *linearis* (Gravenhorst, 1802) nec (Olivier, 1793).Roubal 1910; Pileckis 1960, 1976a; Silfverberg 1992, 2004; Pileckis and Monsevičius 1995; Smetana 2004g; Alonso-Zarazaga 2009a.

*sulcifrons* **(Stephens, 1833)** = *othioides* auct. nec Baudi, 1870.Silfverberg 1992, 2004; Pileckis and Monsevičius 1995; Monsevičius 1997; Alonso-Zarazaga 2009a.

*Phacophallus* **Coiffait, 1956**.

*parumpunctatus* **(Gyllenhal, 1827)**. Smetana 2004g; Alonso-Zarazaga 2009a.

*Gauropterus* **Thomson, 1860**.

[*fulgidus* **(Fabricius, 1787)**]. Known in Latvia (Telnov 2004), Denmark, throughout Sweden (Lundberg and Gustafsson 1995), Poland (Szujecki 1976).

*Gyrohypnus* **Leach, 1819**.

*angustatus* **Stephens, 1833** = *scoticus* (Joy, 1913).Pileckis 1960, 1976a; Dvilevičius et al. 1988; Silfverberg 1992, 2004; Gaidienė 1993; Pileckis and Monsevičius 1995; Monsevičius 1997; Tamutis and Zolubas 2001; Tamutis 2002b; Tamutis et al. 2004; Smetana 2004g; Ferenca 2006b; Alekseev 2008a; Alonso-Zarazaga 2009a.

*atratus* **(Heer, 1839)**. Monsevičius 1985, 1997; Silfverberg 1992, 2004; Pileckis and Monsevičius 1995; Smetana 2004g; Alonso-Zarazaga 2009a.

*fracticornis* **(O.F. Müller, 1776)**. Pileckis 1976a; Silfverberg 1992, 2004; Gaidienė 1993; Pileckis and Monsevičius 1995; Monsevičius 1987; Smetana 2004g; Alonso-Zarazaga 2009a.

*liebei* **(Scheerpeltz, 1926**) = *punctulatus* (Paykull, 1789) = *ater* auct. nec (Stephens, 1833).Pileckis 1976a; Bercio and Folwaczny 1979; Monsevičius 1986b, 1987, 1997; Silfverberg 1992, 2004; Pileckis and Monsevičius 1995; Tamutis and Zolubas 2001; Smetana 2004g; Alonso-Zarazaga 2009a.

*Nudobius* **Thomson, 1860**.

*lentus* **(Gravenhorst, 1806)**. Jakaitis and Valenta 1976; Pileckis 1976a; Monsevičius 1987, 1997; Silfverberg 1992, 2004; Gaidienė 1993; Pileckis and Monsevičius 1995; Tamutis and Zolubas 2001; Smetana 2004g; Alonso-Zarazaga 2009a.

*Megalinus* **Mulsant & Rey, 1877**.

*glabratus* **(Gravenhorst, 1802)**. Silfverberg 1992, 2004; Smetana 2004g; Alonso-Zarazaga 2009a.

*Hypnogyra* **Casey, 1906** = *Phalacrolinus* Coiffait, 1972.

*angularis* **(Ganglbauer, 1895)** = *glabra* (Gravenhorst, 1806) nec (O.F. Müller, 1776).Pileckis 1976a (*Xantholinus glaber*); Silfverberg 1992, 2004; Pileckis and Monsevičius 1995; Smetana 2004g; Alonso-Zarazaga 2009a.

*Leptophius* **Coiffait, 1983**.

*flavocinctus* **(Hochhuth, 1849)** = *relucens* auct. nec (Gravenhorst, 1806).Silfverberg 2004.

*Xantholinus* **Dejean, 1821**.

[*audrasi* **Coiffait, 1956** = *strandi* Coiffait, 1958].Known in Estonia, southern Sweden (Lundberg and Gustafsson 1995), Denmark (Alonso-Zarazaga 2009a), recently found in Latvia (Cibulskis 2006).

*distans* **Mulsant & Rey, 1853**. Roubal 1910; Pileckis 1960, 1976a; Silfverberg 1992, 2004; Gaidienė 1993; Pileckis and Monsevičius 1995; Smetana 2004g; Alonso-Zarazaga 2009a.

[*dvoraki* **Coiffait, 1956 =** *dissimilis* Coiffait, 1956 = *roubali* Coiffait, 1956].Known in Denmark, Estonia, southern Sweden (Lundberg and Gustafsson 1995), Poland (Szujecki 1976).

[*gallicus* **Coiffait, 1956** = *rhenanus* Coiffait, 1962].Known in Latvia, Denmark, Estonia (Silfverberg 2004), Poland (Szujecki 1976).

*laevigatus* **Jacobsen, 1847** = *clairei* Coiffait, 1956.Monsevičius 1987, 1997; Silfverberg 1992, 2004; Gaidienė 1993; Pileckis and Monsevičius 1995; Ivinskis et al. 2009.

*linearis* **(Olivier, 1794)**. Ivinskis et al. 1984; Monsevičius 1987, 1997; Dvilevičius et al. 1988; Silfverberg 1992, 2004; Gaidienė 1993; Pileckis and Monsevičius 1995; Smetana 2004g; Dapkus and Tamutis 2007; Alonso-Zarazaga 2009a.

*longiventris* **Heer, 1839**. Monsevičius 1982, 1986b, 1987, 1997; Dvilevičius et al. 1988; Silfverberg 1992, 2004; Gaidienė 1993; Pileckis and Monsevičius 1995; Tamutis and Zolubas 2001; Tamutis et al. 2004; Smetana 2004g; Alekseev 2008a; Alonso-Zarazaga 2009a.

*tricolor* **(Fabricius, 1787)** = *meyeri* Drugmand, 1994.Monsevičius 1982, 1986b, 1987, 1997; Dvilevičius et al. 1988; Strazdienė 1981, 1988; Silfverberg 1992, 2004; Pileckis and Monsevičius 1995; Smetana 2004g; Dapkus and Tamutis 2007; Alonso-Zarazaga 2009a.

**Othiini Thomson, 1859**.

*Othius* **Stephens, 1829**.

*angustus* **Stephens, 1833** = *melanocephalus* (Gravenhorst, 1806) nec (Geoffroy, 1785).Tamutis 2003; Smetana 2004g; Alonso-Zarazaga 2009a.

*laeviusculus* **Stephens, 1832**. Gaidienė 1993; Silfverberg 2004.

*lapidicola* **Märkel & Kiesenwetter, 1847**. Pileckis 1976a; Monsevičius 1987; Silfverberg 1992, 2004; Pileckis and Monsevičius 1995.

*punctulatus* **(Goeze, 1777)**. Bercio and Folwaczny 1979; Monsevičius 1982, 1986b, 1997; Dvilevičius et al. 1988; Silfverberg 1992, 2004; Gaidienė 1993; Pileckis and Monsevičius 1995; Smetana 2004g; Alonso-Zarazaga 2009a.

*subuliformis* **Stephens, 1833** = *myrmecophilus* Kiesenwetter, 1843.Roubal 1910; Pileckis 1960, 1976a; Jakaitis and Valenta 1976; Bercio and Folwaczny 1979; Ivinskis et al. 1984; Monsevičius 1986b, 1987, 1997; Dvilevičius et al. 1988; Silfverberg 1992, 2004; Gaidienė 1993; Pileckis and Monsevičius 1995; Smetana 2004g; Alonso-Zarazaga 2009a.

[*volans* **J.R. Sahlberg, 1876**]. Known in Latvia (Silfverberg 2004), Estonia, Sweden (Lundberg and Gustafsson 1995).

*Atrecus* **Jacquelin du Val, 1856** = *Baptolinus* Kraatz, 1858.

*affinis* **(Paykull, 1789)**. Jakaitis and Valenta 1976; Pileckis 1976a; Bercio and Folwaczny 1979; Silfverberg 1992, 2004; Gaidienė 1993; Pileckis and Monsevičius 1995; Monsevičius 1997; Alonso-Zarazaga 2009a.

*longiceps* **(Fauvel, 1872)**. Pileckis 1976a; Monsevičius 1987, 1998; Silfverberg 1992, 2004; Pileckis and Monsevičius 1995; Smetana 2004g; Alonso-Zarazaga 2009a.

*pilicornis* **(Paykull, 1790)**. Silfverberg 1992, 2004; Monsevičius 1997, 1998; Löbl and Smetana 2004; Smetana 2004g; Alonso-Zarazaga 2009a.

**Staphylinini Latreille, 1802**.

*Erichsonius* **Fauvel, 1874** = *Actobius* Fauvel, 1876.

*cinerascens* **(Gravenhorst, 1802)**. Monsevičius 1982, 1986b, 1987, 1997; Silfverberg 1992, 2004; Gaidienė 1993; Pileckis and Monsevičius 1995; Smetana 2004g; Alonso-Zarazaga 2009a.

[*signaticornis* **(Mulsant & Rey, 1853)**].Known in northwestern Poland (Szujecki 1980).

*Gabrius* **Stephens, 1829**.

*appendiculatus* **Sharp, 1910** = *subnigritulus* (Reitter, 1909).Silfverberg 1992, 2004; Pileckis and Monsevičius 1995; Monsevičius 1997; Tamutis and Zolubas 2001; Smetana 2004g; Alonso-Zarazaga 2009a.

*astutoides* **(Strand, 1946)**.Tamutis and Ferenca 2006; Ferenca et al. 2006, 2007.

*austriacus* **Scheerpeltz, 1947** = *velox* auct. nec (Sharp, 1910).Pileckis 1976a; Monsevičius 1986b, 1997; Silfverberg 1992, 2004; Pileckis and Monsevičius 1995; Smetana 2004g; Alonso-Zarazaga 2009a.

*breviventer* **(Sperk, 1835)** = *coxalus* (Hochhuth, 1871) = *pennatus* Sharp, 1910.Monsevičius 1986b, 1987, 1997; Silfverberg 1992, 2004; Gaidienė 1993; Pileckis and Monsevičius 1995; Tamutis 2002b; Tamutis et al. 2004; Smetana 2004g; Alonso-Zarazaga 2009a.

[*exiguus* **(Nordmann, 1837)**]. Known in Denmark, Estonia, southern Sweden (Lundberg and Gustafsson 1995), Poland (Szujecki 1980), Belarus (Alexandrovich et al. 1996).

[*expectatus* **Smetana, 1952**]. Known in Estonia, Sweden (Lundberg and Gustafsson 1995), Poland (Szujecki 1980), northern and northwestern Belarus (Alexandrovich et al. 1996).

[*keysianus* **Sharp, 1910**]. Known in Denmark (Lundberg and Gustafsson 1995).

*lividipes* **(Baudi, 1848)**. Horion 1965; Bercio and Folwaczny 1979; Silfverberg 1992, 2004; Pileckis and Monsevičius 1995; Smetana 2004g; Alonso-Zarazaga 2009a.

*nigritulus* **(Gravenhorst, 1802)**. Pileckis 1976a; Silfverberg 1992, 2004; Gaidienė 1993; Pileckis and Monsevičius 1995; Smetana 2004g; Alonso-Zarazaga 2009a.

*osseticus* **(Kolenati, 1846)** = *vernalis* (Gravenhorst, 1802) nec (O.F. Müller, 1776).Pileckis 1976a; Dvilevičius et al. 1988; Silfverberg 1992, 2004; Gaidienė 1993; Pileckis and Monsevičius 1995; Monsevičius 1997; Smetana 2004g.

[*piliger* **Mulsant & Rey, 1876**]. Known in Estonia, southern Sweden (Lundberg and Gustafsson 1995), Poland (Szujecki 1980), Denmark (Alonso-Zarazaga 2009a), northern Belarus (Alexandrovich et al. 1996).

[*sphagnicola* **(Sjöberg, 1950)**]. Known in Latvia (Spuņģis 2008), Estonia, throughout Sweden (Lundberg and Gustafsson 1995), Poland (Szujecki 1980).

*splendidulus* **(Gravenhorst, 1802)**. Jakaitis and Valenta 1976; Pileckis 1976a; Monsevičius 1987, 1997; Silfverberg 1992, 2004; Gaidienė 1993; Pileckis and Monsevičius 1995; Tamutis and Zolubas 2001; Smetana 2004g; Alonso-Zarazaga 2009a.

[*subnigritulus* **Joy, 1913** = *subnigrituloides* (Scheerpeltz, 1933)].Known in Estonia (Lundberg and Gustafsson 1995), northwestern Belarus (Alexandrovich et al. 1996).

[*toxotes* **Joy, 1913**]. Known in Denmark, Estonia, Sweden (Lundberg and Gustafsson 1995).

*trossulus* **(Nordmann, 1837)**. Monsevičius 1986b, 1997; Silfverberg 1992, 2004; Gaidienė 1993; Pileckis and Monsevičius 1995; Smetana 2004g; Alonso-Zarazaga 2009a.

*Bisnius* **Stephens, 1829**. (*Philonthus*)

*cephalotes* **(Gravenhorst, 1802)**. Pileckis 1976a; Silfverberg 1992, 2004; Gaidienė 1993; Pileckis and Monsevičius 1995; Smetana 2004g; Alonso-Zarazaga 2009a.

*fimetarius* **(Gravenhorst, 1802)** = *rigidicornis* (Gravenhorst, 1802).Roubal 1910; Pileckis 1960, 1976a; Silfverberg 1992, 2004; Gaidienė 1993; Pileckis and Monsevičius 1995; Monsevičius 1997; Smetana 2004g; Alonso-Zarazaga 2009a.

[*nigriventris* **(Thomson, 1867)**]. Known in Latvia (Telnov 2004), Denmark, throughout Sweden (Lundberg and Gustafsson 1995).

*nitidulus* **(Gravenhorst, 1802)**. Pileckis 1976a; Silfverberg 1992, 2004; Pileckis and Monsevičius 1995; Smetana 2004g; Alonso-Zarazaga 2009a.

[*parcus* **(Sharp, 1874)**]. Known in Denmark, southern Sweden (Lundberg and Gustafsson 1995).

[*pseudoparcus* **(Brunne, 1976)**]. Known in Denmark, southern Sweden (Lundberg and Gustafsson 1995).

*puella* **(Nordmann, 1837)**. Pileckis 1976a; Monsevičius 1987, 1997; Silfverberg 1992, 2004; Pileckis and Monsevičius 1995; Smetana 2004g; Alonso-Zarazaga 2009a.

*scribae* **(Fauvel 1867)** = *varipennis* (W. Scriba, 1864).Pileckis and Monsevičius 1995; Silfverberg 2004.

*sordidus* **(Gravenhorst, 1802)** = *pachycephalus* (Nordmann 1837).Pileckis 1976a; Silfverberg 1992, 2004; Gaidienė 1993; Pileckis and Monsevičius 1995; Smetana 2004g; Alonso-Zarazaga 2009a.

*spermophili* **(Ganglbauer, 1897)**. Monsevičius 1997, 1999; Tamutis et al. 2004.

*subuliformis* **(Gravenhorst, 1802)** = *fuscus* (Gravenhorst, 1802) nec (Gmelin, 1790).Pileckis 1976a; Silfverberg 1992, 2004; Pileckis and Monsevičius 1995; Monsevičius 1997, 1999; Smetana 2004g; Alonso-Zarazaga 2009a.

*Rabigus* **Mulsant & Rey, 1876** (*Philonthus*).

*tenuis* **(Fabricius, 1793)**. Pileckis 1960, 1976a; Silfverberg 1992, 2004; Gaidienė 1993; Pileckis and Monsevičius 1995; Monsevičius 1998; Smetana 2004g; Ferenca 2006b; Alonso-Zarazaga 2009a.

*Gabronthus* **Tottenham, 1955** (*Philonthus*).

[*thermarum* **(Aubé, 1850)**]. Known in Denmark, Estonia, southern Sweden (Lundberg and Gustafsson 1995), Poland (Szujecki 1980).

*Philonthus* **Stephens, 1829** = *Spatulonthus* Tottenham, 1955.

*addendus* **Sharp, 1867**. Pileckis 1960, 1976a; Silfverberg 1992, 2004; Gaidienė 1993; Pileckis and Monsevičius 1995; Monsevičius 1997; Smetana 2004g; Ferenca 2006b; Tamutis et al. 2007; Alonso-Zarazaga 2009a.

*albipes* **(Gravenhorst, 1802)**.Roubal 1910; Pileckis 1960, 1976a; Silfverberg 1992, 2004; Gaidienė 1993; Pileckis and Monsevičius 1995; Monsevičius 1997; Smetana 2004g; Ferenca 2006b; Alonso-Zarazaga 2009a.

*alpinus* **Eppelsheim, 1875**. Monsevičius 1987, 1997; Silfverberg 1992, 2004; Smetana 2004g; Alonso-Zarazaga 2009a.

*atratus* **(Gravenhorst, 1802)**. Mazurowa and Mazur 1939; Pileckis 1960, 1976a; Zajančkauskas and Pileckis 1968; Monsevičius 1986b, 1997; Dvilevičius et al. 1988; Silfverberg 1992, 2004; Gaidienė 1993; Pileckis and Monsevičius 1995; Smetana 2004g; Ferenca 2006b; Alonso-Zarazaga 2009a.

*binotatus* **(Gravenhorst, 1802)**. Pileckis and Monsevičius 1995; Silfverberg 2004.

[*caerulescens* **(Lacordaire, 1835)**]. Known in Latvia (Telnov 2004), Poland (Szujecki 1980).

*carbonarius* **(Gravenhorst, 1802)** = *varius* (Gyllenhal, 1810).Pileckis 1960, 1976a; Zajančkauskas and Pileckis 1968; Monsevičius 1986b, 1987, 1997; Dvilevičius et al. 1988; Silfverberg 1992, 2004; Gaidienė 1993; Pileckis and Monsevičius 1995; Tamutis and Zolubas 2001; Tamutis 1999, 2002b, c; Tamutis et al. 2004, 2006, 2007; Smetana 2004g; Ferenca 2006b; Vaivilavičius 2008; Alonso-Zarazaga 2009a.

[*caucasicus* **Nordmann, 1837** = *dimidiatus* (C.R. Sahlberg, 1830, nec Panzer, 1796].Known in northwestern Belarus (Alexandrovich et al. 1996), Poland (Szujecki 1980).

[*cyanipennis* **(Fabricius, 1793)**]. Known in Latvia (Telnov 2004), northwestern Belarus (Alexandrovich et al. 1996), Poland (Szujecki 1980).

*cognatus* **Stephens, 1832** = *fuscipennis* (Mannerheim, 1830) nec (Block, 1799).Pileckis 1960, 1976a; Monsevičius 1986b, 1987, 1997; Silfverberg 1992, 2004; Gaidienė 1993; Pileckis and Monsevičius 1995; Tamutis 1999, 2002b; Gliaudys 2001; Tamutis et al. 2004, 2006, 2007; Smetana 2004g; Ferenca 2006b; Vaivilavičius 2008; Alonso-Zarazaga 2009a.

*concinnus* **(Gravenhorst, 1802)** = *ochropus* (Gravenhorst, 1802).Pileckis 1960, 1976a; Silfverberg 1992, 2004; Gaidienė 1993; Pileckis and Monsevičius 1995; Tamutis 2002c; Smetana 2004g; Ferenca 2006b; Alonso-Zarazaga 2009a.

[*confinis* **Strand, 1941** = *isthmius* Kangas, 1979].Known in Latvia (Cibulskis 2010), Denmark, southern Sweden (Lundberg and Gustafsson 1995), Poland (Szujecki 1980).

*coprophilus* **Jarrige, 1949**. Monsevičius 1988a, 1997; Silfverberg 1992, 2004; Pileckis and Monsevičius 1995; Smetana 2004g; Alonso-Zarazaga 2009a.

*corruscus* **(Gravenhorst, 1802)**. Silfverberg 1992, 2004; Pileckis and Monsevičius 1995; Smetana 2004g; Alonso-Zarazaga 2009a.

*corvinus* **Erichson, 1839**. Roubal 1910; Pileckis 1960, 1976a; Zajančkauskas and Pileckis 1968; Silfverberg 1992, 2004; Gaidienė 1993; Pileckis and Monsevičius 1995; Monsevičius 1997; Smetana 2004g; Ferenca 2006b; Alonso-Zarazaga 2009a.

*cruentatus* **(Gmelin, 1790)**. Roubal 1910; Pileckis 1960, 1976a; Jakaitis and Valenta 1976; Bercio and Folwaczny 1979; Monsevičius 1986b, 1997; Silfverberg 1992, 2004; Gaidienė 1993; Pileckis and Monsevičius 1995; Smetana 2004g; Ferenca 2006b; Alonso-Zarazaga 2009a.

*debilis* **(Gravenhorst, 1802)**. Pileckis 1960, 1976a; Bercio and Folwaczny 1979; Silfverberg 1992, 2004; Gaidienė 1993; Pileckis and Monsevičius 1995; Smetana 2004g; Ferenca 2006b; Alonso-Zarazaga 2009a.

*decorus* **(Gravenhorst, 1802)**. Roubal 1910; Pileckis 1960, 1976a; Dvilevičius et al. 1988; Silfverberg 1992, 2004; Gaidienė 1993; Pileckis and Monsevičius 1995; Monsevičius 1997; Gliaudys 2001; Tamutis and Zolubas 2001; Tamutis 2002b; Tamutis et al. 2004; Smetana 2004g; Ferenca 2006b; Vaivilavičius 2008; Alonso-Zarazaga 2009a.

*discoideus* **(Gravenhorst, 1802)**. Pileckis 1960, 1976a; Silfverberg 1992, 2004; Pileckis and Monsevičius 1995; Monsevičius 1997; Smetana 2004g; Ferenca 2006b; Alonso-Zarazaga 2009a.

*ebeninus* **(Gravenhorst, 1802)**. Mazurowa and Mazur 1939; Pileckis 1960, 1976a; Silfverberg 1992, 2004; Gaidienė 1993; Pileckis and Monsevičius 1995; Smetana 2004g; Ferenca 2006b; Alonso-Zarazaga 2009a.

*fumarius* **(Gravenhorst, 1806)**. Monsevičius 1986b, 1997; Silfverberg 1992, 2004; Pileckis and Monsevičius 1995; Smetana 2004g; Alonso-Zarazaga 2009a.

*furcifer* **Renkonen, 1937**. Silfverberg 1992, 2004; Gaidienė 1993; Pileckis and Monsevičius 1995; Monsevičius 1997; Smetana 2004g; Alonso-Zarazaga 2009a.

*intermedius* **(Lacordaire, 1835)**. Pileckis 1976a; Silfverberg 1992, 2004; Pileckis and Monsevičius 1995; Tamutis 2002b, c; Tamutis et al. 2004; Smetana 2004g; Alonso-Zarazaga 2009a.

[*jurgans* **Tottenham, 1937**]. Known in Denmark, southern Sweden (Lundberg and Gustafsson 1995), Poland (Szujecki 1980).

*laevicollis* **(Lacordaire, 1835)**. Pileckis 1976a; Silfverberg 1992, 2004; Gaidienė 1993; Pileckis and Monsevičius 1995; Monsevičius 1997; Alonso-Zarazaga 2009a.

*laminatus* **(Creutzer, 1799)**. Monsevičius 1982, 1986b, 1997; Dvilevičius et al. 1988; Silfverberg 1992, 2004; Gaidienė 1993; Pileckis and Monsevičius 1995; Tamutis 2002b; Tamutis et al. 2004; Smetana 2004g; Alonso-Zarazaga 2009a.

*lepidus* **(Gravenhorst, 1802)**. Pileckis 1976a; Silfverberg 1992, 2004; Gaidienė 1993; Pileckis and Monsevičius 1995; Smetana 2004g; Alonso-Zarazaga 2009a; Ivinskis et al. 2009.

*linki* **Solsky, 1866**. Horion 1951; Silfverberg 1992, 2004; Pileckis and Monsevičius 1995.

*longicornis* **Stephens, 1832**. Mazurowa and Mazur 1939; Pileckis 1960, 1976a; Silfverberg 1992, 2004; Gaidienė 1993; Pileckis and Monsevičius 1995; Smetana 2004g; Ferenca 2006b; Alonso-Zarazaga 2009a.

*mannerheimi* **Fauvel, 1868**. Pileckis 1960, 1976a; Zajančkauskas and Pileckis 1968; Silfverberg 1992, 2004; Gaidienė 1993; Pileckis and Monsevičius 1995; Monsevičius 1997; Tamutis 2002b, c; Tamutis et al. 2004; Smetana 2004g; Ferenca 2006b; Alonso-Zarazaga 2009a.

*marginatus* **(O.F. Müller, 1764)**. Pileckis 1960, 1976a; Monsevičius 1986b, 1987, 1997; Dvilevičius et al. 1988; Silfverberg 1992, 2004; Gaidienė 1993; Pileckis and Monsevičius 1995; Smetana 2004g; Ferenca 2006b; Alonso-Zarazaga 2009a.

*micans* **(Gravenhorst, 1802)**. Pileckis 1960, 1976a; Silfverberg 1992, 2004; Gaidienė 1993; Pileckis and Monsevičius 1995; Monsevičius 1997; Smetana 2004g; Ferenca 2006b; Alonso-Zarazaga 2009a.

*micantoides* **G. Benick & Lohse, 1956**. Monsevičius 1988, 1997; Silfverberg 1992, 2004; Pileckis and Monsevičius 1995; Tamutis et al. 2004; Smetana 2004g; Alonso-Zarazaga 2009a.

*montivagus* **Heer, 1839**. Pileckis 1960, 1976a; Silfverberg 1992, 2004; Gaidienė 1993; Ferenca 2006b.

*nigrita* **(Gravenhorst, 1806)**. Pileckis 1960, 1976a; Monsevičius 1986b, 1987, 1997; Silfverberg 1992, 2004; Gaidienė 1993; Pileckis and Monsevičius 1995; Smetana 2004g; Ferenca 2006b; Alonso-Zarazaga 2009a.

*nitidicollis* **(Lacordaire, 1835)** = *bimaculatus* (Gravenhorst, 1802) nec (Schrank, 1798). Pileckis and Monsevičius 1995; Silfverberg 2004.

*nitidus* **(Fabricius, 1787)**. Roubal 1910; Mazurowa and Mazur 1939; Pileckis 1960, 1976a; Silfverberg 1992, 2004; Gaidienė 1993; Pileckis and Monsevičius 1995; Monsevičius 1997; Smetana 2004g; Ferenca 2006b; Alonso-Zarazaga 2009a.

*parvicornis* **(Gravenhorst, 1802)** = *agilis* (Gravenhorst, 1806).Pileckis 1976a; Silfverberg 1992, 2004; Gaidienė 1993; Pileckis and Monsevičius 1995; Monsevičius 1997; Smetana 2004g; Alonso-Zarazaga 2009a.

*politus* **(Linnaeus, 1758)**. Roubal 1910; Pileckis 1960, 1976a; Monsevičius 1987, 1997; Silfverberg 1992, 2004; Gaidienė 1993; Pileckis and Monsevičius 1995; Smetana 2004g; Alonso-Zarazaga 2009a.

[*pseudovarians* **Strand, 1941**]. Known in Latvia (Cibulskis 2010), Denmark, Estonia (Lundberg and Gustafsson 1995), Poland (Szujecki 1980).

*punctus* **(Gravenhorst, 1802)**. Pileckis 1976a; Silfverberg 1992, 2004; Pileckis and Monsevičius 1995; Smetana 2004g; Alonso-Zarazaga 2009a.

*quisquiliarius* **(Gyllenhal, 1810)**. Pileckis 1976a; Monsevičius 1987, 1997; Silfverberg 1992, 2004; Gaidienė 1993; Pileckis and Monsevičius 1995; Smetana 2004g; Alekseev 2008a; Alonso-Zarazaga 2009a.

*rectangulus* **Sharp, 1874**. Monsevičius 1988a, 1997; Silfverberg 1992, 2004; Gaidienė 1993; Pileckis and Monsevičius 1995; Smetana 2004g; Alonso-Zarazaga 2009a.

*rotundicollis* **(Ménétriés, 1832)**. Pileckis 1960, 1976a; Silfverberg 1992, 2004; Gaidienė 1993; Pileckis and Monsevičius 1995; Monsevičius 1997, 1998; Tamutis 1999, 2002b, c; Tamutis and Zolubas 2001; Tamutis et al. 2004, 2007; Smetana 2004g; Ferenca 2006b; Alonso-Zarazaga 2009a.

*rubripennis* **Stephens,** 1832 = *fulvipes* (Fabricius, 1792) nec (Scopoli, 1763).Roubal 1910; Mazurowa and Mazur 1939; Pileckis 1960, 1976a; Silfverberg 1992, 2004; Gaidienė 1993; Pileckis and Monsevičius 1995; Monsevičius 1997; Smetana 2004g; Ferenca 2006b; Alonso-Zarazaga 2009a.

*rufipes* **(Stephens, 1832)**. Pileckis 1960, 1976a; Bercio and Folwaczny 1979; Silfverberg 1992, 2004; Gaidienė 1993; Pileckis and Monsevičius 1995; Smetana 2004g; Ferenca 2006b; Alonso-Zarazaga 2009a.

[*salinus* **Kiesenwetter, 1844**]. Known in Denmark, southern Sweden (Lundberg and Gustafsson 1995).

*sanguinolentus* **(Gravenhorst, 1802)**. Roubal 1910; Mazurowa and Mazur 1939; Pileckis 1960, 1976a; Bercio and Folwaczny 1979; Silfverberg 1992, 2004; Gaidienė 1993; Pileckis and Monsevičius 1995; Smetana 2004g; Ferenca 2006b; Alonso-Zarazaga 2009a.

*spinipes* **Sharp, 1874**. Monsevičius 1988a, 1997; Silfverberg 1992, 2004; Pileckis and Monsevičius 1995; Tamutis et al. 2004.

*splendens* **(Fabricius, 1793)**. Mazurowa and Mazur 1939; Pileckis 1960, 1976a; Lešinskas and Pileckis 1967; Bercio and Folwaczny 1979; Silfverberg 1992, 2004; Gaidienė 1993; Pileckis and Monsevičius 1995; Monsevičius 1997; Gliaudys 2001; Tamutis 2002b; Tamutis et al. 2004; Smetana 2004g; Ferenca 2006b; Alonso-Zarazaga 2009a.

*subvirescens* **(Thomson, 1884)**. Pileckis and Monsevičius 1995; Silfverberg 2004.

*succicola* **Thomson, 1860** = *chalceus* Ganglbauer, 1895, nec Stephens, 1832.Pileckis 1976a; Monsevičius 1987, 1997; Silfverberg 1992, 2004; Gaidienė 1993; Pileckis and Monsevičius 1995; Tamutis et al. 2004; Smetana 2004g.

[*temporalis* **Mulsant & Rey, 1853**]. Known in Latvia (Telnov 2004), Estonia (Lundberg and Gustafsson 1995), Poland (Szujecki 1980).

*tenuicornis* **Mulsant & Rey, 1853** = *carbonarius* (Gyllenhal, 1810) nec (Gravenhorst, 1802).Roubal 1910; Pileckis 1960, 1976a; Monsevičius 1986b, 1997; Silfverberg 1992, 2004; Gaidienė 1993; Pileckis and Monsevičius 1995; Tamutis and Zolubas 2001; Smetana 2004g; Alonso-Zarazaga 2009a.

*umbratilis* **(Gravenhorst, 1802)**. Silfverberg 1992, 2004; Pileckis and Monsevičius 1995; Smetana 2004g; Alonso-Zarazaga 2009a.

*varians* **(Paykull, 1789)**. Roubal 1910; Mazurowa and Mazur 1939; Pileckis 1960, 1976a; Monsevičius 1987, 1997; Dvilevičius et al. 1988; Silfverberg 1992, 2004; Gaidienė 1993; Pileckis and Monsevičius 1995; Smetana 2004g; Ferenca 2006b; Alonso-Zarazaga 2009a.

*ventralis* **(Gravenhorst, 1802)** = *immundus* (Gravenhorst, 1806).Pileckis 1976a; Silfverberg 1992, 2004; Gaidienė 1993; Pileckis and Monsevičius 1995; Tamutis and Zolubas 2001; Smetana 2004g; Alonso-Zarazaga 2009a; Ivinskis et al. 2009.

*Neobisnius* **Ganglbauer, 1895**.

[*lathrobioides* **(Baudi, 1848)** = *cerrutii* Gridelli, 1943].Known in Latvia (Vorst et al. 2007), Denmark, throughout Sweden (Lundberg and Gustafsson 1995), Estonia (Silfverberg 2004), Poland (Szujecki 1980).

[*procerulus* **(Gravenhorst, 1806)**]. Known in Denmark, Estonia, southern Sweden (Lundberg and Gustafsson 1995), Poland (Szujecki 1980).

*villosulus* **(Stephens, 1833)**. Pileckis 1976a; Silfverberg 1992, 2004; Pileckis and Monsevičius 1995; Monsevičius 1998; Smetana 2004g; Alonso-Zarazaga 2009a.

*Remus* **Holme, 1837**.

[*sericeus* **Holme, 1837**]. Known in Latvia (Telnov 2004), Denmark, southern Sweden (Lundberg and Gustafsson 1995).

*Cafius* **Stephens, 1826**.

*xantholoma* **(Gravenhorst, 1806)**. Horion 1965; Bercio and Folwaczny 1979; Silfverberg 1992, 2004; Pileckis and Monsevičius 1995; Smetana 2004g; Alonso-Zarazaga 2009a.

*Dinothenarus* **Thomson, 1858**.

*pubescens* **(DeGeer, 1774)**. Pileckis 1960, 1976a; Silfverberg 1992, 2004; Gaidienė 1993; Pileckis and Monsevičius 1995; Monsevičius 1997; Smetana 2004g; Ferenca 2006b; Alonso-Zarazaga 2009a.

*Ontholestes* **Ganglbauer, 1895**.

*murinus* **(Linnaeus, 1758)**. Eichwald 1830; Mazurowa and Mazur 1939; Pileckis 1960, 1976a; Silfverberg 1992, 2004; Gaidienė 1993; Pileckis and Monsevičius 1995; Monsevičius 1997; Šablevičius 2000b; Gliaudys 2001; Smetana 2004g; Ferenca 2006b; Tamutis et al. 2007; Alonso-Zarazaga 2009a.

*tessellatus* **(Geoffroy, 1785)**. Pileckis 1960, 1976a; Silfverberg 1992, 2004; Gaidienė 1993; Pileckis and Monsevičius 1995; Monsevičius 1997; Smetana 2004g; Ferenca 2006b; Alonso-Zarazaga 2009a; Šablevičius 2011.

*Emus* **Leach, 1819**.

*hirtus* **(Linnaeus, 1758)**. Mazurowa and Mazur 1939; Pileckis 1960, 1976a; Bercio and Folwaczny 1979; Silfverberg 1992, 2004; Gaidienė 1993; Pileckis and Monsevičius 1995; Šablevičius 2000b, 2011; Ferenca 2004; Smetana 2004g; Alonso-Zarazaga 2009a.

*Platydracus* **Thomson, 1858**.

[*chalcocephalus* **(Fabricius, 1801)**]. Known in Latvia (Telnov 2004), Belarus (Alexandrovich et al. 1996), Denmark (Lundberg and Gustafsson 1995), Estonia (Silfverberg 2004), Poland (Szujecki 1980).

*fulvipes* **(Scopoli, 1763)**. Pileckis 1976a; Monsevičius 1986b, 1997; Dvilevičius et al. 1988; Silfverberg 1992, 2004; Gaidienė 1993; Pileckis and Monsevičius 1995; Smetana 2004g; Žiogas and Zolubas 2005; Dapkus and Tamutis 2008a; Alonso-Zarazaga 2009a.

*latebricola* **(Gravenhorst, 1806)**. Karalius and Monsevičius 1992; Pileckis and Monsevičius 1995; Silfverberg 1996, 2004; Monsevičius 1997; Smetana 2004g; Alonso-Zarazaga 2009a.

*stercorarius* **(Olivier, 1795)**. Mazurowa and Mazur 1939; Pileckis 1960, 1976a; Silfverberg 1992, 2004; Gaidienė 1993; Pileckis and Monsevičius 1995; Smetana 2004g; Alonso-Zarazaga 2009a.

*Staphylinus* **Linnaeus, 1758**.

*caesareus* **Cederhjelm, 1798**. Pileckis 1960, 1976a; Strazdienė 1988; Silfverberg 1992, 2004; Pileckis and Monsevičius 1995; Gliaudys 2001; Smetana 2004g; Ferenca 2006b; Alonso-Zarazaga 2009a.

*dimidiaticornis* **Gemminger, 1851** = *parumtomentosus* Stein, 1903.Pileckis 1976a; Silfverberg 1992, 2004; Gaidienė 1993; Pileckis and Monsevičius 1995; Tamutis 2002b; Tamutis et al. 2004, 2006, 2007; Smetana 2004g; Alonso-Zarazaga 2009a.

*erythropterus* **Linnaeus, 1758**. Eichwald 1830; Pileckis 1960, 1976a; Monsevičius 1986b, 1997; Dvilevičius et al. 1988; Silfverberg 1992, 2004; Gaidienė 1993; Pileckis and Monsevičius 1995; Šablevičius 2000b, 2011; Gliaudys 2001; Lynikienė 2003; Smetana 2004g; Vaivilavičius 2008; Alekseev 2008a; Ivinskis et al. 2008; Alonso-Zarazaga 2009a.

*Ocypus* **Leach, 1819**.

*aeneocephalus* **(DeGeer, 1774)**. Eichwald 1830; Silfverberg 1992, 2004; Pileckis and Monsevičius 1995; Smetana 2004g; Alonso-Zarazaga 2009a.

*brunnipes* **(Fabricius, 1781)**. Monsevičius 1985, 1986b, 1997; Silfverberg 1992, 2004; Pileckis and Monsevičius 1995; Smetana 2004g; Alonso-Zarazaga 2009a.

*fulvipennis* **Erichson, 1840**. Roubal 1910; Pileckis 1960, 1976a; Silfverberg 1992, 2004; Pileckis and Monsevičius 1995; Smetana 2004g; Alonso-Zarazaga 2009a.

*fuscatus* **(Gravenhorst, 1802)**. Eichwald 1830; Pileckis 1960, 1976a; Zajančkauskas and Pileckis 1968; Silfverberg 1992, 2004; Pileckis and Monsevičius 1995; Monsevičius 1997; Smetana 2004g; Ferenca 2006b; Dapkus and Tamutis 2007, 2008a; Alonso-Zarazaga 2009a.

*nitens* **(Schrank, 1781)** = *nero* (Faldermann, 1835) = *similis* (Fabricius, 1792) nec (Herbst, 1784). Eichwald 1830;Pileckis 1960, 1976a; Dvilevičius et al. 1988; Silfverberg 1992, 2004; Gaidienė 1993; Pileckis and Monsevičius 1995; Monsevičius 1997; Smetana 2004g; Ivinskis et al. 2008; Alonso-Zarazaga 2009a.

*olens* **(O.F. Müller, 1764)**. Tamutis et al. 2008.

*ophthalmicus* **(Scopoli, 1763)**. Pileckis 1960, 1976a; Silfverberg 1992, 2004; Pileckis and Monsevičius 1995; Ferenca et al. 2002; Alekseev 2003; Smetana 2004g; Alonso-Zarazaga 2009a; Ivinskis et al. 2009.

*picipennis* **(Fabricius, 1793)**. Mazurowa and Mazur 1939; Pileckis 1960, 1976a; Silfverberg 1992, 2004; Gaidienė 1993; Pileckis and Monsevičius 1995; Gliaudys 2001; Smetana 2004g; Alonso-Zarazaga 2009a.

*Tasgius* **Stephens, 1829** = *Alapsodus* Tottenham, 1939. (*Ocypus*)

*ater* **(Gravenhorst, 1802)**. Pileckis 1976a; Silfverberg 1992, 2004; Pileckis and Monsevičius 1995; Smetana 2004g; Ivinskis et al. 2008, 2009; Alonso-Zarazaga 2009a.

[*globulifer* **(Geoffroy, 1785)**].Known in Latvia (Telnov 2004), Denmark, Estonia, Sweden (Lundberg and Gustafsson 1995), Poland (Szujecki 1980).

*melanarius* **(Heer, 1839)**. Karalius and Monsevičius 1992; Gaidienė 1993; Silfverberg 1996, 2004; Monsevičius 1997; Ivinskis et al. 2009.

*morsitans* **(P. Rossi, 1790)** = *compressus* (Marsham, 1802) nec (Geoffroy, 1785).Łomnicki 1913; Silfverberg 1992, 2004; Gaidienė 1993; Pileckis and Monsevičius 1995.

*pedator* **(Gravenhorst, 1802)**. Ivinskis et al. 2009.

*winkleri* **(Bernhauer, 1906)**.Tamutis 2003.

*Creophilus* **Leach, 1819**.

*maxillosus* **(Linnaeus, 1758)**. Eichwald 1830; Pileckis 1960, 1976a; Lešinskas and Pileckis 1967; Silfverberg 1992, 2004; Gaidienė 1993; Pileckis and Monsevičius 1995; Monsevičius 1997; Gliaudys 2001; Smetana 2004g; Ferenca 2006b; Alonso-Zarazaga 2009a.

*Heterothops* **Stephens, 1829**.

[*binotatus* **(Gravenhorst, 1802)**]. Known in Denmark, Estonia, southern Sweden (Lundberg and Gustafsson 1995), Poland (Szujecki 1980).

*dissimilis* **(Gravenhorst, 1802)**. Bercio and Folwaczny 1979; Silfverberg 1992, 2004; Smetana 2004g; Alonso-Zarazaga 2009a.

[*minutus* **(Wollaston, 1860)** = *brunnipennis* auct. nec Kiesenwetter, 1858].Known in Denmark, Sweden (Lundberg and Gustafsson 1995).

*praevius niger* **Kraatz, 1868**. Pileckis 1976a; Silfverberg 1992, 2004; Pileckis and Monsevičius 1995; Monsevičius 1997, 1999; Smetana 2004g; Alonso-Zarazaga 2009a; Ivinskis et al. 2009.

*praevius praevius* **Erichson, 1839**. Monsevičius 1985; Silfverberg 1992, 2004; Smetana 2004g; Alonso-Zarazaga 2009a.

*quadripunctulus* **(Gravenhorst, 1806)**. Pileckis 1976a; Silfverberg 1992, 2004; Pileckis and Monsevičius 1995; Smetana 2004g; Alonso-Zarazaga 2009a.

*stiglundbergi* **Israelson, 1979**. Monsevičius 1985; Silfverberg 1992, 2004; Pileckis and Monsevičius 1995; Alonso-Zarazaga 2009a.

*Euryporus* **Erichson, 1839**.

*picipes* **(Paykull, 1800)**. Pileckis 1976a; Silfverberg 1992, 2004; Pileckis and Monsevičius 1995; Alonso-Zarazaga 2009a; Ivinskis et al. 2009.

*Velleius* **Leach, 1819**.

*dilatatus* **(Fabricius, 1787)**. Tamutis 2003; Ferenca 2003; Ivinskis et al. 2009.

*Quedius* **Stephens, 1829**.

[*auricomus* **Kiesenwetter, 1850**].Known in Denmark, Estonia (Lundberg and Gustafsson 1995).

*balticus* **Korge, 1860**.Silfverberg 1992, 2004; Smetana 2004g; Alonso-Zarazaga 2009a.

*boopoides* **Munster, 1923**.Monsevičius 1982, 1997; Silfverberg 1992, 2004; Pileckis and Monsevičius 1995; Smetana 2004g; Alonso-Zarazaga 2009a.

*boops* **(Gravenhorst, 1802)**.Monsevičius 1986b, 1997; Silfverberg 1992, 2004; Pileckis and Monsevičius 1995; Smetana 2004g; Alonso-Zarazaga 2009a.

*brevicornis* **(Thomson, 1860)**. Monsevičius 1985, 1987, 1997, 1999; Silfverberg 1992, 2004; Pileckis and Monsevičius 1995; Smetana 2004g; Alonso-Zarazaga 2009a.

*brevis* **Erichson, 1840**. Pileckis 1976a; Silfverberg 1992, 2004; Pileckis and Monsevičius 1995; Monsevičius 1997; Smetana 2004g; Alonso-Zarazaga 2009a.

*cinctus* **(Paykull, 1790)** = *flavescens* (Linnaeus, 1758).Pileckis 1976a; Silfverberg 1992, 2004; Pileckis and Monsevičius 1995; Smetana 2004g; Alonso-Zarazaga 2009a.

*cruentus* **(Olivier, 1795)**. Monsevičius and Jakaitis 1984; Silfverberg 1992, 2004; Gaidienė 1993; Pileckis and Monsevičius 1995; Smetana 2004g; Alonso-Zarazaga 2009a; Ivinskis et al. 2009.

*curtipennis* **Bernhauer, 1908**.Silfverberg 1992, 2004; Pileckis and Monsevičius 1995; Monsevičius 1997; Smetana 2004g; Alonso-Zarazaga 2009a.

*fulgidus* **(Fabricius, 1793)** = *assimilis* (Nordmann, 1837).Pileckis 1976a; Silfverberg 1992, 2004; Gaidienė 1993; Pileckis and Monsevičius 1995; Smetana 2004g; Alonso-Zarazaga 2009a.

*fuliginosus* **(Gravenhorst, 1802)**.Pileckis 1960, 1976a; Monsevičius 1986b, 1987, 1997; Dvilevičius et al. 1988; Silfverberg 1992, 2004; Gaidienė 1993; Pileckis and Monsevičius 1995; Gliaudys 2001; Tamutis and Zolubas 2001; Smetana 2004g; Ferenca 2006b; Vaivilavičius 2008; Alonso-Zarazaga 2009a.

*fulvicollis* **(Stephens, 1833)**.Pileckis 1976a; Silfverberg 1992, 2004; Pileckis and Monsevičius 1995; Smetana 2004g; Alonso-Zarazaga 2009a.

*fumatus* **(Stephens, 1833)**.Silfverberg 1992, 2004; Pileckis and Monsevičius 1995.

*humeralis* **Stephens, 1829** = *oblitteratus* Erichson, 1840.Pileckis 1976a; Silfverberg 1992, 2004; Pileckis and Monsevičius 1995; Smetana 2004g; Alonso-Zarazaga 2009a.

*infuscatus* **Erichson, 1840**. Ferenca et al. 2002; Ferenca 2006a.

[*invreae* **Gridelli, 1924**]. Known in Denmark, southern Sweden (Lundberg and Gustafsson 1995), Poland (Szujecki 1980).

[*lateralis* **(Gravenhorst, 1802)**]. Known in Latvia (Cibulskis 2010), Denmark, southern Sweden (Lundberg and Gustafsson 1995), Estonia (Silfverberg 2004), Poland (Szujecki 1980), Belarus (Alexandrovich et al. 1996).

*levicollis* **(Brullé, 1832)** = *tristis* (Gravenhorst, 1802) nec (Fabricius, 1793).Pileckis 1976a; Silfverberg 1992, 2004; Pileckis and Monsevičius 1995; Smetana 2004g; Alonso-Zarazaga 2009a.

*limbatus* **(Heer, 1834)** = *limbatoides* Coiffait, 1963.Monsevičius 1982, 1986b, 1987, 1997; Dvilevičius et al. 1988; Silfverberg 1992, 2004; Gaidienė 1993; Pileckis and Monsevičius 1995; Smetana 2004g; Alonso-Zarazaga 2009a.

*longicornis* **Kraatz, 1857**. Pileckis 1976a; Silfverberg 1992, 2004; Pileckis and Monsevičius 1995; Monsevičius 1997; Monsevičius 1999.

*lucidulus* **Erichson, 1839**.Silfverberg 1992, 2004; Gaidienė 1993; Pileckis and Monsevičius 1995; Smetana 2004g; Alonso-Zarazaga 2009a.

*maurorufus* **(Gravenhorst, 1806)**.Pileckis 1960, 1976a; Silfverberg 1992, 2004; Gaidienė 1993; Pileckis and Monsevičius 1995; Smetana 2004g; Ferenca 2006b; Alonso-Zarazaga 2009a.

*maurus* **(C.R. Sahlberg, 1830)**. Miländer et al. 1984; Monsevičius 1987, 1997; Silfverberg 1992, 2004; Pileckis and Monsevičius 1995; Smetana 2004g; Alonso-Zarazaga 2009a.

*mesomelinus* **(Marsham, 1802)**. Pileckis 1960, 1976a; Silfverberg 1992, 2004; Gaidienė 1993; Pileckis and Monsevičius 1995; Smetana 2004g; Ferenca 2006b; Alonso-Zarazaga 2009a.

*microps* **Gravenhorst, 1847.** Monsevičius 1985, 1987, 1997; Silfverberg 1992, 2004; Pileckis and Monsevičius 1995; Smetana 2004g; Alonso-Zarazaga 2009a.

*molochinus* **(Gravenhorst, 1806)** = *picipennis* (Paykull, 1800) nec (Fabricius, 1793).Mazurowa and Mazur 1939; Pileckis 1960, 1976a; Monsevičius 1986b, 1997; Silfverberg 1992, 2004; Gaidienė 1993; Pileckis and Monsevičius 1995; Smetana 2004g; Dapkus and Tamutis 2007, 2008a; Alonso-Zarazaga 2009a.

*nemoralis* **Baudi, 1848**.Bercio and Folwaczny 1979; Silfverberg 1992, 2004; Smetana 2004g; Alonso-Zarazaga 2009a.

*nigriceps* **Kraatz, 1857**.Gaidienė 1993; Tamutis 2003; Silfverberg 2004.

*nigrocaeruleus* **Fauvel, 1874**. Pileckis 1976a; Silfverberg 1992, 2004; Pileckis and Monsevičius 1995; Smetana 2004g; Alonso-Zarazaga 2009a.

*nitipennis* **(Stephens, 1833)** = *attenuatus* (Gyllenhal, 1808) nec (Gravenhorst, 1806). Pileckis 1976a (*Q. nitidipennis*); Monsevičius 1987; Dvilevičius et al. 1988; Silfverberg 1992, 2004; Gaidienė 1993; Pileckis and Monsevičius 1995; Smetana 2004g; Alonso-Zarazaga 2009a.

*ochripennis* **(Ménétriés, 1832)**. Gaidienė 1993; Silfverberg 2004.

[*paradisianus* **(Heer, 1839)**]. Known in Latvia (Telnov 2004), Poland (Szujecki 1980).

[*persimilis* **Mulsant & Rey, 1876** = *aridulus* Jansson, 1939].Known in Latvia (Cibulskis et al. 2005), Denmark, southern Sweden (Lundberg and Gustafsson 1995), Poland (Szujecki 1980).

*picipes* **(Mannerheim, 1830)**.Pileckis and Monsevičius 1995; Silfverberg 2004; Smetana 2004g.

*plagiatus* **Mannerheim, 1843** = *laevigatus* (Gyllenhal, 1810) nec (Marsham, 1802).Gaidienė 1993; Pileckis and Monsevičius 1995; Silfverberg 2004.

[*puncticollis* **(Thomson, 1867)**]. Known in Latvia (Cibulskis 2010), Denmark, southern Sweden (Lundberg and Gustafsson 1995), Poland (Szujecki 1980).

[*riparius* **Kellner, 1843**].Known in Latvia (Telnov 2004), Poland (Szujecki 1980).

*scintillans* **(Gravenhorst, 1806)**.Pileckis 1976a; Silfverberg 1992, 2004; Pileckis and Monsevičius 1995; Smetana 2004g; Alonso-Zarazaga 2009a.

*scitus* **(Gravenhorst, 1806)**. Silfverberg 1992, 2004; Gaidienė 1993; Pileckis and Monsevičius 1995; Monsevičius 1997; Smetana 2004g.

[*semiaeneus* **(Stephens, 1833)**]. Known in Denmark, southern Sweden (Lundberg and Gustafsson 1995), Poland (Szujecki 1980).

[*semiobscurus* **(Marsham, 1802)** = *rufipes* (Gravenhorst, 1802) nec (Linnaeus, 1758)].Known in Denmark, southern Sweden (Lundberg and Gustafsson 1995), Poland (Szujecki 1980).

*subunicolor* **Korge, 1861**.Silfverberg 1992, 2004; Alonso-Zarazaga 2009a.

*suturalis* **Kiesenwetter, 1845** = *humeralis* auct. nec (Stephens, 1832).Pileckis 1976a; Silfverberg 1992, 2004; Pileckis and Monsevičius 1995; Smetana 2004g; Alonso-Zarazaga 2009a.

*tenellus* **(Gravenhorst, 1806)**. Łomnicki 1913; Pileckis 1968b, 1976a; Silfverberg 1992, 2004; Pileckis and Monsevičius 1995; Smetana 2004g; Alonso-Zarazaga 2009a.

*truncicola* **Fairmaire & Laboulbéne, 1856** = *ventralis* (Aragona, 1830) nec (Gravenhorst, 1802).Gaidienė 1993; Silfverberg 2004.

*umbrinus* **Erichson, 1839** = *maritimus* (J.R. Sahlberg, 1876) = *pseudoumbrinus* Lohse, 1758.Silfverberg 1992, 2004; Gaidienė 1993; Monsevičius 1997; Monsevičius and Pankevičius 2001; Smetana 2004g; Alonso-Zarazaga 2009a.

*vexans* **Eppelsheim, 1881**. Monsevičius 1999.

*xanthopus* **Erichson, 1839**. Pileckis 1976a; Silfverberg 1992, 2004; Gaidienė 1993; Pileckis and Monsevičius 1995; Monsevičius 1997; Vaivilavičius 2008.

*Acylophorus* **Nordmann, 1837**.

[*glaberrimus* **(Herbst, 1784)**]. Known in Latvia (Telnov 2004), Denmark, Sweden (Lundberg and Gustafsson 1995), northwestern Belarus (Alexandrovich et al. 1996), Poland (Szujecki 1980).

*wagenschieberi* **Kiesenwetter, 1850**. Monsevičius 1982, 1988a, 1997; Ivinskis et al. 1984, 2009; Silfverberg 1992, 2004; Pileckis and Monsevičius 1995; Smetana 2004g; Alonso-Zarazaga 2009a.

*Atanygnathus* **Jakobson, 1909**.

*terminalis* **(Erichson, 1839)**. Ferenca and Tamutis 2009; Ivinskis et al. 2009.

**SCARABAEOIDEA Latreille, 1802**.

**GEOTRUPIDAE Latreille, 1806.** (Scarabaeidae)

**Bolboceratinae Mulsant, 1842**.

**Bolboceratini Mulsant, 1842**.

*Bolboceras* **Kirby, 1819** = *Odonteus* Samouelle, 1819.

*armiger* **(Scopoli, 1772)**. Pileckis 1960, 1976a; Silfverberg 1992, 2004; Gaidienė 1993; Pileckis and Monsevičius 1995; Ferenca 2004, 2006b; Löbl et al. 2006a; Ferenca et al. 2006, 2007; Ivinskis et al. 2009; Inokaitis 2009.

**Geotrupinae Latreille, 1806**.

**Lethrini Oken, 1843**

*Lethrus* **Scopoli, 1777**

*apterus **(Laxmann, 1770)**. Ferenca 2006b; disproved by Pileckis (1960).

**Chromogeotrupini Latreille, 1806**.

*Typhaeus* **Leach, 1815**.

[*typhoeus* **(Linnaeus, 1758)**]. Known in Kaliningrad region, Poland (López-Colón 2009), Denmark, southern Sweden (Lundberg and Gustafsson 1995).

**Geotrupini Latreille, 1802**.

*Geotrupes* **Latreille, 1796**.

*mutator* **(Marsham, 1802)**. Pileckis 1960, 1968c,1970b, 1976a; Silfverberg 1992, 2004; Gaidienė 1993; Pileckis and Monsevičius 1995; Ferenca 2006b; Löbl et al. 2006a; López-Colón 2009.

*spiniger* **Marsham, 1802** = *puncticollis* Malinowsky, 1811. Pileckis 1960, 1968c, 1970b, 1976a; Silfverberg 1992, 2004; Gaidienė 1993; Pileckis and Monsevičius 1995; Ferenca 2006b; Löbl et al. 2006a; López-Colón 2009.

*stercorarius* **(Linnaeus, 1758)**. Heyden 1903; Mazurowa and Mazur 1939; Pileckis 1960, 1976a; Lešinskas and Pileckis 1967; Zajančkauskas and Pileckis 1968; Silfverberg 1992, 2004; Gaidienė 1993; Pileckis and Monsevičius 1995; Monsevičius 1997; Šablevičius 2000b, 2011; Gliaudys 2001; Ferenca 2006b; Löbl et al. 2006a; López-Colón 2009.

*Anoplotrupes* **Jekel, 1865**. (*Geotrupes*)

*stercorosus* **(Scriba, 1791)**. Mazurowa and Mazur 1939; Pileckis 1960, 1976a; Zajančkauskas and Pileckis 1968; Ivinskis et al. 1984; Strazdienė 1981, 1988; Silfverberg 1992, 2004; Gaidienė 1993; Žiogas and Gedminas 1994; Pileckis and Monsevičius 1995; Monsevičius 1997; Šablevičius 2000b; Gliaudys 2001; Tamutis and Zolubas 2001; Lynikienė 2003; Ferenca 2006b; Löbl et al. 2006a; Dapkus and Tamutis 2007, 2008a; Ivinskis et al. 2008; López-Colón 2009.

*Trypocopris* **Motschulsky, 1858**. (*Geotrupes*)

*vernalis* **(Linnaeus, 1758)**. Mazurowa and Mazur 1939; Pileckis 1960, 1976a; Zajančkauskas and Pileckis 1968; Silfverberg 1992, 2004; Gaidienė 1993; Žiogas and Gedminas 1994; Pileckis and Monsevičius 1995; Monsevičius 1997; Šablevičius 2000b, 2011; Gliaudys 2001; Alekseev 2003; Ferenca 2006b; Löbl et al. 2006a; López-Colón 2009.

**TROGIDAE MacLeay, 1819**.

**Troginae MacLeay, 1819**.

*Trox* **Fabricius, 1775**.

*hispidus* **(Pontoppidan, 1763)**. Tamutis 2003; López-Colón 2009.

*sabulosus* **(Linnaeus, 1758)**. Eichwald 1830; Pileckis 1960, 1976a; Silfverberg 1992, 2004; Gaidienė 1993; Pileckis and Monsevičius 1995; Šablevičius 2003a, b; Ferenca 2006b; Pittino 2006; López-Colón 2009.

*scaber* **(Linnaeus, 1767)**. Pileckis 1960, 1976a; Silfverberg 1992, 2004; Pileckis and Monsevičius 1995; Pittino 2006; López-Colón 2009.

**LUCANIDAE Latreille, 1806**.

**Aesalinae** **MacLeay, 1819**.

**Aesalini MacLeay, 1819**.

*Aesalus* **Fabricius, 1801**.

[*scarabaeoides* **(Panzer, 1794)**]. Known in southern Sweden (Lundberg and Gustafsson 1995), Poland (Bartolozzi 2009).

**Ceruchitinae** **Nikolajev, 2006**.

*Ceruchus* **MacLeay, 1819**.

RDB*chrysomelinus* **(Hochenwarth, 1785)**. Lentz 1879; Pileckis 1970a, 1976a, b, 1979, 1982; Pileckis and Jakaitis 1982; Ivinskis et al. 1984, 2004, 2007; Balevičius 1992; Silfverberg 1992, 2004; Pileckis and Monsevičius 1995; Šablevičius 1995, 2003b, 2011; Šablevičius and Ferenca 1995; Monsevičius 1997; Ehnström et al. 2003; Ferenca 2004; Tamutis 2005b; Bartolozzi and Sprecher-Uebersax 2006; Rašomavičius 2007; Uselis et al. 2007; Vaivilavičius 2008; Dapkus and Tamutis 2008b; Bartolozzi 2009; Alekseev 2010b.

**Syndesinae** **MacLeay, 1819**.

*Sinodendron* **Schneider, 1791**.

*cylindricum* **(Linnaeus, 1758)**. Eichwald 1830; Pileckis 1960, 1976a; Lešinskas and Pileckis 1967; Gaidienė and Ferenca 1992; Silfverberg 1992, 2004; Gaidienė 1993; Pileckis and Monsevičius 1995; Monsevičius 1997; Šablevičius 2000b, 2011; Gliaudys 2001; Tamutis and Zolubas 2001; Ferenca 2006b; Bartolozzi and Sprecher-Uebersax 2006; Bartolozzi 2009.

**Lucaninae** **Latreille, 1804**.

**Platycerini Mulsant, 1842**

*Platycerus* **Geoffroy, 1726**.

*caprea* **(DeGeer, 1774)**. Pileckis 1976a; Silfverberg 1992, 2004; Pileckis and Monsevičius 1995; Ehnström et al. 2003; Bartolozzi and Sprecher-Uebersax 2006; Bartolozzi 2009; Šablevičius 2011.

*caraboides* **(Linnaeus, 1758)**. Heyden 1903; Pileckis 1960, 1976a; Zajančkauskas and Pileckis 1968; Pileckis et al. 1968; Gaidienė and Ferenca 1992; Silfverberg 1992, 2004; Gaidienė 1993; Pileckis and Monsevičius 1995; Monsevičius 1997; Šablevičius 2000b, 2011; Gliaudys 2001; Tamutis and Zolubas 2001; Ehnström et al. 2003; Žiogas and Zolubas 2005; Ferenca 2006a, b; Bartolozzi and Sprecher-Uebersax 2006; Bartolozzi 2009.

**Lucanini Latreille, 1804**.

*Dorcus* **MacLeay, 1819**.

*parallelepipedus* **(Linnaeus, 1758)**. Eichwald 1830; Mazurowa and Mazur 1939; Pileckis 1960, 1970b, 1976a, b, 1979; Lešinskas and Pileckis 1967; Gaidienė and Ferenca 1992; Silfverberg 1992, 2004; Gaidienė 1993; Pileckis and Monsevičius 1995; Monsevičius 1997; Šablevičius 2000b, 2011; Gliaudys 2001; Ferenca 2006b; Bartolozzi and Sprecher-Uebersax 2006; Bartolozzi 2009.

*Lucanus* **Scopoli, 1763**.

RDB*cervus* **Linnaeus, 1758**. Eichwald 1830; Heyden 1903; Pileckis 1960, 1970a, b, 1976a, b, 1979, 1982; Lešinskas and Pileckis 1967; Jezerskas et al. 1981; Balevičius 1992; Silfverberg 1992, 2004; Gaidienė 1993; Pileckis and Monsevičius 1995; Ehnström et al. 2003; Tamutis 2005b; Ferenca 2006b; Bartolozzi and Sprecher-Uebersax 2006; Rašomavičius 2007; Bartolozzi 2009; Alekseev 2010b; Šablevičius 2011.

**OCHODAEIDAE Mulsant & Rey, 1871**.

**Ochodaeinae Mulsant & Rey, 1871**.

**Ochodaeini Mulsant & Rey, 1871**.

*Ochodaeus* **Dejean, 1821**.

[*chrysomeloides* **(Schrank, 1825)**].Known in northwestern Belarus (Alexandrovitch et al. 1996), Poland (López-Colón 2009).

**SCARABAEIDAE Latreille, 1802**.

**Aegialiinae** **Laporte, 1840**.

*Aegialia* **Latreille, 1807**.

*arenaria* **(Fabricius, 1787)**. Pileckis 1963b, 1976a, b, 1979; Bercio and Folwaczny 1979; Silfverberg 1992, 2004; Pileckis and Monsevičius 1995; Skeiveris and Paplauskis 1998; Ferenca et al. 2002, 2006, 2007; Ferenca 2004; Ivinskis and Rimšaitė 2005; Stebnicka 2006; Ávilla 2009; Ivinskis et al. 2009.

*Rhysothorax* **Bedel, 1911**.

*rufus* **(Fabricius, 1792)** = *spissipes* LeConte, 1878 = *rufina* Silfverberg, 1977. Stebnicka 2006; Ávilla 2009.

*Psammoporus* **Thomson, 1863**.

*sabuleti* **(Panzer, 1797)**. Monsevičius 1988b; Silfverberg 1992, 2004; Pileckis and Monsevičius 1995; Stebnicka 2006; Ávilla 2009.

**Aphodiinae** **Leach, 1815**.

**Aphodiini Leach, 1815**.

*Calobopterus* **Mulsant, 1842**.

*erraticus* **(Linnaeus, 1758)**. Mazurowa and Mazur 1939; Zajančkauskas and Pileckis 1968; Pileckis 1960, 1976a; Silfverberg 1992, 2004; Gaidienė 1993; Pileckis and Monsevičius 1995 (*Aphodius*); Monsevičius 1997; Šablevičius 2000b, 2004; Ferenca 2006b; Stebnicka et al. 2006; Alonso-Zarazaga 2009a.

*Euplerus* **Mulsant, 1842**.

*subterraneus* **(Linnaeus, 1758)**. Mazurowa and Mazur 1939; Pileckis 1960, 1976a; Bercio and Folwaczny 1979; Silfverberg 1992, 2004; Gaidienė 1993; Pileckis and Monsevičius 1995 (*Aphodius*); Monsevičius 1997; Ferenca 2006b; Stebnicka et al. 2006; Alonso-Zarazaga 2009a.

*Teuchestes* **Mulsant, 1842**.

*fossor* **(Linnaeus, 1758)**. Eichwald 1830; Mazurowa and Mazur 1939; Pileckis 1960, 1963b, 1976a; Zajančkauskas and Pileckis 1968; Silfverberg 1992, 2004; Gaidienė 1993; Pileckis and Monsevičius 1995 (*Aphodius*); Monsevičius 1997; Gliaudys 2001; Šablevičius 2000b, 2004, 2011; Ferenca 2006b; Stebnicka et al. 2006; Alonso-Zarazaga 2009a.

*Oromus* **Mulsant & Rey, 1869**.

*alpinus **(Scopoli, 1763)**. Mazurowa and Mazur 1939 (*Aphodius*); disproved by Pileckis (1960).

*Otophorus* **Mulsant, 1842**.

*haemorrhoidalis* **(Linnaeus, 1758)**. Mazurowa and Mazur 1939; Pileckis 1960, 1976a; Silfverberg 1992, 2004; Gaidienė 1993; Pileckis and Monsevičius 1995 (*Aphodius*); Monsevičius 1997; Šablevičius 2000b, 2004; Stebnicka et al. 2006; Alonso-Zarazaga 2009a.

*Ammoecius* **Mulsant, 1842**.

[*brevis* **Erichson, 1848**]. Known in Latvia (Telnov 2004), Estonia, Denmark, throughout Sweden (Lundberg and Gustafsson 1995), Poland (Stebnicka 1976).

*Plagiogonus* **Mulsant, 1842**.

*putridus* **(Geoffroy, 1785)** = *arenarius* (Olivier, 1789) nec (Fabricius, 1787) = *rhododactylus* (Marsham, 1802). Pileckis 1960, 1968c 1976a; Silfverberg 1992, 2004; Pileckis and Monsevičius 1995(*Aphodius*); Stebnicka et al. 2006; Alonso-Zarazaga 2009a.

*Acrossus* **Mulsant, 1842**.

[*bimaculatus* **(Laxman, 1770)**]. Known in Latvia (Telnov 2004), Belarus (Alexandrovitch et al. 1996), Kaliningrad region (Bercio and Folwaczny 1979), Estonia (Lundberg and Gustafsson 1995), Poland (Stebnicka 1976). 

*depressus* **(Kugelann, 1792)**. Mazurowa and Mazur 1939; Pileckis 1960, 1963, 1976a; Silfverberg 1992, 2004; Gaidienė 1993; Pileckis and Monsevičius 1995 (*Aphodius*); Monsevičius 1997; Stebnicka et al. 2006; Alonso-Zarazaga 2009a.

*luridus* **(Fabricius, 1775)**. Pileckis 1960, 1976a; Zajančkauskas and Pileckis 1968; Silfverberg 1992, 2004; Gaidienė 1993; Pileckis and Monsevičius 1995 (*Aphodius*); Monsevičius 1997; Ferenca 2006b; Stebnicka et al. 2006; Alonso-Zarazaga 2009a.

*rufipes* **(Linnaeus, 1758)**. Heyden 1903; Mazurowa and Mazur 1939; Pileckis 1960, 1976a; Zajančkauskas and Pileckis 1968; Silfverberg 1992, 2004; Gaidienė 1993; Pileckis and Monsevičius 1995 (*Aphodius*); Monsevičius 1997; Šablevičius 2000b; Ferenca 2006b; Stebnicka et al. 2006; Alonso-Zarazaga 2009a.

*Limarus* **Mulsant & Rey, 1870**.

[*zenkeri* **(Germar, 1813)**]. Known in in Denmark, southern Sweden (Lundberg and Gustafsson 1995), northern Poland (Stebnicka 1976).

*Esymus* **Mulsant & Rey, 1870**.

*pusillus* **(Herbst, 1789)**. Mazurowa and Mazur 1939; Pileckis 1960, 1976a; Zajančkauskas and Pileckis 1968; Bercio and Folwaczny 1979; Silfverberg 1992, 2004; Gaidienė 1993; Pileckis and Monsevičius 1995 (*Aphodius*); Monsevičius 1997; Ferenca 2006b; Stebnicka et al. 2006; Alonso-Zarazaga 2009a.

*Euorodalus* **G. Dellacasa, 1983**.

*coenosus* **(Panzer, 1798)** = *tristis* (Zenker, 1801). Pileckis 1960, 1976a; Silfverberg 1992, 2004; Gaidienė 1993; Pileckis and Monsevičius 1995 (*Aphodius*); Ferenca 2006b; Stebnicka et al. 2006; Alonso-Zarazaga 2009a.

[*paracoenosus* **(Balthazar & Hrubant, 1960)**].Known in western Belarus (Alexandrovitch et al. 1996), throughout Poland (Stebnicka 1976).

*Eudolus* **Mulsant & Rey, 1870**.

*quadriguttatus* **(Herbst, 1783)**. Mazurowa and Mazur 1939; Pileckis 1960, 1968c, 1976a; Silfverberg 1992, 2004; Pileckis and Monsevičius 1995 (*Aphodius*); Stebnicka et al. 2006; Alonso-Zarazaga 2009a.

*Phalacronothus* **Motschulsky, 1859**.

[*quadrimaculatus* **(Linnaeus, 1761)**]. Known in Estonia (Süda 2009), Poland (Stebnicka 1976).

*Nimbus* **Mulsant & Rey, 1870**.

*contaminatus* **(Herbst, 1783)**. Pileckis 1960, 1976a; Silfverberg 1992, 2004; Pileckis and Monsevičius 1995 (*Aphodius*); Ferenca 2006b; Stebnicka et al. 2006; Alonso-Zarazaga 2009a.

*Volinus* **Mulsant & Rey, 1870**.

*equestris* **(Panzer, 1798)** = *sticticus* (Panzer, 1798) nec (Linnaeus, 1767). Pileckis 1960, 1976; Silfverberg 1992, 2004; Gaidienė 1993; Pileckis and Monsevičius 1995 (*Aphodius*); Ferenca 2006b; Stebnicka et al. 2006; Alonso-Zarazaga 2009a.

*Chilothorax* **Motschulsky, 1859**.

*conspurcatus* **(Linnaeus, 1758)**. Pileckis 1960, 1976a; Silfverberg 1992, 2004; Pileckis and Monsevičius 1995 (*Aphodius*); Ferenca 2006b; Stebnicka et al. 2006; Alonso-Zarazaga 2009a.

*distinctus* **(O.F. Müller, 1776)** = *inquinatus* (Herbst, 1783). Pileckis 1960, 1976a; Silfverberg 1992, 2004; Gaidienė 1993; Pileckis and Monsevičius 1995 (*Aphodius*); Monsevičius 1997; Gliaudys 2001; Ferenca 2006b; Stebnicka et al. 2006; Alonso-Zarazaga 2009a; Šablevičius 2011.

*melanostictus* **(W. Schmidt, 1840)**. Mazurowa and Mazur 1939; Pileckis 1960, 1976a; Silfverberg 1992, 2004; Gaidienė 1993; Pileckis and Monsevičius 1995 (*Aphodius*); Stebnicka et al. 2006; Alonso-Zarazaga 2009a.

[*paykulli **(Bedel, 1908)** = *tessulatus* (Paykull, 1798) nec (Moll, 1782)]. # 53. Gaidienė 1993; Silfverberg 2004.

[*pictus* **(Sturm, 1805)**].Known in Latvia (Telnov 2004), Denmark, southern Sweden (Lundberg and Gustafsson 1995), Poland (Stebnicka 1976).

*Melinopterus* **Mulsant, 1842**.

*prodromus* **(Brahm, 1790)**. Pileckis 1960, 1976a; Zajančkauskas and Pileckis 1968; Silfverberg 1992, 2004; Gaidienė 1993; Pileckis and Monsevičius 1995 (*Aphodius*); Monsevičius 1997; Tamutis and Zolubas 2001; Žiogas and Zolubas 2005; Ferenca 2006b; Stebnicka et al. 2006; Alonso-Zarazaga 2009a.

[*punctatosulcatus* **(Sturm, 1805)** = *sabulicola* Thomson, 1868]. Known in Latvia (Telnov 2004), western Belarus (Alexandrovitch et al. 1996), Estonia, throughout Sweden (Lundberg and Gustafsson 1995), Poland (Stebnicka 1976).

*sphacelatus* **(Panzer, 1798)**. Pileckis 1960, 1976a; Zajančkauskas and Pileckis 1968; Silfverberg 1992, 2004; Gaidienė 1993; Pileckis and Monsevičius 1995 (*Aphodius*); Monsevičius 1997; Ferenca 2006b; Stebnicka et al. 2006; Alonso-Zarazaga 2009a.

*Pubinus* **Mulsant & Rey, 1870**.

*tomentosus* **(O.F. Müller, 1776)**. Pileckis and Monsevičius 1995 (*Aphodius*); Stebnicka et al. 2006.

*Sigorus* **Mulsant & Rey, 1870**.

*porcus* **(Fabricius, 1792)**. Pileckis and Monsevičius 1995 (*Aphodius*); Silfverberg 2004; Stebnicka et al. 2006.

*Trichonotulus* **Bedel, 1911**.

*scrofa* **(Fabricius, 1787)**. Pileckis 1976a; Silfverberg 1992, 2004; Pileckis and Monsevičius 1995 (*Aphodius*); Stebnicka et al. 2006; Alonso-Zarazaga 2009a.

*Esymus* **Mulsant & Rey, 1870**.

*merdarius* **(Fabricius, 1775)**. Mazurowa and Mazur 1939; Pileckis 1960, 1976a; Zajančkauskas and Pileckis 1968; Silfverberg 1992, 2004; Gaidienė 1993; Pileckis and Monsevičius 1995 (*Aphodius*); Monsevičius 1997; Ferenca 2006b; Stebnicka et al. 2006; Alonso-Zarazaga 2009a.

*Aphodius* **Illiger, 1798**.

*fimetarius* **(Linnaeus, 1758)**. Eichwald 1830; Roubal 1910; Mazurowa and Mazur 1939; Pileckis 1960, 1976a; Zajančkauskas and Pileckis 1968; Strazdienė 1976; Silfverberg 1992, 2004; Gaidienė 1993; Pileckis and Monsevičius 1995; Monsevičius 1997; Šablevičius 2000b, 2011; Gliaudys 2001; Tamutis and Zolubas 2001; Ferenca 2006b; Stebnicka et al. 2006; Alonso-Zarazaga 2009a.

*foetens* **(Fabricius, 1787)** = *aestivalis* Stephens, 1839. Pileckis 1960, 1976a; Bercio and Folwaczny 1979; Silfverberg 1992, 2004; Gaidienė 1993; Pileckis and Monsevičius 1995; Monsevičius 1997; Tamutis and Zolubas 2001; Ferenca 2006b; Stebnicka et al. 2006; Alonso-Zarazaga 2009a.

*foetidus* **(Herbst, 1783)** = *scybalarius* auct. nec (Fabricius, 1781). Pileckis 1976a; Silfverberg 1992, 2004; Pileckis and Monsevičius 1995; Stebnicka et al. 2006; Alonso-Zarazaga 2009a.

*Planolinus* **Mulsant & Rey, 1870**.

*borealis* **(Gyllenhal, 1827)**. Silfverberg 1992, 2004; Gaidienė 1993; Pileckis and Monsevičius 1995 (*Aphodius*); Stebnicka et al. 2006; Alonso-Zarazaga 2009a.

*uliginosus* **(Hardy, 1847)** = *putridus* (Herbst, 1789) nec (Geoffroy, 1785) = *fasciatus* (Olivier, 1789) nec (Linnaeus, 1758) = *tenellus* auct. nec (Say, 1823). Pileckis 1976a; Jakaitis 1985; Silfverberg 1992, 2004; Gaidienė 1993; Pileckis and Monsevičius 1995 (*Aphodius*); Stebnicka et al. 2006; Alonso-Zarazaga 2009a.

*Agrilinus* **Mulsant & Rey, 1870**.

*ater* **(DeGeer, 1774)**. Eichwald 1830; Pileckis 1960, 1976a; Silfverberg 1992, 2004; Gaidienė 1993; Pileckis and Monsevičius 1995 (*Aphodius*); Monsevičius 1997; Stebnicka et al. 2006; Alonso-Zarazaga 2009a.

*rufus* **(Moll, 1782)** = *scybalarius* (Fabricius, 1781). Roubal 1910; Tenenbaum 1923, 1931; Mazurowa and Mazur 1939; Pileckis 1960, 1968b, 1976a; Strazdienė 1976; Silfverberg 1992, 2004; Pileckis and Monsevičius 1995 (*Aphodius*); Monsevičius 1997; Stebnicka et al. 2006; Alonso-Zarazaga 2009a; Ivinskis et al. 2009.

*sordidus* **(Fabricius, 1775)**. Eichwald 1830; Mazurowa and Mazur 1939; Pileckis 1960, 1976a; Silfverberg 1992, 2004; Gaidienė 1993; Pileckis and Monsevičius 1995 (*Aphodius*); Monsevičius 1997; Ferenca 2006b; Stebnicka et al. 2006; Alonso-Zarazaga 2009a; Ivinskis et al. 2009.

*Agoliinus* **A. Schmidt, 1913**.

*nemoralis* **(Erichson, 1848)** = *ardoei* (Landin, 1954). Pileckis 1976a; Silfverberg 1992, 2004; Gaidienė 1993; Pileckis and Monsevičius 1995 (*Aphodius*); Stebnicka et al. 2006; Alonso-Zarazaga 2009a.

*piceus* **(Gyllenhal, 1808)**. Jakaitis 1985; Silfverberg 1992, 2004; Pileckis and Monsevičius 1995 (*Aphodius*); Monsevičius 1997; Stebnicka et al. 2006; Alonso-Zarazaga 2009a.

*Acanthobodilus* **G. Dellacasa, 1983**.

*immundus* **(Creutzer, 1799)**. Mazurowa and Mazur 1939; Pileckis 1960, 1968c, 1976a; Silfverberg 1992, 2004; Pileckis and Monsevičius 1995 (*Aphodius*); Stebnicka et al. 2006; Alonso-Zarazaga 2009a.

*Bodilus* **Mulsant & Rey, 1870**.

*ictericus* **(Laicharting, 1781)** = *nitidulus* (Fabricius, 1792). Mazurowa and Mazur 1939; Pileckis 1960, 1976a; Silfverberg 1992, 2004; Pileckis and Monsevičius 1995 (*Aphodius*); Monsevičius 1997; Stebnicka et al. 2006; Alonso-Zarazaga 2009a.

[*lugens* **(Creutzer, 1799)**]. Known in Latvia (Telnov 2004), Belarus (Alexandrovitch et al. 1996), Estonia (Silfverberg 2004), Poland (Stebnicka 1976).

*punctipennis **(Erichson, 1848)**. # 54. Gaidienė 1993 (*Aphodius*); Silfverberg 2004.

*Nialus* **Mulsant & Rey, 1870**.

*varians* **Duftschmid, 1805** = *bimaculatus* (Fabricius, 1787) nec (Laxmann, 1770) = *niger* (Panzer, 1797). Pileckis 1960, 1976a; Bercio and Folwaczny 1979; Silfverberg 1992, 2004; Gaidienė 1993; Pileckis and Monsevičius 1995 (*Aphodius*); Ferenca 2006b; Stebnicka et al. 2006; Alonso-Zarazaga 2009a.

*Liothorax* **Motschulsky, 1859**.

*plagiatus* **(Linnaeus, 1767)**. Pileckis 1960, 1976a; Bercio and Folwaczny 1979; Silfverberg 1992, 2004; Gaidienė 1993; Pileckis and Monsevičius 1995 (*Aphodius*); Ferenca 2006b; Stebnicka et al. 2006; Alonso-Zarazaga 2009a.

*Labarrus* **Mulsant & Rey, 1870**.

*lividus* **(Olivier, 1789)**. Pileckis 1976a; Silfverberg 1992, 2004; Pileckis and Monsevičius 1995 (*Aphodius*); Stebnicka et al. 2006; Alonso-Zarazaga 2009a.

*Calamosternus* **Motschulsky, 1859**.

*granarius* **(Linnaeus, 1767)**. Heyden 1903; Roubal 1910; Mazurowa and Mazur 1939; Pileckis 1960, 1976a; Silfverberg 1992, 2004; Gaidienė 1993; Pileckis and Monsevičius 1995 (*Aphodius*); Stebnicka et al. 2006; Alonso-Zarazaga 2009a.

*Euheptaulacus* **G. Dellacasa, 1983**.

*sus* **(Herbst, 1783)**. Gaidienė and Ferenca 1988; Silfverberg 1992, 2004; Gaidienė 1993; Pileckis and Monsevičius 1995 (*Heptaulacus*); Stebnicka et al. 2006; Alonso-Zarazaga 2009a.

[*villosus* **(Gyllenhal, 1806)**].Known in Latvia (Telnov 2004), Denmark, Estonia, southern Sweden (Lundberg and Gustafsson 1995), northern Poland (Stebnicka 1976).

*Heptaulacus* **Mulsant, 1842**.

*testudinarius* **(Fabricius, 1775)**. Pileckis 1976a; Silfverberg 1992, 2004; Gaidienė 1993; Pileckis and Monsevičius 1995; Stebnicka et al. 2006; Alonso-Zarazaga 2009a.

*Oxyomus* **Dejean, 1833**.

*sylvestris* **(Scopoli, 1763)**. Pileckis 1960, 1976a; Silfverberg 1992, 2004; Gaidienė 1993; Pileckis and Monsevičius 1995; Monsevičius 1997; Inokaitis 2004; Ferenca 2006b; Stebnicka et al. 2006; Alonso-Zarazaga 2009a.

**Psammodiini Mulsant, 1842**.

*Psammodius* **Fallén, 1807**.

*asper* **(Fabricius, 1775)** = *sulcicollis*(Illiger, 1802). Pileckis 1960, 1976a; Bercio and Folwaczny 1979; Silfverberg 1992, 2004; Gaidienė 1993; Pileckis and Monsevičius 1995; Ferenca 2004, 2006b; Stebnicka et al. 2006; Alekseev 2008a; Ivinskis et al. 2009.

*Diastictus* **Mulsant, 1842**.

*vulneratus* **(Sturm, 1805)**. Pileckis 1976a; Silfverberg 1992, 2004; Pileckis and Monsevičius 1995; Stebnicka et al. 2006.

*Pleurophorus* **Mulsant, 1842**.

[*caesus* **(Creutzer, 1796)**]. Known in Latvia ( Telnov 2004), southern Sweden (Lundberg and Gustafsson 1995), Kaliningrad region (Bercio and Folwaczny 1979), Poland (Stebnicka 1976).

*Rhyssemus* **Mulsant, 1842**.

*germanus* **(Linnaeus, 1767)**.Mazurowa and Mazur 1939; Pileckis 1960, 1976a; Silfverberg 1992, 2004; Pileckis and Monsevičius 1995; Stebnicka et al. 2006.

**Scarabaeinae** **Latreille, 1802**.

**Coprini Leach, 1815**.

*Copris* **Geoffroy, 1762**.

*lunaris* **(Linnaeus, 1758)**. Eichwald 1830; Mazurowa and Mazur 1939; Pileckis 1960, 1970b, 1976a; Lešinskas and Pileckis 1967; Silfverberg 1992, 2004; Gaidienė 1993; Pileckis and Monsevičius 1995; Monsevičius 1997; Šablevičius 2000b, 2011; Gliaudys 2001; Ferenca 2006b; Löbl et al. 2006a; Ferenca et al. 2006, 2007.

**Oniticellini Kolbe, 1905**.

*Euoniticellus* **Janssens, 1953**.

*fulvus* **(Goeze, 1777)**. Pileckis 1960, 1968c, 1976a; Silfverberg 1992, 2004; Gaidienė 1993; Pileckis and Monsevičius 1995 (*Oniticellus*); Gliaudys 2001; Ferenca 2006b.

**Onthophagini Burmeister, 1846**.

*Caccobius* **Thomson, 1859**.

*schreberi* **(Linnaeus, 1767)**. Mazurowa and Mazur 1939; Pileckis 1960, 1963b, 1976a; Silfverberg 1992, 2004; Gaidienė 1993; Pileckis and Monsevičius 1995; Gliaudys 2001; Ferenca 2006b; Löbl et al. 2006c.

*Onthophagus* **Latreille, 1802**.

*coenobita* **(Herbst, 1787)**. Eichwald 1830; Pileckis 1960, 1976a; Silfverberg 1992, 2004; Gaidienė 1993; Pileckis and Monsevičius 1995; Šablevičius 2004; Löbl et al. 2006c; Alonso-Zarazaga 2009a.

*fracticornis* **(Preyssler, 1790)**. Mazurowa and Mazur 1939; Pileckis 1960, 1976a; Bercio and Folwaczny 1979; Silfverberg 1992, 2004; Gaidienė 1993; Pileckis and Monsevičius 1995; Monsevičius 1997; Šablevičius 2000b; Gliaudys 2001; Löbl et al. 2006c.

*gibbulus* **(Pallas, 1781)** = *austriacus* (Panzer, 1793). Mazurowa and Mazur 1939; Pileckis 1960, 1976a; Bercio and Folwaczny 1979; Silfverberg 1992, 2004; Pileckis and Monsevičius 1995; Šablevičius 2000b; Löbl et al. 2006c.

[*illyricus* **(Scopoli, 1763)**].Known in southern Sweden (Lundberg and Gustafsson 1995), Belarus (Alexandrovitch et al. 1996), Poland (Stebnicka 1976).

*joannae* **Goljan, 1953**. Silfverberg 1992, 2004; Pileckis and Monsevičius 1995; Alonso-Zarazaga 2009a.

*nuchicornis* **(Linnaeus, 1758)**. Eichwald 1830; Mazurowa and Mazur 1939; Pileckis 1960, 1976a; Bercio and Folwaczny 1979; Silfverberg 1992, 2004; Gaidienė 1993; Pileckis and Monsevičius 1995; Šablevičius 2000b; Gliaudys 2001; Ferenca 2006b; Löbl et al. 2006c; Alonso-Zarazaga 2009a.

*ovatus* **(Linnaeus, 1767)**. Silfverberg 1992, 2004; Pileckis and Monsevičius 1995; Alonso-Zarazaga 2009a.

*similis* **(Scriba, 1790)**. Monsevičius 1988b, 1997; Silfverberg 1992, 2004; Pileckis and Monsevičius 1995; Löbl et al. 2006c; Alonso-Zarazaga 2009a.

*taurus* **(Schreber, 1759)**. Pileckis 1960, 1976a; Silfverberg 1992, 2004; Gaidienė 1993; Pileckis and Monsevičius 1995; Ivinskis et al. 1997a; Ferenca et al. 2002; Ferenca 2006b; Löbl et al. 2006c.

*vacca* **(Linnaeus, 1767)**. Eichwald 1830; Pileckis 1960, 1976a; Silfverberg 1992, 2004; Gaidienė 1993; Pileckis and Monsevičius 1995; Šablevičius 2000b; Ferenca 2006b; Löbl et al. 2006c; Alonso-Zarazaga 2009a.

**Melolonthinae Leach, 1819**.

**Sericini Kirby, 1837**.

*Serica* **MacLeay, 1819**.

*brunnea* **(Linnaeus, 1758)**. Heyden 1903; Roubal 1910; Mazurowa and Mazur 1939; Pileckis 1960, 1976a; Lešinskas and Pileckis 1967; Silfverberg 1992, 2004; Gaidienė 1993; Pileckis and Monsevičius 1995; Monsevičius 1997; Žiogas 1997; Šablevičius 2000b; Gliaudys 2001; Ferenca 2006b; Ahrens 2006; Ostrauskas and Ferenca 2010.

*Maladera* **Mulsant, 1871**.

*holosericea* **(Scopoli, 1772)**. Eichwald 1830; Pileckis 1960, 1963b, 1968c,1976a; Silfverberg 1992, 2004; Gaidienė 1993; Pileckis and Monsevičius 1995; Gliaudys 2001; Žiogas and Zolubas 2005; Ferenca 2006b; Ahrens 2006; Inokaitis 2009.

*Omaloplia* **Schönherr, 1817**.

*ruricola* **(Fabricius, 1775)** = *nigromarginata*(Herbst, 1785). Medvdev 1952; Pileckis 1960, 1963b, 1976a; Bercio and Folwaczny 1979; Silfverberg 1992, 2004; Gaidienė 1993; Pileckis and Monsevičius 1995; Monsevičius 1997; Gliaudys 2001; Ferenca 2006b; Ahrens 2006.

**Melolonthini Leach, 1819**.

*Amphimallon* **Berthold, 1827**.

[*assimile* **(Herbst, 1970)**].Known in northwestern Belarus (Alexandrovitch et al. 1996), Poland (Stebnicka 1978).

[*ochraceum* **(Knoch, 1801)**].Known in Denmark, southern Sweden (Lundberg and Gustafsson 1995), Poland (Stebnicka 1978).

[*ruficorne* **(Fabricius, 1775)**]. Known in in western Belarus (Alexandrovitch et al. 1996), Poland (Stebnicka 1978).

*solstitiale* **(Linnaeus, 1758)**. Eichwald 1830; Ivanauskas and Vailionis 1922; Mastauskis 1936; Mazurowa and Mazur 1939; Pileckis 1960, 1976a; Valenta 1965a, 2000a; Lešinskas and Pileckis 1967; Pileckis et al. 1968; Zajančkauskas and Pileckis 1968; Pileckis and Vengeliauskaitė 1977, 1996; Mensonienė 1981; Pileckis et al. 1983; Silfverberg 1992, 2004; Gaidienė 1993; Pileckis and Monsevičius 1995; Monsevičius 1997; Žiogas 1997; Šablevičius 2000b; Gliaudys 2001; Ferenca 2006b; Smetana and Král 2006; Alekseev 2008a.

*Melolontha* **Fabricius, 1775**.

*hippocastani* **Fabricius, 1801**. Ogijewicz 1929, 1931, 1932, 1938; Pileckis 1960, 1963a, 1976a; Valenta 1965a, 2000a; Lešinskas and Pileckis 1967; Pileckis et al. 1968; Ivinskis et al. 1984; Gavelis and Žiogas 1991; Silfverberg 1992, 2004; Gaidienė 1993; Pileckis and Monsevičius 1995; Žiogas 1997; Šablevičius 2000b; Gliaudys 2001; Ferenca 2006b; Bezdek 2006.

*melolontha* **(Linnaeus, 1758)**. Eichwald 1830; Heyden 1903; Ivanauskas and Vailionis 1922; Mastauskis 1926, 1936; Ogijewicz 1929, 1931, 1932, 1938; Mazurowa and Mazur 1939; Pileckis 1960, 1963a, 1976a; Valenta 1965a, 2000a; Lešinskas and Pileckis 1967; Zajančkauskas and Pileckis 1968; Pileckis et al. 1968, 1983; Strazdienė 1976; Pileckis and Vengeliauskaitė 1977, 1996; Mensonienė 1981; Ivinskis et al. 1984; Silfverberg 1992, 2004; Gaidienė 1993; Pileckis and Monsevičius 1995; Monsevičius 1997; Žiogas 1997; Šablevičius 2000b; Gliaudys 2001; Tamutis and Zolubas 2001; Ferenca 2006b; Bezdek 2006.

*Polyphylla* **Harris, 1842**.

RDB*fullo* **(Linnaeus, 1758)**. Eichwald 1830; Ivanauskas and Vailionis 1922; Pileckis 1960, 1968c, 1970b, 1976a, 1982; Lešinskas and Pileckis 1967; Pileckis et al. 1968; Bercio and Folwaczny 1979; Strazdienė 1988; Balevičius 1992; Silfverberg 1992, 2004; Gaidienė 1993; Pileckis and Monsevičius 1995; Ostrauskas 1995; Ivinskis et al. 1996b, 2004a, 2009; Monsevičius 1997, 1998; Skeiveris and Paplauskis 1998; Žiogas 1997; Gliaudys 2001; Alekseev 2003, 2010b; Dapkus 2004; Žiogas and Zolubas 2005; Tamutis 2005b; Ivinskis and Rimšaitė 2005; Ferenca 2006a, b; Bezdek 2006; Rašomavičius 2007, Bačianskas 2009; Šablevičius 2011.

**Hopliini Latreille, 1829**.

*Hoplia* **Illiger, 1803**.

*graminicola* **(Fabricius, 1792)**. Pileckis 1960, 1976a; Silfverberg 1992, 2004; Gaidienė 1993; Pileckis and Monsevičius 1995; Šablevičius 2000b; Gliaudys 2001; Ferenca 2006b; Smetana 2006a; Alonso-Zarazaga 2009a.

*parvula* **Krynicki, 1832**. Pileckis 1959, 1960, 1976a; Bercio and Folwaczny 1979; Silfverberg 1992, 2004; Gaidienė 1993; Pileckis and Monsevičius 1995; Gliaudys 2001; Ferenca 2006b; Smetana 2006a; Alonso-Zarazaga 2009a; Šablevičius 2011.

*philanthus* **(Fuesslin, 1775)** = *farinosa*(Linnaeus, 1761). Pileckis and Monsevičius 1995.

[*praticola* **Duftschmid, 1805**].Known in Latvia (Telnov 2004), Poland (Stebnicka 1978). 

[*subnuda* **Reitter, 1903**].Known in western Belarus (Alexandrovitch et al. 1996), Poland (Stebnicka 1978).

[*zaitzevi* **Jakobson, 1914**].Known in Latvia (Telnov 2004), western Belarus (Alexandrovitch et al. 1996), Estonia (Lundberg and Gustafsson 1995).

**Rutelinae** **MacLeay, 1819**.

**Anomalini Streubel, 1839**.

*Anomala* **Leach, 1819**.

*dubia* **(Scopoli, 1763)**. Eichwald 1830; Heyden 1903; Mazurowa and Mazur 1939; Pileckis 1960, 1976a; Lešinskas and Pileckis 1967; Pileckis et al. 1968; Pileckis and Vengeliauskaitė 1977, 1996; Ivinskis et al. 1984; Strazdienė 1988; Silfverberg 1992, 2004; Gaidienė 1993; Pileckis et al. 1994a; Pileckis and Monsevičius 1995; Monsevičius 1997; Žiogas 1997; Monsevičius 1998; Šablevičius 2000b; Gliaudys 2001; Ferenca 2006b; Zorn 2006; Alonso-Zarazaga 2009a; Vaivilavičius 2008; as *A. aenea* Deg. in: Ogijewicz 1929, 1931.

*Phyllopertha* **Stephens, 1830**.

*horticola* **(Linnaeus, 1758)**. Eichwald 1830; Heyden 1903; Ogijewicz 1929, 1931, 1932, 1938; Mazurowa and Mazur 1939; Pileckis 1960, 1976a; Lešinskas and Pileckis 1967; Zajančkauskas and Pileckis 1968; Pileckis et al. 1968; Pileckis and Vengeliauskaitė 1977, 1996; Mensonienė 1981; Pileckis et al. 1983; 1994a; Silfverberg 1992, 2004; Gaidienė 1993; Pileckis and Monsevičius 1995; Monsevičius 1997; Žiogas 1997; Šablevičius 2000b; Gliaudys 2001; Tamutis and Zolubas 2001; Ferenca 2006b; Zorn 2006; Alonso-Zarazaga 2009a; Vaivilavičius 2008.

*Chaetopteroplia* **Medvedev, 1949**.

*segetum* **(Herbst, 1783)**. Pileckis and Monsevičius 1982, 1995; Silfverberg 1992, 2004; Alonso-Zarazaga 2009a.

*Anisoplia* **Schönherr, 1817**.

*agricola* **(Poda, 1761)**. Pileckis 1968a, c, 1970b, 1976a; Pileckis and Monsevičius 1982, 1995; Silfverberg 1992, 2004; Alonso-Zarazaga 2009a.

*austriaca **(Herbst, 1783)**. Pileckis 1968a, c, 1970b, 1976; Silfverberg 1992, 2004; Alonso-Zarazaga 2009a; disproved by Pileckis and Monsevičius (1995).

**Dynastinae** **MacLeay, 1819**.

**Oryctini Mulsant, 1842**.

*Oryctes* **Illiger, 1758**.

*nasicornis* **(Linnaeus, 1758)**. Eichwald 1830; Heyden 1903; Pileckis 1960, 1976a, 1982; Lešinskas and Pileckis 1967; Gaidienė and Ferenca 1992; Silfverberg 1992, 2004; Gaidienė 1993; Pileckis and Monsevičius 1995; Gliaudys 2001; Ferenca 2006b; Krell 2006, 2009; Šablevičius 2011.

**Cetoniinae** **Leach, 1815**.

**Cetoniini Leach, 1815**.

*Oxythyrea* **Mulsant, 1842**.

*funesta* **(Poda, 1761)**. Pileckis 1960, 1968c, 1970b, 1976a, b, 1979; Silfverberg 1992, 2004; Gaidienė 1993; Pileckis and Monsevičius 1995; Gliaudys 2001; Šablevičius 2004, 2011; Ferenca 2006b; Smetana 2006b; Krell 2009; Ivinskis et al. 2009; Inokaitis 2009.

*Tropinota* **Mulsant, 1842**.

*hirta* **(Poda, 1761)**. Pileckis 1959, 1960, 1968c, 1976a; Silfverberg 1992, 2004; Gaidienė 1993; Pileckis and Monsevičius 1995; Ivinskis et al. 2004a; Ferenca 2006b; Smetana 2006b; Krell 2009.

*Cetonia* **Fabricius, 1775**.

*aurata* **(Linnaeus, 1758)**. Eichwald 1830; Heyden 1903; Pileckis 1960, 1976a; Lešinskas and Pileckis 1967; Zajančkauskas and Pileckis 1968; Gaidienė and Ferenca 1992; Silfverberg 1992, 2004; Gaidienė 1993; Pileckis and Monsevičius 1995; Monsevičius 1997; Šablevičius 2000b, 2011; Gliaudys 2001; Tamutis and Zolubas 2001; Ferenca 2006b; Smetana 2006b; Vaivilavičius 2008; Krell 2009.

*Protaetia* **Burmeister, 1842**.

*aeruginosa* **(Linnaeus, 1767)**. Pileckis 1968b, c, 1976a, b, 1979; Silfverberg 1992, 2004; Gaidienė 1993; Pileckis and Monsevičius 1995 (*Netocia*); Inokaitis 2004; Krell 2009.

*cuprea* **(Fabricius, 1775)** = *metallica* (Herbst, 1786). Mazurowa and Mazur 1939; Pileckis 1960, 1976a, 1982; Zajančkauskas and Pileckis 1968; Silfverberg 1992, 2004; Gaidienė 1993; Pileckis and Monsevičius 1995 (*Netocia*); Monsevičius 1997; Gliaudys 2001; Ferenca 2006b; Smetana 2006b; Vaivilavičius 2008; Krell 2009; Šablevičius 2011.

*fieberi* **(Kraatz, 1880)**. Pileckis and Monsevičius 1982, 1995 (*Netocia*); Ivinskis et al. 1984; Silfverberg 1992, 2004; Monsevičius 1997; Inokaitis 2004; Krell 2009.

RDB*lugubris* **(Herbst, 1786)** = *marmorata* (Fabricius, 1792). Pileckis 1960, 1976a; Zajančkauskas and Pileckis 1968; Balevičius 1992; Gaidienė and Ferenca 1992; Silfverberg 1992, 2004; Gaidienė 1993; Auglys 1994; Ostrauskas 1994; Pileckis and Monsevičius 1995 (*Netocia*); Auglys 1996; Ivinskis et al. 1996b, 2004b, 2007a, 2009; Monsevičius 1997, 1998; Pankevičius 2000; Jonaitis et al. 2000; Gliaudys 2001; Ehnström et al. 2003; Meržijevskis 2004; Tamutis 2005b; Ferenca 2006b; Smetana 2006b, 2011; Butvila et al. 2007; Kriaučiūnienė and Zaplatkin 2007; Rašomavičius 2007; Vaivilavičius 2008; Dapkus and Tamutis 2008b; Krell 2009; Noreika 2009; Alekseev 2010b.

**Valgini Mulsant, 1842**.

*Valgus* **Scriba, 1790**.

*hemipterus* **(Linnaeus, 1758)**. Pileckis 1959, 1960, 1968c 1976a, b, 1979; Bercio and Folwaczny 1979; Silfverberg 1992, 2004; Gaidienė 1993; Pileckis and Monsevičius 1995; Šablevičius 2000b, 2003a; Ivinskis et al. 2004a, 2009; Inokaitis 2004, 2009; Ferenca 2004; Alekseev 2006; Smetana 2006b; Krell 2009.

**Trichiini Fleming, 1821**.

*Osmoderma* **LePeletier & Audinet-Serville, 1828**. # 55.

*barnabita* **(Motschulsky, 1845)**. Smetana 2006b; Audisio et al. 2007; Ivinskis et al. 2009; Noreika 2009.

RDB*eremita **(Scopoli, 1763)**. Eichwald 1830; Pileckis 1960, 1968c, 1976a, b, 1979; Balevičius 1992; Silfverberg 1992, 2004; Gaidienė 1993; Auglys 1994; Pileckis and Monsevičius 1995; Auglys 1996; Ivinskis et al. 1996b, 2004b, 2007a, b; Strazdienė 1998; Obelevičius 2000; Gliaudys 2001; Ehnström et al. 2003; Meržijevskis 2004; Inokaitis 2004; Ferenca 2004, 2006b; Tamutis 2005b; Ranius et al. 2005; Butvila et al. 2007; Žitkevičius 2007; Rašomavičius 2007; Vaivilavičius 2008; Šablevičius 2011; disproved by Audisio et al. (2007).

*Gnorimus* **LePeletier & Audinet-Serville, 1828**.

[*nobilis*(**Linnaeus, 1758)**]. Known in Latvia (Telnov 2004), western Belarus (Alexandrovitch et al. 1996), Kaliningrad region (Alekseev and Nikitsky 2008), Denmark, Estonia, southern Sweden(Lundberg and Gustafsson 1995), throughout Poland (Stebnicka 1978).

RDB*variabilis* **(Linnaeus, 1758)** = *octopunctatus* (Fabricius, 1775). Pileckis 1960, 1968c, 1976a, b, 1979; Balevičius 1992; Silfverberg 1992, 2004; Gaidienė 1993; Pileckis and Monsevičius 1995; Ivinskis et al. 1996a, b, 2004a, 2007; 2009; Monsevičius 1997; Gliaudys 2001; Ehnström et al. 2003; Inokaitis 2004; Ferenca 2006b; Tamutis 2005b; Smetana 2006b; Uselis et al. 2007; Rašomavičius 2007; Krell 2009; Bačianskas 2009; Alekseev 2010b.

*Trichius* **Fabricius, 1787**.

*fasciatus* **(Linnaeus, 1758)**. Eichwald 1830; Heyden 1903; Pileckis 1960, 1976a; Lešinskas and Pileckis 1967; Zajančkauskas and Pileckis 1968; Bercio and Folwaczny 1979; Ivinskis et al. 1984; Silfverberg 1992, 2004; Gaidienė 1993; Pileckis and Monsevičius 1995; Monsevičius 1997; Šablevičius 2000b, 2011; Gliaudys 2001; Žiogas and Zolubas 2005; Ferenca 2006b; Smetana 2006b; Krell 2009; Vaivilavičius 2008.

**SCIRTOIDEA Fleming, 1821**.

**EUCINETIDAE Lacordaire, 1857**.

*Eucinetus* **Germar, 1818**.

*haemorrhoidalis* **(Germar, 1818)** = *haemorrhous* (Duftschmid, 1825). Pileckis 1976a; Silfverberg 1992, 2004; Pileckis and Monsevičius 1995; Šablevičius 2004; Ferenca 2004; Ferenca et al. 2004, 2006, 2007; Vít 2006; Audisio 2009.

**CLAMBIDAE Fischer von Waldheim, 1821**.

**Calyptomerinae Crowson, 1955**.

*Calyptomerus* **Redtenbacher, 1849**.

*dubius* **(Marsham, 1802)**. Löbl 2006a, 2009.

**Clambinae** **Fischer von Waldheim, 1821**.

*Clambus* **Fischer von Waldheim, 1821**.

*armadillo* **(DeGeer, 1774)**. Silfverberg 1992, 2004; Pileckis and Monsevičius 1995; Löbl 2006a, 2009.

*gibbulus* **(LeConte, 1850)** = *radula* Endrödy-Youhga, 1960. Silfverberg 1992, 2004; Lundberg and Gustafsson 1995; Löbl 2006a, 2009.

*minutus* **(Sturm, 1807)**. Pileckis 1976a; Pileckis and Monsevičius 1995; Silfverberg 2004.

[*nigrellus* **Reitter, 1914**].Known in Latvia (Vorst et al. 2007), Estonia (Süda 2009), Denmark, Sweden (Lundberg and Gustafsson 1995).

[*pallidulus* **Reitter, 1911**].Known in Denmark (Lundberg and Gustafsson 1995), Sweden (Löbl 2009).

*pubescens* **Redtenbacher, 1849**. Silfverberg 1992, 2004; Lundberg and Gustafsson 1995; Löbl 2006a, 2009.

*punctulum* **(Beck, 1817)** = *borealis* Strand, 1946. Silfverberg 1992, 2004; Lundberg and Gustafsson 1995; Löbl 2006a, 2009.

**SCIRTIDAE Fleming, 1821.** (Helodidae)

**Scirtinae Fleming, 1821**.

*Elodes* **Latreille, 1796**.

[*elongata* **Tournier, 1868** = *tricuspis* Nyholm, 1985]. Known in Latvia (Telnov 2004), Estonia (Silfverberg 2004), Denmark, throughout Sweden (Lundberg and Gustafsson 1995).

[*marginata* **(Fabricius, 1798)**].Known in Denmark, Sweden (Lundberg and Gustafsson 1995).

*minuta* **(Linnaeus, 1767)**. Roubal 1910; Pileckis 1960, 1976a; Monsevičius 1981; Silfverberg 1992, 2004; Gaidienė 1993; Pileckis and Monsevičius 1995; Klausnitzer 2006; Ivinskis et al. 2009.

*pseudominuta* **Klausnitzer, 1971**. # 56.Klausnitzer 2006.

*Microcara* **Thomson, 1859**.

*testacea* **(Linnaeus, 1767)** = *bohemanni* (Mannerheim, 1844). Pileckis 1960, 1976a; Zajančkauskas and Pileckis 1968; Bercio and Folwaczny 1979; Monsevičius 1981, 1997; Silfverberg 1992, 2004; Gaidienė 1993; Pileckis and Monsevičius 1995; Ferenca 2006b; Klausnitzer 2006; Ivinskis et al. 2008; Sapiejewski 2009; Ostrauskas and Ferenca 2010.

*Cyphon* **Paykull, 1799**.

*coarctatus* **Paykull, 1799**. Mazurowa and Mazur 1939; Pileckis 1960, 1976a; Monsevičius 1981, 1997; Silfverberg 1992, 2004; Pileckis and Monsevičius 1995; Klausnitzer 2006.

*hilaris* **Nyholm, 1944**. Monsevičius 1981, 1997; Silfverberg 1992, 2004; Pileckis and Monsevičius 1995; Klausnitzer 2006; Ivinskis et al. 2009.

*kongsbergensis* **Munster, 1924**. Monsevičius 1981, 1997; Silfverberg 1992, 2004; Pileckis and Monsevičius 1995; Klausnitzer 2006.

*laevipennis* **Tournier, 1868** = *phragmiteticola* Nyholm, 1955. Monsevičius 1981, 1997, 1998; Silfverberg 1992, 2004; Pileckis and Monsevičius 1995; Klausnitzer 2006; Ivinskis et al. 2009.

*ochraceus* **Stephens, 1830**. Pileckis 1960, 1976a; Monsevičius 1981, 1997; Silfverberg 1992, 2004; Gaidienė 1993; Pileckis and Monsevičius 1995; Ferenca 2006b; Klausnitzer 2006; Ostrauskas and Ferenca 2010.

*padi* **(Linnaeus, 1767)**. Pileckis 1960, 1976a; Bercio and Folwaczny 1979; Monsevičius 1981, 1997; Silfverberg 1992, 2004; Gaidienė 1993; Pileckis and Monsevičius 1995; Tamutis and Zolubas 2001; Ferenca 2006b; Klausnitzer 2006.

*palustris* **Thomson, 1855**. Mazurowa and Mazur 1939; Pileckis 1960, 1976a; Monsevičius 1981, 1997; Silfverberg 1992, 2004; Pileckis and Monsevičius 1995; Klausnitzer 2006; Sapiejewski 2009.

*pubescens* **(Fabricius, 1792)**. Monsevičius 1981, 1997; Silfverberg 1992, 2004; Pileckis and Monsevičius 1995; Klausnitzer 2006; Sapiejewski 2009.

*punctipennis* **Sharp, 1873**. Monsevičius 1981, 1997; Silfverberg 1992, 2004; Pileckis and Monsevičius 1995; Tamutis and Zolubas 2001; Klausnitzer 2006.

[*ruficeps* **Tournier 1868**].Known in Latvija (Telnov 2004), northwestern Belarus (Alexandrovitch et al. 1996), Poland (Sapiejewski 2009).

*variabilis* **(Thunberg, 1787)**. Roubal 1910; Pileckis 1960, 1963b, 1976a; Zajančkauskas and Pileckis 1968; Bercio and Folwaczny 1979; Monsevičius 1981, 1997; Silfverberg 1992, 2004; Gaidienė 1993; Pileckis and Monsevičius 1995; Ferenca 2006b; Klausnitzer 2006; Dapkus and Tamutis 2007; Ostrauskas and Ferenca 2010.

*Prionocyphon* **Redtenbacher, 1858**.

*serricornis* **(O.F. Müller, 1821)**. Pileckis 1963b, 1976a; Silfverberg 1992, 2004; Pileckis and Monsevičius 1995; Monsevičius 1997; Klausnitzer 2006; Ostrauskas and Ferenca 2010.

*Scirtes* **Illiger, 1807**.

*hemisphaericus* **(Linnaeus, 1758)**. Roubal 1910; Pileckis 1960, 1976a; Monsevičius 1981, 1997; Silfverberg 1992, 2004; Gaidienė 1993; Pileckis and Monsevičius 1995; Klausnitzer 2006.

*orbicularis* **(Panzer, 1793)**. Pileckis and Monsevičius 1995; Silfverberg 2004.

**DASCILLOIDEA Guérin-Ménevillé, 1843 (1834)**.

**DASCILLIDAE Guérin-Ménévillé, 1843(1834)**.

**Dascillinae** **Guérin-Ménévillé, 1843(1834)**.

**Dascillini Guérin-Ménévillé, 1843(1834)**.

*Dascillus* **Latreille, 1796**.

*cervinus* **(Linnaeus, 1758)**. Pileckis 1960, 1963b, 1976a; Silfverberg 1992, 2004; Gaidienė 1993; Pileckis and Monsevičius 1995; Ferenca 2004; Löbl 2006b; Jäch 2009b.

**BUPRESTOIDEA Leach, 1815**.

**BUPRESTIDAE Leach, 1815**.

**Chrysochroinae** **Laporte, 1835**.

**Chrysochroini Laporte, 1835**.

*Chalcophora* **Dejean, 1833**.

*mariana* **(Linnaeus, 1758)**. Eichwald 1830; Mazurowa and Mazur 1939; Ogijewicz 1939; Pileckis 1958a, 1960, 1976a; Lešinskas and Pileckis 1967; Pileckis et al. 1968; Jakaitis and Valenta 1976; Ivinskis et al. 1984; Gaidienė 1993; Silfverberg 1992, 2004; Pileckis and Monsevičius 1995; Monsevičius 1997; Žiogas 1997; Gliaudys 2001; Ehnström et al. 2003; Kubán and Bílý 2006; Kubán 2009; Šablevičius 2011.

**Dicercini Gistel, 1848**.

*Dicerca* **Eschscholtz, 1829** = *Argante* Gistlel, 1834.

*aenea* **(Linnaeus, 1761)**. Pileckis 1960, 1976a; Silfverberg 1992, 2004; Pileckis and Monsevičius 1995; Kubán and Bílý 2006.

*alni* **(Fischer von Waldheim, 1823)**. Pileckis 1960, 1976a; Pileckis et al. 1968; Silfverberg 1992, 2004; Gaidienė 1993; Pileckis and Monsevičius 1995; Ehnström et al. 2003; Ferenca 2004; Kubán and Bílý 2006; Butvila et al. 2007; Kubán 2009.

*furcata* **(Thunberg, 1787)** = *acuminata* (Pallas, 1782) nec (DeGeer, 1774). Richter 1952; Pileckis 1960, 1976a; Silfverberg 1992, 2004; Pileckis and Monsevičius 1995; Ehnström et al. 2003; Ferenca 2004; Kubán and Bílý 2006; Kubán 2009.

*moesta* **(Fabricius, 1792)**. Pileckis 1960, 1976a; Silfverberg 1992, 2004; Gaidienė 1993; Pileckis and Monsevičius 1995; Kubán and Bílý 2006; Kubán 2009.

*Lamprodila* **Motchulsky, 1860.**

RDB*rutilans* **(Fabricius, 1777)**. Pileckis 1968b, 1970a, b, 1976a; Silfverberg 1992, 2004; Gaidienė 1993; Pileckis and Monsevičius 1995 (*Ovalisia*); Ferenca 2003; Ehnström et al. 2003; Inokaitis 2004; Ivinskis et al. 2004a; Tamutis 2005b; Rašomavičius 2007; Vaivilavičius 2008; Dapkus and Tamutis 2008b; Alekseev 2010b.

*Poecilonota* **Eschscholtz, 1829** = *Descarpentriesia* Leraut, 1983.

*variolosa* **(Paykull, 1799)**. Pileckis 1958a, 1960, 1976a; Silfverberg 1992, 2004; Gaidienė 1993; Pileckis and Monsevičius 1995; Ehnström et al. 2003; Kubán and Bílý 2006; Kubán 2009; Inokaitis 2009.

**Buprestinae Leach, 1815**.

**Anthaxiini Gory &Laporte, 1839**.

*Anthaxia* **Eschscholtz, 1829**.

[*godeti* **Gory, 1841** = *submontana* Obenberger, 1930].Known in Latvia (Telnov 2004), Belarus (Alexandrovitch et al. 1996), Kaliningrad region (Kubán 2009), Sweden (Lundberg and Gustafsson 1995), Poland (Kubán 2009).

[*helvetica* **Stierlin, 1868**].Known in Latvia (Telnov 2004), Estonia (Lundberg and Gustafsson 1995), Poland (Kubán 2009).

*manca* **(Linnaeus, 1767)**. Richter 1949; Pileckis 1960, 1970a, 1976a; Silfverberg 1992, 2004; Pileckis and Monsevičius 1995; Kubán 2009.

*nitidula* **(Linnaeus, 1758)**. Richter 1949; Pileckis 1960, 1970a, 1976a; Silfverberg 1992, 2004; Pileckis and Monsevičius 1995; Kubán and Bílý 2006; Kubán 2009.

*quadripunctata* **(Linnaeus, 1758)**. Eichwald 1830; Mazurowa and Mazur 1939; Ogijewicz 1939; Pileckis 1958a, 1960, 1976a; Pileckis et al. 1968; Silfverberg 1992, 2004; Gaidienė 1993; Pileckis and Monsevičius 1995; Monsevičius 1997; Žiogas 1997; Šablevičius 2000b, 2011; Gliaudys 2001; Kubán and Bílý 2006; Kubán 2009; Zeniauskas and Gedminas 2010; Ostrauskas and Ferenca 2010.

[*salicis* **(Fabricius, 1777)**].Known in Latvia (Telnov 2004), Estonia (Lundberg and Gustafsson 1995).

*similis* **Saunders, 1871** = *morio* (Herbst, 1801) nec (Fabricius, 1792). Silfverberg 1992, 2004; Pileckis and Monsevičius 1995.

**Buprestini Leach, 1815**.

*Buprestis* **Linnaeus, 1758**.

*haemorrhoidalis* **Herbst, 1780**. Ogijewicz 1939; Pileckis 1958a, 1960, 1976a; Lešinskas and Pileckis 1967; Silfverberg 1992, 2004; Gaidienė 1993; Pileckis and Monsevičius 1995; Monsevičius 1997; Gliaudys 2001; Ferenca 2004; Kubán and Bílý 2006; Kubán 2009.

*novemmaculata* **Linnaeus, 1767**. Pileckis 1960, 1963b, 1976a; Zajančkauskas and Pileckis 1968; Silfverberg 1992, 2004; Gaidienė 1993; Pileckis and Monsevičius 1995; Monsevičius 1997; Ehnström et al. 2003; Kubán and Bílý 2006; Kubán 2009.

*octoguttata* **Linnaeus, 1758**. Eichwald 1830; Ogijewicz 1939; Pileckis 1958a, 1960, 1976a, 1982; Lešinskas and Pileckis 1967; Pileckis et al. 1968; Bercio and Folwaczny 1979; Ivinskis et al. 1984; Silfverberg 1992, 2004; Gaidienė 1993; Pileckis and Monsevičius 1995; Monsevičius 1997; Gliaudys 2001; Alekseev 2006; Kubán and Bílý 2006; Kubán 2009; Šablevičius 2011.

*rustica* **Linnaeus, 1758**. Eichwald 1830; Pileckis 1958a, 1960, 1976a; Lešinskas and Pileckis 1967; Pileckis et al. 1968; Silfverberg 1992, 2004; Gaidienė 1993; Pileckis and Monsevičius 1995; Gliaudys 2001; Kubán and Bílý 2006; Kubán 2009; Ivinskis et al. 2009.

[*splendens* **Fabricius 1775**].Known in Denmark, Sweden (Lundberg and Gustafsson 1995), Belarus (Alexandrovitch et al. 1996), Poland (Kubán 2009).

**Chrysobothrini Gory & Laporte, 1836**.

*Chrysobothris* **Eschscholtz, 1829**.

*affinis* **(Fabricius, 1794)**. Pileckis 1958a, 1960, 1976a; Silfverberg 1992, 2004; Gaidienė 1993; Pileckis and Monsevičius 1995; Šablevičius 2000b, 2003a; Ferenca 2004; Inokaitis 2009.

*chrysostigma* **(Linnaeus, 1758)**. Pileckis 1958a, 1960, 1963b, 1976a; Silfverberg 1992, 2004; Pileckis and Monsevičius 1995; Gliaudys 2001; Šablevičius 2003a, b; Kubán et al. 2006; Kubán 2009.

[*solieri* **Laporte & Gory, 1837**].Known in northern and western Belarus (Alexandrovitch et al. 1996).

**Melanophilini Bedel, 1921**.

*Melanophila* **Eschscholtz, 1829**.

RDB*acuminata* **(DeGeer, 1774)**. Pileckis 1958a, 1960, 1976; Pileckis et al. 1968; Bercio and Folwaczny 1979; Silfverberg 1992, 2004; Gaidienė 1993; Pileckis and Monsevičius 1995; Ehnström et al. 2003; Kubán and Bílý 2006; Kubán 2009.

*Phaenops* **Dejean, 1833**.

*cyanea* **(Fabricius, 1775)**. Mazurowa and Mazur 1939; Pileckis 1958a, 1960, 1976a; Pileckis et al. 1968; Bercio and Folwaczny 1979; Ivinskis et al. 1984; Silfverberg 1992, 2004; Gaidienė 1993; Pileckis and Monsevičius 1995; Monsevičius 1997; Žiogas 1997; Valenta 2000b; Gliaudys 2001; Gedminas and Lynikienė 2005; Alekseev 2006; Kubán and Bílý 2006; Kubán 2009; Šablevičius 2011.

*formaneki* **Jacobson, 1913**. Tamutis et al. 2008.

**Agrilinae** **Laporte, 1835**.

**Agrilini Laporte, 1835**.

*Agrilus* **Curtis, 1825**.

*angustulus* **(Illiger, 1803)**. Ogijewicz 1939; Pileckis 1960, 1976a; Silfverberg 1992, 2004; Pileckis and Monsevičius 1995; Kubán and Jendek 2006; Kubán 2009.

*ater* **(Linnaeus, 1767)**. Horion 1955; Pileckis 1968b, 1976a; Silfverberg 1992, 2004; Pileckis and Monsevičius 1995; Ehnström et al. 2003; Kubán and Jendek 2006.

[*auricollis* **Kiesenwetter, 1857**].Known in Latvia (Telnov 2004), Belarus (Alexandrovitch et al. 1996).

*betuleti* (**Ratzeburg, 1837**) = *foveicollis* Marseul, 1869. Pileckis 1959, 1960, 1976a; Pileckis et al. 1968; Silfverberg 1992, 2004; Pileckis and Monsevičius 1995; Monsevičius 1997; Kubán and Jendek 2006.

*biguttatus* **(Fabricius, 1777)** = *pannonicus* (Piller & Mitterpacher, 1783). Pileckis 1958a, 1960, 1976a; Lešinskas and Pileckis 1967; Pileckis et al. 1968; Silfverberg 1992, 2004; Gaidienė 1993; Pileckis and Monsevičius 1995; Žiogas 1997; Ferenca 2006b; Kubán and Jendek 2006; Vaivilavičius 2008; Šablevičius 2011.

[*convexicollis* **Radtenbacher, 1849**].Known in Latvia (Telnov 2004), southern Sweden (Lundberg and Gustafsson 1995), Poland (Kubán 2009).

*cuprescens* **Ménétriés, 1832** = *aurichalceus* Radtenbacher, 1849 = *communis* Obenberger, 1924 = *chrysoderes* Bétis, 1912. Pileckis 1962, 1963b, 1976a; Silfverberg 1992, 2004; Pileckis and Monsevičius 1995; Šablevičius 2000b; Kubán and Jendek 2006; Ivinskis et al. 2009.

*cyanescens* **Ratzeburg, 1837** = *coeruleus* (P. Rossi, 1790) nec (Thunberg, 1789). Ogijewicz 1939; Pileckis 1960, 1963b, 1976a; Silfverberg 1992, 2004; Gaidienė 1993; Pileckis and Monsevičius 1995; Kubán and Jendek 2006; Ferenca et al. 2006, 2007; Inokaitis 2009.

*delphinensis* **Abeille de Perrin, 1897** = *pseudocyaneus* auct. nec Kiesenwetter, 1857. Silfverberg 1992, 2004; Pileckis and Monsevičius 1995; Kubán and Jendek 2006; Ivinskis et al. 2009.

*guerini* **Lacordaire, 1835.** Inokaitis 2004, 2009.

*hyperici* **(Creutzer, 1799)**. Horion 1955; Pileckis 1968b, 1976a; Silfverberg 1992, 2004; Pileckis and Monsevičius 1995; Kubán and Jendek 2006.

*integerrimus* **(Ratzeburg, 1839)**. Pileckis 1976a; Silfverberg 1992, 2004; Pileckis and Monsevičius 1995; Kubán and Jendek 2006.

*kaluganus* **Obenberger, 1940**. Silfverberg 1992, 2004; Pileckis and Monsevičius 1995.

*laticornis* **(Illiger, 1803)** = *scaberrimus* Ratzeburg, 1837 = *asperrimus* Marsuel, 1866. Pileckis 1976a; Silfverberg 1992, 2004; Pileckis and Monsevičius 1995; Kubán and Jendek 2006; Kubán 2009; Inokaitis 2009.

*mendax* **Mannerheim, 1837**. Pileckis 1968b, 1976a; Silfverberg 1992, 2004; Pileckis and Monsevičius 1995; Ehnström et al. 2003; Kubán and Jendek 2006; Kubán 2009.

[*olivicolor* **Kiesenwetter, 1857**]. Known in Belarus (Alexandrovitch et al. 1996), Poland, Kaliningrad region (Kubán 2009), southern Sweden (Lundberg and Gustafsson 1995).

*roberti* **Chevrolat, 1837** = *pratensis* (Ratzeburg, 1839). Pileckis 1962, 1963b, 1976a; Silfverberg 1992, 2004; Pileckis and Monsevičius 1995; Kubán and Jendek 2006; Inokaitis 2009.

[*sinuatus* **(Olivier, 1790)**]. Known in Belarus, Sweden, Poland (Kubán 2009), Estonia (Lundberg and Gustafsson 1995).

*subauratus* **(Gebler, 1833)**. Pileckis 1959, 1960, 1976a; Silfverberg 1992, 2004; Pileckis and Monsevičius 1995; Monsevičius 1997; Šablevičius 2000b; Kubán and Jendek 2006; Kubán 2009.

*sulcicollis* **Lacordaire, 1835**. Pileckis 1960, 1976a; Silfverberg 1992, 2004; Pileckis and Monsevičius 1995; Šablevičius 2000b; Kubán and Jendek 2006; Kubán 2009; Inokaitis 2009.

[*suvorovi* **Obenberger, 1935** = *populneus* Schaefer, 1946]. Known in Latvia (Telnov 2004), southern Sweden (Lundberg and Gustafsson 1995), Poland (Kubán 2009).

*viridis*(Linnaeus, 1758). Ogijewicz 1939; Pileckis 1958a, 1960, 1976a; Lešinskas and Pileckis 1967; Pileckis et al. 1968; Silfverberg 1992, 2004; Gaidienė 1993; Pileckis et al. 1994a; Pileckis and Monsevičius 1995; Šablevičius 2000b; Gliaudys 2001; Kubán and Jendek 2006; Kubán 2009; Ostrauskas and Ferenca 2010.

**Aphanisticini Jacquelin du Val, 1859**.

*Aphanisticus* **Latreille, 1810**.

*emarginatus* **(Olivier, 1790)**. Horion 1955; Pileckis 1968, 1976a; Silfverberg 1992, 2004; Pileckis and Monsevičius 1995.

*pusillus* **(Olivier, 1790)**. Silfverberg 1992, 2004; Kubán and Jendek 2006; Kubán 2009.

**Coraebini Bedel, 1921**.

*Coraebus* **Gory & Laporte, 1839**.

*elatus* **(Fabricius, 1787)**. Pileckis and Monsevičius 1982, 1995; Ivinskis et al. 1984; Silfverberg 1992, 2004; Monsevičius 1997.

[*undatus* **(Fabricius, 1787)** = *pruni* (Panzer, 1796)]. Known in Latvia (Telnov 2004), Poland (Kubán 2009).

**Tracheini Laporte, 1835**.

*Habroloma* **Thomson, 1864**.

*nanum* **(Paykull, 1799)** = *geranii* (Silfverberg, 1977). Kubán and Jendek 2006; Kubán 2009.

*Trachys* **Fabricius, 1801**.

*fragariae* **Brisout, 1874**. Silfverberg 1992, 2004; Pileckis and Monsevičius 1995.

*minutus* **(Linnaeus, 1758)**. Heyden 1903; Ogijewicz 1939; Pileckis 1958a, 1960, 1976a; Pileckis et al. 1968; Zajančkauskas and Pileckis 1968; Silfverberg 1992, 2004; Gaidienė 1993; Pileckis and Monsevičius 1995; Monsevičius 1997; Šablevičius 2000b, 2011; Ferenca 2006b; Kubán and Jendek 2006; Kubán 2009.

*scrobiculatus* **Kiesenwetter, 1857** = *pumilus* auct. nec Illiger, 1803. Pileckis 1976a; Silfverberg 1992, 2004; Pileckis and Monsevičius 1995.

*troglodytes* **Gyllenhal, 1817** =

*puncticollis* Abeille de Perrin, 1900. Pileckis 1959, 1960, 1976a; Silfverberg 1992, 2004; Gaidienė 1993; Pileckis and Monsevičius 1995; Kubán and Jendek 2006; Kubán 2009.

**BYRRHOIDEA Latreille, 1804**.

**BYRRHIDAE Latreille, 1804**.

**Byrrhinae** **Latreille, 1804**.

**Simplocariini Mulsant & Rey, 1869**.

*Simplocaria* **Stephens, 1830**.

[*maculosa* **Erichson, 1847**]. Known in Latvia (Vorst et al. 2007), Poland (Mroczkowski 1958).

[*metallica* **(Sturm, 1807)**]. Known in Latvia (Telnov 2004), Sweden (Lundberg and Gustafsson 1995), Poland (Mroczkowski 1958).

*semistriata* **(Fabricius, 1794)**. Pileckis 1968b, 1976a; Silfverberg 1992, 2004; Pileckis and Monsevičius 1995; Monsevičius 1997; Šablevičius 2003a, 2004; Jaeger and Pütz 2006; Sánchez-Terrón 2009.

**Morychini El Moursy, 1961**.

*Morychus* **Erichson, 1846**.

*aeneus*(Fabricius, 1775). Pileckis 1960, 1976a; Bercio and Folwaczny 1979; Silfverberg 1992, 2004; Gaidienė 1993; Pileckis and Monsevičius 1995; Monsevičius 1997, 1998; Tamutis and Zolubas 2001; Šablevičius 2004; Jaeger and Pütz 2006; Sánchez-Terrón 2009.

**Byrrhini Latreille, 1804**.

*Lamprobyrrhulus* **Ganglbauer, 1902**.

*nitidus* **(Schaller, 1783)**. Pileckis 1960, 1976a; Gaidienė 1993; Pileckis and Monsevičius 1995; Monsevičius 1997; Silfverberg 1992, 2004; Šablevičius 2004; Ferenca 2006b; Jaeger and Pütz 2006; Sánchez-Terrón 2009.

*Cytilus* **Erichson, 1846**.

*auricomus* **(Duftschmid, 1825)**. Gaidienė 1993; Ferenca 2003; Silfverberg 2004; Ivinskis et al. 2009.

*sericeus* **(Forster, 1771)**. Eichwald 1830; Pileckis 1960, 1976a; Zajančkauskas and Pileckis 1968; Silfverberg 1992, 2004; Gaidienė 1993; Pileckis and Monsevičius 1995; Monsevičius 1997; Šablevičius 2000b; Tamutis and Zolubas 2001; Ferenca 2006b; Jaeger and Pütz 2006; Sánchez-Terrón 2009.

*Byrrhus* **Linnaeus, 1767**.

*arietinus* **(Steffahny, 1842)**. Mazurowa and Mazur 1939; Pileckis 1960, 1976a; Silfverberg 1992, 2004; Pileckis and Monsevičius 1995; Monsevičius 1997; Ferenca 2006b; Jaeger and Pütz 2006; Sánchez-Terrón 2009.

*fasciatus* **(Forster, 1771)**. Pileckis 1968b, 1976a; Silfverberg 1992, 2004; Gaidienė 1993; Pileckis and Monsevičius 1995; Monsevičius 1997; Žiogas and Zolubas 2005; Jaeger and Pütz 2006; Sánchez-Terrón 2009; Ivinskis et al. 2009.

*pilula* **(Linnaeus, 1758)**. Eichwald 1830; Heyden 1903; Mazurowa and Mazur 1939; Pileckis 1960, 1976a; Lešinskas and Pileckis 1967; Silfverberg 1992, 2004; Gaidienė 1993; Pileckis and Monsevičius 1995; Monsevičius 1997; Šablevičius 2000b; Gliaudys 2001; Tamutis and Zolubas 2001; Ferenca 2006b; Jaeger and Pütz 2006.

*pustulatus* **(Forster, 1771)**. Eichwald 1830; Pileckis 1960, 1976a; Silfverberg 1992, 2004; Gaidienė 1993; Pileckis and Monsevičius 1995; Gliaudys 2001; Ferenca 2004; Jaeger and Pütz 2006; Sánchez-Terrón 2009.

*Porcinolus* **Mulsant & Rey, 1869**.

*murinus* **(Fabricius, 1794)**. Pileckis 1968b, 1976a; Silfverberg 1992, 2004; Gaidienė 1993; Pileckis and Monsevičius 1995; Jaeger and Pütz 2006.

**Syncalyptinae** **Mulsant & Rey, 1869**.

**Syncalyptini Mulsant & Rey, 1869**.

*Chaetophora* **Kirby & Spence, 1823** = *Syncalypta* Dillwyn, 1829.

[*spinosa* **(P. Rossi, 1794)**]. Known in Latvia (Telnov 2004), throughout Belarus (Alexandrovitch et al. 1996), Denmark, southern Sweden (Lundberg and Gustafsson 1995), Poland (Mroczkowski 1958).

*Curimopsis* **Ganglbauer, 1902**.

[*nigrita* **(Palm, 1934)**]. Known in Denmark, southern Sweden (Lundberg and Gustafsson 1995), northern Poland (Mroczkowski 1958).

*paleata* **(Erichson, 1846)**. Monsevičius 1988b; Silfverberg 1992, 2004; Pileckis and Monsevičius 1995.

*setigera* **Duftschmid, 1825** = *setosa* (Waltl, 1838). Bercio and Folwaczny 1979; Miländer et al. 1984; Silfverberg 1992, 2004; Pileckis and Monsevičius 1995.

**ELMIDAE Curtis, 1830**.

**Larainae** **LeConte, 1861**.

**Laraini LeConte, 1861**.

*Potamophilus* **Germar, 1811**.

[*acuminatus* **(Fabricius, 1792)**]. Known in northern Belarus (Alexandrovitch et al. 1996), Poland (Więźlak 1986).

**Elminae** **Curtis, 1830**.

**Elmini Curtis, 1830**

*Stenelmis* **Dufour, 1835**.

[*canaliculatus* **(Gyllenhal, 1808)**].Known in southern Sweden (Lundberg and Gustafsson 1995), Poland (Więźlak 1986). 

*Elmis* Latreille, 1798.

*aenea* **(O.F. Müller, 1806)**. Silfverberg 1992, 2004; Jäch et al. 2006; Kovács et al. 2008; Jäch 2009a; Alekseev 2010a.

*maugetii* **Latreille, 1806**. Pileckis and Monsevičius 1995; Kovács et al. 2008; Alekseev 2010a; Kazlauskas and Pileckis 1968(*Helmis maugei magelei*); Pileckis 1976a.

[*obscura* **(O.F. Müller, 1806)**].Known in Latvia (Telnov 2004), Poland (Więźlak 1986).

*Esolus* **Mulsant & Rey, 1872**.

[*angustatus* **(O.F. Müller, 1821)**].Known in Latvia (Telnov 2004), southern Sweden (Lundberg and Gustafsson 1995), Poland (Więźlak 1986).

*pygmaeus* **(O.F. Müller, 1806)**. Kazlauskas and Pileckis 1968; Pileckis 1976a; Silfverberg 1992, 2004; Pileckis and Monsevičius 1995; Alekseev 2010a.

*Oulimnius* **DesGozis, 1886**.

*troglodytes* **(Gyllenhal, 1827)**. Kazlauskas and Pileckis 1968; Pileckis 1976a; Silfverberg 1992, 2004; Pileckis and Monsevičius 1995; Jäch 2009a; Alekseev 2010a.

*tuberculatus* **(O.F. Müller, 1806)**. Kazlauskas and Pileckis 1968; Pileckis 1976a; Silfverberg 1992, 2004; Gaidienė 1993; Pileckis and Monsevičius 1995; Jäch et al. 2006; Kovács et al. 2008; Jäch 2009a; Alekseev 2010a.

*Limnius* **Illiger, 1802** = *Latelmis* Reitter, 1883.

[*intermedius* **Fairmaire, 1881**].Known in Denmark (Lundberg and Gustafsson 1995), Belarus (Jäch 2009a).

*muelleri* **(Erichson, 1847)**. Kazlauskas and Pileckis 1968; Pileckis 1976a; Silfverberg 1992, 2004; Pileckis and Monsevičius 1995; Alekseev 2010a.

[*opacus* **(O.F. Müller, 1806)**].Known in Latvia (Telnov 2004), Poland (Więźlak 1986).

*volckmari* **(Panzer, 1793)**. Kazlauskas and Pileckis 1968; Pileckis 1976a; Silfverberg 1992, 2004; Pileckis and Monsevičius 1995; Jäch et al. 2006; Kovács et al. 2008; Jäch 2009a; Alekseev 2010a.

*Normandia* **Pic, 1900**.

*nitens* **(O.F. Müller, 1817)**. Kazlauskas and Pileckis 1968; Pileckis 1976a; Silfverberg 1992, 2004; Pileckis and Monsevičius 1995; Jäch et al. 2006; Jäch 2009a; Alekseev 2010a.

*Riolus* **Mulsant & Rey, 1872**.

*cupreus* **(O.F. Müller, 1806)**. Kazlauskas and Pileckis 1968; Pileckis 1976; Silfverberg 1992, 2004; Pileckis and Monsevičius 1995; Jäch et al. 2006; Kovács et al. 2008; Jäch 2009a; Alekseev 2010a.

*Macronychus* **O.F. Müller, 1806**.

*quadrituberculatus* **O.F. Müller, 1806**. Kazlauskas and Pileckis 1968; Pileckis 1976a; Silfverberg 1992, 2004; Pileckis and Monsevičius 1995; Jäch et al. 2006; Kovács et al. 2008; Jäch 2009a; Alekseev 2010a.

**DRYOPIDAE Billberg, 1820**.

*Pomatinus* **Sturm, 1853**.

*substriatus* **(O.F. Müller, 1806)**. Pileckis and Monsevičius 1995; Silfverberg 2004; Löbl and Smetana 2006; Kovács et al. 2008; Ivinskis et al. 2009; Alekseev 2010a.

*Dryops* **Olivier, 1791**.

*anglicanus* **Edwards, 1909**. Monsevičius and Pankevičius 2001; Kodada and Jäch 2006.

*auriculatus* **(Geoffroy, 1785)**. Kazlauskas and Pileckis 1968; Pileckis 1976a; Bercio and Folwaczny 1979; Silfverberg 1992, 2004; Gaidienė 1993; Pileckis and Monsevičius 1995; Monsevičius 1997; Kodada and Jäch 2006; Jäch 2009a; Alekseev 2010a.

*ernesti* **DesGozis, 1886**. Kazlauskas and Pileckis 1968; Pileckis 1976a; Silfverberg 1992, 2004; Gaidienė 1993; Pileckis and Monsevičius 1995; Kodada and Jäch 2006; Jäch 2009a; Alekseev 2010a.

[*griseus* **(Erichson, 1847)**].Known in Latvia (Telnov 2004), Belarus (Alexandrovitch et al. 1996), Kaliningrad region (Alekseev 2010a), Estonia, Denmark Sweden (Lundberg and Gustafsson 1995), Poland (Więźlak 1986).

*luridus* **(Erichson, 1847)**. Kazlauskas and Pileckis 1968; Pileckis 1976a; Strazdienė 1976, 1988; Silfverberg 1992, 2004; Gaidienė 1993; Pileckis and Monsevičius 1995; Kodada and Jäch 2006; Jäch 2009a; Alekseev 2010a.

*lutulentus* **(Erichson, 1847)**. Monsevičius and Pankevičius 2001.

*nitidulus* **(Heer, 1841)**. Pileckis 1960, 1976a; Kazlauskas and Pileckis 1968; Silfverberg 1992, 2004; Pileckis and Monsevičius 1995; Ferenca 2006b; Kodada and Jäch 2006; Jäch 2009a; Alekseev 2010a.

[*similaris* **Bollow, 1936**].Known in Latvia, Estonia, Denmark, southern Sweden (Lundberg and Gustafsson 1995), Poland (Więźlak 1986).

*viennensis* **(Laporte, 1840)**. Mazurowa and Mazur 1939; Pileckis 1960, 1976a; Kazlauskas and Pileckis 1968; Silfverberg 1992, 2004; Pileckis and Monsevičius 1995; Kodada and Jäch 2006; Jäch 2009a; Alekseev 2010a.

**LIMNICHIDAE Erichson, 1846**. (Byrrhidae)

**Limnichinae** **Erichson, 1846**.

**Limnichini Erichson, 1846**.

*Limnichus* **Dejean, 1821**.

[*incanus* **Kiesenwetter, 1838**].Known in northwestern Belarus (Alexandrovitch et al. 1996).

*pygmaeus* **(Sturm, 1807)**.Pileckis and Monsevičius 1995; Silfverberg 2004; Hernando and Ribera 2006; Ribera and Hernando 2009.

*sericeus* **(Duftschmid, 1825)**.Ferenca et al. 2002.

*Pelochares* **Mulsant & Rey, 1872**.

*versicolor* **(Waltl, 1838)**. Ferenca et al. 2002, 2006; 2007; Šablevičius 2004.

**HETEROCERIDAE MacLeay, 1825**.

**Heterocerinae** **MacLeay, 1825**.

**Heterocerini MacLeay, 1825**

*Heterocerus* **Fabricius, 1792**.

*fenestratus* **(Thunberg, 1784)**. Pileckis 1960, 1976a; Bercio and Folwaczny 1979; Silfverberg 1992, 2004; Gaidienė 1993; Pileckis and Monsevičius 1995; Monsevičius 1997; Ferenca 2006b; Mascagni 2006, 2009.

*flexuosus* **Stephens, 1828**. Pileckis and Monsevičius 1995; Silfverberg 2004; Mascagni 2006, 2009.

[*fossor* **Kiesenwetter, 1843**].Known in Belarus (Alexandrovitch et al. 1996), southern Sweden (Lundberg and Gustafsson 1995).

*fusculus* **Kiesenwetter, 1843**. Bercio and Folwaczny 1979; Monsevičius 1988b, 1997; Silfverberg 1992, 2004; Pileckis and Monsevičius 1995; Mascagni 2006; 2009; Ivinskis et al. 2009.

*marginatus* **(Fabricius, 1787)**. Pileckis 1976a; Bercio and Folwaczny 1979; Silfverberg 1992, 2004; Gaidienė 1993; Pileckis and Monsevičius 1995; Žiogas and Zolubas 2005; Mascagni 2006, 2009.

*obsoletus* **Curtis, 1828**. Bercio and Folwaczny 1979; Silfverberg 1992, 2004; Pileckis and Monsevičius 1995; Mascagni 2006, 2009.

**Augylini Pacheco, 1964**.

*Augyles* **Shiödte, 1866**.

[*aureolus* **(Schiödte, 1866)**].Known in Denmark (Lundberg and Gustafsson 1995), Finland (Mascagni 2006, 2009).

*hispidulus* **Kiesenwetter, 1843**. Bercio and Folwaczny 1979; Pileckis and Monsevičius 1982, 1995 (*Heterocerus*); Silfverberg 1992, 2004; Gaidienė 1993; Mascagni 2006, 2009.

*intermedius* **Kiesenwetter, 1843**. Pileckis and Monsevičius 1995 (*Heterocerus*); Silfverberg 2004; Šablevičius 2003a, 2004; Mascagni 2006, 2009; Ivinskis et al. 2009.

[*maritimus* **Guerin-Meneville, 1844**]. Known in Latvia (Telnov 2004).

[*pruinosus* **Kiesenwetter, 1843**].Known in Latvia (Telnov 2004), Poland (Mascagni 2006, 2009).

[*senescens* **Kiesenwetter, 1865**].Known in southern Sweden (Lundberg and Gustafsson 1995).

**PSEPHENIDAE Lacordaire, 1854**. (Elmidae)

**Eubriinae** **Lacordaire, 1857**.

*Eubria* **Germar, 1818**.

*palustris* **Germar, 1818**. Pileckis and Monsevičius 1995; Silfverberg 2004.

**ELATEROIDEA Leach, 1815**.

**EUCNEMIDAE Eschscholtz, 1829**.

**Melasinae** **Fleming, 1821**.

**Melasini Fleming, 1821**

*Isoriphis* **Boisduval & Lacordaire 1835**.

[*marmottani* **Bonvouloir, 1871**]. Known in Estonia, Sweden (Silfverberg 2004), Poland (Burakowski 1991).

*melasoides* **Castelnau, 1825**.Ferenca and Tamutis 2009.

*Melasis* **Olivier, 1790**.

*buprestoides* **(Linnaeus, 1761)**. Ferenca et al. 2006, 2007; Tamutis and Ferenca 2006.

**Hylocharini Jacquelin du Val, 1859**.

*Hylochares* **Latreille, 1834**.

[*cruentatus* **(Gyllenhal, 1808)**].Known in Latvia (Telnov 2004), Estonia (Lundberg and Gustafsson 1995), northern Poland (Burakowski 1991), Belarus (Alexandrovitch et al. 1996).

**Calyptocerini Muona, 1993**.

*Otho* **Lacordaire, 1857**.

[*sphondyloides* **(Germar, 1818)**].Known in Latvia (Telnov 2004), western Belarus (Alexandrovitch et al. 1996), Poland (Burakowski 1991).

**Xylobiini Reitter, 1911**.

*Xylophilus* **Mannerheim, 1823** = *Xylobius* Latreille, 1834.

[*corticalis* **(Paykull, 1800)**].Known in Latvia (Telnov 2004), Kaliningrad region (Muona 2009), western Belarus (Alexandrovitch et al. 1996), Denmark, southern Sweden (Lundberg and Gustafsson 1995), Estonia (Silfverberg 2004), Poland (Burakowski 1991).

[*testaceus* **(Herbst, 1806)**].Known in western Belarus (Alexandrovitch et al. 1996), northern Poland (Burakowski 1991).

**Epiphanini Muona, 1993**.

*Hylis* **DesGozis, 1886** = *Hypocaelus* Guérin-Ménéville, 1843, nec Dejean, 1833.

[*cariniceps* **(Reitter, 1902)**].Known in southern Sweden (Lundberg and Gustafsson 1995), Estonia (Süda 2009), Poland (Burakowski 1991).

[*foveicollis* **(Thomson, 1847)** = *fleischeri* (Olexa, 1954)].Known in Latvia (Telnov 2004), Denmark, southern Sweden (Lundberg and Gustafsson 1995), Poland (Burakowski 1991). 

[*olexai* **(Palm, 1955)**].Known in Latvia (Telnov 2004), Denmark, southern Sweden (Lundberg and Gustafsson 1995), Poland (Burakowski 1991).

*procerulus* **(Mannerheim, 1823)**. Pileckis and Jakaitis 1989; Silfverberg 1992, 2004; Pileckis and Monsevičius 1995; Ferenca 2002; Muona 2007a, 2009; Ostrauskas and Ferenca 2010.

**Dirhagini Reitter, 1911**.

*Clypeorhagus* **Olexa, 1975**.

[*clypeatus* **(Hampe, 1850)**].Known in Latvia (Telnov 2004), Poland (Burakowski 1991).

*Microrhagus* **Dejean, 1833** = *Dirhagus* Latreille, 1834.

*emyi* **(Rouget, 1855)**. Tamutis 2003.

*lepidus* **Rosenhauer, 1847** = *lindbergi* (Palm, 1958). Pileckis and Monsevičius 1995; Šablevičius 2001, 2003a; Silfverberg 2004; Muona 2007a, 2009.

*pygmaeus* **(Fabricius, 1793)**. Tamutis 2003; Muona 2007a; Ostrauskas and Ferenca 2010.

*Rhacopus* **Hampe, 1855**.

[*attenuatus* **(Maeklin, 1845)**].Known in northeastern Poland (Burakowski 1991), Estonia (Süda 2009), Finland (Lundberg and Gustafsson 1995).

*sahlbergi* **(Mannerheim, 1823)**.Ostrauskas and Ferenca 2010.

**Eucneminae** **Eschscholtz, 1829**.

**Eucnemini Eschscholtz, 1829**.

*Eucnemis* **Ahrens, 1812**.

*capucina* **Ahrens, 1812**. Pileckis 1976a; Silfverberg 1992, 2004; Gaidienė 1993; Pileckis and Monsevičius 1995; Ferenca et al. 2006, 2007; Muona 2007a, 2009.

**Macraulacinae** **Fleutiaux, 1923**.

**Macraulacini Fleutiaux, 1923**.

*Dromaeolus* **Kiesenwetter, 1858**.

[*barnabita* **(Villa, 1838)**].Known in Sweden (Lundberg and Gustafsson 1995), Poland (Burakowski 1991).

**TROSCIDAE Laporte, 1840**.

*Aulonothroscus* **Horn, 1890**.

[*brevicollis* **(Bonvouloir, 1859)**]. Known in Latvia (Telnov 2004), Belarus (Alexandrovitch et al. 1996), Poland (Burakowski 1991).

[*laticollis* **(Rybinski, 1897)**]. Known in Belarus (Alexandrovitch et al. 1996), Poland (Burakowski 1991).

*Trixagus* **Kugelann, 1794** = *Troscus* Latreille, 1796.

[*atticus* **Reitter, 1921**]. Known in Denmark, Seweden (Silfverberg 2004).

*carinifrons* **(Bonvouloir, 1859)**. Pileckis 1976a; Silfverberg 1992, 2004; Pileckis and Monsevičius 1995.

*dermestoides* **(Linnaeus, 1767)**. Pileckis and Monsevičius 1982, 1995; Silfverberg 1992, 2004; Gaidienė 1993; Monsevičius 1997; Leseigneur 2007a, 2009; Alekseev 2008a; Ostrauskas and Ferenca 2010.

[*elateroides* **(Heer, 1841)**]. Known in Latvia (Telnov 2004), Poland (Leseigneur 2007a, 2009).

[*exul* **(Bonvouloir, 1859)**]. Known in Latvia (Telnov 2004), Denmark, southern Sweden (Lundberg and Gustafsson 1995), northern Poland (Burakowski 1991).

[*leseigneuri* **Muona, 2002**].Known in Sweden (Silfverberg 2004).

[*meybohmi* **Leseigneur, 2005**]. Known in Latvia (Telnov et al. 2007).

**ELATERIDAE Leach, 1815**.

**Agrypninae** **Candèze, 1857** = Pyrophorinae Candeze, 1863.

**Agrypnini Candèze, 1857**.

*Agrypnus* **Linnaeus, 1758**.

*murinus* **(Linnaeus, 1758)**. Eichwald 1830; Heyden 1903; Ogijewicz 1929, 1931; 1939; Pileckis 1960, 1976a; Lešinskas and Pileckis 1967; Silfverberg 1992, 2004; Gaidienė 1993; Pileckis and Monsevičius 1995; Monsevičius 1997; Šablevičius 2000b, 2011; Gliaudys 2001; Tamutis and Zolubas 2001; Ferenca 2006b; Tamutis et al. 2006, 2007; Cate 2007a, 2009; Vaivilavičius 2008.

*Lacon* **Laporte, 1838** = *Danosoma* Thomson, 1859.

[*lepidopterus* **(Panzer, 1801)**]. Known in Latvia (Telnov 2004), Denmark, southern Sweden (Lundberg and Gustafsson 1995), northern Poland (Tarnawski and Buchholz 2008a), Balarus (Alexandrovitch et al. 1996).

*querceus* **(Herbst, 1784)**. Pileckis 1976a; Silfverberg 1992, 2004; Gaidienė 1993; Cate 2007a, 2009.

*Danosoma* **Thomson, 1859**.

[*conspersum* **(Gyllenhal, 1808)**]. Known in Latvia (Telnov 2004), western Belarus (Alexandrovitch et al. 1996), Estonia, Sweden (Lundberg and Gustafsson 1995), northeastern Poland (Tarnawski and Buchholz 2008a).

*fasciatum* **(Linnaeus, 1758)**. Pileckis and Jakaitis 1982; Silfverberg 1992, 2004; Pileckis and Monsevičius 1995 (*Lacon*); Ferenca 2004; Cate 2007a, 2009; Inokaitis 2009; Tamutis et al. 2010.

**Lissominae** **Laporte, 1835** (Throscidae).

*Drapetes* **Dejean, 1821**.

*mordelloides* **(Host, 1789)** = *cinctus* (Panzer, 1796) = *biguttatus* (Piller & Mitterpacher, 1783) nec (Fabricius, 1777). Ferenca 1988; Silfverberg 1992, 2004; Pileckis and Monsevičius 1995; Monsevičius 1997; Ehnström et al. 2003; Cate 2007a; Tamutis et al. 2010.

**Dendrometrinae** **Gistel, 1848** = Athoinae Candeze, 1859.

**Dendrometrini Gistel, 1848**.

*Hemicrepidius* **Germar, 1839** = *Pseudathous* Méquignot, 1930.

*hirtus* **(Herbst, 1784)**. Ogijewicz 1939; Pileckis 1960, 1976a; Lešinskas and Pileckis 1967; Zajančkauskas and Pileckis 1968; Pileckis and Vengeliauskaitė 1977, 1996; Silfverberg 1992, 2004; Gaidienė 1993; Pileckis and Monsevičius 1995 (*Athous*); Monsevičius 1997; Šablevičius 2000b; Tamutis and Zolubas 2001; Ferenca 2006b; Tamutis et al. 2006, 2007; Cate 2007a, 2009.

*niger* **(Linnaeus, 1758)**. Heyden 1903; Ogijewicz 1939; Pileckis 1960, 1976a; Lešinskas and Pileckis 1967; Zajančkauskas and Pileckis 1968; Pileckis and Vengeliauskaitė 1977, 1996; Strazdienė 1981, 1988; Silfverberg 1992, 2004; Gaidienė 1993; Pileckis and Monsevičius 1995 (*Athous*); Monsevičius 1997; Šablevičius 2000b; Gliaudys 2001; Ferenca 2006b; Tamutis et al. 2006, 2007; Cate 2007a, 2009; Ostrauskas and Ferenca 2010.

*Crepidophorus* **Mulsant & Guillebeau, 1853**.

*mutilatus* **(Rosenhauer, 1847)**. Pileckis 1960, 1976a; Silfverberg 1992, 2004; Pileckis and Monsevičius 1995 (*Athous*); Ferenca 2006b; Cate 2007a, 2009; Tamutis et al. 2010

*Athous* **Eschscholtz, 1829**.

*haemorrhoidalis* **(Fabricius, 1801)**. Ogijewicz 1929, 1931, 1939; Pileckis 1960, 1976a; Zajančkauskas and Pileckis 1968; Strazdienė 1976; Silfverberg 1992, 2004; Gaidienė 1993; Pileckis and Monsevičius 1995; Monsevičius 1997; Gliaudys 2001; Ferenca 2006b; Cate 2007a, 2009; Vaivilavičius 2008.

*subfuscus* **(O.F. Müller, 1764)**. Heyden 1903; Ogijewicz 1939; Pileckis 1960, 1976a; Zajančkauskas and Pileckis 1968; Strazdienė 1976, 1981, 1988; Silfverberg 1992, 2004; Gaidienė 1993; Pileckis and Monsevičius 1995; Monsevičius 1997; Šablevičius 2000b; Tamutis and Zolubas 2001; Žiogas and Zolubas 2005; Ferenca 2006b; Cate 2007a, 2009; Vaivilavičius 2008; Ivinskis et al. 2008; Ostrauskas and Ferenca 2010.

*vittatus* **(Fabricius, 1792)**. Heyden 1903; Pileckis 1960, 1976a; Silfverberg 1992, 2004; Gaidienė 1993; Pileckis and Monsevičius 1995; Šablevičius 2000b; Ferenca 2006b; Cate 2007a, 2009; Vaivilavičius 2008; Tamutis et al. 2010.

*Pheletes* **Kiesenwetter, 1858**.

[*aeneoniger* **(DeGeer, 1774)**].Known in Estonia, Denmark, throughout Sweden (Lundberg and Gustafsson 1995), Poland (Tarnawski and Buchholz 2008b).

*Limoniscus* Reitter, 1905.

*violaceus* **(O.F. Müller, 1821)**].Known in Estonia, Denmark (Lundberg and Gustafsson 1995), Poland (Tarnawski and Buchholz 2008b).

*Harminius* **Fairmaire, 1851** = *Diacanthous* Reitter, 1905.

*undulatus* **(DeGeer, 1774)**. Pileckis and Jakaitis 1982; Silfverberg 1992, 2004; Pileckis and Monsevičius 1995; Pankevičius 2000; Ferenca et al. 2002; Cate 2007a, 2009; Inokaitis 2009; Tamutis et al. 2010.

*Stenagostus* **Thomson, 1859** = *Athous* Eschscholtz, 1829.

[*rhombeus* **(Olivier, 1790)** = *villosus* auct. nec (Geoffroy, 1785)].Known in Denmark, southern Sweden (Lundberg and Gustafsson 1995), Belarus (Alexandrovitch et al. 1996), northern Poland (Tarnawski and Buchholz 2008b).

RDB*rufus* **(DeGeer, 1774)**. Pileckis 1960, 1970b, 1976a; Bercio and Folwaczny 1979; Pileckis and Jakaitis 1982; Gaidienė 1993; Silfverberg 1992, 2004; Pileckis and Monsevičius 1995; Tamutis 2005b; Ferenca 2006a; Rašomavičius 2007; Cate 2007a, 2009; Alekseev 2010b; Šablevičius 2011.

*Denticollis* **Piller & Mitterpacher, 1783**.

[*borealis* **(Paykull, 1800)**]. Known in Latvia (Telnov 2004), northern Belarus (Alexandrovitch et al. 1996), Estonia, Sweden (Lundberg and Gustafsson 1995), Poland (Tarnawski and Buchholz 2008b).

*linearis* **(Linnaeus, 1758)**. Ogijewicz 1939; Pileckis 1960, 1976a; Silfverberg 1992, 2004; Gaidienė 1993; Pileckis and Monsevičius 1995; Monsevičius 1997; Šablevičius 2000b; Tamutis and Zolubas 2001; Ehnström et al. 2003; Gedminas 2005; Ferenca 2006a; Gedminas et al. 2007; Cate 2007a, 2009; Ostrauskas and Ferenca 2010.

*rubens* **Piller & Mitterpacher, 1783**. Ehnström et al. 2003; Tamutis et al. 2010.

*Cidnopus* **Thomson, 1859**.

*aeruginosus* **(Olivier, 1790)**. Heyden 1903; Ogijewicz 1929, 1931, 1939; Pileckis 1960, 1976a; Lešinskas and Pileckis 1967; Zajančkauskas and Pileckis 1968; Silfverberg 1992, 2004; Gaidienė 1993; Pileckis and Monsevičius 1995; Monsevičius 1997; Ferenca 2006b; Cate 2007a, 2009.

*pilosus* **(Leske, 1785)**. Pileckis 1960, 1976a; Silfverberg 1992, 2004; Pileckis and Monsevičius 1995; Ferenca 2006b; Cate 2007a, 2009; Tamutis et al. 2010.

*Limonius* **Eschscholtz, 1829** *=* *Kibunea* Kishii, 1966.

*minuta* **(Linnaeus, 1758)**. Ogijewicz 1939; Pileckis 1960, 1976a; Silfverberg 1992, 2004; Gaidienė 1993; Pileckis and Monsevičius 1995; Ferenca 2006b; Cate 2007a, 2009; Tamutis et al. 2010.

*Nothodes* **LeConte, 1861**.

[*parvulus* **(Panzer, 1799)**]. Known in Latvia (Telnov 2004), Estonia (Lundberg and Gustafsson 1995), Poland (Tarnawski and Buchholz 2008b).

**Hypnoidini Schwarz, 1906 (1860)**.

*Hypnoidus* Dillwyn, 1829.

*riparius* **(Fabricius, 1792)**. Ogijewicz 1939; Pileckis 1960, 1976a; Silfverberg 1992, 2004; Gaidienė 1993; Pileckis and Monsevičius 1995; Ferenca 2004; Cate 2007a, 2009; Tamutis et al. 2010.

[*rivularius* **(Gyllenhal, 1808)** = *consobrinus* (Mulsant & Guillebeau, 1855)].Known in Latvia (Telnov 2004), Belarus (Alexandrovitch et al. 1996), Estonia, Sweden (Lundberg and Gustafsson 1995), Poland (Tarnawski and Buchholz 2008b).

**Prosternini Gistel, 1856** = Ctenicerini Fleutiaux, 1936.

*Ctenicera* **Latreille, 1829** = *Corymbites* Latreille, 1834.

[*cuprea **(Fabricius, 1775)**]. # 57. Monsevičius 1988b, 1997; Silfverberg 1992, 2004; Pileckis and Monsevičius 1995; Cate 2007a, 2009; disproved by Tamutis et al. (2010).

*pectinicornis* **(Linnaeus, 1758)**. Eichwald 1830; Heyden 1903; Ogijewicz 1939; Pileckis 1960, 1970a, 1976a, b, 1979; Lešinskas and Pileckis 1967; Zajančkauskas and Pileckis 1968; Silfverberg 1992, 2004; Gaidienė 1993; Pileckis and Monsevičius 1995; Monsevičius 1997; Šablevičius 2000b, 2011; Gliaudys 2001; Ferenca 2006b; Cate 2007a, 2009; Tamutis et al.2010.

*Liotrichus* **Kiesenwetter, 1858**.

*affinis* **(Paykull, 1800)**. Pileckis 1960, 1976a; Silfverberg 1992, 2004; Gaidienė 1993; Pileckis and Monsevičius 1995; Monsevičius 1997; Ferenca 2006b; Cate 2007a, 2009.

*Orithales* **Kiesenwetter, 1858**.

*serraticornis* **(Paykull, 1800)**. Pileckis and Monsevičius 1995; Silfverberg 2004.

*Actenicerus* **Kiesenwetter, 1858**.

*sjaelandicus* **(O.F. Müller, 1764)**. Heyden 1903; Ogijewicz 1939; Pileckis 1960, 1976a; Lešinskas and Pileckis 1967; Zajančkauskas and Pileckis 1968; Strazdienė 1976; Silfverberg 1992, 2004; Gaidienė 1993; Pileckis and Monsevičius 1995; Monsevičius 1997; Ferenca 2006b; Cate 2007a, 2009; Ivinskis et al. 2008; Tamutis et al. 2010.

*Prosternon* **Latreille, 1834**.

*tessellatum* **(Linnaeus, 1758)**. Eichwald 1830; Heyden 1903; Ogijewicz 1939; Pileckis 1960, 1976a; Zajančkauskas and Pileckis 1968; Strazdienė 1981, 1988; Silfverberg 1992, 2004; Gaidienė 1993; Pileckis and Monsevičius 1995; Monsevičius 1997; Gliaudys 2001; Ferenca 2006b; Cate 2007a, 2009.

*Anostirus* **Thomson, 1859**.

*castaneus* **(Linnaeus, 1758)**. Lentz 1879; Pileckis 1968b, 1976a; Silfverberg 1992, 2004; Gaidienė 1993; Pileckis and Monsevičius 1995; Tamutis and Zolubas 2001; Šablevičius 2003a, 2004, 2007; Ferenca et al. 2006, 2007; Cate 2007a, 2009; Inokaitis 2009; Tamutis et al. 2010.

RDB*purpureus* **(Poda, 1761)**. Ferenca 1988, 2003; Gaidienė and Ferenca 1992; Silfverberg 1992, 2004; Pileckis and Monsevičius 1995; Ivinskis et al. 1997b, 2000, 2004a; Tamutis 2005b; Cate 2007a, 2009; Rašomavičius 2007; Vaivilavičius 2008; Alekseev 2010b.

*Aplotarsus* **Stephens, 1830**.

[*incanus* **(Gyllenhal, 1827)**]. Known in Latvia (Telnov 2004), western Belarus (Alexandrovitch et al. 1996), Estonia, Denmark, Sweden (Lundberg and Gustafsson 1995), Poland (Tarnawski and Buchholz 2008b).

*Paraphotistus* **Kishii, 1966** = *Mosotalesus* Kishii, 1977.

*impressus* **(Fabricius, 1792)**. Pileckis 1963b, 1970a, 1976a, b, 1979; Strazdienė 1981; 1988; Silfverberg 1992, 2004; Gaidienė 1993; Pileckis and Monsevičius 1995 (*Selatosomus*); Monsevičius 1997; Žiogas and Zolubas 2005; Ferenca 2006b; Cate 2007a, 2009; Tamutis et al. 2010.

*nigricornis* **(Panzer, 1799)**. Pileckis 1960, 1976a; Strazdienė 1981, 1988; Silfverberg 1992, 2004; Gaidienė 1993; Pileckis and Monsevičius 1995 (*Selatosomus*); Monsevičius 1997; Ferenca 2006b; Cate 2007a, 2009; Tamutis et al. 2010.

*Selatosomus* Stephens, 1830.

*aeneus* **(Linnaeus, 1758)**. Ogijewicz 1929, 1931, 1939; Mazurowa and Mazur 1939; Pileckis 1960, 1976a; Lešinskas and Pileckis 1967; Zajančkauskas and Pileckis 1968; Pileckis and Vengeliauskaitė 1977, 1996; Pileckis et al. 1983, 1994b; Strazdienė 1981, 1988; Silfverberg 1992, 2004; Gaidienė 1993; Pileckis and Monsevičius 1995; Monsevičius 1997; Šablevičius 2000b, 2011; Gliaudys 2001; Tamutis and Zolubas 2001; Ferenca 2006b; Cate 2007a, 2009; Vaivilavičius 2008.

*cruciatus* **(Linnaeus, 1758)**. Eichwald 1830; Heyden 1903; Ogijewicz 1939; Pileckis 1960, 1976a; Lešinskas and Pileckis 1967; Silfverberg 1992, 2004; Gaidienė 1993; Pileckis and Monsevičius 1995; Šablevičius 2000b, 2011; Gliaudys 2001; Tamutis and Zolubas 2001; Ferenca 2006b; Cate 2007a, 2009.

*gravidus* **(Germar, 1843)** = *latus* (Fabricius, 1801) nec (Füssly, 1775). Pileckis 1976a; Strazdienė 1981, 1988; Silfverberg 1992, 2004; Gaidienė 1993; Pileckis and Monsevičius 1995; Cate 2007a, 2009.

[*melancholicus* **(Fabricius, 1798)**].Known in northern Belarus (Alexandrovitch et al. 1996), Sweden (Lundberg and Gustafsson 1995), Poland (Tarnawski and Buchholz 2008b). 

*Hypoganus* **Kiesenwetter, 1858**.

[*inunctus* **(Lacordaire, 1835)** = *cinctus* (Paykull, 1800) nec (Panzer, 1796)].Known in Denmark, southern Sweden and (Lundberg and Gustafsson 1995), Poland (Tarnawski and Buchholz 2008b).

*Calambus* **Thomson, 1859**.

*bipustulatus* **(Linnaeus, 1767)**. Heyden 1903; Pileckis 1960, 1970b, 1976a; Silfverberg 1992, 2004; Gaidienė 1993; Pileckis and Monsevičius 1995; Ivinskis et al.1998b; Ferenca et al. 2002; Ferenca 2004; Cate 2007a, 2009; Vaivilavičius 2008; Tamutis et al.2010.

**Negastriinae** **Nakane & Kishii, 1956**.

**Negastriini Nakane & Kishii, 1956**.

*Negastrius* **Thomson, 1859**.

*arenicola* **(Boheman, 1853)**.Ferenca 2004, 2006a.

*pulchellus* **(Linnaeus, 1758)**. Ogijewicz 1939; Pileckis 1960, 1976a; Bercio and Folwaczny 1979; Silfverberg 1992, 2004; Gaidienė 1993; Pileckis and Monsevičius 1995; Ferenca 2006b; Cate 2007a, 2009; Šablevičius 2011.

*sabulicola* **(Boheman, 1853)**. Bercio and Folwaczny 1979; Silfverberg 1992, 2004; Pileckis and Monsevičius 1995; Ferenca 2004; Cate 2007a, 2009; Šablevičius 2011.

*Zorochros* **Thomson, 1859**.

*dermestoides* **(Herbst, 1806)** = *minimus* (Lacordaire, 1835).Cate 2007a.

*Oedostethus* **LeConte, 1853**.

*quadripustulatus* **(Fabricius, 1792)**. Pileckis 1968a, 1976a; Strazdienė 1976; Bercio and Folwaczny 1979; Silfverberg 1992, 2004; Gaidienė 1993; Pileckis and Monsevičius 1995; Tamutis et al. 2006, 2007; Cate 2007a, 2009; Šablevičius 2011.

*tenuicornis* **(Germar, 1824)**. Pileckis 1976a; Silfverberg 1992, 2004; Gaidienė 1993; Pileckis and Monsevičius 1995; Cate 2007a, 2009.

**Elaterinae** **Leach, 1815**.

**Megapenthini Gurjeva, 1973**.

*Procraerus* **Reitter, 1905**.

*tibialis* **(Lacordaire, 1835)**. Pileckis 1960, 1970a, b, 1976a; Silfverberg 1992, 2004; Gaidienė 1993; Pileckis and Monsevičius 1995; Ferenca 2006b; Cate 2007a, 2009; Tamutis et al. 2010.

**Ampedini Gistel, 1848**.

*Ampedus* **Dejean, 1833** = *Elater* auct. nec Linnaeus, 1758.

*balteatus* **(Linnaeus, 1758)**. Ogijewicz 1939; Pileckis 1960, 1976a; Lešinskas and Pileckis 1967; Zajančkauskas and Pileckis 1968; Strazdienė 1981, 1988; Silfverberg 1992, 2004; Gaidienė 1993; Pileckis and Monsevičius 1995; Monsevičius 1997; Šablevičius 2000b; Gliaudys 2001; Tamutis and Zolubas 2001; Ferenca 2006b; Cate 2007a, 2009; Vaivilavičius 2008.

[*bouweri* **Schimmel, 1984**]. Known in Latvia (Barševskis 2005).

[*cardinalis* **(Schiödte 1865)**].Known in Latvia (Telnov 2004), Denmark, Sweden (Lundberg and Gustafsson 1995), Poland (Cate 2007a, 2009).

*cinnabarinus* **(Eschscholtz, 1829)**. Ogijewicz 1939; Pileckis 1960, 1976a; Lešinskas and Pileckis 1967; Silfverberg 1992, 2004; Gaidienė 1993; Pileckis and Monsevičius 1995; Šablevičius 2000b, 2011; Gliaudys 2001; Tamutis and Zolubas 2001; Ehnström et al. 2003; Cate 2007a, 2009.

*elegantulus* **(Schönherr 1817)**.Ferenca 2004; Vaivilavičius 2008; Inokaitis 2009; Tamutis et al. 2010.

*elongatulus* **(Fabricius, 1787)**. Silfverberg 1992, 2004; Pileckis and Monsevičius 1995; Ferenca 2003; Ehnström et al. 2003; Cate 2007a, 2009; Tamutis et al. 2010.

*erythrogonus* **(O.F. Müller, 1821)**. Ogijewicz 1939; Pileckis 1960, 1976a; Silfverberg 1992, 2004; Pileckis and Monsevičius 1995; Tamutis and Zolubas 2001; Ferenca 2004; Šablevičius 2001, 2004; Cate 2007a, 2009; Vaivilavičius 2008; Ostrauskas and Ferenca 2010.

*hjorti* **(Rye 1905)**.Cate 2007a, 2009.

[*nemoralis* **Bouwer, 1980**].Known in Latvia (Barševskis 2005).

[*nigerrimus* **(Lacordaire, 1835)**].Known in Denmark, southern Sweden (Lundberg and Gustafsson 1995), Poland (Cate 2007a, 2009).

*nigrinus* **(Herbst, 1784)**. Pileckis and Monsevičius 1982, 1995; Silfverberg 1992, 2004; Monsevičius 1997; Tamutis and Zolubas 2001; Ferenca 2004; Cate 2007a, 2009; Tamutis et al. 2010; Ostrauskas and Ferenca 2010.

*nigroflavus* **(Goeze, 1777)**. Silfverberg 1992, 2004; Pileckis and Monsevičius 1995; Ehnström et al. 2003; Ferenca 2003, 2006a; Vaivilavičius 2008; Inokaitis 2009; Tamutis et al. 2010.

*pomonae* **(Stephens, 1830)**. Mazurowa and Mazur 1939; Ogijewicz 1939; Pileckis 1960, 1976a; Zajančkauskas and Pileckis 1968; Strazdienė 1981, 1988; Silfverberg 1992, 2004; Gaidienė 1993; Pileckis and Monsevičius 1995; Monsevičius 1997; Tamutis and Zolubas 2001; Ferenca 2006b; Cate 2007a, 2009.

*pomorum* **(Herbst, 1784)** = *ferrugatus* (Lacordaire, 1835). Heyden 1903; Pileckis 1963b, 1976a; Strazdienė 1981, 1988; Silfverberg 1992, 2004; Gaidienė 1993; Pileckis and Monsevičius 1995; Šablevičius 2000b; Tamutis and Zolubas 2001; Cate 2007a, 2009.

*praeustus* **(Fabricius, 1792)**. Ogijewicz 1939; Pileckis 1960, 1976a; Strazdienė 1981, 1988; Silfverberg 1992, 2004; Gaidienė 1993; Pileckis and Monsevičius 1995; Monsevičius 1997; Cate 2007a, 2009; Tamutis et al. 2010.

[*quercicola* **(Buysson, 1887)**].Known in Denmark (Lundberg and Gustafsson 1995). 

[*rufipennis* **(Stephens, 1830)**].Known in Denmark, southern Sweden (Lundberg and Gustafsson 1995), Poland (Cate 2007a, 2009).

*sanguineus* **(Linnaeus, 1758)**. Ogijewicz 1939; Pileckis 1960, 1976a; Silfverberg 1992, 2004; Gaidienė 1993; Pileckis and Monsevičius 1995; Monsevičius 1997; Ehnström et al. 2003; Ferenca 2006b; Cate 2007a, 2009; Vaivilavičius 2008; Šablevičius 2011.

*sanguinolentus* **(Schrank, 1776)**. Ogijewicz 1939; Pileckis 1960, 1963b, 1976a; Silfverberg 1992, 2004; Gaidienė 1993; Pileckis and Monsevičius 1995; Monsevičius 1997; Šablevičius 2000b; Ehnström et al. 2003; Ferenca 2006b; Cate 2007a, 2009; Inokaitis 2009; Tamutis et al. 2010.

[*suecicus* **Palm, 1976** = *borealis* (Palm, 1947) nec (Paykull, 1800)]. Known in Latvia (Telnov 2004), Belarus (Alexandrovitch et al. 1996), Sweden (Lundberg and Gustafsson 1995), Poland (Cate 2007a, 2009).

[*triangulum* **Dorn, 1925**]. Known in Denmark, southern Sweden (Lundberg and Gustafsson 1995).

*tristis* **(Linnaeus, 1758)**. Silfverberg 1992, 2004; Pileckis and Monsevičius 1995; Ferenca et al. 2002; Tamutis et al. 2010.

[*vandalitiae* **Lohse, 1976**]. Known in Latvia (Barševskis 2005), Poland (Cate 2007a, 2009).

[*ziegleri* **Zeising & Sieg, 1983**].Known in Latvia (Barševskis and Nitcis 2010).

*Brachygonus* **Buysson, 1912** = *Kometsukia* Kishii, 1957.

[*megerlei* **(Lacordaire, 1835)** = *dubius* Platia & Cate, 1990]. Known in southern Sweden (Lundberg and Gustafsson 1995), Poland (Cate 2007a, 2009).

*Ischnodes* **Germar, 1844**.

[*sanguinicollis* **(Panzer, 1793)**]. Known in Denmark, southern Sweden (Lundberg and Gustafsson 1995), Poland (Cate 2007a, 2009).

**Elaterini Leach, 1815**.

*Elater* **Linnaeus, 1758** = *Ludius* Berthold, 1827.

*ferrugineus* **Linnaeus, 1758**. Meržijevskis and Tamutis 2010.

*Sericus* **Eschscholtz, 1829**.

*brunneus* **(Linnaeus, 1758)**. Heyden 1903; Ogijewicz 1939; Pileckis 1960, 1976a; Strazdienė 1988; Silfverberg 1992, 2004; Gaidienė 1993; Pileckis and Monsevičius 1995; Monsevičius 1997; Šablevičius 2000b; Gliaudys 2001; Tamutis and Zolubas 2001; Cate 2007a, 2009.

**Melanotini Candeze, 1859 (1848)** = Cratonychini Gistel, 1856.

*Melanotus* **Eschscholtz, 1829**.

*castanipes* **(Paykull, 1800)**. Pileckis 1976a; Silfverberg 1992, 2004; Pileckis and Monsevičius 1995; Cate 2007a, 2009.

*crassicollis* **(Erichson, 1841)**. Cate 2009.

[*punctolineatus* **(Pelerin, 1829)** = *niger* (Fabricius, 1792) nec (Linnaeus, 1758)].Known in Latvia (Telnov 2004), Denmark, Sweden (Lundberg and Gustafsson 1995), Poland (Cate 2007a, 2009).

*villosus* **(Geoffroy, 1785)** = *erythropus* (Gmelin, 1790) = *rufipes* (Herbst, 1784) nec (Goeze, 1777). Heyden 1903; Ogijewicz 1939; Pileckis 1960, 1976a; Lešinskas and Pileckis 1967; Strazdienė 1981; Silfverberg 1992, 2004; Gaidienė 1993; Pileckis and Monsevičius 1995; Monsevičius 1997; Šablevičius 2000b; Gliaudys 2001; Tamutis and Zolubas 2001; Ferenca 2006b; Cate 2007a, 2009.

**Agriotini Laporte, 1840**.

*Agriotes* **Eschscholtz, 1829**.

*acuminatus* **(Stephens, 1830)**. Pileckis 1960, 1976a; Silfverberg 1992, 2004; Pileckis and Monsevičius 1995; Ferenca 2006b, Cate 2007a, 2009.

*lineatus* **(Linnaeus, 1767)**. Heyden 1903; Ogijewicz 1939; Pileckis 1960, 1976a; Lešinskas and Pileckis 1967; Zajančkauskas and Pileckis 1968; Strazdienė 1976, 1988; Pileckis and Vengeliauskaitė 1977, 1996; Pileckis et al. 1983, 1994b; Silfverberg 1992, 2004; Gaidienė 1993; Pileckis and Monsevičius 1995; Monsevičius 1997; Žiogas 1997; Šablevičius 2000b; Tamutis and Zolubas 2001; Ferenca 2006b; Tamutis et al. 2006, 2007; Cate 2007a, 2009.

*obscurus* **(Linnaeus, 1758)**. Heyden 1903; Ogijewicz 1929, 1931, 1939; Pileckis 1960, 1976a; Lešinskas and Pileckis 1967; Strazdienė 1976, 1988; Pileckis and Vengeliauskaitė 1977, 1996; Silfverberg 1992, 2004; Gaidienė 1993; Pileckis et al. 1994b; Pileckis and Monsevičius 1995; Monsevičius 1997; Žiogas 1997; Gliaudys 2001; Tamutis and Zolubas 2001; Ferenca 2006b; Tamutis et al. 2006, 2007; Cate 2007a, 2009.

[*pilosellus* **(Schönherr, 1817)** = *elongatus* auct. nec (Marsham, 1802)].Known in northwestern Belarus (Alexandrovitch et al. 1996), Denmark, Sweden (Lundberg and Gustafsson 1995), Poland (Cate 2007a, 2009).

*sputator* **(Linnaeus, 1758)**. Ogijewicz 1929, 1931, 1939; Pileckis 1960, 1976a; Lešinskas and Pileckis 1967; Strazdienė 1988; Pileckis and Vengeliauskaitė 1977, 1996; Pileckis et al. 1983, 1994b; Silfverberg 1992, 2004; Gaidienė 1993; Pileckis and Monsevičius 1995; Žiogas 1997; Šablevičius 2000b; Gliaudys 2001; Cate 2007a, 2009.

*ustulatus* **(Schaller, 1783)**. Ogijewicz 1939; Pileckis 1960, 1976a; Silfverberg 1992, 2004; Gaidienė 1993; Pileckis and Monsevičius 1995; Ferenca 2006b; Cate 2007a, 2009.

*Ectinus* **Eschscholtz, 1761**.

*aterrimus* **(Linnaeus, 1761)**. Eichwald 1830; Ogijewicz 1939; Pileckis 1960, 1976a; Strazdienė 1988; Silfverberg 1992, 2004; Gaidienė 1993; Pileckis and Monsevičius 1995; Žiogas and Zolubas 2005; Ferenca 2006b; Cate 2007a, 2009; Vaivilavičius 2008.

*Dalopius* **Eschscholtz, 1829**.

*marginatus* **(Linnaeus, 1758)**. Heyden 1903; Ogijewicz 1939; Pileckis 1960, 1976a; Zajančkauskas and Pileckis 1968; Strazdienė 1976, 1981, 1988; Silfverberg 1992, 2004; Gaidienė 1993; Pileckis and Monsevičius 1995; Monsevičius 1997; Šablevičius 2000b; Tamutis and Zolubas 2001; Ferenca 2006b; Cate 2007a, 2009; Vaivilavičius 2008; Ivinskis et al. 2008; Ostrauskas and Ferenca 2010.

*Adrastus* **Eschscholtz, 1829**.

[*lacertosus* **Erichson, 1841**]. Known in Latvia (Telnov et al. 2005), Poland (Cate 2007a, 2009).

*limbatus* **(Fabricius, 1777)**. Roubal 1910; Pileckis 1960, 1976a; Silfverberg 1992, 2004; Gaidienė 1993; Pileckis and Monsevičius 1995; Cate 2007a, 2009.

*pallens* **(Fabricius, 1792)** = *nitidulus* (Marsham, 1802) nec (Fabricius, 1787). Heyden 1903; Ogijewicz 1939; Pileckis 1960, 1976a; Silfverberg 1992, 2004; Gaidienė 1993; Pileckis and Monsevičius 1995; Monsevičius 1997; Šablevičius 2000b; Žiogas and Zolubas 2005; Cate 2007a, 2009.

*rachifer* **Geoffroy, 1785**. Mazurowa and Mazur 1939; Ogijewicz 1939; Pileckis 1960, 1976a; Silfverberg 1992, 2004; Gaidienė 1993; Pileckis and Monsevičius 1995; Cate 2007a, 2009.

*Synaptus* **Eschscholtz, 1829**.

*filiformis* **Fabricius, 1781**. Heyden 1903; Ogijewicz 1939; Pileckis 1960, 1976a; Silfverberg 1992, 2004; Gaidienė 1993; Pileckis and Monsevičius 1995; Ferenca 2006b, Cate 2007a, 2009.

**Cardiophorinae** **Candèze, 1859**.

*Cardiophorus* **Eschscholtz, 1829**.

*asellus* **Erichson, 1840**.Tamutis 2003; Inokaitis 2009; Tamutis et al. 2010.

[*atramentarius* **Erichson, 1840** = *vestigialis* Erichson, 1840].Known in southern Sweden (Lundberg and Gustafsson 1995), Poland (Cate 2007a, 2009).

*ebeninus* **(Germar, 1824)**.Cate 2007a, 2009.

[*goezei* **Sanchez-Ruiz, 1996** = *rufipes* (Goeze, 1777)]. Known in Latvia (Telnov 2004), southern Sweden (Lundberg and Gustafsson 1995), Poland (Cate 2007a, 2009).

[*gramineus* **(Scopoli, 1763)**]. Known in Latvia (Telnov 2004), southern Sweden (Lundberg and Gustafsson 1995), Kaliningrad region, Poland (Cate 2007a, 2009).

*ruficollis* **(Linnaeus, 1758)**. Ogijewicz 1939; Pileckis 1960, 1976a; Silfverberg 1992, 2004; Gaidienė 1993; Pileckis and Monsevičius 1995; Tamutis and Zolubas 2001; Ferenca 2004, 2006b; Lynikienė and Gedminas 2006; Cate 2007a, 2009.

*Dicronychus* **Brullé, 1832**.

*cinereus* **(Herbst, 1784)**. Ogijewicz 1939; Pileckis 1960, 1976a; Silfverberg 1992, 2004; Gaidienė 1993; Pileckis and Monsevičius 1995; Ferenca 2006b; Cate 2007a, 2009; Tamutis et al. 2010.

*equiseti* **(Herbst, 1784)**. Pileckis 1960, 1976a; Silfverberg 1992, 2004; Gaidienė 1993; Pileckis and Monsevičius 1995; Tamuts & Zolubas 2001; Ferenca 2006b; Cate 2007a, 2009; Tamutis et al. 2010.

[*equisetoides* **Lohse, 1976**].Known in Latvia (Telnov 2004), Denmark, southern Sweden (Lundberg and Gustafsson 1995), Poland (Cate 2007a, 2009).

*Paracardiophorus* **Schwarz, 1895**.

*musculus* **(Erichson, 1840)**. Pileckis 1963b, 1970a, b, 1976a; Silfverberg 1992, 2004; Gaidienė 1993; Pileckis and Monsevičius 1995; Cate 2007a, 2009; Tamutis et al. 2010.

**DRILIDAE Blanchard, 1845**.

**Drilinae** Blanchard, 1845

*Drilus* **Olivier, 1790**.

*concolor* **Ahrens, 1812**. Silfverberg 2004; Ferenca and Tamutis 2009, as expected for Lithuania species noted by Pileckis and Monsevičius (1995).

**LYCIDAE Laporte, 1836**.

**Dictyopterinae** **Houlbert, 1922**.

**Dictyopterini Houlbert, 1922**.

*Dictyoptera* **Latreille, 1829**.

*aurora* **(Herbst, 1784)**. Pileckis 1960, 1963b, 1976a; Silfverberg 1992, 2004; Gaidienė 1993; Pileckis and Monsevičius 1995; Šablevičius 2000b; Ferenca 2006b; Bocáková and Bocák 2007; Bocák 2009.

*Pyropterus* **Mulsant, 1836**.

*nigroruber* **(DeGeer, 1774)** = *affinis* (Paykull, 1799). Pileckis 1960, 1976a; Silfverberg 1992, 2004; Gaidienė 1993; Pileckis and Monsevičius 1995; Ferenca 2006b; Bocáková and Bocák 2007; Bocák 2009; Ostrauskas and Ferenca 2010.

*Platycis* **Thomson, 1859** = *Glabroplatycis* Pic, 1914.

[*cosnardi* **(Chevrolat, 1829)**].Recently found in Latvia (Barševskis et al. 2008) and Kaliningrad region (Alekseev and Bukejs 2010), known in Denmark, southern Sweden (Lundberg and Gustafsson 1995), Poland (Bocák 2009).

*minuta* **(Fabrcius, 1787)**. Pileckis 1962, 1963b, 1976a; Silfverberg 1992, 2004; Gaidienė 1993; Pileckis and Monsevičius 1995; Gliaudys 2001.

*Lopheros* **LeConte, 1884**.

*rubens* **(Gyllenhal, 1817)**. Gaidienė 1993; Silfverberg 2004.

**Lycinae Laporte, 1836**.

**Metriorrhynchini Kleine, 1926**.

*Xylobanellus* **Kleine, 1930** = *Rossioptera* Kasantsev, 1988.

*erythropterus* **(Baudi di Selve, 1871)**.Jakaitis 1985; Pileckis and Monsevičius 1995.

**Calochromini Lacordaire, 1857**.

*Lygistopterus* **Dejean, 1833**.

*sanguineus* **(Linnaeus, 1758)**. Pileckis 1960, 1963b, 1976a; Silfverberg 1992, 2004; Gaidienė 1993; Pileckis and Monsevičius 1995; Monsevičius 1997; Šablevičius 2000b; Gliaudys 2001; Ferenca 2006b; Bocáková and Bocák 2007; Bocák 2009.

**LAMPYRIDAE Latreille, 1817**.

**Lampyrinae Rafinesque, 1815**.

**Lampyrini Rafinesque, 1815**.

*Lampyris* **Geoffroy, 1762**.

*noctiluca* **(Linnaeus, 1758)**. Pileckis 1960, 1976a, 1982; Lešinskas and Pileckis 1967; Zajančkauskas and Pileckis 1968; Silfverberg 1992, 2004; Gaidienė 1993; Pileckis and Monsevičius 1995; Monsevičius 1997; Šablevičius 2000b, 2011; Gliaudys 2001; Žiogas and Zolubas 2005; Ferenca 2006b; Lynikienė and Gedminas 2006; Geisthardt and Satô 2007; Geisthardt 2009.

**Lucidotini Lacordaire, 1857**.

*Phosphaenus* **Laporte, 1833**.

*hemipterus* **(Goeze, 1777)**. Strazdienė 1976; Slavinskas 1982; Silfverberg 1992, 2004; Gaidienė 1993; Pileckis and Monsevičius 1995; Ferenca 2004; Geisthardt and Satô 2007; Vaivilavičius 2008; Geisthardt 2009; Ivinskis et al. 2009.

**CANTHARIDAE Imhoff, 1856 (1815)**.

**Cantharinae** **Imhoff, 1856 (1815)**.

**Podabrini Gistel, 1856**.

*Podabrus* **Westwood, 1838**.

*alpinus* **(Paykull, 1798)**. Heyden 1903; Pileckis 1960, 1976a; Silfverberg 1992, 2004; Gaidienė 1993; Pileckis and Monsevičius 1995; Kazantsev and Brancucci 2007; Vaivilavičius 2008; Kazantsev 2009.

**Cantharini Imhoff, 1856 (1815)**.

*Ancistronycha* **Märkel, 1852**.

*cyanipennis* **(Faldermann, 1835)** = *violacea* (Paykull, 1798) nec (Thunberg, 1784). Silfverberg 1992, 2004; Gaidienė 1993; Pileckis and Monsevičius 1995; Kazantsev and Brancucci 2007; Vaivilavičius 2008.

*Cantharis* **Linnaeus, 1758**.

[*annularis* **Menetriez, 1836** = *oculata* Gebler, 1827]. Known in Denmark (Lundberg and Gustafsson 1995), northern Belarus (Alexandrovitch et al. 1996).

[*cryptica* **Ashe, 1947**]. Known in Denmark, southern Sweden (Lundberg and Gustafsson 1995), Poland (Kazantsev 2009).

[*decipiens* **Baudi, 1871**]. Known in Latvia (Telnov 2004), Denmark, Sweden (Lundberg and Gustafsson 1995), Poland (Kazantsev 2009).

*figurata* **Mannerheim, 1843**. Pileckis 1963b, 1976a; Zajančkauskas and Pileckis 1968; Silfverberg 1992, 2004; Gaidienė 1993; Pileckis and Monsevičius 1995; Monsevičius 1997; Kazantsev and Brancucci 2007; Kazantsev 2009.

*flavilabris* **Fallén, 1807** = *fulvicollis* Fabricius, 1792, nec Scopoli, 1763. Heyden 1903; Pileckis 1960, 1963b, 1976a; Zajančkauskas and Pileckis 1968; Silfverberg 1992, 2004; Gaidienė 1993; Pileckis and Monsevičius 1995; Monsevičius 1997; Žiogas and Zolubas 2005; Ferenca 2006b; Kazantsev and Brancucci 2007 Kazantsev 2009.

*fusca* **Linnaeus, 1758**. Eichwald 1830; Heyden 1903; Roubal 1910; Pileckis 1960, 1976a, 1982; Zajančkauskas and Pileckis 1968; Silfverberg 1992, 2004; Gaidienė 1993; Pileckis and Monsevičius 1995; Monsevičius 1997; Gliaudys 2001; Ferenca 2006b; Tamutis et al. 2007; Kazantsev and Brancucci 2007; Kazantsev 2009.

*lateralis* **Linnaeus, 1758**. Heyden 1903; Pileckis 1960, 1976a; Silfverberg 1992, 2004; Gaidienė 1993; Pileckis and Monsevičius 1995; Ferenca 2006b; Tamutis et al. 2007; Kazantsev and Brancucci 2007; Vaivilavičius 2008; Kazantsev 2009.

*livida* **Linnaeus, 1758**. Eichwald 1830; Heyden 1903; Pileckis 1960, 1963b, 1976a; Strazdienė 1976; Silfverberg 1992, 2004; Gaidienė 1993; Pileckis and Monsevičius 1995; Monsevičius 1997; Šablevičius 2000b; Gliaudys 2001; Ferenca 2006b; Kazantsev and Brancucci 2007; Kazantsev 2009.

*nigra* **(DeGeer, 1774)** = *thoracica* (Olivier, 1790) = *bicolor* Herbst, 1784, nec Linnaeus, 1763. Pileckis and Monsevičius 1982, 1995; Ivinskis et al. 1984; Silfverberg 1992, 2004; Monsevičius 1997; Ivinskis et al. 2009; Ostrauskas and Ferenca 2010.

*nigricans* **(O.F. Müller, 1776)**. Heyden 1903; Pileckis 1960, 1976a; Silfverberg 1992, 2004; Gaidienė 1993; Pileckis and Monsevičius 1995; Kazantsev and Brancucci 2007; Kazantsev 2009; Ostrauskas and Ferenca 2010.

*obscura* **Linnaeus, 1758**. Pileckis 1960, 1976a; Silfverberg 1992, 2004; Gaidienė 1993; Pileckis and Monsevičius 1995; Monsevičius 1997; Šablevičius 2000b; Gliaudys 2001; Ferenca 2006b; Kazantsev and Brancucci 2007; Kazantsev 2009.

*pallida* **Goeze, 1777**. Pileckis and Monsevičius 1982, 1995; Gaidienė 1993; Silfverberg 1992, 2004; Monsevičius 1997; Kazantsev and Brancucci 2007; Dapkus and Tamutis 2007, 2008a.

*paludosa* **Fallén, 1807**. Monsevičius 1997; Silfverberg 2004; Ivinskis et al. 2009.

*pellucida* **Fabricius, 1792**. Pileckis 1960, 1976a; Silfverberg 1992, 2004; Gaidienė 1993; Pileckis and Monsevičius 1995; Monsevičius 1997; Ferenca 2006b; Kazantsev and Brancucci 2007; Kazantsev 2009.

[*pulicaria* **Fabricius, 1781**].Known in Latvia (Telnov 2004), eastern Belarus (Alexandrovitch et al. 1996), Estonia (Silfverberg 2004), Poland (Kazantsev 2009).

*quadripunctata* **(O.F. Müller, 1776)**. Pileckis 1960, 1976a; Silfverberg 1992, 2004; Gaidienė 1993; Pileckis and Monsevičius 1995; Ferenca 2006b; Kazantsev and Brancucci 2007; Kazantsev 2009.

*rufa* **Linnaeus, 1758**. Pileckis 1960, 1976a; Lešinskas and Pileckis 1967; Zajančkauskas and Pileckis 1968; Bercio and Folwaczny 1979; Silfverberg 1992, 2004; Gaidienė 1993; Pileckis and Monsevičius 1995; Monsevičius 1997; Gliaudys 2001; Ferenca 2006b; Tamutis et al. 2007; Kazantsev and Brancucci 2007; Dapkus and Tamutis 2008a; Kazantsev 2009.

*rustica* **Fallén, 1807**. Eichwald 1830; Heyden 1903; Pileckis 1960, 1976a; Lešinskas and Pileckis 1967; Zajančkauskas and Pileckis 1968; Strazdienė 1976; Silfverberg 1992, 2004; Gaidienė 1993; Pileckis and Monsevičius 1995; Monsevičius 1997; Šablevičius 2000b; Gliaudys 2001; Ferenca 2006b; Kazantsev and Brancucci 2007; Vaivilavičius 2008; Kazantsev 2009.

*Metacantharis* **Bourgeois, 1886**.

*clypeata* **(Illiger, 1798)** = *haemorrhoidalis* (Fabricius, 1792) nec (Gmelin, 1790). Heyden 1903; Pileckis 1960, 1976a; Silfverberg 1992, 2004; Gaidienė 1993; Pileckis and Monsevičius 1995; Kazantsev and Brancucci 2007; Kazantsev 2009.

*Rhagonycha* **Eschscholtz, 1830**.

*atra* **(Linnaeus, 1767)**. Bercio and Folwaczny 1979; Monsevičius 1988b, 1997; Silfverberg 1992, 2004; Pileckis and Monsevičius 1995; Kazantsev and Brancucci 2007; Kazantsev 2009.

*elongata* **(Fallén, 1807)**. Pileckis 1960, 1976a; Bercio and Folwaczny 1979; Silfverberg 1992, 2004; Gaidienė 1993; Pileckis and Monsevičius 1995; Ferenca 2006b; Kazantsev and Brancucci 2007; Kazantsev 2009.

*fugax* **Mannerheim, 1843**.Kazantsev and Brancucci 2007; Kazantsev 2009.

*fulva* **(Scopoli, 1763)**. Lešinskas and Pileckis 1967; Pileckis 1968a, 1976a; Strazdienė 1976; Silfverberg 1992, 2004; Gaidienė 1993; Pileckis and Monsevičius 1995; Monsevičius 1997; Šablevičius 2000b; Gliaudys 2001; Kazantsev and Brancucci 2007; Vaivilavičius 2008; Kazantsev 2009.

*lignosa* **(O.F. Müller, 1764)**. Heyden 1903; Pileckis 1960, 1963b, 1976a; Silfverberg 1992, 2004; Gaidienė 1993; Pileckis and Monsevičius 1995; Monsevičius 1997; Žiogas and Zolubas 2005; Kazantsev and Brancucci 2007; Kazantsev 2009.

*limbata* **(Thomson, 1864)**. Heyden 1903; Pileckis 1960, 1976a; Bercio and Folwaczny 1979; Silfverberg 1992, 2004; Gaidienė 1993; Pileckis and Monsevičius 1995; Monsevičius 1997; Ferenca 2006b; Kazantsev and Brancucci 2007; Kazantsev 2009.

*lutea* **(O.F. Müller, 1764)**.Ferenca et al. 2002.

*nigripes* **Redtenbacher, 1842**. Tenenbaum 1923, 1931; Pileckis 1968b, 1976a; Silfverberg 1992, 2004; Pileckis and Monsevičius 1995; Kazantsev and Brancucci 2007; Kazantsev 2009.

*testacea* **(Linnaeus, 1758)**. Heyden 1903; Pileckis 1960, 1976a; Zajančkauskas and Pileckis 1968; Strazdienė 1976; Silfverberg 1992, 2004; Gaidienė 1993; Pileckis and Monsevičius 1995; Monsevičius 1997; Ferenca 2006b; Kazantsev and Brancucci 2007; Kazantsev 2009.

[*translucida* **(Krynicki, 1832)**].Known in western Belarus (Alexandrovitch et al. 1996), Poland (Kazantsev 2009).

*Cratosilis* **Motschulsky, 1860**.

*denticollis **(Schummel, 1844)**. # 58.Roubal 1910; Pileckis 1960.

*Podistra* **Motschulsky, 1839** = *Absidia* Mulsant, 1863.

[*rufotestacea* **(Letzner, 1845)**].Known in Latvia (Telnov 2004), Estonia, Denmark, throughout Sweden (Lundberg and Gustafsson 1995), Belarus (Alexandrovitch et al. 1996), Poland (Kazantsev 2009).

*schoenherri* **(Dejean, 1837)** = *pilosa* (Paykull, 1798) nec (Scopoli, 1763). Pileckis 1976a; Silfverberg 1992, 2004; Gaidienė 1993; Pileckis and Monsevičius 1995; Monsevičius 1997; Kazantsev and Brancucci 2007; Kazantsev 2009; Ivinskis et al. 2009.

**Silinae** **Mulsant, 1862**.

**Silini Mulsant, 1862**.

*Crudosilis* **Kazantsev, 1994**.

*ruficollis* **(Fabricius, 1775)**. Pileckis and Monsevičius 1982, 1995; Ivinskis et al. 1984; Silfverberg 1992, 2004; Gaidienė 1993; Monsevičius 1997; Ferenca et al. 2002; Šablevičius 2004; Kazantsev and Brancucci 2007; Ivinskis et al. 2009.

*Silis* **Charpentier, 1825**.

*nitidula* **(Fabricius, 1792)**. Pileckis 1960, 1976a; Silfverberg 1992, 2004; Pileckis and Monsevičius 1995; Ferenca et al. 2002; Šablevičius 2004; Ferenca 2006b; Kazantsev and Brancucci 2007.

**Malthininae** **Kiesenwetter, 1852** = MalthodinaeBöving & Craighead, 1931.

**Malthinini Kiesenwetter, 1852**.

*Malthinus* **Latreille, 1806**.

[*balteatus* **Suffrian, 1851**].Known in Latvia (Telnov 2004), southern Sweden (Lundberg and Gustafsson 1995).

*biguttatus* **(Linnaeus, 1758)** = *biguttulus* (Paykull, 1800). Pileckis 1976a; Silfverberg 1992, 2004; Gaidienė 1993; Pileckis and Monsevičius 1995; Ferenca 2004; Ivinskis et al. 2009; Ostrauskas and Ferenca 2010.

*facialis* **Thomson, 1864**. Pileckis 1976a; Silfverberg 1992, 2004; Pileckis and Monsevičius 1995.

[*frontalis* **(Marsham, 1802)**].Known in Latvia (Telnov 2004), Estonia, Denmark, Sweden (Lundberg and Gustafsson 1995).

*flaveolus* **(Herbst, 1786)** = *punctatus* (Geoffroy, 1785). Roubal 1910; Pileckis 1960, 1963b; 1976a; Silfverberg 1992, 2004; Gaidienė 1993; Pileckis and Monsevičius 1995; Monsevičius 1997; Ferenca 2004, 2006b; Kazantsev and Brancucci 2007; Kazantsev 2009.

[*seriepunctatus* **Kiesenwetter, 1851**].Known in southern Sweden (Lundberg and Gustafsson 1995), Belarus (Alexandrovitch et al. 1996).

**Malthodini Böving & Craighead, 1931**.

*Malthodes* **Kiesenwetter, 1852**.

*brevicollis* **(Paykull, 1798)**. Pileckis 1976a; Bercio and Folwaczny 1979; Silfverberg 1992, 2004; Pileckis and Monsevičius 1995.

[*crassicornis* **(Mäklin, 1846)**].Known in Latvia (Telnov 2004), Estonia (Süda 2009), Denmark, Sweden (Lundberg and Gustafsson 1995), Belarus (Alexandrovitch et al. 1996).

[*debilis* **Kiesenwetter, 1852**].Known in Latvia (Telnov 2004), Poland (Kazantsev 2009).

[*dimidiatocollis* **(Rosenhauer, 1847)**].Known in Latvia (Telnov 2004).

[*dispar* **(Germar, 1824)**].Known in Denmark, southern Sweden (Lundberg and Gustafsson 1995), Poland (Kazantsev 2009).

[*europaeus* **Wittmer, 1970**].Known in Latvia (Telnov 2004), Poland (Kazantsev 2009).

*fibulatus* **Kiesenwetter, 1852**.Tamutis 2003.

*flavoguttatus* **Kiesenwetter, 1824**. Pileckis 1960, 1976a; Silfverberg 1992, 2004; Pileckis and Monsevičius 1995; Ferenca 2006b.

[*fuscus* **(Waltl, 1838)**].Known in Latvia (Telnov 2004), Denmark, Estonia, throughout Sweden (Lundberg and Gustafsson 1995).

*guttifer* **Kiesenwetter, 1852**. Pileckis and Monsevičius 1982, 1995; Silfverberg 1992, 2004; Monsevičius 1997.

*marginatus* **(Latreille, 1806)**. Pileckis 1976a; Bercio and Folwaczny 1979; Silfverberg 1992, 2004; Pileckis and Monsevičius 1995.

[*maurus* **(Laporte, 1840)**].Known in Latvia (Telnov 2004), Denmark, Estonia, throughout Sweden (Lundberg and Gustafsson 1995).

*minimus* **(Linnaeus, 1758)**. Pileckis 1976a; Silfverberg 1992, 2004; Pileckis and Monsevičius 1995; Ferenca 2004.

[*mysticus* **Kiesenwetter, 1852**].Known in Estonia, Denmark, throughout Sweden (Lundberg and Gustafsson 1995).

*pumilus* **(Brébisson, 1835)**. Bercio and Folwaczny 1979; Silfverberg 1992, 2004; Ferenca 2004.

[*spathifer* **Kiesenwetter, 1852**].Known in Latvia (Telnov 2004), Denmark, Estonia, Sweden (Lundberg and Gustafsson 1995).

**DERODONTOIDEA** **LeConte, 1861**.

**DERODONTIDAE LeConte, 1861**.

**Laricobiinae Mulsant & Rey, 1864**.

*Laricobius* **Rosenhauer, 1846**.

[*erichsoni* **Rosenhauer, 1846**].Known in Denmark, southern Sweden (Lundberg and Gustafsson 1995).

**NOSODENDRIDAE Erichson, 1846**.

*Nosodendron* **Latreille, 1804**.

*fasciculare* **(Olivier, 1790)**. Pileckis and Monsevičius 1995; Silfverberg 2004; Ferenca et al. 2006, 2007; Steinhammer 2007.

**BOSTRICHOIDEA** **Latreille 1802**.

**DERMESTIDAE Latreille, 1804**.

**Dermestinae** **Latreille, 1804**.

**Dermestini Latreille, 1804**.

*Dermestes* **Linnaeus, 1758**.

*ater* **DeGeer, 1774** = *cadaverinus* Fabricius, 1775. Pileckis 1960, 1976a; Silfverberg 1992, 2004; Pileckis and Monsevičius 1995; Háva and Löbl 2007.

[*bicolor* **Fabricius, 1781**]. Known in Latvia (Telnov et al. 2005), northern Poland (Burakowski et al. 1986a).

[*erichsoni* **Ganglbauer, 1904** = *tessellatus* Erichson, 1846, nec Fabricius, 1775]. Known in Latvia (Telnov 2004).

*frischi* **Kugelann, 1792**. Bercio and Folwaczny 1979; Silfverberg 1992, 2004; Gaidienė 1993; Pileckis and Monsevičius 1995; Háva and Löbl 2007.

*gyllenhali* **Laporte, 1840** = *atomarius* Erichson, 1846. Pileckis 1976a; Bercio and Folwaczny 1979; Silfverberg 1992, 2004; Gaidienė 1993; Pileckis and Monsevičius 1995; Ferenca 2004; Háva and Löbl 2007; Zhantiev 2009.

*haemorrhoidalis* **Küster, 1852**].Known in Denmark, Sweden (Lundberg and Gustafsson 1995), Poland (Burakowski et al . 1986a).

*laniarius* **Illiger, 1801**. Pileckis 1968a, 1976a; Silfverberg 1992, 2004; Pileckis and Monsevičius 1995; Háva and Löbl 2007; Zhantiev 2009.

*lardarius* **Linnaeus, 1758**. Pileckis 1960, 1976a, 1998; Lešinskas and Pileckis 1967; Silfverberg 1992, 2004; Gaidienė 1993; Pileckis and Monsevičius 1995; Šablevičius 2000b, 2011; Gliaudys 2001; Ferenca 2006b; Háva and Löbl 2007; Zhantiev 2009.

*maculatus* **DeGeer, 1774** = *vulpinus* Fabricius, 1781. Monsevičius 1988b; Ferenca 1988; Gaidienė 1993; Silfverberg 1992, 2004; Pileckis and Monsevičius 1995; Ferenca et al. 2002; Háva and Löbl 2007; Zhantiev 2009.

*murinus* **Linnaeus, 1758**. Pileckis 1960, 1976a; Bercio and Folwaczny 1979; Silfverberg 1992, 2004; Gaidienė 1993; Pileckis and Monsevičius 1995; Šablevičius 2000b, 2011; Gliaudys 2001; Žiogas and Zolubas 2005; Ferenca 2006b; Háva and Löbl 2007; Zhantiev 2009.

[*peruvianus* **Laporte, 1840**].Known in Sweden (Lundberg and Gustafsson 1995), Poland (Burakowski et al. 1986a).

[*szekessyi* **Kalik, 1950**].Known in Denmark, southern Sweden (Lundberg and Gustafsson 1995), Belarus (Zhantiev 2009), Poland (Burakowski et al. 1986a).

*undulatus* **Brahm, 1790**.Tamutis 2003; Háva and Löbl 2007; Ivinskis et al. 2009.

**Trinodinae** **Casey, 1900**.

**Trinodini Casey, 1900**.

*Trinodes* **Dejean, 1821**.

*hirtus* **(Fabricius, 1781)**. Háva and Löbl 2007.

**Thylodriini Semenov, 1909**.

*Thylodrias* **Motschulsky, 1839**.

[*contractus* **Motschulsky, 1839**]. Known in Denmark, Sweden (Lundberg and Gustafsson 1995).

**Attageninae** **Laporte, 1840**.

**Attagenini Laporte, 1840**.

*Attagenus* **Latreille, 1802**.

[*brunneus* **Faldermann, 1835**]. Known in Latvia (Telnov 2004).

[*pantherinus* **(Ahrens, 1814)**].Known in northeastern Poland (Burakowski et al. 1986a).

*pellio* **(Linnaeus, 1758)**. Heyden 1903; Pileckis 1960, 1976a, 1998; Lešinskas and Pileckis 1967; Silfverberg 1992, 2004; Gaidienė 1993; Pileckis and Monsevičius 1995; Gliaudys 2001; Ferenca 2006b; Háva and Löbl 2007; Zhantiev 2009; Šablevičius 2011.

[*punctatus* **(Scopoli, 1772)**].Known in northern Poland (Burakowski et al. 1986a).

*schaefferi* **(Herbst, 1792)**. Pileckis 1976a; Silfverberg 1992, 2004; Pileckis and Monsevičius 1995; Háva and Löbl 2007; Zhantiev 2009.

*smirnovi* **Zhantiev, 1973**. Barševskis 2009.

*unicolor* **(Brahm, 1791)** = *piceus* (Olivier, 1790) nec (Thunberg, 1781) = *megatoma* (Fabricius, 1798). Heyden 1903; Pileckis 1960, 1963b; 1976a; Silfverberg 1992, 2004; Gaidienė 1993; Pileckis and Monsevičius 1995; Monsevičius 1997; Háva and Löbl 2007; Zhantiev 2009.

[*woodroffei* **Halstead & Green, 1979** = *fasciatus* auct. nec (Thunberg, 1795) = *gloriosae* auct. nec (Fabricius, 1798)]. Known in Denmark, Sweden (Lundberg and Gustafsson 1995).

**Megatominae** **Leach, 1815**.

**Megatomini Leach, 1815**.

*Trogoderma* **Dejean, 1821**.

*angustum* **(Solier, 1849)**. Šablevičius 2004, 2007, 2011; Ferenca et al. 2006, 2007; Tamutis and Ferenca 2006.

*glabrum* **(Herbst, 1783)**. Pileckis and Monsevičius 1995; Silfverberg 2004; Zhantiev 2009.

[*granarium* **Everts, 1898**]. Known in Denmark, Sweden (Lundberg and Gustafsson 1995), Poland (Burakowski et al. 1986a).

[*megatomoides* **Reitter, 1881**]. Known in western Belarus (Alexandrovitch et al. 1996), Sweden (Lindberg 1995).

*variabile* **Ballion, 1878**. Silfverberg 2004; Zhantiev 2009.

*versicolor* **(Creutzer, 1799)**. Pileckis 1984; Silfverberg 1992, 2004; Pileckis and Monsevičius 1995.

*Reesa* **Beal, 1967**.

[*vespulae* **(Milliron, 1939)**]. Known in Denmark, Sweden (Lundberg and Gustafsson 1995), Latvia (Telnov et al. 2005), Estonia (Süda 2009), Kaliningrad region (Alekseev and Nikitsky 2008).

*Globicornis* **Latreille, 1829**.

*corticalis* **(Eichoff, 1863)**.Tamutis and Ferenca 2006; Ferenca et al. 2006, 2007.

*emarginata* **(Gyllenhal, 1808)** = *marginata* (Paykull, 1798) nec (Thunberg, 1781). Silfverberg 1992, 2004; Pileckis and Monsevičius 1995; Šablevičius 2000b; Háva and Löbl 2007; Zhantiev 2009.

[*nigripes* **(Fabricius, 1792)** = *rufitarsis* (Panzer, 1796)]. Known in Denmark, southern Sweden (Lundberg and Gustafsson 1995), Belarus (Zhantiev 2009), Poland (Burakowski et al. 1986a).

*Megatoma* **Herbst, 1792**.

[*graeseri* **(Reitter, 1887)**]. Known in Latvia (Telnov 2010). 

*pubescens* **(Zetterstedt, 1828)**. Pileckis and Monsevičius 1982; 1995; Silfverberg 1992, 2004.

*undata* **(Linnaeus, 1758)**. Pileckis 1960, 1963b; 1976a; Silfverberg 1992, 2004; Gaidienė 1993; Pileckis and Monsevičius 1995; Gliaudys 2001; Tamutis and Zolubas 2001; Žiogas and Zolubas 2005; Ferenca 2006b; Háva and Löbl 2007; Zhantiev 2009; Šablevičius 2011.

*Ctesias* **Stephens, 1830**.

*serra* **(Fabricius, 1792)**. Bercio and Folwaczny 1979; Silfverberg 1992, 2004; Pileckis and Monsevičius 1995; Háva and Löbl 2007; Zhantiev 2009.

**Anthrenini Gistel, 1848**.

*Anthrenus* **Geoffroy, 1762**.

[*caucasicus* **Reitter, 1881**]. Known in Latvia (Telnov 2010).

[*flavipes* **LeConte, 1854**]. Known in Denmark (Lundberg and Gustafsson 1995), Poland (Zhantiev 2009).

*fuscus* **(Olivier, 1789)**. Pileckis 1960, 1976a; Bercio and Folwaczny 1979; Silfverberg 1992, 2004; Gaidienė 1993; Pileckis and Monsevičius 1995; Ferenca 2006b; Háva and Löbl 2007; Zhantiev 2009.

*museorum* **(Linnaeus, 1761)**. Pileckis 1960, 1976a; Silfverberg 1992, 2004; Gaidienė 1993; Pileckis and Monsevičius 1995; Monsevičius 1997; Šablevičius 2000b, 2011; Gliaudys 2001; Háva and Löbl 2007; Zhantiev 2009; Ostrauskas and Ferenca 2010.

[*olgae* **Kalik, 1946**].Known in Latvia (Telnov 2004), Sweden (Lundberg and Gustafsson 1995), Poland (Burakowski et al. 1986a).

*pimpinellae* **Fabricius, 1775**. Roubal 1910; Pileckis 1960, 1976a; Silfverberg 1992, 2004; Pileckis and Monsevičius 1995; Háva and Löbl 2007.

*polonicus* **Mroczkowski, 1951**. Miländer et al. 1984; Silfverberg 1992, 2004; Pileckis and Monsevičius 1995; Monsevičius 1997.

*scrophulariae* **(Linnaeus, 1758)**. Eichwald 1830; Heyden 1903; Pileckis 1960, 1976a, 1998; Lešinskas and Pileckis 1967; Bercio and Folwaczny 1979; Silfverberg 1992, 2004; Gaidienė 1993; Pileckis and Monsevičius 1995; Monsevičius 1997; Šablevičius 2000b, 2011; Ferenca 2006b; Háva and Löbl 2007; Zhantiev 2009.

*verbasci* **(Linnaeus, 1767)**. Eichwald 1830; Roubal 1910; Pileckis 1960, 1976a; Silfverberg 1992, 2004; Pileckis and Monsevičius 1995; Háva and Löbl 2007.

**BOSTRICHIDAE Latreille, 1802**.

**Bostrichinae** **Latreille 1802**.

**Bostrichini Latreille 1802**.

*Bostrichus* **Geoffroy, 1762**.

*capucinus* **(Linnaeus, 1758)**.Eichwald 1830; Lešinskas and Pileckis 1967; Borowski 2007.

**Dinoderinae** **Thomson, 1863**.

*Stephanopachys* **Waterhouse, 1888**.

*linearis* **(Kugelann, 1792)**. Pileckis and Monsevičius 1995; Lundberg and Gustafsson 1995; Silfverberg 2004; Borowski 2007.

*substriatus* **(Paykull, 1800)**. Borowski 2007; Nardi 2009.

*Rhyzopertha* **Stephens, 1830**.

*dominica* **(Fabricius, 1793)**. Tamutis 2003.

*Dinoderus* **Stephens, 1830**.

[*minutus* **(Fabricius, 1775)**].Known in Sweden (Lundberg and Gustafsson 1995), Poland (Burakowski et al. 1986a).

**Lyctinae** **Billberg, 1820**.

**Lyctini Billberg, 1820**.

*Lyctus* **Fabricius, 1792**.

*africanus* **Lesne, 1907**.Ivinskis et al. 2009.

[*brunneus* **(Stephens, 1830)**].Known in Denmark, Sweden (Lundberg and Gustafsson 1995), Poland (Burakowski et al. 1986a).

*linearis* **(Goeze, 1777)**. Pileckis 1960, 1976a; Silfverberg 1992, 2004; Pileckis and Monsevičius 1995; Ferenca 2006b; Borowski 2007; Nardi 2009.

[*pubescens* **Panzer, 1793**].Known in northern Poland (Burakowski et al. 1986a).

*Minthea* **Pascoe, 1863**.

[*rugicollis* **(Walker, 1858)**].Known in Denmark, Sweden (Lundberg and Gustafsson 1995).

**Trogoxylini Lesne, 1921**.

*Trogoxylon* **LeConte, 1862**.

[*impressum* **(Comolli, 1837)**].Known in Denmark, Sweden (Lundberg and Gustafsson 1995).

**PTINIDAE Latreille, 1802**.

**Eucradinae** **LeConte, 1861**.

**Eucradini LeConte, 1861**.

*Ptinomorphus* **Mulsant & Rey, 1868**.

[*imperialis* **(Linnaeus, 1767)**].Known in Latvia (Telnov 2004), Kaliningrad region (Alekseev and Bukejs 2010), Estonia, Denmark, Sweden (Lundberg and Gustafsson 1995), Poland (Burakowski et al. 1986a).

**Ptininae** **Latreille, 1802**.

**Gibbiini Jacquelin du Val, 1860**.

*Gibbium* **Scopoli, 1777**.

[*psylloides* **(Czempinski, 1778)**].Known in Denmark, Sweden, Estonia (Silfverberg 2004), Poland (Burakowski et al. 1986a).

**Meziini Bellés, 1985**.

*Mezium* **Curtis, 1828**.

[*affine* **Boieldieu, 1856**].Known in Denmark (Lundberg and Gustafsson 1995), Sweden (Silfverberg 2004), Poland (Zahradník 2009).

[*sulcatum* **(Fabricius, 1781)**].Known in Sweden (Lundberg and Gustafsson 1995), Denmark (Silfverberg 2004).

*Trigonogenius* **Solier, 1849**.

[*globosus* **Solier, 1849**].Known in Denmark, Sweden (Lundberg and Gustafsson 1995), Poland (Burakowski et al. 1986a).

**Sphaericini Portevin, 1931**.

*Sphaericus* **Wollaston, 1854**.

[*gibboides* **(Boieldieu, 1854)**].Known in Denmark (Lundberg and Gustafsson 1995), Sweden (Zahradník 2009), Poland (Burakowski et al. 1986a).

**Ptinini Latreille, 1802**.

*Niptus* **Boieldieu, 1856**.

*hololeucus* **(Faldermann, 1836)**. Mastauskis 1925; Pileckis 1960, 1963b, 1976a, 1998; Lešinskas and Pileckis 1967; Zubrys 1967; Pileckis and Vengeliauskaitė 1977, 1996; Silfverberg 1992, 2004; Gaidienė 1993; Pileckis et al. 1994b; Pileckis and Monsevičius 1995; Ferenca 2006b; Borowski and Zahradník 2007; Zahradník 2009.

*Epauloecus* **Mulsant & Rey, 1868** = *Tipnus* Thomson, 1859, nec Boieldieu, 1856.

*unicolor* **(Piller & Mitterpacher, 1783)**. Pileckis and Monsevičius 1995; Silfverberg 2004; Borowski and Zahradník 2007; Zahradník 2009.

*Pseudeurostus* **Heyden, 1906**.

[*hilleri* **(Reitter, 1877)**]. Known in Denmark, Sweden (Lundberg and Gustafsson 1995).

*Ptinus* **Linnaeus, 1767**.

[*bicinctus* **Sturm, 1837**].Known in Latvia (Telnov 2004), Belarus (Alexandrovitch et al. 1996), Estonia, Denmark Sweden (Lundberg and Gustafsson 1995), northern Poland (Burakowski et al. 1986a).

*clavipes* **Panzer, 1792** = *testaceus* Olivier, 1790, nec Thunberg, 1784 = *latro* Fabricius, 1775. Silfverberg 1992, 2004; Gaidienė 1993; Pileckis and Monsevičius 1995; Tamutis 2003; Žiogas and Zolubas 2005; Borowski and Zahradník 2007; Zahradník 2009; Ostrauskas and Ferenca 2010.

*coarcticollis* **Sturm, 1837**.Borowski and Zahradník 2007; Zahradník 2009; Ivinskis et al. 2009.

*dubius* **Sturm, 1837**.Borowski and Zahradník 2007; Zahradník 2009.

*fur* **(Linnaeus, 1758)**. Ogyjewicz 1934; Pileckis 1960, 1976a, 1998; Lešinskas and Pileckis 1967; Zubrys 1967; Pileckis and Vengeliauskaitė 1977, 1996; Silfverberg 1992, 2004; Gaidienė 1993; Pileckis et al. 1994b; Pileckis and Monsevičius 1995; Monsevičius 1997; Šablevičius 2000b; Ferenca 2006b; Borowski and Zahradník 2007; Zahradník 2009.

*lichenum* **Marsham, 1802**.Borowski and Zahradník 2007.

*pilosus* **O.F. Müller, 1821**. Pileckis and Monsevičius 1995; Silfverberg 2004.

*podolicus* **Iablokoff-Khnzorian & Karapetyan, 1991**.Borowski and Zahradník 2007; Zahradník 2009.

[*pusillus* **Sturm, 1837**].Known in Denmark (Lundberg and Gustafsson 1995), Sweden (Zahradník 2009), northern Poland (Burakowski et al. 1986a).

*raptor* **Sturm, 1837**. Pileckis 1960, 1963b, 1976a; Zubrys 1967; Silfverberg 1992, 2004; Pileckis and Monsevičius 1995; Monsevičius 1997; Borowski and Zahradník 2007; Zahradník 2009.

*rufipes* **Olivier, 1789**. Roubal 1910; Pileckis 1960, 1976a; Zubrys 1967; Silfverberg 1992, 2004; Gaidienė 1993; Pileckis and Monsevičius 1995; Borowski and Zahradník 2007; Zahradník 2009.

[*sexpunctatus* **Panzer, 1795**].Known in Latvia (Telnov 2004), Belarus (Alexandrovitch et al. 1996), Denmark, southern Sweden (Lundberg and Gustafsson 1995), Poland (Burakowski et al. 1986a).

*subpilosus* **Sturm, 1837**. Silfverberg 1992, 2004; Pileckis and Monsevičius 1995; Ferenca et al. 2002; Borowski and Zahradník 2007; Zahradník 2009.

*tectus* **Boieldieu, 1856**. Borowski and Zahradník 2007; Zahradník 2009.

[*variegatus* **Rossi, 1792**]. Known in northern Poland (Burakowski et al. 1986a).

*villiger* **Reitter, 1884** = *balticus* Iablokoff-Khnzorian & Karapetian, 1991. Gaidienė 1993; Silfverberg 2004; Borowski and Zahradník 2007; Zahradník 2009.

**Dryophilinae** **Gistel, 1848**.

**Dryophilini Gistel, 1848**.

*Grynobius* **Thomson, 1859**.

[*planus* **(Fabricius, 1787)** = *tricolor* (Olivier, 1790)].Known in Latvia (Telnov et al. 2006), Denmark, southern Sweden (Lundberg and Gustafsson 1995), Poland (Burakowski et al. 1986a).

*Dryophilus* **Chevrolat, 1832**.

*pusillus* **(Gyllenhal, 1808)**. Bercio and Folwaczny 1979; Silfverberg 1992, 2004; Pileckis and Monsevičius 1995; Borowski and Zahradník 2007; Zahradník 2009; Ostrauskas and Ferenca 2010.

**Ernobiinae** **Pic, 1912**.

*Ochina* **Sturm, 1826**.

*ptinoides* **(Marsham, 1802)**. Silfverberg 1992, 2004; Pileckis and Monsevičius 1995; Borowski and Zahradník 2007; Zahradník 2009.

*Xestobium* **Motschulsky, 1845**.

[*plumbeum* **(Illiger, 1801)**]. Known in Belarus (Alexandrovitch et al. 1996), Denmark (Lundberg and Gustafsson 1995), Poland (Burakowski et al. 1986a).

RDB*rufovillosum* **(DeGeer, 1774)**. Ferenca et al. 2002; Ehnström et al. 2003; Ferenca 2004; Ivinskis et al. 2004b, 2007; Butvila et al. 2007; Rašomavičius 2007; Borowski and Zahradník 2007; Vaivilavičius 2008; Zahradník 2009; Alekseev 2010b.

*Ernobius* **Thomson, 1859**.

*abietinus* **(Gyllenhal, 1808)**. Ferenca et al. 2002, 2006, 2007; Tamutis and Ferenca 2006; Ivinskis et al. 2009.

*abietis* **(Fabricius, 1792)**. Pileckis 1959, 1960, 1976a; Pileckis et al. 1968; Milišauskas 1976, 2000; Silfverberg 1992, 2004; Gaidienė 1993; Pileckis and Monsevičius 1995; Žiogas 1997; Gliaudys 2001; Borowski and Zahradník 2007; Zahradník 2009.

*angusticollis* **(Ratzeburg, 1847)** = *tabidus* Kiesenwetter, 1877. Pileckis 1959, 1960, 1976a; Milišauskas 1976, 2000; Silfverberg 1992, 2004; Pileckis and Monsevičius 1995; Borowski and Zahradník 2007; Zahradník 2009.

[*explanatus* **(Mannerheim, 1843)**]. Known in Latvia (Telnov 2004), northern Belarus (Alexandrovitch et al. 1996), Estonia, Sweden (Lundberg and Gustafsson 1995), Poland (Burakowski et al. 1986a).

*gigas* **(Mulsant & Rey, 1843)**. Silfverberg 1992, 2004; Pileckis and Monsevičius 1995; Borowski and Zahradník 2007; Zahradník 2009.

*kiesenwetteri* **(Schilsky, 1898)**. Silfverberg 1992, 2004; Pileckis and Monsevičius 1995; Borowski and Zahradník 2007; Zahradník 2009.

*longicornis* **(Sturm, 1837)**. Pileckis 1959, 1960, 1976a; Milišauskas 1976; Bercio and Folwaczny 1979; Silfverberg 1992, 2004; Pileckis and Monsevičius 1995; Borowski and Zahradník 2007; Zahradník 2009.

*mollis* **(Linnaeus, 1758)**. Pileckis 1960, 1976a; Silfverberg 1992, 2004; Gaidienė 1993; Pileckis and Monsevičius 1995; Ferenca 2006b; Borowski and Zahradník 2007; Zahradník 2009; Ostrauskas and Ferenca 2010.

[*mulsanti* **Kiesenwetter, 1877**].Known in northeastern Poland (Burakowski et al. 1986a).

*nigrinus* **(Sturm, 1837)**. Silfverberg 1992, 2004; Pileckis and Monsevičius 1995; Borowski and Zahradník 2007; Zahradník 2009.

*pini* **(Sturm, 1837)**. Tamutis 2003.

**Anobiinae** **Fleming, 1821**.

*Oligomerus* **Redtenbacher, 1849**.

*brunneus* **(Olivier, 1790)**. Pileckis 1960, 1976a; Bercio and Folwaczny 1979; Silfverberg 1992, 2004; Gaidienė 1993; Pileckis and Monsevičius 1995; Ferenca 2006b; Borowski and Zahradník 2007; Zahradník 2009.

*Stegobium* **Motschulsky, 1860** = *Sitodrepa* Thomson, 1863.

*paniceum* **(Linnaeus, 1758)**. Heyden 1903; Mastauskis 1927; Pileckis 1960, 1970a, 1976a, 1998; Lešinskas and Pileckis 1967; Zubrys 1967; Pileckis and Vengeliauskaitė 1977, 1996; Silfverberg 1992, 2004; Gaidienė 1993 Pileckis et al. 1994b; Pileckis and Monsevičius 1995; Gliaudys 2001; Borowski and Zahradník 2007; Zahradník 2009.

*Gastralus* **Jacquelin du Val, 1860**.

[*immarginatus* **(O.F. Müller, 1821)**].Known in Latvia (Telnov 2004), Denmark, Sweden (Lundberg and Gustafsson 1995), Poland (Burakowski et al. 1986a).

*Anobium* **Fabricius, 1775** = *Hemicoelus* LeConte, 1861.

[*costatum* **Aragona, 1830**]. Known in Denmark, southern Sweden (Lundberg and Gustafsson 1995), Poland (Burakowski et al. 1986a).

*fulvicorne* **Sturm, 1837**. Pileckis and Monsevičius 1995; Silfverberg 2004.

*nitidum* **Fabricius, 1792**. Ostrauskas and Ferenca 2010.

*punctatum* **(DeGeer, 1774)** = *domesticum* (Geoffroy, 1785). Heyden 1903; Pileckis 1960, 1976a, 1998; Lešinskas and Pileckis 1967; Pileckis and Vengeliauskaitė 1977, 1996; Silfverberg 1992, 2004; Gaidienė 1993; Pileckis and Monsevičius 1995; Žiogas 1997, 2000b; Gliaudys 2001; Borowski and Zahradník 2007; Zahradník 2009.

*rufipes* **Fabricius, 1792**. Monsevičius 1988b; Silfverberg 1992, 2004; Gaidienė 1993; Pileckis and Monsevičius 1995; Borowski and Zahradník 2007; Vaivilavičius 2008.

*thomsoni* **(Kraatz, 1881)**. Silfverberg 2004.

*Microbregma* **Seidlitz, 1889**.

*emarginata* **(Duftschmid, 1825)**. Pileckis et al. 1968; Pileckis 1963b, 1976a; Pileckis and Monsevičius 1995; Silfverberg 1992, 2004; Borowski and Zahradník 2007; Zahradník 2009; Ostrauskas and Ferenca 2010.

*Hadrobregmus* **Thomson, 1859**.

[*confusus* **(Kraatz, 1881)**]. Known in Latvia (Telnov 2004), Sweden (Lundberg and Gustafsson 1995), Poland (Zahradník 2009).

*denticollis* **(Creutzer, 1796)**. Pileckis and Monsevičius 1982, 1995; Silfverberg 1992, 2004; Borowski and Zahradník 2007; Zahradník 2009.

*pertinax* **(Linnaeus, 1758)**. Heyden 1903; Pileckis 1960, 1963b, 1976a, 1998; Lešinskas and Pileckis 1967; Silfverberg 1992, 2004; Gaidienė 1993; Pileckis and Monsevičius 1995; Žiogas 1997, 2000b; Gliaudys 2001; Tamutis and Zolubas 2001; Borowski and Zahradník 2007; Zahradník 2009.

*Priobium* **Motschulsky, 1845**.

*carpini* **(Herbst, 1793)**. Pileckis 1960, 1976a; Silfverberg 1992, 2004; Gaidienė 1993; Pileckis and Monsevičius 1995; Monsevičius 1997; Ferenca 2006b; Borowski and Zahradník 2007; Zahradník 2009.

**Ptilininae** **Shuckard, 1840**.

*Ptilinus* **Geoffroy, 1762**.

*fuscus* **Geoffroy, 1785**. Ferenca et al. 2002.

*pectinicornis* **(Linnaeus, 1758)**. Pileckis 1960, 1976a; Silfverberg 1992, 2004; Pileckis and Monsevičius 1995; Ferenca 2004; Ferenca 2006b; Butvila et al. 2007; Borowski and Zahradník 2007; Zahradník 2009.

*Pseudoptilinus* **Leiler, 1963**.

[*fissicollis* **(Reitter, 1877)**]. Known in Sweden (Lundberg and Gustafsson 1995), Poland (Burakowski et al. 1986a).

**Xyletininae** **Gistel, 1848**.

**Xyletinini Gistel, 1848**.

*Xyletinus* **Latreille, 1809**.

*ater* **(Creutzer, 1796)**. Lentz 1879; Pileckis 1976a; Bercio and Folwaczny 1979; Pileckis and Monsevičius 1995; Silfverberg 1992, 2004; Borowski and Zahradník 2007; Zahradník 2009.

[*fibyensis* **Lundblad, 1949** = *gronblomi* Y.Kangas, 1955].Known in Latvia (Telnov 2004), Belarus (Borowski and Zahradník 2007; Zahradník 2009), Sweden (Lundberg and Gustafsson 1995), Poland (Burakowski et al. 1986a).

[*hanseni* **Jansson, 1947**].Known in Latvia (Telnov et al. 2006), Estonia (Süda 2009), Denmark, Sweden (Lundberg and Gustafsson 1995).

*laticollis* **(Duftschmid, 1825)**. Bercio and Folwaczny 1979; Silfverberg 1992, 2004; Borowski and Zahradník 2007; Zahradník 2009.

[*longitarsis* **Jansson, 1942**].Known in Latvia (Telnov 2004), Belarus (Alexandrovitch et al. 1996), Denmark (Zahradník 2009), Sweden (Lundberg and Gustafsson 1995).

*pectinatus* **(Fabricius, 1792)**. Tamutis 2003; Borowski and Zahradník 2007; Zahradník 2009.

[*planicollis* **Lohse, 1957** = *suecicus* Lundblad, 1972]. Known in Latvia (Telnov 2004), Sweden (Lundberg and Gustafsson 1995), Poland (Zahradník 2009).

[*tremulicola* **Y. Kangas, 1958**]. Known in Sweden (Lundberg and Gustafsson 1995), Estonia (Süda 2009).

[*vaederoeensis* **Lundberg and Gustafsson, 1969**]. Known in Sweden (Lundberg and Gustafsson 1995).

**Lasiodermini Böving, 1927**.

*Lasioderma* **Stephens, 1835**.

[*serricorne* **(Fabricius, 1792)**].Known in Latvia (Telnov 2004), Estonia, Denmark, Sweden (Lundberg and Gustafsson 1995), Poland (Burakowski et al. 1986a).

**Dorcatominae** **Thomson, 1859**.

*Stagetus* **Wollaston, 1861** = *Theca* Mulsant & Rey, 1860, nec Morris, 1845.

[*borealis* **Israelson, 1971** = *pilulus* auct. nec (Aubé, 1861)]. Known in Latvia (Telnov 2004), Kalinigrad region (Alekseev and Bukejs 2010), Estonia (Süda 2009), Sweden (Lundberg and Gustafsson 1995).

*Dorcatoma* **Herbst, 1792**.

[*ambjoerni* **Baranowski, 1985**].Known in southern Sweden (Baranowski 1985).

*chrysomelina* **Sturm, 1837**. Pileckis 1976a; Bercio and Folwaczny 1979; Silfverberg 1992, 2004; Pileckis and Monsevičius 1995; Borowski and Zahradník 2007; Zahradník 2009.

*dresdensis* **Herbst, 1792**. Pileckis 1976a; Bercio and Folwaczny 1979; Silfverberg 1992, 2004; Pileckis and Monsevičius 1995; Monsevičius 1998; Ferenca 2004; Šablevičius 2004; Ferenca et al. 2006, 2007; Butvila et al. 2007; Borowski and Zahradník 2007; Vaivilavičius 2008; Zahradník 2009; Ivinskis et al. 2009; Ostrauskas and Ferenca 2010.

*flavicornis* **(Fabricius, 1793)**. Bercio and Folwaczny 1979; Silfverberg 1992, 2004; Pileckis and Monsevičius 1995; Borowski and Zahradník 2007; Zahradník 2009.

[*janssoni* **Büche & Lundberg and Gustafsson, 2002**].Known in Latvia, Sweden, Poland (Büche and Lundberg and Gustafsson 2002).

*lomnickii* **Reitter, 1903**.Pileckis 1968b, 1976a; Pileckis and Monsevičius 1995; Ferenca et al. 2006, 2007; Ostrauskas and Ferenca 2010.

[*minor* **Zahradnik, 1993**].Known in Sweden (Silfverberg 2004).

[*punctulata* **Mulsant & Rey, 1864**].Known in Latvia (Telnov 2004), Estonia (Silfverberg 2004), Sweden (Lundberg and Gustafsson 1995), Poland (Burakowski et al. 1986a).

[*robusta* **Strand, 1938**].Known in Latvia (Telnov 2004), Estonia (Silfverberg 2004), Belarus (Alexandrovitch et al. 1996), Sweden (Lundberg and Gustafsson 1995), Poland (Burakowski et al. 1986a).

[*substriata* **Hummel, 1829** = *serra* (Panzer, 1795) nec (Fabricius, 1792)]. Known in Latvia (Telnov 2004), Estonia (Silfverberg 2004), Denmark, Sweden (Lundberg and Gustafsson 1995), Belarus (Alexandrovitch et al. 1996), Poland (Burakowski et al. 1986a).

*Caenocara* **Thomson, 1859**.

*affinis* **Sturm, 1837**. Pileckis and Monsevičius 1982, 1995; Silfverberg 1992, 2004; Monsevičius 1997; Borowski and Zahradník 2007; Zahradník 2009.

*bovistae* **(Hoffmann, 1803)**. Pileckis 1976a; Bercio and Folwaczny 1979; Silfverberg 1992, 2004; Pileckis and Monsevičius 1995; Borowski and Zahradník 2007; Zahradník 2009.

[*subglobosa* **(Mulsant & Rey, 1864)**]. Known in Estonia (Süda 2009), Belarus (Alexandrovitch et al. 1996), northern Poland (Burakowski et al. 1986a).

*Anitys* **Thomson, 1863**.

[*rubens* **(Hoffmann, 1803)**]. Known in Latvia (Telnov 2004), Denmark, southern Sweden (Lundberg and Gustafsson 1995), northern Poland (Burakowski et al. 1986a).

**LYMEXYLOIDEA Fleming, 1821**.

**LYMEXYLIDAE Fleming, 1821**.

**Hyleocoetinae** **Germar, 1818**.

*Hyleocoetus* **Latreille, 1806** = *Elateroides* Houlbert & Bétis, 1905.

*dermestoides* **(Linnaeus, 1761)**. Mastauskis 1953; Pileckis 1960, 1976a; Pileckis et al. 1968; Zajančkauskas and Pileckis 1968; Silfverberg 1992, 2004; Gaidienė 1993; Pileckis and Monsevičius 1995; Monsevičius 1997; Žiogas 1997, 2000b; Valenta 2000b; Tamutis and Zolubas 2001; Ferenca 2006b; Cuccodoro 2007; Audisio 2009; Šablevičius 2011.

*flabellicornis* **(Schneider, 1791)**. Pileckis 1963b, 1976a; Pileckis et al. 1968; Silfverberg 1992, 2004; Gaidienė 1993; Pileckis and Monsevičius 1995; Žiogas 2000b; Cuccodoro 2007; Audisio 2009.

**Lymexylinae** **Fleming, 1821**.

*Lymexylon* **Fabricius, 1775**.

*navale* **(Linnaeus, 1758)**. Pileckis 1968b, 1976a; Silfverberg 1992, 2004; Pileckis and Monsevičius 1995; Ehnström et al. 2003; Butvila et al. 2007; Cuccodoro 2007; Vaivilavičius 2008; Audisio 2009; Inokaitis 2009; Šablevičius 2011.

**CLEROIDEA** **Latreille, 1802**.

**PHLOEOPHILIDAE Kiesenwetter, 1863**.

*Phloeophilus* **Stephens, 1830**.

[*edwardsii* **Stephens, 1830**]. Known in Denmark, southern Sweden (Lundberg and Gustafsson 1995), northern Poland (Burakowski et al. 1986a).

**TROGOSSITIDAE Latreille, 1802**.

**Peltinae** **Latreille, 1806**.

**Peltini Latreille, 1806**.

*Peltis* **Kugelann, 1792**.

RDB*grossa* **(Linnaeus, 1758)**. Pileckis and Jakaitis 1982; Balevičius 1992; Silfverberg 1992, 2004; Gaidienė 1993; Auglys 1994; Šablevičius 1994, 2000, 2001, 2003a, b; Pileckis and Monsevičius 1995; Šablevičius and Ferenca 1995; Ivinskis et al. 1996a, b, 1999, 2000, 2004a, 2007, 2009; Monsevičius 1997, 1998; Pankevičius 2000, 2007; Šablevičius 2000a, b, 2011; Gliaudys 2001; Ehnström et al. 2003; Ferenca 2003, 2004; Meržijevskis 2004; Inokaitis 2004; Tamutis 2005b; Rašomavičius 2007; Masiulis 2007; Leonavičius 2007; Lopeta 2007a; Butvila et al. 2007; Uselis et al. 2007; Kriaučiūnienė and Zaplatkin 2007; Kolibáč 2007, 2009; Vaivilavičius 2008; Dapkus and Tamutis 2008b; Švitra and Aliukonis 2009; Bačianskas 2009; Noreika; Alekseev 2010b.

*Ostoma* **Laicharting, 1781**.

RDB*ferruginea* **(Linnaeus, 1758)**. Pileckis 1960, 1976a; Lešinskas and Pileckis 1967; Silfverberg 1992, 2004; Gaidienė 1993; Pileckis and Monsevičius 1995; Monsevičius 1997; Tamutis and Zolubas 2001; Ehnström et al. 2003; Ferenca 2004, 2006b; Tamutis 2005b; Rašomavičius 2007; Butvila et al. 2007; Ivinskis et al. 2007, 2009; Lopeta 2007a; Uselis et al. 2007; Kolibáč 2007, 2009; Vaivilavičius 2008; Dapkus and Tamutis 2008b; Alekseev 2010b; Šablevičius 2011.

**Thymalini Léveillé, 1888**.

*Thymalus* **Latreille, 1802**.

*limbatus* **(Fabricius, 1787)**. Pileckis 1976a; Silfverberg 1992, 2004; Pileckis and Monsevičius 1995; Kolibáč 2007, 2009.

**Lopchocaterini Crowson, 1964.**

*Grynocharis* **Thomson, 1859**.

*oblonga* **(Linnaeus, 1758)**. Pileckis 1960, 1963b, 1976a; Silfverberg 1992, 2004; Gaidienė 1993; Pileckis and Monsevičius 1995; Šablevičius 2003a, 2011; Ferenca 2004; Ferenca et al. 2007; Kolibáč 2007, 2009; Vaivilavičius 2008; Ivinskis et al. 2009.

**Trogossitinae** **Latreille, 1802**.

**Calytini Reitter, 1922**.

*Calitys* **Thomson, 1859**.

*scabra* **(Thunberg, 1784)**. Pileckis and Monsevičius 1995; Silfverberg 2004; Kolibáč 2007, 2009.

**Trogossitini Latreille, 1802**.

*Nemozoma* **Latreille, 1804**.

*elongatum* **(Linnaeus, 1761)**. Valenta and Jakaitis 1972; Jakaitis 1973; Pileckis 1976a; Silfverberg 1992, 2004; Pileckis and Monsevičius 1995; Gedminas 2005; Butvila et al. 2007; Kolibáč 2007, 2009; Zeniauskas and Gedminas 2010.

*Temnoschila* **Westwood, 1835**.

[*caerulea* **(Olivier, 1790)**]. Known in Sweden (Lundberg and Gustafsson 1995), northern Poland (Burakowski et al. 1986a).

*Tenebroides* **Piller & Mitterpacher, 1783** = *Trogossita* Olivier, 1790.

[*fuscus* **(Goeze, 1777)**]. Known in Belarus (Alexandrovitch et al. 1996), northern Poland (Burakowski et al. 1986a), Estonia (Kolibáč 2007, 2009).

*mauritanicus* **(Linnaeus, 1758)**. Ogyjewicz 1934; Pileckis 1960, 1976a, 1998; Lešinskas and Pileckis 1967; Zubrys 1967; Silfverberg 1992, 2004; Gaidienė 1993; Pileckis et al. 1994b; Pileckis and Monsevičius 1995; Kolibáč 2007, 2009.

**CLERIDAE Latreille, 1802**.

**Tillinae** **Fischer von Waldheim, 1813**.

*Tillus* **Olivier, 1790**.

*elongatus* **(Linnaeus, 1758)**. Pileckis 1960, 1976a; Silfverberg 1992, 2004; Gaidienė 1993; Pileckis and Monsevičius 1995; Ferenca 2004, 2006b; Butvila et al. 2007; Löbl et al. 2007; Vaivilavičius 2008; Ivinskis et al. 2009, Inokaitis 2009.

**Clerinae** **Ltreille, 1802**.

*Opilo* **Latreille, 1802**.

[*domesticus* **(Sturm, 1837)**].Known in Denmark, southern Sweden (Lundberg and Gustafsson 1995), northern Poland (Burakowski et al. 1986a).

*mollis* **(Linnaeus, 1758)**.Löbl et al. 2007.

[*pallidus* **Olivier, 1795**]. Known in northern Poland (Burakowski et al. 1986a).

*Thanasimus* **Latreille, 1806**.

*femoralis* **(Zetterstedt, 1828)** = *rufipes* (Brahm, 1797) nec (DeGeer, 1775). Silfverberg 1992, 2004; Pileckis and Monsevičius 1995; Gliaudys 2001; Tamutis and Zolubas 2001; Löbl et al. 2007; Ostrauskas and Ferenca 2010.

*formicarius* **(Linnaeus, 1758)**. Pileckis 1960, 1976a, 1982; Lešinskas and Pileckis 1967; Valenta and Jakaitis 1972; Silfverberg 1992, 2004; Gaidienė 1993; Pileckis and Monsevičius 1995; Monsevičius 1997; Šablevičius 2000b, 2011; Gliaudys 2001; Tamutis and Zolubas 2001; Ferenca 2004; Gedminas 2005; Ferenca 2006b; Löbl et al. 2007; Zeniauskas and Gedminas 2010.

*Trichodes* **Herbst, 1792**.

[*alvearius* **(Fabricius, 1792)**]. Known in northern Poland (Burakowski et al. 1986a).

*apiarius* **(Linnaeus, 1758)**. Pileckis 1960, 1976a, 1982; Lešinskas and Pileckis 1967; Zajančkauskas and Pileckis 1968; Silfverberg 1992, 2004; Gaidienė 1993; Pileckis and Monsevičius 1995; Monsevičius 1997; Šablevičius 2000b, 2011; Gliaudys 2001; Tamutis and Zolubas 2001; Ferenca 2006b; Löbl et al. 2007.

**Korynetinae** **Laporte, 1836**.

*Tarsostenus* **Spinola 1844**.

[*univittatus* **(Rossi, 1792)**]. Known in northern Poland (Burakowski et al. 1986a).

*Korynetes* **Herbst, 1792**.

*caeruleus* **(DeGeer, 1775)**. Slavinskas 1982; Silfverberg 1992, 2004; Gaidienė 1993; Pileckis and Monsevičius 1995; Ostrauskas and Ferenca 2010.

*Necrobia* **Olivier, 1795**.

*ruficollis* **(Fabricius, 1775)**. Pileckis 1968a, 1976a; Silfverberg 1992, 2004; Pileckis and Monsevičius 1995; Löbl et al. 2007.

*rufipes* **(DeGeer, 1775)**. Pileckis 1960, 1970a, 1976a; Gaidienė 1993; Pileckis and Monsevičius 1995; Silfverberg 1992, 2004; Šablevičius 2004; Butvila et al. 2007; Löbl et al. 2007.

*violacea* **(Linnaeus, 1758)**. Pileckis 1960, 1970a, 1976a; Silfverberg 1992, 2004; Gaidienė 1993; Pileckis and Monsevičius 1995; Ferenca 2006b; Löbl et al. 2007.

*Opetiopalpus* **Spinola, 1844**.

*scutellaris* **(Panzer, 1797)**. Pileckis and Monsevičius 1995; Silfverberg 2004; Löbl et al. 2007.

**MELYRIDAE Leach, 1815.**

**Rhadalinae LeConte, 1861**.

*Aplocnemus* **Stephens, 1830**.

*impressus* **(Marsham, 1802)** = *pini* (Redtenbacher, 1849).Mayor 2007a; Liberti 2009.

*nigricornis* **(Fabricius, 1793)**. Pileckis 1976a; Silfverberg 1992, 2004; Pileckis and Monsevičius 1995; Mayor 2007a; Liberti 2009; Ivinskis et al. 2009; Ostrauskas and Ferenca 2010.

*tarsalis* **(C.R. Sahlberg, 1822)**. Pileckis 1976a; Silfverberg 1992, 2004; Pileckis and Monsevičius 1995; Ferenca 2003; Mayor 2007a; Liberti 2009.

*Trichoceble* **Thomson, 1859**.

*floralis* **(Olivier, 1790)**. Pileckis 1976a; Bercio and Folwaczny 1979; Silfverberg 1992, 2004; Pileckis and Monsevičius 1995; Mayor 2007a; Liberti 2009.

*memmonia* **(Kiesenwetter, 1861)**. Bercio and Folwaczny 1979; Silfverberg 1992, 2004; Pileckis and Monsevičius 1995; Mayor 2007a; Liberti 2009.

**Dasytinae** **Laporte, 1840**.

**Danaceini Thomson, 1859**.

*Danacea* **Laporte, 1836**.

[*nigritarsis* **(Küster, 1850)**].Known in southern Sweden (Lundberg and Gustafsson 1995), Poland (Burakowski et al. 1986a).

[*pallipes* **(Panzer, 1793)**].Known in Belarus (Alexandrovitch et al. 1996), Estonia, southern Sweden (Lundberg and Gustafsson 1995), northern Poland (Burakowski et al. 1986a).

**Dasytini Laporte, 1840**.

*Dasytes* **Paykull, 1799**.

[*aeratus* **Stephens, 1830** = *aerosus* Kiesenwetter, 1867].Known in Denmark, Estonia, southern Sweden, (Lundberg and Gustafsson 1995), Poland (Burakowski et al. 1986a).

[*alpigradus* **(Kiesenwetter, 1863)**].Known in Estonia (Lundberg and Gustafsson 1995), Poland (Burakowski et al. 1986a).

*cyaneus* **(Fabricius, 1775)** = *caeruleus* (DeGeer, 1774) nec (Linnaeus, 1758). Pileckis 1976a; Silfverberg 1992, 2004; Pileckis and Monsevičius 1995; Mayor 2007b; Liberti 2009; Ostrauskas and Ferenca 2010.

*fusculus* **(Illiger, 1801)**. Pileckis 1976a; Silfverberg 1992, 2004; Gaidienė 1993; Pileckis and Monsevičius 1995; Mayor 2007b; Liberti 2009.

*niger* **(Linnaeus, 1761)**. Heyden 1903; Roubal 1910; Mazurowa and Mazur 1939; Pileckis 1960, 1976a; Zajančkauskas and Pileckis 1968; Silfverberg 1992, 2004; Gaidienė 1993; Pileckis and Monsevičius 1995; Monsevičius 1997; Šablevičius 2000b; Tamutis and Zolubas 2001; Gedminas 2005; Gedminas et al. 2007; Mayor 2007b; Liberti 2009; Ostrauskas and Ferenca 2010.

[*nigrocyaneus* **Mulsant & Rey, 1868**]. Known in Denmark, southern Sweden (Lundberg and Gustafsson 1995), Poland (Burakowski et al. 1986a).

*obscurus* **Gyllenhal, 1813**.Mayor 2007b; Liberti 2009.

*plumbeus* **(O.F. Müller, 1776)**. Heyden 1903; Pileckis 1960, 1976a; Silfverberg 1992, 2004; Gaidienė 1993; Pileckis and Monsevičius 1995; Tamutis and Zolubas 2001; Žiogas and Zolubas 2005; Mayor 2007b; Vaivilavičius 2008; Liberti 2009; Ostrauskas and Ferenca 2010.

[*subaeneus* **Schönherr, 1817**].Known in Latvia (Telnov 2004), Denmark (Silfverberg 2004), Poland (Burakowski et al. 1986a).

[*virens* **(Marsham, 1802)** = *flavipes* (Olivier, 1790) nec (Fabricius, 1781)].Known in Latvia (Telnov 2004), Estonia (Lundberg and Gustafsson 1995), Poland (Burakowski et al. 1986a).

*Psilothrix* **Redtenbacher, 1858**.

[*viridicoerulea* **(Geoffroy, 1785)** = *cyaneus* auct. nec (Fabricius, 1775)].Known in Latvia, Denmark, southern Sweden (Lundberg and Gustafsson 1995; Mayor 2007b; Liberti 2009).

*Dolichosoma* **Stephens, 1730**.

*lineare* (**P. Rossi, 1792)**. Pileckis 1960, 1976a; Bercio and Folwaczny 1979; Silfverberg 1992, 2004; Gaidienė 1993; Pileckis and Monsevičius 1995; Monsevičius 1997; Šablevičius 2000b; Ferenca 2006b; Mayor 2007b; Liberti 2009.

**Malachiinae** **Fleming, 1821**.

**Malachiini Fleming, 1821**

*Hypebaeus* **Kiesenwetter, 1863**.

[*flavipes* **(Fabricius 1797)**]. Known in Estonia (Süda 2009), southern Sweden (Lundberg and Gustafsson 1995), Kaliningrad region (Alekseev and Nikitsky 2008), Poland (Burakowski et al. 1986a).

*Charopus* **Erichson, 1840**.

*graminicola* **(Dejean, 1833)** = *flavipes* (Paykull, 1798) nec (Fabricius, 1787). Pileckis 1976a; Bercio and Folwaczny 1979; Silfverberg 1992, 2004; Pileckis and Monsevičius 1995; Ferenca 2004; Alekseev 2008a.

*Ebaeus* Erichson, 1840.

*pedicularius* **(Fabricius, 1777)** = *praeoccupatus* Gemminger, 1870 = *lapplandicus* (Evers, 1993). Heyden 1903; Mazurowa and Mazur 1939; Pileckis 1960, 1976a; Silfverberg 1992, 2004; Gaidienė 1993; Pileckis and Monsevičius 1995.

*Nepachys* **Thomson, 1859**.

[*cardiacae* **(Linnaeus, 1761)**]. Known in Latvia (Telnov 2004), Estonia, Sweden (Lundberg and Gustafsson 1995), western Belarus (Alexandrovitch et al. 1996), Poland (Burakowski et al. 1986a).

*Axinotarsus* Motschulsky, 1854.

*marginalis* **(Laporte, 1840)**. Roubal 1910; Pileckis 1960, 1976a; Silfverberg 1992, 2004; Gaidienė 1993; Pileckis and Monsevičius 1995.

*pulicarius* **(Fabricius, 1775)**. Pileckis 1962, 1963b, 1976a; Silfverberg 1992, 2004; Gaidienė 1993; Pileckis and Monsevičius 1995; Ostrauskas and Ferenca 2010.

[*ruficollis* **(Olivier, 1790)**]. Known in Denmark, southern Sweden (Lundberg and Gustafsson 1995), northern Poland (Burakowski et al. 1986a).

*Cordylepherus* **Evers, 1985**.

*viridis* **(Fabricius, 1787)**. Pileckis 1960, 1976a; Silfverberg 1992, 2004; Pileckis and Monsevičius 1995; Mayor 2007c.

*Malachius* **Fabricius, 1775**.

*aeneus* **(Linnaeus, 1758)**. Heyden 1903; Pileckis 1960, 1976a, 1982; Lešinskas and Pileckis 1967; Zajančkauskas and Pileckis 1968; Silfverberg 1992, 2004; Gaidienė 1993; Pileckis and Monsevičius 1995; Monsevičius 1997; Šablevičius 2000b; Ferenca 2006b; Mayor 2007c.

*bipustulatus* **(Linnaeus, 1758)**. Pileckis 1960, 1976a; Zajančkauskas and Pileckis 1968; Bercio and Folwaczny 1979; Silfverberg 1992, 2004; Gaidienė 1993; Pileckis and Monsevičius 1995; Monsevičius 1997; Šablevičius 2000b; Ferenca 2006b; Mayor 2007c.

*rubidus* **Erichson, 1840**. Tenenbaum 1931; Pileckis 1968b, 1976a; Gaidienė 1993; Pileckis and Monsevičius 1995; Silfverberg 2004; Mayor 2007c.

*scutellaris* **Erichson, 1840**. Pileckis 1960, 1976a; Zajančkauskas and Pileckis 1968; Pileckis and Monsevičius 1995; Monsevičius 1997; Silfverberg 2004; Ferenca 2006b; Mayor 2007c.

*Clanoptilus* **Motschulsky, 1854**.

[*barnevillei* **(Puton, 1865)**].Known in Denmark, southern Sweden (Lundberg and Gustafsson 1995).

*marginellus* **(Olivier, 1790)**. Pileckis 1960, 1976a; Zajančkauskas and Pileckis 1968; Silfverberg 1992, 2004; Gaidienė 1993; Pileckis and Monsevičius 1995; Monsevičius 1997; Mayor 2007c.

*Anthocomus* **Erichson, 1840**.

*equestris* **(Fabricius, 1781)** = *bipunctatus* (Harrer, 1784). Heyden 1903; Pileckis 1960, 1963b, 1976a; Gaidienė 1993; Silfverberg 1992, 2004; Pileckis and Monsevičius 1995; Ferenca 2006b; Mayor 2007c.

*fasciatus* **(Linnaeus, 1758)**. Pileckis 1960, 1963b, 1976a; Bercio and Folwaczny 1979; Silfverberg 1992, 2004; Gaidienė 1993; Pileckis and Monsevičius 1995; Monsevičius 1997; Šablevičius 2000b; Ferenca 2006b; Mayor 2007c.

*rufus* **(Herbst, 1784)** = *coccineus* (Schaller, 1783) nec (Linnaeus, 1761). Pileckis 1963b, 1976a; Zajančkauskas and Pileckis 1968; Silfverberg 1992, 2004; Gaidienė 1993; Pileckis and Monsevičius 1995; Monsevičius 1997; Šablevičius 2003a; Ferenca et al. 2006, 2007; Mayor 2007c.

*Cerapheles* **Mulsant & Rey, 1867**.

*terminatus* **(Ménétries, 1832)**. Pileckis and Monsevičius 1995.

*Paratinus* **Abeille de Perrin, 1891**.

*femoralis* **Erichson, 1840**. Lentz 1879; Pileckis 1968b, 1976a; Bercio and Folwaczny 1979; Silfverberg 1992, 2004; Gaidienė 1993; Pileckis and Monsevičius 1995; Šablevičius 2003a; Ferenca 2004; Ferenca et al. 2006, 2007; Mayor 2007c; Alekseev 2008a.

**>CUCUJOIDEA Latreille, 1802**.

**BYTURIDAE Gistel, 1848**.

**Byturinae Gistel, 1848**.

*Byturus* **Latreille, 1796**.

*ochraceus* **(Scriba, 1791)** = *aestivus* auct. nec (Linnaeus, 1758). Heyden 1903; Pileckis 1960, 1976a; Silfverberg 1992, 2004; Gaidienė 1993; Pileckis and Monsevičius 1997; Tamutis and Zolubas 2001; Žiogas and Zolubas 2005; Löbl 2007b; Jelínek 2009.

*tomentosus* **(DeGeer, 1774)**. Heyden 1903; Ogijewicz 1929, 1931, 1932; Pileckis 1960, 1976a; Lešinskas and Pileckis 1967; Pileckis and Vengeliauskaitė 1977, 1996; Silfverberg 1992, 2004; Gaidienė 1993; Pileckis et al. 1994a; Pileckis and Monsevičius 1997; Gliaudys 2001; Ferenca 2006b; Löbl 2007b; Jelínek 2009.

**SPHINDIDAE Jacquelin du Val, 1860**.

**Sphindinae** **Jacquelin du Val, 1860**.

*Sphindus* **Megerle, 1821**.

*dubius* **(Gyllenhal, 1808)**. Pileckis 1976a; Bercio and Folwaczny 1979; Silfverberg 1992, 2004; Pileckis and Monsevičius 1997; Jelínek 2007a, 2009.

*Aspidiphorus* **Ziegler, 1821**.

*orbiculatus* **(Gyllenhal, 1808)**. Monsevičius 1988b, 1997; Silfverberg 1992, 2004; Pileckis and Monsevičius 1997; Šablevičius 2000a, b, 2003a; Jelínek 2007a, 2009.

**BIPHYLLIDAE LeConte, 1861**.

*Biphyllus* **Dejean, 1821**.

[*lunatus* **(Fabricius, 1792)**].Known in Belarus (Alexandrovitch et al. 1996), Sweden (Lundberg and Gustafsson1995), Denmark (Wegrzynowicz 2007c, 2009), northern Poland (Burakowski et al. 1986c).

*Diplocoelus* **Guérin-Ménéville, 1844**.

[*fagi* **Guérin-Ménéville, 1844**]. Known in Belarus (Alexandrovitch et al. 1996), Denmark, Sweden (Lundberg and Gustafsson1995), Kaliningrad region (Bercio and Folwaczny 1979), northern Poland (Burakowski et al. 1986c).

**EROTYLIDAE Latreille, 1802**.

**Xenoscelinae** **Ganglbauer, 1899**.

*Zavaljus* **Reitter, 1880** = *Eicolyctus* J.R. Sahlberg, 1919.

[*brunneus* **(Gyllenhal, 1808)** = *fausti* (Reitter, 1880)]. Known in Latvia (Telnov 2004), Sweden (Lundberg and Gustafsson 1995).

**Erotylinae** **Latreille, 1802**.

**Dacnini Gistel, 1848**.

*Dacne* **Latreille, 1796**.

*bipustulata* **(Thunberg, 1781)**. Mazurowa and Mazur 1939; Pileckis 1960, 1963b; 1976a; Bercio and Folwaczny 1979; Silfverberg 1992, 2004; Gaidienė 1993; Monsevičius 1997; Pileckis and Monsevičius 1997; Šablevičius 2000b, 2011; Gliaudys 2001; Tamutis and Zolubas 2001; Alekseev 2008a; Wegrzynowicz 2009.

[*notata* **(Gmelin, 1790)**]. Known in Estonia (Lundberg and Gustafsson 1995), northern Poland (Burakowski et al. 1986c), Kaliningrad region (Bercio and Folwaczny 1979), northwestern Belarus (Alexandrovitch et al. 1996).

[*rufifrons* **(Fabricius, 1775)**]. Known in Denmark, southern Sweden (Lundberg and Gustafsson 1995), northern Poland (Burakowski et al. 1986c).

*Combocerus* **Bedel, 1867**.

*glaber* **(Schaller, 1783)**. Silfverberg 1992, 2004; Pileckis and Monsevičius 1997; Wegrzynowicz 2007c, 2009.

**Tritomini Curtis, 1834**.

*Tritoma* **Fabricius, 1775**.

*bipustulata* **(Fabricius, 1775)**. Pileckis and Monsevičius 1997; Šablevičius 2001, 2003a; Silfverberg 2004; Wegrzynowicz 2007c.

*subbasalis* **(Reitter, 1896)** = *jakowlewi* (Semenow, 1898) = *consobrina* Lewis, 1874. Pileckis 1968a, 1976a; Gaidienė 1993; Silfverberg 1992, 2004; Pileckis and Monsevičius 1997; Šablevičius 2000a, 2001, 2011; Gedminas 2005; Ferenca 2006b; Gedminas et al. 2007; Vaivilavičius 2008; Wegrzynowicz 2007c, 2009; Ivinskis et al. 2009.

*Triplax* **Herbst, 1793**.

*aenea* **(Schaller, 1783)**. Gaidienė and Ferenca 1988; Silfverberg 1992, 2004; Gaidienė 1993; Pileckis and Monsevičius 1997; Wegrzynowicz 2007c, 2009; Ostrauskas and Ferenca 2010; Šablevičius 2011.

[*lepida* **(Faldermann, 1837)**]. Known in Belarus (Alexandrovitch et al. 1996), Sweden (Wegrzynowicz 2007c, 2009), Poland (Burakowski et al. 1986c).

*rufipes* **(Fabricius, 1781)**. Pileckis 1976a; Silfverberg 1992, 2004; Gaidienė 1993; Monsevičius 1997; Pileckis and Monsevičius 1997; Šablevičius 2001, 2003a, b; Wegrzynowicz 2007c, 2009.

*russica* **(Linnaeus, 1758)**. Lešinskas and Pileckis 1967; Pileckis and Monsevičius 1982, 1997; Silfverberg 1992, 2004; Gaidienė 1993; Monsevičius 1997; Šablevičius 2000b, 2011; Wegrzynowicz 2007c, 2009; Ivinskis et al. 2009.

*scutellaris* **Charpentier, 1825**. Pileckis 1976a; Silfverberg 1992, 2004; Pileckis and Monsevičius 1997; Šablevičius 2001, 2003a; Wegrzynowicz 2007c, 2009.

**MONOTOMIDAE Laporte, 1840**.

**Rhizophaginae Redtenbacher, 1845**.

*Rhizophagus* **Herbst, 1793**. (Rhizophagidae)

*aeneus* **Richter, 1820**. Heyden 1903; Pileckis 1960, 1976a; Silfverberg 1992, 2004; Pileckis and Monsevičius 1997; Ferenca 2004; Jelínek 2007d; Jelínek and Audisio 2009.

*bipustulatus* **(Fabricius, 1798)**. Pileckis 1976a; Jakaitis and Valenta 1976; Silfverberg 1992, 2004; Gaidienė 1993; Monsevičius 1997; Pileckis and Monsevičius 1997; Šablevičius 2000b; Ferenca 2006b; Jelínek 2007d; Jelínek and Audisio 2009.

*cribratus* **Gyllenhal, 1827**. Valenta and Jakaitis 1972; Jakaitis 1973; Silfverberg 1992, 2004; Pileckis and Monsevičius 1997; Jelínek 2007d; Jelínek and Audisio 2009.

*depressus* **(Fabricius, 1793)**. Valenta and Jakaitis 1972; Silfverberg 1992, 2004; Gaidienė 1993; Monsevičius 1997; Pileckis and Monsevičius 1997; Tamutis and Zolubas 2001; Jelínek 2007d; Jelínek and Audisio 2009.

*dispar* **(Paykull, 1800)**. Valenta and Jakaitis 1972; Jakaitis 1973; Pileckis 1976a; Bercio and Folwaczny 1979; Silfverberg 1992, 2004; Gaidienė 1993; Monsevičius 1997; Pileckis and Monsevičius 1997; Jelínek 2007d; Jelínek and Audisio 2009; Vaivilavičius 2008.

*ferrugineus* **(Paykull, 1800)**. Pileckis 1963b, 1982;Valenta and Jakaitis 1972; Jakaitis and Valenta 1976; Silfverberg 1992, 2004; Gaidienė 1993; Pileckis and Monsevičius 1997; Jelínek 2007d; Jelínek and Audisio 2009; Ivinskis et al. 2009.

*grandis* **Gyllenhal, 1827**. Pileckis and Monsevičius 1997; Silfverberg 2004; Ostrauskas and Ferenca 2010.

*nitidulus* **(Fabricius, 1798)**.Pileckis and Jakaitis 1989; Silfverberg 1992, 2004; Pileckis and Monsevičius 1997; Jelínek 2007d; Jelínek and Audisio 2009.

*parallelocollis* **Gyllenhal, 1827**. Pileckis and Jakaitis 1989; Silfverberg 1992, 2004; Gaidienė 1993; Pileckis and Monsevičius 1997; Jelínek 2007d; Jelínek and Audisio 2009.

*parvulus* **(Paykull, 1800)** *= fenestralis* (Linnaeus, 1758). Jakaitis 1985; Silfverberg 1992, 2004; Gaidienė 1993; Pileckis and Monsevičius 1997; Tamutis and Zolubas 2001; Šablevičius 2003a; Jelínek 2007d; Vaivilavičius 2008; Jelínek and Audisio 2009.

[*perforatus* **Erichson, 1845**].Known in Latvia (Telnov 2004), Denmark, Sweden (Lundberg and Gustafsson 1995), Belarus (Alexandrovitch et al. 1996), Poland (Burakowski et al. 1986b), recently found in Estonia (Süda 2009).

*picipes* **(Olivier, 1790)**. Gaidienė 1993; Pileckis and Monsevičius 1997; Tamutis and Zolubas 2001; Šablevičius 2003a, 2004; Silfverberg 2004; Ferenca et al. 2007; Ivinskis et al. 2009.

[*puncticollis* **R.F. Sahlberg, 1837**].Known in northwestern Belarus (Alexandrovitch et al. 1996), eastern Poland (Burakowski et al. 1986b).

**Monotominae Laporte, 1840**.

**Monotomini Laporte, 1840**.

*Monotoma* **Herbst, 1793**. (Cucujidae)

*angusticollis* **(Gyllenhal, 1827)**. Pileckis 1976a; Silfverberg 1992, 2004; Monsevičius 1997; Pileckis and Monsevičius 1997; Jelínek 2007d; Jelínek and Audisio 2009.

[*bicolor* **Villa, 1835**].Known in Latvia (Telnov 2004), Denmark, Sweden (Lundberg and Gustafsson 1995), Estonia (Silfverberg 2004), Belarus (Alexandrovitch et al. 1996), Poland (Burakowski et al. 1986b).

[*brevicollis* **Aubé, 1837**].Known in Latvia (Telnov 2004), Denmark, Sweden (Lundberg and Gustafsson 1995), Estonia (Silfverberg 2004), northwestern Belarus (Alexandrovitch et al. 1996), northern Poland (Burakowski et al. 1986b).

*conicicollis* **Aubé, 1837**. Monsevičius 1988b, 1997; Silfverberg 1992, 2004; Pileckis and Monsevičius 1997; Jelínek 2007d; Jelínek and Audisio 2009.

[*longicollis* **(Gyllenhal, 1827)**]. Known Denmark, Sweden (Lundberg and Gustafsson 1995), Estonia (Silfverberg 2004), northwestern Belarus (Alexandrovitch et al. 1996), northern Poland (Burakowski et al. 1986b).

*picipes* **Herbst, 1793** = *brevipennis* Kunze, 1839. Gaidienė 1993; Pileckis and Monsevičius 1997; Silfverberg 2004; Ferenca et al. 2006; Jelínek and Audisio 2009.

[*quadricollis* **Aubé, 1837**].Known in Denmark, Sweden (Silfverberg 2004).

[*quadrifoveolata* **Aubé, 1837**].Known inDenmark, Sweden (Lundberg and Gustafsson 1995), northern Poland (Burakowski et al. 1986b).

*spinicollis* **Aubé, 1837**. Silfverberg 1992, 2004; Pileckis and Monsevičius 1997.

[*testacea* **Motschulsky, 1845**].Known in Denmark, Sweden (Lundberg and Gustafsson 1995), Estonia (Silfverberg 2004), Poland (Burakowski et al. 1986b).

**CRYPTOPHAGIDAE Kirby, 1826**.

**Cryptophaginae** **Kirby, 1826**.

**Cryptophagini Kirby, 1826**.

*Paramecosoma* **Curtis, 1833**.

*melanocephalum* **(Herbst, 1793)**. Pileckis and Monsevičius 1997; Silfverberg 2004; Johnson et al. 2007; Otero et al. 2009.

*Henoticus* **Thomson, 1868**.

*serratus* **(Gyllenhal, 1808)**. Horion 1960; Pileckis 1968b, 1976a; Silfverberg 1992, 2004; Pileckis and Monsevičius 1997; Johnson et al. 2007; Otero et al. 2009.

*Pteryngium* **Reitter, 1887**.

*crenatum* **(Fabricius, 1798)**. Pileckis 1960, 1976a; Silfverberg 1992, 2004; Gaidienė 1993; Pileckis and Monsevičius 1997; Ferenca 2006b; Johnson et al. 2007; Otero et al. 2009.

*Micrambe* **Thomson, 1863**.

*abietis* **(Paykull, 1798)**. Pileckis 1976a; Silfverberg 1992, 2004; Pileckis and Monsevičius 1997 (*Cryptophagus*); Tamutis and Zolubas 2001; Johnson et al. 2007; Otero et al. 2009.

*bimaculatus* **(Panzer, 1798)**. Otero et al. 2009.

*lindbergorum* **Bruce, 1934**. Otero et al. 2009.

*longitarsis* **J.R. Sahlberg, 1900**. Otero et al. 2009.

*villosus* **(Heer, 1841)**. Otero et al. 2009.

*vini* **(Panzer, 1797).** Pileckis and Monsevičius 1997; Ivinskis et al. 2009.

*Cryptophagus* **Herbst, 1792**.

*acutangulus* **Gyllenhal, 1827**. Pileckis 1976a, 1998; Silfverberg 1992, 2004; Gaidienė 1993; Pileckis and Monsevičius 1997; Ferenca 2006b; Johnson et al. 2007; Otero et al. 2009.

*affinis* **Sturm, 1845** = *laticollis* Lucas, 1846. Pileckis 1963b, 1976a; Silfverberg 1992, 2004; Pileckis and Monsevičius 1997; Johnson et al. 2007.

*badius* **Sturm, 1845**. Silfverberg 1992, 2004; Pileckis and Monsevičius 1997; Johnson et al. 2007.

*cellaris* **(Scopoli, 1763)**. Tamutis et al. 2008.

*cylindrus* **Kiesenwetter, 1858**. Otero et al. 2009.

*confertus* **Casey, 1900** = *archangelicus* J.R. Sahlberg, 1926. Otero et al. 2009.

*confusus* **Bruce, 1934**.Otero et al. 2009.

[*corticinus* **Thomson, 1863**].Known in Latvia (Telnov 2004), Denmark, Sweden (Lundberg and Gustafsson 1995), northwestern Belarus (Alexandrovitch et al. 1996), Kaliningrad region, Poland (Otero et al. 2009).

*dentatus* **(Herbst, 1793)**. Pileckis 1976a; Bercio and Folwaczny 1979; Silfverberg 1992, 2004; Pileckis and Monsevičius 1997; Šablevičius 2000a, 2003a; Johnson et al. 2007; Otero et al. 2009; Ostrauskas and Ferenca 2010.

*distinguendus* **Sturm, 1845**. Pileckis 1963b, 1976a, 1998; Silfverberg 1992, 2004; Pileckis and Monsevičius 1997; Johnson et al. 2007.

*dorsalis* **C.R. Sahlberg, 1819**. Pileckis 1976a; Bercio and Folwaczny 1979; Silfverberg 1992, 2004; Pileckis and Monsevičius 1997; Johnson et al. 2007; Otero et al. 2009.

[*falcozi* **Roubal, 1927** = *westi* Bruce, 1943]. Known in Estonia, Denmark (Silfverberg 2004).

*fallax* **Balfour-Browne, 1953** = *fumatus* auct. nec Marsham, 1802. Monsevičius and Pankevičius 2001; Johnson et al. 2007; Otero et al. 2009.

*fuscicornis* **Sturm, 1845**. Bercio and Folwaczny 1979; Silfverberg 2004; Johnson et al. 2007; Otero et al. 2009.

*immixtus* **Rey, 1889** = *postpositus* J.R. Sahlberg, 1903. Monsevičius and Pankevičius 2001; Johnson et al. 2007; Otero et al. 2009.

[*intermedius* **Bruce, 1934**].Known in Denmark, Sweden (Lundberg and Gustafsson 1995), Estonia (Silfverberg 2004), Poland (Burakowski et al. 1986b).

*labilis* **Erichson, 1846**. Silfverberg 1992, 2004; Johnson et al. 2007; Otero et al. 2009; as *Cryptophagus labiatus* Er. in Pileckis 1976a.

*lapponicus* **Gyllenhal, 1827**.Otero et al. 2009.

*lycoperdi* **(Scopoli, 1763)**. Monsevičius and Pankevičius 2001; Johnson et al. 2007.

*lysholmi* **Munster, 1932**.Otero et al. 2009.

[*micaceus* **Rey, 1889**].Known in Latvia (Telnov 2004), Denmark, Sweden (Lundberg and Gustafsson 1995), Belarus, Kaliningrad region, Poland (Otero et al. 2009).

*obsoletus* **Reitter, 1879**. Monsevičius and Pankevičius 2001; Johnson et al. 2007.

*pallidus* **Sturm, 1845**. Tenenbaum 1931; Pileckis 1968b, 1976a; Silfverberg 1992, 2004; Pileckis and Monsevičius 1997; Johnson et al. 2007; Otero et al. 2009.

[*parallelus* **Brisout, 1863** = *angustus* Ganglbauer, 1899].Known in Latvia (Telnov 2004), Estonia, Denmark, throughout Sweden (Lundberg and Gustafsson 1995).

*pilosus* **Gyllenhal, 1828** *= punctipennis* Brisout, 1863 = *denticulatus* Heer, 1841. Monsevičius and Pankevičius 2001; Johnson et al. 2007; Otero et al. 2009.

[*populi* **Paykull, 1800**].Known in Latvia (Telnov 2004), Estonia (Silfverberg 2004), Denmark, Sweden (Lundberg and Gustafsson 1995), northern Poland (Burakowski et al. 1986b), Belarus (Otero et al. 2009).

*pseudodentatus* **Bruce, 1934**. Monsevičius and Pankevičius 2001; Otero et al. 2009.

*pubescens* **Sturm, 1845**. Roubal 1910; Pileckis 1960, 1976a; Silfverberg 1992, 2004; Pileckis and Monsevičius 1997; Johnson et al. 2007.

*quercinus* **Kraatz, 1852**. Pileckis 1976a; Silfverberg 1992, 2004; Pileckis and Monsevičius 1997; Johnson et al. 2007; Otero et al. 2009.

*saginatus* **Sturm, 1845**. Monsevičius and Pankevičius 2001; Johnson et al. 2007; Otero et al. 2009.

*scanicus* **(Linnaeus, 1758)** *= reflexus* Rey, 1889. Pileckis 1976a; Silfverberg 1992, 2004; Pileckis and Monsevičius 1997; Johnson et al. 2007; Otero et al. 2009; Ivinskis et al. 2009.

*schmidti* **Sturm, 1845**. Otero et al. 2009.

*scutellatus* **Newman, 1834**. Pileckis 1976a; Pileckis and Monsevičius 1997; Silfverberg 1992, 2004; Johnson et al. 2007; Otero et al. 2009.

*setulosus* **Sturm, 1845**. Bercio and Folwaczny 1979; Silfverberg 1992, 2004; Johnson et al. 2007; Ivinskis et al. 2009.

*subdepressus* **Gyllenhal, 1827**.Otero et al. 2009.

*subfumatus* **Kraatz, 1856**. Pileckis 1962, 1963b, 1976a; Silfverberg 1992, 2004; Pileckis and Monsevičius 1997; Johnson et al. 2007; Otero et al. 2009.

*Spavius* **Motschulsky, 1844** = *Emphylus* Erichson, 1846.

*glaber* **(Gyllenhal, 1808)**. Pileckis 1976a; Silfverberg 1992, 2004; Monsevičius 1997; Pileckis and Monsevičius 1997; Johnson et al. 2007 Otero et al. 2009.

*Antherophagus* **Dejean, 1821**.

*canescens* **Grouvelle, 1916**. Monsevičius and Pankevičius 2001; Johnson et al. 2007; Otero et al. 2009.

*nigricornis* **(Fabricius, 1787)**. Mazurowa and Mazur 1939; Pileckis 1960, 1976a; Gaidienė 1993; Monsevičius 1997; Pileckis and Monsevičius 1997; Silfverberg 1992, 2004; Otero et al. 2009.

*pallens* **(Linnaeus, 1758)**. Silfverberg 1992, 2004; Pileckis and Monsevičius 1997; Šablevičius 2004; Johnson et al. 2007; Otero et al. 2009; Ostrauskas and Ferenca 2010.

*Telmatophilus* **Heer, 1841**.

[*brevicollis* **Aubé, 1862**]. Known in northwestern Belarus (Alexandrovitch et al. 1996), Denmark (Silfverberg 2004), Sweden, Estonia, Latvia (Otero et al. 2009), Poland (Burakowski et al. 1986b).

*caricis* **(Olivier, 1790)**. Pileckis 1976a; Silfverberg 1992, 2004; Gaidienė 1993; Pileckis and Monsevičius 1997; Johnson et al. 2007; Otero et al. 2009.

[*schoenherrii* **(Gyllenhal, 1808)**].Known in Latvia (Telnov 2004), Denmark, Sweden (Lundberg and Gustafsson 1995), northern Poland (Burakowski et al. 1986b).

*typhae* **(Fallén, 1802)**. Pileckis 1976a; Bercio and Folwaczny 1979; Silfverberg 1992, 2004; Pileckis and Monsevičius 1997; Ferenca 2004; Johnson et al. 2007; Otero et al. 2009.

**Caenoscelini Casey, 1900**.

*Caenoscelis* **Thomson, 1863**.

*ferruginea* **(C.R. Sahlberg, 1820)**. Pileckis 1976a; Silfverberg 1992, 2004; Pileckis and Monsevičius 1997; Johnson et al. 2007; Otero et al. 2009.

[*sibirica* **Reitter, 1889** = *fleischeri* Reitter, 1889 = *grandis* Thomson, 1892].Known in Denmark, throughout Sweden (Lundberg and Gustafsson 1995), Estonia (Sillfverberg 2004), Poland (Otero et al. 2009).

*subdeplanata* **Brisout, 1882**. Otero et al. 2009.

**Atomariinae** **LeConte, 1861**.

**Atomariini LeConte, 1861**.

*Atomaria* **Stephens, 1830**.

*affinis* **R.F. Sahlberg, 1834**. Tamutis and Zolubas 2001; Silfverberg 2004; Johnson et al. 2007.

*alpina* **Heer, 1841**.Otero et al. 2009.

*analis* **Erichson, 1846**. Monsevičius and Pankevičius 2001; Johnson et al. 2007; Otero et al. 2009.

*apicalis* **Erichson, 1846**.Otero et al. 2009.

[*atra* **(Herbst, 1793)**].Known in Latvia (Telnov 2004), northwestern Belarus (Alexandrovitch et al. 1996), northeastern Poland (Burakowski et al. 1986b).

*atrata* **Reitter, 1875**. Monsevičius and Pankevičius 2001; Johnson et al. 2007.

*atricapilla* **Stephens, 1830**. Monsevičius and Pankevičius 2001; Johnson et al. 2007.

*badia* **Erichson, 1846** = *sahlbergi* Sjöberg, 1947].Known in Sweden (Lundberg and Gustafsson 1995), Belarus (Alexandrovitch et al. 1996), Poland (Otero et al. 2009).

*barani* **Brisout, 1863**. Otero et al. 2009.

*basalis* **Erichson, 1846**. Monsevičius and Pankevičius 2001; Johnson et al. 2007; Otero et al. 2009.

*bella* **Reitter, 1875**. Otero et al. 2009.

[*clavigera* **Ganglbauer, 1899**].Known in Denmark, throughout Sweden (Lundberg and Gustafsson 1995), northeastern Poland (Burakowski et al. 1986b).

*diluta* **Erichson, 1846**. Tamutis and Zolubas 2001; Silfverberg 2004; Johnson et al. 2007; Otero et al. 2009.

[*elongatula* **Erichson, 1846**]. Known in Latvia (Telnov 2004), Estonia, Denmark, Poland (Otero et al. 2009), Sweden (Lundberg and Gustafsson 1995).

*fimetarii* **(Fabricius, 1792)**. Monsevičius and Pankevičius 2001; Johnson et al. 2007; Otero et al. 2009.

*fuscata* **(Schönherr, 1808)** = *agnita* Kangas, 1961. Heyden 1903; Pileckis 1960, 1976a; Silfverberg 1992, 2004; Pileckis and Monsevičius 1997; Šablevičius 2001, 2003a; Johnson et al. 2007; Otero et al.2009.

*fuscipes* **(Gyllenhal, 1808)**. Pileckis 1976a; Bercio and Folwaczny 1979; Silfverberg 1992, 2004; Pileckis and Monsevičius 1997; Johnson et al. 2007; Otero et al. 2009.

[*gravidula* **Erichson, 1846**].Known in Kaliningrad region, Poland, Sweden (Otero et al. 2009), Estonia (Lundberg and Gustafsson 1995).

[*gutta* **Newman, 1834**]. Known in Latvia (Telnov 2004), Denmark, southern Sweden (Lundberg and Gustafsson 1995), northern Poland (Burakowski et al. 1986b), Kaliningrad region (Otero et al. 2009).

[*hislopi* **Wollaston, 1857**].Known in Sweden (Lundberg and Gustafsson 1995), Estonia (Silfverberg 2004).

[*impressa* **Erichson, 1846**].Known in Sweden (Lundberg and Gustafsson 1995), Estonia (Sillfverberg 2004), northern Poland (Burakowski et al. 1986b), Belarus, Kaliningrad region (Otero et al. 2009).

*lewisi* **Reitter, 1877**. Monsevičius and Pankevičius 2001; Johnson et al. 2007; Otero et al. 2009.

[*linearis* **Stephens, 1830**]. Known in all neighbours countries (Burakowski et al. 1986b; Lundberg and Gustafsson 1995; Alexandrovitch et al. 1996; Telnov 2004; Otero et al. 2009).

*mesomela* **(Herbst, 1792)**. Bercio and Folwaczny 1979; Silfverberg 1992, 2004; Johnson et al. 2007; Otero et al. 2009.

*morio* **Kolenati, 1846**. Monsevičius and Pankevičius 2001; Johnson et al. 2007.

*munda* **Erichson, 1846**. Monsevičius and Pankevičius 2001; Johnson et al. 2007.

*nigripennis* **(Kugelann, 1794)**. Pileckis 1976a; Silfverberg 1992, 2004; Pileckis and Monsevičius 1997; Johnson et al. 2007.

*nigrirostris* **Stephens, 1830** = *fuscicollis* Mannerheim, 1852). Monsevičius and Pankevičius 2001; Johnson et al. 2007; Otero et al. 2009.

*nigriventris* **Stephens, 1830**. Otero et al. 2009.

*nitidula* **(Marsham, 1802)** = *reitteri* Sjöberg, 1947. Monsevičius and Pankevičius 2001; Johnson et al. 2007; Otero et al. 2009.

[*ornata* **Heer, 1841** = *contaminata* Erichson, 1846]. Known in Denmark, throughout Sweden (Lundberg and Gustafsson 1995), northern Poland (Burakowski et al. 1986b), Kaliningrad region (Otero et al. 2009).

*peltata* **Kraatz, 1853**.Otero et al. 2009.

[*peltataeformis* **Sjöberg, 1947**].Known in Sweden (Lundberg and Gustafsson 1995), Estonia (Sillfverberg 2004).

*procerula* **Erichson, 1846** = *longicornis* Thomson, 1863. Monsevičius and Pankevičius 2001; Johnson et al. 2007; Otero et al. 2009.

*pseudoatra* **Reitter, 1887** = *reitteri* Lövendal, 1892. Monsevičius and Pankevičius 2001; Johnson et al. 2007; Otero et al. 2009.

*pulchra* **Erichson, 1846** = *vespertina* Mäklin, 1853. Monsevičius and Pankevičius 2001, Johnson et al. 2007.

[*puncticollis* **Thomson, 1868**].Known in Denmark, Sweden (Lundberg and Gustafsson 1995), Poland (Burakowski et al. 1986b).

[*punctithorax* **Reitter, 1887** = *consanguinea* Johnson, 1976].Known in Denmark, southern Sweden (Lundberg and Gustafsson 1995).

*pusilla* **(Paykull, 1798)**. Bercio and Folwaczny 1979; Silfverberg 1992, 2004; Johnson et al. 2007; Otero et al. 2009.

[*rhenana* **Kraatz, 1853**]. Known in Denmark, southern Sweden (Lundberg and Gustafsson 1995), northern Poland (Otero et al. 2009).

*rubella* **Heer, 1841** = *berolinensis* Kraatz, 1852. Monsevičius and Pankevičius 2001; Johnson et al. 2007; Otero et al. 2009.

*rubida* **Reitter, 1875**.Otero et al. 2009.

[*rubricollis* **Brisout, 1863**]. Known in Latvia (Telnov 2004), Denmark, southern Sweden (Lundberg and Gustafsson 1995), Estonia (Silfverberg 2004), northern Poland (Burakowski et al. 1986b).

[*soedermani* **Sjöberg, 1947**].Known in Sweden (Lundberg and Gustafsson 1995), Estonia, Denmark (Sillfverberg 2004).

[*strandi* **Johnson, 1967**]. Known in Denmark, Sweden (Silfverberg 2004).

[*subangulata* **C.R. Sahlberg, 1826**].Known in Latvia (Telnov 2004), Denmark (Silfverberg 2004), Sweden (Lundberg and Gustafsson 1995).

*testacea* **Stephens, 1830** = *ruficornis* (Marsham, 1802) nec (Gmelin, 1790). Pileckis and Monsevičius 1997; Silfverberg 2004; Johnson et al. 2007; Otero et al. 2009.

*turgida* **Erichson, 1846**. Tamutis and Zolubas 2001; Monsevičius and Pankevičius 2001; Silfverberg 2004; Johnson et al. 2007; Otero et al. 2009.

*umbrina* **(Gyllenhal, 1827)** = *sjöbergi* Palm, 1949. Tamutis and Zolubas 2001; Silfverberg 2004; Johnson et al. 2007; Otero et al. 2009.

[*wollastoni* **Sharp, 1867**].Known in Denmark, Sweden (Lundberg and Gustafsson 1995), Estonia (Otero et al. 2009).

*zetterstedti* **(Zetterstedt, 1838)**.Otero et al. 2009.

*Ootypus* **Ganglbauer, 1899**.

*globosus* **(Waltl, 1838)**. Pileckis and Monsevičius 1997; Silfverberg 2004; Johnson et al. 2007; Otero et al. 2009.

*Ephistemus* **Stephens, 1829**.

*globulus* **(Paykull, 1798)**. Pileckis 1976a; Bercio and Folwaczny 1979; Silfverberg 1992, 2004; Pileckis and Monsevičius 1997; Johnson et al. 2007; Otero et al. 2009.

*Curelius* **Casey, 1900**.

*exiguus* **(Erichson, 1846)**.Otero et al. 2009.

**Hypocoprini Reitter, 1880**.

*Hypocoprus* **Motschulsky, 1839**.

*latridioides* **Motschulsky, 1839** = *quadricollis* Reitter, 1877. Horion 1960; Pileckis 1968b, 1976a; Bercio and Folwaczny 1979; Silfverberg 1992, 2004; Pileckis and Monsevičius 1997; Johnson et al. 2007.

**PHLOESTICHIDAE Reitter, 1911**.

*Phloeostichus* **Redtenbacher, 1842**.

[*denticollis* **Redtenbacher, 1842**]. Known in Denmark (Silfverberg 2004), northeastern Poland (Burakowski et al. 1986b).

**SILVANIDAE Kirby, 1837**.

**Brontinae Blanchard, 1845**.

**Brontini Blanchard, 1845**.

*Uleiota* **Latreille, 1796**. (Cucujidae)

*planata* **(Linnaeus, 1761)**. Pileckis 1968a, 1976a, 1982; Bercio and Folwaczny 1979; Silfverberg 1992, 2004; Gaidienė 1993; Monsevičius 1997; Pileckis and Monsevičius 1997; Šablevičius 2000b, 2011; Ehnström et al. 2003; Ivinskis et al. 2006; Halstead et al. 2007; Vaivilavičius 2008; Ślipinski 2009.

*Dendrophagus* **Schönherr, 1809**. (Cucujidae)

*crenatus* **(Paykull, 1799)**. Silfverberg 1992, 2004; Gaidienė 1993; Žiogas and Gedminas 1994; Monsevičius 1997; Pileckis and Monsevičius 1997; Ehnström 2003; Šablevičius 2000b, 2001, 2003a, 2011; Ferenca 2004; Ferenca et al. 2006, 2007; Butvila et al. 2007; Halstead et al. 2007; Vaivilavičius 2008; Ślipinski 2009; Ivinskis et al. 2009.

**Telephanini LeConte, 1861**.

*Psammoecus* **Latreille, 1829**.

*bipunctatus* **(Fabricius, 1792)**. Miländer et al. 1984; Silfverberg 1992, 2004; Pileckis and Monsevičius 1997; Ferenca 2004; Butvila et al. 2007; Alekseev 2008a; Ślipinski 2009; Ivinskis et al. 2009.

**Silvaninae Kirby, 1837**.

*Airaphilus* **Redtenbacher, 1858**.

[*elongatus* **(Gyllenhal, 1813)**]. Known in Latvia (Telnov 2004), Estonia, Sweden (Lundberg and Gustafsson 1995), Poland (Burakowski et al. 1986b).

[*perangustus* **Lindberg, 1943**].Known in Finland (Lundberg and Gustafsson 1995), northern Poland (Burakowski et al. 1986b).

*Ahasverus* **Dez Gozis, 1881**.

*advena* **(Waltl, 1834)**. Monsevičius and Pankevičius 2001; Halstead et al. 2007; Ślipinski 2009.

*Oryzaephilus* **Ganglbauer, 1899**.

*mercator* **(Fauvel, 1889)**. Silfverberg 1992, 2004; Lundberg and Gustafsson 1995; Halstead et al. 2007; Ślipinski 2009.

*surinamensis* **(Linnaeus, 1758)**. Pileckis 1960, 1970a, 1976a, 1998; Lešinskas and Pileckis 1967; Zubrys 1967; Silfverberg 1992, 2004; Gaidienė 1993; Pileckis et al. 1994b; Pileckis and Monsevičius 1997; Halstead et al. 2007; Ślipinski 2009.

*Silvanus* **Latreille, 1807**.

*bidentatus* **(Fabricius, 1792)**. Pileckis 1976a; Silfverberg 1992, 2004; Pileckis and Monsevičius 1997; Halstead et al. 2007; Ślipinski 2009; Vaivilavičius 2008.

*unidentatus* **(Olivier, 1790)**. Pileckis 1976a; Silfverberg 1992, 2004; Pileckis and Monsevičius 1997; Šablevičius 2000b; Ferenca 2004; Halstead et al. 2007; Vaivilavičius 2008; Ślipinski 2009.

*Silvanoprus* **Reitter, 1911**.

*fagi* **(Guérin-Ménéville, 1844)**. Gaidienė and Ferenca 1988; Silfverberg 1992, 2004; Gaidienė 1993; Pileckis and Monsevičius 1997; Halstead et al. 2007; Ślipinski 2009.

**CUCUJIDAE Latreille, 1802**.

*Cucujus* **Fabricius, 1775**.

RDB*cinnaberinus* **(Scopoli, 1763)**. Pileckis 1960, 1976a; Silfverberg 1992, 2004; Pileckis and Monsevičius 1997; Ehnström et al. 2003; Ferenca 2004; Ivinskis et al. 2004b, 2007b; Tamutis 2005b; Rašomavičius 2007; Vitkauskas 2007; Wegrzynowicz 2007a; Vaivilavičius 2008; Dapkus and Tamutis 2008b; Inokaitis 2009; Alikonis and Švitra 2009; Alekseev 2010b; Šablevičius 2011.

RDB*haematodes* **Erichson, 1845**. Pileckis 1976a, 1982; Silfverberg 1992, 2004; Pileckis and Monsevičius 1997; Monsevičius 1997; Ehnström et al. 2003; Tamutis 2005b; Ivinskis et al. 2007; Rašomavičius 2007; Wegrzynowicz 2007a; Vaivilavičius 2008; Alekseev 2010b; Šablevičius 2011.

*Pediacus* **Shuckard, 1839**.

*depressus* **(Herbst, 1797)**. Pileckis and Monsevičius 1997; Silfverberg 2004; Butvila et al. 2007.

[*dermestoides* **(Fabricius, 1793)**].Known in Denmark (Lundberg and Gustafsson 1995), Estonia (Ślipinski 2009), northeastern Poland (Burakowski et al. 1986b).

[*fuscus* **Erichson, 1845**]. Known in Latvia (Telnov 2004), Estonia, Sweden (Lundberg and Gustafsson 1995).

**PHALACRIDAE Leach, 1815**.

**Phalacrinae Leach, 1815**.

*Phalacrus* **Paykull, 1800**.

[*championi* **Guillebeau, 1892** = *suecicus* Palm, 1947]. Known in Latvia (Telnov 2004), Sweden (Lundberg and Gustafsson 1995), Estonia (Silfverberg 2004).

*corruscus* **(Panzer, 1797)** = *fimetarius* auct. nec (Fabricius, 1775). Silfverberg 1992, 2004; Lundberg and Gustafsson 1995; Švec 2007, 2009.

[*fimetarius* **(Fabricius, 1775)** = *brisouti* Rye, 1872 = *hybridus* Flach 1888]. Known in Latvia (Telnov 2004), Denmark, southern Sweden (Lundberg and Gustafsson 1995), Poland (Švec 2007, 2009).

[*grossus* **Erichson, 1845** = *dieckmanni* Vogt, 1967]. Known in Latvia (Telnov et al. 2005), Denmark, southern Sweden (Lundberg and Gustafsson 1995), northern Poland (Burakowski et al. 1986b), recently found in Estonia (Süda 2009).

*nigrinus* **(Marsham, 1802)** = *caricis* Sturm, 1807. Monsevičius 1988b, 1997; Silfverberg 1992, 2004; Pileckis and Monsevičius 1997; Švec 2007, 2009; Alekseev 2008a; Ivinskis et al. 2009.

*substriatus* **Gyllenhal, 1813**. Pileckis 1976a; Silfverberg 1992, 2004; Gaidienė 1993; Monsevičius 1997; Pileckis and Monsevičius 1997; Švec 2007, 2009.

*Olibrus* **Erichson, 1845**.

*aeneus* **(Fabricius, 1792)**. Pileckis 1968b, 1976a; Silfverberg 1992, 2004; Gaidienė 1993; Pileckis and Monsevičius 1997; Švec 2007, 2009.

*affinis* **(Sturm, 1807)**. Pileckis 1968b, 1976a; Silfverberg 1992, 2004; Monsevičius 1997; Pileckis and Monsevičius 1997; Švec 2007, 2009.

[*baudueri* **Flach, 1888**].Known in Denmark, southern Sweden (Lundberg and Gustafsson 1995), Belarus (Alexandrovitch et al. 1996), northern Poland (Burakowski et al. 1986b).

*bicolor* **(Fabricius, 1792)**. Pileckis 1968b, 1976a; Silfverberg 1992, 2004; Gaidienė 1993; Monsevičius 1997; Pileckis and Monsevičius 1997; Šablevičius 2000b; Žiogas and Zolubas 2005; Švec 2007a.

*bimaculatus* **Küster, 1848**. Ivinskis et al. 2009.

*corticalis* **(Panzer, 1797)**. Bercio and Folwaczny 1979; Silfverberg 1992, 2004; Pileckis and Monsevičius 1997; Švec 2007, 2009.

[*flavicornis* **(Sturm, 1807)**].Known in Latvia, Estonia, Sweden (Lundberg and Gustafsson 1995), northern Poland (Burakowski et al. 1986b).

[*liquidus* **Erichson, 1845**]. Known in Latvia (Telnov 2004), northern Poland (Burakowski et al. 1986b).

*millefolii* **(Paykull, 1800)**. Roubal 1910; Pileckis 1960, 1976a; Silfverberg 1992, 2004; Gaidienė 1993; Pileckis and Monsevičius 1997; Švec 2007, 2009.

*norvegicus* **Munster, 1901**. Monsevičius and Pankevičius 2001; Švec 2007, 2009.

[*pygmaeus* **(Sturm, 1807)**].Known in Latvia (Telnov 2004), Denmark, southern Sweden (Lundberg and Gustafsson 1995), northern Poland (Burakowski et al. 1986b).

*Stilbus* **Seidlitz, 1872**.

[*atomarius* **(Linnaeus, 1767)**].Known in Latvia (Telnov 2004), Estonia, Denmark, Sweden (Lundberg and Gustafsson 1995), northern Poland (Burakowski et al. 1986b).

*oblongus* **(Erichson, 1845)**. Gaidienė 1993; Pileckis and Monsevičius 1997; Silfverberg 2004.

*testaceus* **(Panzer, 1797)**. Gaidienė 1993; Pileckis and Monsevičius 1997; Silfverberg 2004.

**LAEMOPHLOEIDAE Ganglbauer, 1899**. (Cucujidae)

*Laemophloeus* **Dejean, 1835**.

[*kraussi* **Ganglbauer, 1897**]. Known in northern Poland (Burakowski et al. 1986b).

[*monilis* **(Fabricius, 1787)**].Known in Denmark, Sweden (Lundberg and Gustafsson 1995), Belarus (Alexandrovitch et al. 1996), northern Poland (Burakowski et al. 1986b).

[*muticus* **(Fabricius, 1781)**]. Known in Latvia (Telnov 2004), Estonia, Denmark, Sweden (Lundberg and Gustafsson 1995), northern Belarus (Alexandrovitch et al. 1996), northeastern Poland (Burakowski et al. 1986b).

*Notolaemus* **Lefkovitch, 1959**.

*castaneus* **(Erichson, 1845)**.Tamutis and Ferenca 2006; Ferenca et al. 2006, 2007.

[*unifasciatus* **(Latreille, 1804)** = *bimaculatus* (Paykull, 1801) nec (Olivier, 1791)]. Known in Denmark, Sweden (Lundberg and Gustafsson 1995), northern Poland (Burakowski et al. 1986b), recently found in Estonia (Süda 2009).

*Cryptolestes* **Ganglbauer, 1899**.

*abietis* **(Wankowicz, 1865)**. Pileckis 1976a; Silfverberg 1992, 2004; Pileckis and Monsevičius 1997; Wegrzynowicz 2007b; Ślipinski 2009.

*alternans* **(Erichson, 1846)**. Valenta and Jakaitis 1972; Jakaitis 1973; Pileckis 1976a; Silfverberg 1992, 2004; Pileckis and Monsevičius 1997; Šablevičius 2004; Wegrzynowicz 2007b.

[*capensis* **(Waltl, 1832)**].Known in Denmark (Lundberg and Gustafsson 1995), Sweden (Ślipinski 2009), Poland (Burakowski et al. 1986b).

*corticinus* **(Erichson, 1846)**. Pileckis 1976a; Silfverberg 1992, 2004; Pileckis and Monsevičius 1997; Wegrzynowicz 2007b.

[*duplicatus* **(Waltl, 1839)**].Known in Denmark, Sweden (Lundberg and Gustafsson 1995), Belarus (Alexandrovitch et al. 1996), northern Poland (Burakowski et al. 1986b).

*ferrugineus* **(Stephens, 1831)**. Ogyjewicz 1934; Pileckis 1960, 1970a, 1976a; Zubrys 1967; Silfverberg 1992, 2004; Pileckis and Monsevičius 1997; Wegrzynowicz 2007b; Šablevičius 2011.

[*pusillus* **(Schönherr, 1817)** = *minutus* (Olivier, 1791) nec (Geoffroy, 1785)]. Known in Denmark, Sweden (Lundberg and Gustafsson 1995), northern Belarus (Alexandrovitch et al. 1996), northern Poland (Burakowski et al. 1986b).

[*spartii* **(Curtis, 1834)** = *alter* (Olivier, 1795) nec (Geoffroy, 1785)].Known in Estonia, Sweden (Lundberg and Gustafsson 1995), northern Poland (Burakowski et al. 1986b).

[*turcicus* **(Grouvelle, 1876)**].Known in Latvia (Telnov 2010), Denmark, Sweden (Lundberg and Gustafsson 1995), northern Poland (Burakowski et al. 1986b).

*Placonotus* **MacLeay, 1871**.

[*testaceus* **(O.F. Müller, 1821)**].Known in Latvia (Telnov 2010), Sweden (Lundberg and Gustafsson 1995), Denmark (Silfverberg 2004), Belarus (Alexandrovitch et al. 1996), northern Poland (Burakowski et al. 1986b).

*Lathropus* **Erichson, 1845**.

[*sepicola* **(Erichson, 1846)**].Known in nortwestern Belarus (Alexandrovitch et al. 1996), Poland (Burakowski et al. 1986b).

**KATERETIDAE Erichson, 1846**.

*Kateretes* **Herbst, 1793**.

[*dalmatinus* **(Sturm, 1844)**]. Known in Latvia (Telnov et al. 2006), Belarus (Audisio and Jelínek 2009).

*pedicularius* **(Linnaeus, 1758)**. Pileckis 1968a, 1976a; Silfverberg 1992, 2004; Gaidienė 1993; Pileckis and Monsevičius 1997; Monsevičius 1997; Jelínek 2007c; Audisio and Jelínek 2009.

*pusillus* **(Thunberg, 1794)** = *bipustulatus* (Paykull, 1798) nec (Thunberg, 1781). Pileckis and Monsevičius 1982, 1997; Silfverberg 1992, 2004; Gaidienė 1993; Monsevičius 1997; Šablevičius 2003a; Jelínek 2007c; Audisio and Jelínek 2009.

*rufilabris* **(Latreille, 1807)**. Gaidienė 1993; Tamutis 2003; Silfverberg 2004.

*Heterhelus* **Jacquelin du Val, 1858**.

*scutellaris* **(Heer, 1841)**. Ferenca and Tamutis 2009; Audisio and Jelínek 2009.

*Brachypterus* **Kugelann, 1794**.

*fulvipes* **Erichson, 1843**. Monsevičius 1988b, 1997; Silfverberg 1992, 2004; Gaidienė 1993; Pileckis and Monsevičius 1997; Šablevičius 2000b; Jelínek 2007c.

*glaber* **(Stephens, 1835)**. Pileckis and Monsevičius 1982, 1997; Silfverberg 1992, 2004; Gaidienė 1993; Jelínek 2007c; Audisio and Jelínek 2009.

*urticae* **(Fabricius, 1792)** = *erythropus* (Marsham, 1802). Roubal 1910; Pileckis 1960, 1963b, 1976a; Silfverberg 1992, 2004; Pileckis and Monsevičius 1997; Gliaudys 2001; Jelínek 2007c; Audisio and Jelínek 2009; Šablevičius 2011.

*Brachypterolus* **Grouvelle, 1913**.

*antirrhini* **(Murray, 1864)** = *villiger* (Reitter, 1885). Bercio and Folwaczny 1979; Silfverberg 1992, 2004; Monsevičius 1998; Audisio and Jelínek 2009.

*linariae* **(Stephens, 1830)** = *cornelii* Spornraft, 1966.Audisio and Jelínek 2009.

*pulicarius* **(Linnaeus, 1758)**. Bercio and Folwaczny 1979; Pileckis and Monsevičius 1982, 1997; Silfverberg 1992, 2004; Gaidienė 1993; Monsevičius 1997; Audisio and Jelínek 2009; Jelínek 2007c; Alekseev 2008a.

**NITIDULIDAE Latreille, 1802**.

**Epuraeinae** **Kirejtshuk, 1986**.

**Epuraeini Kirejtshuk, 1986**.

*Epuraea* **Erichson, 1843**.

*aestiva* **(Linnaeus, 1758)** = *depressa* (Illiger, 1798). Silfverberg 1992, 2004; Pileckis and Monsevičius 1997; Jelínek and Audisio 2007; Audisio and Jelínek 2009; Ivinskis et al. 2009.

*angustula* **Sturm, 1844**. Karalius and Monsevičius 1992; Silfverberg 1996, 2004; Pileckis and Monsevičius 1997; Jelínek and Audisio 2007; Audisio and Jelínek 2009.

*biguttata* **(Thunberg, 1784)**. Monsevičius and Pankevičius 2001; Jelínek and Audisio 2007; Audisio and Jelínek 2009; Ivinskis et al. 2009.

*binotata* **Reitter, 1872**. Monsevičius and Pankevičius 2001; Jelínek and Audisio 2007; Audisio and Jelínek 2009.

*boreella* **(Zetterstedt, 1828)**. Pileckis 1976a; Silfverberg 1992, 2004; Pileckis and Monsevičius 1997; Jelínek and Audisio 2007; Audisio and Jelínek 2009; Ivinskis et al. 2009.

[*deubeli* **Reitter, 1898**]. Known in Estonia, Denmark, Sweden (Lundberg and Gustafsson 1995), Poland (Burakowski et al. 1986b).

*distincta* **(Grimmer, 1841)**. Karalius and Monsevičius 1992; Silfverberg 1996, 2004; Pileckis and Monsevičius 1997.

[*excisicollis* **Reitter, 1872**].Known in Denmark, southern Sweden (Lundberg and Gustafsson 1995), Belarus, Latvia (Audisio and Jelínek 2009). 

*fageticola* **Audisio, 1991** = *castanea* Duftschmid, 1825. # 59.Jelínek and Audisio 2007; Audisio and Jelínek 2009.

[*fuscicollis* **(Stephens, 1835)**]. Known in Denmark, southern Sweden (Lundberg and Gustafsson 1995), Kaliningrad region (Audisio and Jelínek 2009), Poland (Burakowski et al. 1986b).

*fussi* **Reitter, 1875** = *interjecta* Sjöberg, 1939. Monsevičius and Pankevičius 2001.

*guttata* **(Olivier, 1811)** = *decemguttata* Fabricius, 1792. Roubal 1910; Pileckis 1960, 1976a; Lešinskas and Pileckis 1967; Silfverberg 1992, 2004; Gaidienė 1993; Pileckis and Monsevičius 1997; Ferenca 2004; Jelínek and Audisio 2007; Audisio and Jelínek 2009.

*laeviuscula* **(Gyllenhal, 1827)**. Monsevičius 1988a, 1997; Silfverberg 1992, 2004; Pileckis and Monsevičius 1997; Jelínek and Audisio 2007; Audisio and Jelínek 2009.

*limbata* **(Fabricius, 1787)**. Bercio and Folwaczny 1979; Monsevičius and Pankevičius 2001; Jelínek and Audisio 2007; Audisio and Jelínek 2009; Ivinskis et al. 2009.

*longiclavis* **Sjöberg, 1939**.Jelínek and Audisio 2007; Audisio and Jelínek 2009.

*longula* **Erichson, 1845**.Jelínek and Audisio 2007; Audisio and Jelínek 2009.

*marseuli* **Reitter, 1872** = *bickhardti* Saint-Claire Deville, 1906 = *acuta* Biström, 1977 = *pusilla* (Illiger, 1798) nec (Thunberg, 1794). Bercio and Folwaczny 1979; Monsevičius 1988a, 1997; Silfverberg 1992, 2004; Pileckis and Monsevičius 1997; Jelínek and Audisio 2007; Audisio and Jelínek 2009; Ivinskis et al. 2009.

*melanocephala* **(Marsham, 1802)**. Monsevičius and Pankevičius 2001; Jelínek and Audisio 2007; Audisio and Jelínek 2009.

*melina* **Erichson, 1843**. Monsevičius and Pankevičius 2001; Jelínek and Audisio 2007; Audisio and Jelínek 2009.

*muehli* **Reitter, 1908**.Jelínek and Audisio 2007.

*neglecta* **(Heer, 1841)**. Monsevičius 1988b; Silfverberg 1992, 2004; Pileckis and Monsevičius 1997; Jelínek and Audisio 2007; Audisio and Jelínek 2009; Ivinskis et al. 2009.

*oblonga* **(Herbst, 1793)** = *danica* Sjöberg, 1939. Silfverberg 1992, 2004; Pileckis and Monsevičius 1997; Jelínek and Audisio 2007; Audisio and Jelínek 2009.

[*opalizans* **J.R. Sahlberg, 1889**]. Known in Estonia, Denmark, throughout Sweden (Lundberg and Gustafsson 1995).

*pallescens* **(Stephens, 1835)** = *florea* Erichson, 1845 = *abietina* J.R. Sahlberg, 1889. # 60. Heyden 1903; Roubal 1910; Pileckis 1960, 1976a; Bercio and Folwaczny 1979; Silfverberg 1992, 2004; Pileckis and Monsevičius 1997; Jelínek and Audisio 2007; Audisio and Jelínek 2009.

*placida* **Mäklin, 1853**.Jelínek and Audisio 2007; Audisio and Jelínek 2009.

*pygmaea* **(Gyllenhal, 1808)**. Pileckis 1976a; Silfverberg 1992, 2004; Gaidienė 1993; Pileckis and Monsevičius 1997; Jelínek and Audisio 2007; Audisio and Jelínek 2009.

*rufomarginata* **(Stephens, 1832)**. Bercio and Folwaczny 1979; Silfverberg 1992, 2004; Šablevičius 2004; Jelínek and Audisio 2007; Audisio and Jelínek 2009

*silacea* **(Herbst, 1784)** = *deleta* (Erichson, 1843).Jelínek and Audisio 2007; Audisio and Jelínek 2009.

*silesiaca* **Reitter, 1872**.Monsevičius and Pankevičius 2001; Jelínek and Audisio 2007; Audisio and Jelínek 2009.

*terminalis* **(Mannerheim, 1843)** = *adumbrata* Mannerheim, 1852 = *tenenbaumi* Sjöberg, 1939. Roubal 1910; Pileckis 1960, 1976a; Bercio and Folwaczny 1979; Silfverberg 1992, 2004; Gaidienė 1993; Pileckis and Monsevičius 1997; Jelínek and Audisio 2007; Audisio and Jelínek 2009.

*thoracica* **Tournier, 1872**. Tamutis 2003; Jelínek and Audisio 2007; Audisio and Jelínek 2009.

*unicolor* **(Olivier, 1790)** = *x-rubrum* J.R. Sahlberg, 1911. Silfverberg 1992, 2004; Pileckis and Monsevičius 1997; Jelínek and Audisio 2007; Audisio and Jelínek 2009.

*variegata* **(Herbst, 1793)**. Monsevičius and Pankevičius 2001; Jelínek and Audisio 2007; Audisio and Jelínek 2009.

**Carpophilinae** **Erichson, 1842**.

*Carpophilus* **Stephens, 1830**.

[*dimidiatus* **(Fabricius, 1792)**]. Worldwide distributed, known in Denmark, Sweden, Estonia (Lundberg and Gustafsson 1995), Poland (Nunberg 1976).

*hemipterus* **(Linnaeus, 1758)**. Pileckis 1984; Silfverberg 1992, 2004; Pileckis and Monsevičius 1997; Audisio and Jelínek 2009.

[*ligneus* **Murray, 1864**]. Known in neotropical and nearctic regions, imported in Denmark, Sweden (Lundberg and Gustafsson 1995), Poland (Nunberg 1976).

*marginellus* **Motschulsky, 1858**.Audisio and Jelínek 2009.

*mutilatus* **Erichson, 1843**.Monsevičius and Pankevičius 2001.

**Meligethinae** **Thomson, 1859**. # 61.

*Acanthogethes* **Reitter, 1871**.

*solidus* **(Kugelann, 1794)**.Jelínek and Audisio 2007.

*Boragogethes* **Audisio & Cline, 2009**.

*symphyti* **(Heer, 1841)**. Tamutis 2003; Žiogas and Zolubas 2005; Jelínek and Audisio 2007; Audisio and Jelínek 2009.

*Afrogethes* **Audisio & Cline, 2009**.

[*planiusculus* **(Heer, 1841)**].Known in Denmark (Lundberg and Gustafsson 1995), Sweden (Silfverberg 2004), Poland (Burakowski et al. 1986b), Belarus (Audisio and Jelínek 2009).

*tristis* **(Sturm, 1845)**. Tamutis 2003; Jelínek and Audisio 2007; Audisio and Jelínek 2009.

*Genistogethes* **Audisio & Cline, 2009**.

[*bidentatus* **(Brisout, 1863)**]. Known in Denmark (Lundberg and Gustafsson 1995), northeastern Poland (Burakowski et al. 1986b), Belarus (Audisio and Jelínek 2009).

*carinulatus* **(Förster, 1849)** = *erythropus* auct. nec (Marsham, 1802).Tamutis 2003; Jelínek and Audisio 2007; Audisio and Jelínek 2009.

*Fabogethes* **Audisio & Cline, 2009**.

*nigrescens* **(Stephens, 1830)**. Tamutis 1999; 2003; Silfverberg 2004; Jelínek and Audisio 2007; Audisio and Jelínek 2009.

*Thymogethes* **Audisio & Cline, 2009**.

*egenus* **(Erichson, 1845)**. Tamutis 1999, 2003; Silfverberg 2004; Jelínek and Audisio 2007; Audisio and Jelínek 2009.

*exilis* **(Sturm, 1845)**.Jelínek and Audisio 2007; Audisio and Jelínek 2009.

*gagathinus* **(Erichson, 1845)**.Jelínek and Audisio 2007; Audisio and Jelínek 2009.

*lugubris* **(Sturm, 1845)**.Jelínek and Audisio 2007; Audisio and Jelínek 2009.

[*norvegicus* **(Easton 1959)**]. Known in southern Norway (Endrestøl et al. 2005), Kaliningrad region (Audisio personal comment).

*Sagittogethes* **Audisio & Cline, 2009**.

*distinctus* **(Sturm, 1845)** = *obscurus* auct. nec Erichson, 1845. Tamutis 2003; Jelínek and Audisio 2007.

[*hoffmanni* **(Reitter, 1871)**].Known in southern Sweden (Lundberg and Gustafsson 1995), Poland (Burakowski et al. 1986b), Belarus (Audisio and Jelínek 2009).

*maurus* **(Sturm, 1845)**. Tamutis 2003; Jelínek and Audisio 2007; Audisio and Jelínek 2009.

*ovatus* **(Sturm, 1845)**.Jelínek and Audisio 2007; Audisio and Jelínek 2009.

*umbrosus* **(Sturm, 1845)**.Jelínek and Audisio 2007; Audisio and Jelínek 2009.

*Astylogethes* **Kirejtshuk, 1979**.

[*caudatus* **(Guillebeau, 1897)**].Known in Latvia, Poland (Audisio and Jelínek 2009), Denmark, Sweden (Silfverberg 2004), Belarus (Tsinkevch and Alexandrovich 2002).

*corvinus* **(Erichson, 1845)**. Heyden 1903; Pileckis 1960, 1976a; Silfverberg 1992, 2004; Pileckis and Monsevičius 1997 (*Meligethes*); Jelínek and Audisio 2007; Audisio and Jelínek 2009.

*subrugosus* **(Gyllenhal, 1808)**. Tamutis 2003; Jelínek and Audisio 2007; Audisio and Jelínek 2009.

*Stachygethes* **Audisio & Cline, 2009**.

[*nanus* **Erichson, 1845**].Known in southern Sweden (Lundberg and Gustafsson 1995), Poland (Burakowski et al. 1986b), Belarus (Audisio and Jelínek 2009).

*ruficornis* **(Marsham, 1802)** = *flavipes* Sturm, 1845.Jelínek and Audisio 2007; Audisio and Jelínek 2009.

*Lamiogethes* **Audisio & Cline, 2009**.

[*atramentarius* (**Förster, 1849)**].Known in Latvia (Telnov 2004), Belarus (Alexandrovitch et al. 1996), Denmark, southern Sweden (Lundberg and Gustafsson 1995), northeastern Poland (Burakowski et al. 1986b), Kaliningrad region (Audisio and Jelínek 2009).

*bidens* **(Brisout, 1863)**. Tamutis and Zolubas 2001; Silfverberg 2004; Audisio and Jelínek 2009.

*brunnicornis* **(Sturm, 1845)**. Tamutis 2003; Jelínek and Audisio 2007; Audisio and Jelínek 2009.

*difficilis* **(Heer, 1841)**. Bercio and Folwaczny 1979; Silfverberg 1992, 2004; Jelínek and Audisio 2007; Audisio and Jelínek 2009.

*haemorrhoidalis* **(Förster, 1849)**. Pileckis 1976a; Silfverberg 1992, 2004; Pileckis and Monsevičius 1997 (*Meligethes*).

[*kunzei* **(Erichson, 1845)**].Known in Latvia (Telnov 2004), northern Poland (Burakowski et al. 1986b), Belarus (Audisio and Jelínek 2009).

*morosus* **(Erichson, 1845)**. Pileckis 1976a; Silfverberg 1992, 2004; Pileckis and Monsevičius 1997 (*Meligethes*); Jelínek and Audisio 2007; Audisio and Jelínek 2009.

*ochropus* **(Sturm, 1845)**. Tamutis 1999, 2003; Silfverberg 2004; Žiogas and Zolubas 2005; Jelínek and Audisio 2007; Audisio and Jelínek 2009.

*pedicularius* **(Gyllenhal, 1808)** = *viduatus* (Heer, 1841). Bercio and Folwaczny 1979; Monsevičius 1988b, 1997; Silfverberg 1992, 2004; Pileckis and Monsevičius 1997 (*Meligethes*); Jelínek and Audisio 2007; Audisio and Jelínek 2009; Ostrauskas and Ferenca 2010.

*persicus* **(Faldermann, 1837)**. Lundberg and Gustafsson 1995; Silfverberg 2004; Ostrauskas and Ferenca 2010.

*serripes* **(Gyllenhal, 1827)**.Jelínek and Audisio 2007; Audisio and Jelínek 2009.

*sulcatus* **(Brisout, 1863)**. Tamutis 2003; Jelínek and Audisio 2007; Audisio and Jelínek 2009.

*Meligethes* **Stephens, 1830**.

*atratus* **(Olivier, 1790)**. Audisio and Jelínek 2009.

*denticulatus* **(Heer, 1841)** = *hebes* Erichson, 1845. Pileckis and Jakaitis 1989; Silfverberg 1992, 2004; Gaidienė 1993; Pileckis and Monsevičius 1997 (*Meligethes*); Tamutis and Zolubas 2001; Šablevičius 2003a; Jelínek and Audisio 2007; Audisio and Jelínek 2009; Ivinskis et al. 2009; Ostrauskas and Ferenca 2010.

*flavimanus* **Stephens, 1830** = *lumbaris* Sturm, 1845. Jelínek and Audisio 2007; Audisio and Jelínek 2009.

*Brassicogethes* **Audisio & Cline, 2009**. 

*aeneus* **(Fabricius, 1775)**. Heyden 1903; Roubal 1910; Ogijewicz 1929, 1931, 1932; Pileckis 1960, 1976a; Lešinskas and Pileckis 1967; Zajančkauskas and Pileckis 1968; Pileckis and Vengeliauskaitė 1977, 1996; Pileckis et al. 1983, 1994b; Silfverberg 1992, 2004; Gaidienė 1993; Pileckis and Monsevičius 1997 (*Meligethes*); Monsevičius 1997; Tamutis 1999; Šablevičius 2000b, 2011; Gliaudys 2001; Šurkus and Gaurilčikienė 2002; Jelínek and Audisio 2007; Audisio and Jelínek 2009.

[*anthracinus* **(Brisout, 1863)**].Known in Estonia (Silfverberg 2004), Belarus (Audisio and Jelínek 2009), Sweden (Lundberg and Gustafsson 1995), Poland (Burakowski et al. 1986b).

*coeruleoivirens* **(Förster, 1849)**.Jelínek and Audisio 2007; Audisio and Jelínek 2009.

*coracinus* **(Sturm, 1845)**. Bercio and Folwaczny 1979; Silfverberg 1992, 2004; Jelínek and Audisio 2007; Audisio and Jelínek 2009; Ivinskis et al. 2009; Ostrauskas and Ferenca 2010.

*czwalinai* **(Reitter, 1871)**.Audisio and Jelínek 2009.

[*matronalis* **(Audisio & Spornraft, 1990)**].Known in Denmark, Sweden (Silfverberg 2004), Belarus (Audisio and Jelínek 2009).

*subaeneus* **(Sturm, 1845)**.Jelínek and Audisio 2007; Audisio and Jelínek 2009.

*viridescens* **(Fabricius, 1787)**. Ogyjewicz 1931, 1938; Pileckis 1960, 1976a; Silfverberg 1992, 2004; Pileckis et al. 1994b; Pileckis and Monsevičius 1997 (*Meligethes*); Monsevičius 1997; Jelínek and Audisio 2007; Audisio and Jelínek 2009; Ostrauskas and Ferenca 2010.

*Pria* **Stephens, 1830** = *Laria* Scopoli, 1763.

*dulcamarae* **(Scopoli, 1763)**. Silfverberg 1992, 2004; Gaidienė 1993; Pileckis and Monsevičius 1997; Jelínek and Audisio 2007.

**Nitidulinae** **Latreille, 1802**.

**Nitidulini Latreille, 1802**.

*Omosita* **Erichson, 1843**.

*colon* **(Linnaeus, 1758)**. Roubal 1910; Pileckis 1960, 1976a; Silfverberg 1992, 2004; Gaidienė 1993; Pileckis and Monsevičius 1997; Ferenca 2006b; Jelínek and Audisio 2007; Alekseev 2008a; Audisio and Jelínek 2009.

*depressa* **(Linnaeus, 1758)**. Heyden 1903; Pileckis 1960, 1976a; Lešinskas and Pileckis 1967; Bercio and Folwaczny 1979; Silfverberg 1992, 2004; Gaidienė 1993; Monsevičius 1997; Pileckis and Monsevičius 1997; Šablevičius 2000b, 2003b, 2011; Jelínek and Audisio 2007; Audisio and Jelínek 2009.

*discoidea* (**Fabricius, 1775)**. Heyden 1903; Pileckis 1960, 1976a; Bercio and Folwaczny 1979; Silfverberg 1992, 2004; Pileckis and Monsevičius 1997; Ferenca 2006b; Jelínek and Audisio 2007.

*Nitidula* **Fabricius, 1775**.

*bipunctata* **(Linnaeus, 1758)**. Eichwald 1830; Pileckis 1960, 1963b, 1976a; Lešinskas and Pileckis 1967; Silfverberg 1992, 2004; Gaidienė 1993; Pileckis and Monsevičius 1997; Šablevičius 2003a, 2011; Jelínek and Audisio 2007; Audisio and Jelínek 2009.

*carnaria* **(Schaller, 1783)**. Pileckis 1960, 1976a; Bercio and Folwaczny 1979; Silfverberg 1992, 2004; Gaidienė 1993; Pileckis and Monsevičius 1997; Ferenca et al. 2006, 2007; Jelínek and Audisio 2007; Alekseev 2008a; Audisio and Jelínek 2009.

*rufipes* **(Linnaeus, 1767)**. Pileckis 1960, 1976a; Silfverberg 1992, 2004; Gaidienė 1993; Pileckis and Monsevičius 1997; Šablevičius 2004; Šablevičius 2011; Ferenca et al. 2006, 2007; Jelínek and Audisio 2007; Audisio and Jelínek 2009.

*Amphotis* **Erichson, 1843**.

*marginata* **(Fabricius, 1781)**.Pileckis 1960, 1976a; Silfverberg 1992, 2004; Gaidienė 1993; Pileckis and Monsevičius 1997; Jelínek and Audisio 2007; Audisio and Jelínek 2009; Ivinskis et al. 2009.

*Soronia* **Erichson, 1843**.

*grisea* **(Linnaeus, 1758)**. Monsevičius 1988b; Silfverberg 1992, 2004; Gaidienė 1993; Pileckis and Monsevičius 1997; Šablevičius 2003a, 2011; Butvila et al. 2007; Jelínek and Audisio 2007; Audisio and Jelínek 2009; Ivinskis et al. 2009.

*punctatissima* **(Illiger, 1794)**. Roubal 1910; Pileckis 1960, 1976a; Pileckis and Monsevičius 1982, 1997; Silfverberg 1992, 2004; Gaidienė 1993; Šablevičius 2003a; Jelínek and Audisio 2007; Audisio and Jelínek 2009; Ivinskis et al. 2009.

*Ipidia* **Erichson, 1843**.

*binotata* **Reitter, 1875** = *quadriplagiata* Biström, 1978 = *quadrimaculata* (Quensel, 1790) nec (Scopoli, 1772) = *quadrinotata* (Fabricius, 1798) nec (Scriba, 1793). Pileckis 1960, 1976a; Silfverberg 1992, 2004; Monsevičius 1997; Pileckis and Monsevičius 1997; Šablevičius 2003a, 2011; Ferenca 2004, 2006b; Jelínek and Audisio 2007; Vaivilavičius 2008; Audisio and Jelínek 2009; Ivinskis et al. 2009.

*sexguttata **(R.F. Sahlberg, 1834)**. # 62. Wankowicz 1867a; Łomnicki 1913; Pileckis 1968b, 1976a; Silfverberg 1992, 2004; Pileckis and Monsevičius 1997.

*Pocadius* **Erichson, 1843**.

*adustus* **Reitter, 1888**.Audisio and Jelínek 2009.

*ferrugineus* **(Fabricius, 1775)** = *striatus* Olivier, 1790. Ferenca 1988, 2004; Silfverberg 1992, 2004; Gaidienė 1993; Monsevičius 1997; Pileckis and Monsevičius 1997; Šablevičius 2003a, 2011; Jelínek and Audisio 2007; Audisio and Jelínek 2009.

*Physoronia* **Reitter 1884** = *Pocadiodes* Ganglbauer, 1899.

*wajdelota* **(Wankowicz, 1869)**. Wankowicz, 1869; Roubal 1910; Horion 1960; Pileckis 1960, 1976a; Silfverberg 1992, 2004; Gaidienė 1993; Pileckis and Monsevičius 1997; Šablevičius 2003a; Jelínek and Audisio 2007.

*Thalycra* **Erichson, 1843**.

*fervida* **(Olivier, 1790)**. Bercio and Folwaczny 1979; Silfverberg 1992, 2004; Gaidienė 1993; Pileckis and Monsevičius 1997; Ferenca 2006b; Jelínek and Audisio 2007; Audisio and Jelínek 2009.

**Cyllodini Everts, 1898**.

*Cyllodes* **Erichson, 1843**.

*ater* **(Herbst, 1792)**. Jakaitis 1985; Gaidienė 1993; Pileckis and Monsevičius 1997; Tamutis and Zolubas 2001; Silfverberg 2004; Ferenca 2004; Vaivilavičius 2008; Audisio and Jelínek 2009.

**Cychramini Gistel, 1848**.

*Cychramus* **Kugelann, 1794**.

*luteus* **(Fabricius, 1787)**. Roubal 1910; Pileckis 1960, 1976a; Silfverberg 1992, 2004; Gaidienė 1993; Monsevičius 1997; Pileckis and Monsevičius 1997; Šablevičius 2000b, 2011; Ferenca 2006b; Jelínek and Audisio 2007; Audisio and Jelínek 2009; Ostrauskas and Ferenca 2010.

*variegatus* **(Herbst, 1792)** = *quadripunctatus* (Herbst, 1792). Roubal 1910; Pileckis 1960, 1976a; Silfverberg 1992, 2004; Gaidienė 1993; Pileckis and Monsevičius 1997; Jelínek and Audisio 2007; Audisio and Jelínek 2009; Šablevičius 2011.

**Cryptarchinae** **Thomson, 1859**.

**Cryptarchini Thomson, 1859**.

*Cryptarcha* **Shuckard, 1839**.

*strigata* **(Fabricius, 1787)**. Miländer et al. 1984; Silfverberg 1992, 2004; Gaidienė 1993; Pileckis and Monsevičius 1997; Ferenca et al. 2002; Jelínek and Audisio 2007; Audisio and Jelínek 2009; Šablevičius 2011.

*undata* **(Olivier, 1790)**. Monsevičius 1988b, 1997; Silfverberg 1992, 2004; Pileckis and Monsevičius 1997; Ferenca et al. 2006, 2007; Jelínek and Audisio 2007; Ivinskis et al. 2009.

*Glischrochilus* **Reitter 1873** = *Librador* Reitter, 1874.

*grandis* **(Tournier, 1872)**. Ferenca et al. 2006, 2007; Tamutis and Ferenca 2006.

*hortensis* **(Geoffroy, 1785)**. Heyden 1903; Roubal 1910; Pileckis 1960, 1976a; Silfverberg 1992, 2004; Gaidienė 1993; Pileckis and Monsevičius 1997; Šablevičius 2000b; Gliaudys 2001; Tamutis and Zolubas 2001; Jelínek and Audisio 2007; Audisio and Jelínek 2009.

*quadriguttatus* **(Fabricius, 1777)**. Silfverberg 1992, 2004; Pileckis and Monsevičius 1997; Barševskis 2001a; Jelínek and Audisio 2007; Audisio and Jelínek 2009.

*quadripunctatus* **(Linnaeus, 1758)**. Heyden 1903; Pileckis 1960, 1976a, 1982; Lešinskas and Pileckis 1967; Silfverberg 1992, 2004; Gaidienė 1993; Pileckis and Monsevičius 1997; Šablevičius 2000b, 2011; Gliaudys 2001; Ferenca 2006b; Jelínek and Audisio 2007; Audisio and Jelínek 2009.

*quadrisignatus* **(Say, 1835)**. Ferenca et al. 2006, 2007; Tamutis and Ferenca 2006; Ivinskis et al. 2009.

*Pityophagus* **Shuckard, 1839**.

*ferrugineus* **(Linnaeus, 1761)**. Pileckis 1960, 1976a; Jakaitis and Valenta 1976; Zajančkauskas and Pileckis 1968; Silfverberg 1992, 2004; Gaidienė 1993; Monsevičius 1997; Pileckis and Monsevičius 1997; Tamutis and Zolubas 2001; Šablevičius 2003a, 2004; Inokaitis 2004; Ferenca 2006b; Jelínek and Audisio 2007; Audisio and Jelínek 2009; Zeniauskas and Gedminas 2010.

**Cybocephalinae Jacquelin du Val, 1858**.

*Cybocephalus* **Erichson, 1844**.

[*politus* **(Gyllenhal, 1813)**].Known in Latvia (Telnov 2004), Estonia, Denmark, Sweden (Lundberg and Gustafsson 1995), northeastern Poland (Burakowski et al. 1986b), recently found in Kaliningrad region (Alekseev and Bukejs 2010).

**BOTHRIDERIDAE Erichson, 1845**.

**Teredinae** **Seidlitz, 1888**.

**Teredini Seidlitz, 1888**.

*Teredus* **Dejean, 1835**.

*cylindricus* **(Olivier, 1790)** Ślipinski 2009.

[*opacus* **Habelmann, 1854**]. Known in northern Poland (Burakowski et al. 1986c).

*Oxylaemus* **Erichson, 1845**.

[*cylindricus* **(Panzer, 1796)**].Known in northern Poland (Burakowski et al. 1986c), Denmark (Ślipinski 2009).

[*variolosus* **(Dufour, 1843)**].Known in northern Poland (Burakowski et al. 1986c), Sweden (Lundberg and Gustafsson 1995).

**Anommatinae** **Ganglbauer, 1899**.

*Anommatus* **Wesmael, 1835**.

[*diecki* **Reitter, 1875**].Known in Sweden (Lundberg and Gustafsson1995), Denmark (Olberg and Olsen 2009).

[*duodecimstriatus* (**O.F. Müller, 1821)**].Known in Denmark, Sweden (Lundberg and Gustafsson1995), Poland (Burakowski et al. 1986c). 

**Bothriderinae** **Erichson, 1845**. (Colydiidae)

*Bothrideres* **Dejean, 1835**.

*contractus* **(Geoffroy, 1785)** = *bipunctatus* (Gmelin, 1790). Ferenca 1988; Silfverberg 1992, 2004; Monsevičius 1997; Pileckis and Monsevičius 1997; Ferenca 2004; Ferenca et al. 2006, 2007; Ślipinski 2009.

**CERYLONIDAE Billberg, 1820**.

**Ceryloninae** **Billberg, 1820**.

*Cerylon* **Latreille, 1802**.

*deplanatum* **Gyllenhal, 1827**. Monsevičius 1998; Silfverberg 2004; Ślipinski 2007.

*fagi* **Brisout, 1867**. Monsevičius 1988b, 1997; Silfverberg 1992, 2004; Pileckis and Monsevičius 1997; Ślipinski 2007.

*ferrugineum* **Stephens, 1830**. Roubal 1910; Pileckis 1960, 1976a; Silfverberg 1992, 2004; Gaidienė 1993; Pileckis and Monsevičius 1997; Tamutis and Zolubas 2001; Ślipinski 2009.

*histeroides* **(Fabricius, 1793)**. Roubal 1910; Mazurowa and Mazur 1939; Pileckis 1960, 1976a, 1982; Bercio and Folwaczny 1979; Silfverberg 1992, 2004; Gaidienė 1993; Monsevičius 1997; Pileckis and Monsevičius 1997; Šablevičius 2000b; Ślipinski 2009.

*impressum* **Erichson, 1845**. Pileckis 1976a; Silfverberg 1992, 2004; Gaidienė 1993; Monsevičius 1997; Pileckis and Monsevičius 1997; Ślipinski 2009; Ostrauskas and Ferenca 2010.

**ALEXIIDAE Imhoff, 1856** = SPHAEROSOMATIDAE Ganglbauer, 1899.

*Sphaerosoma* **Stephens, 1832**.

*pilosum* **(Panzer, 1793)**. Šablevičius 2000a, 2003a.

**ENDOMYCHIDAE Leach, 1815**.

**Merophysiinae** **Seidlitz, 1872**.

*Holoparamecus* **Curtis, 1833**.

[*caularum* **(Aubé, 1843)**].Known in Denmark, Sweden (Silfverberg 2004), Poland (Shockley et al. 2009).

**Leiestinae** **Thomson, 1863**.

*Leiestes* **Chevrolat, 1837**.

*seminiger* **(Gyllenhal, 1808)**. Silfverberg 1992, 2004; Pileckis and Monsevičius 1997; Rücker et al. 2007; Tomaszewska 2009; Šablevičius 2011.

**Mycetaeinae** **Jacquelin du Val, 1857**.

*Mycetaea* **Stephens, 1830**.

*subterranea* **(Fabricius, 1801)** = *hirta* (Marsham, 1802) nec (Schaeffer, 1769). Monsevičius and Pankevičius 2001; Šablevičius 2003a; Rücker et al. 2007; Tomaszewska 2009.

**Endomychinae** **Leach, 1815**.

*Endomychus* **Panzer, 1795**.

*coccineus* **(Linnaeus, 1758)**. Pileckis 1960, 1976a; Silfverberg 1992, 2004; Gaidienė 1993; Monsevičius 1997; Pileckis and Monsevičius 1997; Šablevičius 2000b, 2011; Rücker et al. 2007; Tomaszewska 2009; Ostrauskas and Ferenca 2010.

**Lycoperdininae** **Bromhead, 1838**.

*Lycoperdina* **Latreille, 1807**.

[*bovistae* **(Fabricius, 1793)**].Known in Denmark, Sweden (Lundberg and Gustafsson1995), northern Poland (Burakowski et al. 1986c).

*succincta* **(Linnaeus, 1767)**. Slavinskas 1982; Silfverberg 1992, 2004; Pileckis and Monsevičius 1997; Rücker et al. 2007; Tomaszewska 2009; Šablevičius 2011.

*Mycetina* **Mulsant, 1846**.

*cruciata* **(Schaller, 1783)**. Monsevičius 1988b, 1997; Silfverberg 1992, 2004; Pileckis and Monsevičius 1997; Šablevičius 2000a, 2001, 2003a, 2011; Rücker et al. 2007; Tomaszewska 2009; Ostrauskas and Ferenca 2010.

**COCCINELLIDAE Latreille, 1807**.

**Coccinellinae** **Latreille, 1807**.

**Coccidulini Mulsant, 1846**.

*Coccidula* **Kugelann, 1798**.

*scutellata* **(Herbst, 1783)**. Pileckis 1960, 1963b, 1976a, 1982; Zajančkauskas and Pileckis 1968; Silfverberg 1992, 2004; Monsevičius 1997; Pileckis and Monsevičius 1997; Ferenca 2006b; Alekseev 2008b; Šablevičius 2011.

*rufa* **(Herbst, 1783)**. Gaidienė 1993; Pileckis and Monsevičius 1997; Šablevičius 2003a; Silfverberg 2004; Kovář 2007; Alekseev 2008b; Ivinskis et al. 2009.

*Rhyzobius* **Stephens, 1832**.

[*chrysomeloides* **(Herbst, 1792)**]. Known in Denmark (Lundberg and Gustafsson1995), northern Poland (Burakowski et al. 1986c).

[*litura* **(Fabricius, 1787)**]. Known in Denmark, Sweden (Lundberg and Gustafsson1995), northern Poland (Burakowski et al. 1986c).

**Stethorini Dobzhansky, 1924**.

*Stethorus* **Weise, 1885**.

*punctillum* **Weise, 1891**. Heyden 1903; Pileckis 1960, 1976a; Silfverberg 1992, 2004; Pileckis and Monsevičius 1997; Šablevičius 2003a, 2011; Alekseev 2008b.

**Scymnini Mulsant, 1846**.

*Scymnus* **Kugelann, 1794.**

*abietis* **(Paykull, 1798)**. Pileckis 1976a; Silfverberg 1992, 2004; Pileckis and Monsevičius 1997; Kovář 2007; Alekseev 2008b.

*ater* **Kugelann, 1794**. Kovář 2007.

*auritus* **Thunberg, 1795**. Bercio and Folwaczny 1979; Silfverberg 1992, 2004; Kovář 2007; Alekseev 2008b.

[*femoralis* **(Gyllenhal, 1827)**].Known in Latvia (Telnov 2004), Denmark, throughout Sweden (Lundberg and Gustafsson 1995).

*ferrugatus* **(Moll, 1785)**. Pileckis 1960, 1976a; Silfverberg 1992, 2004; Gaidienė 1993; Pileckis and Monsevičius 1997; Šablevičius 2003b; Alekseev 2008b.

*frontalis* **(Fabricius, 1787)**. Pileckis 1976a; Silfverberg 1992, 2004; Gaidienė 1993; Pileckis and Monsevičius 1997; Šablevičius 2000a, 2003a, 2011; Kovář 2007; Alekseev 2008a, b; Ivinskis et al. 2009.

*haemorrhoidalis* **Herbst, 1797**. Tamutis 2003; Žiogas and Zolubas 2005; Kovář 2007; Alekseev 2008b; Ostrauskas and Ferenca 2010.

*limbatus* **Stephens, 1832**. Kovář 2007.

*mimulus* **Capra & Fürsch, 1967** = *rufipes* auct. nec (Fabricius, 1798). Bercio and Folwaczny 1979; Silfverberg 1992, 2004; Alekseev 2008b.

*nigrinus* **Kugelann, 1794**. Pileckis and Monsevičius 1997; Šablevičius 2003b; Silfverberg 2004; Kovář 2007; Alekseev 2008b.

[*pallipediformis apetzoides* **Capra & Fürsch, 1965**]. Known in Latvia (Telnov 2004), Sweden (Lundberg and Gustafsson 1995), Poland (Burakowski et al. 1986c).

*rubromaculatus* **(Goeze, 1777)**. Pileckis and Monsevičius 1997; Šablevičius 2000a, 2003a; Silfverberg 2004; Alekseev 2008b.

[*silesiacus* **Weise, 1902** = *stiglundbergi* Fürsch, 1969].Known in Sweden (Lundberg and Gustafsson 1995), Poland (Burakowski et al. 1986c).

*suturalis* **Thunberg, 1795**. Heyden 1903; Pileckis 1960, 1976a; Bercio and Folwaczny 1979; Silfverberg 1992, 2004; Gaidienė 1993; Monsevičius 1997; Pileckis and Monsevičius 1997; Šablevičius 2003a, b, 2011; Žiogas and Zolubas 2005; Kovář 2007; Alekseev 2008b; Canepari 2009.

*Nephus* **Mulsant, 1846**.

*bipunctatus* **(Kugelann, 1794)**. Pilckis 1976a; Silfverberg 1992, 2004; Pileckis and Monsevičius 1997 (*Scymnus*); Kovář 2007; Alekseev 2008b.

[*bisignatus* **(Boheman, 1850)**]. Known in Denmark, Sweden (Lundberg and Gustafsson1995), northern Poland (Canepari 2009).

*quadrimaculatus* **(Herbst, 1783)**. Pileckis and Monsevičius 1997 (*Scymnus*); Silfverberg 2004; Alekseev 2008b.

*redtenbacheri redtenbacheri* **Mulsant, 1846**. Gaidienė 1993; Pileckis and Monsevičius 1997 (*Scymnus*); Šablevičius 2003a; Silfverberg 2004; Kovář 2007; Alekseev 2008b.

**Hyperaspidini Mulsant, 1846.**

*Hyperaspis* **Chevrolat, 1837**.

*campestris* **(Herbst, 1783)**. Pileckis 1960, 1970a, 1976a; Silfverberg 1992, 2004; Gaidienė 1993; Pileckis and Monsevičius 1997; Šablevičius 2003a; Ferenca 2006b; Kovář 2007; Alekseev 2008a, b.

*pseudopustulata* **Mulsant, 1853** = *reppensis* auct. nec (Herbst, 1783). Pileckis and Monsevičius 1997; Šablevičius 2000a, 2003a; Silfverberg 2004; Kovář 2007; Alekseev 2008b.

**Platynaspini Mulsant, 1846**.

*Platynaspis* **Redtenbacher, 1843**.

*luteorubra* **(Goeze, 1777)**. Pileckis 1960, 1976a; Silfverberg 1992, 2004; Pileckis and Monsevičius 1997; Šablevičius 2003a; Ferenca 2006b; Ferenca et al. 2006, 2007; Kovář 2007; Alekseev 2008b.

**Chilochorini Mulsant, 1846**.

*Chilocorus* **Leach, 1815**.

*bipustulatus* **(Linnaeus, 1758)**. Pileckis 1960, 1976a; Zajančkauskas and Pileckis 1968; Silfverberg 1992, 2004; Gaidienė 1993; Monsevičius 1997; Pileckis and Monsevičius 1997; Ferenca 2006b; Kovář 2007; Alekseev 2008b.

*renipustulatus* **(Scriba, 1790)**. Pileckis 1960, 1976a, 1982; Lešinskas and Pileckis 1967; Zajančkauskas and Pileckis 1968; Silfverberg 1992, 2004; Gaidienė 1993; Monsevičius 1997; Pileckis and Monsevičius 1997; Gliaudys 2001; Ferenca 2006b; Alekseev 2008b; Šablevičius 2011.

*Exochomus* **Redtenbacher, 1843**.

*nigromaculatus* **(Goeze, 1777)** = *flavipes* (Thunberg, 1781). Pileckis 1968b, 1976a; Silfverberg 1992, 2004; Gaidienė 1993; Pileckis and Monsevičius 1997; Alekseev 2008b.

*quadripustulatus* **(Linnaeus, 1758)**. Pileckis 1960, 1976a, 1982; Zajančkauskas and Pileckis 1968; Silfverberg 1992, 2004; Gaidienė 1993; Monsevičius 1997; Pileckis and Monsevičius 1997; Šablevičius 2000b; Šablevičius 2011; Ferenca 2006b; Kovář 2007; Alekseev 2008b.

**Coccinellini Latreille, 1807**.

*Coccinula* **Dobzhansky, 1925**.

*quatuordecimpustulata* **(Linnaeus, 1758)**. Pileckis 1960, 1976a; Lešinskas and Pileckis 1967; Zajančkauskas and Pileckis 1968; Silfverberg 1992, 2004; Gaidienė 1993; Monsevičius 1997; Pileckis and Monsevičius 1997; Šablevičius 2000b, 2011; Ferenca 2006b; Kovář 2007; Alekseev 2008b; Canepari 2009.

*Anisosticta* **Chevrolat, 1837**.

*novemdecimpunctata* **(Linnaeus, 1758)**. Pileckis 1960, 1976a; Silfverberg 1992, 2004; Gaidienė 1993; Monsevičius 1997; Pileckis and Monsevičius 1997; Šablevičius 2000b; Gliaudys 2001; Ferenca 2006b; Kovář 2007; Alekseev 2008b.

*Sospita* **Mulsant, 1846**.

*vigintiguttata* **(Linnaeus, 1758)**. Pileckis 1960, 1976a; Silfverberg 1992, 2004; Gaidienė 1993; Pileckis and Monsevičius 1997; Ferenca et al. 2002; Ferenca 2006b; Kovář 2007; Vaivilavičius 2008; Alekseev 2008b; Šablevičius 2011.

*Myzia* **Mulsant, 1846** = *Neomyzia* Casey, 1899.

*oblongoguttata* **(Linnaeus, 1758)**. Pileckis 1960, 1976a; Zajančkauskas and Pileckis 1968; Silfverberg 1992, 2004; Gaidienė 1993; Monsevičius 1997; Pileckis and Monsevičius 1997; Gliaudys 2001; Žiogas and Zolubas 2005; Ferenca 2006b; Kovář 2007; Alekseev 2008b; Šablevičius 2011.

*Myrrha* **Mulsant, 1846**.

*octodecimguttata* **(Linnaeus, 1758)**. Pileckis 1960, 1976a; Silfverberg 1992, 2004; Gaidienė 1993; Monsevičius 1997; Pileckis and Monsevičius 1997; Gliaudys 2001; Kovář 2007; Alekseev 2008b.

*Propylea* **Mulsant, 1846**.

*quatuordecimpunctata* **(Linnaeus, 1758)**. Heyden 1903; Mazurowa and Mazur 1939; Pileckis 1960, 1976a, 1982; Zajančkauskas and Pileckis 1968; Silfverberg 1992, 2004; Gaidienė 1993; Monsevičius 1997; Monsevičius 1997; Pileckis and Monsevičius 1997; Šablevičius 2000b, 2011; Ferenca 2006b; Alekseev 2008b; Canepari 2009.

*Calvia* **Mulsant, 1846**.

*decemguttata* **(Linnaeus, 1767)**. Pileckis 1960, 1976a; Silfverberg 1992, 2004; Gaidienė 1993; Monsevičius 1997; Pileckis and Monsevičius 1997; Ferenca 2006b; Kovář 2007; Alekseev 2008b; Ivinskis et al. 2009.

*quatuordecimguttata* **(Linnaeus, 1758)**. Heyden 1903; Mazurowa and Mazur 1939; Pileckis 1960, 1976a, 1982; Zajančkauskas and Pileckis 1968; Silfverberg 1992, 2004; Gaidienė 1993; Monsevičius 1997; Pileckis and Monsevičius 1997; Šablevičius 2000b; Gliaudys 2001; Ferenca 2006b; Kovář 2007; Alekseev 2008b; Canepari 2009.

*quindecimguttata* **(Fabricius, 1777)**. Gaidienė 1993; Silfverberg 2004; Alekseev 2008b.

*Anatis* **Mulsant, 1846**.

*ocellata* **(Linnaeus, 1758)**. Pileckis 1960, 1976a; Zajančkauskas and Pileckis 1968; Silfverberg 1992, 2004; Gaidienė 1993; Monsevičius 1997; Pileckis and Monsevičius 1997; Šablevičius 2000b, 2011; Gliaudys 2001; Tamutis and Zolubas 2001; Žiogas and Zolubas 2005; Ferenca 2006b; Kovář 2007; Alekseev 2008b.

*Aphidecta* **Weise, 1893**.

*obliterata* **(Linnaeus, 1758)**. Pileckis 1960, 1970a, 1976a; Silfverberg 1992, 2004; Gaidienė 1993; Monsevičius 1997; Pileckis and Monsevičius 1997; Žiogas and Zolubas 2005; Ferenca 2006b; Kovář 2007; Alekseev 2008b; Ostrauskas and Ferenca 2010.

*Hippodamia* **Chevrolat, 1837** = *Adonia* Mulsant, 1846 = *Semiadalia* Crotch, 1874.

*notata* **(Laicharting, 1781)**. Pileckis 1968a, 1976a; Silfverberg 1992, 2004; Gaidienė 1993; Pileckis and Monsevičius 1997; Kovář 2007; Alekseev 2008b; Ivinskis et al. 2009.

*septemmaculata* **(DeGeer, 1775)**. Pileckis 1976a; Silfverberg 1992, 2004; Pileckis and Monsevičius 1997; Kovář 2007; Alekseev 2008b.

*tredecimpunctata* **(Linnaeus, 1758)**. Pileckis 1960, 1976a, 1982; Lešinskas and Pileckis 1967; Zajančkauskas and Pileckis 1968; Silfverberg 1992, 2004; Gaidienė 1993; Monsevičius 1997; Pileckis and Monsevičius 1997; Šablevičius 2000b; Ferenca 2006b; Kovář 2007; Alekseev 2008b; Canepari 2009.

[*undecimnotata* **(Schneider, 1792)**].Known in Latvia (Telnov 2004), Belarus (Alexandrovitch et al. 1996), Poland (Canepari 2009).

*variegata* **(Goeze, 1777)**. Heyden 1903; Pileckis 1960, 1976a, 1982; Silfverberg 1992, 2004; Gaidienė 1993; Pileckis and Monsevičius 1997; Šablevičius 2000b; Gliaudys 2001; Ferenca 2006b; Kovář 2007; Alekseev 2008b; Canepari 2009.

*Coccinella* **Linnaeus, 1758**.

*hieroglyphica* **Linnaeus, 1758**. Mazurowa and Mazur 1939; Pileckis 1960, 1963b; 1976a; Lešinskas and Pileckis 1967; Zajančkauskas and Pileckis 1968; Silfverberg 1992, 2004; Gaidienė 1993; Monsevičius 1997; Pileckis and Monsevičius 1997; Šablevičius 2000b; Ferenca 2006b; Kovář 2007; Alekseev 2008b.

*magnifica* **Redtenbacher, 1843** = *divaricata* auct. nec Olivier, 1808 = *distincta* Faldermann, 1837, nec Herbst, 1793. Mazurowa and Mazur 1939; Pileckis 1960, 1976a; Silfverberg 1992, 2004; Gaidienė 1993; Pileckis and Monsevičius 1997; Kovář 2007; Alekseev 2008b.

*quinquepunctata* **Linnaeus, 1758**. Heyden 1903; Mazurowa and Mazur 1939; Pileckis 1960, 1976a, 1982; Lešinskas and Pileckis 1967; Zajančkauskas and Pileckis 1968; Silfverberg 1992, 2004; Gaidienė 1993; Monsevičius 1997; Pileckis and Monsevičius 1997; Šablevičius 2000b; Gliaudys 2001; Tamutis and Zolubas 2001; Ferenca 2006b; Kovář 2007; Alekseev 2008b.

[*saucerottii lutshniki* **Dobzhansky, 1917**]. Known in Latvia (Telnov 2004), Belarus (Alexandrovitch et al. 1996), Poland (Burakowski et al. 1986c).

*septempunctata* **Linnaeus, 1758**. Heyden 1903; Pileckis 1960, 1976a, 1982; Lešinskas and Pileckis 1967; Zajančkauskas and Pileckis 1968; Silfverberg 1992, 2004; Gaidienė 1993; Monsevičius 1997; Pileckis and Monsevičius 1997; Šablevičius 2000b; Gliaudys 2001; Ferenca 2006b; Kovář 2007; Alekseev 2008b; Canepari 2009.

*undecempunctata* **Linnaeus, 1758**. Pileckis 1963b, 1970a, 1976a; Silfverberg 1992, 2004; Gaidienė 1993; Pileckis and Monsevičius 1997; Alekseev 2008b.

*Oenopia* **Mulsant, 1850** = *Synharmonia* Ganglbauer, 1899.

*conglobata* **Linnaeus, 1758**. Heyden 1903; Pileckis 1960, 1976a; Silfverberg 1992, 2004; Gaidienė 1993; Pileckis and Monsevičius 1997; Gliaudys 2001; Ferenca 2006b; Kovář 2007; Alekseev 2008b.

*Adalia* **Mulsant, 1850**.

*bipunctata* **(Linnaeus, 1758)**. Heyden 1903; Pileckis 1960, 1976a, 1982; Lešinskas and Pileckis 1967; Zajančkauskas and Pileckis 1968; Silfverberg 1992, 2004; Gaidienė 1993; Monsevičius 1997; Pileckis and Monsevičius 1997; Šablevičius 2000b; Gliaudys 2001; Ferenca 2006b; Kovář 2007; Alekseev 2008b; Canepari 2009; Ostrauskas and Ferenca 2010.

*conglomerata* **(Linnaeus, 1758)**. Monsevičius 1988b; Silfverberg 1992, 2004; Pileckis and Monsevičius 1997; Alekseev 2008b.

*decempunctata* **(Linnaeus, 1758)**. Heyden 1903; Pileckis 1960, 1976a, 1982; Zajančkauskas and Pileckis 1968; Silfverberg 1992, 2004; Gaidienė 1993; Monsevičius 1997; Pileckis and Monsevičius 1997; Šablevičius 2000b; Kovář 2007; Alekseev 2008b; Canepari 2009.

*Harmonia* **Mulsant, 1846**.

[*axyridis* **(Pallas, 1773)**]. Recently found in Latvia (Barševskis 2009).

*quadripunctata* **(Pontoppidan, 1763)**. Pileckis 1976a; Silfverberg 1992, 2004; Gaidienė 1993; Pileckis and Monsevičius 1997; Kovář 2007; Alekseev 2008b.

*Vibidia* **Mulsant, 1846**.

*duodecimguttata* **(Poda, 1761)**. Pileckis 1976a; Silfverberg 1992, 2004; Pileckis and Monsevičius 1997; Kovář 2007; Alekseev 2008b; Canepari 2009.

*Halyzia* **Mulsant, 1846**.

*sedecimguttata* **(Linnaeus, 1758)**. Pileckis 1960, 1976a; Zajančkauskas and Pileckis 1968; Bercio and Folwaczny 1979; Silfverberg 1992, 2004; Gaidienė 1993; Monsevičius 1997; Pileckis and Monsevičius 1997; Gliaudys 2001; Ferenca 2006b; Kovář 2007; Alekseev 2008b; Canepari 2009.

*Psyllobora* **Chevrolat, 1837** = *Thea* Mulsant, 1846.

*vigintiduopunctata* **(Linnaeus, 1758)**.Heyden 1903; Pileckis 1960, 1976a; Silfverberg 1992, 2004; Gaidienė 1993; Monsevičius 1997; Pileckis and Monsevičius 1997; Semaškienė et al. 1999; Šablevičius 2000b, 2011; Gliaudys 2001; Ferenca 2006b; Kovář 2007; Alekseev 2008b; Canepari 2009.

*Tytthaspis* **Crotch, 1874**.

*sedecimpunctata* **(Linnaeus, 1761)**. Pileckis 1968a, 1976a; Silfverberg 1992, 2004; Gaidienė 1993; Monsevičius 1997; Pileckis and Monsevičius 1997; Kovář 2007; Alekseev 2008b; Canepari 2009.

**Epilachnini Mulsant, 1846**.

*Henosepilachna* **Li, 1961**.

*vigintioctomaculata **(Motschulsky, 1857)**. # 63. Šablevičius 2000b, 2011.

*Subcoccinella* **Agassiz, 1846**.

*vigintiquatuorpunctata* **(Linnaeus, 1758)**. Roubal 1910; Mazurowa and Mazur 1939; Pileckis 1960, 1976a; Lešinskas and Pileckis 1967; Pileckis and Vengeliauskaitė 1977, 1996; Silfverberg 1992, 2004; Gaidienė 1993; Monsevičius 1997; Pileckis and Monsevičius 1997; Šablevičius 2000b, 2011; Ferenca 2006b; Kovář 2007; Alekseev 2008b.

**Cynegetini Thomson, 1866**.

*Cynegetis* **Chevrolat, 1837**.

[*impunctata* **(Linnaeus, 1761)**]. Known in Latvia (Telnov 2004), Denmark, Sweden (Lundberg and Gustafsson1995), northern Poland (Burakowski et al. 1986c).

**CORYLOPHIDAE LeConte, 1852**.

**Corylophinae** **LeConte, 1852**.

**Corylophini LeConte, 1852**.

*Corylophus* **Stephens, 1835**.

*cassidoides* **(Marsham, 1802)**. Pileckis and Monsevičius 1997; Silfverberg 2004; Alekseev 2008a.

**Sericoderini Matthews, 1888**.

*Sericoderus* **Stephens, 1829**.

*lateralis* **(Gyllenhal, 1827)**. Silfverberg 1992, 2004; Pileckis and Monsevičius 1997; Bowestead 2007.

**Parmulini Poey, 1854**.

*Clypastraea* **Haldeman, 1842** = *Sacium* LeConte, 1852.

*pusilla* **(Gyllenhal, 1810)**. Silfverberg 1992, 2004; Pileckis and Monsevičius 1997; Bowestead 2007.

*Arthrolips* **Wollaston, 1854**.

*obscura* **(R.F. Sahlberg, 1833)**. Bowestead 1999, 2007; Silfverberg 2004.

**Rypobiinae** **Paulian, 1950**.

**Rypobiini Paulian, 1950**.

*Rypobius* **LeConte, 1852**.

[*praetermissus* **Bowestead, 1999** = *ruficollis* auct. nec (Jacquelin du Val, 1854)]. Distributed from southern Sweden in the north through southern and central Europe (Bowestead 1999).

**Orthoperini Jacquelin du Val, 1857**.

*Orthoperus* **Stephens, 1829**.

*atomus* **(Gyllenhal, 1808)**. Tamutis 2003.

*brunnipes* **(Gyllenhal, 1808)**. Tamutis and Ferenca 2006; Ferenca et al. 2006, 2007.

[*corticalis* **Redtenbacher, 1849** = *improvisus* Bruce, 1946]. Known in Denmark, Latvia (Silfverberg 2004), Sweden, Belarus, Poland (Bowestead 1999).

[*nigrescens* **Stephens, 1829** = *mundus* Matthews, 1885 = *coriaceus* auct. nec Mulsant & Rey, 1861]. Known in Latvia (Telnov 2004), Denmark, Sweden (Lundberg and Gustafsson 1995), Poland (Audisio 2009).

*punctatus* **(Wankowicz, 1865)**. Silfverberg 1992, 2004; Pileckis and Monsevičius 1997; Šablevičius 2003a; Bowestead 2007.

*rogeri* **Kraatz, 1874** = *punctulatus* Reitter, 1876. Pileckis and Monsevičius 1997; Silfverberg 2004.

**LATRIDIIDAE Erichson, 1842**.

**Latridiinae** **Erichson, 1842**.

*Latridius* **Herbst, 1793**.

[*assimilis* **Mannerheim, 1844** = *pseudominutus* Strand, 1958].Known in Latvia (Telnov 2004), Denmark, Sweden (Lundberg and Gustafsson 1995).

[*brevicollis* **(Thomson, 1868)**].Known in Latvia (Telnov 2004), Denmark, Sweden (Lundberg and Gustafsson 1995), northwestern Belarus (Alexandrovitch et al. 1996), northern Poland (Burakowski et al. 1986c).

*consimilis* **Mannerheim, 1844**. Monsevičius and Pankevičius 2001; Johnson 2007.

[*gemmelatus* **Mannerheim, 1844** = *nidicola* (Palm, 1944)].Known in Latvia (Telnov 2004), Denmark, Sweden (Lundberg and Gustafsson 1995), Belarus (Alexandrovitch et al. 1996).

*hirtus* **Gyllenhal, 1827**. Pileckis 1976a; Silfverberg 1992, 2004; Pileckis and Monsevičius 1997; Johnson 2007; Rücker 2009.

*minutus* **(Linnaeus, 1767)**. Pileckis 1963b, 1976a; Silfverberg 1992, 2004; Pileckis and Monsevičius 1997; Johnson 2007; Rücker 2009.

*porcatus* **Herbs, 1793** =*anthracinus* Mannerheim, 1844. Monsevičius and Pankevičius 2001; Johnson 2007.

*Enicmus* **Thomson, 1859**.

[*atriceps* **Hansen, 1962**].Known in Denmark, Sweden (Silfverberg 2004).

*brevicornis* **(Mannerheim, 1844)**.Rücker 2009.

*fungicola* **Thomson, 1868**.Monsevičius and Pankevičius 2001; Rücker 2009.

*histrio* **Joy & Tomlin, 1910**. Monsevičius and Pankevičius 2001; Johnson 2007.

[*planipennis* **Strand, 1940**].Known in Latvia (Telnov 2004), Sweden (Lundberg and Gustafsson 1995), northwestern Belarus (Alexandrovitch et al. 1996).

*rugosus* **(Herbst, 1793)**. Jakaitis 1985; Silfverberg 1992, 2004; Pileckis and Monsevičius 1997; Johnson 2007; Alekseev 2008a; Rücker 2009; Ostrauskas and Ferenca 2010.

*testaceus* **(Stephens, 1830)**. Tamutis 2003.

*transversus* **(Olivier, 1790)**. Pileckis 1976a; Silfverberg 1992, 2004; Pileckis and Monsevičius 1997; Johnson 2007; Rücker 2009; Ostrauskas and Ferenca 2010.

*Dienerella* **Reitter, 1911** = *Cartodere* Thomson, 1863, nec Thomson, 1859.

[*argus* **(Reitter, 1884)**]. Known in Latvia (Telnov 2004).

[*clathrata* **(Mannerheim, 1844)** = *separanda* auct. nec (Reitter, 1887)].Known in Denmark, Sweden (Lundberg and Gustafsson 1995).

[*costulata* **(Reitter, 1877)**]. Known in Latvia (Telnov 2004), Denmark (Silfverberg 2004).

*elongata* **(Curtis, 1830)**. Ferenca et al. 2002; Johnson 2007.

*filiformis* **(Gyllenhal, 1827)**. Monsevičius and Pankevičius 2001.

*filum* **(Aubé, 1850)**. Monsevičius and Pankevičius 2001; Rücker 2009.

*ruficollis* **(Marsham, 1802)**. Pileckis 1968a, 1976a; Silfverberg 1992, 2004; Pileckis and Monsevičius 1997; Johnson 2007; Rücker 2009.

*Adistemia* **Fall, 1899**.

[*watsoni* **(Wollaston, 1871)**].Known in Latvia, Denmark, Sweden (Lundberg and Gustafsson 1995, Rücker 2009).

*Stephostethus* **LeConte, 1878** = *Lathridius* auct. nec Herbst, 1793.

*alternans* **(Mannerheim, 1844)**. Monsevičius and Pankevičius 2001; Johnson 2007.

*angusticollis* **(Gyllenhal, 1827)** = *kokujewi* (Semenow, 1898). Jakaitis 1985; Silfverberg 1992, 2004; Pileckis and Monsevičius 1997; Šablevičius 2000b; Johnson 2007; Alekseev 2008a; Rücker 2009.

*lardarius* **(DeGeer, 1775)**. Tamutis 1999; Monsevičius and Pankevičius 2001; Silfverberg 2004; Johnson 2007.

*pandellei* **(Brisout, 1863)**. Bercio and Folwaczny 1979; Silfverberg 1992, 2004; Johnson 2007; Rücker 2009.

*rugicollis* **(Olivier, 1790)**. Monsevičius and Pankevičius 2001; Johnson 2007; Rücker 2009.

*Thes* **Semenov-Tian-Shansky, 1910**.

*bergrothi* **(Reitter, 1880)**. Roubal 1910; Pileckis 1960, 1976a; Silfverberg 1992, 2004; Pileckis and Monsevičius 1997; Johnson 2007; Rücker 2009.

*Cartodere* **Thomson, 1859**.

[*bifasciata* **(Reitter, 1877)**].Known in Denmark, Sweden (Lundberg and Gustafsson 1995, Rücker 2009).

*constricta* **(Gyllenhal, 1827)**. Silfverberg 1992, 2004; Pileckis and Monsevičius 1997; Johnson 2007; Rücker 2009.

*nodifer* **(Westwood, 1839)**. Monsevičius and Pankevičius 2001; Johnson 2007; Rücker 2009.

**Corticariinae Curtis, 1829**.

*Corticaria* **Marsham, 1802**.

[*crenicollis* **Mannerheim, 1844**].Known in northwestern Belarus (Alexandrovitch et al. 1996), Sweden (Lundberg and Gustafsson 1995).

*crenulata* **(Gyllenhal, 1827)**. Tamutis 2003; Johnson 2007.

*elongata* **(Gyllenhal, 1827)**. Pileckis 1976a; Silfverberg 1992, 2004; Pileckis and Monsevičius 1997; Johnson 2007; Rücker 2009.

[*fagi* **Wollaston, 1854**].Known in Denmark, southern Sweden (Lundberg and Gustafsson 1995).

*ferruginea* **(Marsham, 1802)**. Bercio and Folwaczny 1979; Silfverberg 1992, 2004; Johnson 2007; Rücker 2009.

*foveola* **(Beck, 1817)**. Tamutis and Zolubas 2001; Silfverberg 2004; Johnson 2007.

*fulva* **(Comolli, 1837)**. Pileckis 1976a; Silfverberg 1992, 2004; Pileckis and Monsevičius 1997; Johnson 2007; Rücker 2009.

*impressa* **(Olivier, 1790)**. Bercio and Folwaczny 1979; Silfverberg 1992, 2004; Rücker 2009; Johnson 2007.

[*inconspicua* **Wollaston, 1860**].Known in Denmark, southern Sweden (Lundberg and Gustafsson 1995), Poland (Burakowski et al. 1986c).

[*lapponica* **(Zetterstedt, 1838)**].Known in northwestern Belarus (Alexandrovitch et al. 1996), Denmark, Sweden (Lundberg and Gustafsson 1995).

[*lateritia* **Mannerheim, 1844**].Known in Latvia (Telnov 2004), Sweden (Lundberg and Gustafsson 1995).

*linearis* **(Paykull, 1798)** = *rubripes* Mannerheim, 1844. Ivinskis et al. 2009; Rücker 2009.

*longicollis* **(Zetterstedt, 1838)**. Pileckis 1976a; Silfverberg 1992, 2004; Pileckis and Monsevičius 1997; Johnson 2007; Rücker 2009.

*longicornis* **(Herbst, 1783)** = *abietorum* Motschulsky, 1867. Tamutis 2003, Rücker 2009.

[*orbicollis* **Mannerheim, 1853** = *munsteri* Strand, 1937].Known in northwestern Belarus (Alexandrovitch et al. 1996), Sweden (Lundberg and Gustafsson 1995), Poland (Rücker 2009).

[*pineti* **Lohse, 1960**].Known in southern Sweden (Lundberg and Gustafsson 1995), Denmark (Silfverberg 2004).

[*polypori* **J.R. Sahlberg, 1900** = *eppelsheimi* Reitter, 1886, nec Reitter, 1875]. Known in Latvia (Telnov 2004), Sweden (Lundberg and Gustafsson 1995), northwestern Belarus (Alexandrovitch et al. 1996), Poland (Burakowski et al. 1986c).

*pubescens* **(Gyllenhal, 1827)**. Roubal 1910; Pileckis 1960, 1963b, 1976a; Silfverberg 1992, 2004; Pileckis and Monsevičius 1997; Rücker 2009; Johnson 2007.

*saginata* **Mannerheim, 1844**. Johnson 2007.

*serrata* **(Paykull, 1798)**. Heyden 1903; Pileckis 1960, 1976a; Silfverberg 1992, 2004; Pileckis and Monsevičius 1997; Johnson 2007; Rücker 2009.

*umbilicata* **(Beck, 1817)**. Monsevičius and Pankevičius 2001; Ferenca 2004; Ferenca et al. 2006, 2007; Johnson 2007; Rücker 2009.

*Cortinicara* **Johnson, 1975**.

*gibbosa* **(Herbst, 1793)**. Heyden 1903; Pileckis 1960, 1976a; Silfverberg 1992, 2004; Gaidienė 1993; Pileckis and Monsevičius 1997; Semaškienė et al. 1999; Tamutis and Zolubas 2001; Johnson 2007; Rücker 2009; Ostrauskas and Ferenca 2010.

*Corticarina* **Reitter, 1880**.

*lambiana* **(Sharp, 1910)**. Monsevičius and Pankevičius 2001; Johnson 2007.

[*latipennis* **(J.R. Sahlberg, 1871)**]. Known in Latvia (Telnov 2004), Estonia, Sweden (Lundberg and Gustafsson 1995), norther Poland (Burakowski et al. 1986c).

*minuta* **Fabricius, 1792** = *fuscula* (Gyllenhal, 1827). Heyden 1903; Pileckis 1960, 1976a; Silfverberg 1992, 2004; Gaidienė 1993; Pileckis and Monsevičius 1997; Johnson 2007; Alekseev 2008a; Rücker 2009.

[*obfuscata* **Strand, 1937**].Known in Latvia (Telnov 2004), Sweden (Lundberg and Gustafsson 1995), northwestern Belarus (Alexandrovitch et al. 1996).

*similata* **(Gyllenhal, 1827)**. Pileckis 1976a; Bercio and Folwaczny 1979; Silfverberg 1992, 2004; Pileckis and Monsevičius 1997; Johnson 2007; Rücker 2009.

*truncatella* **(Mannerheim, 1844)**. Bercio and Folwaczny 1979; Silfverberg 1992, 2004; Johnson 2007; Alekseev 2008a; Rücker 2009.

*Melanophthalma* **Motschulsky, 1866**.

*transversalis* **(Gyllenhal, 1827)** = *corticollis* (Mannerheim, 1844) = *hortensis* (Mannerheim, 1844). # 64. Pileckis 1976a, Silfverberg 1992, 2004; Pileckis and Monsevičius 1997; Johnson 2007; Rücker 2009.

*distinguenda* **(Comolli, 1837)**. Mazurowa and Mazur 1939; Pileckis 1960, 1976a; Silfverberg 1992, 2004; Pileckis and Monsevičius 1997; Johnson 2007; Rücker 2009.

[*suturalis* **(Mannerheim, 1844)**].Known in Latvia (Telnov et al. 2006), Sweden (Lundberg and Gustafsson 1995), Denmark (Silfverberg 2004), northwestern Belarus (Alexandrovitch et al. 1996).

*Migneauxia* **Jacquelin du Val, 1859**. 

[*lederi* **Reitter, 1875** = *orientalis* Reitter, 1877]. Known in Sweden, Denmark (Silfverberg 2004), Poland (Rücker 2009).

**TENEBRIONOIDEA latreille, 1802**.

**MYCETOPHAGIDAe Leach, 1815.**

**Mycetophaginae Leach, 1815**.

**Mycetophagini Leach, 1815**.

*Triphyllus* **Dejean, 1821**.

*bicolor* **(Fabricius, 1777)**. Ferenca et al. 2002; Ehnström et al. 2003.

*Litargus* **Erichson, 1846**.

*connexus* **(Geoffroy, 1785)**. Pileckis 1960, 1976a; Silfverberg 1992, 2004; Gaidienė 1993; Pileckis and Monsevičius 1997; Šablevičius 2000b; Tamutis and Zolubas 2001; Nikitsky 2008a, 2009; Šablevičius 2011.

*Mycetophagus* **Hellwig, 1792**.

*ater* **(Reitter, 1879)**. Ferenca et al. 2002, 2006, 2007; Silfverberg 2004; Ferenca 2004; Vaivilavičius 2008; Nikitsky 2008a; Šablevičius 2011.

*atomarius* **(Fabricius, 1787)**. Pileckis 1960, 1976a; Silfverberg 1992, 2004; Pileckis and Monsevičius 1997; Šablevičius 2000b, 2011; Nikitsky 2008a, 2009.

*decempunctatus* **Fabricius, 1801**. Pileckis and Monsevičius 1997; Silfverberg 2004; Nikitsky 2008a, 2009.

*fulvicollis* **Fabricius, 1792**. Pileckis 1976a; Silfverberg 1992, 2004; Gaidienė 1993; Pileckis and Monsevičius 1997; Vaivilavičius 2008; Nikitsky 2008a, 2009.

*multipunctatus* **Fabricius, 1792**. Šablevičius 2003a; Ferenca 2004; Vaivilavičius 2008; Nikitsky 2008a, 2009; Ivinskis et al. 2009.

*piceus* **(Fabricius, 1777)**. Pileckis 1976a; Silfverberg 1992, 2004; Gaidienė 1993; Pileckis and Monsevičius 1997; Ferenca 2004; Alekseev 2008a; Nikitsky 2008a, 2009.

*populi* **Fabricius, 1798**. Pileckis 1960, 1976a; Silfverberg 1992, 2004; Pileckis and Monsevičius 1997; Ferenca 2006b; Nikitsky 2008a, 2009; Ivinskis et al. 2009.

*quadriguttatus* **O.F. Müller, 1821**. Pileckis 1963b, 1976a; Silfverberg 1992, 2004; Pileckis and Monsevičius 1997; Šablevičius 2000b; Nikitsky 2008a, 2009.

*quadripustulatus* **(Linnaeus, 1761)**. Pileckis 1960, 1976a; Silfverberg 1992, 2004; Gaidienė 1993; Pileckis and Monsevičius 1997; Šablevičius 2000b, 2011; Ehnström et al. 2003; Ferenca 2006a, b; Vaivilavičius 2008; Nikitsky 2008a, 2009.

[*salicis* **Brisout, 1862**].Known in Latvia (Silfverberg 2004), Kaliningrad region (Nikitsky 2008a, 2009), Poland (Burakowski et al. 1986c).

**Typhaeini Thomson, 1863**.

*Typhaea* **Stephens, 1829**.

[*decipiens* **Lohse, 1989**].Known in Denmark, southern Sweden (Lundberg and Gustafsson 1995).

*stercorea* **(Linnaeus, 1758)**. Jakaitis 1985; Silfverberg 1992, 2004; Pileckis and Monsevičius 1997; Nikitsky 2008a, 2009; Ostrauskas and Ferenca 2010.

**CIIDAE Leach, 1819**.

**Ciinae Leach, 1819**.

**Ciini Leach, 1819**.

*Cis* **Latreille, 1796**.

*alter* **Silfverberg, 1991** = *nitidus* auct. nec (Fabricius, 1792). Pileckis 1976a; Silfverberg 1992, 2004; Gaidienė 1993; Pileckis and Monsevičius 1997; Šablevičius 2000b; Alekseev 2008a; Jelínek and Audisio 2009; Ivinskis et al. 2009.

*bidentatus* **(Olivier, 1790)**. Pileckis 1976a; Silfverberg 1992, 2004; Pileckis and Monsevičius 1997; Jelínek 2008; Jelínek and Audisio 2009.

*boleti* **(Scopoli, 1763)**. Miländer et al. 1984; Silfverberg 1992, 2004; Gaidienė 1993; Šablevičius and Ferenca 1995; Pileckis and Monsevičius 1997; Šablevičius 2000b; Jelínek 2008; Jelínek and Audisio 2009.

*castaneus* **Mellié, 1848** = *fusciclavis* Nyholm, 1954.Jelínek 2008.

*comptus* **Gyllenhal, 1827**. Gaidienė 1993; Ferenca et al. 2002; Silfverberg 2004; Alekseev 2008a.

[*dentatus* **Mellié, 1848**]. Known in Latvia (Telnov 2004), Sweden (Lundberg and Gustafsson 1995), Poland (Burakowski et al. 1987), western Belarus (Alexandrovitch et al. 1996), recently found in Estonia (Süda 2009).

*fagi* **Waltl, 1839**. Gaidienė 1993; Tamutis 2003; Silfverberg 2004; Alekseev 2008a.

*fissicornis* **Mellié, 1848**. Łomnicki 1913; Horrion 1951; Pileckis 1960, 1968b; 1976a; Silfverberg 1992, 2004; Pileckis and Monsevičius 1997; Jelínek 2008.

*glabratus* **Mellié, 1848**. Tamutis and Ferenca 2006; Ferenca et al. 2006, 2007.

[*hanseni* **Strand, 1965**].Known in Latvia (Telnov 2004), Denmark, southern Sweden (Lundberg and Gustafsson 1995), Poland (Jelínek and Audisio 2009).

*hispidus* **(Paykull, 1798)**. Pileckis 1976a; Silfverberg 1992, 2004; Gaidienė 1993; Pileckis and Monsevičius 1997; Šablevičius 2000b; Tamutis and Zolubas 2001; Gedminas 2005; Gedminas et al. 2007; Alekseev 2008a; Jelínek and Audisio 2009; Ivinskis et al. 2009.

*jacquemartii* **Mellié, 1848**. Roubal 1910; Pileckis 1960, 1976a; Silfverberg 1992, 2004; Gaidienė 1993; Pileckis and Monsevičius 1997; Šablevičius 2000b; Alekseev 2008a; Jelínek 2008; Jelínek and Audisio 2009.

[*laminatus* **Mellié, 1849**].Known in northern Poland (Burakowski et al. 1987), Kaliningrad region (Jelínek and Audisio 2009).

[*lineatocribratus* **Mellié, 1848**]. Known in Latvia, Denmark, Sweden (Lundberg and Gustafsson 1995), Poland (Burakowski et al. 1987), western Belarus (Alexandrovitch et al. 1996), recently found in Estonia (Süda 2009).

*micans* **(Fabricius, 1792)**. Pileckis and Jakaitis 1989; Silfverberg 1992, 2004; Pileckis and Monsevičius 1997; Šablevičius 2000b; Jelínek 2008; Jelínek and Audisio 2009.

*punctulatus* **Gyllenhal, 1827**. Monsevičius and Pankevičius 2001; Jelínek 2008.

*quadridens* **Mellié, 1848**. Pileckis and Monsevičius 1997; Silfverberg 2004.

*rugulosus* **Mellié, 1848**. Jelínek 2008.

*setiger* **Mellié, 1848** = *petropolitanus* Jacobson, 1895. Pileckis and Monsevičius 1997; Silfverberg 2004; Alekseev 2008a; Ivinskis et al. 2009.

*submicans* **(Abeille de Perrin, 1874)** = *micans* sensu auct. nec (Fabricius, 1792). Jelínek 2008.

*Ennearthron* **Mellié, 1847**.

*cornutum* **(Gyllenhal, 1827)**. Gaidienė 1993; Pileckis and Monsevičius 1997; Silfverberg 2004; Vaivilavičius 2008; Alekseev 2008a; Jelínek 2008.

*laricinum* **(Mellié, 1848)**. Pileckis 1976a; Silfverberg 1992, 2004; Pileckis and Monsevičius 1997; Ostrauskas and Ferenca 2010.

[*palmi* **Lohse, 1966** = *filum* auct. nec Abeille de Perrin, 1874].Known in Sweden (Lundberg and Gustafsson 1995), Poland (Burakowski et al. 1987), western Belarus (Alexandrovitch et al. 1996).

*Orthocis* **Casey, 1898**.

*alni* **(Gyllenhal, 1813)**. Miländer et al. 1984; Silfverberg 1992, 2004; Pileckis and Monsevičius 1997; Tamutis and Zolubas 2001; Gedminas 2005; Gedminas et al. 2007; Jelínek 2008; Jelínek and Audisio 2009.

*festivus* **(Panzer, 1793)**. Šablevičius and Ferenca 1995 (*Cis gestivus* Gyll.); Pileckis and Monsevičius 1997; Silfverberg 2004.

*linearis* **(J.R. Sahlberg, 1900)** = *perrisi* auct. nec (Abeille de Perrin, 1874). Vaivilavičius 2008; Ferenca and Tamutis 2009.

[*lucasi* **(Abeille de Perrin, 1874)**]. Known in Latvia (Telnov et al. 2006), western Belarus (Alexandrovitch et al. 1996).

[*pygmaeus* **(Marsham, 1802)** = *rhododactylus* (Marsham, 1802)].Known in Latvia (Telnov 2004), Denmark, southern Sweden (Lundberg and Gustafsson 1995), Poland (Burakowski et al. 1987).

[*vestitus* **(Mellié, 1848)** = *pygmaeus* auct. nec (Marsham, 1802)]. Known in Latvia (Telnov 2004), Denmark, southern Sweden (Lundberg and Gustafsson 1995), western Belarus (Alexandrovitch et al. 1996).

*Hadreule* **Thomson, 1859**.

*elongatula* **(Gyllenhal, 1827)**. Tamutis and Zolubas 2001; Silfverberg 2004; as *Cis elongatulis* Gyll. in: Šablevičius and Ferenca 1995; Šablevičius 2000b.

*Sulcacis* **Dury, 1917**.

*affinis* **(Gyllenhal, 1827)**. Silfverberg 1992, 2004; Gaidienė 1993; Pileckis and Monsevičius 1997; Tamutis and Zolubas 2001; Gedminas 2005; Ferenca et al. 2006, 2007; Gedminas et al. 2007; Vaivilavičius 2008; Alekseev 2008a; Jelínek 2008; Jelínek and Audisio 2009; Ivinskis et al. 2009; as *Ennearthron affine* Gyll. in: Jakaitis 1973; Pileckis 1976a.

[*bidentulus* **(Rosenhauer, 1847)**]. Known in northern Belarus (Alexandrovitch et al. 1996), Poland (Burakowski et al. 1987).

[*fronticornis* **(Panzer, 1809)**]. Known in Latvia (Telnov 2004), Denmark, Sweden (Lundberg and Gustafsson 1995), Poland (Burakowski et al. 1987), western Belarus (Alexandrovitch et al. 1996), recently found in Estonia (Süda 2009).

*Wagaicis* **Lohse, 1964**.

*wagai* **(Wankowicz, 1869)**. Pileckis and Monsevičius 1997; Silfverberg 2004.

*Ropalodontus* **Mellié, 1847**.

*perforatus* **(Gyllenhal, 1813)**. Monsevičius and Pankevičius 2001.

[*strandi* **Lohse, 1969**]. Known in Sweden (Lundberg and Gustafsson 1995), recently found in Estonia (Süda 2009).

*Octotemnus* **Mellié, 1847**.

*glabriculus* **(Gyllenhal, 1827)**. Miländer et al. 1984; Silfverberg 1992, 2004; Pileckis and Monsevičius 1997; Vaivilavičius 2008; Jelínek 2008; Jelínek and Audisio 2009.

*mandibularis* **(Gyllenhal, 1813)**. Łomnicki 1913; Horion 1960; Pileckis 1968b; 1976a; Silfverberg 1992, 2004; Pileckis and Monsevičius 1997; Jelínek 2008; Jelínek and Audisio 2009.

**TETRATOMIDAE Billberg, 1820**.

**Tetratominae** **Billberg, 1820**.

*Tetratoma* **Fabricius, 1790**. (Melandryidae)

*ancora* **Fabricius, 1790**. Monsevičius and Pankevičius 2001; Nikitsky 2008b.

[*dermarestii* **Latreille, 1807**]. Known in Denmark, southern Sweden (Lundberg and Gustafsson 1995).

*fungorum* **Fabricius, 1790**. Silfverberg 1992, 2004; Pileckis and Monsevičius 1997; Nikitsky 2008b.

**Hallomeninae Gistel, 1848**. (Melandryidae)

*Hallomenus* **Panzer, 1794**.

*axillaris* **(Illiger, 1807)**. Monsevičius and Pankevičius 2001; Nikitsky and Pollock 2008; Nikitsky 2009; Ivinskis et al. 2009.

*binotatus* **(Quensel, 1790)**. Ferenca 1988; Silfverberg 1992, 2004; Pileckis and Monsevičius 1997; Šablevičius 2003a.

**Eustropinae Gistel, 1848**.

*Eustrophus* **Latreille, 1807**.

*dermestoides* **(Fabricius, 1793)**.Nikitsky and Pollock 2008; Nikitsky 2009.

**MELANDRYIDAE Leach, 1815**.

**Melandryinae Leach, 1815**.

**Orchesiini Mulsant, 1856**.

*Orchesia* **Panzer, 1794**.

*fasciata* **(Illiger, 1798)**. Silfverberg 1992, 2004; Gaidienė 1993; Pileckis and Monsevičius 1997; Šablevičius 2003a, 2011; Nikitsky and Pollock 2008; Nikitsky 2009

[*fusiformis* **Solsky, 1871**].Known in Latvia (Telnov 2004), northwestern Belarus (Alexandrovitch et al. 1996), Poland (Burakowski et al. 1987).

[*luteipalpis* **Mulsant & Guillebeau, 1857**].Known in Latvia (Telnov 2004), northwestern Belarus (Alexandrovitch et al. 1996), Poland (Burakowski et al. 1987).

*micans* **(Panzer, 1794)**. Roubal 1910; Pileckis 1960, 1976a; Silfverberg 1992, 2004; Gaidienė 1993; Pileckis and Monsevičius 1997; Šablevičius 2003a, 2011; Ferenca 2004; Ferenca et al. 2006, 2007; Nikitsky 2009; Ostrauskas and Ferenca 2010.

*minor* **Walker, 1837**. Pileckis 1976a; Silfverberg 1992, 2004; Pileckis and Monsevičius 1997; Ferenca et al. 2006, 2007; Nikitsky and Pollock 2008; Nikitsky 2009; Ostrauskas and Ferenca 2010.

[*undulata* **Kraatz, 1853**].Known in Latvia (Telnov 2004), Denmark, Sweden (Lundberg and Gustafsson 1995), Estonia (Silfverberg 2004), northwestern Belarus (Alexandrovitch et al. 1996), Poland (Burakowski et al. 1987).

**Dircaeini Kirby, 1837**.

*Anisoxya* **Mulsant, 1856**.

*fuscula* **(Illiger, 1798)**. Tamutis 2003.

*Abdera* **Stephens, 1832**.

*affinis* **(Paykull, 1799)**. Pileckis 1976a; Silfverberg 1992, 2004; Pileckis and Monsevičius 1997; Šablevičius 2003b, 2004; Nikitsky and Pollock 2008; Nikitsky 2009

[*bifasciata* **(Marsham, 1802)** = *biflaxuosa* (Curtis, 1829)].Known in Estonia, Denmark (Silfverberg 2004), Poland (Nikitsky 2009).

*flexuosa* **(Paykull, 1799)**.Nikitsky and Pollock 2008; Nikitsky 2009.

*Wanachia* **Schulze, 1912**.

*triguttata* **(Gyllenhal, 1810)**. Ferenca et al. 2002.

*Dircaea* **Fabricius, 1798**.

[*australis* **Fairmaire, 1856**].Known in southern Sweden (Lundberg and Gustafsson 1995), Belarus (Nikitsky 2009).

[*quadriguttata* **(Paykull, 1798)**].Known in Latvia (Telnov 2004), Estonia, southern Sweden (Lundberg and Gustafsson 1995), western Belarus (Alexandrovitch et al. 1996), northeastern Poland (Burakowski et al. 1987).

*Phloiotrya* **Stephens, 1832**.

*rufipes* **(Gyllenhal, 1810)**. Ferenca et al. 2002.

**Xylitini Thomson, 1864**.

*Xylita* **Paykull, 1798**.

*laevigata* **(Hellenius, 1786)**. Pileckis 1960, 1963b, 1976a; Silfverberg 1992, 2004; Gaidienė 1993; Pileckis and Monsevičius 1997; Šablevičius 2003a; Ferenca 2004, 2006b; Vaivilavičius 2008; Nikitsky and Pollock 2008; Nikitsky 2009.

*Dolotarsus* **Jacquelin du Val, 1863**.

*lividus* **(C.R. Sahlberg, 1833)**. Šablevičius 2004; Ferenca 2004.

**Seropalpini Latreille, 1829**.

*Serropalpus* **Hellenius, 1786**.

*barbatus* **(Schaller, 1783)**. Pileckis 1960, 1963b, 1976a; Silfverberg 1992, 2004; Monsevičius 1997; Pileckis and Monsevičius 1997; Ferenca 2006b; Nikitsky and Pollock 2008; Ivinskis et al. 2009; Šablevičius 2011.

**Hypulini Gistel, 1848**.

*Hypulus* **Paykull, 1798**.

*bifasciatus* **(Fabricius, 1792)**. Silfverberg 1992, 2004; Pileckis and Monsevičius 1997; Ehnström et al. 2003; Nikitsky and Pollock 2008; as *H. fasciatus* F. in (Pileckis 1976a).

*quercinus* **(Quensel, 1790)**. Ehnström et al. 2003; Ferenca 2004; Ferenca et al. 2006, 2007; Vaivilavičius 2008.

**Zylorini Desbrochers des Loges, 1900**.

*Zilora* **Mulsant, 1856**.

[*elongata* **J. R. Sahlberg, 1881**].Known in Estonia (Silfverberg 2004), Belarus (Nikitsky 2009).

*ferruginea* **(Paykull, 1798)**.Nikitsky and Pollock 2008.

*obscura* **(Fabricius, 1794)** = *sericea* (Sturm, 1807). Gaidienė and Ferenca 1988; Gaidienė 1993; Pileckis and Monsevičius 1997; Ferenca et al. 2002; Silfverberg 2004; Nikitsky 2009; Ivinskis et al. 2009.

**Melandryini Leach, 1815**.

*Melandrya* **Fabricius, 1801**.

[*barbata* **(Fabricius, 1792)**]. Known in Denmark, southern Sweden (Lundberg and Gustafsson 1995), Poland (Burakowski et al. 1987).

[*caraboides* **(Linnaeus, 1760)**].Known in Latvia (Telnov 2004), Denmark, southern Sweden (Lundberg and Gustafsson 1995), northeastern Poland (Burakowski et al. 1987). 

*dubia* **(Schaller, 1783)**. Pileckis 1968a, 1976a; Silfverberg 1992, 2004; Gaidienė 1993; Pileckis and Monsevičius 1997; Šablevičius 2000b, 2011; Ehnström et al. 2003; Ivinskis et al. 2004b; Butvila et al. 2007; Vaivilavičius 2008; Nikitsky and Pollock 2008; Nikitsky 2009; Ivinskis et al. 2009.

*Phryganophilus* **R.F. Sahlberg, 1833**.

*auritus* **Motschulsky, 1845**. Tamutis et al. 2008; Inokaitis 2009.

*ruficollis* **(Fabricius, 1798)**. Inokaitis 2009.

**OpsyinaeMulsant, 1856 (1839)**

*Conopalpus* **Gyllenhal, 1810**.

[*testaceus* **(Olivier, 1790)**].Known in Latvia (Telnov 2004), Denmark, southern Sweden (Lundberg and Gustafsson 1995), northern Poland (Burakowski et al. 1987).

*Osphya* **Illiger, 1807**.

[*bipunctata* **(Fabricius, 1775)**].Known in Denmark, southern Sweden (Lundberg and Gustafsson 1995), northern Poland (Burakowski et al. 1987), Belarus (Nikitsky 2009).

**MORDELLIDAE Latreille, 1802**.

**Mordellinae** **Latreille, 1802**.

**Mordellini Latreille, 1802**.

*Tomoxia* **Costa, 1854**.

*bucephala* **Costa, 1854** = *biguttata* (Gyllenhal, 1827) nec (P. Rossi, 1794). Mazurowa and Mazur 1939; Pileckis 1960, 1976a; Silfverberg 1992, 2004; Gaidienė 1993; Pileckis and Monsevičius 1997; Šablevičius 2000b; Gliaudys 2001.

*Variimorda* **Méquignon, 1946**.

[*briantea* **(Comolli, 1837)**].Known in Latvia (Telnov 2004), Estonia (Silfverberg 2004), Poland (Burakowski et al. 1987).

*villosa* **(Schrank, 1781)** = *fasciata* (Fabricius, 1775) nec (Forster, 1771). Mazurowa and Mazur 1939; Pileckis 1960, 1976a; Lešinskas and Pileckis 1967; Zajančkauskas and Pileckis 1968; Silfverberg 1992, 2004; Gaidienė 1993; Monsevičius 1997; Pileckis and Monsevičius 1997; Šablevičius 2000b; Gliaudys 2001; Tamutis and Zolubas 2001; Ferenca 2006b; Ostrauskas and Ferenca 2010.

*Mordella* **Linnaeus, 1758**.

*aculeata* **Linnaeus, 1758**. Eichwald 1830; Monsevičius 1988a, 1997; Silfverberg 1992, 2004; Gaidienė 1993; Pileckis and Monsevičius 1997; Gedminas et al. 2007; Ivinskis et al. 2009.

[*brachyura* **Mulsant, 1856**].Known in southern Sweden (Lundberg and Gustafsson 1995), Poland (Burakowski et al. 1987), western Belarus (Alexandrovitch et al. 1996).

*holomelaena* **Apfelbeck, 1914**. Gaidienė 1993; Pileckis and Monsevičius 1997; Šablevičius 2000b; Silfverberg 2004; Ivinskis et al. 2009.

[*huetheri* **Ermisch, 1956**].Known in southern Sweden (Lundberg and Gustafsson 1995), northeastern Poland (Burakowski et al. 1987).

[*leucaspis* **Küster, 1849**].Known in Estonia (Silfverberg 2004), Poland (Burakowski et al. 1987).

*Hoshihananomia* **Kono, 1935**.

*perlata* **(Sulzer, 1776)**. Pileckis 1976a; Silfverberg 1992, 2004; Gaidienė 1993; Pileckis and Monsevičius 1997.

*Curtimorda* **Méquignon, 1946**.

[*bisignata* **(Redtenbacher, 1849)**]. Known in Latvia (Telnov et al. 2006), Poland (Burakowski et al. 1987).

*maculosa* **(Naezen, 1794)**. Pileckis and Monsevičius 1997; Silfverberg 2004.

**Mordellistenini Ermisch, 1941**.

*Mordellistenula* **Stshegoleva-Barovskaja, 1930**.

[*perrisi* **(Mulsant, 1856)** = *rectangula* (Thomson, 1868)]. Known in Denmark, southern Sweden (Lundberg and Gustafsson 1995), northern Poland (Burakowski et al. 1987).

*Mordellistena* **Costa, 1854**.

*brevicauda* **(Boheman, 1849)** = *episternalis* (Mulsant, 1856). Pileckis 1960, 1976a; Gaidienė 1993; Silfverberg 2004; Ferenca 2006b; as doubtful in (Pileckis and Monsevičius 1997).

[*carinthiaca* **Ermisch, 1966**].Known in southern Sweden (Lundberg and Gustafsson 1995), Germany (Horák 2009).

[*connata* **Ermisch, 1969**].Known in Sweden (Lundberg and Gustafsson 1995), Poland (Burakowski et al. 1987), Belarus (Alexandrovitch et al. 1996).

[*dieckmanni* **Ermisch, 1963**]. Known in southern Sweden (Lundberg and Gustafsson 1995), Poland (Burakowski et al. 1987).

[*falsoparvula* **Ermisch, 1956**].Known in Estonia (Silfverberg 2004), Poland (Burakowski et al. 1987).

*humeralis* **(Linnaeus, 1758)**. Pileckis 1976a; Silfverberg 1992, 2004; Gaidienė 1993; Monsevičius 1997; Pileckis and Monsevičius 1997; Vaivilavičius 2008.

[*koelleri* **Ermisch, 1956**]. Known in southern Sweden (Lundberg and Gustafsson 1995), Denmark (Horák 2009), Poland (Burakowski et al. 1987).

[*neuwaldeggiana* **(Panzer, 1796)**].Known in Denmark, southern Sweden (Lundberg and Gustafsson 1995), northern Poland (Burakowski et al. 1987), Belarus (Alexandrovitch et al. 1996), recently found in Estonia (Süda 2009).

*parvula* **(Gyllenhal, 1827)**. Roubal 1910; Pileckis 1960, 1976a; Silfverberg 1992, 2004; Pileckis and Monsevičius 1997; Alekseev 2008a.

[*pseudobrevicauda* **Ermisch, 1963**]. Known in Estonia (Silfverberg 2004), Poland, Germany (Horák 2008, 2009).

*pseudonana* **Ermisch, 1956** = *nana* auct. nec (Motschulsky, 1860). Roubal 1910; Pileckis 1960, 1976a; Silfverberg 1992, 2004; Pileckis and Monsevičius 1997.

*pseudopumila* **Ermisch, 1963**. Monsevičius and Pankevičius 2001.

*pumila* **(Gyllenhal, 1810)**. Silfverberg 1992, 2004; Gaidienė 1993; Pileckis and Monsevičius 1997; Ivinskis et al. 2009.

[*purpureonigricans* **Ermisch, 1963**]. Known in Estonia (Silfverberg 2004), Denmark, southern Sweden (Lundberg and Gustafsson 1995), northern Poland (Burakowski et al. 1987).

*pygmaeola* **Ermisch, 1956**. Ferenca and Tamutis 2009.

[*rhenana* **Ermisch, 1956**].Known in Estonia (Silfverberg 2004), Germany (Horák 2009).

[*secreta* **Horák, 1983**]. Known in Sweden (Silfverberg 2004), Poland (Horák 2009).

*stenidea* **(Mulsant, 1856)**.Pileckis 1968b, 1976a; Pileckis and Monsevičius 1997; Silfverberg 2004.

[*thurepalmi* **Ermisch, 1965** = *palmi* Ermisch, 1965, nec Liljeblad 1945].Known in Sweden (Lundberg and Gustafsson 1995), Poland (Burakowski et al. 1987).

*variegata* **(Fabricius, 1798)**. Pileckis 1968b, 1976a; Silfverberg 1992, 2004; Gaidienė 1993; Pileckis and Monsevičius 1997.

*Mordellochroa* **Emery, 1876**.

*abdominalis* **(Fabricius, 1775)**. Gaidienė 1993; Pileckis and Monsevičius 1997; Silfverberg 2004; Horák 2008, 2009.

[*tournieri* **(Emery, 1876)**]. Known in Latvia, southern Sweden (Lundberg and Gustafsson 1995), Poland (Burakowski et al. 1987).

**RHIPIPHORIDAE Laporte, 1840**.

**Pelecotominae** **Seidlitz, 1875**.

*Pelecotoma* **Fischer v. Waldheim, 1809**.

*fennica* **(Paykull, 1799)**. Pileckis 1976a; Silfverberg 1992, 2004; Pileckis and Monsevičius 1997; Ehnström et al. 2003.

**Ripidiinae** **Gerstaecker, 1855**.

**Ripidiini Gerstaecker, 1855**.

*Ripidius* **Thunberg, 1806**.

[*quadriceps* **Abeille de Perrin, 1872**].Recently found in Latvia (Telnov et al. 2010) and Estonia (Süda 2009), known in Sweden (Lundberg and Gustafsson 1995), Poland (Pawłowski et al. 2002).

**Rhipiphorinae** **Gemminger, 1870 (1855)**.

**Macrosiagonini Heyden, 1908**.

*Metoecus* **Dejean, 1834**.

*paradoxus* **(Linnaeus, 1761)**. Pileckis and Monsevičius 1997; Ferenca et al. 2002; Ferenca 2003, 2004; Inokaitis 2004; Silfverberg 2004; Vaivilavičius 2008.

**ZOPHERIDAE Solier, 1834**. (Colydidae)

**Colydiinae** **Billberg, 1820**.

**Colydiini Billberg, 1820**.

*Colydium* **Fabricius, 1792**.

*elongatum* **(Fabricius, 1787)**. Pileckis and Monsevičius 1997; Silfverberg 2004; Inokaitis 2004; Ślipiński and Schuh 2008.

*filiforme* **Fabricius, 1792**. Inokaitis 2004; Ferenca 2004; Ivinskis et al. 2009.

*Aulonium* **Erichson, 1845**.

*trisulcum* **(Geoffroy, 1785)**. Pileckis 1968b, 1976a; Silfverberg 1992, 2004; Pileckis and Monsevičius 1997; Ślipinski 2009.

**Synchitini Erichson, 1845**.

*Synchita* **Hellwig, 1792**.

*humeralis* **(Fabricius, 1793)**. Silfverberg 1992, 2004; Gaidienė 1993; Pileckis and Monsevičius 1997; Šablevičius 2001, 2003a; Ferenca 2004; Ferenca et al. 2006, 2007; Butvila et al. 2007; Ślipinski 2009; Ivinskis et al. 2009; Ostrauskas and Ferenca 2010.

[*mediolanensis* **(Villa & Villa, 1833)**]. Known in northern Poland (Burakowski et al. 1987).

[*separanda* **(Reitter, 1881)**]. Known in Sweden (Lundberg and Gustafsson 1995), Poland (Burakowski et al. 1987).

*variegata* **Hellwig, 1792**. Ślipinski 2009.

*Bitoma* **Herbst, 1793**.

*crenata* **(Fabricius, 1775)**. Eichwald 1830; Mazurowa and Mazur 1939; Pileckis 1960, 1963b, 1976a; Lešinskas and Pileckis 1967; Jakaitis and Valenta 1976; Silfverberg 1992, 2004; Gaidienė 1993; Pileckis and Monsevičius 1997; Šablevičius 2000b; Tamutis and Zolubas 2001; Šablevičius 2003b; Gedminas 2005; Gedminas et al. 2007; Ślipinski 2009.

*Lasconotus* **Erichson, 1845** = *Lado* Wankowicz, 1867.

*jelskii* **(Wankowicz, 1867)**. Pileckis 1976a; Silfverberg 1992, 2004; Pileckis and Monsevičius 1997; Ślipiński and Schuh 2008.

*Xylolaemus* **Dejean, 1835**.

[*fasciculosus* **(Gyllenhal, 1827)**]. Known in southern Sweden (Lundberg and Gustafsson 1995).

**Orthocerini Blanchard, 1845**.

*Orthocerus* **Latreille, 1796**.

*clavicornis* **(Linnaeus, 1758)**. Pileckis 1960, 1976a; Silfverberg 1992, 2004; Gaidienė 1993; Pileckis and Monsevičius 1997; Ferenca 2004, 2006b; Ferenca et al. 2006, 2007; Ślipinski 2009.

[*crassicornis* **(Erichson, 1845)**]. Known in Estonia (Silfverberg 2004), Poland (Ślipinski 2009).

**TENEBRIONIDAE Latreille, 1802**.

**Lagriinae** **Latreille, 1825**. (Lagriidae)

**Lagriini Latreille, 1825**.

*Lagria* **Fabricius, 1775**.

[*atripes* **Mulsant & Guillebeau, 1855**]. Known in northern Poland (Burakowski et al. 1987).

*hirta* **(Linnaeus, 1758)**. Heyden 1903; Roubal 1910; Mazurowa and Mazur 1939; Pileckis 1960, 1976a; Lešinskas and Pileckis 1967; Zajančkauskas and Pileckis 1968; Silfverberg 1992, 2004; Gaidienė 1993; Monsevičius 1997; Pileckis and Monsevičius 1997; Šablevičius 2000b; Gliaudys 2001; Žiogas and Zolubas 2005; Ferenca 2006b; Ivinskis et al. 2008; Löbl et al. 2008; Fattorini 2009; Ostrauskas and Ferenca 2010.

**Tenebrioninae** **Latreille, 1802**.

**Blaptini Leach, 1815**.

*Blaps* **Fabricius, 1775**.

[*lethifera* **Marsham, 1802**]. Known in Latvia (Telnov 2004), Estonia, Denmark, southern Sweden (Lundberg and Gustafsson 1995), northern Poland (Burakowski et al. 1987).

*mortisaga* **(Linnaeus, 1758)**. Eichwald 1830; Heyden 1903; Pileckis 1960, 1970c, 1976a; Lešinskas and Pileckis 1967; Zubrys 1967; Silfverberg 1992, 2004; Gaidienė 1993; Pileckis and Monsevičius 1997; Gliaudys 2001; Ferenca 2006b; Löbl et al. 2008; Fattorini 2009; Šablevičius 2011.

[*mucronata* **Latreille, 1804** = *obtusa* Gyllenhal, 1813].Known in Denmark, southern Sweden (Lundberg and Gustafsson 1995), northeastern Poland (Burakowski et al. 1987).

[*sinuaticollis suecica* **Ferrer & Picka, 1990**]. Known in southern Sweden (Lundberg and Gustafsson 1995).

**Platyscelidini Lacordaire, 1859**.

*Platyscelis* **Latreille, 1825**.

*polita* **(Sturm, 1807)**. Pileckis 1968b, 1970a, b, c, 1976a, b, 1979; Pileckis and Monsevičius 1997; Silfverberg 2004; Löbl et al. 2008.

**Pedinini Eschscholtz, 1829**.

*Phylan* **Dejean, 1821**.

*gibbus* **(Fabricius, 1775)**. Silfverberg 1992, 2004; Pileckis and Monsevičius 1997; Šablevičius 2000a, 2003a, 2011; Ferenca 2004; Löbl et al. 2008; Fattorini 2009.

*Pedinus* **Latreille, 1796**.

*femoralis **(Linnaeus, 1767)**. # 65. Pileckis and Monsevičius 1997; Silfverberg 2004.

**Opatrini Brullé, 1832**.

*Opatrum* **Fabricius, 1775**.

*riparium* **Scriba, 1865**. Pileckis 1960, 1970c, 1976a; Silfverberg 1992, 2004; Gaidienė 1993; Pileckis and Monsevičius 1997; Ferenca 2006b; Löbl et al. 2008; Fattorini 2009.

*sabulosum* **(Linnaeus, 1761)**. Mazurowa and Mazur 1939; Pileckis 1960, 1976a; Lešinskas and Pileckis 1967; Pileckis et al. 1968; Zajančkauskas and Pileckis 1968; Silfverberg 1992, 2004; Gaidienė 1993; Monsevičius 1997; Pileckis and Monsevičius 1997; Gliaudys 2001; Ferenca 2006b; Löbl et al. 2008; Fattorini 2009.

**Melanimonini Seidlitz, 1894 (1854)**.

*Melanimon* **Steven, 1829**.

*tibialis* **(Fabricius, 1781)**. Pileckis 1960, 1970c, 1976a; Silfverberg 1992, 2004; Gaidienė 1993; Pileckis and Monsevičius 1997; Žiogas and Zolubas 2005; Ferenca 2006b; Löbl et al. 2008; Fattorini 2009; Šablevičius 2011.

**Ulomini Blanchard, 1845**.

*Uloma* **Dejean, 1821**.

RDB*culinaris* **(Linnaeus, 1758)**. Pileckis 1988; Silfverberg 1992, 2004; Gaidienė 1993; Pileckis and Monsevičius 1997; Ivinskis et al. 1997a; Ferenca et al. 2002; Ehnström et al. 2003; Ferenca 2004; Tamutis 2005b; Rašomavičius 2007; Löbl et al. 2008; Fattorini 2009; Alekseev 2010b; Šablevičius 2011.

*rufa* **(Piller & Mitterpacher, 1783)** = *perroudii* (Mulsant & Guillebeau, 1855). Pileckis 1960, 1976a; Jakaitis and Valenta 1976; Silfverberg 1992, 2004; Gaidienė 1993; Monsevičius 1997; Pileckis and Monsevičius 1997; Gliaudys 2001; Ferenca 2006b; Alekseev 2008a; Löbl et al. 2008; Fattorini 2009; Šablevičius 2011.

**Triboliini Gistel, 1848**.

*Tribolium* **MacLeay, 1825**.

*castaneum* **(Herbst, 1797)**. Pileckis 1970a, c, 1976a; Silfverberg 1992, 2004; Pileckis and Monsevičius 1997; Löbl et al. 2008; Fattorini 2009; as *Tribolium ferrugineum* F. in (Pileckis 1962, 1963b; Zubrys 1967).

*confusum* **Jacquelin du Val, 1863**. Ogyjewicz 1938; Pileckis 1960, 1970a, c, 1976a, 1998; Lešinskas and Pileckis 1967; Zubrys 1967; Pileckis and Vengeliauskaitė 1977, 1996; Silfverberg 1992, 2004; Gaidienė 1993; Pileckis et al. 1994b; Pileckis and Monsevičius 1997; Gliaudys 2001; Löbl et al. 2008; Fattorini 2009.

*destructor* **Uyttenboogart, 1934**. Pileckis 1968b, 1970a, c, 1976a, 1998; Pileckis and Vengeliauskaitė 1977, 1996; Pileckis et al. 1994b; Silfverberg 1992, 2004; Pileckis and Monsevičius 1997; Löbl et al. 2008; Fattorini 2009.

*madens* **(Charpentier, 1825)**. Tamutis and Zolubas 2001; Silfverberg 2004.

*Latheticus* **Waterhouse, 1880**.

[*oryzae* **(Waterhouse, 1880)**]. Imported in Latvia, Denmark, Sweden (Lundberg and Gustafsson 1995), known in Poland (Fattorini 2009).

**Tenebrionini Latreille, 1802**.

*Tenebrio* **Linnaeus, 1758**.

*molitor* **Linnaeus, 1758**. Heyden 1903; Ivanauskas and Vailionis 1922; Mastauskis 1936; Ogyjewicz 1934; Mazurowa and Mazur 1939; Pileckis 1960, 1970a, c, 1976a, 1998; Lešinskas and Pileckis 1967; Zubrys 1967; Pileckis and Vengeliauskaitė 1977, 1996; Silfverberg 1992, 2004; Gaidienė 1993; Pileckis et al. 1994b; Monsevičius 1997; Pileckis and Monsevičius 1997; Šablevičius 2000b, 2011; Gliaudys 2001; Ferenca 2006b; Vaivilavičius 2008; Löbl et al. 2008; Fattorini 2009.

*obscurus* **Fabricius, 1792**. Monsevičius 1988b; Silfverberg 1992, 2004; Pileckis and Monsevičius 1997; Ferenca et al. 2006, 2007; Vaivilavičius 2008; Löbl et al. 2008; Fattorini 2009.

[*opacus* **Duftschmid, 1812**].Known in Denmark, southern Sweden (Lundberg and Gustafsson 1995), northern Poland (Burakowski et al. 1987).

*Neatus* **LeConte, 1862**.

*picipes* **(Herbst, 1797)**. Pileckis and Monsevičius 1997; Silfverberg 2004.

*Bius* **Dejean, 1834**.

[*thoracicus* **(Fabricius, 1792)**]. Known in western Belarus (Alexandrovich et al. 1996), Poland (Burakowski et al. 1987), Estonia, Sweden (Lundberg and Gustafsson 1995).

*Upis* **Fabricius, 1792**.

*ceramboides* **(Linnaeus, 1758)**. Łomnicki 1913; Pileckis 1968b, 1970a, 1976a, b, 1979; Silfverberg 1992, 2004; Pileckis and Monsevičius 1997; Ehnström et al. 2003; Löbl et al. 2008; Fattorini 2009.

*Menephilus* **Mulsant, 1854**.

[*cylindricus* **(Herbst, 1784)**]. Known in northern Poland (Burakowski et al. 1987), southern Sweden (Lundberg and Gustafsson 1995).

**Alphitobiini Reitter, 1917**.

*Alphitobius* **Stephens, 1829**.

*diaperinus* **(Panzer, 1797)**. Pileckis 1984; Silfverberg 1992, 2004; Pileckis and Monsevičius 1997; Fattorini 2009.

[*laevigatus* **(Fabricius, 1781)**]. Imported in Latvia, Estonia, Denmark, Sweden (Lundberg and Gustafsson 1995), known in Poland (Fattorini 2009).

**Helopini Latreille, 1802**.

*Stenomax* **Allard, 1876**.

[*aeneus* **(Scopoli, 1763)** = *lanipes* (Linnaeus, 1771)]. Known in Latvia (Telnov 2004), Poland (Burakowski et al. 1987), northwestern Belarus (Alexandrovich et al. 1996).

*Nalassus* **Mulsant, 1854**.

*dermestoides* **(Illiger, 1798)**. Pileckis 1960, 1976a; Silfverberg 1992, 2004; Gaidienė 1993; Monsevičius 1997; Pileckis and Monsevičius 1997; Ferenca 2006b; Fattorini 2009.

**Bolitophagini Kirby, 1837**.

*Bolitophagus* **Illiger, 1798**.

*reticulatus* **(Linnaeus, 1767)**. Pileckis 1960, 1970c, 1976a; Lešinskas and Pileckis 1967; Silfverberg 1992, 2004; Gaidienė 1993; Monsevičius 1997; Pileckis and Monsevičius 1997; Šablevičius 2000b, 2011; Gliaudys 2001; Tamutis and Zolubas 2001; Vaivilavičius 2008; Löbl et al. 2008.

*Eledonoprius* **Reitter, 1911**.

[*armatus* **(Panzer, 1799)**]. Known in Denmark, southern Sweden (Lundberg and Gustafsson 1995), northern Poland (Burakowski et al. 1987).

*Eledona* **Latreille, 1796**.

*agricola* **(Herbst, 1783)**. Silfverberg 1992, 2004; Pileckis and Monsevičius 1997; Šablevičius 2000a, b, 2001, 2003a, b, 2011; Ferenca 2004; Vaivilavičius 2008; Löbl et al. 2008.

**Palorini Matthews, 2003**.

*Palorus* **Mulsant, 1854**.

*depressus* **(Fabricius, 1790)**. Pileckis 1960, 1970a, 1976a; Zubrys 1967; Pileckis and Monsevičius 1997; Silfverberg 2004.

*ratzeburgi* **(Wissmann, 1848)**. Pileckis 1960, 1976a; Zubrys 1967; Silfverberg 1992, 2004; Pileckis and Monsevičius 1997.

[*subdepressus* **(Wollaston, 1864)**]. Imported in Latvia, Estonia, Denmark, Sweden (Lundberg and Gustafsson 1995), known in Poland (Fattorini 2009).

**Alleculinae** **Laporte, 1840**. (Alleculidae)

**Alleculini Laporte, 1840**.

*Allecula* **Fabricius, 1801**.

[*morio* **(Fabricius, 1787)**]. Known in Latvia (Telnov 2004), Denmark, Sweden (Lundberg and Gustafsson 1995), northern Poland (Burakowski et al. 1987), Kaliningrad region (Alekseev and Bukejs 2010), western Belarus (Alexandrovich et al. 1996).

[*rhenana* **Bach, 1856**].Known in Denmark, southern Sweden (Lundberg and Gustafsson 1995), Poland (Burakowski et al. 1987).

*Hymenorus* **Mulsant, 1852**.

[*doublieri* **(Mulsant, 1851)**]. Known in southern Sweden (Lundberg and Gustafsson 1995), northeastern Poland (Burakowski et al. 1987).

*Prionychus* **Solier, 1835**.

*ater* **(Fabricius, 1775)**. Pileckis 1968a, 1976a, b, 1979; Silfverberg 1992, 2004; Gaidienė 1993; Pileckis and Monsevičius 1997; Ehnström et al. 2003; Šablevičius 2003a; Butvila et al. 2007; Vaivilavičius 2008; Löbl et al. 2008; Fattorini 2009; Inokaitis 2009.

[*melanarius* **(Germar, 1813)**].Known in southern Sweden (Lundberg and Gustafsson 1995), northeastern Poland (Burakowski et al. 1987), Belarus (Alexandrovich et al. 1996).

*Hymenalia* **Mulsant, 1856**.

[*rufipes* **(Fabricius, 1793)**]. Known in Latvia (Telnov 2004), Denmark, southern Sweden (Lundberg and Gustafsson 1995), northern Poland (Burakowski et al. 1987).

*Pseudocistela* **Crotch, 1873**.

*ceramboides* **(Linnaeus, 1758)**. Pileckis 1960, 1976a; Lešinskas and Pileckis 1967; Silfverberg 1992, 2004; Gaidienė 1993; Monsevičius 1997; Pileckis and Monsevičius 1997; Ferenca 2006b; Löbl et al. 2008; Fattorini 2009.

*Gonodera* **Mulsant, 1856**.

[*luperus* **(Herbst, 1783)**]. Known in Estonia, Denmark, southern Sweden (Lundberg and Gustafsson 1995), northern Poland (Burakowski et al. 1987), Belarus (Alexandrovich et al. 1996).

*Isomira* **Mulsant, 1856**.

*murina* **(Linnaeus, 1758)**. Heyden 1903; Pileckis 1960, 1963b, 1976a, b, 1979; Silfverberg 1992, 2004; Gaidienė 1993; Pileckis and Monsevičius 1997; Ferenca et al. 2006, 2007; Löbl et al. 2008; Fattorini 2009; Ivinskis et al. 2009.

[*semiflava* **(Küster, 1852)**]. Known in northern Poland (Burakowski et al. 1987).

*Mycetochara* **Berthold, 1827**.

*axillaris* **(Paykull, 1799)**. Pileckis 1976a; Silfverberg 1992, 2004; Pileckis and Monsevičius 1997; Ehnström et al. 2003; Löbl et al. 2008; Fattorini 2009.

*flavipes* **(Fabricius, 1792)**. Pileckis 1963b, 1976a; Silfverberg 1992, 2004; Gaidienė 1993; Monsevičius 1997; Pileckis and Monsevičius 1997; Ehnström et al. 2003; Šablevičius 2000b, 2003a, b, 2004; Ferenca 2004, 2006a; Ferenca et al. 2006, 2007; Löbl et al. 2008; Fattorini 2009; Inokaitis 2009.

*humeralis* **(Fabricius, 1787)**. Silfverberg 1992, 2004; Gaidienė 1993; Pileckis and Monsevičius 1997; Ehnström et al. 2003; Šablevičius 2004; Ferenca 2006a; Löbl et al. 2008; Fattorini 2009.

*linearis* **(Illiger, 1794)**. Ferenca and Tamutis 2009.

[*obscura* **(Zetterstedt, 1838)**]. Known in Latvia (Telnov 2004), Sweden (Lundberg and Gustafsson 1995), Poland (Fattorini 2009), recently found in Estonia (Süda 2009).

**Cteniopodini, Solier, 1835**.

*Cteniopus* **Solier, 1835**.

*sulphureus* **(Linnaeus, 1758)** = *flavus* (Scopoli, 1763). Eichwald 1830; Roubal 1910; Pileckis 1960, 1963b, 1970b, 1976a, b, 1979; Silfverberg 1992, 2004; Gaidienė 1993; Pileckis and Monsevičius 1997; Šablevičius 2003a; Inokaitis 2004; Löbl et al. 2008; Fattorini 2009; Ivinskis et al. 2009.

*Omophlus* **Dejean, 1834**.

*betulae* **(Herbst, 1783)** = *rufitarsis* (Leske, 1785). Pileckis 1962, 1963b, 1976a, b, 1979; Silfverberg 1992, 2004; Gaidienė 1993; Monsevičius 1997; Pileckis and Monsevičius 1997; Löbl et al. 2008; Fattorini 2009.

[*lividipes* **Mulsant, 1856**]. Known in northern Poland (Burakowski et al. 1987).

**Diaperinae** **Latreille, 1802**.

**Hypophlaeini Billberg, 1820**.

*Hypophloeus* **Fabricius, 1790** = *Corticeus* Piller & Mitterpacher, 1783.

*bicolor* **(Olivier, 1790)**. Pileckis 1960, 1976a; Silfverberg 1992, 2004; Gaidienė 1993; Pileckis and Monsevičius 1997; Ferenca 2006b; Löbl et al. 2008; Fattorini 2009.

*fasciatus* **(Fabricius, 1790)**. Pileckis and Monsevičius 1997; Šablevičius 2003a; Silfverberg 2004; Ferenca et al. 2006, 2007.

*fraxini* **(Kugelann, 1794)** Pileckis 1960, 1976a, 1982; Silfverberg 1992, 2004; Gaidienė 1993; Pileckis and Monsevičius 1997; Ferenca 2006b; Löbl et al. 2008; Fattorini 2009.

*linearis* **(Fabricius, 1790)**. Valenta and Jakaitis 1972; Jakaitis 1973; Silfverberg 1992, 2004; Gaidienė 1993; Pileckis and Monsevičius 1997; Gedminas 2005; Löbl et al. 2008; Fattorini 2009; Šablevičius 2011.

*longulus* **(Gyllenhal, 1827)**. Horion 1956; Pileckis 1968b, 1976a; Silfverberg 1992, 2004; Pileckis and Monsevičius 1997; Šablevičius 2003a; Löbl et al. 2008; Fattorini 2009.

*pini* **(Panzer, 1799)**. Pileckis and Jakaitis 1982; Pileckis and Monsevičius 1997; Šablevičius 2001, 2003a; Silfverberg 2004.

*suturalis* **(Paykull, 1800)**. Pileckis and Jakaitis 1982; Pileckis and Monsevičius 1997; Šablevičius 2003a; Silfverberg 1992, 2004; Löbl et al. 2008; Fattorini 2009.

*unicolor* **Piller & Mitterpacher, 1783**. Pileckis and Monsevičius 1982, 1997; Silfverberg 1992, 2004; Monsevičius 1997; Ehnström et al. 2003; Šablevičius 2003b, 2011; Ferenca 2004; Vaivilavičius 2008; Löbl et al. 2008; Fattorini 2009.

**Phaleriini Blanchard, 1845**.

*Phaleria* **Latreille, 1802**.

[*cadaverina* **(Fabricius, 1792)**]. Known in Denmark, southern Sweden (Lundberg and Gustafsson 1995).

**Crypticini Brullé, 1832**.

*Crypticus* **Latreille, 1817**.

*quisquilius* **(Linnaeus, 1761)**. Mazurowa and Mazur 1939; Pileckis 1960, 1970b, c, 1976a; Silfverberg 1992, 2004; Gaidienė 1993; Monsevičius 1997; Pileckis and Monsevičius 1997; Žiogas and Zolubas 2005; Ferenca 2006b; Löbl et al. 2008; Fattorini 2009; Šablevičius 2011.

**Diaperini Latreille, 1802**.

*Diaperis* **Geoffroy, 1762**.

*boleti* **(Linnaeus, 1758)**. Pileckis 1960, 1970c, 1976a; Lešinskas and Pileckis 1967; Silfverberg 1992, 2004; Gaidienė 1993; Monsevičius 1997; Pileckis and Monsevičius 1997; Šablevičius 2000b, 2011; Gliaudys 2001; Ferenca 2006b; Vaivilavičius 2008; Löbl et al. 2008; Fattorini 2009.

*Gnatocerus* **Thunberg, 1814**.

*cornutus* **(Fabricius, 1798)**. Lundberg and Gustafsson 1995; Löbl et al. 2008; Fattorini 2009

*Neomida* **Dahl, 1823** = *Oplocephala* Laporte & Brullé, 1831.

*haemorrhoidalis* **(Fabricius, 1787)**. Pileckis 1976a; Silfverberg 1992, 2004; Gaidienė 1993; Pileckis and Monsevičius 1997; Ehnström et al. 2003; Šablevičius 2000a, 2001, 2003a, b, 2011; Ferenca 2004, 2006a; Ferenca et al. 2006, 2007; Butvila et al. 2007; Alekseev and Nikitsky 2008; Alekseev 2008a; Löbl et al. 2008; Fattorini 2009; Ivinskis et al. 2009.

*Alphitophagus* **Stephens, 1832**.

*bifasciatus* **(Say, 1823)**. Pileckis 1968b, 1976; Silfverberg 1992, 2004; Pileckis and Monsevičius 1997; Šablevičius 2003a; Löbl et al. 2008; Fattorini 2009.

*Platydema* **Laporte & Brullé, 1831**.

[*dejeani* **(Laporte & Brullé, 1831)**]. Known in northwestern Belarus (Alexandrovich et al. 1996), eastern Poland (Burakowski et al. 1987).

*violaceum* **(Fabricius, 1790)**. Ferenca 1988; Silfverberg 1992, 2004; Gaidienė 1993; Pileckis and Monsevičius 1997; Ehnström et al. 2003; Löbl et al. 2008; Fattorini 2009.

*Pentaphyllus* **Dejean, 1821**.

[*testaceus* **(Hellwig, 1792)**]. Known in Latvia (Telnov 2004), Estonia, Denmark, southern Sweden (Lundberg and Gustafsson 1995), northern Poland (Burakowski et al. 1987), Belarus (Alexandrovich et al. 1996).

**Scaphidemini Reitter, 1922**.

*Scaphidema* **Redtenbacher, 1849**.

*metallicum* **(Fabricius, 1793)**. Gaidienė and Ferenca 1988; Silfverberg 1992, 2004; Gaidienė 1993; Pileckis and Monsevičius 1997; Ferenca et al. 2002, 2006, 2007; Šablevičius 2004; Löbl et al. 2008; Fattorini 2009.

**Myrmechixenini Jacquelin du Val, 1858**.

*Myrmechixenus* **Chevrolat, 1835**.

*subterraneus* **Chevrolat, 1835**. Barševskis 2001a; Monsevičius and Pankevičius 2001; Silfverberg 2004.

[*vaporariorum* **Guérin-Ménéville, 1843**].Known in Demark, Sweden (Lundberg and Gustafsson 1995), northern Poland (Burakowski et al. 1987), Belarus (Alexandrovich et al. 1996).

**PROSTOMIDAE Thomson, 1859**.

*Prostomis* **Latreille, 1829**.

[*mandibularis* **(Fabricius, 1801)**]. Known in Demark, Sweden (Lundberg and Gustafsson 1995), northern Poland (Burakowski et al. 1987), Belarus (Alexandrovich et al. 1996).

**STENOTRACHELIDAE Thomson, 1859** = cephaloidae LeConte, 1862.

*Scotodes* **Eschscholtz, 1818**.

*annulatus* **Eschscholtz, 1818**. Audisio 2009.

**OEDEMERIDAE Latreille, 1810**.

**Calopodinae** **Costa, 1852**.

*Calopus* **Fabricius, 1775**.

*serraticornis* **(Linnaeus, 1758)**. Pileckis 1968a, 1976a; Silfverberg 1992, 2004; Gaidienė 1993; Pileckis and Monsevičius 1997; Löbl 2008; Vázquez-Albalate 2009.

**Oedemerinae** **Latreille, 1810**.

**Nacerdini Mulsant, 1858**.

*Nacerdes* **Dejean, 1834** = *Xanthochroa* W. Schmidt, 1844.

[*carniolica* **(Gistel, 1832)**].Known in Sweden (Lundberg and Gustafsson 1995), Belarus (Alexandrovich et al. 1996).

*melanura* **(Linnaeus, 1758)**. Silfverberg 1992, 2004; Pileckis and Monsevičius 1997; Löbl 2008; Vázquez-Albalate 2009.

*Anogcodes* **Dejean, 1834** = *Anoncodes* Dejean, 1834.

*ferrugineus* **(Schrank, 1776)** = *adusta* (Panzer, 1795). Gaidienė 1993; Pileckis and Monsevičius 1997; Silfverberg 2004; Inokaitis 2004; Löbl 2008.

*rufiventris* **(Scopoli, 1763)**. Pileckis 1976a; Silfverberg 1992, 2004; Pileckis and Monsevičius 1997; Šablevičius 2004; Inokaitis 2004; Löbl 2008; Vázquez-Albalate 2009.

*ustulatus* **(Scopoli, 1763)** = *ustulata*(Fabricius, 1787).Pileckis 1960, 1968b, 1976a; Silfverberg 1992, 2004; Gaidienė 1993; Monsevičius 1997; Pileckis and Monsevičius 1997; Inokaitis 2004; Löbl 2008; Vázquez-Albalate 2009.

**Ditylini Mulsant, 1858**.

*Ditylus* **Fischer von Waldheim, 1817**.

*laevis* **(Fabricius, 1787)**. Pileckis 1976a; Silfverberg 1992, 2004; Pileckis and Monsevičius 1997; Löbl 2008; Vázquez-Albalate 2009.

*Chrysanthia* **W. Schmidt, 1844**.

*geniculata* **Heyden, 1877** = *nigricornis* (Westhoff, 1881) = *viridis* W. Schmidt, 1846, nec (DeGeer, 1775). Mazurowa and Mazur 1939; Pileckis 1960, 1976a; Silfverberg 1992, 2004; Gaidienė 1993; Pileckis and Monsevičius 1997; Gliaudys 2001; Ferenca 2006b; Löbl 2008; Vázquez-Albalate 2009; Ostrauskas and Ferenca 2010.

*viridissima* (**Linnaeus, 1758)** = *viridis* (DeGeer, 1775). Pileckis 1960, 1976a; Lešinskas and Pileckis 1967; Silfverberg 1992, 2004; Monsevičius 1997; Pileckis and Monsevičius 1997; Šablevičius 2000b; Žiogas and Zolubas 2005; Löbl 2008; Vázquez-Albalate 2009.

**Asclerini Gistel, 1848**.

*Ischnomera* **Stephens, 1832** = *Asclera* Dejean, 1834.

[*caerulea* **(Linnaeus, 1758)**].Known in southern Sweden (Lundberg and Gustafsson 1995), northern Poland (Burakowski et al. 1987).

[*cinerascens cinerascens* **(Pandellé in Grenier, 1867)**].Known in Demark, southern Sweden (Lundberg and Gustafsson 1995), Poland (Burakowski et al. 1987).

[*cyanea* **(Fabricius, 1792)**].Known in Demark, southern Sweden (Lundberg and Gustafsson 1995), Poland (Vázquez-Albalate 2009).

*sanguinicollis* **(Fabricius, 1787)**. Pileckis and Monsevičius 1982, 1997; Silfverberg 1992, 2004; Monsevičius 1997; Löbl 2008; Vázquez-Albalate 2009.

**Oedemerini Latreille, 1810**.

*Oedemera* **Olivier, 1789**.

[*croceicollis* **(Gyllenhal, 1827)**].Known in Estonia, Demark, Sweden (Lundberg and Gustafsson 1995), northern Poland (Burakowski et al. 1987).

*femorata* **(Scopoli, 1763)** = *flavescens* (Linnaeus, 1767). Pileckis 1968b, 1976a; Silfverberg 1992, 2004; Gaidienė 1993; Pileckis and Monsevičius 1997; Vaivilavičius 2008; Ivinskis et al. 2009; Ostrauskas and Ferenca 2010.

*flavipes* **(Fabricius, 1792)**. Pileckis 1962, 1963b, 1976a, b, 1979; Silfverberg 1992, 2004; Gaidienė 1993; Pileckis and Monsevičius 1997; Löbl 2008.

*lurida* **(Marsham, 1802)**. Pileckis 1960, 1976a; Zajančkauskas and Pileckis 1968; Silfverberg 1992, 2004; Gaidienė 1993; Monsevičius 1997; Pileckis and Monsevičius 1997; Ferenca 2006b; Löbl 2008; Vázquez-Albalate 2009.

*podagrariae* **(Linnaeus, 1767)**. Pileckis 1968b, 1976a, b, 1979; Silfverberg 1992, 2004; Pileckis and Monsevičius 1997; Löbl 2008; Vázquez-Albalate 2009.

[*pthysica* **(Scopoli, 1763)** = *subulata* Olivier, 1795]. Known in Demark, southern Sweden (Lundberg and Gustafsson 1995), northeastern Poland (Burakowski et al. 1987).

*subrobusta* **(Nakane, 1954)**. Barševskis 2008.

*virescens* **(Linnaeus, 1767)**. Pileckis 1960, 1976a; Silfverberg 1992, 2004; Gaidienė 1993; Monsevičius 1997; Pileckis and Monsevičius 1997; Šablevičius 2000b; Ferenca 2006b; Löbl 2008; Vázquez-Albalate 2009.

**MELOIDAE Gyllenhal, 1810**.

**Meloinae** **Gyllenhal, 1810**.

**Cerocomini Leach, 1815**.

*Cerocoma* **Geoffroy, 1762**.

*schaefferi* **(Linnaeus, 1758)**. Eichwald 1830; Tamutis 2003.

**Lyttini Solier, 1851**.

*Lytta* **Fabricius, 1775**.

*vesicatoria* **(Linnaeus, 1758)**. Eichwald 1830; Heyden 1903; Ivanauskas and Vailionis 1922; Ogyjewicz 1938; Mazurowa and Mazur 1939; Pileckis 1960, 1976a; Lešinskas and Pileckis 1967; Pileckis et al. 1968; Silfverberg 1992, 2004; Gaidienė 1993; Pileckis and Monsevičius 1997; Skeiveris and Paplauskis 1998; Ferenca 2006b; Bologna 2008, 2009; Ivinskis et al. 2009; Šablevičius 2011.

**Meloini Gyllenhal, 1810**.

*Meloe* **Linnaeus, 1758**.

*brevicollis* **Panzer, 1793**. Pileckis 1960, 1963b, 1976a; Silfverberg 1992, 2004; Pileckis and Monsevičius 1997; Ferenca 2006b; Ferenca et al. 2006, 2007; Bologna 2008, 2009; Ivinskis et al. 2009; Liekis 2009.

*coriarius* **Brandt & Erichson, 1832**]. Known in northern Poland (Burakowski et al. 1987).

*proscarabaeus* **Linnaeus, 1758**. Eichwald 1830; Pileckis 1960, 1976a; Lešinskas and Pileckis 1967; Zajančkauskas and Pileckis 1968; Gaidienė and Ferenca 1992; Silfverberg 1992, 2004; Gaidienė 1993; Monsevičius 1997; Pileckis and Monsevičius 1997; Šablevičius 2000b, 2011; Gliaudys 2001; Ferenca 2006b; Bologna 2008, 2009; Liekis 2009.

[*scabriusculus* **Brandt & Erichson, 1832**]. Known in northern Poland (Burakowski et al. 1987).

*variegatus* **Donovan, 1793**. Pileckis 1976a; Silfverberg 1992, 2004; Pileckis and Monsevičius 1997; Bologna 2008, 2009.

*violaceus* **Marsham, 1802**. Pileckis 1960, 1963b, 1976a; Silfverberg 1992, 2004; Monsevičius 1997; Pileckis and Monsevičius 1997; Monsevičius 1998; Ferenca 2006b; Bologna 2008, 2009; Ivinskis et al. 2009; Liekis 2009.

**Nemognathinae** **Laporte, 1840**.

**Nemognathini Laporte, 1840**.

*Apalus* **Fabricius, 1775**.

*bimaculatus* **(Linnaeus, 1761)**.Bologna 2008, 2009.

*Stenoria* **Mulsant, 1857**.

[*analis* **Schaum 1859**].Known in northern Poland (Burakowski et al. 1987).

**MYCTERIDAE Oken, 1843**.

**Mycterinae Oken, 1843**.

*Mycterus* **Clairville & Schellenberg, 1798**.

[*curculioides* **(Fabricius, 1781)**].Known in Latvia (Telnov 2004), Poland (Burakowski et al. 1987).

**BORIDAE Thomson, 1859**.

**Borinae Thomson, 1859**

*Boros* **Herbst, 1797**.

RDB*schneideri* **(Panzer, 1795)**. Łomnicki 1913; Pileckis 1968b, 1976a; Jakaitis and Valenta 1976; Balevičius 1992; Silfverberg 1992, 2004; Gaidienė 1993; Šablevičius 1994, 1998, 2001, 2003a, b, 2011; Ivinskis et al. 1996b, 2007; Monsevičius 1997; Pileckis and Monsevičius 1997; Čeponis and Šablevičius 1997; Ferenca 2003, 2004; Ehnström et al. 2003; Meržijevskis 2004; Tamutis 2005b; Karalius et al. 2006; Masiulis 2007; Uselis et al. 2007; Rašomavičius 2007; Pollock 2008; Švitra and Aliukonis 2009; Vázquez-Albalate 2009; Alekseev 2010b; Blažytė-Čereškienė and Karalius 2010.

**PYTHIDAE Solier, 1834**.

*Pytho* **Latreille, 1796**.

*depressus* **(Linnaeus, 1767)**. Eichwald 1830; Pileckis 1960, 1976a; Jakaitis and Valenta 1976; Strazdienė 1988; Silfverberg 1992, 2004; Gaidienė 1993; Monsevičius 1997; Pileckis and Monsevičius 1997; Tamutis and Zolubas 2001; Ehnström et al. 2003; Šablevičius 2003b, 2011; Ferenca 2006a; Pollock 2008; Vázquez-Albalate 2009; Ivinskis et al. 2009.

*kolwensis* **Sahlberg, 1833**. Vazquez – Albalate 2010.

**PYROCHROIDAE Latreille, 1806. **

**Pyrochroinae Latreille, 1806**.

*Pyrochroa* **Geoffroy, 1762**.

*coccinea* **(Linnaeus, 1761)**. Pileckis 1960, 1976a; Lešinskas and Pileckis 1967; Silfverberg 1992, 2004; Gaidienė 1993; Monsevičius 1997; Pileckis and Monsevičius 1997; Šablevičius 2000b, 2011; Gliaudys 2001; Gedminas 2005; Ferenca 2006b; Pollock and Young 2008.

*serraticornis* **(Scopoli, 1763)**. Pileckis 1976a; Silfverberg 1992, 2004; Pileckis and Monsevičius 1997; Gedminas et al. 2007; Pollock and Young 2008.

*Schizotus* **Newman, 1838**.

*pectinicornis* **(Linnaeus, 1758)**. Pileckis 1976a; Silfverberg 1992, 2004; Gaidienė 1993; Pileckis and Monsevičius 1997; Gliaudys 2001; Pollock and Young 2008; Šablevičius 2011.

**SALPINGIDAE Leach, 1815**.

**Agleninae** **Horn, 1878**.

*Aglenus* **Erichson, 1845**.

[*brunneus* **(Gyllenhal, 1813)**].Known in Demark, southern Sweden (Lundberg and Gustafsson 1995).

**Salpinginae** **Leach, 1815**.

*Lissodema* **Curtis, 1833**.

*cursor* **(Gyllenhal, 1813)**. Tamutis et al. 2008.

[*denticolle* **(Gyllenhal, 1813)** = *quadripustulatum* (Marsham, 1802) nec (Fabricius, 1775)].Known in Demark, southern Sweden (Lundberg and Gustafsson 1995), northern Poland (Burakowski et al. 1987).

*Colposis* **Mulsant, 1859**.

*mutilatus* **(Beck, 1817)**. Roubal 1910; Pileckis 1960, 1976a; Silfverberg 1992, 2004; Pileckis and Monsevičius 1997 (*Rabocerus*); Pollock and Löbl 2008; Vázquez-Albalate 2009; Ivinskis et al. 2009.

*Rabdocerus* **Mulsant, 1859**.

*foveolatus* **(Ljungh, 1823)**. Monsevičius 1988b; Silfverberg 1992, 2004; Pileckis and Monsevičius 1997 (*Rabocerus*); Pollock and Löbl 2008; Vázquez-Albalate 2009.

*gabrieli* **(Gerhardt, 1901)**. Pileckis and Monsevičius 1997 (*Rabocerus*); Monsevičius 1998; Silfverberg 2004; Ivinskis et al. 2009.

*Sphaeriestes* **Stephens, 1829** = *Salpingus* auct. nec Illiger, 1801.

*bimaculatus* **(Gyllenhal, 1810)**. Pileckis 1976a; Pileckis and Monsevičius 1982, 1997; Silfverberg 1992, 2004; Pollock and Löbl 2008; Vázquez-Albalate 2009.

*castaneus* **(Panzer, 1796)**. Pileckis 1976a; Silfverberg 1992, 2004; Pileckis and Monsevičius 1997; Vázquez-Albalate 2009.

[*reyi* **(Abeille de Perrin, 1874)**].Known in Demark, southern Sweden (Lundberg and Gustafsson 1995), northern Poland (Burakowski et al. 1987).

*stockmanni* **(Biström, 1977)** = *ater* (Paykull, 1798) nec (DeGeer, 1774). Pileckis and Monsevičius 1997; Tamutis and Zolubas 2001; Silfverberg 2004.

*Vincenzellus* **Reitter, 1911**.

[*ruficollis* **(Panzer, 1794)** = *viridipennis* (Latreille, 1804)]. Known in Demark, southern Sweden (Lundberg and Gustafsson 1995), northern Poland (Burakowski et al. 1987).

*Salpingus* **Illiger, 1801** = *Rhinosimus* Latreille, 1802.

[*aeneus* **(Olivier, 1807)**].Known in Latvia (Telnov 2004), Estonia (Silfverberg 2004), northern Poland (Burakowski et al. 1987), northwestern Belarus (Alexandrovich et al. 1996).

*planirostris* **(Fabricius, 1787)**. Miländer et al. 1984; Silfverberg 1992, 2004; Gaidienė 1993; Pileckis and Monsevičius 1997; Tamutis and Zolubas 2001; Šablevičius 2003a; Ferenca 2004; Pollock and Löbl 2008; Vázquez-Albalate 2009.

*ruficollis* **(Linnaeus, 1761)**. Pileckis 1968a, 1976a; Silfverberg 1992, 2004; Gaidienė 1993; Pileckis and Monsevičius 1997; Šablevičius 2000a, 2001, 2003a; Butvila et al. 2007; Pollock and Löbl 2008; Vázquez-Albalate 2009.

**ANTHICIDAE Latreille, 1819**.

**Anthicinae** **Latreille, 1819**.

**Formicomini Bonadona, 1974**.

*Anthelephila* **Hope, 1833** = *Formicomus* LaFerté-Sénectère, 1849.

[*pedestris* **(P. Rossi, 1790)**]. Known in Latvia (Telnov 2004), Poland (Burakowski et al. 1987).

**Anthicini Latreille, 1819**.

*Cyclodinus* **Mulsant & Rey, 1866**.

[*humilis* **(Germar, 1824)** = *sibiricus* auct. nec (Pic, 1893)]. Known in Demark, southern Sweden (Lundberg and Gustafsson 1995), northern Poland (Burakowski et al. 1987).

*Omonadus* **Mulsant & Rey, 1866**.

[*bifasciatus* **(P. Rossi, 1792)** = *kolenatii* (Kolenati, 1846)]. Known in Demark, southern Sweden (Lundberg and Gustafsson 1995), Poland (Burakowski et al. 1987).

*floralis* **(Linnaeus, 1758)**. Pileckis 1968a, 1976a; Silfverberg 1992, 2004; Gaidienė 1993; Pileckis and Monsevičius 1997 (*Anthicus*); Šablevičius 2003a; Chandler et al. 2008; Nardi 2009.

*formicarius* **(Goeze, 1777)** = *quisquilius* (Thomson, 1864). Pileckis 1960, 1976a; Silfverberg 1992, 2004; Pileckis and Monsevičius 1997 (*Anthicus*); Chandler et al. 2008; Nardi 2009; Ferenca et al. 2010.

*Cordicollis* **Marseul, 1879** = *Cordicomus* Pic, 1894.

*gracilis* **(Panzer, 1797)**. Monsevičius 1988b, 1997; Silfverberg 1992, 2004; Pileckis and Monsevičius 1997 (*Anthicus*); Nardi 2009 Ferenca et al. 2010.

[*instabilis instabilis* **(W.L.E. Schmidt, 1842)**].Known in Demark, southern Sweden (Lundberg and Gustafsson 1995), Poland (Nardi 2009).

*Stricticollis* **Marseul, 1879** = *Stricticomus* Pic, 1894.

*tobias* **(Marseul, 1879)**. Barševskis 2001; Silfverberg 2004; Nardi 2009.

*Anthicus* **Paykull, 1798**.

*antherinus* **(Linnaeus, 1761)**. Pileckis 1968b, 1976a; Zajančkauskas and Pileckis 1968; Silfverberg 1992, 2004; Gaidienė 1993; Monsevičius 1997; Pileckis and Monsevičius 1997; Šablevičius 2003a; Ferenca 2004; Chandler et al. 2008; Nardi 2009; Ferenca et al. 2010.

*ater* **(Thunberg, 1787)**. Jakaitis 1973; Silfverberg 1992, 2004; Monsevičius 1997; Pileckis and Monsevičius 1997; Ferenca 2004; Chandler et al. 2008; Nardi 2009; Ivinskis et al. 2009; Ferenca et al. 2010.

*axillaris* **W.L.E. Schmidt, 1842**. Silfverberg 1992, 2004; Pileckis and Monsevičius 1997; Chandler et al. 2008; Nardi 2009.

*bimaculatus* **Illiger, 1801**. Silfverberg 1992, 2004; Pileckis and Monsevičius 1997; Šablevičius 2003a; Ferenca et al. 2006, 2007; Alekseev 2008a; Chandler et al. 2008; Nardi 2009; Ivinskis et al. 2009; Ferenca et al. 2010.

*flavipes* **(Panzer, 1796)**. Mazurowa and Mazur 1939; Pileckis 1960, 1976a; Silfverberg 1992, 2004; Gaidienė 1993; Pileckis and Monsevičius 1997; Šablevičius 2003a; Ferenca 2004; Chandler et al. 2008; Nardi 2009; Ferenca et al. 2010.

*sellatus* **(Panzer, 1796)**. Pileckis 1960, 1976a; Silfverberg 1992, 2004; Pileckis and Monsevičius 1997; Ferenca 2004, 2006b; Chandler et al. 2008; Nardi 2009; Ferenca et al. 2010.

[*umbrinus* **LaFerté-Sénectère, 1849**].Known in Latvia (Telnov 2004), Sweden (Lundberg and Gustafsson 1995).

*Hirticomus* **Pic, 1894**.

[*hispidus* **(Rossi, 1792)**]. Known in Latvia (Telnov 2004), southern Sweden (Lundberg and Gustafsson 1995), northern Poland (Burakowski et al. 1987).

**Notoxinae** **Stephens, 1829**.

*Notoxus* **Geoffroy, 1762**.

*monoceros* **(Linnaeus, 1761)**. Heyden 1903; Mazurowa and Mazur 1939; Pileckis 1960, 1976a; Silfverberg 1992, 2004; Gaidienė 1993; Pileckis and Monsevičius 1997; Monsevičius 1997, 1998; Šablevičius 2000b; Gliaudys 2001; Tamutis and Zolubas 2001; Žiogas and Zolubas 2005; Ferenca 2006b; Chandler et al. 2008; Nardi 2009; Ostrauskas and Ferenca 2010; Ferenca et al. 2010.

*Mecynotarsus* **LaFerté-Sénectère, 1847**.

*serricornis* **(Panzer, 1796)**. Tamutis 2003.

**ADERIDAE Csiki, 1909**.

**Phytobaenini Báguena Corella, 1948**.

*Phytobaenus* **R.F. Sahlberg, 1834**.

[*amabilis* **R.F. Sahlberg, 1834**]. Known in Latvia (Telnov 2004), southern Sweden (Lundberg and Gustafsson 1995), Estonia (Silfverberg 2004), western Belarus (Alexandrovich et al. 1996), northern Poland (Burakowski et al. 1987), recently found in Kaliningrad region (Alekseev and Bukejs 2010).

**Aderini Csiki, 1909**.

*Aderus* **Stephens, 1829** = *Hylophilus* Berthold, 1827, nec Temminck, 1822.

*populneus* **(Creutzer, 1796)**. Pileckis 1976a; Silfverberg 1992, 2004; Pileckis and Monsevičius 1997; Barševskis 2001a; Ferenca 2004; Nardi 2009, 2008.

**Euglenesini Seidlitz, 1875**.

*Euglenes* **Westwood, 1830**.

[*oculatus* **(Paykull, 1798)**]. Known in Latvia (Telnov 2004), Denmark, southern Sweden (Lundberg and Gustafsson 1995), western Belarus (Alexandrovich et al. 1996), northern Poland (Burakowski et al. 1987).

*pygmaeus* **(DeGeer, 1775)** = *lokvenci* (Roubal, 1938). Barševskis 2001a; Silfverberg 2004; Ferenca et al. 2006, 2007; Ivinskis et al. 2009.

*Anidorus* **Mulsant & Rey, 1866**.

*nigrinus* **(Germar, 1842)**. Pileckis 1976a; Silfverberg 1992, 2004; Pileckis and Monsevičius 1997; Barševskis 2001a; Ferenca 2004, 2005, 2006, 2007; Nardi 2009, 2008; Alekseev and Nikitsky 2008.

*Pseudanidorus* **Pic, 1893** = *Pseudeuglenes* Pic, 1897.

[*pentatomus* **(Thomson, 1864)**]. Known in southern Sweden (Lundberg and Gustafsson 1995), recently found in Estonia (Süda 2009).

*Vanonus* **Casey, 1895**.

[*brevicornis* **(Perris, 1869)**]. Known in Demark, southern Sweden (Lundberg and Gustafsson 1995).

**SCRAPTIIDAE Gistel, 1848**. (Anaspidae)

**Scraptiinae** **Gistel, 1848**.

**Scraptiini Gistel, 1848**.

*Scraptia* **Latreille, 1807**.

[*fuscula* **O.F. Müller, 1821** = *innata* (Kangas, 1959)]. Known in Latvia (Telnov 2004), Denmark, southern Sweden (Lundberg and Gustafsson 1995), western Belarus (Alexandrovich et al. 1996), Poland (Horak 2009), recently found in Estonia (Süda 2009).

**Anaspidinae** **Mulsant, 1856**.

**Anaspidini Mulsant, 1856**.

*Cyrtanaspis* **Emery, 1876**.

[*phalerata* **(Germar, 1847)**]. Known in Latvia (Telnov 2004), Estonia, southern Sweden (Lundberg and Gustafsson 1995), western Belarus (Alexandrovich et al. 1996), northern Poland (Burakowski et al. 1987).

*Anaspis* **Geoffroy, 1762**.

[*arctica* **Zetterstedt, 1828**]. Known in Latvia (Telnov 2004), Estonia (Silfverberg 2004), northern Sweden (Lundberg and Gustafsson 1995), Poland (Burakowski et al. 1987).

[*bohemica* **Schilsky, 1898** = *norvegica* Munster, 1924]. Known in Latvia (Telnov 2004), Denmark, southern Sweden (Lundberg and Gustafsson 1995), Poland (Burakowski et al. 1987), recently found in Estonia (Süda 2009).

*brunnipes* **(Mulsant, 1856)**. Pileckis 1976a; Silfverberg 1992, 2004; Gaidienė 1993; Pileckis and Monsevičius 1997.

[*costai* **Emery, 1876**]. Known in Denmark, (Lundberg and Gustafsson 1995), northern Poland (Burakowski et al. 1987).

*flava* **(Linnaeus, 1758)**. Gaidienė 1993; Pileckis and Monsevičius 1997; Silfverberg 2004.

*frontalis* **(Linnaeus, 1758)**. Heyden 1903; Pileckis 1960, 1963b, 1976a; Silfverberg 1992, 2004; Gaidienė 1993; Pileckis and Monsevičius 1997; Šablevičius 2000b; Žiogas and Zolubas 2005; Ferenca 2006b; Ostrauskas and Ferenca 2010.

[*garneysi* **Fowler, 1889**]. Known in Latvia (Telnov 2004), Denmark, southern Sweden (Lundberg and Gustafsson 1995).

[*lurida* **Stephens, 1832**].Known in Latvia (Telnov 2004), Poland (Burakowski et al. 1987).

[*marginicollis* **Lindberg, 1925**].Known in Latvia (Telnov 2004), Estonia, Denmark, throughout Sweden (Lundberg and Gustafsson 1995), northern Poland (Burakowski et al. 1987).

[*melanopa* **(Forster, 1771)** = *maculata* Fourcroy, 1785].Known in Denmark, southern Sweden (Lundberg and Gustafsson 1995), Poland (Burakowski et al. 1987).

*melanostoma* **Costa, 1854**. Silfverberg 1992, 2004; Lundberg and Gustafsson 1995.

[*palpalis* **Gerhardt, 1876**].Known in northern Poland (Burakowski et al. 1987).

*pulicaria* **Costa, 1854**. Pileckis 1960, 1976a; Silfverberg 1992, 2004; Gaidienė 1993; Pileckis and Monsevičius 1997; Ferenca 2006b.

[*regimbarti* **Schilsky, 1895**].Known in Denmark, southern Sweden (Lundberg and Gustafsson 1995), Poland (Horak 2009).

*ruficollis* **(Fabricius, 1793)**. Gaidienė 1993; Silfverberg 2004.

*rufilabris* **(Gyllenhal, 1827)**. Pileckis 1968b, 1976a; Silfverberg 1992, 2004; Gaidienė 1993; Pileckis and Monsevičius 1997; Ostrauskas and Ferenca 2010.

*thoracica* **(Linnaeus, 1758)**. Pileckis 1960, 1976a; Silfverberg 1992, 2004; Gaidienė 1993; Pileckis and Monsevičius 1997; Ivinskis et al. 2009.

[*varians* **(Mulsant, 1856)**].Known in Latvia, Estonia (Lundberg and Gustafsson 1995), northern Poland (Burakowski et al. 1987).

**CHRYSOMELOIDEA Latreille, 1802**.

**CERAMBYCIDAE Latreille, 1802**.

**Prioninae** **Latreille, 1802**.

**Callipogonini Thomson, 1861**.

*Ergates* **Audinet-Serville, 1832**.

RDB*faber* **(Linnaeus, 1761)**. Eichwald 1830; Zawadzki 1936; Stanilisówna 1939; Pileckis 1959, 1960, 1970b, 1976a, 1982; Lešinskas and Pileckis 1967; Pileckis et al. 1968; Balevičius 1992; Silfverberg 1992, 2004; Gaidienė 1993; Obelevičius 1994; Ivinskis et al. 1996b, 1997a, 2004a; Monsevičius 1997; Pileckis and Monsevičius 1997; Althoff and Danilevsky 1997; Šablevičius 2000a, 2003a, 2004, 2011; Gliaudys 2001; Danilevsky 2003; Ehnström et al. 2003; Ivinskis 2003; Meržijevskis 2004; Ferenca 2006b; Rašomavičius 2007; Alekseev 2007, 2010b; Sama 2009; Drumont and Komiya 2010.

**Prionini Latreille, 1802**.

*Prionus* **Geoffroy, 1762**.

RDB*coriarius* **(Linnaeus, 1758)**. Eichwald 1830; Zawadzki 1936; Stanilisówna 1939; Mazurowa and Mazur 1939; Pileckis 1960, 1976a; Lešinskas and Pileckis 1967; Pileckis et al. 1968; Ivinskis et al. 1984, 2004a; Silfverberg 1992, 2004; Gaidienė 1993; Monsevičius 1997; Pileckis and Monsevičius 1997; Althoff and Danilevsky 1997; Gliaudys 2001; Danilevsky 2003; Ehnström et al. 2003; Šablevičius 2003a, 2007, 2011; Dapkus 2004; Ferenca 2006b; Rašomavičius 2007; Alekseev 2007, 2010b; Vaivilavičius 2008; Sama 2009; Bačianskas 2009; Ivinskis et al. 2009; Drumont and Komiya 2010.

**Meroscelisini Thomson, 1861**.

*Tragosoma* **Audinet-Serville, 1832**.

*depsarium* **(Linnaeus, 1767)**. Zawadzki 1936; Pileckis 1960, 1970a, 1976a, b, 1979, 1982; Silfverberg 1992, 2004; Monsevičius 1997; Pileckis and Monsevičius 1997; Althoff and Danilevsky 1997; Danilevsky 2003; Ehnström et al. 2003; Alekseev 2007; Sama 2009; Drumont and Komiya 2010.

**Lepturinae** **Latreille, 1802**.

**Oxymirini Danilevsky, 1997**.

*Oxymirus* **Mulsant, 1858**.

*cursor* **(Linnaeus, 1758)**. Zawadzki 1936; Stanilisówna 1939; Pileckis 1960, 1976a; Zajančkauskas and Pileckis 1968; Silfverberg 1992, 2004; Gaidienė 1993; Monsevičius 1997; Pileckis and Monsevičius 1997; Althoff and Danilevsky 1997; Gliaudys 2001; Danilevsky 2003; Inokaitis 2004; Ferenca 2006b; Alekseev 2007; Sama 2009; Ivinskis et al. 2009; Sama and Löbl 2010.

**Rhamnusiini Sama, 2009**.

*Rhamnusium* **Latreille, 1829**.

*bicolor* **(Schrank, 1781)** = *gracilicorne* Théry, 1895. # 67.Zawadzki 1936; Stanilisówna 1939; Pileckis 1960, 1976a; Zajančkauskas and Pileckis 1968; Silfverberg 1992, 2004; Gaidienė 1993; Monsevičius 1997; Pileckis and Monsevičius 1997 (*Rh. virgo = bicolor*); Althoff and Danilevsky 1997; Ehnström et al. 2003; Danilevsky 2003; Ferenca 2006b; Alekseev 2007; Sama 2009; Sama and Löbl 2010.

**Rhagiini Kirby, 1837**.

*Rhagium* **Fabricius, 1775**.

*bifasciatum* **Fabricius, 1775**. Althoff and Danilevsky 1997; Danilevsky 2003; Silfverberg 2004; Alekseev 2007; Sama 2009; Sama and Löbl 2010.

*inquisitor* **(Linnaeus, 1758)**. Eichwald 1830; Heyden 1903; Ivanauskas and Vailionis 1922; Zawadzki 1936; Stanilisówna 1939; Pileckis 1960, 1976a; Lešinskas and Pileckis 1967; Pileckis et al. 1968; Jakaitis and Valenta 1976; Silfverberg 1992, 2004; Gaidienė 1993; Žiogas and Gedminas 1994; Monsevičius 1997; Pileckis and Monsevičius 1997; Žiogas 1997; Althoff and Danilevsky 1997; Valenta 2000b; Šablevičius 2000b; Gliaudys 2001; Tamutis and Zolubas 2001; Danilevsky 2003; Ferenca 2006b; Alekseev 2007; Sama 2009; Sama and Löbl 2010.

*mordax* **(DeGeer, 1775)**. Eichwald 1830; Zawadzki 1936; Stanilisówna 1939; Pileckis 1960, 1976a; Lešinskas and Pileckis 1967; Pileckis et al. 1968; Gaidienė and Ferenca 1992; Silfverberg 1992, 2004; Gaidienė 1993; Monsevičius 1997; Pileckis and Monsevičius 1997; Žiogas 1997; Althoff and Danilevsky 1997; Šablevičius 2000b, 2011; Gliaudys 2001; Tamutis and Zolubas 2001; Danilevsky 2003; Gedminas 2005; Ferenca 2006b; Alekseev 2007; Gedminas et al. 2007; Sama 2009; Sama and Löbl 2010.

*sycophanta* **(Schranck, 1781)**. Zawadzki 1936; Pileckis 1960, 1970b, 1976a; Silfverberg 1992, 2004; Gaidienė 1993; Pileckis and Monsevičius 1997; Žiogas 1997; Althoff and Danilevsky 1997; Danilevsky 2003; Ferenca 2006b; Alekseev 2007; Sama 2009; Sama and Löbl 2010.

*Stenocorus* **Geoffroy, 1762**.

*meridianus* **(Linnaeus, 1758)**. Heyden 1903; Zawadzki 1936; Pileckis 1960, 1976a; Silfverberg 1992, 2004; Gaidienė 1993; Pileckis and Monsevičius 1997; Althoff and Danilevsky 1997; Šablevičius 2000b; Gliaudys 2001; Danilevsky 2003; Alekseev 2007; Sama 2009; Sama and Löbl 2010.

*Pachyta* **Dejean, 1821**.

*lamed* **(Linnaeus, 1758)**. Pileckis 1976a; Silfverberg 1992, 2004; Pileckis and Monsevičius 1997; Althoff and Danilevsky 1997; Šablevičius 2001, 2003a, 2004, 2011; Danilevsky 2003; Alekseev 2007; Sama 2009; Sama and Löbl 2010.

*quadrimaculata* **(Linnaeus, 1758)**. Zawadzki 1936; Stanilisówna 1939; Pileckis 1960, 1970a,1976 Lešinskas and Pileckis 1967; Silfverberg 1992, 2004; Gaidienė 1993; Monsevičius 1997; Pileckis and Monsevičius 1997; Althoff and Danilevsky 1997; Šablevičius 2000b, 2011; Gliaudys 2001; Tamutis and Zolubas 2001; Danilevsky 2003; Ferenca 2006b; Alekseev 2007; Vaivilavičius 2008; Sama 2009; Sama and Löbl 2010.

*Brachyta* **Fairmaire, 1864**.

*interrogationis* **(Linnaeus, 1758)**. Pileckis 1960, 1970a, 1976a; Silfverberg 1992, 2004; Pileckis and Monsevičius 1997 (*Evodinus*); Althoff and Danilevsky 1997; Danilevsky 2003; Alekseev 2007; Sama 2009; Sama and Löbl 2010.

*Evodinus* **LeConte, 1850** = *Evodinellus* Plavilstshikov, 1915.

*borealis* **(Gyllenhal, 1827)**. Pileckis and Monsevičius 1997; Althoff and Danilevsky 1997; Danilevsky 2003; Ferenca 2003; Silfverberg 2004; Alekseev 2007; Sama 2009.

*Acmaeops* **LeConte, 1850**.

[*angusticollis* **(Gebler, 1833)**]. Known in northern Belarus (Alexandrovitch et al. 1996), Poland (Burakowski et al. 1989).

*marginata* **(Fabricius, 1781)**. Stanilisówna 1939; Pileckis 1960, 1976a; Silfverberg 1992, 2004; Pileckis and Monsevičius 1997; Althoff and Danilevsky 1997; Danilevsky 2003; Alekseev 2007; Sama 2009; Sama and Löbl 2010.

*septentrionis* **(Thomson, 1866)**.Althoff and Danilevsky 1997; Danilevsky 2003; Sama 2009; Sama and Löbl 2010.

*smaragdulus* **(Fabricius, 1792)**. Zawadzki 1936; Pileckis 1960, 1976a; Silfverberg 1992, 2004; Pileckis and Monsevičius 1997; Althoff and Danilevsky 1997; Danilevsky 2003; Alekseev 2007; Sama 2009.

*Gnathacmaeops* **Linsley & Chemsak, 1972**.

*pratensis* **(Laicharting, 1784)**. Eichwald 1830; Zawadzki 1936; Pileckis 1960, 1976a; Silfverberg 1992, 2004; Pileckis and Monsevičius 1997 (*Acmaeops*); Althoff and Danilevsky 1997; Danilevsky 2003; Alekseev 2007; Sama 2009; Sama and Löbl 2010.

*Dinoptera* **Mulsant, 1863**.

*collaris* **(Linnaeus, 1758)**. Eichwald 1830; Zawadzki 1936; Stanilisówna 1939; Pileckis 1960, 1963b, 1970a, 1976a; Zajančkauskas and Pileckis 1968; Silfverberg 1992, 2004; Gaidienė 1993; Monsevičius 1997; Pileckis and Monsevičius 1997 (*Acmaeops*); Althoff and Danilevsky 1997; Šablevičius 2000b; Gliaudys 2001; Danilevsky 2003; Ferenca 2006b; Alekseev 2007; Vaivilavičius 2008; Sama 2009; Sama and Löbl 2010.

*Gaurotes* **LeConte, 1850** = *Carilia* Mulsant, 1863.

*virginea* **(Linnaeus, 1758)**. Zawadzki 1936; Pileckis 1960, 1976a; Silfverberg 1992, 2004; Gaidienė 1993; Monsevičius 1997; Pileckis and Monsevičius 1997; Althoff and Danilevsky 1997; Šablevičius 2000b; Gliaudys 2001; Danilevsky 2003; Alekseev 2007; Sama 2009; Sama and Löbl 2010.

*Pidonia* **Mulsant, 1863**.

*lurida* **(Fabricius, 1787)**. Althoff and Danilevsky 1997; Danilevsky 2003; Tamutis 2003; Alekseev 2007; Sama 2009; Inokaitis 2009; Sama and Löbl 2010.

**Lepturini Latreille, 1802**.

*Cortodera* **Mulsant, 1863**.

*femorata* **(Fabricius, 1787)**. Pileckis 1959, 1960, 1976a, b, 1979; Silfverberg 1992, 2004; Pileckis and Monsevičius 1997; Althoff and Danilevsky 1997; Ostrauskas 2000; Šablevičius 2001, 2003a, 2004; Danilevsky 2003; Ferenca 2004; Inokaitis 2004, 2009; Butvila et al. 2007; Alekseev 2007; Sama 2009; Sama and Löbl 2010.

*Grammoptera* **Audinet-Serville, 1835**.

[*abdominalis* **(Stephens, 1831)**]. Known in Denmark, Sweden (Silfverberg 2004), Belarus (Alexandrovitch et al. 1996), northern Poland (Burakowski et al. 1989), Kaliningrad region (Alekseev 2007). 

*ruficornis* **(Fabricius, 1781)**. Zawadzki 1936; Pileckis 1959, 1960, 1970b, 1976a; Silfverberg 1992, 2004; Gaidienė 1993; Pileckis and Monsevičius 1997; Althoff and Danilevsky 1997; Danilevsky 2003; Šablevičius 2004, 2003a, 2007; Alekseev 2007; Sama 2009; Sama and Löbl 2010.

[*ustulata* **(Schaller, 1783)**]. Known in Denmark, southern Sweden (Lundberg and Gustafsson 1995), Belarus (Alexandrovitch et al. 1996), Poland (Burakowski et al. 1989).

*Alosterna* **Mulsant, 1863**.

*ingrica* **(Baeckmann, 1902)**. Pileckis 1976a; Silfverberg 1992, 2004; Pileckis and Monsevičius 1997 (*Grammoptera erythropus ingrica*); Althoff and Danilevsky 1997; Danilevsky 2003; Alekseev 2007; Sama 2009; Sama and Löbl 2010.

*tabacicolor* **(DeGeer, 1775)**. Zawadzki 1936; Stanilisówna 1939; Pileckis 1960, 1963b, 1976a; Silfverberg 1992, 2004; Gaidienė 1993; Monsevičius 1997; Pileckis and Monsevičius 1997; Althoff and Danilevsky 1997; Danilevsky 2003; Alekseev 2007; Sama 2009; Sama and Löbl 2010.

*Nivellia* **Mulsant, 1863**.

[*sanguinosa* **(Gyllenhal, 1827)**]. Known in Sweden (Silfverberg 2004), northwestern Belarus (Alexandrovitch et al. 1996), northeastern Poland (Burakowski et al. 1989), Kaliningrad region (Alekseev 2007).

*Pseudovadonia* **Lobanov, Danilevsky & Murzin, 1981**.

*livida* **(Fabricius, 1777)**. Roubal 1910; Zawadzki 1936; Stanilisówna 1939; Mazurowa and Mazur 1939; Pileckis 1960, 1976a; Silfverberg 1992, 2004; Gaidienė 1993; Monsevičius 1997; Pileckis and Monsevičius 1997 (*Anoplodera*); Ferenca 2006b; Alekseev 2007; Sama 2009; Sama and Löbl 2010.

*Anoplodera* **Mulsant, 1839** = *Corymbia* Gozis, 1886.

*rufipes* **(Schaller, 1783)**. Althoff and Danilevsky 1997; Danilevsky 2003; Ferenca 2003; Ferenca et al. 2006, 2007; Alekseev 2007; Sama 2009; Inokaitis 2009; Sama and Löbl 2010.

*sexguttata* **(Fabricius, 1775)**. Zawadzki 1936; Stanilisówna 1939; Pileckis 1959, 1960, 1976a; Silfverberg 1992, 2004; Pileckis and Monsevičius 1997; Althoff and Danilevsky 1997; Danilevsky 2003; Inokaitis 2004; Alekseev, 2007 Sama 2009; Ivinskis et al. 2009; Sama and Löbl 2010.

*Lepturobosca* **Reitter, 1912**.

*virens* **(Linnaeus, 1758)**. Zawadzki 1936; Pileckis 1960, 1970a, 1976a; Silfverberg 1992, 2004; Pileckis and Monsevičius 1997 (*Anoplodera*); Gliaudys 2001; Alekseev 2007; Sama 2009; Sama and Löbl 2010; Šablevičius 2011.

*Corymbia* **Gosis, 1886**= *Stictoleptura* Casey, 1924.

*rubra* **(Linnaeus, 1758)**. Heyden 1903; Ivanauskas and Vailionis 1922; Zawadzki 1936; Stanilisówna 1939; Mazurowa and Mazur 1939; Pileckis 1960, 1976a; Lešinskas and Pileckis 1967; Pileckis and Zajančkauskas 1968; Jakaitis and Valenta 1976; Silfverberg 1992, 2004; Gaidienė 1993; Monsevičius 1997; Pileckis and Monsevičius 1997 (*Anoplodera*); Althoff and Danilevsky 1997; Šablevičius 2000b; Gliaudys 2001; Danilevsky 2003; Ferenca 2006b; Alekseev 2007; Vaivilavičius 2008; Sama 2009; Sama and Löbl 2010.

*variicornis* **(Dalman, 1817)**. Pileckis 1976a; Pileckis and Monsevičius 1997 (*Anoplodera*); Althoff and Danilevsky 1997; Danilevsky 2003; Silfverberg 1992, 2004; Alekseev 2007; Sama 2009.

*Paracorymbia* **Miroshnikov, 1998**.

*fulva **(DeGeer, 1775)**. # 68. Gaidienė 1993; Silfverberg 2004.

*maculicornis* **(DeGeer, 1775)**. Heyden 1903; Zawadzki 1936; Stanilisówna 1939; Pileckis 1960, 1976a; Silfverberg 1992, 2004; Gaidienė 1993; Monsevičius 1997; Pileckis and Monsevičius 1997 (*Anoplodera*); Althoff and Danilevsky 1997; Šablevičius 2000b; 1992, 2004; Danilevsky 2003; Ferenca 2006b; Alekseev 2007; Vaivilavičius 2008; Sama 2009; Sama and Löbl 2010.

[*scutellata* **(Fabricius, 1781)**]. Known in Latvia (Telnov 2004), Denmark, southern Sweden (Lundberg and Gustafsson 1995), northwestern Belarus (Alexandrovitch et al. 1996), northeastern Poland (Burakowski et al. 1989).

*Anastrangalia* **Casey, 1924**.

*dubia* **(Scopoli, 1763)**. # 69.Pileckis 1960, 1976a; Zajančkauskas and Pileckis 1968; Silfverberg 1992, 2004; Gaidienė 1993; Monsevičius 1997; Pileckis and Monsevičius 1997 (*Anoplodera*); Althoff and Danilevsky 1997; Tamutis and Zolubas 2001; Danilevsky 2003; Ferenca 2006b; Sama 2009; Sama and Löbl 2010.

*reyi* **(Heyden, 1889)** = *inexpectata* (Jansson and Sjöberg, 1928). # 69. Zawadzki 1936; Stanilisówna 1939; Pileckis 1960, 1976a; Silfverberg 1992, 2004; Gaidienė 1993; Monsevičius 1997; Pileckis and Monsevičius 1997 (*Anoplodera*); Althoff and Danilevsky 1997; Danilevsky 2003; Alekseev 2007; Vaivilavičius 2008; Sama 2009; Sama and Löbl 2010.

*sanguinolenta* **(Linnaeus, 1761)**. Zawadzki 1936; Stanilisówna 1939; Pileckis 1960, 1976a; Silfverberg 1992, 2004; Monsevičius 1997; Pileckis and Monsevičius 1997 (*Anoplodera*); Althoff and Danilevsky 1997; Danilevsky 2003; Ferenca 2006b; Alekseev 2007; Vaivilavičius 2008; Sama 2009; Sama and Löbl 2010.

*sequensi **(Reitter, 1898).** Stanilisówna 1939; disproved by Pileckis (1960).

*Judolia* **Mulsant, 1863**.

*sexmaculata* **(Linnaeus, 1758)**. Zawadzki 1936; Pileckis 1960, 1963b, 1976a; Silfverberg 1992, 2004; Gaidienė 1993; Monsevičius 1997; Pileckis and Monsevičius 1997; Althoff and Danilevsky 1997; Danilevsky 2003; Inokaitis 2004, 2009; Alekseev 2007; Sama 2009; Sama and Löbl 2010.

*Pachytodes* Pic, 1891.

*cerambyciformis* **(Schrank, 1781)**. Miländer et al. 1984; Silfverberg 1992, 2004; Gaidienė 1993; Pileckis and Monsevičius 1997 (*Judolia*); Althoff and Danilevsky 1997; Danilevsky 2003; Ferenca 2004; Inokaitis 2004, 2009; Alekseev 2007; Sama 2009; Ivinskis et al. 2009; Sama and Löbl 2010.

[*erraticus* **(Dalman, 1817)**].Known in northwestern Belarus (Alexandrovitch et al. 1996), Poland (Burakowski et al. 1989).

*Macroleptura* **Nakane & Ohbayashi, 1957**.

*thoracica* **(Creutzer, 1799)**. Zawadzki 1936; Pileckis 1960, 1976a; Silfverberg 1992, 2004; Gaidienė 1993; Pileckis and Monsevičius 1997 (*Leptura*); Althoff and Danilevsky 1997; Danilevsky 2003; Ehnström et al. 2003; Ferenca 2006b; Alekseev 2007; Sama 2009; Sama and Löbl 2010.

*Leptura* **Linnaeus, 1758**.

*aethiops* **Poda, 1761**. Zawadzki 1936; Stanilisówna 1939; Pileckis 1960, 1976a; Lešinskas and Pileckis 1967; Silfverberg 1992, 2004; Gaidienė 1993; Monsevičius 1997; Pileckis and Monsevičius 1997; Althoff and Danilevsky 1997; Šablevičius 2000b; Gliaudys 2001; Danilevsky 2003; Ferenca 2006b; Alekseev 2007; Vaivilavičius 2008; Sama 2009; Sama and Löbl 2010.

*annularis* **Fabricius, 1792** = *arcuata* Panzer, 1793, nec Linnaeus, 1758 = *mimica* Bates, 1884. Stanilisówna 1939; Pileckis 1960, 1976a; Silfverberg 1992, 2004; Gaidienė 1993; Althoff and Danilevsky 1997; Pileckis and Monsevičius 1997; Šablevičius 2000b; Ferenca et al. 2002; Danilevsky 2003; Alekseev 2007; Sama 2009; Ivinskis et al. 2009; Sama and Löbl 2010.

*aurulenta **Fabricius, 1792**. # 70. Pileckis 1963b, 1970b, 1976a, b, 1979; Pileckis and Monsevičius 1997; Silfverberg 1992, 2004; Alekseev, 2007; Sama and Löbl 2010.

*quadrifasciata* **Linnaeus, 1758**. Eichwald 1830; Zawadzki 1936; Stanilisówna 1939; Mazurowa and Mazur 1939; Pileckis 1960, 1976a; Lešinskas and Pileckis 1967; Zajančkauskas and Pileckis 1968; Silfverberg 1992, 2004; Gaidienė 1993; Althoff and Danilevsky 1997; Monsevičius 1997; Pileckis and Monsevičius 1997; Šablevičius 2000b, 2011; Gliaudys 2001; Danilevsky 2003; Ferenca 2006b; Alekseev 2007; Sama 2009; Sama and Löbl 2010.

*Lepturalia* **Reitter, 1913**.

*nigripes* **DeGeer, 1775**. Zawadzki 1936; Pileckis 1960, 1976a; Silfverberg 1992, 2004; Monsevičius 1997; Pileckis and Monsevičius 1997 (*Leptura*); Althoff and Danilevsky 1997; Danilevsky 2003; Alekseev 2007; Sama 2009; Sama and Löbl 2010.

*Rutpela* **Nakane & Ohbayashi, 1957**.

*maculata* **(Poda, 1761)**. Pileckis 1976a; Silfverberg 1992, 2004; Pileckis and Monsevičius 1997 (*Leptura*); Althoff and Danilevsky 1997; Danilevsky 2003; Alekseev 2007; Sama 2009; Sama and Löbl 2010.

*Stenurella* **Villiers, 1974**.

*bifasciata* **(O.F. Müller, 1776)**. Zawadzki 1936; Stanilisówna 1939; Pileckis 1960, 1963b, 1976a; Silfverberg 1992, 2004; Gaidienė 1993; Pileckis and Monsevičius 1997 (*Leptura*); Althoff and Danilevsky 1997; Danilevsky 2003; Alekseev 2007; Vaivilavičius 2008; Sama 2009; Ostrauskas and Ferenca 2010; Sama and Löbl 2010.

*melanura* **(Linnaeus, 1758)**. Heyden 1903; Zawadzki 1936; Stanilisówna 1939; Mazurowa and Mazur 1939; Pileckis 1960, 1976a; Zajančkauskas and Pileckis 1968; Silfverberg 1992, 2004; Gaidienė 1993; Althoff and Danilevsky 1997; Monsevičius 1997; Pileckis and Monsevičius 1997 (*Leptura*); Šablevičius 2000b; Gliaudys 2001; Danilevsky 2003; Ferenca 2006b; Alekseev 2007; Vaivilavičius 2008; Sama 2009; Ostrauskas and Ferenca 2010; Sama and Löbl 2010.

*nigra* **(Linnaeus, 1758)**. Pileckis 1962, 1963b, 1976a; Silfverberg 1992, 2004; Gaidienė 1993; Monsevičius 1997; Pileckis and Monsevičius 1997 (*Leptura*); Šablevičius 2000b; Althoff and Danilevsky 1997; Danilevsky 2003; Inokaitis 2004; Alekseev 2007; Sama 2009; Sama and Löbl 2010.

*Pedostrangalia* **Sokolov, 1896**.

*pubescens* **(Fabricius, 1787)**. Zawadzki 1936; Pileckis 1960, 1976a; Silfverberg 1992, 2004; Monsevičius 1997; Pileckis and Monsevičius 1997 (*Leptura*); Althoff and Danilevsky 1997; Danilevsky 2003; Ferenca 2003; Alekseev 2007; Sama 2009; Sama and Löbl 2010.

[*revestita* **(Linnaeus, 1767)**]. Known in Denmark, southern Sweden (Lundberg and Gustafsson 1995), northern Poland (Burakowski et al. 1989).

*Strangalia* **Audinet-Serville, 1835**.

*attenuata* **(Linnaeus, 1758)**. Eichwald 1830; Zawadzki 1936; Pileckis 1960, 1976a; Zajančkauskas and Pileckis 1968; Silfverberg 1992, 2004; Gaidienė 1993; Monsevičius 1997; Althoff and Danilevsky 1997; Pileckis and Monsevičius 1997; Šablevičius 2000b; Gliaudys 2001; Danilevsky 2003; Ferenca 2006b; Alekseev 2007; Sama 2009; Sama and Löbl 2010.

**Spondylidinae** **Audinet-Serville, 1832**.

**Asemini Thomson, 1861**.

*Arhopalus* **Audinet-Serville, 1834** = *Criocephalus* Dejean, 1835.

*rusticus* **(Linnaeus, 1758)**. Eichwald 1830; Roubal 1910; Zawadzki 1936; Stanilisówna 1939; Mazurowa and Mazur 1939; Pileckis 1960, 1976a; Lešinskas and Pileckis 1967; Pileckis . 1968; Zajančkauskas and Pileckis 1968; Jakaitis and Valenta 1976; Silfverberg 1992, 2004; Gaidienė 1993; Monsevičius 1997; Pileckis and Monsevičius 1997; Althoff and Danilevsky 1997; Valenta 2000b; Gliaudys 2001; Danilevsky 2003; Ivinskis et al. 2004b; Ferenca 2006b; Alekseev 2007; Sama 2009; Sama and Löbl 2010.

*tristis* **(Fabricius, 1787)** = *ferus* (Mulsant, 1839). Heyden 1903; Zawadzki 1936; Stanilisówna 1939; Pileckis 1960, 1963b,1976a; Silfverberg 1992, 2004; Gaidienė 1993; Pileckis and Monsevičius 1997; Althoff and Danilevsky 1997; Danilevsky 2003; Ehnström et al. 2003; Ferenca 2006b; Alekseev 2007; Sama 2009; Sama and Löbl 2010.

*Asemum* **Eschscholtz, 1830**.

*striatum* **(Linnaeus, 1758)**. Zawadzki 1936; Stanilisówna 1939; Pileckis 1960, 1976a; Lešinskas and Pileckis 1967; Jakaitis and Valenta 1976; Silfverberg 1992, 2004; Gaidienė 1993; Monsevičius 1997; Pileckis and Monsevičius 1997; Althoff and Danilevsky 1997; Gliaudys 2001; Tamutis and Zolubas 2001; Danilevsky 2003; Alekseev 2007, 2008a; Sama 2009; Sama and Löbl 2010.

[*tenuicorne* **Kraatz, 1879**]. Known in southern Sweden (Lundberg and Gustafsson 1995).

*Tetropium* **Kirby, 1837**.

*castaneum* **(Linnaeus, 1758)**. Stanilisówna 1939; Pileckis 1960, 1976a; Valenta 1965b, 2000b; Lešinskas and Pileckis 1967; Pileckis et al. 1968; Zajančkauskas and Pileckis 1968; Gavelis and Žiogas; 1991; Silfverberg 1992, 2004; Gaidienė 1993; Monsevičius 1997; Pileckis and Monsevičius 1997; Žiogas 1997; Althoff and Danilevsky 1997; Šablevičius 2000b; Tamutis and Zolubas 2001; Danilevsky 2003; Ferenca 2006b; Alekseev 2007; Sama 2009; Sama and Löbl 2010.

*fuscum* **(Fabricius, 1787)**. Zawadzki 1936; Stanilisówna 1939; Pileckis 1960, 1963b, 1976a; Silfverberg 1992, 2004; Gaidienė 1993; Monsevičius 1997; Pileckis and Monsevičius 1997; Althoff and Danilevsky 1997; Tamutis and Zolubas 2001; Danilevsky 2003; Alekseev 2007; Sama 2009; Ivinskis et al. 2009; Sama and Löbl 2010.

[*gabrieli* **Weise, 1905**]. Known in Denmark, northern Poland (Burakowski et al. 1989).

*Nothorhina* **Redtenbacher, 1845**.

*muricata* **(Dalman, 1817)** = *punctata* (Fabricius, 1798). Pileckis 1976a; Silfverberg 1992, 2004; Pileckis and Monsevičius 1997; Althoff and Danilevsky 1997; Danilevsky 2003; Ehnström et al. 2003; Alekseev 2007; Sama 2009; Sama and Löbl 2010.

**Spondylidini Audinet-Serville, 1832**.

*Spondylis* **Fabricius, 1775**.

*buprestoides* **(Linnaeus, 1758)**. Eichwald 1830; Zawadzki 1936; Stanilisówna 1939; Mazurowa and Mazur 1939; Pileckis 1960, 1976a; Lešinskas and Pileckis 1967; Pileckis et al. 1968; Jakaitis and Valenta 1976; Silfverberg 1992, 2004; Gaidienė 1993; Monsevičius 1997; Pileckis and Monsevičius 1997; Althoff and Danilevsky 1997; Žiogas 1997; Valenta 2000b; Šablevičius 2000b, 2011; Gliaudys 2001; Danilevsky 2003; Ferenca 2006b; Alekseev 2007; Sama 2009; Sama and Löbl 2010.

**Necydalinae** **Latreille, 1825**.

*Necydalis* **Linnaeus, 1758**.

RDB*major* **Linnaeus, 1758**. Pileckis 1960, 1976a; Lešinskas and Pileckis 1967; Silfverberg 1992, 2004; Gaidienė 1993; Pileckis and Monsevičius 1997; Althoff and Danilevsky 1997; Gliaudys 2001; Danilevsky 2003; Ehnström et al. 2003; Ferenca 2004; Rašomavičius 2007; Uselis et al. 2007; Alekseev 2007, 2010b; Sruoga 2008; Sama 2009; Ivinskis et al. 2009; Inokaitis 2009; Sama and Löbl 2010.

*ulmi* **Chevrolat, 1836**. Silfverberg 1992, 2004; Althoff and Danilevsky 1997; Danilevsky 2003; Sama 2009; Sama and Löbl 2010.

**Cerambycinae** **Latreille, 1802**.

**Graciliini Mulsant, 1839**.

*Gracilia* **Audinet-Serville, 1834**.

*minuta* **(Fabricius, 1781)**.Stanilisówna 1939; Pileckis 1959, 1960, 1970a, 1976a; Pileckis et al. 1968; Silfverberg 1992, 2004; Pileckis and Monsevičius 1997; Althoff and Danilevsky 1997; Danilevsky 2003; Alekseev, 2007; Sama and Löbl 2010.

*Axinopalpis* **Duponchel & Chevrolat, 1842**.

*gracilis* **(Krynicki, 1832)**. Pileckis 1960, 1970b, 1976a; Silfverberg 1992, 2004; Pileckis and Monsevičius 1997; Althoff and Danilevsky 1997; Danilevsky 2003; Ferenca 2006b; Alekseev 2007; Sama 2009; Sama and Löbl 2010.

**Psebiini Lacordaire, 1868**.

*Nathrius* **Bréthes, 1916**.

[*brevipennis* **(Mulsant, 1839)**]. Imported in Denmark, Sweden (Lundberg and Gustafsson 1995), Poland (Burakowski et al. 1989).

**Molorchini Gistel, 1848**.

*Molorchus* **Fabricius, 1792**.

*minor* **(Linnaeus, 1758)**. Zawadzki 1936; Stanilisówna 1939; Pileckis 1960, 1976a; Lešinskas and Pileckis 1967; Silfverberg 1992, 2004; Gaidienė 1993; Pileckis and Monsevičius 1997; Althoff and Danilevsky 1997; Gliaudys 2001; Tamutis and Zolubas 2001; Danilevsky 2003; Šablevičius 2003a; Ferenca 2006b Alekseev 2007; Sama 2009; Zeniauskas and Gedminas 2010; Sama and Löbl 2010.

*Glaphyra* **Newman, 1840**.

*umbellatarum* **(Schreber, 1759)**. Pileckis 1976a; Silfverberg 1992, 2004; Gaidienė 1993; Pileckis and Monsevičius 1997; Althoff and Danilevsky 1997; Danilevsky 2003; Inokaitis 2004; Alekseev 2007; Sama 2009; Sama and Löbl 2010.

**Obriini Mulsant, 1839**.

*Obrium* **Dejean, 1821**.

*brunneum* **(Fabricius, 1792)**. Althoff and Danilevsky 1997; Danilevsky 2003; Šablevičius 2004; Ferenca 2004, 2006; Sama 2009; Ivinskis et al. 2009; Sama and Löbl 2010.

*cantharinum* **(Linnaeus, 1767)**. Pileckis 1960, 1976a; Silfverberg 1992, 2004; Gaidienė 1993; Pileckis and Monsevičius 1997; Althoff and Danilevsky 1997; Gliaudys 2001; Danilevsky 2003; Šablevičius 2003a; Alekseev 2007; Sama 2009; Ivinskis et al. 2009; Sama and Löbl 2010.

**Cerambycini Latreille, 1802**.

*Cerambyx* **Linnaeus, 1758**.

RDB*cerdo* **Linnaeus, 1758**. Pileckis 1959, 1960, 1970a, b, 1976a; Lešinskas and Pileckis 1967; Pileckis et al. 1968; Balevičius 1992; Silfverberg 1992, 2004; Gaidienė 1993; Ivinskis et al. 1996b; Totorienė 1996; Monsevičius 1997; Pileckis and Monsevičius 1997; Althoff and Danilevsky 1997; Danilevsky 2003; Ferenca 2006b; Rašomavičius 2007; Alekseev 2007, 2010b.

*scopoli* **Fuessil, 1775**. Pileckis 1960, 1970a, b, 1976a; Silfverberg 1992, 2004; Pileckis and Monsevičius 1997; Althoff and Danilevsky 1997; Danilevsky 2003; Alekseev 2007; Sama 2009; Sama and Löbl 2010.

**Trachyderini Dupont, 1836**.

*Purpuricenus* **Dejean, 1821**.

[*kaehleri* **(Linnaeus, 1758)**]. Known in eastern Belarus (Alexandrovitch et al. 1996), Poland (Burakowski et al. 1989).

**Callichromatini Swainson, 1840**.

*Aromia* **Audinet-Serville, 1833**.

*moschata* **(Linnaeus, 1758)**. Ivanauskas and Vailionis 1922; Mastauskis 1925; Zawadzki 1936; Stanilisówna 1939; Pileckis 1960, 1976a; Lešinskas and Pileckis 1967; Zajančkauskas and Pileckis 1968; Gaidienė and Ferenca 1992; Silfverberg 1992, 2004; Gaidienė 1993; Monsevičius 1997; Pileckis and Monsevičius 1997; Althoff and Danilevsky 1997; Šablevičius 2000b, 2011; Gliaudys 2001; Danilevsky 2003; Ferenca 2006b; Alekseev 2007; Sama 2009; Sama and Löbl 2010.

**Hylotrupini Zagaikievich, 1991**.

*Hylotrupes* **Audinet-Serville, 1834**.

*bajulus* **(Linnaeus, 1758)**. Eichwald 1830; Zawadzki 1936; Stanilisówna 1939; Pileckis 1960, 1970a, 1976a; Lešinskas and Pileckis 1967; Silfverberg 1992, 2004; Gaidienė 1993; Monsevičius 1997; Pileckis and Monsevičius 1997; Žiogas 1997, 2000b; Althoff and Danilevsky 1997; Gliaudys 2001; Danilevsky 2003; Ferenca 2006b; Alekseev 2007; Sama 2009; Inokaitis 2009; Sama and Löbl 2010.

**Callidiini Kirby, 1837**.

*Leioderes* **Redtenbacher, 1849**.

*kollari* **Redtenbacher, 1849**. Pileckis 1968b, 1970a, b, 1976a; Silfverberg 1992, 2004; Pileckis and Monsevičius 1997; Althoff and Danilevsky 1997; Danilevsky 2003; Alekseev 2007; Sama 2009; Ivinskis et al. 2009; Sama and Löbl 2010.

*Semanotus* **Mulsant, 1839**.

*undatus* **(Linnaeus, 1758)**. Pileckis 1970a, 1976a; Silfverberg 1992, 2004; Pileckis and Monsevičius 1997; Althoff and Danilevsky 1997; Danilevsky 2003; Alekseev 2007; Sama 2009; Sama and Löbl 2010.

*Ropalopus* **Mulsant, 1839**.

*clavipes* **(Fabricius, 1775)**. Stanilisówna 1939; Pileckis 1960, 1976a; Silfverberg 1992, 2004; Gaidienė 1993; Pileckis and Monsevičius 1997; Althoff and Danilevsky 1997; Gliaudys 2001; Danilevsky 2003; Ferenca 2006b; Alekseev 2007; Sama 2009; Sama and Löbl 2010.

*femoratus* **(Linnaeus, 1758)**. Althoff and Danilevsky 1997; Danilevsky 2003.

*macropus* **(Germar, 1824)**. Pileckis et al. 1968; Gaidienė 1993; Tamutis 2003; Silfverberg 2004; Inokaitis 2004, 2009; Alekseev 2007.

*Callidium* **Fabricius, 1775**.

*aeneum* **(DeGeer, 1775)**. Zawadzki 1936; Pileckis 1960, 1976a; Silfverberg 1992, 2004; Pileckis and Monsevičius 1997; Althoff and Danilevsky 1997; Danilevsky 2003; Alekseev 2007; Sama 2009; Inokaitis 2009; Zeniauskas and Gedminas 2010; Sama and Löbl 2010.

*coriaceum* **Paykull, 1800**. Gaidienė 1993; Pileckis and Monsevičius 1997; Althoff and Danilevsky 1997; Danilevsky 2003; Ehnström et al. 2003; Silfverberg 2004; Inokaitis 2004; Masiulis 2007; Alekseev, 2007; Sama 2009; Ivinskis et al. 2009; Sama and Löbl 2010.

*violaceum* **(Linnaeus, 1758)**. Eichwald 1830; Zawadzki 1936; Stanilisówna 1939; Pileckis 1960, 1976a; Lešinskas and Pileckis 1967; Silfverberg 1992, 2004; Gaidienė 1993; Monsevičius 1997; Pileckis and Monsevičius 1997; Althoff and Danilevsky 1997; Žiogas 2000b; Šablevičius 2000b; Gliaudys 2001; Danilevsky 2003; Ferenca 2006b; Alekseev 2007; Sama 2009; Sama and Löbl 2010.

*Pyrrhidium* **Fairmaire, 1864**.

*sanguineum* **(Linnaeus, 1758)**. Zawadzki 1936; Pileckis 1960, 1976a; Silfverberg 1992, 2004; Pileckis and Monsevičius 1997; Althoff and Danilevsky 1997; Danilevsky 2003; Alekseev 2007; Sama 2009; Inokaitis 2009; Sama and Löbl 2010.

*Phymatodes* **Mulsant, 1839**.

*testaceus* **(Linnaeus, 1758)**. Stanilisówna 1939; Pileckis 1960, 1976a; Pileckis et al. 1968; Silfverberg 1992, 2004; Gaidienė 1993; Monsevičius 1997; Pileckis and Monsevičius 1997; Althoff and Danilevsky 1997; Danilevsky 2003; Ferenca 2006b; Alekseev 2007; Sama 2009; Sama and Löbl 2010; Šablevičius 2011.

*Poecilium* **Fairmaire, 1864**.

*alni* **(Linnaeus, 1767)**. Althoff and Danilevsky 1997; Danilevsky 2003; Silfverberg 2004; Gedminas 2005; Ferenca et al. 2006, 2007; Gedminas et al. 2007; Alekseev 2007; Sama 2009; Inokaitis 2009; Sama and Löbl 2010.

[*pusillum* **(Fabricius, 1787)**]. Known in southern Sweden (Lundberg and Gustafsson 1995), Poland (Burakowski et al. 1989).

**Anaglyptini Lacordaire, 1868**.

*Anaglyptus* **Mulsant, 1839**.

*mysticus* **(Linnaeus, 1758)**. Althoff and Danilevsky 1997; Danilevsky 2003; Silfverberg 2004; Alekseev 2007; Sama 2009; Sama and Löbl 2010.

**Clytini Mulsant, 1839**.

*Xylotrechus* **Chevrolat, 1860**.

*antilope* **(Schönherr, 1817)**.Ferenca and Tamutis 2009.

*arvicola* **(Olivier, 1758)**. Zawadzki 1936; Pileckis 1959, 1960, 1970a, 1976a; Silfverberg 1992, 2004; Pileckis and Monsevičius 1997; Althoff and Danilevsky 1997; Danilevsky 2003; Ehnström et al. 2003; Alekseev 2007; Sama 2009; Sama and Löbl 2010.

*ibex* **(Gebler, 1825)**. Monsevičius 1988b; Silfverberg 1992, 2004; Pileckis and Monsevičius 1997; Alekseev 2007.

*Rusticoclytus* **Vives, 1977**.

*pantherinus* **(Savenius, 1825)**. Inokaitis 2004 (*Xylotrechus*).

*rusticus* **(Linnaeus, 1758)**. Zawadzki 1936; Stanilisówna 1939; Pileckis 1960, 1963b, 1976a; Pileckis et al. 1968; Silfverberg 1992, 2004; Gaidienė 1993; Pileckis and Monsevičius 1997 (*Xylotrechus*); Althoff and Danilevsky 1997; Šablevičius 2000b; Gliaudys 2001; Tamutis and Zolubas 2001; Danilevsky 2003; Ehnström et al. 2003; Ferenca 2006b; Alekseev 2007; Sama 2009; Sama and Löbl 2010.

*Plagionotus* **Mulsant, 1842**.

*arcuatus* **(Linnaeus, 1758)**. Stanilisówna 1939; Pileckis 1960, 1976a; Pileckis et al. 1968; Silfverberg 1992, 2004; Gaidienė 1993; Pileckis and Monsevičius 1997; Althoff and Danilevsky 1997; Šablevičius 2000b, 2011; Gliaudys 2001; Danilevsky 2003; Inokaitis 2004; Ferenca 2006b; Alekseev 2007; Sama 2009; Sama and Löbl 2010.

*detritus* **(Linnaeus, 1758)**. Stanilisówna 1939; Pileckis 1960, 1976a; Silfverberg 1992, 2004; Pileckis and Monsevičius 1997; Šablevičius 2000b; Gliaudys 2001; Althoff and Danilevsky 1997; Danilevsky 2003; Šablevičius 2003a; Ferenca 2004; Inokaitis 2004; Alekseev 2007; Vaivilavičius 2008; Sama 2009; Ivinskis et al. 2009; Sama and Löbl 2010.

*Echinocerus* **Mulsant, 1863**.

*floralis* **(Pallas, 1773)**. Althoff and Danilevsky 1997; Danilevsky 2003; Silfverberg 2004; Sama 2009; Sama and Löbl 2010.

*Isotomus* **Mulsant, 1863**.

*comptus **(Mannerheim, 1825)**. # 71. Gaidienė 1993.

*speciosus **(Schneider, 1787)**. # 72.Pileckis 1959; Althoff and Danilevsky 1997; Danilevsky 2003; Sama 2009; disproved by Pileckis (1960).

*Chlorophorus* **Chevrolat, 1863**.

*figuratus* **(Scopoli, 1763)**. Althoff and Danilevsky 1997; Danilevsky 2003; Silfverberg 2004; Sama 2009; Sama and Löbl 2010.

*herbstii* **(Brahm, 1790)**. Stanilisówna 1939; Pileckis 1960, 1976a; Silfverberg 1992, 2004; Gaidienė 1993; Pileckis and Monsevičius 1997; Althoff and Danilevsky 1997; Danilevsky 2003; Butvila et al. 2007; Alekseev 2007; Sama 2009; Sama and Löbl 2010.

[*sartor* **(O.F. Müller, 1766)**].Known in northern Poland (Burakowski et al. 1989), northern Belarus (Alexandrovitch et al. 1996).

*varius* **(O.F. Müller, 1766)**. Pileckis and Jakaitis 1989; Silfverberg 1992, 2004; Pileckis and Monsevičius 1997; Althoff and Danilevsky 1997; Danilevsky 2003; Alekseev 2007; Sama 2009; Sama and Löbl 2010.

*Rhaphuma* **Pascoe, 1858**.

[*gracilipes* **(Faldermann, 1835)**]. Known in northwestern Belarus (Alexandrovitch et al. 1996), Poland (Sama 2009).

*Cyrtoclytus* **Ganglbauer, 1881**.

*capra* **(Germar, 1824)**. Pileckis 1960, 1976a; Silfverberg 1992, 2004; Gaidienė 1993; Pileckis and Monsevičius 1997; Althoff and Danilevsky 1997; Danilevsky 2003; Ehnström et al. 2003; Alekseev 2007; Sama 2009; Ivinskis et al. 2009; Sama and Löbl 2010.

*Clytus* **Laicharting, 1784**.

*arietis* **(Linnaeus, 1758)**. Pileckis 1960; 1976a; Silfverberg 1992, 2004; Gaidienė 1993; Pileckis and Monsevičius 1997; Althoff and Danilevsky 1997; Gliaudys 2001; Tamutis and Zolubas 2001; Danilevsky 2003; Ferenca 2004; Inokaitis 2004; Butvila et al. 2007; Alekseev 2007; Sama 2009; Ivinskis et al. 2009; Sama and Löbl 2010.

[*lama* **Mulsant, 1847**]. Known in northwestern Belarus (Alexandrovitch et al. 1996), Poland (Burakowski et al. 1989). Expected in Lithuania.

[*rhamni **(Germar, 1817)**]. # 73. Pileckis 1959; Silfverberg 2004; Alekseev 2007; Sama 2009; disproved by Pileckis (1960).

[*tropicus* **(Panzer, 1795)**]. Known in northwestern Belarus (Alexandrovitch et al. 1996), Poland (Burakowski et al. 1989).

**Lamiinae** **Latreille, 1825**.

**Mesosini Mulsant, 1839**.

*Mesosa* **Latreille, 1829** = *Aplocnemia* Stephens, 1831.

*curculionoides* **(Linnaeus, 1761)**. Pileckis 1959, 1960, 1976a; Silfverberg 1992, 2004; Pileckis and Monsevičius 1997; Althoff and Danilevsky 1997; Danilevsky 2003; Ferenca 2004, Alekseev 2007; Sama 2009; Sama and Löbl 2010; misprint in (Tamutis and Ferenca 2006), this data belongs to *Mesosa myops*.

*myops* **(Dalman, 1817)**. Althoff and Danilevsky 1997; Danilevsky 2003; Tamutis and Ferenca 2006; Ferenca et al. 2006, 2007.

[*nebulosa* **(Fabricius, 1781)**]. Known in Denmark, southern Sweden (Lundberg and Gustafsson 1995), northern Poland (Burakowski et al. 1989), Belarus (Alexandrovitch et al. 1996).

**Monochamini Gistel, 1848**.

*Monochamus* **Dejean, 1821**.

*galloprovincialis* (**Olivier, 1795)**. Zawadzki 1936; Stanilisówna 1939; Mazurowa and Mazur 1939; Pileckis 1960, 1976a; Valenta 1965b, 1977, 2000b; Lešinskas and Pileckis 1967; Pileckis et al. 1968; Ivinskis et al. 1984; Silfverberg 1992, 2004; Gaidienė 1993; Monsevičius 1997; Pileckis and Monsevičius 1997; Žiogas 1997; Gliaudys 2001; Tamutis and Zolubas 2001; Althoff and Danilevsky 1997; Danilevsky 2003; Gedminas and Lynikienė 2005; Ferenca 2006b; Alekseev 2007; Sama 2009; Sama and Löbl 2010; Šablevičius 2011.

*rosenmuelleri* **(Cederhjelm, 1798)** = *urussovi* (Fischer von Waldheim, 1806). Zawadzki 1936; Stanilisówna 1939; Pileckis 1960, 1976a; Lešinskas and Pileckis 1967; Silfverberg 1992, 2004; Gaidienė 1993; Pileckis and Monsevičius 1997; Althoff and Danilevsky 1997; Šablevičius 2000b, 2011; Gliaudys 2001; Danilevsky 2003; Alekseev 2007; Sama 2009; Ivinskis et al. 2009; Sama and Löbl 2010.

*saltuarius* **Gebler, 1730**. Pileckis and Jakaitis 1982; Silfverberg 1992, 2004; Gaidienė 1993; Pileckis and Monsevičius 1997; Alekseev 2007.

*sartor* **(Fabricius, 1787)**. Pileckis 1959, 1960, 1976a; Silfverberg 1992, 2004; Pileckis and Monsevičius 1997; Althoff and Danilevsky 1997; Danilevsky 2003; Ferenca 2006b; Alekseev, 2007; Sama and Löbl 2010.

*sutor* **(Linnaeus, 1758)**. Zawadzki 1936; Stanilisówna 1939; Pileckis 1960, 1976a; Valenta 1965b, 2000b; Lešinskas and Pileckis 1967; Pileckis et al. 1968; Gavelis and Žiogas; 1991; Silfverberg 1992, 2004; Gaidienė 1993; Monsevičius 1997; Pileckis and Monsevičius 1997; Žiogas 1997; Althoff and Danilevsky 1997; Šablevičius 2000b, 2011; Gliaudys 2001; Danilevsky 2003; Ferenca 2006b; Alekseev 2007; Sama 2009; Sama and Löbl 2010.

**Lamiini Latreille, 1825**.

*Lamia* **Fabricius, 1775**.

*textor* **(Linnaeus, 1758)**. Eichwald 1830; Zawadzki 1936; Stanilisówna 1939; Pileckis 1960, 1976a; Lešinskas and Pileckis 1967; Pileckis et al. 1968; Zajančkauskas and Pileckis 1968; Silfverberg 1992, 2004; Gaidienė 1993; Monsevičius 1997; Pileckis and Monsevičius 1997; Althoff and Danilevsky 1997; Šablevičius 2000b, 2011; Gliaudys 2001; Danilevsky 2003; Ferenca 2006b; Alekseev 2007; Sama 2009; Sama and Löbl 2010.

**Dorcadionini Swainson, 1840**.

*Iberodorcadion* **Breuning, 1943**.

[*fuliginator* **(Linnaeus, 1758)**]. Known in Latvia (Telnov 2004).

**Pogonocherini Mulsant, 1839**.

*Pogonocherus* **Dejean, 1821** = *Pityphilus* Mulsant, 1863.

*decoratus* **Fairmaire, 1855**. Zawadzki 1936; Pileckis 1959, 1960, 1976a; Silfverberg 1992, 2004; Gaidienė 1993; Pileckis and Monsevičius 1997; Althoff and Danilevsky 1997; Danilevsky 2003; Ferenca 2006b; Alekseev 2007; Sama 2009; Sama and Löbl 2010.

*fasciculatus* **(DeGeer, 1775)**. Zawadzki 1936; Stanilisówna 1939; Pileckis 1960, 1976a; Pileckis et al. 1968; Zajančkauskas and Pileckis 1968; Valenta and Jakaitis 1972; Silfverberg 1992, 2004; Gaidienė 1993; Monsevičius 1997; Pileckis and Monsevičius 1997; Althoff and Danilevsky 1997; Danilevsky 2003; Alekseev 2007; Sama 2009; Zeniauskas and Gedminas 2010; Sama and Löbl 2010.

*hispidulus* **(Piller and Mitterpacher, 1781)**. Gaidienė 1993; Althoff and Danilevsky 1997; Danilevsky 2003; Silfverberg 2004; Ferenca 2006b; Alekseev 2007; Sama 2009; Sama and Löbl 2010.

*hispidus* **(Linnaeus, 1758)**. Pileckis 1959, 1960, 1976a; Zajančkauskas and Pileckis 1968; Silfverberg 1992, 2004; Gaidienė 1993; Monsevičius 1997; Pileckis and Monsevičius 1997; Tamutis and Zolubas 2001; Althoff and Danilevsky 1997; Danilevsky 2003; Alekseev 2007; Sama 2009; Sama and Löbl 2010.

*ovatus* **(Goeze, 1777)**. Pileckis 1959, 1960, 1976a; Zajančkauskas and Pileckis 1968; Silfverberg 1992, 2004; Gaidienė 1993; Monsevičius 1997; Pileckis and Monsevičius 1997; Althoff and Danilevsky 1997; Danilevsky 2003; Ferenca 2006b; Alekseev 2007; Sama 2009.

**Desmiphorini Thomson, 1860**.

*Anaesthetis* **Dejean, 1835**.

[*testacea* **(Fabricius, 1781)**].Known in Latvia (Telnov 2004), Estonia, southern Sweden (Lundberg and Gustafsson 1995), northern Poland (Burakowski et al. 1989) and northwestern Belarus (Alexandrovitch et al. 1996).

**Acanthoderini Thomson, 1860**.

*Oplosia* **Mulsant, 1863**.

*cinerea* **(Mulsant, 1839)** = *fennica* (Paykull, 1800) nec (Linnaeus, 1758). Pileckis 1976a; Silfverberg 1992, 2004; Pileckis and Monsevičius 1997; Gliaudys 2001; Althoff and Danilevsky 1997; Danilevsky 2003; Ferenca 2004; Inokaitis 2004; Alekseev 2007; Sama 2009; Sama and Löbl 2010.

*Aegomorphus* **Haldeman, 1847** = *Acanthoderes* auct. nec Audinet-Serville, 1835.

*clavipes* **(Schrank, 1781)** = *wojtylai* Hilszczaňski & Bystrowski, 2005.Pileckis 1976a; Silfverberg 1992, 2004; Pileckis and Monsevičius 1997; Althoff and Danilevsky 1997; Gliaudys 2001; Danilevsky 2003; Ehnström et al. 2003; Butvila et al. 2007; Alekseev 2007; Sama 2009; Sama and Löbl 2010.

**Acanthocinini Blanchard, 1845**.

*Acanthocinus* **Dejean, 1821**.

*aedilis* **(Linnaeus, 1758)**. Eichwald 1830; Mastauskis 1925; Zawadzki 1936; Stanilisówna 1939; Mazurowa and Mazur 1939; Pileckis 1960, 1976a; Valenta 1965b, 1977, 2000b; Lešinskas and Pileckis 1967; Pileckis et al. 1968; Zajančkauskas and Pileckis 1968; Valenta and Jakaitis 1972; Jakaitis and Valenta 1976; Ivinskis et al. 1984; Silfverberg 1992, 2004; Gaidienė 1993; Žiogas and Gedminas 1994; Monsevičius 1997; Pileckis and Monsevičius 1997; Althoff and Danilevsky 1997; Žiogas 1997; Šablevičius 2000b, 2011; Gliaudys 2001; Danilevsky 2003; Ehnström et al. 2003; Gedminas and Lynikienė 2005; Ferenca 2006a, b; Alekseev 2007; Sama 2009; Sama and Löbl 2010.

*griseus* **(Fabricius, 1792)**. Stanilisówna 1939; Mazurowa and Mazur 1939; Pileckis 1960, 1976a; Silfverberg 1992, 2004; Gaidienė 1993; Monsevičius 1997; Pileckis and Monsevičius 1997; Althoff and Danilevsky 1997; Danilevsky 2003; Ferenca 2006b; Alekseev 2007; Sama 2009; Ostrauskas and Ferenca 2010; Sama and Löbl 2010.

[*reticulatus **(Razoumowsky, 1789)**]. # 74.Pileckis 1959; disproved by Pileckis (1960).

*Leiopus* **Audinet-Serville, 1835**.

*femoratus **Fairmaire, 1859**. # 75.Ferenca 2004.

*linnei* **Walinn, Nylander & Kvamme, 2009**. Gutowski et al. 2010.

*nebulosus* **(Linnaeus, 1758)**. Stanilisówna 1939; Pileckis 1959, 1960, 1976a; Silfverberg 1992, 2004; Gaidienė 1993; Pileckis and Monsevičius 1997; Althoff and Danilevsky 1997; Šablevičius 2000b; Danilevsky 2003; Inokaitis 2004; Gedminas 2005; Ferenca 2006b; Alekseev 2007; Gedminas et al. 2007; Sama 2009; Ostrauskas and Ferenca 2010; Sama and Löbl 2010.

*punctulatus* **(Paykull, 1800)**. Althoff and Danilevsky 1997; Danilevsky 2003; Silfverberg 2004; Butvila et al. 2007; Sama 2009; Inokaitis 2009; Sama and Löbl 2010.

*Exocentrus* **Dejean, 1835**.

[*adspersus* **Mulsant, 1846**].Known in southern Sweden (Lundberg and Gustafsson 1995), Poland (Burakowski et al. 1989).

*lusitanus* **(Linnaeus, 1767)**. Pileckis 1959, 1960, 1970a, 1976a; Silfverberg 1992, 2004; Gaidienė 1993; Pileckis and Monsevičius 1997; Althoff and Danilevsky 1997; Danilevsky 2003; Alekseev 2007; Sama 2009; Sama and Löbl 2010.

[*punctipennis* **Mulsant & Guillebeau, 1856**].Known in northern Poland (Burakowski et al. 1989), western Belarus (Alexandrovitch et al. 1996).

**Tetropini Portevin, 1927**.

*Tetrops* **Kirby, 1826**.

*praeusta* **(Linnaeus, 1758)**. Heyden 1903; Zawadzki 1936; Stanilisówna 1939; Ogyjewicz 1931, 1932, 1938; Pileckis 1960, 1976a; Lešinskas and Pileckis 1967; Gaidienė and Ferenca 1992; Silfverberg 1992, 2004; Gaidienė 1993; Monsevičius 1997; Pileckis and Monsevičius 1997; Althoff and Danilevsky 1997; Šablevičius 2000b, 2011; Danilevsky 2003; Ferenca 2006b; Alekseev 2007; Sama 2009; Sama and Löbl 2010.

*starkii* **Chevrolat, 1859**. # 76.Althoff and Danilevsky 1997; Danilevsky 2003; Sama 2009; Sama and Löbl 2010.

**Saperdini Mulsant, 1839**.

*Saperda* **Fabricius, 1775** = *Anaerea* Mulsant, 1839; *Compsidia* Mulsant, 1839.

*carcharias* **(Linnaeus, 1758)**. Eichwald 1830; Ivanauskas and Vailionis 1922; Zawadzki 1936; Stanilisówna 1939; Pileckis 1960, 1976a; Lešinskas and Pileckis 1967; Pileckis et al. 1968; Zajančkauskas and Pileckis 1968; Gavelis and Žiogas; 1991; Silfverberg 1992, 2004; Gaidienė 1993; Monsevičius 1997; Pileckis and Monsevičius 1997; Žiogas 1997; Althoff and Danilevsky 1997; Valenta 2000b; Gliaudys 2001; Danilevsky 2003; Ferenca 2006b; Alekseev 2007; Sama 2009; Sama and Löbl 2010; Šablevičius 2011.

*octopunctata* **(Scopoli, 1772)**. Miländer et al. 1984; Pileckis and Monsevičius 1997; Silfverberg 1992, 2004; Alekseev, 2007.

*perforata* **(Pallas, 1773)**. Stanilisówna 1939; Pileckis 1960, 1963b, 1976a; Silfverberg 1992, 2004; Gaidienė 1993; Monsevičius 1997; Pileckis and Monsevičius 1997; Althoff and Danilevsky 1997; Šablevičius 2000b; Gliaudys 2001; Danilevsky 2003; Ehnström et al. 2003; Inokaitis 2004; Ivinskis et al. 2004b; Ferenca 2006, 2006b; Alekseev 2007; Sama 2009; Sama and Löbl 2010.

*populnea* **(Linnaeus, 1758)**. Eichwald 1830; Zawadzki 1936; Stanilisówna 1939; Pileckis 1960, 1976a; Lešinskas and Pileckis 1967; Pileckis et al. 1968; Silfverberg 1992, 2004; Gaidienė 1993; Pileckis and Monsevičius 1997; Žiogas 1997; Althoff and Danilevsky 1997; Valenta 2000b; Šablevičius 2000b; Gliaudys 2001; Danilevsky 2003; Alekseev 2007; Sama 2009; Sama and Löbl 2010.

*punctata* **(Linnaeus, 1767)**. Sama and Löbl 2010.

*scalaris* **(Linnaeus, 1758)**. Zawadzki 1936; Stanilisówna 1939; Pileckis 1960, 1976a; Lešinskas and Pileckis 1967; Zajančkauskas and Pileckis 1968; Gaidienė and Ferenca 1992; Silfverberg 1992, 2004; Gaidienė 1993; Žiogas and Zolubas 1995; Monsevičius 1997; Pileckis and Monsevičius 1997; Althoff and Danilevsky 1997; Šablevičius 2000b, 2011; Gliaudys 2001; Danilevsky 2003; Gedminas 2005; Ferenca 2006b; Alekseev 2007; Gedminas et al. 2007; Vaivilavičius 2008; Sama 2009; Sama and Löbl 2010.

*similis* **Laicharting, 1784**. Zawadzki 1936; Pileckis 1960, 1976a; Silfverberg 1992, 2004; Pileckis and Monsevičius 1997; Althoff and Danilevsky 1997; Danilevsky 2003; Alekseev 2007.

*Menesia* **Mulsant, 1856**.

*bipunctata* **(Zoubkoff, 1829)**. Pileckis 1960, 1976a; Silfverberg 1992, 2004; Pileckis and Monsevičius 1997; Althoff and Danilevsky 1997; Ferenca et al. 2002; Danilevsky 2003; Šablevičius 2003a; Butvila et al. 2007; Alekseev 2007; Sama 2009; Inokaitis 2009; Sama and Löbl 2010.

*Stenostola* **Dejean, 1835**.

*ferrea* **(Schrank, 1776)**. Pileckis 1968a, 1970a, b, 1976a; Silfverberg 1992, 2004; Pileckis and Monsevičius 1997; Althoff and Danilevsky 1997; Danilevsky 2003; Ferenca 2004; Inokaitis 2004; Alekseev 2007; Sama 2009; Sama and Löbl 2010.

[*dubia* **(Laicharting, 1784)**].Known in Latvia (Telnov 2004), Estonia (Süda 2009), Denmark, southern Sweden (Lundberg and Gustafsson 1995), Poland (Burakowski et al. 1989).

**Phytoeciini Mulsant, 1839**.

*Oberea* **Dejean, 1835**.

*erythrocephala* **(Schrank, 1776)**. Pileckis 1959, 1960, 1970a, b, 1976a; Silfverberg 1992, 2004; Pileckis and Monsevičius 1997; Althoff and Danilevsky 1997; Danilevsky 2003; Ferenca 2006b; Alekseev 2007; Sama 2009; Sama and Löbl 2010.

*linearis* **(Linnaeus, 1761)**. Sama and Löbl 2010.

*oculata* **(Linnaeus, 1758)**. Zawadzki 1936; Stanilisówna 1939; Pileckis 1960, 1976a; Lešinskas and Pileckis 1967; Gaidienė and Ferenca 1992; Silfverberg 1992, 2004; Gaidienė 1993; Monsevičius 1997; Pileckis and Monsevičius 1997; Althoff and Danilevsky 1997; Gliaudys 2001; Danilevsky 2003; Ferenca 2006b; Alekseev 2007; Sama 2009; Sama and Löbl 2010; Šablevičius 2011.

*pupillata* **(Gyllenhal, 1817)**. Heyden 1903; Zawadzki 1936; Stanilisówna 1939; Pileckis 1960, 1976a; Gaidienė and Ferenca 1992; Silfverberg 1992, 2004; Gaidienė 1993; Pileckis and Monsevičius 1997; Althoff and Danilevsky 1997; Danilevsky 2003; Alekseev 2007; Sama 2009; Sama and Löbl 2010.

*Phytoecia* **Dejean, 1835**.

*affinis* **(Harrer, 1784)** = *nigripes* (Panzer, 1794) nec (DeGeer, 1775). Zawadzki 1936; Pileckis 1959, 1960, 1976a; Silfverberg 1992, 2004; Pileckis and Monsevičius 1997; Alekseev 2007.

*caerulea* **(Scopoli, 1772)**. Zawadzki 1936; Pileckis 1959 (*Ph. rufimana* Schrank.); Pileckis 1960, 1976; Silfverberg 1992, 2004; Pileckis and Monsevičius 1997.

*coerulescens* **(Scopoli, 1763)**. Lentz 1879; Pileckis 1968b, 1970b, 1976a; Silfverberg 1992, 2004; Pileckis and Monsevičius 1997; Alekseev 2007.

*cylindrica* **(Linnaeus, 1758)**. Monsevičius 1997; Pileckis and Monsevičius 1997; Althoff and Danilevsky 1997; Danilevsky 2003; Šablevičius 2003a; Silfverberg 2004; Butvila et al. 2007; Alekseev 2007; Sama 2009; Sama and Löbl 2010.

*nigricornis* **(Fabricius, 1781)**. Monsevičius 1988b; Silfverberg 1992, 2004; Pileckis and Monsevičius 1997; Althoff and Danilevsky 1997; Danilevsky 2003; Šablevičius 2004; Alekseev 2007; Sama 2009; Sama and Löbl 2010.

*pustulata* **(Schrank, 1776)**. Pileckis 1960, 1976a; Silfverberg 1992, 2004; Gaidienė 1993; Pileckis and Monsevičius 1997; Alekseev, 2007.

[*rufipes* **(Olivier, 1795)**]. Known in Latvia, Belarus and Poland (Althoff and Danilevsky 1997; Danilevsky 2003).

*virgula* **(Charpentier, 1825)**. Pileckis 1959, 1960, 1970b, 1976a; Silfverberg 1992, 2004; Pileckis and Monsevičius 1997; Ferenca et al. 2006, 2007; Alekseev 2007.

**Agapanthiini Mulsant, 1839**.

*Agapanthia* **Audinet-Serville, 1835**.

[*intermedia* **Ganglbauer, 1883**]. Known in Belarus, Poland (Althoff and Danilevsky 1997; Danilevsky 2003).

*villosoviridescens* **(DeGeer, 1775)**. Zawadzki 1936; Pileckis 1960, 1976a, b, 1979; Zajančkauskas and Pileckis 1968; Silfverberg 1992, 2004; Gaidienė 1993; Pileckis and Monsevičius 1997; Monsevičius 1997; Althoff and Danilevsky 1997; Šablevičius 2000b, 2011; Gliaudys 2001; Danilevsky 2003; Alekseev 2007; Vaivilavičius 2008; Sama 2009; Sama and Löbl 2010.

[*violacea* **(Fabricius, 1775)**]. Known in Belarus (Alexandrovitch et al. 1996), northern Poland (Burakowski et al. 1989).

**MEGALOPODIDAE Latreille, 1802**. (Chrysomelidae)

**Zeugophorinae** **Böving and Craighead, 1931**

*Zeugophora* **Kunze, 1818**.

*frontalis* **Suffrian, 1840** = *flavicollis* auct. nec (Marsham, 1802). Pileckis 1959, 1960, 1976a; Silfverberg 1992, 2004, 2010a; Gaidienė 1993; Monsevičius 1997; Pileckis and Monsevičius 1997; Barševskis 2001a.

*scutellaris* **Suffrian, 1840**. Pileckis and Monsevičius 1997; Silfverberg 2004, 2010a.

*subspinosa* **(Fabricius, 1781)**. Pileckis and Monsevičius 1997; Silfverberg 2004, 2010a.

*turneri* **Power, 1863**. Pileckis 1976a; Silfverberg 1992, 2004, 2010a; Monsevičius 1997; Pileckis and Monsevičius 1997.

**ORSODACNIDAE Thomson, 1859**. (Chrysomelidae)

**Orsodacninae** **Thomson, 1859**.

*Orsodacne* **Latreille, 1802**.

*cerasi* **(Linnaeus, 1758)**. Eichwald 1830; Pileckis 1960, 1976a; Lešinskas and Pileckis 1967; Lopatin 1986; Silfverberg 1992, 2004; 2010b; Gaidienė 1993; Pileckis and Monsevičius 1997; Ferenca 2006b.

**CHRYSOMELIDAE Latreille, 1802**.

**Bruchinae** **Latreille, 1802**. (Bruchidae)

**Bruchini Latreille, 1802**.

*Bruchus* **Linnaeus, 1767**.

*affinis* **Frölich, 1799**. Pileckis 1962, 1963b, 1972, 1976a; Zubrys 1967; Silfverberg 1992, 2004; Pileckis and Monsevičius 1997; Šablevičius 2004; Anton 2010.

*atomarius* **(Linnaeus, 1761)**. Mastauskis 1936; Pileckis 1960, 1972, 1976a; Lešinskas and Pileckis 1967; Zubrys 1967; Pileckis and Vengeliauskaitė 1977, 1996; Silfverberg 1992, 2004; Gaidienė 1993; Pileckis and Monsevičius 1997; Ferenca 2006b; Audisio 2009; Anton 2010.

[*laticollis* **Boheman, 1833**]. Known in Latvia (Telnov et al. 2005).

*loti* **Paykull, 1800**. Pileckis 1960, 1972, 1976a; Lešinskas and Pileckis 1967; Zubrys 1967; Silfverberg 1992, 2004; Gaidienė 1993; Pileckis and Monsevičius 1997; Žiogas and Zolubas 2005; Ferenca 2006b; Audisio 2009; Anton 2010.

[*luteicornis* **Illiger, 1794**]. Known in southern Sweden (Lundberg and Gustafsson 1995), northern Belarus (Alexandrovitch et al. 1996), northern Poland (Burakowski et al. 1991).

*pisorum* **(Linnaeus, 1758)**. Ivanauskas and Vailionis 1922; Ogyjewicz 1929, 1931, 1932, 1938; Pileckis 1960, 1972, 1976a, 1998; Lešinskas and Pileckis 1967; Zubrys 1967; Pileckis and Vengeliauskaitė 1977, 1996; Pileckis et al. 1983, 1994b; Silfverberg 1992, 2004; Pileckis and Monsevičius 1997; Gliaudys 2001; Audisio 2009.

*rufimanus* **Boheman, 1833**. Pileckis 1960, 1972, 1976a; Lešinskas and Pileckis 1967; Zubrys 1967; Pileckis and Vengeliauskaitė 1977, 1996; Silfverberg 1992, 2004; Pileckis and Monsevičius 1997; Audisio 2009; Anton 2010.

[*viciae* **Olivier, 1795**]. Known in Denmark, southern Sweden (Lundberg and Gustafsson 1995), northeastern Poland (Burakowski et al. 1991).

*Bruchidius* **Schilsky, 1905**.

[*ater* **(Marsham, 1802) =** *villosus* (Fabricius, 1792) = *fasciatus* (Olivier, 1795)]. Recently discovered in Latvia (Bukejs 2010c), known in Denmark, southern Sweden (Lundberg and Gustafsson 1995), northeastern Poland (Burakowski et al. 1991), Belarus (Alexandrovitch et al. 1996).

*marginalis* **(Fabricius, 1777)**. Gaidienė 1993; Pileckis and Monsevičius 1997; Barševskis 2001a; Šablevičius 2000a, 2003a, 2004, 2007; Ferenca et al. 2002, 2006, 2007; Silfverberg 2004.

*unicolor* **(Olivier, 1795)**. Pileckis and Monsevičius 1982, 1997; Silfverberg 1992, 2004.

*Callosobruchus* **Pic, 1902**.

*chinensis* **(Linnaeus, 1758)**. # 77. Audisio 2009.

*Acanthoscelides* **Schilsky, 1905**.

*obtectus* **(Say, 1831)** = *obsoletus* auct. nec (Say, 1831). Pileckis 1972, 1976a, 1998; Pileckis and Vengeliauskaitė 1977, 1996; Pileckis et al. 1983, 1994b; Silfverberg 1992, 2004; Gaidienė 1993; Pileckis and Monsevičius 1997; Gliaudys 2001; Audisio 2009; Anton 2010.

**Amblycerini Bridwell, 1932**.

*Spermophagus* **Schönherr, 1833**.

*calystegiae* **(Lukjanovic & Ter-Minasjan, 1957)**. Audisio 2009; Bukejs et al. 2011.

*sericeus* **(Geoffroy, 1785)**. Pileckis and Monsevičius 1997; Silfverberg 2004; Audisio 2009.

**Donaciinae** **Kirby, 1837**.

**Donaciini Kirby, 1837**.

*Macroplea* **Samouelle, 1819** = *Haemonia* Dejean, 1821.

*appendiculata* **(Panzer, 1794)**. Pileckis 1976a; Lopatin 1986; Silfverberg 1992, 2004, 2010c; Gaidienė 1993; Pileckis and Monsevičius 1997; Kovács et al. 2008; Audisio 2009.

[*mutica* **(Fabricius, 1792)**]. Known in Latvia (Telnov 2004), Denmark, Estonia, throughout Sweden (Lundberg and Gustafsson 1995), northern Belarus (Alexandrovitch et al. 1996), northern Poland (Burakowski et al. 1990); Kaliningrad region (Alekseev 2003).

*Donacia* **Fabricius, 1775**.

*antiqua* **Kunze, 1818**. Pileckis and Monsevičius 1997; Barševskis 2001a; Silfverberg 2004, 2010c.

*aquatica* **(Linnaeus, 1758)**. Pileckis 1960, 1976a; Zajančkauskas and Pileckis 1968; Silfverberg 1992, 2004, 2010c; Gaidienė 1993; Monsevičius 1997; Pileckis and Monsevičius 1997; Ferenca 2006b; Audisio 2009; Šablevičius 2011.

*bicolora* **Zschach, 1788**. Pileckis 1960, 1976a; Pileckis and Zajančkauskas 1968; Silfverberg 1992, 2004, 2010c; Monsevičius 1997; Pileckis and Monsevičius 1997; Ferenca 2006b.

*brevicornis* **Ahrens, 1810**. Pileckis 1960, 1976a; Silfverberg 1992, 2004, 2010c; Gaidienė 1993; Pileckis and Monsevičius 1997; Šablevičius 2003a; Ferenca 2006b; Audisio 2009.

*brevitarsis* **Thomson, 1884**. Silfverberg 2010c.

*cinerea* **Herbst, 1784**. Pileckis 1960, 1976a; Zajančkauskas and Pileckis 1968; Silfverberg 1992, 2004, 2010c; Gaidienė 1993; Monsevičius 1997; Pileckis and Monsevičius 1997; Gliaudys 2001; Ferenca 2006b; Audisio 2009.

*clavipes* **Fabricius, 1792**. Eichwald 1830; Pileckis 1960, 1976a; Zajančkauskas and Pileckis 1968; Ivinskis et al. 1984; Silfverberg 1992, 2004, 2010c; Gaidienė 1993; Monsevičius 1997; Pileckis and Monsevičius 1997; Ferenca 2006b; Audisio 2009.

*crassipes* **Fabricius, 1775**. Pileckis 1960, 1976a; Zajančkauskas and Pileckis 1968; Silfverberg 1992, 2004, 2010c; Gaidienė 1993; Monsevičius 1997; Pileckis and Monsevičius 1997; Ferenca 2006b; Audisio 2009.

*dentata* **Hoppe, 1795**. Eichwald 1830; Pileckis 1960, 1976a; Zajančkauskas and Pileckis 1968; Silfverberg 1992, 2004, 2010c; Gaidienė 1993; Monsevičius 1997; Pileckis and Monsevičius 1997; Šablevičius 2000b, 2011; Ferenca 2006b; Audisio 2009.

*fennica* **(Paykull, 1800)**. Barševskis 2001a; Silfverberg 2004, 2010c.

*impressa* **Paykull, 1799**. Pileckis 1960, 1976a; Silfverberg 1992, 2004, 2010c; Gaidienė 1993; Monsevičius 1997; Pileckis and Monsevičius 1997; Ferenca 2006b; Audisio 2009.

*malinovskyi* **Ahrens, 1810**. Gaidienė 1993; Pileckis and Monsevičius 1997; Silfverberg 2004, 2010c; Audisio 2009.

*marginata* **Hoppe, 1795**. Pileckis 1960, 1976a; Zajančkauskas and Pileckis 1968; Silfverberg 1992, 2004; Monsevičius 1997; Pileckis and Monsevičius 1997; Ferenca 2006b; Audisio 2009; 2010c.

*obscura* **Gyllenhal, 1813**.Audisio 2009; Silfverberg 2010c.

*semicuprea* **Panzer, 1796**. Pileckis 1960, 1963b, 1976a; Zajančkauskas and Pileckis 1968; Ivinskis et al. 1984; Silfverberg 1992, 2004, 2010c; Gaidienė 1993; Monsevičius 1997; Pileckis and Monsevičius 1997; Gliaudys 2001; Ferenca 2006b; Audisio 2009.

*simplex* **Fabricius, 1775**. Pileckis 1960, 1976a; Zajančkauskas and Pileckis 1968; Silfverberg 1992, 2004, 2010c; Gaidienė 1993; Monsevičius 1997; Pileckis and Monsevičius 1997; Ferenca 2006b; Audisio 2009.

*sparganii* **Ahrens, 1810**. Gaidienė 1993; Pileckis and Monsevičius 1997; Barševskis 2001a; Silfverberg 2004, 2010c; Audisio 2009.

*thalassina* **Germar, 1811**. Pileckis1968b, 1976a; Silfverberg 1992, 2004, 2010c; Monsevičius 1997; Pileckis and Monsevičius 1997.

*tomentosa* **Ahrens, 1810**. Pileckis 1960, 1976a; Zajančkauskas and Pileckis 1968; Silfverberg 1992, 2004, 2010c; Gaidienė 1993; Monsevičius 1997; Pileckis and Monsevičius 1997; Ferenca 2006b; Audisio 2009.

*versicolorea* **(Brahm, 1790)**. Silfverberg 1992, 2004, 2010c; Gaidienė 1993; Pileckis and Monsevičius 1997; Audisio 2009.

*vulgaris* **Zschach, 1788**. Pileckis 1960, 1976a; Zajančkauskas and Pileckis 1968; Silfverberg 1992, 2004, 2010c; Gaidienė 1993; Monsevičius 1997; Pileckis and Monsevičius 1997; Ferenca 2006b; Audisio 2009.

*Plateumaris* **Thomson, 1859**.

*affinis* **(Kunze, 1818)**. Pileckis 1960, 1963b, 1976a; Zajančkauskas and Pileckis 1968; Silfverberg 1992, 2004, 2010c; Gaidienė 1993; Monsevičius 1997; Pileckis and Monsevičius 1997; Audisio 2009.

*braccata* **(Scopoli, 1772)**. Pileckis 1960, 1976a; Zajančkauskas and Pileckis 1968; Strazdienė 1976; Silfverberg 1992, 2004, 2010c; Gaidienė 1993; Monsevičius 1997; Pileckis and Monsevičius 1997; Ferenca 2006b; Audisio 2009.

*consimilis* **(Schrank, 1781)**. Pileckis 1960, 1976a; Zajančkauskas and Pileckis 1968; Silfverberg 1992, 2004, 2010c; Gaidienė 1993; Monsevičius 1997; Pileckis and Monsevičius 1997; Ferenca 2006b; Audisio 2009.

*discolor* **(Herbst, 1795)**. Monsevičius 1997; Silfverberg 2004, 2010c; Ivinskis et al. 2009.

*rustica* **(Kunze, 1818)**. Pileckis 1968b, 1976a; Zajančkauskas and Pileckis 1968; Silfverberg 1992, 2004, 2010c; Monsevičius 1997; Pileckis and Monsevičius 1997; Ferenca 2006b.

*sericea* **(Linnaeus, 1758)**. Pileckis 1960, 1976a; Zajančkauskas and Pileckis 1968; Silfverberg 1992, 2004, 2010c; Gaidienė 1993; Monsevičius 1997; Pileckis and Monsevičius 1997; Audisio 2009.

**Criocerinae** **Latreille, 1804**.

**Criocerini Latreille, 1804**

*Crioceris* **Geoffroy, 1762**.

*asparagi* **(Linnaeus, 1758)**. Tenenbaum 1931; Pileckis and Monsevičius 1997; Silfverberg 2004; Schmitt 2010.

*duodecimpunctata* **(Linnaeus, 1758)**. Pileckis 1960, 1970b, 1976a; Pileckis et al. 1983; Silfverberg 1992, 2004; Gaidienė 1993; Pileckis and Vengeliauskaitė 1996; Pileckis and Monsevičius 1997; Šablevičius 2000b; Gliaudys 2001; Audisio 2009; Schmitt 2010.

[*quatuordecimpunctata* **(Scopoli, 1763)**]. Known in Kaliningrad region (Alekseev 2003), Belarus (Alexandrovitch et al. 1996), Poland (Burakowski et al. 1990).

*Lilioceris* **Reitter, 1912**.

*lilii* **(Scopoli, 1763)**. Pileckis 1960, 1976a; Lopatin 1986; Silfverberg 1992, 2004; Gaidienė 1993; Pileckis and Vengeliauskaitė 1996; Pileckis and Monsevičius 1997; Šablevičius 2000b, 2011; Gliaudys 2001; Audisio 2009; Schmitt 2010.

*merdigera* **(Linnaeus, 1758)**. Eichwald 1830; Pileckis 1960, 1976a; Silfverberg 1992, 2004; Gaidienė 1993; Monsevičius 1997; Pileckis and Monsevičius 1997; Ferenca 2006b; Audisio 2009; Schmitt 2010.

**Lemini Gyllenhal, 1813**

*Lema* **Fabricius, 1798**.

*cyanella* **(Linnaeus, 1758)** = *puncticollis* (Curtis, 1830). Pileckis 1960, 1976a; Zajančkauskas and Pileckis 1968; Pileckis and Vengeliauskaitė 1977, 1996; Dabkevičius 1984; Silfverberg 1992, 2004; Gaidienė 1993; Pileckis et al. 1994b; Monsevičius 1997; Pileckis and Monsevičius 1997; Šurkus and Gaurilčikienė 2002; Ferenca 2006b; Audisio 2009; Schmitt 2010.

*Oulema* **Gozis, 1886**.

*duftschmidi* **(Redtenbacher, 1874)**. Bukejs and Ferenca 2010; Bukejs et al. 2011.

*erichsonii* **(Suffrian, 1841)**. # 78. Pileckis 1960, 1976a; Silfverberg 1992, 2004; Monsevičius 1997; Pileckis and Monsevičius 1997; Bukejs and Ferenca 2010; Schmitt 2010.

*gallaeciana* **(Heyden, 1870)** = *lichenis* (Weise, 1881). Pileckis 1960, 1976a; Gaidienė 1993; Monsevičius 1997; Pileckis and Monsevičius 1997; Šablevičius 2000b; Silfverberg 1992, 2004; Audisio 2009; Bukejs and Ferenca 2010; Schmitt 2010.

*melanopus* **(Linnaeus, 1758)**. Pileckis 1960, 1976a; Lešinskas and Pileckis 1967; Dabkevičius 1984; Pileckis and Vengeliauskaitė 1977, 1996; Gaidienė 1993; Pileckis et al. 1994b; Monsevičius 1997; Pileckis and Monsevičius 1997; Šablevičius 2000b; Gliaudys 2001; Šurkus and Gaurilčikienė 2002; Silfverberg 1992, 2004; Ferenca 2006b; Audisio 2009; Bukejs and Ferenca 2010; Schmitt 2010.

*septentrionis* **(Weise, 1880)**. # 78. Pileckis 1960, 1976a; Silfverberg 1992, 2004; Pileckis and Monsevičius 1997; Bukejs and Ferenca 2010; Schmitt 2010; Bukejs et al. 2011.

*tristis* **(Herbst, 1786)**. Tamutis and Ferenca 2006; Ferenca et al. 2006, 2007; Bukejs and Ferenca 2010; Schmitt 2010.

**Cassidinae** **Gyllenhal, 1813**.

**Hispini Gyllenhal, 1813**.

*Hispa* **Linnaeus, 1767**.

*atra* **Linnaeus, 1767**. Warchałowski 1978; Pileckis and Monsevičius 1997 (*Hispella*); Ferenca et al. 2002; Silfverberg 2004; Šablevičius 2011.

**Cassidini Gyllenhal, 1813**.

*Pilemostoma* **Desbrocher des Loges, 1891**.

[*fastuosa **(Schaller, 1783)**]. # 79. Šablevičius 2000a; Silfverberg 2004.

*Hypocassida* **Weise, 1893**.

*subferruginea* **(Schrank, 1776)**. Mazurowa and Mazur 1939; Pileckis 1960, 1976a; Silfverberg 1992, 2004; Pileckis and Monsevičius 1997; Audisio 2009; Borowiec and Sekerka 2010.

*Cassida* **Linnaeus, 1758**.

[*azurea **Fabricius, 1801**]. # 80. Tamutis 2003.

[*berolinensis* **Suffrian, 1844**]. Known in southern Sweden (Lundberg and Gustafsson 1995) northern Poland (Burakowski et al. 1991).

*denticollis* **Suffrian, 1844**. Mazurowa and Mazur 1939; Pileckis 1960, 1976a; Zajančkauskas and Pileckis 1968; Silfverberg 1992, 2004; Gaidienė 1993; Monsevičius 1997; Pileckis and Monsevičius 1997; Ferenca 2006b; Audisio 2009; Borowiec and Sekerka 2010.

*ferruginea* **Goeze, 1777**. Ferenca et al. 2002.

*flaveola* **Thunberg, 1794**. Pileckis 1960, 1976a; Zajančkauskas and Pileckis 1968; Silfverberg 1992, 2004; Gaidienė 1993; Monsevičius 1997; Pileckis and Monsevičius 1997; Tamutis and Zolubas 2001; Ferenca 2006b; Audisio 2009; Borowiec and Sekerka 2010.

*hemisphaerica* **Herbst, 1799**. Pileckis 1960, 1976a; Lopatin 1986; Silfverberg 1992, 2004; Gaidienė 1993; Pileckis and Monsevičius 1997; Ferenca 2006b; Audisio 2009; Borowiec and Sekerka 2010.

[*lineola* **Creutzer, 1799**]. Known in Latvia (Bukejs et al. 2009), Belarus (Alexandrovitch et al. 1996), northern Poland (Burakowski et al. 1991).

RDB*margaritacea* **Schaller, 1783**. Monsevičius 1988b; Silfverberg 1992, 2004; Pileckis and Monsevičius 1997; Ivinskis et al. 1997a, 2000, 2004a; Ferenca et al. 2002; Rašomavičius 2007; Audisio 2009; Alekseev 2010b; Borowiec and Sekerka 2010.

*murraea* **Linnaeus, 1767**. Pileckis 1960, 1976a; Silfverberg 1992, 2004; Pileckis and Monsevičius 1997; Ferenca et al. 2002; Ivinskis 2003; Ferenca 2006b; Audisio 2009; Borowiec and Sekerka 2010; Šablevičius 2011.

*nebulosa* **Linnaeus, 1758**. Eichwald 1830; Mazurowa and Mazur 1939; Pileckis 1960, 1976a; Lešinskas and Pileckis 1967; Pileckis and Vengeliauskaitė 1977, 1996; Pileckis et al. 1983, 1994b; Silfverberg 1992, 2004; Gaidienė 1993; Monsevičius 1997; Pileckis and Monsevičius 1997; Šablevičius 2000b, 2011; Gliaudys 2001; Ferenca 2006b; Audisio 2009; Borowiec and Sekerka 2010.

*nobilis* **Linnaeus, 1758**. Eichwald 1830; Pileckis 1960, 1976a; Silfverberg 1992, 2004; Gaidienė 1993; Pileckis et al. 1994b; Pileckis and Monsevičius 1997; Audisio 2009; Borowiec and Sekerka 2010; Šablevičius 2011.

*pannonica* **Suffrian, 1844**. Pileckis and Monsevičius 1997; Silfverberg 2004.

*panzeri* **Weise, 1907**. Silfverberg 1992, 2004; Gaidienė 1993; Pileckis and Monsevičius 1997; Ferenca et al. 2002; Šablevičius 2003, 2004; Audisio 2009; Borowiec and Sekerka 2010.

*prasina* **Illiger, 1798**. Pileckis 1960, 1976a; Silfverberg 1992, 2004; Gaidienė 1993; Pileckis and Monsevičius 1997; Audisio 2009; Borowiec and Sekerka 2010.

*rubiginosa* **O.F. Müller, 1776**. Pileckis 1960, 1976a; Zajančkauskas and Pileckis 1968; Silfverberg 1992, 2004; Gaidienė 1993; Monsevičius 1997; Pileckis and Monsevičius 1997; Ferenca 2006b; Audisio 2009; Borowiec and Sekerka 2010.

[*rufovirens* **Suffrian, 1844**]. Known in northern Poland (Burakowski et al. 1991).

*sanguinolenta* **O.F. Müller, 1776**. Pileckis 1960, 1976a; Silfverberg 1992, 2004; Gaidienė 1993; Pileckis and Monsevičius 1997; Audisio 2009; Borowiec and Sekerka 2010.

*sanguinosa* **Suffrian, 1844**. Pileckis 1960, 1976a; Silfverberg 1992, 2004; Gaidienė 1993; Monsevičius 1997; Pileckis and Monsevičius 1997; Ferenca 2006b; Audisio 2009; Borowiec and Sekerka 2010.

*seladonia* **Gyllenhal, 1827**. Heyden 1903; Pileckis 1960, 1976a; Silfverberg 1992, 2004; Pileckis and Monsevičius 1997; Šablevičius 2003a; Audisio 2009; Borowiec and Sekerka 2010.

*stigmatica* **Suffrian, 1844**. Pileckis and Monsevičius 1997; Monsevičius 1997; Silfverberg 2004; Ostrauskas and Ferenca 2010.

*vibex* **Linnaeus, 1767**. Pileckis 1960, 1976a; Silfverberg 1992, 2004; Gaidienė 1993; Pileckis and Monsevičius 1997; Ferenca 2006b; Vaivilavičius 2008; Audisio 2009; Borowiec and Sekerka 2010.

*viridis* **Linnaeus, 1758**. Eichwald 1830; Mazurowa and Mazur 1939; Pileckis 1960, 1976a; Silfverberg 1992, 2004; Gaidienė 1993; Pileckis and Monsevičius 1997; Šablevičius 2000b, 2011; Gliaudys 2001; Ferenca 2006b; Vaivilavičius 2008; Audisio 2009; Borowiec and Sekerka 2010.

*vittata* **Villers, 1789**. Pileckis 1960, 1976a; Silfverberg 1992, 2004; Gaidienė 1993; Pileckis and Monsevičius 1997; Ferenca 2006b; Audisio 2009; Borowiec and Sekerka 2010.

**Chrysomelinae** **Latreille, 1802**.

**Chrysomelini Latreille, 1802**.

*Leptinotarsa* **Chevrolat, 1836**.

*decemlineata* **(Say, 1824)**. Pileckis 1960, 1970a, 1976a; Lešinskas and Pileckis 1967; Šurkus 1971; Žematienė 1973a, b; Pileckis and Vengeliauskaitė 1977, 1996; Pileckis et al. 1983, 1994b; Silfverberg 1992, 2004; Gaidienė 1993; Monsevičius 1997; Pileckis and Monsevičius 1997; Šablevičius 2000b; Gliaudys 2001; Šurkus and Gaurilčikienė 2002; Vaivilavičius 2008; Audisio 2009.

*Chrysolina* **Motschulsky, 1860**.

[*americana* **(Linnaeus, 1758)**]. Known in Latvia (Telnov 2004; Bukejs and Telnov 2010).

*analis* **(Linnaeus, 1767)**. Pileckis 1960, 1963b, 1976a; Silfverberg 1992, 2004; Gaidienė 1993; Monsevičius 1997; Pileckis and Monsevičius 1997; Ferenca 2006b; Bukejs et al. 2011.

*aurichalcea* **(Gebler, 1825)**. Gaidienė 1993; Bukejs et al. 2011.

*carnifex* **(Fabricius, 1792)**. Pileckis 1960, 1970b, 1976a; Silfverberg 1992, 2004; Gaidienė 1993; Pileckis and Monsevičius 1997; Ferenca 2006b; Bukejs et al. 2011.

*cerealis* **(Linnaeus, 1767)**. Mazurowa and Mazur 1939; Pileckis 1960, 1976a; Lopatin 1986; Silfverberg 1992, 2004; Gaidienė 1993; Pileckis and Monsevičius 1997; Ferenca 2006b; Kippenberg 2010.

[*coerulans* **(L.G. Scriba, 1791)**]. Recently found in Latvia (Bukejs 2009c), known in Estonia (Lundberg and Gustafsson 1995), western Belarus (Alexandrovitch et al. 1996), northern Poland (Burakowski et al. 1990).

*fastuosa fastuosa* **(Scopoli, 1763)**. Eichwald 1830; Heyden 1903; Mazurowa and Mazur 1939; Pileckis 1960, 1963b, 1976a; Lešinskas and Pileckis 1967; Zajančkauskas and Pileckis 1968; Silfverberg 1992, 2004; Gaidienė 1993; Monsevičius 1997; Pileckis and Monsevičius 1997 (*Dlochrysa*); Šablevičius 2000b, 2011; Gliaudys 2001; Ferenca 2006b.

*geminata* **(Paykull, 1799)**. Pileckis and Monsevičius 1997; Silfverberg 2004.

*graminis* **(Linnaeus, 1758)**. Pileckis 1960, 1976a; Silfverberg 1992, 2004; Gaidienė 1993; Monsevičius 1997; Pileckis and Monsevičius 1997.

*gypsophilae* **(Küster, 1845)**. Mazurowa and Mazur 1939; Pileckis 1960, 1976a; Lešinskas and Pileckis 1967; Ivinskis et al. 1984; Silfverberg 1992, 2004; Gaidienė 1993; Monsevičius 1997; Pileckis and Monsevičius 1997; Gliaudys 2001.

*haemoptera* **(Linnaeus, 1758)**. Pileckis 1968a, 1976a; Lopatin 1986; Silfverberg 1992, 2004; Gaidienė 1993; Pileckis and Monsevičius 1997; Alekseev 2008a; Kippenberg 2010.

*herbacea* **(Duftschmid, 1825)** *= menthastri* (Suffrian, 1851). # 81. Tamutis 2003; Bukejs et al. 2011.

*hyperici* **(Forster, 1771)**. Pileckis 1968a, 1976a; Silfverberg 1992, 2004; Monsevičius 1997; Pileckis and Monsevičius 1997; Gliaudys 2001; Bukejs et al. 2011.

*kuesteri* **(Helliesen, 1912)**.Audisio 2009.

*limbata* **(Fabricius, 1775)**. Eichwald 1830; Pileckis 1960, 1976a; Silfverberg 1992, 2004; Gaidienė 1993; Pileckis and Monsevičius 1997; Bukejs et al. 2011.

*marginata* **(Linnaeus, 1758)**. Pileckis 1960, 1976a; Silfverberg 1992, 2004; Gaidienė 1993; Pileckis and Monsevičius 1997; Ferenca 2006b; Kippenberg 2010.

*oricalcia* **(O.F. Müller, 1776)**. Gaidienė and Ferenca 1988; Silfverberg 1992, 2004; Pileckis and Monsevičius 1997; Bukejs et al. 2011.

*polita* **(Linnaeus, 1758)**. Eichwald 1830; Heyden 1903; Pileckis 1960, 1963b, 1976a; Zajančkauskas and Pileckis 1968; Silfverberg 1992, 2004; Gaidienė 1993; Monsevičius 1997; Pileckis and Monsevičius 1997; Šablevičius 2000b; Gliaudys 2001; Tamutis and Zolubas 2001; Ferenca 2006b; Vaivilavičius 2008; Audisio 2009; Kippenberg 2010.

*reitteri* **(Weise, 1884)** = *lurida* (Linnaeus, 1767) nec (Scopoli, 1763). Pileckis 1960, 1970b, 1976a; Silfverberg 1992, 2004; Pileckis and Monsevičius 1997.

*sanguinolenta* **(Linnaeus, 1758)**. Eichwald 1830; Pileckis 1960, 1963b, 1976a; Silfverberg 1992, 2004; Gaidienė 1993; Pileckis and Monsevičius 1997; Šablevičius 2000b, 2011; Ferenca 2006b.

*staphylaea* **(Linnaeus, 1758)**. Eichwald 1830; Pileckis 1960, 1976a; Lešinskas and Pileckis 1967; Zajančkauskas and Pileckis 1968; Silfverberg 1992, 2004; Gaidienė 1993; Monsevičius 1997; Pileckis and Monsevičius 1997; Šablevičius 2000b; Gliaudys 2001; Ferenca 2006b; Audisio 2009; Kippenberg 2010.

*sturmi* **(Westhoff, 1882)** = *diversipes* (Bedel, 1892) = *violacea* auct. nec (O.F. Müller, 1776). Pileckis 1960, 1963b, 1976a; Silfverberg 1992, 2004; Gaidienė 1993; Monsevičius 1997; Pileckis and Monsevičius 1997; Ferenca 2006b; Audisio 2009; Kippenberg 2010.

*varians* **(Schaller, 1783)**. Mazurowa and Mazur 1939; Pileckis 1960, 1963b, 1976a; Zajančkauskas and Pileckis 1968; Silfverberg 1992, 2004; Gaidienė 1993; Monsevičius 1997; Pileckis and Monsevičius 1997; Ferenca 2006b; Audisio 2009; Kippenberg 2010.

*Oreina* **Chevrolat, 1836** = *Chrysochloa* Hope, 1840.

[*caerulea nobilis* **(Waltl, 1839)**]. Known in western Belarus (Alexandrovitch et al. 1996), Poland (Burakowski et al. 1990), Denmark (Lundberg and Gustafsson 1995).

*Colaphus* **Dahl, 1823** = *Colaphellus* Weise, 1916.

*sophiae* **(Schaller, 1783)**. Ogyjewicz 1929, 1931, 1938; Mazurowa and Mazur 1939; Pileckis 1960, 1963b, 1976a; Silfverberg 1992, 2004; Pileckis and Monsevičius 1997; Gliaudys 2001; Audisio 2009.

*Gastrophysa* **Chevrolat, 1836**.

*polygoni* **(Linnaeus, 1758)**. Eichwald 1830; Roubal 1910; Ogijewicz 1929; Mazurowa and Mazur 1939; Pileckis 1960, 1976a; Silfverberg 1992, 2004; Gaidienė 1993; Monsevičius 1997; Pileckis and Monsevičius 1997; Šablevičius 2000b; Gliaudys 2001; Ferenca 2006b; Audisio 2009.

*viridula* **(DeGeer, 1775)**. Mazurowa and Mazur 1939; Pileckis 1960, 1976a; Lešinskas and Pileckis 1967; Zajančkauskas and Pileckis 1968; Silfverberg 1992, 2004; Gaidienė 1993; Monsevičius 1997; Pileckis and Monsevičius 1997; Šablevičius 2000b; Gliaudys 2001; Ferenca 2006b.

*Phaedon* **Dahl, 1823**.

*armoraciae* **(Linnaeus, 1758)**. Pileckis 1960, 1976a; Silfverberg 1992, 2004; Gaidienė 1993; Pileckis and Monsevičius 1997; Ferenca 2006b; Alekseev 2008a; Audisio 2009.

*cochleariae* **(Fabricius, 1792)**. Mastauskis 1927; Pileckis 1960, 1976a; Lešinskas and Pileckis 1967; Pileckis and Vengeliauskaitė 1977, 1996; Pileckis et al. 1983; Silfverberg 1992, 2004; Gaidienė 1993; Pileckis and Monsevičius 1997; Gliaudys 2001.

[*concinnus* **(Stephens, 1831)**]. Known in Denmark, Estonia, throughout Sweden (Lundberg and Gustafsson 1995), northern Poland (Burakowski et al. 1990).

*laevigatus* **(Duftschmid, 1825)**. Roubal 1910; Pileckis 1960, 1976a; Silfverberg 1992, 2004; Pileckis and Monsevičius 1997.

*Hydrothassa* **Thomson, 1859**.

*glabra* **(Herbst, 1783)**. Mazurowa and Mazur 1939; Pileckis 1960, 1976a; Silfverberg 1992, 2004; Gaidienė 1993; Pileckis and Monsevičius 1997; Ferenca 2006b.

*marginella* **(Linnaeus, 1758)**. Roubal 1910; Pileckis 1960, 1976a; Silfverberg 1992, 2004; Gaidienė 1993; Monsevičius 1997; Pileckis and Monsevičius 1997; Šablevičius 2000b, 2011; Ferenca 2006b; Audisio 2009.

*hannoveriana* **(Fabricius, 1775)**. Pileckis 1968a, 1976a; Silfverberg 1992, 2004; Gaidienė 1993; Pileckis and Monsevičius 1997; Žiogas and Zolubas 2005; Audisio 2009.

*Prasocuris* **Latreille, 1802**.

*junci* **(Brahm, 1790)**. Tamutis 2003; Silfverberg 2004; Audisio 2009.

*phellandrii* **(Linnaeus, 1758)**. Eichwald 1830; Pileckis 1960, 1976a; Zajančkauskas and Pileckis 1968; Silfverberg 1992, 2004; Gaidienė 1993; Monsevičius 1997; Pileckis and Monsevičius 1997; Žiogas and Zolubas 2005; Ferenca 2006b; Audisio 2009; Šablevičius 2011.

*Plagiodera* **Chevrolat, 1836**.

*versicolora* **(Laicharting, 1781)**. Heyden 1903; Pileckis 1960, 1976a; Pileckis et al. 1968; Pileckis and Zajančkauskas 1968; Silfverberg 1992, 2004; Gaidienė 1993; Monsevičius 1997; Pileckis and Monsevičius 1997; Šablevičius 2000b.

*Chrysomela* **Linnaeus, 1758**.

*collaris* **Linnaeus, 1758**. Pileckis 1960, 1976a; Silfverberg 1992, 2004; Gaidienė 1993; Pileckis and Monsevičius 1997; Monsevičius 1997; Ferenca 2006b.

*cuprea* **Fabricius, 1775**. Pileckis 1959, 1960, 1976a; Silfverberg 1992, 2004; Pileckis and Monsevičius 1997; Šablevičius 2003a; Alekseev 2003.

*lapponica* **Linnaeus, 1758**. Mazurowa and Mazur 1939; Pileckis 1960, 1970a, 1976a, b, 1979; Pileckis and Monsevičius 1997; Silfverberg 1992, 2004.

*populi* **Linnaeus, 1758**. Eichwald 1830; Heyden 1903; Ogyjewicz 1938; Pileckis 1960, 1976a; Lešinskas and Pileckis 1967; Pileckis et al. 1968; Ivinskis et al. 1984; Silfverberg 1992, 2004; Gaidienė 1993; Monsevičius 1997; Pileckis and Monsevičius 1997; Žiogas 1997, 2000a; Gliaudys 2001; Ferenca 2006b.

*saliceti* **(Suffrian, 1849)**. Mazurowa and Mazur 1939; Pileckis 1960, 1976a; Pileckis et al. 1968; Zajančkauskas and Pileckis 1968; Silfverberg 1992, 2004; Gaidienė 1993; Monsevičius 1997; Pileckis and Monsevičius 1997; Ferenca 2006b; Vaivilavičius 2008.

*tremula* **Fabricius, 1787**. Roubal 1910; Pileckis 1960, 1976a; Lešinskas and Pileckis 1967; Pileckis et al. 1968; Silfverberg 1992, 2004; Gaidienė 1993; Pileckis and Monsevičius 1997; Monsevičius 1997; Žiogas 1997, 2000a; Šablevičius 2000b; Gliaudys 2001; Tamutis and Zolubas 2001; Ferenca 2006b; Vaivilavičius 2008.

*vigintipunctata* **(Scopoli, 1763)**. Heyden 1903; Pileckis 1960, 1963b, 1976a; Silfverberg 1992, 2004; Gaidienė 1993; Monsevičius 1997; Pileckis and Monsevičius 1997; Šablevičius 2000b, 2011; Gliaudys 2001; Žiogas and Zolubas 2005; Vaivilavičius 2008; Ivinskis et al. 2009.

*Plagiosterna* **Motschulsky, 1860** = *Linnaeidea* Motschulsky, 1860.

*aenea* **(Linnaeus, 1758)**. Heyden 1903; Ogyjewicz 1938; Mazurowa and Mazur 1939; Pileckis 1960, 1976a; Lešinskas and Pileckis 1967; Zajančkauskas and Pileckis 1968; Ivinskis et al. 1984; Silfverberg 1992, 2004; Gaidienė 1993; Monsevičius 1997; Pileckis and Monsevičius 1997 (*Linaeidea*); Žiogas 2000a; Šablevičius 2000b; Gliaudys 2001; Ferenca 2006b.

*Gonioctena* **Chevrolat, 1836** = *Phytodecta* Kirby, 1837.

*decemnotata* **(Marsham, 1802)** = *rufipes* (DeGeer, 1775) nec (Linnaeus, 1758). Pileckis 1959, 1960, 1976a; Lešinskas and Pileckis 1967; Silfverberg 1992, 2004; Gaidienė 1993; Monsevičius 1997; Pileckis and Monsevičius 1997; Gliaudys 2001; Vaivilavičius 2008; Audisio 2009.

*flavicornis* **(Suffrian, 1851)**. Pileckis 1960, 1976a; Silfverberg 1992, 2004; Pileckis and Monsevičius 1997; Ferenca 2006b.

*intermedia* **(Helliesen, 1913)**. Bukejs et al. 2011.

*linnaeana* **(Schranck, 1781)**. Pileckis 1960, 1976a; Silfverberg 1992, 2004; Gaidienė 1993; Pileckis and Monsevičius 1997; Šablevičius 2000b; Ferenca 2006b.

[*olivacea* **(Forster, 1771)**]. Known in Denmark (Lundberg and Gustafsson 1995), northwestern Belarus (Alexandrovitch et al. 1996), northern Poland (Burakowski et al. 1990), Kaliningrad region (Audisio 2009).

*pallida* **(Linnaeus, 1758)**. Pileckis 1960, 1976a; Silfverberg 1992, 2004; Gaidienė 1993; Pileckis and Monsevičius 1997; Ferenca 2006b; Audisio 2009; Ostrauskas and Ferenca 2010; Kippenberg 2010.

*quinquepunctata* **(Fabricius, 1787)**. Heyden 1903; Pileckis 1960, 1976a; Silfverberg 1992, 2004; Gaidienė 1993; Pileckis and Monsevičius 1997; Ferenca 2006b; Audisio 2009; Kippenberg 2010; Bukejs et al. 2011.

*viminalis* **(Linnaeus, 1758)**. Roubal 1910; Pileckis 1960, 1976a; Zajančkauskas and Pileckis 1968; Silfverberg 1992, 2004; Gaidienė 1993; Monsevičius 1997; Pileckis and Monsevičius 1997; Šablevičius 2000b, 2011; Gliaudys 2001; Ferenca 2006b; Vaivilavičius 2008; Audisio 2009.

*Phratora* **Chevrolat, 1836** = *Phyllodecta* Kirby, 1837.

*atrovirens* **(Cornelius, 1757)**. Mazurowa and Mazur 1939; Pileckis 1960, 1976a; Silfverberg 1992, 2004; Gaidienė 1993; Monsevičius 1997; Pileckis and Monsevičius 1997; Ferenca 2006b.

*laticollis* **(Suffrian, 1851)**. Mazurowa and Mazur 1939; Pileckis 1960, 1976a; Silfverberg 1992, 2004; Gaidienė 1993; Monsevičius 1997; Pileckis and Monsevičius 1997; Tamutis and Zolubas 2001; Ferenca 2006b; Ostrauskas and Ferenca 2010.

*tibialis* **(Suffrian, 1851)**. Heyden 1903; Mazurowa and Mazur 1939; Pileckis 1960, 1976a; Pileckis et al. 1968; Silfverberg 1992, 2004; Gaidienė 1993; Monsevičius 1997; Pileckis and Monsevičius 1997; Šablevičius 2000b; Ferenca 2006b.

*vitellinae* **(Linnaeus, 1758)**. Heyden 1903; Roubal 1910; Mazurowa and Mazur 1939; Pileckis 1960, 1976a; Pileckis et al. 1968; Zajančkauskas and Pileckis 1968; Silfverberg 1992, 2004; Gaidienė 1993; Monsevičius 1997; Pileckis and Monsevičius 1997; Šablevičius 2000b; Ferenca 2006b; Vaivilavičius 2008.

*vulgatissima* **(Linnaeus, 1758)**. Roubal 1910; Mazurowa and Mazur 1939; Pileckis 1960, 1976a; Lešinskas and Pileckis 1967; Pileckis et al. 1968; Zajančkauskas and Pileckis 1968; Silfverberg 1992, 2004; Gaidienė 1993; Monsevičius 1997; Pileckis and Monsevičius 1997; Šablevičius 2000b; Ferenca 2006b; Vaivilavičius 2008.

*Entomoscelis* **Chevrolat, 1836**.

*adonidis* **(Pallas, 1771)**. Pileckis 1976a; Lopatin 1986; Silfverberg 1992, 2004; Pileckis et al. 1994b; Pileckis and Monsevičius 1997.

**Timarchini Motschulsky, 1860**.

*Timarcha* **Dejean, 1821**.

*goettingensis* **(Linnaeus, 1758)**. # 82. Eichwald 1830 (*Chrysomela coriaria*); Pileckis 1960, 1976a; Pileckis and Monsevičius 1997; Silfverberg 2004; Ferenca 2006b.

**Galerucinae** **Latreille, 1802**.

**Galerucini Latreille, 1802**.

*Galerucella* **Crotch, 1873**.

*calmariensis* **(Linnaeus, 1767)**. Pileckis 1960, 1963b, 1976a; Zajančkauskas and Pileckis 1968; Silfverberg 1992, 2004; Gaidienė 1993; Monsevičius 1997; Pileckis and Monsevičius 1997; Ferenca 2006b; Audisio 2009; Beenen 2010.

*grisescens* **(Joannis, 1865)**. Pileckis 1960, 1976a; Silfverberg 1992, 2004; Gaidienė 1993; Monsevičius 1997; Pileckis and Monsevičius 1997; Ferenca 2006b; Audisio 2009; Beenen 2010.

*lineola* **(Fabricius, 1781)**. Heyden 1903; Mazurowa and Mazur 1939; Pileckis 1960, 1963b, 1976a; Pileckis et al. 1968; Zajančkauskas and Pileckis 1968; Silfverberg 1992, 2004; Gaidienė 1993; Monsevičius 1997; Pileckis and Monsevičius 1997; Šablevičius 2000b; Ferenca 2006b; Audisio 2009; Beenen 2010.

*nymphaeae* **(Linnaeus, 1758)** = *aquatica* (Geoffroy, 1785). # 83.Heyden 1903; Pileckis 1960, 1976a; Zajančkauskas and Pileckis 1968; Silfverberg 1992, 2004; Gaidienė 1993; Monsevičius 1997; Pileckis and Monsevičius 1997; Ferenca 2006b; Audisio 2009; Beenen 2010.

*pusilla* **(Duftschmid, 1825)**. Pileckis 1960, 1976a; Silfverberg 1992, 2004; Gaidienė 1993; Monsevičius 1997; Pileckis and Monsevičius 1997; Audisio 2009; Beenen 2010.

*sagittariae* **(Gyllenhal, 1713)**= *kerstensi* Lohse, 1989.# 83.Silfverberg 1992, 2004; Lundberg and Gustafsson 1995.

*tenella* **(Linnaeus, 1761)**. Mazurowa and Mazur 1939; Pileckis 1960, 1963b, 1976a; Zajančkauskas and Pileckis 1968; Silfverberg 1992, 2004; Gaidienė 1993; Pileckis et al. 1994a; Monsevičius 1997; Pileckis and Monsevičius 1997; Ferenca 2006b; Audisio 2009; Beenen 2010.

*Pyrrhalta* **Joannis, 1865**.

*viburni* **(Paykull, 1799)**. Pileckis 1960, 1976a; Silfverberg 1992, 2004; Gaidienė 1993; Pileckis and Vengeliauskaitė 1996; Pileckis and Monsevičius 1997; Gliaudys 2001; Audisio 2009; Beenen 2010.

*Xanthogaleruca* **Laboissiére, 1934**.

*luteola* **(O.F. Müller, 1766)**. Pileckis 1960, 1976a; Silfverberg 1992, 2004; Pileckis and Monsevičius 1997; Audisio 2009; Beenen 2010.

*Lochmaea* **Weise, 1883**.

*caprea* **(Linnaeus, 1758)**. Heyden 1903; Pileckis 1960, 1976a; Lešinskas and Pileckis 1967; Pileckis et al. 1968; Zajančkauskas and Pileckis 1968; Silfverberg 1992, 2004; Gaidienė 1993; Monsevičius 1997; Pileckis and Monsevičius 1997; Šablevičius 2000b; Gliaudys 2001; Tamutis and Zolubas 2001; Ferenca 2006b; Vaivilavičius 2008; Audisio 2009; Beenen 2010.

*crataegi* **(Forster, 1771)**. Pileckis 1960, 1976a; Silfverberg 1992, 2004; Gaidienė 1993; Pileckis and Monsevičius 1997; Šablevičius 2003a; Audisio 2009; Beenen 2010.

*suturalis* **(Thomson, 1866)**. Monsevičius 1988b, 1997; Silfverberg 1992, 2004; Gaidienė 1993; Pileckis and Monsevičius 1997; Audisio 2009; Beenen 2010.

*Galeruca* **Geoffroy, 1762**.

*interrupta* **(Illiger, 1802)**. Pileckis 1960, 1976a; Silfverberg 1992, 2004; Gaidienė 1993; Pileckis and Monsevičius 1997; Ferenca 2006b; Audisio 2009.

*jucunda* **Faldermann, 1837**.Beenen 2010.

*laticollis* **R.F. Sahlberg, 1838**. Pileckis and Monsevičius 1997; Silfverberg 2004.

[*melanocephala* **Ponza, 1805**]. Known in northwestern Belarus (Alexandrovitch et al. 1996), Denmark, Sweden (Lundberg and Gustafsson 1995).

*pomonae* **(Scopoli, 1763)**. Pileckis 1960, 1976a; Silfverberg 1992, 2004; Gaidienė 1993; Pileckis and Monsevičius 1997; Ferenca 2006b; Audisio 2009; Beenen 2010.

*tanaceti* **(Linnaeus, 1758)**. Heyden 1903; Pileckis 1960, 1976a; Lešinskas and Pileckis 1967; Silfverberg 1992, 2004; Gaidienė 1993; Monsevičius 1997; Pileckis and Monsevičius 1997; Šablevičius 2000b; Gliaudys 2001; Ferenca 2006b; Audisio 2009; Beenen 2010.

**Hylaspini Chapuis, 1875**.

*Sermylassa* **Reitter, 1912**.

*halensis* **(Linnaeus, 1767)**. Pileckis 1976a; Silfverberg 1992, 2004; Gaidienė 1993; Pileckis and Monsevičius 1997; Ferenca et al. 2002; Šablevičius 2003a, 2011; Inokaitis 2004; Audisio 2009.

*Agelastica* **Chevrolat, 1836**.

*alni* **(Linnaeus, 1758)**. Heyden 1903; Ogyjewicz 1938; Pileckis 1960, 1976a; Lešinskas and Pileckis 1967; Zajančkauskas and Pileckis 1968; Pileckis et al. 1968; Silfverberg 1992, 2004; Gaidienė 1993; Monsevičius 1997; Pileckis and Monsevičius 1997; Žiogas 1997, 2000; Šablevičius 2000b; Gliaudys 2001; Ferenca 2006b; Audisio 2009; Beenen 2010.

**Luperini Gistel, 1848**.

*Phyllobrotica* **Chevrolat, 1836**.

*quadrimaculata* **(Linnaeus, 1758)**. Pileckis 1960, 1976a; Silfverberg 1992, 2004; Gaidienė 1993; Monsevičius 1997; Pileckis and Monsevičius 1997; Šablevičius 2000b; Ferenca et al. 2002; Ferenca 2006b; Audisio 2009; Beenen 2010.

*Calomicrus* **Dillwyn, 1829**.

*pinicola* **(Duftschmid, 1825)**. Pileckis and Monsevičius 1982, 1997; Lopatin 1986; Silfverberg 1992, 2004; Gaidienė 1993; Monsevičius 1997; Audisio 2009; Beenen 2010.

*Luperus* **Geoffroy, 1762**.

*flavipes* **(Linnaeus, 1758)**. Roubal 1910; Pileckis 1960, 1976a; Pileckis et al. 1968; Zajančkauskas and Pileckis 1968; Mensonienė 1981; Silfverberg 1992, 2004; Gaidienė 1993; Monsevičius 1997; Pileckis and Monsevičius 1997; Šablevičius 2000b; Ferenca 2006b; Beenen 2010.

*longicornis* **(Fabricius, 1781)**. Pileckis 1959, 1960, 1976a; Silfverberg 1992, 2004; Pileckis and Monsevičius 1997; Beenen 2010.

*luperus* **(Sulzer, 1776)**. Pileckis 1960, 1976a; Silfverberg 1992, 2004; Gaidienė 1993; Pileckis and Monsevičius 1997; Beenen 2010.

**Alticini Newman, 1834**.

*Phyllotreta* **Chevrolat, 1836**.

*armoraciae* **(Koch, 1803).** Mazurowa and Mazur 1939; Pileckis 1960, 1976a; Silfverberg 1992, 2004; Pileckis and Monsevičius 1997; Gruev and Döberl 1997; Šablevičius 2000b; Gliaudys 2001; Audisio 2009; Ostrauskas and Ferenca 2010.

**[***astrachanica* **Lopatin, 1977].**Recently discovered in Latvia (Bukejs 2011), known in southwestern Belarus (Lopatin and Nesterova 2005), southeastern Sweden (Wanntorp 2005), Poland (Doberl 2010).

*atra* **(Fabricius, 1775)**. Kamiński 1936; Pileckis 1960, 1976a; Putele 1972; Pileckis and Vengeliauskaitė 1977, 1996; Silfverberg 1992, 2004; Gaidienė 1993; Pileckis et al. 1994b; Monsevičius 1997; Pileckis and Monsevičius 1997; Gruev and Döberl 1997; Gliaudys 2001; Ferenca 2006b; Audisio 2009; Ostrauskas and Ferenca 2010; Döberl 2010.

*cruciferae* **(Goeze, 1777)**. Kamiński 1936; Pileckis 1960, 1976a; Silfverberg 1992, 2004; Pileckis and Monsevičius 1997; Gruev and Döberl 1997; Audisio 2009; Döberl 2010.

*dilatata* **Thomson, 1866**. Bukejs et al. 2011.

*exclamationis* **(Thunberg, 1784)**. Pileckis 1960, 1976a; Silfverberg 1992, 2004; Monsevičius 1997; Pileckis and Monsevičius 1997; Gruev and Döberl 1997; Audisio 2009.

*flexuosa* **(Illiger, 1794)**. Kamiński 1936; Pileckis 1960, 1976a; Silfverberg 1992, 2004; Gaidienė 1993; Monsevičius 1997; Pileckis and Monsevičius 1997; Gruev and Döberl 1997; Ferenca 2006b Audisio 2009; Döberl 2010.

*nemorum* **(Linnaeus, 1758)**. Heyden 1903; Ogijewicz 1929, 1931; Kamiński 1936; Pileckis 1960, 1963b, 1976a; Lešinskas and Pileckis 1967; Putele 1972; Pileckis and Vengeliauskaitė 1977, 1996; Pileckis et al. 1983, 1994b; Silfverberg 1992, 2004; Gaidienė 1993; Monsevičius 1997; Pileckis and Monsevičius 1997; Gruev and Döberl 1997; Gliaudys 2001; Tamutis and Zolubas 2001; Šurkus and Gaurilčikienė 2002; Ferenca 2006b; Audisio 2009; Döberl 2010.

*nigripes* **(Fabricius, 1775)**. Kamiński 1936; Pileckis 1960, 1976a; Lešinskas and Pileckis 1967; Silfverberg 1992, 2004; Pileckis et al. 1994b; Pileckis and Monsevičius 1997; Gruev and Döberl 1997; Šurkus and Gaurilčikienė 2002; Audisio 2009; Döberl 2010.

*ochripes* **(Curtis, 1837)**. Kamiński 1936; Pileckis 1960, 1976a; Silfverberg 1992, 2004; Pileckis and Monsevičius 1997; Gruev and Döberl 1997; Audisio 2009; Döberl 2010.

*striolata* **(Fabricius, 1803)** = *vittata* auct. nec (Fabricius, 1775). Kamiński 1936; Mazurowa and Mazur 1939; Pileckis 1960, 1976a; Lešinskas and Pileckis 1967; Putele 1972; Pileckis and Vengeliauskaitė 1977, 1996; Pileckis et al. 1983; Silfverberg 1992, 2004; Gaidienė 1993; Monsevičius 1997; Pileckis and Monsevičius 1997; Gruev and Döberl 1997; Šablevičius 2000b; Gliaudys 2001; Šurkus and Gaurilčikienė 2002; Ferenca 2006b; Audisio 2009; Ostrauskas and Ferenca 2010; Döberl 2010.

*tetrastigma* **(Comoli, 1837)**. Kamiński 1936; Pileckis 1960, 1976a; Silfverberg 1992, 2004; Gaidienė 1993; Pileckis and Monsevičius 1997; Gruev and Döberl 1997; Audisio 2009; Döberl 2010.

*undulata* **Kutschera, 1860**. Heyden 1903; Roubal 1910; Ogijewicz 1929, 1931; Kamiński 1936; Pileckis 1960, 1976a; Lešinskas and Pileckis 1967; Pileckis and Zajančkauskas 1968; Putele 1972; Pileckis and Vengeliauskaitė 1977, 1996; Pileckis et al. 1983, 1994b; Silfverberg 1992, 2004; Gaidienė 1993; Monsevičius 1997; Pileckis and Monsevičius 1997; Gruev and Döberl 1997; Šablevičius 2000b; Šurkus and Gaurilčikienė 2002; Ferenca 2006b; Audisio 2009; Ostrauskas and Ferenca 2010; Döberl 2010.

*vittula* **(Redtenbacher, 1849)**. Pileckis 1960, 1976a; Lešinskas and Pileckis 1967; Putele 1972; Pileckis and Vengeliauskaitė 1977, 1996; ; Silfverberg 1992, 2004; Gaidienė 1993; Pileckis et al. 1994b; Monsevičius 1997; Pileckis and Monsevičius 1997; Gruev and Döberl 1997; Tamutis 1999; Ferenca 2006b; Audisio 2009; Ostrauskas and Ferenca 2010; Döberl 2010.

*Aphthona* **Chevrolat, 1836**.

*abdominalis* **(Duftschmid, 1825)**. Pileckis and Monsevičius 1997; Silfverberg 2004.

*atrocaerulea* **(Stephens, 1831)** = *cyanella* (Redtenbacher, 1849). Heyden 1903; Pileckis 1960, 1976a; Silfverberg 1992, 2004; Gruev and Döberl 1997; Pileckis and Monsevičius 1997; Audisio 2009.

[*cyparissiae* **(Koch, 1803)**]. Known in northern Poland (Burakowski et al. 1991).

[*czwalinai* **Weise, 1888**]. Recently discovered in Kaliningrad region (Alekseev and Bukejs 2011), known in northern Poland (Burakowski et al. 1991).

*erichsoni* **(Zetterstedt, 1838)**. Monsevičius 1988b, 1997; Silfverberg 1992, 2004; Pileckis and Monsevičius 1997; Gruev and Döberl 1997; Audisio 2009; Ivinskis et al. 2009; Döberl 2010.

*euphorbiae* **(Schrank, 1781)**. Kamiński 1936; Mazurowa and Mazur 1939; Pileckis 1960, 1976a; Zajančkauskas and Pileckis 1968; Putele 1972; Pileckis and Vengeliauskaitė 1977, 1996; Silfverberg 1992, 2004; Gaidienė 1993; Pileckis et al. 1994b; Monsevičius 1997; Pileckis and Monsevičius 1997; Gruev and Döberl 1997; Šurkus and Gaurilčikienė 2002; Ferenca 2006b; Audisio 2009; Döberl 2010.

*flaviceps **Allard, 1859**. # 84.Šurkus and Gaurilčikienė 2002.

*lutescens* **(Gyllenhal, 1813)**. Kamiński 1936; Pileckis 1960, 1976a; Putele 1972; Silfverberg 1992, 2004; Monsevičius 1997; Pileckis and Monsevičius 1997; Gruev and Döberl 1997; Audisio 2009; Döberl 2010.

*nonstriata* **(Goeze, 1777)** = *coerulea* (Geoffroy, 1785). Kamiński 1936; Pileckis 1960, 1976a; Putele 1972; Silfverberg 1992, 2004; Gaidienė 1993; Monsevičius 1997; Pileckis and Monsevičius 1997; Gruev and Döberl 1997; Ferenca 2006b; Vaivilavičius 2008; Audisio 2009; Ivinskis et al. 2009; Döberl 2010.

*pallida* **(Bach, 1856)**. Kamiński 1936; Pileckis 1960, 1976a; Putele 1972; Silfverberg 1992, 2004; Pileckis and Monsevičius 1997; Gruev and Döberl 1997; Audisio 2009; Döberl 2010.

*pygmaea* **Kutschera, 1861**. Roubal 1910; Pileckis 1960, 1976a; Silfverberg 1992, 2004; Gaidienė 1993; Pileckis and Monsevičius 1997; Gruev and Döberl 1997; Ferenca 2006b; Audisio 2009; Döberl 2010.

*venustula* **Kutschera, 1861**. Kamiński 1936; Pileckis 1960, 1976a; Silfverberg 1992, 2004; Gaidienė 1993; Monsevičius 1997; Pileckis and Monsevičius 1997; Gruev and Döberl 1997; Tamutis 1999; Ferenca 2006b; Audisio 2009; Döberl 2010.

*violacea* **(Koch, 1803)**. Pileckis and Monsevičius 1997; Silfverberg 2004.

*Longitarsus* **Berthold, 1827**.

[*absynthii* **Kutschera, 1862**]. Known in Latvia (Telnov 2004), Sweden (Lundberg and Gustafsson 1995), Poland (Burakowski et al. 1991).

*aeneicollis* **(Faldermann, 1837)** = *suturalis* (Marsham, 1802) nec (Fabricius, 1775). Putele 1972; Silfverberg 1992, 2004; Pileckis and Monsevičius 1997; Gruev and Döberl 1997; Audisio 2009; Döberl 2010.

*anchusae* **(Paykull, 1799)**. Putele 1972; Pileckis 1976a; Silfverberg 1992, 2004; Pileckis and Monsevičius 1997; Gruev and Döberl 1997; Audisio 2009; Döberl 2010.

[*apicalis* **(Beck, 1817)**]. Known in Latvia (Telnov 2004), Estonia, Denmark, throughout Sweden (Lundberg and Gustafsson 1995), northwestern Belarus (Alexandrovitch et al. 1996), Poland (Burakowski et al. 1991).

*atricillus* **(Linnaeus, 1761)**. Kamiński 1936; Pileckis 1960, 1976a; Putele 1972; Silfverberg 1992, 2004; Monsevičius 1997; Pileckis and Monsevičius 1997; Gruev and Döberl 1997; Audisio 2009; Döberl 2010.

[*ballotae* **(Marsham, 1802)**]. Known in Latvia (Telnov 2004), Poland (Burakowski et al. 1991), Belarus (Audisio 2009).

*brunneus* **(Duftschmid, 1825)**. Putele 1972; Pileckis 1976a; Silfverberg 1992, 2004; Pileckis and Monsevičius 1997; Gruev and Döberl 1997; Audisio 2009; Döberl 2010.

*curtus* **(Allard, 1860)**. Kamiński 1936; Mazurowa and Mazur 1939; Pileckis 1960, 1976a; Putele 1972; Silfverberg 1992, 2004; Gaidienė 1993; Pileckis and Monsevičius 1997; Gruev and Döberl 1997; Audisio 2009; Döberl 2010.

*echii* **(Koch, 1803)**. Ogijewicz 1929, 1931; Pileckis 1960, 1976a; Pileckis and Monsevičius 1997; Gruev and Döberl 1997; Silfverberg 2004; Audisio 2009; Ivinskis et al. 2009; Döberl 2010.

*exsoletus* **(Linnaeus, 1758)**. Putele 1972; Pileckis 1976a; Silfverberg 1992, 2004; Gaidienė 1993; Pileckis and Monsevičius 1997; Gruev and Döberl 1997; Audisio 2009; Döberl 2010.

*ferrugineus* **(Foudras, 1860)** = *waterhousei* (Kutschera, 1864). Kamiński 1936; Mazurowa and Mazur 1939; Pileckis 1960, 1976a; Silfverberg 1992, 2004; Pileckis and Monsevičius 1997; Gruev and Döberl 1997; Audisio 2009; Döberl 2010.

[*fulgens* **(Foudras, 1860)**]. Known in Latvia (Telnov 2004; Bukejs 2010a), Belarus (Alexandrovitch et al. 1996), Poland (Burakowski et al. 1991).

[*fuscoaeneus* **Redtenbacher, 1849**]. Known in northeastern Poland (Burakowski et al. 1991).

*ganglbaueri* **Heikertinger, 1912**. Kamiński 1936; Pileckis 1960, 1976a; Putele 1972; Silfverberg 1992, 2004; Gaidienė 1993; Pileckis and Monsevičius 1997; Gruev and Döberl 1997; Audisio 2009; Döberl 2010.

*gracilis* **Kutschera, 1864**. Kamiński 1936; Pileckis 1960, 1976a; Putele 1972; Silfverberg 1992, 2004; Pileckis and Monsevičius 1997; Gruev and Döberl 1997; Audisio 2009; Döberl 2010.

*holsaticus* **(Linnaeus, 1758)**. Mazurowa and Mazur 1939; Pileckis 1960, 1976a; Silfverberg 1992, 2004; Monsevičius 1997; Pileckis and Monsevičius 1997; Gruev and Döberl 1997; Audisio 2009; Döberl 2010.

*jacobaeae* **(Waterhouse, 1858)**. Kamiński 1936; Mazurowa and Mazur 1939; Pileckis 1960, 1976a; Silfverberg 1992, 2004; Pileckis and Monsevičius 1997; Gruev and Döberl 1997; Audisio 2009; Döberl 2010.

*kutscherai* **(Rye, 1872)**. Tamutis 1999; Silfverberg 2004.

[*lewisii* **(Baly, 1874)**]. # 85. Known in Latvia (Bukejs 2010a), Estonia, Finland (Silfverberg 2004).

*longipennis* **Kutschera, 1864**.Audisio 2009.

*longiseta* **Weise, 1889**. Kamiński 1936; Mazurowa and Mazur 1939; Pileckis 1960, 1976a; Silfverberg 1992, 2004; Pileckis and Monsevičius 1997; Gruev and Döberl 1997; Audisio 2009.

*luridus* **(Scopoli, 1763)**. Kamiński 1936; Mazurowa and Mazur 1939; Pileckis 1960, 1976a; Putele 1972; Silfverberg 1992, 2004; Pileckis and Monsevičius 1997; Gruev and Döberl 1997; Audisio 2009; Döberl 2010.

*lycopi* **(Foudras, 1860)**. Kamiński 1936; Pileckis 1960, 1976a; Putele 1972; Silfverberg 1992, 2004; Pileckis and Monsevičius 1997; Gruev and Döberl 1997; Audisio 2009; Döberl 2010.

[*medvedevi* **Shapiro, 1956**]. Known in southern Sweden (Lundberg and Gustafsson 1995), Poland (Burakowski et al. 1991).

*melanocephalus* **(DeGeer, 1775)**. Kamiński 1936; Mazurowa and Mazur 1939; Pileckis 1960, 1976a; Putele 1972; Silfverberg 1992, 2004; Pileckis and Monsevičius 1997; Gruev and Döberl 1997; Audisio 2009; Döberl 2010.

*minusculus **(Foudras, 1860)**. Putele 1972; Pileckis 1976a; disproved by Pileckis and Monsevičius (1997).

*nasturtii* **(Fabricius, 1792)**. Kamiński 1936; Pileckis 1960, 1976a; Putele 1972; Silfverberg 1992, 2004; Pileckis and Monsevičius 1997; Gruev and Döberl 1997; Audisio 2009; Döberl 2010.

*niger* **(Koch, 1803)**. Gaidienė 1993; Silfverberg 2004.

*nigerrimus* **(Gyllenhal, 1827)**. Mazurowa and Mazur 1939; Pileckis 1960, 1976a; Silfverberg 1992, 2004; Monsevičius 1997; Pileckis and Monsevičius 1997; Gruev and Döberl 1997; Audisio 2009; Ivinskis et al. 2009; Döberl 2010.

[*nigrofasciatus* **(Goeze, 1777)**]. Known in Latvia (Telnov 2004), Belarus (Alexandrovitch et al. 1996), northern Poland (Burakowski et al. 1991).

[*noricus* **Leonardi, 1976**]. Known in northern Poland (Burakowski et al. 1991), Latvia (Bukejs 2010a).

*obliteratus* **(Rosenhauer, 1847)**. Putele 1972; Pileckis 1976a; Pileckis and Monsevičius 1997; Gruev and Döberl 1997; Silfverberg 2004; Döberl 2010.

[*ochroleucus* **(Marsham, 1802)**]. Known in Latvia (Telnov 2004), Denmark, southern Sweden (Lundberg and Gustafsson 1995), northern Poland (Burakowski et al. 1991).

*parvulus* **(Paykull, 1799)**. Kamiński 1936; Mazurowa and Mazur 1939; Pileckis 1960, 1976a; Lešinskas and Pileckis 1967; Zajančkauskas and Pileckis 1968; Putele 1972; Pileckis and Vengeliauskaitė 1977, 1996; Silfverberg 1992, 2004; Pileckis et al. 1994b; Monsevičius 1997; Pileckis and Monsevičius 1997; Gruev and Döberl 1997; Šurkus and Gaurilčikienė 2002; Ferenca 2006b; Audisio 2009; Döberl 2010.

*pellucidus* **(Foudras, 1860)**. Kamiński 1936; Mazurowa and Mazur 1939; Pileckis 1960, 1976a; Putele 1972; Silfverberg 1992, 2004; Pileckis and Monsevičius 1997; Gruev and Döberl 1997; Audisio 2009; Döberl 2010.

[*plantagomaritimus* **Dollman, 1912**]. Known in Denmark, southern Sweden (Lundberg and Gustafsson 1995).

*pratensis* **(Panzer, 1974)**. Kamiński 1936; Mazurowa and Mazur 1939; Pileckis 1960, 1976a; Putele 1972; Silfverberg 1992, 2004; Pileckis and Monsevičius 1997; Gruev and Döberl 1997; Audisio 2009; Döberl 2010.

[*pulmonaria* **Weise, 1893**]. Known in Denmark (Silfverberg 2004), Poland (Burakowski et al. 1991).

[*quadriguttatus* **(Pontoppidan, 1765)**]. Known in Estonia, Denmark, southern Sweden (Lundberg and Gustafsson 1995), northwestern Belarus (Alexandrovitch et al. 1996), northern Poland (Burakowski et al. 1991).

[*reichei* **(Allard, 1860)**]. Known in Denmark, southern Sweden (Lundberg and Gustafsson 1995).

*rubiginosus* **(Foudras, 1860)**. Kamiński 1936; Mazurowa and Mazur 1939; Pileckis 1960, 1976a; Putele 1972; Silfverberg 1992, 2004; Pileckis and Monsevičius 1997; Gruev and Döberl 1997; Audisio 2009; Döberl 2010.

*succineus* **(Foudras, 1860)**. Kamiński 1936; Mazurowa and Mazur 1939; Pileckis 1960, 1976a; Putele 1972; Silfverberg 1992, 2004; Gaidienė 1993; Pileckis and Monsevičius 1997; Gruev and Döberl 1997; Döberl 2010.

*suturellus* **(Duftschmid, 1825)**. Putele 1972; Pileckis 1976a; Silfverberg 1992, 2004; Pileckis and Monsevičius 1997; Gruev and Döberl 1997; Audisio 2009; Döberl 2010.

*symphyti* **Heikertinger, 1912**. Putele 1972; Pileckis 1976a; Silfverberg 1992, 2004; Pileckis and Monsevičius 1997; Gruev and Döberl 1997; Audisio 2009; Döberl 2010.

*tabidus* **(Fabricius, 1775)**. Kamiński 1936; Pileckis 1960, 1976a; Silfverberg 1992, 2004; Gaidienė 1993; Pileckis and Monsevičius 1997; Gruev and Döberl 1997; Ferenca et al. 2006, 2007; Audisio 2009; Döberl 2010.

*Altica* **Geoffroy, 1762**.

*aenescens* **Weise, 1888**. Silfverberg 1992, 2004; Gruev and Döberl 1997; Pileckis and Monsevičius 1997; Audisio 2009; Döberl 2010.

*brevicollis* **Foudras, 1860**.Audisio 2009; Döberl 2010.

[*carduorum* **(Guérin-Mènèville, 1858)** = *cirsii* Israelson, 1956]. Known in Latvia (Telnov 2004), Sweden (Silfverberg 2004).

*carinthiaca* **Weise, 1888**.Audisio 2009; Bukejs et al. 2011.

*chamaenerii* **H. Lindberg, 1926**. # 86. Putele 1972; Pileckis 1976a; Silfverberg 1992, 2004; Pileckis and Monsevičius 1997; Gruev and Döberl 1997; Audisio 2009.

*engstromi* **J.R. Sahlberg, 1893**. # 86. Pileckis 1959, 1960, 1976a; Zajančkauskas and Pileckis 1968; Putele 1972; Silfverberg 1992, 2004; Monsevičius 1997; Pileckis and Monsevičius 1997; Gruev and Döberl 1997; Audisio 2009.

*longicollis* **(Allard, 1860)** = *britteni* Sharp, 1914 = *sandini* Kemner, 1919]. Known in Latvia (Telnov 2004), Denmark, Estonia, throughout Sweden (Lundberg and Gustafsson 1995), northwestern Belarus (Alexandrovitch et al. 1996), northern Poland (Burakowski et al. 1991), Kaliningrad region (Alekseev 2003).

*helianthemi* **(Allard, 1859)** = *pusilla* Duftschmid, 1825, nec Gyllenhal, 1813. Putele 1972; Pileckis 1976a; Silfverberg 1992, 2004; Pileckis and Monsevičius 1997; Gruev and Döberl 1997; Audisio 2009; Döberl 2010.

[*impressicollis* **(Reiche, 1862)**]. Known in Latvia (Telnov et al. 2007; Bukejs 2011), Poland (Burakowski et al. 1991) recently found in Kaliningrad region (Alekseev and Bukejs 2011) recently found in Kaliningrad region (Alekseev and Bukejs 2011).

*lythri* **Aubé, 1843**. Heyden 1903; Pileckis 1960, 1963b, 1976a; Zajančkauskas and Pileckis 1968; Monsevičius 1997; Pileckis and Monsevičius 1997; Silfverberg 2004.

*oleracea* **(Linnaeus, 1758)**. Roubal 1910; Ivanauskas and Vailionis 1922; Ogijewicz 1931; Kamiński 1936; Mazurowa and Mazur 1939; Pileckis 1960, 1963b, 1976a; Zajančkauskas and Pileckis 1968; Putele 1972; Silfverberg 1992, 2004; Gaidienė 1993; Monsevičius 1997; Pileckis and Monsevičius 1997; Gruev and Döberl 1997; Ferenca 2006b; Audisio 2009; Döberl 2010.

*palustris* **Weise, 1888**. Putele 1972; Pileckis 1976a; Silfverberg 1992, 2004; Pileckis and Monsevičius 1997; Gruev and Döberl 1997; Audisio 2009; Döberl 2010.

*quercetorum saliceti* **Weise, 1888**. Pileckis 1960, 1976a; Lešinskas and Pileckis 1967; Pileckis et al. 1968; Silfverberg 1992, 2004; Gaidienė 1993; Monsevičius 1997; Pileckis and Monsevičius 1997; Gruev and Döberl 1997; Gliaudys 2001; Ferenca 2006b; Audisio 2009; Döberl 2010.

*tamaricis* **Schrank, 1785**. Mazurowa and Mazur 1939; Pileckis 1960, 1976a; Silfverberg 1992, 2004; Gaidienė 1993; Pileckis and Monsevičius 1997; Gruev and Döberl 1997; Ferenca 2006b; Audisio 2009; Döberl 2010.

*Hermaeophaga* **Foudras, 1860**.

*mercurialis* **(Fabricius, 1792)**. Gaidienė 1993; Pileckis and Monsevičius 1997; Silfverberg 2004; Vaivilavičius 2008.

*Batophila* **Foudras, 1860**.

*rubi* **(Paykull, 1799)**. Kamiński 1936; Pileckis 1960, 1976a; Putele 1972; Pileckis and Vengeliauskaitė 1977, 1996; Silfverberg 1992, 2004; Gaidienė 1993; Monsevičius 1997; Pileckis and Monsevičius 1997; Gruev and Döberl 1997; Ferenca 2006b; Audisio 2009; Döberl 2010.

*Lythraria* **Bedel, 1897**.

*salicariae* **(Paykull, 1800)**. Kamiński 1936; Mazurowa and Mazur 1939; Pileckis 1960, 1976a; Putele 1972; Silfverberg 1992, 2004; Monsevičius 1997; Pileckis and Monsevičius 1997; Gruev and Döberl 1997; Ivinskis et al. 2008; Audisio 2009; Döberl 2010.

*Neocrepidodera* **Heikertinger, 1911** = *Asiorestia* Jacobson, 1925 = *Crepidodera* auct. nec Chevrolat, 1836.

[*brevicollis* **(Daniel, 1904**)]. Known in Denmark, southern Sweden (Lundberg and Gustafsson 1995), Belarus (Alexandrovitch et al. 1996), Poland (Burakowski et al. 1991) ; recently found in Latvia (Telnov et al. 2011).

*femorata* **(Gyllenhal, 1813)**. Pileckis 1960, 1976a; Silfverberg 1992, 2004; Pileckis and Monsevičius 1997; Gruev and Döberl 1997; Audisio 2009.

*ferruginea* **(Scopoli, 1763)**. Kamiński 1936; Mazurowa and Mazur 1939; Pileckis 1960, 1963b, 1976a; Putele 1972; Silfverberg 1992, 2004; Gaidienė 1993; Monsevičius 1997; Pileckis and Monsevičius 1997; Vaivilavičius 2008; Audisio 2009; Ivinskis et al. 2009; Ostrauskas and Ferenca 2010; Döberl 2010.

*interpunctata* **(Motschulsky, 1859)**. Pileckis1968b, 1976a; Zajančkauskas and Pileckis 1968; Gaidienė 1993; Monsevičius 1997; Pileckis and Monsevičius 1997; Silfverberg 2004.

*motschulskii* **(Konstantinov, 1991)** = *sublaevis* auct. nec (Motschulsky, 1859). Mazurowa and Mazur 1939; Pileckis 1960, 1976a; Silfverberg 1992, 2004; Monsevičius 1997; Pileckis and Monsevičius 1997; Audisio 2009; Döberl 2010.

*nigritula* **(Gyllenhal, 1813)**. Monsevičius 1988b; Silfverberg 1992, 2004; Gaidienė 1993; Monsevičius 1997; Pileckis and Monsevičius 1997; Gruev and Döberl 1997; Audisio 2009; Ivinskis et al. 2009; Döberl 2010.

*transversa* **(Marsham, 1802)**. Pileckis and Monsevičius1982, 1997; Silfverberg 1992, 2004; Gaidienė 1993; Monsevičius 1997; Gruev and Döberl 1997; Šablevičius 2000b; Audisio 2009; Döberl 2010.

*Derocrepis* **Weise, 1886**.

*rufipes* **(Linnaeus, 1758)**. Ferenca et al. 2002, 2006, 2007; Šablevičius 2003a; Silfverberg 2004; Vaivilavičius 2008.

*Hippuriphila* **Foudras, 1860**.

*modeeri* **(Linnaeus, 1761)** = *laevicollis* (Hellén, 1933). Kamiński 1936; Mazurowa and Mazur 1939; Pileckis 1960, 1976a; Putele 1972; Silfverberg 1992, 2004; Gaidienė 1993; Monsevičius 1997; Pileckis and Monsevičius 1997; Gruev and Döberl 1997; Ferenca 2006b; Audisio 2009; Döberl 2010.

*Crepidodera* **Chevrolat, 1836** = *Chalcoides* Foudras, 1860.

*aurata* **(Marsham, 1802)**. Heyden 1903; Kamiński 1936; Ogyjewicz 1938; Pileckis 1960, 1976a; Lešinskas and Pileckis 1967; Zajančkauskas and Pileckis 1968; Putele 1972; Silfverberg 1992, 2004; Gaidienė 1993; Monsevičius 1997; Pileckis and Monsevičius 1997; Gruev and Döberl 1997; Šablevičius 2000b; Ferenca 2006b; Vaivilavičius 2008; Audisio 2009; Ostrauskas and Ferenca 2010; Döberl 2010.

*aurea* **(Geoffroy, 1785)**. Pileckis 1960, 1976a; Silfverberg 1992, 2004; Gaidienė 1993; Pileckis and Monsevičius 1997; Gruev and Döberl 1997; Žiogas and Zolubas 2005; Ferenca 2006b; Audisio 2009; Döberl 2010.

*fulvicornis* **(Fabricius, 1792)** = *lapponica* (Heikertinger, 1950). Heyden 1903; Kamiński 1936; Mazurowa and Mazur 1939; Pileckis 1960, 1976a; Pileckis et al. 1968; Zajančkauskas and Pileckis 1968; Putele 1972; Silfverberg 1992, 2004; Gaidienė 1993; Monsevičius 1997; Pileckis and Monsevičius 1997; Gruev and Döberl 1997; Šablevičius 2000b; Ferenca 2006b; Vaivilavičius 2008; Audisio 2009; Döberl 2010.

*nitidula* **(Linnaeus, 1758)**. Pileckis 1960, 1976a; Silfverberg 1992, 2004; Gaidienė 1993; Monsevičius 1997; Pileckis and Monsevičius 1997; Gruev and Döberl 1997; Šablevičius 2000b; Tamutis and Zolubas 2001; Ferenca 2006b; Audisio 2009; Ostrauskas and Ferenca 2010; Döberl 2010.

*plutus* **(Latreille, 1804)**. Tamutis and Ferenca 2006; Ferenca et al. 2006, 2007.

*Epitrix* **Foudras, 1860**.

*pubescens* **(Koch, 1803)**. Kamiński 1936; Pileckis 1960, 1976a; Silfverberg 1992, 2004; Monsevičius 1997; Pileckis and Monsevičius 1997; Gruev and Döberl 1997; Audisio 2009; Döberl 2010.

*cucumeris **(Harris, 1851)**. # 87.Pileckis and Vengeliauskaitė 1996.

*Podagrica* **Chevrolat, 1836**.

[*fuscicornis* **(Linnaeus, 1767)**]. Known in Denmark (Lundberg and Gustafsson 1995), northern Poland (Burakowski et al. 1991), Latvia (Audisio 2009).

*Mantura* **Stephens, 1831**.

*chrysanthemi* **(Koch, 1803)**. Kamiński 1936; Mazurowa and Mazur 1939; Pileckis 1960, 1963b, 1976a; Silfverberg 1992, 2004; Gaidienė 1993; Monsevičius 1997; Pileckis and Monsevičius 1997; Gruev and Döberl 1997; Audisio 2009; Döberl 2010.

[*obtusata* **(Gyllenhal, 1813)** = *ambigua* Kutschera, 1862]. Known in Latvia (Telnov 2004), Denmark, Estonia, Sweden (Lundberg and Gustafsson 1995), northern Poland (Burakowski et al. 1991).

*rustica* **(Linnaeus, 1767)**. Roubal 1910; Kamiński 1936; Pileckis 1960, 1976a; Silfverberg 1992, 2004; Gaidienė 1993; Pileckis and Monsevičius 1997; Gruev and Döberl 1997; Audisio 2009; Döberl 2010.

*Chaetocnema* **Stephens, 1831**.

[*aerosa* **(Letzner, 1846)**]. Known in Latvia (Telnov 2004), Estonia, throughout Sweden (Lundberg and Gustafsson 1995), northwestern Belarus (Alexandrovitch et al. 1996), Poland (Burakowski et al. 1991).

*arida* **Foudras, 1860**. Mazurowa and Mazur 1939; Pileckis 1960, 1976a; Pileckis and Monsevičius 1997; Silfverberg 2004.

*aridula* **(Gyllenhal, 1827)**. Kamiński 1936; Mazurowa and Mazur 1939; Pileckis 1960, 1976a; Lešinskas and Pileckis 1967; Putele 1972; Pileckis and Vengeliauskaitė 1977, 1996; Dabkevičius 1984; Silfverberg 1992, 2004; Gaidienė 1993; Pileckis et al. 1994b; Monsevičius 1997; Pileckis and Monsevičius 1997; Gruev and Döberl 1997; Ferenca 2006b; Audisio 2009; Döberl 2010.

*breviuscula* **(Faldermann, 1837)**. Pileckis and Monsevičius 1997; Silfverberg 2004.

[*compressa* **(Letzner, 1864)**]. Recently found in Kaliningrad region (Alekseev and Bukejs 2010), known in Poland (Burakowski et al. 1991).

*concinna* **(Marsham, 1802)**. Kamiński 1936; Mazurowa and Mazur 1939; Pileckis 1960, 1976a; Lešinskas and Pileckis 1967; Putele 1972; Pileckis and Vengeliauskaitė 1977, 1996; Pileckis et al. 1983, 1994b; Silfverberg 1992, 2004; Gaidienė 1993; Monsevičius 1997; Pileckis and Monsevičius 1997; Gruev and Döberl 1997; Šurkus and Gaurilčikienė 2002; Ferenca 2006b; Audisio 2009; Döberl 2010.

[*confusa* **(Bohemann, 1851)**]. Known in Latvia (Telnov 2004), Denmark, southern Sweden (Lundberg and Gustafsson 1995), northern Poland (Burakowski et al. 1991).

*hortensis* **(Geoffroy, 1785)**. Kamiński 1936; Mazurowa and Mazur 1939; Pileckis 1960, 1976a; Putele 1972; Pileckis and Vengeliauskaitė 1977; Dabkevičius 1984; Silfverberg 1992, 2004; Gaidienė 1993; Monsevičius 1997; Pileckis and Monsevičius 1997; Gruev and Döberl 1997; Audisio 2009; Döberl 2010.

*mannerheimii* **(Gyllenhal, 1827)**. Silfverberg 2004; Ivinskis et al. 2009.

[*obesa* **(Boieldieu, 1859)**]. Known in Latvia (Telnov 2004), Poland (Burakowski et al. 1991), Belarus (Alexandrovitch et al. 1996).

*picipes* **Stephens, 1831** = *laevicollis* (Thomson, 1866) = *heikertingeri* Lubischev, 1963. Pileckis and Monsevičius1982, 1997; Silfverberg 1992, 2004; Monsevičius 1997; Gruev and Döberl 1997; Audisio 2009; Döberl 2010.

*sahlbergii* **(Gyllenhal, 1827)**. Mazurowa and Mazur 1939; Pileckis 1960, 1976a; Silfverberg 1992, 2004; Monsevičius 1997; Pileckis and Monsevičius 1997; Gruev and Döberl 1997; Audisio 2009; Ivinskis et al. 2009; Döberl 2010.

*semicoerulea* **(Koch, 1803)**. Kamiński 1936; Mazurowa and Mazur 1939; Pileckis 1960, 1976a; Silfverberg 1992, 2004; Gaidienė 1993; Pileckis and Monsevičius 1997; Gruev and Döberl 1997; Ferenca 2006b; Döberl 2010.

*subcoerulea* **(Kutschera, 1864)**.Audisio 2009; Döberl 2010.

[*tibialis* **(Illiger, 1807)**]. Known in Latvia (Telnov 2004), Poland (Burakowski et al. 1991); recently found in Kaliningrad region (Alekseev and Bukejs 2011).

*Dibolia* **Latreille, 1829**.

*cryptocephala* **(Koch, 1803)**. Pileckis 1960, 1976a; Silfverberg 1992, 2004; Pileckis and Monsevičius 1997; Gruev and Döberl 1997; Ferenca 2006b; Audisio 2009; Döberl 2010.

[*cynoglossi* **(Koch, 1803)**]. Known in Latvia (Telnov 2004), Estonia, Denmark (Lundberg and Gustafsson 1995), northeastern Poland (Burakowski et al. 1991), Kaliningrad region (Alekseev 2003).

*depressiuscula* **Letzner, 1846**. Kamiński 1936; Mazurowa and Mazur 1939; Pileckis 1960, 1976a; Silfverberg 1992, 2004; Pileckis and Monsevičius 1997; Gruev and Döberl 1997; Audisio 2009; Döberl 2010.

[*foersteri* **Bach, 1859**]. Known in eastern Belarus (Alexandrovitch et al. 1996), Poland (Burakowski et al. 1991).

*occultans* **(Koch, 1803)**. Ferenca 2003.

*Sphaeroderma* **Stephens, 1831**.

*rubidum* **(Graëlis, 1858)**. Pileckis and Monsevičius 1982, 1997; Lopatin 1986; Silfverberg 1992, 2004; Gaidienė 1993; Gruev and Döberl 1997; Audisio 2009; Döberl 2010.

*testaceum* **(Fabricius, 1775)**. Warchałowski 1978; Gaidienė 1993; Monsevičius 1997; Pileckis and Monsevičius 1997; Šablevičius 2003a; Silfverberg 2004; Ivinskis et al. 2009.

*Argopus* **Fischer von Waldheim, 1824**.

[*ahrensi* **(Germar, 1817)**]. Known in northeastern Poland (Burakowski et al. 1991), doubtful in Latvia (Bukejs 2009b).

[*nigritarsis* **(Gebler, 1823)**]. Known in Latvia (Bukejs 2008), northeastern Poland (Burakowski et al. 1991).

*Mniophila* **Stephens, 1831**.

*muscorum* **(Koch, 1803)**. Warchałowski 1978; Pileckis and Monsevičius 1997; Silfverberg 2004.

*Psylliodes* **Berthold, 1827**.

*affinis* **(Paykull, 1799)**. Kamiński 1936; Pileckis 1960, 1976a; Putele 1972; Pileckis et al. 1983, 1994b; Silfverberg 1992, 2004; Gaidienė 1993; Pileckis and Vengeliauskaitė 1996; Monsevičius 1997; Pileckis and Monsevičius 1997; Gruev and Döberl 1997; Gliaudys 2001; Ferenca 2006b; Audisio 2009; Döberl 2010.

*attenuata* **(Koch, 1803)**. Lešinskas and Pileckis 1967; Pileckis 1976a; Pileckis et al. 1994b; Silfverberg 1992, 2004; Pileckis and Monsevičius 1997; Gruev and Döberl 1997; Audisio 2009; Döberl 2010.

*chalcomera* **(Illiger, 1807)**. Kamiński 1936; Pileckis 1960, 1976a; Silfverberg 1992, 2004; Pileckis and Monsevičius 1997; Gruev and Döberl 1997; Audisio 2009; Döberl 2010.

*chrysocephala* **(Linnaeus, 1758)**. Kamiński 1936; Pileckis 1960, 1976a; Silfverberg 1992, 2004; Pileckis et al. 1994b; Pileckis and Monsevičius 1997; Gruev and Döberl 1997; Audisio 2009; Döberl 2010.

[*crambicola* **Lohse, 1954**]. Known in Denmark, southern Sweden (Lundberg and Gustafsson 1995).

*cucullata* **(Illiger, 1807)**. Kamiński 1936; Mazurowa and Mazur 1939; Pileckis 1960, 1976a; Putele 1972; Silfverberg 1992, 2004; Monsevičius 1997; Pileckis and Monsevičius 1997; Gruev and Döberl 1997; Audisio 2009; Ostrauskas and Ferenca 2010; Döberl 2010.

*cuprea* **(Koch, 1803)**. Kamiński 1936; Pileckis 1960, 1963b, 1976a; Silfverberg 1992, 2004; Pileckis and Monsevičius 1997; Gruev and Döberl 1997; Audisio 2009; Döberl 2010.

*cupreata* **(Duftschmid, 1825)**. Kamiński 1936; Pileckis 1960, 1976a; Pileckis and Monsevičius 1997; Gruev and Döberl 1997; Silfverberg 2004; Audisio 2009; Döberl 2010.

*dulcamarae* **(Koch, 1803)**. Kamiński 1936; Pileckis 1960, 1976a; Pileckis and Monsevičius1982, 1997; Silfverberg 1992, 2004; Gaidienė 1993; Monsevičius 1997; Gruev and Döberl 1997; Ferenca 2006b; Vaivilavičius 2008; Audisio 2009; Döberl 2010.

*hyoscyami* **(Linnaeus, 1758)**. Kamiński 1936; Pileckis 1960, 1976a; Silfverberg 1992, 2004; Pileckis and Monsevičius 1997; Gruev and Döberl 1997; Audisio 2009; Döberl 2010.

[*isatidis* **Heikertinger, 1912**]. Known in southern Sweden (Lundberg and Gustafsson 1995).

[*luteola* **(O.F. Müller, 1776)**]. Known in Latvia (Telnov 2004), Estonia, Denmark (Silfverberg 2004), northern Poland (Burakowski et al. 1991). 

*marcida* **(Illiger, 1807)**. Łomnicki 1913; Pileckis and Monsevičius 1997; Silfverberg 2004; Bukejs et al. 2011.

*napi* **(Fabricius, 1792)**. Kamiński 1936; Pileckis 1960, 1976a; Silfverberg 1992, 2004; Pileckis and Monsevičius 1997; Gruev and Döberl 1997; Audisio 2009; Döberl 2010.

*picina* **(Marsham, 1802)**. Monsevičius 1998; Ferenca et al. 2002.

*tricolor* **Weise, 1888 =** *sophiae* **Heikertinger, 1914.** Bercio and Folwaczny 1979; Silfverberg 1992, 2004; Gruev and Döberl 1997; Audisio 2009.

**Lamprosomatinae** **Lacordaire, 1848**.

**Lamprosomatini Lacordaire, 1848**.

*Oomorphus* **Curtis, 1831**.

[*concolor* **(Sturm, 1807)**]. Known in Latvia (Telnov 2004), Denmark, southern Sweden (Lundberg and Gustafsson 1995), Poland (Burakowski et al. 1990).

**Cryptocephalinae** **Gyllenhal, 1813**.

**Clytrini Kirby, 1837**.

*Labidostomis* **Germar, 1817**.

*cyanicornis* **Germar, 1822**. Regalin and Medvedev 2010.

*humeralis* **(Schneider, 1792)**. Pileckis 1968a, 1976a; Silfverberg 1992, 2004; Pileckis and Monsevičius 1997; Audisio 2009.

*lepida* **Lefèvre, 1872**.Audisio 2009.

*longimana* **(Linnaeus, 1761)**. Eichwald 1830; Pileckis 1960, 1963b, 1976a; Silfverberg 1992, 2004; Gaidienė 1993; Pileckis and Monsevičius 1997; Šablevičius 2000b; Audisio 2009; Regalin and Medvedev 2010.

*tridentata* **(Linnaeus, 1758)**. Pileckis 1968a, 1976a; Silfverberg 1992, 2004; Pileckis and Monsevičius 1997; Audisio 2009; Regalin and Medvedev 2010.

*Clytra* **Laicharting, 1781**.

[*laeviuscula* **Ratzeburg, 1837**]. Known in Latvia (Telnov 2004), Estonia (Lundberg and Gustafsson 1995) and northern Poland (Burakowski et al. 1990).

*quadripunctata* **(Linnaeus, 1758)**. Pileckis 1960, 1976a; Lešinskas and Pileckis 1967; Silfverberg 1992, 2004; Gaidienė 1993; Monsevičius 1997; Pileckis and Monsevičius 1997; Šablevičius 2000b, 2011; Gliaudys 2001; Ferenca 2006b; Audisio 2009; Regalin and Medvedev 2010.

*Smaragdina* **Chevrolat, 1836** = *Gynandrophthalma* Lacordaire, 1848.

[*affinis* **(Illiger, 1794)**]. Known in Kaliningrad region (Alekseev 2003), Belarus (Alexandrovitch et al. 1996), Estonia (Süda 2009), northern Poland (Burakowski et al. 1990).

[*aurita* **(Linnaeus, 1767)**]. Known in Denmark, southern Sweden (Lundberg and Gustafsson 1995), northern Poland (Burakowski et al. 1990).

*flavicollis* **(Charpentier, 1825)**. Pileckis 1960, 1976a; Silfverberg 1992, 2004; Pileckis and Monsevičius 1997; Šablevičius 2003a; Audisio 2009; Ivinskis et al. 2009; Regalin and Medvedev 2010.

*salicina* **(Scopoli, 1763)** = *cyanea* (Fabricius, 1775). Pileckis 1959, 1960, 1976a; Silfverberg 1992, 2004; Gaidienė 1993; Monsevičius 1997; Pileckis and Monsevičius 1997; Šablevičius 2000b; Audisio 2009; Regalin and Medvedev 2010.

*Coptocephala* **Chevrolat, 1836**.

[*rubicunda rossica* **(L.Medvedev, 1977)**]. Known in northern Belarus (Alexandrovitch et al. 1996).

*unifasciata* **(Scopoli, 1763)**. Pileckis 1960, 1976a; Silfverberg 1992, 2004; Pileckis and Monsevičius 1997; Šablevičius 2000b; Šablevičius 2003a, 2011; Inokaitis 2004; Audisio 2009; Regalin and Medvedev 2010.

**Cryptocephalini Gyllenhal, 1813**.

*Pachybrachis* **Chevrolat, 1836**.

*hieroglyphicus* **(Laicharting, 1781**). Pileckis 1960, 1976a; Silfverberg 1992, 2004; Gaidienė 1993; Pileckis and Monsevičius 1997; Audisio 2009; Šablevičius 2011.

*Cryptocephalus* **Geoffroy, 1762**.

*androgyne* **Marseul 1875** = *caerulescens* R.F. Sahlberg, 1839. Pileckis and Monsevičius 1982, 1997; Lopatin 1986; Silfverberg 1992, 2004; Gaidienė 1993; Monsevičius 1997; Audisio 2009; Lopatin et al. 2010; Bukejs et al. 2011.

*aureolus* **Suffrian, 1847**. Pileckis 1960, 1976a; Silfverberg 1992, 2004; Gaidienė 1993; Pileckis and Monsevičius 1997; Audisio 2009; Lopatin et al. 2010.

[*bameuli* **Duhaldeborde, 1999**]. Known in Belarus (Lopatin and Nesterova 2005), Denmark, southern Sweden (Wanntorp 2009) and Poland (Audisio 2009).

*biguttatus* **(Scopoli, 1763)**. Pileckis 1960, 1963b, 1976a; Lopatin 1986; Silfverberg 1992, 2004; Gaidienė 1993; Pileckis and Monsevičius 1997; Šablevičius 2000b, 2011; Ferenca 2006b; Audisio 2009; Lopatin et al. 2010.

*bilineatus* **(Linnaeus, 1767)**. Pileckis and Monsevičius 1982, 1997; Silfverberg 1992, 2004; Audisio 2009; Lopatin et al. 2010.

*bipunctatus* **(Linnaeus, 1758)**. Mazurowa and Mazur 1939; Pileckis 1960, 1976a; Silfverberg 1992, 2004; Monsevičius 1997; Pileckis and Monsevičius 1997; Šablevičius 2000b, 2004; Audisio 2009; Lopatin et al. 2010.

*chrysopus* **Gmelin, 1790**. Ivinskis et al. 1999.

*cordiger* **(Linnaeus, 1758)**. Silfverberg 1992, 2004; Pileckis and Monsevičius 1997; Audisio 2009; Lopatin et al. 2010.

*coryli* **(Linnaeus, 1758)**. Pileckis 1976a; Silfverberg 1992, 2004; Gaidienė 1993; Pileckis and Monsevičius 1997; Šablevičius 2003a; Audisio 2009; Lopatin et al. 2010.

*decemmaculatus* **(Linnaeus, 1758)** = *bothnicus* (Linnaeus, 1758). Mazurowa and Mazur 1939; Pileckis 1960, 1976a; Silfverberg 1992, 2004; Gaidienė 1993; Monsevičius 1997; Pileckis and Monsevičius 1997; Šablevičius 2000b, 2003a; Audisio 2009; Lopatin et al. 2010.

*distiguendus* **Schneider, 1792**. Pileckis 1988; Silfverberg 1992, 2004; Gaidienė 1993; Pileckis and Monsevičius 1997; Audisio 2009; Lopatin et al. 2010.

*elegantulus* **Gravenhorst, 1807**.Audisio 2009.

*exiguus* **Schneider, 1792**. Šablevičius and Ferenca 1995; Šablevičius 2000b, 2003a; Silfverberg 2004; Audisio 2009.

*flavipes* **Fabricius, 1781**. Pileckis 1960, 1976a; Lopatin 1986; Silfverberg 1992, 2004; Gaidienė 1993; Pileckis and Monsevičius 1997; Šablevičius 2003a, 2004, 2007; Ferenca 2006b; Audisio 2009; Lopatin et al. 2010; Bukejs et al. 2011.

*frenatus* **Laicharting, 1781**. Pileckis 1960, 1976a; Gaidienė 1993; Pileckis and Monsevičius 1997; Silfverberg 2004; Lopatin et al. 2010.

*frontalis* **Marsham, 1802**. Pileckis and Monsevičius 1997; Silfverberg 2004; Audisio 2009; Lopatin et al. 2010.

*fulvus* **(Goeze, 1777)**. Roubal 1910; Mazurowa and Mazur 1939; Pileckis 1960, 1976a; Silfverberg 1992, 2004; Gaidienė 1993; Pileckis and Monsevičius 1997; Monsevičius 1998; Audisio 2009; Lopatin et al. 2010.

*hypochoeridis* **(Linnaeus, 1758)** = *cristula* Dufour, 1843. Pileckis 1960, 1968b, 1976a; Zajančkauskas and Pileckis 1968; Silfverberg 1992, 2004; Gaidienė 1993; Pileckis and Monsevičius 1997; Šablevičius 2000b; Ferenca 2006b.

*janthinus* **Germar, 1824**. Pileckis 1968b, 1976a; Zajančkauskas and Pileckis 1968; Monsevičius 1997; Pileckis and Monsevičius 1997; Silfverberg 2004; Audisio 2009; Lopatin et al. 2010.

*labiatus* **(Linnaeus, 1761)**. Pileckis 1960, 1963b, 1976a; Zajančkauskas and Pileckis 1968; Ivinskis et al. 1984; Silfverberg 1992, 2004; Gaidienė 1993; Monsevičius 1997; Pileckis and Monsevičius 1997; Šablevičius 2000b; Ferenca 2006b; Audisio 2009; Lopatin et al. 2010.

[*laetus* **Fabricius, 1792**]. Known in Kaliningrad region (Alekseev 2003), northwestern Belarus (Alexandrovitch et al. 1996), northern Poland (Burakowski et al. 1990).

*moraei* **(Linnaeus, 1758)**. Mazurowa and Mazur 1939; Pileckis 1960, 1976a; Silfverberg 1992, 2004; Gaidienė 1993; Monsevičius 1997; Pileckis and Monsevičius 1997; Šablevičius 2000b; Ferenca 2006b; Audisio 2009; Lopatin et al. 2010.

*nitidulus* **Fabricius, 1787**. Pileckis 1959, 1960, 1976a; Lopatin 1986; Silfverberg 1992, 2004; Pileckis and Monsevičius 1997; Audisio 2009; Lopatin et al. 2010.

*nitidus* **(Linnaeus, 1758)**. Pileckis 1959, 1960, 1976a; Gaidienė 1993; Pileckis and Monsevičius 1997; Šablevičius 2003a, b; Silfverberg 1992, 2004; Audisio 2009; Lopatin et al. 2010.

*ocellatus* **Drapiez, 1819**. Roubal 1910; Pileckis 1960, 1963b, 1976a; Ivinskis et al. 1984; Silfverberg 1992, 2004; Gaidienė 1993; Pileckis and Monsevičius 1997; Audisio 2009; Lopatin et al. 2010.

*ochroleucus* **Fairmaire, 1859.** Bukejs and Ferenca 2011.

*octacosmus* **Bedel, 1891** = *anticus* Suffrian, 1848.Ferenca 2003; Audisio 2009; Lopatin et al. 2010.

*octopunctatus* **(Scopoli, 1763)**. Pileckis 1960, 1976a; Lešinskas and Pileckis 1967; Silfverberg 1992, 2004; Gaidienė 1993; Monsevičius 1997; Pileckis and Monsevičius 1997; Šablevičius 2000b, 2011; Gliaudys 2001; Ferenca 2006b; Audisio 2009; Lopatin et al. 2010.

*pallifrons* **Gyllenhal, 1813**. Pileckis and Monsevičius 1997; Barševskis 2001; Silfverberg 2004; Audisio 2009; Lopatin et al. 2010.

*parvulus* **O.F. Müller, 1776**. Pileckis and Monsevičius 1982, 1997; Silfverberg 1992, 2004; Monsevičius 1997; Ferenca et al. 2006, 2007; Audisio 2009; Lopatin et al. 2010.

*pini* **(Linnaeus, 1758)**. Pileckis 1960, 1976a; Silfverberg 1992, 2004; Gaidienė 1993; Pileckis and Monsevičius 1997; Masiulis 2007; Audisio 2009; Lopatin et al. 2010.

*punctiger* **Paykull, 1799**.Audisio 2009; Lopatin et al. 2010.

*pusillus* **Fabricius, 1777**. Pileckis 1959, 1960, 1976a; Silfverberg 1992, 2004; Gaidienė 1993; Pileckis and Monsevičius 1997; Ferenca et al. 2002, 2006, 2007; Šablevičius 2003a, b; Ferenca 2004; Audisio 2009; Lopatin et al. 2010.

[*pygmaeus vittula* **Suffrian, 1848**]. Known in northern Poland (Burakowski et al. 1990).

*quadripustulatus* **Fabricius, 1775**. Silfverberg 1992, 2004; Gaidienė 1993; Audisio 2009; Lopatin et al. 2010.

*querceti* **Suffrian, 1848**. Pileckis and Monsevičius 1997; Silfverberg 2004; Ferenca et al. 2006, 2007; Audisio 2009.

*rufipes* **(Goeze, 1777)**. Ferenca et al. 2002.

[*saliceti* **Zebe, 1855**]. Known in Belarus (Audisio 2009), Estonia (Süda 2009), Kaliningrad region (Alekseev 2003), Poland (Burakowski et al. 1990).

*sericeus* **(Linnaeus, 1758)**. Eichwald 1830; Mazurowa and Mazur 1939; Pileckis 1960, 1976a; Lešinskas and Pileckis 1967; Zajančkauskas and Pileckis 1968; Silfverberg 1992, 2004; Gaidienė 1993; Monsevičius 1997; Pileckis and Monsevičius 1997; Šablevičius 2000b, 2011; Gliaudys 2001; Ferenca 2006b; Audisio 2009; Lopatin et al. 2010.

*sexpunctatus* **(Linnaeus, 1758)**. Eichwald 1830; Pileckis 1959, 1960, 1976a; Silfverberg 1992, 2004; Pileckis and Monsevičius 1997; Šablevičius 2000b, 2011; Audisio 2009; Lopatin et al. 2010.

*solivagus* **Leonardi & Sassi 2001**.Bukejs and Barševskis 2008; Lopatin et al. 2010; Bukejs et al. 2011.

*violaceus* **(Laicharting, 1781)**. Pileckis 1963b, 1976a; Lopatin 1986; Pileckis and Monsevičius 1997; Silfverberg 2004; Audisio 2009; Lopatin et al. 2010.

*virens **Suffrian, 1847**. Pileckis 1960, 1976a; disproved by Pileckis and Monsevičius (1997).

*vittatus* **Fabricius, 1775**. Mazurowa and Mazur 1939; Pileckis 1960, 1963b, 1970b, 1976a; Silfverberg 1992, 2004; Gaidienė 1993; Pileckis and Monsevičius 1997; Šablevičius 2000b, 2011; Gliaudys 2001; Audisio 2009.

**Eumolpinae** **Hope, 1840**.

**Eumolpini Hope, 1840**.

*Eumolpus* **Illiger, 1798**.

*asclepiadeus* **(Pallas, 1776)**. Pileckis and Monsevičius1982, 1997 (*Chrysochus*); Lopatin 1986; Silfverberg 1992, 2004; Audisio 2009.

**Adoxini Baly, 1863**.

*Pachnephorus* **Chevrolat, 1836**.

*pilosus* **(P. Rossi, 1790)**. Roubal 1910; Mazurowa and Mazur 1939; Pileckis 1960, 1976a; Silfverberg 1992, 2004; Gaidienė 1993; Pileckis and Monsevičius 1997; Ferenca 2006b; Audisio 2009; Moseyko and Sprecher-Uebersax 2010.

*tessellatus* **(Duftschmid, 1825)**. Pileckis 1976a; Silfverberg 1992, 2004; Pileckis and Monsevičius 1997; Ferenca et al. 2002; Audisio 2009; Moseyko and Sprecher-Uebersax 2010.

*Bromius* **Chevrolat, 1837** = *Adoxus* Kirby, 1837.

*obscurus* **(Linnaeus, 1758)**. Mazurowa and Mazur 1939; Pileckis 1960, 1963b, 1976a; Silfverberg 1992, 2004; Gaidienė 1993; Monsevičius 1997; Pileckis and Monsevičius 1997; Šablevičius 2000b, 2011; Ferenca 2006b; Vaivilavičius 2008; Audisio 2009; Moseyko and Sprecher-Uebersax 2010.

**Synetinae** **LeConte & Horn, 1883**.

*Syneta* **Dejean, 1837**.

*betulae* **(Fabricius, 1792)**. Strazdienė 1976.

**CURCULIONOIDEA Latreille, 1802**.

**NEMONYCHIDAE Bedel, 1882**.

**Cimberidinae** **Gozis, 1882**.

**Cimberidini Gozis, 1882**.

*Cimberis* **Gozis, 1881** = *Rhinomacer* auct. nec O.F. Müller, 1764.

*attelaboides* **(Fabricius, 1787)**. Silfverberg 1992, 2004; Pileckis and Monsevičius 1997; Šablevičius 2003a; Alonso-Zarazaga 2009a.

**Doydirhynchini Pierce, 1916**.

*Doydirhynchus* **Dejean, 1821**.

*austriacus* **(Olivier, 1807)**. Tamutis 2003.

**ANTHRIBIDAE Billberg, 1820**.

**Anthribinae** **Billberg, 1820**.

**Tropiderini Lacordaire, 1865**.

*Tropideres* **Schönherr, 1823**.

*albirostris* **(Herbst, 1783)**. Gaidienė 1993; Šablevičius and Ferenca 1995; Pileckis and Monsevičius 1997; Šablevičius 2000b; Tamutis and Zolubas 2001; Ferenca et al. 2002; 2006, 2007; Šablevičius 2003a; Silfverberg 2004.

*Gonotropis* **LeConte, 1876**.

*dorsalis* **(Thunberg, 1796)**. Pileckis and Monsevičius 1982, 1997; Silfverberg 1992, 2004; Ferenca et al. 2002; Ehnström et al. 2003; Alonso-Zarazaga 2009a; Ostrauskas and Ferenca 2010.

**Stenocerini Kolbe, 1895**.

*Allandrus* **LeConte, 1876** = *Tropiderinus* auct. nec Reitter, 1916.

*undulatus* **(Panzer, 1795)**. Tamutis 2003.

*Enedreytes* **Schönherr, 1839**.

[*sepicola* **(Fabricius, 1792)**]. Known in Denmark, southern Sweden (Lundberg and Gustafsson 1995), Poland (Wanat and Mokrzycki 2005), Kaliningrad region (Alekseev 2005b).

**Zygaenodini Lacordaire, 1865**.

*Rhaphitropis* **Reitter, 1916**.

*marchicus* **(Herbst, 1797)**. Ferenca et al. 2002.

*Dissoleucas* **Jordan, 1925**.

*niveirostris* **(Fabricius, 1798)**. Miländer et al. 1984; Silfverberg 1992, 2004; Gaidienė 1993; Pileckis and Monsevičius 1997; Gedminas 2005; Ferenca et al. 2006, 2007; Gedminas et al. 2007; Alonso-Zarazaga 2009a.

**Platyrhinini Bedel, 1882**.

*Platyrhinus* **Clairville & Schellenberg, 1798**.

*resinosus* **(Scopoli, 1763)**. Pileckis and Monsevičius 1982, 1997; Silfverberg 1992, 2004; Ferenca 2003; Ehnström et al. 2003; Inokaitis 2004; Butvila et al. 2007; Alonso-Zarazaga 2009a.

**Platysomini Pierce, 1916**.

*Platystomus* **Schneider, 1791**.

*albinus* **(Linnaeus, 1758)**. Pileckis 1960, 1976a; Lešinskas and Pileckis 1967; Silfverberg 1992, 2004; Gaidienė 1993; Pileckis and Monsevičius 1997; Gedminas 2005; Ferenca 2006b; Gedminas et al. 2007; Alonso-Zarazaga 2009a.

**Anthribini Billberg, 1820**.

*Anthribus* **Geoffroy, 1762** = *Brachytarsus* Schönherr, 1823.

[*fasciatus* **Forster, 1770**]. Known in Latvia (Telnov 2004), Estonia, Denmark, southern Sweden (Lundberg and Gustafsson 1995), Poland (Wanat and Mokrzycki 2005).

*nebulosus* **Forster, 1770**. Pileckis 1968a, 1976a; Silfverberg 1992, 2004; Gaidienė 1993; Pileckis and Monsevičius 1997; Ferenca 2006b; Alonso-Zarazaga 2009a.

*scapularis* **(Gebler, 1833)**. Pileckis 1968b, 1976a; Zajančkauskas and Pileckis 1968; Silfverberg 1992, 2004; Monsevičius 1997; Pileckis and Monsevičius 1997; Alonso-Zarazaga 2009a.

**Choraginae** **Kirby, 1819**.

**Araecerini Lacordaire, 1865**.

*Araecerus* **Schönherr, 1823**.

*coffeae* **(Fabricius, 1801)** = *fasciculatus* auct. nec (DeGeer, 1775). Tamutis 2003.

**Choragini Kirby, 1819**.

*Choragus* **Kirby, 1819**.

[*horni* **Wolfrum, 1930**]. Known in Latvia (Telnov 2004), Denmark, southern Sweden (Lundberg and Gustafsson 1995), Poland (Wanat and Mokrzycki 2005).

[*sheppardi* **Kirby, 1819**]. Known in Denmark, southern Sweden (Lundberg and Gustafsson 1995), Estonia (Süda 2009), Poland (Wanat and Mokrzycki 2005), Kaliningrad region (Alekseev 2005b).

**Urodontinae** **Thomson, 1859**. (Urodontidae)

*Bruchela* **Dejean, 1821** = *Urodon* Schönherr, 1823.

[*suturalis **(Fabricius, 1793)**]. # 88. Silfverberg 2004.

**ATTELABIDAE Billberg, 1820.**

**Attelabinae** **Billberg, 1820**.

**Attelabini Billberg, 1820**.

*Attelabus* **Linnaeus, 1758**.

*nitens* **(Scopoli, 1763)**. Pileckis 1959, 1960, 1974a, b, 1976a; Pileckis et al. 1968; Silfverberg 1992, 2004; Gaidienė 1993; Pileckis and Monsevičius 1997; Šablevičius 2000b, 2011; Biondi 2009.

**Apoderinae** **Jekel, 1860**.

**Apoderini Jekel, 1860**.

*Apoderus* **Olivier, 1807**.

*coryli* **(Linnaeus, 1758)**. Heyden 1903; Pileckis 1960, 1974a, b, 1976a; Lešinskas and Pileckis 1967; Zajančkauskas and Pileckis 1968; Pileckis et al. 1968; Silfverberg 1992, 2004; Gaidienė 1993; Monsevičius 1997; Pileckis and Monsevičius 1997; Šablevičius 2000b, 2011; Gliaudys 2001; Žiogas and Zolubas 2005; Ferenca 2006b.

*erythropterus* **(Gmelin, 1790)**. Pileckis 1960, 1974a, b, 1976a; Silfverberg 1992, 2004; Gaidienė 1993; Pileckis and Monsevičius 1997; Ferenca et al. 2006, 2007; Ferenca 2006b.

**Rhynchitinae** **Gistel, 1848**.

**Auletini Desbrochers, 1908**.

*Auletobius* **Desbrochers, 1869**.

[*sanguisorbae* **(Schrank, 1798)**].Known in Poland (Wanat and Mokrzycki 2005), northern and central European Russia (Legalov 2006).

**Rhynchitini Gistel, 1848**.

*Lasiorhynchites* **Jekel, 1860**.

[*cavifrons* **(Gyllenhal, 1833)**]. Known in Denmark (Lundberg and Gustafsson 1995), Poland (Wanat and Mokrzycki 2005).

*coeruleocephalus* **(Schaller, 1783)**. Pileckis and Monsevičius 1982, 1997; Ivinskis et al. 1984; Silfverberg 1992, 2004; Monsevičius 1997.

[*olivaceus* **(Gyllenhal, 1833)**]. Known in Denmark, southern Sweden (Lundberg and Gustafsson 1995), Poland (Wanat and Mokrzycki 2005).

[*sericeus* **(Herbst, 1797)**]. Known in Denmark (Lundberg and Gustafsson 1995), Poland (Wanat and Mokrzycki 2005).

*Temnocerus* **Thunberg, 1815** = *Pselaphorhychites* Schilsky, 1903.

*longiceps* **(Thomson, 1888)**. Pileckis and Monsevičius 1997; Silfverberg 2004.

*nanus* **(Paykull, 1792)**. Roubal 1910; Pileckis 1960, 1963b; 1974a, b, 1976a; Silfverberg 1992, 2004; Gaidienė 1993; Pileckis and Monsevičius 1997.

*tomentosus* **(Gyllenhal, 1839)**. Gaidienė 1993; Pileckis and Monsevičius 1997; Silfverberg 2004.

*Neocoenorrhinus* **Voss, 1952** = *Caenorhinus* auct. nec Thomson, 1859.

*aeneovirens* **(Marsham, 1802)**. Gaidienė 1993; Tamutis 2003; Silfverberg 2004.

*germanicus* **(Herbst, 1797)**. Gaidienė 1993; Silfverberg 2004.

[*interpunctatus* **(Stephens, 1831)**]. Known in Denmark, southern Sweden (Lundberg and Gustafsson 1995), Estonia (Silfverberg 2004), Poland (Wanat and Mokrzycki 2005), Kaliningrad region (Alekseev 2005b).

*pauxillus* **(Germar, 1824)**. Pileckis 1959, 1960, 1974a, b, 1976a; Lešinskas and Pileckis 1967; Mensonienė 1981; Silfverberg 1992, 2004; Gaidienė 1993; Pileckis et al. 1994a; Pileckis and Monsevičius 1997; Biondi 2009.

*Tatianaerhynchites* **Legalov, 2002**.

*aequatus* **(Linnaeus, 1767)**. Eichwald 1830; Heyden 1903; Pileckis 1960, 1974a, b, 1976a; Pileckis and Vengeliauskaitė 1977, 1996; Mensonienė 1981; Silfverberg 1992, 2004; Pileckis et al. 1994a; Pileckis and Monsevičius 1997 (*Coenorhinus*); Šablevičius 2000b, 2011; Biondi 2009.

*Involvulus* **Schrank, 1798**.

*cupreus* **(Linnaeus, 1758)**. Pileckis 1960, 1974a, b, 1976a; Lešinskas and Pileckis 1967; Mensonienė 1981; Silfverberg 1992, 2004; Pileckis and Monsevičius 1997 (*Rhynchites*); Ferenca 2006b; Biondi 2009.

*Haplorhynchites* **Voss, 1924**.

*caeruleus* **(DeGeer, 1775)**. Tamutis 2003.

[*pubescens* **(Fabricius 1775)**]. Known in Poland (Wanat and Mokrzycki 2005), northwestern and central European Russia (Legalov 2006).

*Rhynchites* **Schneider, 1791**.

*auratus* **(Scopoli, 1763)**. Pileckis 1960, 1974a, b, 1976a; Silfverberg 1992, 2004; Gaidienė 1993; Pileckis and Monsevičius 1997; Ferenca 2006b.

*bacchus* **(Linnaeus, 1758)**. Pileckis 1959, 1960, 1974a, b, 1976a; Lešinskas and Pileckis 1967; Silfverberg 1992, 2004; Pileckis et al. 1994a; Pileckis and Monsevičius 1997.

**Byctiscini Voss, 1923**.

*Bytiscus* **Thomson, 1859**.

*betulae* **(Linnaeus, 1758)**. Ivanauskas and Vailionis 1922; Pileckis 1960, 1974a, b, 1976a; Lešinskas and Pileckis 1967; Pileckis et al. 1968; Zajančkauskas and Pileckis 1968; Pileckis and Vengeliauskaitė 1977, 1996; Silfverberg 1992, 2004; Gaidienė 1993; Pileckis et al. 1994a; Monsevičius 1997; Pileckis and Monsevičius 1997; Šablevičius 2000b, 2011; Gliaudys 2001; Ferenca 2006b.

*populi* **(Linnaeus, 1758)**. Eichwald 1830; Heyden 1903; Pileckis 1960, 1974a, b, 1976a; Lešinskas and Pileckis 1967; Pileckis et al. 1968; Zajančkauskas and Pileckis 1968; Silfverberg 1992, 2004; Gaidienė 1993; Monsevičius 1997; Pileckis and Monsevičius 1997; Šablevičius 2000b, 2011; Tamutis and Zolubas 2001; Ferenca 2006b.

**Deporaini Voss, 1929**.

*Deporaus* **Leach, 1819** = *Caenorhinus* Thomson, 1859.

*betulae* **(Linnaeus, 1758)**. Eichwald 1830; Ogyjewicz 1938; Pileckis 1960, 1974a, b, 1976a; Lešinskas and Pileckis 1967; Pileckis et al. 1968; Zajančkauskas and Pileckis 1968; Silfverberg 1992, 2004; Gaidienė 1993; Monsevičius 1997; Pileckis and Monsevičius 1997; Šablevičius 2000b, 2011; Gliaudys 2001; Ferenca 2006b.

*mannerheimii* **(Hummel, 1823)**. Pileckis 1962, 1963b, 1974a, b, 1976a; Silfverberg 1992, 2004; Pileckis and Monsevičius 1997; Ivinskis et al. 2009.

**BRENTIDAE Billberg, 1820.** (Curculionidae)

**Apioninae** **Schönherr, 1823**.

**Apionini Schönherr, 1823**.

*Omphalapion* **Schilsky, 1901**.

[*dispar* **(Germar, 1817)**]. Known in Estonia, Denmark, Sweden (Lundberg and Gustafsson 1995), Belarus (Alexandrovitch et al. 1996), throughout Poland (Smreczyński 1965; Wanat and Mokrzycki 2005).

*hookerorum* **(Kirby, 1808)** = *hookeri* (Kirby, 1808). Pileckis 1976a; Silfverberg 1992, 2004; Gaidienė 1993; Pileckis and Monsevičius 1997 (*Apion*); Alonso-Zarazaga 2009a.

*laevigatum* **(Paykull, 1792)** = *sorbi* (Fabricius, 1792). Tamutis and Pankevičius 2001.

*Ceratapion* **Schilsky, 1901**.

[*armatum* **(Gerstaecker, 1854)**]. Known in Latvia (Telnov et al. 2005), Estonia, Denmark, southern Sweden (Lundberg and Gustafsson 1995), Poland (Smreczyński 1965; Wanat and Mokrzycki 2005), northern Belarus (Alexandrovitch et al. 1996).

[*austriacum* **(Wagner, 1904)**]. Known in Latvia (Telnov et al. 2005), Estonia, Denmark, (Lundberg and Gustafsson 1995), Poland (Smreczyński 1965; Wanat and Mokrzycki 2005).

[*basicorne* **(Illiger, 1807)** = *alliariae* auct. nec (Linnaeus, 1758)]. Known in Latvia (Telnov et al. 2005), Denmark, southern Sweden (Lundberg and Gustafsson 1995), throughout Poland (Smreczyński 1965; Wanat and Mokrzycki 2005).

[*carduorum* **(Kirby, 1808)**]. Known in Denmark, southern Sweden (Lundberg and Gustafsson 1995), throughout Poland (Smreczyński 1965; Wanat and Mokrzycki 2005) and Belarus (Alexandrovitch et al. 1996).

*gibbirostre* **(Gyllenhal, 1813)** = *carduorum* auct. nec (Kirby, 1808). Pileckis and Monsevičius 1997 (*Apion*); Silfverberg 2004.

*onopordi* **(Kirby, 1808)**. Miländer 1982; Silfverberg 1992, 2004; Gaidienė 1993; Pileckis and Monsevičius 1997 (*Apion*); Alonso-Zarazaga 2009a.

*penetrans* **(Germar, 1817)**. Pileckis 1976a; Silfverberg 1992, 2004; Pileckis and Monsevičius 1997 (*Apion*); Alonso-Zarazaga 2009a.

*Diplapion* **Reitter, 1916**.

*confluens* **(Kirby, 1808)**. Pileckis 1960, 1976a Silfverberg 1992, 2004; Gaidienė 1993; Pileckis and Monsevičius 1997 (*Apion*); Alonso-Zarazaga 2009a.

[*detritum* **(Mulsant & Rey, 1859)**]. Known in Latvia (Telnov 2010), Denmark (Lundbrg 1995), Poland (Smreczyński 1965; Wanat and Mokrzycki 2005).

*stolidum* **(Germar, 1817)**. Silfverberg 2004; Alonso-Zarazaga 2009a.

*Taphrotopium* **Reitter, 1916**.

*sulcifrons* **(Herbst, 1797)**. Mensonienė 1974; Miländer 1982; Silfverberg 1992, 2004; Gaidienė 1993; Pileckis and Monsevičius 1997 (*Apion*); Alonso-Zarazaga 2009a.

*Aspidapion* **Schilsky, 1901**.

*aeneum* **(Fabricius, 1775)**. Pileckis and Monsevičius 1997 (*Apion*); Silfverberg 2004.

*radiolus* **(Marsham, 1802)**. Heyden 1903; Pileckis 1960, 1976a; Silfverberg 1992, 2004; Pileckis and Monsevičius 1997 (*Apion*); Alonso-Zarazaga 2009a.

*Trichopterapion* **Wagner, 1930**.

[*holosericeum* **(Gyllenhal, 1833)**]. Known in Latvia (Telnov 2004), western Belarus (Alexandrovitch et al. 1996).

*Melanapion* **Wagner, 1930**.

*minimum* **(Herbst, 1797)**. Pileckis 1960, 1963b, 1976a; Silfverberg 1992, 2004; Monsevičius 1997; Pileckis and Monsevičius 1997 (*Apion*); Alonso-Zarazaga 2009a.

*Squamapion* **Bokor, 1923**.

[*atomarium* **(Kirby, 1808)**]. Known in Latvia (Telnov 2004), Estonia, Denmark, southern Sweden (Lundberg and Gustafsson 1995), throughout Belarus (Alexandrovitch et al. 1996) and Poland (Smreczyński 1965; Wanat and Mokrzycki 2005).

*cineraceum* **(Wencker, 1864)** = *millum* (Bach, 1854). Smreczynski 1965; Pileckis 1968b, 1976a; Silfverberg 1992, 2004; Pileckis and Monsevičius 1997 (*Apion*); Alonso-Zarazaga 2009a.

*elongatum* **(Germar, 1817)**. Miländer 1982; Gaidienė 1993; Silfverberg 1992, 2004; Pileckis and Monsevičius 1997 (*Apion*); Alonso-Zarazaga 2009a.

[*flavimanum* **(Gyllenhal, 1833)**]. Known in Latvia (Telnov 2004), Estonia, Denmark, Sweden (Lundberg and Gustafsson 1995), Belarus (Alexandrovitch et al. 1996), Poland (Smreczyński 1965; Wanat and Mokrzycki 2005).

[*hoffmanni* **(Wagner, 1930)**]. Known in northwestern Belarus (Alexandrovitch et al. 1996), southern Sweden (Lundberg and Gustafsson 1995), Poland (Smreczyński 1965; Wanat and Mokrzycki 2005).

[*oblivium* **(Schilsky, 1902)**]. Known in Latvia (Telnov 2010), Denmark, southern Sweden (Lundberg and Gustafsson 1995), Poland (Smreczyński 1965; Wanat and Mokrzycki 2005).

[*origani* **(Planet, 1918)**]. Known in southern Sweden (Lundberg and Gustafsson 1996).

[*vicinum* **(Kirby, 1808)**]. Known in Estonia, Denmark, southern Sweden (Lundberg and Gustafsson 1995), Belarus (Alexandrovitch et al. 1996), Poland (Smreczyński 1965; Wanat and Mokrzycki 2005).

*Kalcapion* **Schilsky, 1906**.

*pallipes* **(Kirby, 1808)**. Tamutis and Pankevičius 2001.

*Taeniapion* **Schilsky, 1906**.

*urticarium* **(Herbst, 1784)**. Pileckis and Monsevičius 1997 (*Apion*); Silfverberg 2004; Ferenca et al. 2006, 2007.

*Malvapion* **Hoffmann, 1958**.

[*malvae* **(Fabricius, 1775)**]. Known in northwestern Belarus (Alexandrovitch et al. 1996), Poland (Smreczyński 1965; Wanat and Mokrzycki 2005).

*Pseudapion* **Schilsky, 1906**.

*rufirostre* **(Fabricius, 1775)**. Ferenca and Tamutis 2009.

*Exapion* **Bedel, 1887**.

[*difficile* **(Herbst, 1784)**]. Known in southern Belarus (Alexandrovitch et al. 1996), throughout Poland (Smreczyński 1965; Wanat and Mokrzycki 2005), Denmark (Lundberg and Gustafsson 1995).

*elongatulum* **(Desbrochers, 1891)**. Silfverberg 2004.

[*formaneki* **(Wagner, 1929)**]. Known in western Belarus (Alexandrovitch et al. 1996), throughout Poland (Smreczyński 1965; Wanat and Mokrzycki 2005).

[*fuscirostre* **(Fabricius, 1775)**]. Known in Denmark, southern Sweden (Lundberg and Gustafsson 1995), Belarus (Alexandrovitch et al. 1996), throughout Poland (Smreczyński 1965; Wanat and Mokrzycki 2005).

*Pseudoprotapion* **Ehret, 1990**.

*astragali* **(Paykull, 1800)**. Gaidienė 1993; Pileckis and Monsevičius 1997 (*Apion*); Silfverberg 2004.

[*elegantulum* **(Germar, 1818)**]. Known in northwestern Belarus (Alexandrovitch et al. 1996).

*Protapion* **Schilsky, 1908**.

*apricans* **(Herbst, 1797)**. Pileckis 1960, 1976a; Lešinskas and Pileckis 1967; Zajančkauskas and Pileckis 1968; Mensonienė 1974; Pileckis and Vengeliauskaitė 1977, 1996; Terechova 1978, 1986; Silfverberg 1992, 2004; Gaidienė 1993; Pileckis et al. 1994b; Monsevičius 1997; Pileckis and Monsevičius 1997 (*Apion*); Gliaudys 2001; Tamutis and Zolubas 2001; Šurkus and Gaurilčikienė 2002; Ferenca 2006b; Alonso-Zarazaga 2009a.

*assimile* **(Kirby, 1808)**. Pileckis 1960, 1976a; Lešinskas and Pileckis 1967; Silfverberg 1992, 2004; Gaidienė 1993; Monsevičius 1997; Pileckis and Monsevičius 1997 (*Apion*); Gliaudys 2001; Alonso-Zarazaga 2009a.

*dissimile* **(Germar, 1817)**. Ferenca et al. 2002.

*filirostre* **(Kirby, 1808)**. Pileckis 1976a; Silfverberg 1992, 2004; Gaidienė 1993; Pileckis and Monsevičius 1997 (*Apion*); Alonso-Zarazaga 2009a.

*fulvipes* **(Geoffroy, 1785)** = *dichroum* (Bedel, 1885) = *flavipes* (Paykull, 1792) nec (DeGeer, 1775). Ogyjewicz 1938; Pileckis 1960, 1976a; Lešinskas and Pileckis 1967; Pileckis and Vengeliauskaitė 1977, 1996; Terechova 1986; Silfverberg 1992, 2004; Gaidienė 1993; Pileckis et al. 1994b; Monsevičius 1997; Pileckis and Monsevičius 1997 (*Apion*); Gliaudys 2001; Šurkus and Gaurilčikienė 2002; Ferenca 2006b; Alonso-Zarazaga 2009a; Ostrauskas and Ferenca 2010.

*gracilipes* **(Dietrich, 1857)**. Gønget 1997; Silfverberg 2004.

[*interjectum* **(Desbrochers, 1895)** = *boreum* (Gönget, 1997)]. Known in Latvia (Telnov et al. 2005), Denmark, southern Sweden (Lundberg and Gustafsson 1995), Poland (Smreczyński 1976; Wanat and Mokrzycki 2005). 

[*nigritarse* **(Kirby, 1808)**]. Known in Latvia (Telnov 2004), Estonia, Denmark, southern Sweden (Lundberg and Gustafsson 1995), throughout Belarus (Alexandrovitch et al. 1996) and Poland (Smreczyński 1965; Wanat and Mokrzycki 2005).

*ononidis* **(Gyllenhal, 1827)** = *ononicola* (Bach, 1854). Tamutis 2004.

*ruficrus* **(Germar, 1817)**. Tamutis and Pankevičius 2001; Alonso-Zarazaga 2009a.

*trifolii* **(Linnaeus, 1768)** = *aestivum* (Germar, 1817). Ogyjewicz 1938; Pileckis 1960, 1976a; Lešinskas and Pileckis 1967; Zajančkauskas and Pileckis 1968; Silfverberg 1992, 2004; Gaidienė 1993; Monsevičius 1997; Pileckis and Monsevičius 1997 (*Apion*); Gliaudys 2001; Ferenca 2006b; Alonso-Zarazaga 2009a.

*varipes* **(Germar, 1817)**. Miländer 1982; Silfverberg 1992, 2004; Pileckis and Monsevičius 1997 (*Apion*); Ferenca 2006b; Alonso-Zarazaga 2009a.

*Pseudoperapion* **Wagner, 1930**.

*brevirostre* **(Herbst, 1797)**.Žiogas and Zolubas 2005.

*Pseudostenapion* **Wagner, 1930**.

*simum* **(Germar, 1817)**. Miländer 1982; Gaidienė 1993; Pileckis and Monsevičius 1997 (*Apion*); Silfverberg 1992, 2004; Alonso-Zarazaga 2009a.

*Perapion* **Wagner, 1907**.

*affine* **(Kirby, 1808)**. Tamutis 2004.

*curtirostre* **(Germar, 1817)**. Roubal 1910; Pileckis 1960, 1976a; Silfverberg 1992, 2004; Gaidienė 1993; Pileckis and Monsevičius 1997 (*Apion*); Alonso-Zarazaga 2009a.

[*hydrolapathi* **(Marsham, 1802)**]. Known in Denmark, southern Sweden (Lundberg and Gustafsson 1995).

*marchicum* **(Herbst, 1797)**. Bercio and Folwaczny 1979; Silfverberg 1992, 2004; Alonso-Zarazaga 2009a.

[*oblongum* **(Gyllenhal, 1827)**]. # 89.Known in Latvia (Telnov 2004), Belarus (Alexandrovitch et al. 1996), Poland (Wanat and Mokrzycki 2005).

*violaceum* **(Kirby, 1808)**. Mazurowa and Mazur 1939; Pileckis 1960, 1976a; Silfverberg 1992, 2004; Gaidienė 1993; Monsevičius 1997; Pileckis and Monsevičius 1997 (*Apion*); Ferenca 2006b; Alonso-Zarazaga 2009a.

*Aizobius* **Alonso-Zarazaga 1990**.

*sedi* **(Germar, 1818)**. Miländer 1982; Silfverberg 1992, 2004; Gaidienė 1993; Pileckis and Monsevičius 1997 (*Apion*); Alonso-Zarazaga 2009a.

*Apion* **Herbst, 1797**.

*cruentatum* **Walton, 1844**. Pileckis 1960; Pileckis and Monsevičius 1997; Silfverberg 2004; Ferenca 2006b.

*frumentarium* **(Linnaeus, 1758)** = *sanguineum* (DeGeer, 1775) = *miniatum* Germar, 1833. Eichwald 1830; Pileckis 1960, 1976a; Silfverberg 1992, 2004; Gaidienė 1993; Pileckis and Monsevičius 1997; Ferenca 2006b; Alonso-Zarazaga 2009a.

*haematodes* **Kirby, 1808**. Silfverberg 1992, 2004; Gaidienė 1993; Pileckis and Monsevičius 1997; Alonso-Zarazaga 2009a.

*rubens* **Stephens, 1839**. Silfverberg 2004.

*rubiginosum* **Grill, 1893** = *sanguineum* auct. nec (DeGeer, 1775). Silfverberg 1992, 2004; Alonso-Zarazaga 2009a.

*Catapion* **Schilsky, 1906**.

[*burdigalense* **(Wencker, 1859)**]. Known in northern and northwestern Belarus (Alexandrovitch et al. 1996).

[*meieri* **(Desbrochers, 1901)**]. Known in Latvia (Telnov 2010), Denmark, southern Sweden (Lundberg and Gustafsson 1995), Estonia (Silfverberg 2004), Poland (Wanat and Mokrzycki 2005).

[*pubescens* **(Kirby, 1808)**]. Known in Latvia (Telnov et al. 2005), Estonia, Denmark, southern Sweden (Lundberg and Gustafsson 1995), western Belarus (Alexandrovitch et al. 1996), throughout Poland (Smreczyński 1965; Wanat and Mokrzycki 2005).

*seniculus* **(Kirby, 1808)**. Pileckis 1960, 1976a; Pileckis and Vengeliauskaitė 1977, 1996; Terechova 1986; Silfverberg 1992, 2004; Gaidienė 1993; Pileckis et al. 1994b; Pileckis and Monsevičius 1997 (*Apion*); Ferenca 2006b; Alonso-Zarazaga 2009a.

*Betulapion* **Ehret, 1994**.

*simile* **(Kirby, 1811)**. Pileckis et al. 1968; Silfverberg 1992, 2004; Gaidienė 1993; Žiogas and Zolubas 2005; Ferenca 2006b; Alonso-Zarazaga 2009a; Ostrauskas and Ferenca 2010.

*Stenopterapion* **Bokor, 1923**.

*meliloti* **(Kirby, 1808)**. Semaškienė et al. 1999.

*tenue* **(Kirby, 1808)**. Miländer 1982; Silfverberg 1992, 2004; Pileckis and Monsevičius 1997 (*Apion*); Alonso-Zarazaga 2009a.

*Ischnopterapion* **Bokor, 1923**.

*loti* **(Kirby, 1808)**. Miländer 1982; Silfverberg 1992, 2004; Gaidienė 1993; Pileckis and Monsevičius 1997 (*Apion*); Alonso-Zarazaga 2009a.

[*modestum* **(Germar, 1817)**]. Known in Denmark, southern Sweden (Lundberg and Gustafsson 1995), Poland (Wanat and Mokrzycki 2005).

*virens* **(Herbst, 1797)**. Roubal 1910; Pileckis 1960, 1976a; Terechova 1978; Silfverberg 1992, 2004; Gaidienė 1993; Pileckis and Monsevičius 1997 (*Apion*); Alonso-Zarazaga 2009a; Ivinskis et al. 2009.

*Protopirapion* **Alonso-Zarazaga, 1990**.

[*atratulum* **(Kirby, 1808)**]. Known in Denmark (Lundberg and Gustafsson 1995), Poland (Wanat and Mokrzycki 2005). 

*Synapion* **Schilsky, 1902**.

*ebeninum* **(Kirby, 1808)**. Gaidienė 1993; Silfverberg 2004.

*Holotrichapion* **Gyorffy, 1956**.

*aethiops* **(Herbst, 1797)**. Pileckis and Monsevičius 1997; Silfverberg 2004.

*ononis* **(Kirby, 1808)**. Bercio and Folwaczny 1979; Silfverberg 1992, 2004; Alonso-Zarazaga 2009a.

*pisi* **(Fabricius, 1801)**. Miländer 1982; Silfverberg 1992, 2004; Pileckis and Monsevičius 1997 (*Apion*); Alonso-Zarazaga 2009a.

*pullum* **(Gyllenhal, 1833)** = *aestimatum* (Faust, 1890). Pileckis 1976a; Silfverberg 1992, 2004; Pileckis and Monsevičius 1997 (*Apion*); Alonso-Zarazaga 2009a.

*Hemitrichapion* **Voss, 1959**.

*pavidum* **(Germar, 1817)**. Pileckis and Monsevičius 1997 (*Apion*); Silfverberg 2004.

*Pirapion* **Reitter, 1916**.

[*immune* **(Kirby, 1808)**].Known in Denmark (Lundberg and Gustafsson 1995), northwestern Belarus (Alexandrovitch et al. 1996), northern and northwestern Poland (Smreczyński 1965; Wanat and Mokrzycki 2005).

*Mesotrichapion* **Gyorffy, 1956**.

*punctirostre* **(Gyllenhal, 1839)**. Gaidienė 1993; Silfverberg 2004.

*Cyanapion* **Bokor, 1923**.

[*afer* **(Gyllenhal, 1833)**]. Known in Latvia (Telnov 2004), Poland (Wanat and Mokrzycki 2005), Estonia, southern Sweden (Lundberg and Gustafsson 1995), probably noted for northwestern Belarus as *Apion platalea* Germar, 1817 (Alexandrovitch et al. 1996), mistakenly synonymised with *Apion punctigerum* (Fabricius, 1992) by R. Ferenca (2006b).

[*columbinum* **(Germar, 1817)**]. Known Denmark, southern Sweden (Lundberg and Gustafsson 1995), Estonia (Silfverberg 2004), Poland (Smreczyński 1965; Wanat and Mokrzycki 2005).

*gyllenhali* **(Kirby, 1808)**. Miländer 1982; Silfverberg 1992, 2004; Gaidienė 1993; Pileckis and Monsevičius 1997 (*Apion*).

*spencii* **(Kirby, 1808)**. Miländer 1982; Silfverberg 1992, 2004; Gaidienė 1993; Pileckis and Monsevičius 1997 (*Apion*); Alonso-Zarazaga 2009a.

*Oxystoma* **Duméril, 1805**.

*cerdo* **(Gerstaecker 1854)**. Pileckis 1963b, 1976; Zubrys 1967; Silfverberg 1992, 2004; Pileckis and Monsevičius 1997 (*Apion*); Žiogas and Zolubas 2005; Alonso-Zarazaga 2009a.

*craccae* **(Linnaeus 1767)**. Miländer 1982; Silfverberg 1992, 2004; Pileckis and Monsevičius 1997 (*Apion*); Ferenca 2006b; Alonso-Zarazaga 2009a.

[*ochropus* **(Germar, 1818)**]. Known in northwestern Belarus (Alexandrovitch et al. 1996), throughout Poland (Smreczyński 1965; Wanat and Mokrzycki 2005).

*opeticum* **(Bach, 1854)**. Heyden 1903; Mensonienė 1974; Pileckis 1960, 1976a; Silfverberg 1992, 2004; Gaidienė 1993; Pileckis and Monsevičius 1997 (*Apion*); Alonso-Zarazaga 2009a.

*pomonae* **(Fabricius, 1798)**. Heyden 1903; Pileckis 1960, 1976a; Zajančkauskas and Pileckis 1968; Silfverberg 1992, 2004; Monsevičius 1997; Pileckis and Monsevičius 1997 (*Apion*); Alonso-Zarazaga 2009a.

*subulatum* **(Kirby, 1808)**. Pileckis 1959, 1960, 1976a; Silfverberg 1992, 2004; Gaidienė 1993; Pileckis and Monsevičius 1997 (*Apion*); Ferenca 2006b; Alonso-Zarazaga 2009a.

*Eutrichapion* **Reitter, 1916**.

*ervi* **(Kirby, 1808)**. Gaidienė 1993; Pileckis and Monsevičius 1997 (*Apion*); Silfverberg 2004.

[*facetum* **(Gyllenhal, 1839)**]. Known in Latvia (Telnov 2004), Estonia, Denmark, throughout Sweden (Lundberg and Gustafsson 1995), northwestern Belarus (Alexandrovitch et al. 1996), Poland (Wanat and Mokrzycki 2005).

*melancholicum* **(Wencker, 1864)**. Tamutis and Pankevičius 2001.

*punctigerum* **(Paykull, 1792)**. Pileckis 1960, 1976a; Silfverberg 1992, 2004; Gaidienė 1993; Pileckis and Monsevičius 1997 (*Apion*); Alonso-Zarazaga 2009a; as *A. afer* (Gyllenhal, 1833) in (Ferenca 2006b).

*viciae* **(Paykull, 1800)** = *voisini* (Ehret, 1997). Pileckis 1976a; Silfverberg 1992, 2004; Gaidienė 1993; Pileckis and Monsevičius 1997 (*Apion*); Tamutis 1999; Alonso-Zarazaga 2009a.

*vorax* **(Herbst, 1797)**. Roubal 1910; Pileckis 1960, 1976a; Miländer 1982; Silfverberg 1992, 2004; Pileckis and Monsevičius 1997 (*Apion*); Ferenca 2006b; Alonso-Zarazaga 2009a.

**Nanophyinae** **Gistel, 1848**. (Curculionidae)

**Nanophyini Gistel, 1848**.

*Nanomimus* **Alonso-Zarazaga, 1989**.

[*circumscriptus* **(Aubé, 1864)**]. Known in Latvia (Telnov 2004), Estonia, southern Sweden (Lundberg and Gustafsson 1995), northwestern Belarus (Alexandrovitch et al. 1996), Poland (Smreczyński 1976; Wanat and Mokrzycki 2005).

[*hemisphaericus* **(Olivier, 1807)**]. Known in northwestern Belarus (Alexandrovitch et al. 1996), Poland (Smreczyński 1976; Wanat and Mokrzycki 2005).

*Nanophyes* **Schönherr, 1838** = *Nanodes* Schönherr, 1825.

*globulum* **(Germar, 1821)**. Tamutis and Pankevičius 2001.

*marmoratus* **(Goeze, 1777)**. Pileckis 1960, 1976a; Silfverberg 1992, 2004; Gaidienė 1993; Pileckis and Monsevičius 1997; Ferenca 2006b; Alonso-Zarazaga 2009a; Šablevičius 2011.

*Microon* **Alonso-Zarazaga, 1989**.

[*sahlbergi* **(R.F. Sahlberg, 1835)**]. Known in Estonia, southern Sweden (Lundberg and Gustafsson 1995), Denmark (Silfverberg 2004), northwestern Belarus (Alexandrovitch et al. 1996), Poland (Smreczyński 1976; Wanat and Mokrzycki 2005).

**DRYOPHTHORIDAE Schönherr, 1825**. (Curculionidae)

**Dryophthorinae Schönherr, 1825**.

*Dryophthorus* **Germar, 1824**.

*corticallis* **(Paykull, 1792)**. Pileckis 1962, 1963b, 1970b, 1976a; Silfverberg 1992, 2004; Gaidienė 1993; Pileckis and Monsevičius 1997; Šablevičius 2004; Ferenca et al. 2006, 2007; Alonso-Zarazaga 2009a.

**Rhynchophorinae** **Schönherr, 1833**.

**Litosomini Lacordaire, 1865**.

*Sitophilus* **Schönherr, 1838**.

*granarius* **(Linnaeus, 1758)**. Ogyjewicz 1934; Mastauskis 1936; Pileckis 1960, 1970a, 1976a, 1998; Lešinskas and Pileckis 1967; Zubrys 1967; Pileckis and Vengeliauskaitė 1977, 1996; Pileckis et al. 1994b; Silfverberg 1992, 2004; Gaidienė 1993; Pileckis and Monsevičius 1997; Alonso-Zarazaga 2009a.

*oryzae* **(Linnaeus, 1763)**. Pileckis and Monsevičius 1997; Tamutis 2003.

[*zeamais* **Motschulsky, 1855**]. Known in Latvia (Telnov 2004), Denmark, Sweden (Lundberg and Gustafsson 1995).

**BRACHYCERIDAE Billberg, 1820**. (Curculionidae)

**Erirhininae** **Schönherr, 1825**.

**Erirhinini Schönherr, 1825**.

*Grypus* **Germar, 1817**.

*brunnirostris* **(Fabricius, 1792)**. Pileckis 1976a; Silfverberg 1992, 2004; Pileckis and Monsevičius 1997; Šablevičius 2000b; Alonso-Zarazaga 2009a.

*equiseti* **(Fabricius, 1775)**. Pileckis 1960, 1976a; Mensonienė 1974; Silfverberg 1992, 2004; Gaidienė 1993; Pileckis and Monsevičius 1997; Šablevičius 2000b, 2011; Ferenca 2006b; Alonso-Zarazaga 2009a.

*Thryogenes* **Bedel, 1884**.

*festucae* **(Herbst, 1795)**. Pileckis 1968a, 1976a; Silfverberg 1992, 2004; Gaidienė 1993; Monsevičius 1997; Pileckis and Monsevičius 1997; Alonso-Zarazaga 2009a.

*nereis* **(Paykull, 1800)**. Pileckis 1960, 1976a; Silfverberg 1992, 2004; Gaidienė 1993; Pileckis and Monsevičius 1997; Ferenca 2006b; Alonso-Zarazaga 2009a.

*scirrhosus* **(Gyllenhal, 1836)**. Tamutis 2004.

*Tournotaris* **Alonso-Zarazaga and Lyal 1999**. # 90.

*bimaculatus* **(Fabricius, 1787)**. Pileckis 1976a; Silfverberg 1992, 2004; Gaidienė 1993; Pileckis and Monsevičius 1997 (*Notaris*); Šablevičius 2000b.

*Notaris* **Germar, 1817**.

*acridula* **(Linnaeus, 1758)**. Pileckis 1959, 1960, 1976a; Silfverberg 1992, 2004; Gaidienė 1993; Pileckis and Monsevičius 1997; Šablevičius 2000b.

*aethiops* **(Fabricius, 1792)**. Pileckis 1960, 1976a; Silfverberg 1992, 2004; Pileckis and Monsevičius 1997; Alonso-Zarazaga 2009a.

*maerkeli* **(Boheman, 1843)**. Pileckis 1960, 1976a; Silfverberg 1992, 2004; Gaidienė 1993; Pileckis and Monsevičius 1997; Ferenca 2006b; Alonso-Zarazaga 2009a.

*scirpi* **(Fabricius, 1792)**. Pileckis 1960, 1976a; Silfverberg 1992, 2004; Gaidienė 1993; Monsevičius 1997; Pileckis and Monsevičius 1997; Šablevičius 2000b; Ferenca 2006b.

**Tanysphyrini Gistel, 1848**.

*Tanysphyrus* **Germar, 1817**.

[*ater* **Blatchley, 1928** = *makolskii* Smreczynski, 1957]. Known in Latvia (Telnov et al. 2006), Sweden (Lundberg and Gustafsson 1995), Poland (Wanat and Mokrzycki 2005).

*lemnae* **(Fabricius, 1792)**. Silfverberg 1992, 2004; Gaidienė 1993; Pileckis and Monsevičius 1997; Šablevičius 2000b, 2011; Alonso-Zarazaga 2009a; Alekseev 2008a.

**CURCULIONIDAE Latreille, 1802**.

**Curculioninae** **Latreille, 1802**.

**Ellescini Thomson, 1859**.

*Ellescus* **Dejean, 1821**.

*bipunctatus* **(Linnaeus, 1758)**. Heyden 1903; Pileckis 1960, 1976a; Silfverberg 1992, 2004; Pileckis and Monsevičius 1997; Alonso-Zarazaga 2009a.

*infirmus* **(Herbst, 1795)**. Miländer 1982; Silfverberg 1992, 2004; Pileckis and Monsevičius 1997; Vaivilavičius 2008; Alonso-Zarazaga 2009a.

*scanicus* **(Paykull, 1792)**. Pileckis 1960, 1976a; Silfverberg 1992, 2004; Pileckis and Monsevičius 1997; Ferenca 2006b; Vaivilavičius 2008; Alonso-Zarazaga 2009a; Ostrauskas and Ferenca 2010.

*Dorytomus* **Germar, 1817**.

*dejeani* **Faust, 1882**. Pileckis and Monsevičius 1997; Silfverberg 2004.

*dorsalis* **(Linnaeus, 1758)**. Pileckis 1959, 1960, 1970b, 1976a; Silfverberg 1992, 2004; Gaidienė 1993; Pileckis and Monsevičius 1997; Šablevičius 2000b, 2003a; Vaivilavičius 2008; Alonso-Zarazaga 2009a.

*edoughensis* **Desbrochers, 1875** sensu Silfverberg, 1992= *affinis* (Paykull, 1800) nec (Schrank, 1781). Pileckis 1976a; Silfverberg 1992, 2004; Pileckis and Monsevičius 1997; Ferenca et al. 2006, 2007; Vaivilavičius 2008; Alonso-Zarazaga 2009a.

*filirostris* **(Gyllenhal, 1836)**. Tamutis 2003.

*hirtipennis* **Bedel, 1884**. Silfverberg 1992, 2004; Pileckis and Monsevičius 1997; Alonso-Zarazaga 2009a.

*ictor* **(Herbst, 1795)** = *validirostris* (Gyllenhal, 1836). Pileckis 1960, 1976a; Silfverberg 1992, 2004; Gaidienė 1993; Pileckis and Monsevičius 1997; Ferenca 2006b; Alonso-Zarazaga 2009a.

*longimanus* **(Forster, 1771)**. Pileckis 1960, 1976a; Mensonienė 1974; Silfverberg 1992, 2004; Gaidienė 1993; Pileckis and Monsevičius 1997; Šablevičius 2000b; Ferenca 2006b; Alonso-Zarazaga 2009a.

*majalis* **(Paykull, 1792)**. Tamutis 2003.

*melanophthalmus* **(Paykull, 1792)**. Silfverberg 1992, 2004; Pileckis and Monsevičius 1997; Šablevičius 2003a, 2004.

[*minutus* **(Gyllenhal, 1836)**]. Known in Latvia (Telnov 2004), Estonia (Lundberg and Gustafsson 1995) Poland (Smreczyński 1972; Wanat and Mokrzycki 2005).

*nordenskioldi* **Faust, 1882**. Pileckis and Monsevičius 1997; Silfverberg 2004.

*occallescens* **(Gyllenhal, 1836)**. Tamutis 2003.

[*reussi* **Formanek, 1908**]. Known in Latvia (Telnov 2004), Poland (Smreczyński 1972; Wanat and Mokrzycki 2005).

*rufatus* **(Bedel, 1888)** = *rufulus* (Bedel, 1884) nec (Mannerheim, 1853). Pileckis 1960, 1976a; Zajančkauskas and Pileckis 1968; Silfverberg 1992, 2004; Monsevičius 1997; Pileckis and Monsevičius 1997; Ferenca 2006b; Alonso-Zarazaga 2009a.

[*salicinus* **(Gyllenhal, 1827)**]. Known in Latvia (Telnov 2004), Estonia, Denmark, throughout Sweden (Lundberg and Gustafsson 1995), Belarus (Alexandrovitch et al. 1996), Poland (Smreczyński 1972; Wanat and Mokrzycki 2005).

*salicis* **Walton, 1851**. Pileckis and Monsevičius 1997; Silfverberg 2004.

*schoenherri* **Faust, 1882**. Pileckis 1960, 1976a; Silfverberg 1992, 2004; Gaidienė 1993; Pileckis and Monsevičius 1997; Ferenca 2006b; Alonso-Zarazaga 2009a.

*suratus* **(Gyllenhal, 1836)** = *flavipes* (Panzer, 1797) nec (DeGeer, 1775). Pileckis and Monsevičius 1997; Silfverberg 2004.

*taeniatus* **(Fabricius, 1781)**. Pileckis 1960, 1976a; Silfverberg 1992, 2004; Pileckis and Monsevičius 1997; Šablevičius 2000b; Vaivilavičius 2008; Alonso-Zarazaga 2009a.

*tortrix* **(Linnaeus, 1761)**. Pileckis 1960, 1976a; Silfverberg 1992, 2004; Gaidienė 1993; Pileckis and Monsevičius 1997; Ferenca 2006b; Alonso-Zarazaga 2009a.

*tremulae* **(Fabricius, 1787)**. Pileckis 1976a; Silfverberg 1992, 2004; Gaidienė 1993; Pileckis and Monsevičius 1997; Alonso-Zarazaga 2009a.

**Styphlini Jekel, 1861**.

*Orthochaetes* **Germar, 1824** = *Comasinus* Dejean, 1821.

[*setiger* **(Beck, 1817)**]. Known in Denmark, southern Sweden (Lundberg and Gustafsson 1995), northern Poland (Smreczyński 1972; Wanat and Mokrzycki 2005).

*Pseudostyphlus* **Tournier, 1874**.

*pillumus* **(Gyllenhal, 1835)**. Tamutis and Pankevičius 2001.

**Smicronychini Seidlitz, 1891**.

*Smicronyx* **Schönherr, 1843**.

*coecus* **(Reich, 1797)**. Tamutis and Pankevičius 2001; Vaivilavičius 2008.

[*jungermanniae* **(Reich, 1797)**]. Known in Denmark, southern Sweden (Lundberg and Gustafsson 1995), Belarus (Alexandrovitch et al. 1996), throughout Poland (Smreczyński 1972; Wanat and Mokrzycki 2005).

[*reichii* **(Gyllenhal, 1836)**]. Known in southern Sweden (Lundberg and Gustafsson 1995), Poland (Smreczyński 1972; Wanat and Mokrzycki 2005).

[*smreczynskii* **Solari, 1952** = *politus* Thomson, 1865, nec Boheman, 1843]. Known in Denmark, southern Sweden (Lundberg and Gustafsson 1995), Poland (Smreczyński 1972; Wanat and Mokrzycki 2005).

**Cionini Schönherr, 1825**.

*Cionus* **Clairville, 1798**.

*hortulanus* **(Geoffroy, 1785)**. Pileckis 1960, 1976a; Silfverberg 1992, 2004; Gaidienė 1993; Pileckis and Monsevičius 1997; Ferenca 2006b; Ivinskis et al. 2009; Šablevičius 2011.

*longicollis* **Brisout, 1863** = *montanus* Wingelmüller, 1914. Mazurowa and Mazur 1939; Pileckis 1960, 1976a; Silfverberg 1992, 2004; Pileckis and Monsevičius 1997.

*nigritarsis* **Retter, 1904**. Balalaikins et al. 2010.

[*olivieri **Rosenschöld, 1838**]. # 91. Tamutis 2003.

*scrophulariae* **(Linnaeus, 1758)**. Pileckis 1960, 1976a; Silfverberg 1992, 2004; Gaidienė 1993; Pileckis and Monsevičius 1997; Šablevičius 2000b, 2011; Ferenca 2006b; Vaivilavičius 2008, Balalaikins et al. 2010.

[*thapsus* **(Fabricius 1792)**]. Known northwestern Belarus (Alexandrovich et al. 1996), northeastern Poland (Smreczynski 1976), Denmark (Alonso-Zarazaga 2009a).

*tuberculosus* **(Scopoli, 1763)**. Pileckis 1960, 1976a; Mensonienė 1974; Silfverberg 1992, 2004; Gaidienė 1993; Pileckis and Monsevičius 1997; Šablevičius 2000b, 2011; Balalaikins et al. 2010.

*Stereonychus* **Suffrian, 1854**.

*fraxini* **(DeGeer, 1775).** Barševskis 2001a; Tamutis and Pankevičius 2001; Ferenca et al. 2002, 2006, 2007; Ferenca 2003; Šablevičius 2003a, 2011; Silfverberg 2004.

*Cleopus* **Dejean, 1821**.

*pulchellus* **(Herbst, 1795)**. Tamutis 2003.

*solani* **(Fabricius, 1792)**. Šablevičius 2003a.

**Tychiini Gistel, 1848**.

*Tychius* **Germar, 1817**.

*aureolus* **Kiesenwetter, 1851**.Alonso-Zarazaga 2009a.

*breviusculus* **Desbrochers, 1873**. Pileckis 1976a; Silfverberg 1992, 2004; Pileckis and Monsevičius 1997; Semaškienė et al. 1999; Žiogas and Zolubas 2005; Alonso-Zarazaga 2009a.

[*crassirostris* **Kirsch, 1871**]. Known in Denmark (Lundberg and Gustafsson 1995), Belarus (Alexandrovitch et al. 1996); Poland (Smreczyński 1972; Wanat and Mokrzycki 2005).

*junceus* **(Reich, 1797)**. Pileckis 1976a; Silfverberg 1992, 2004; Pileckis and Monsevičius 1997; Alonso-Zarazaga 2009a.

*lineatulus* **Stephens, 1831**. Tamutis 2003

*medicaginis* **Brisout, 1862**. Tamutis 2004; Alonso-Zarazaga 2009a.

*meliloti* **Stephens, 1831**. Pileckis 1976a; Silfverberg 1992, 2004; Pileckis and Monsevičius 1997; Alonso-Zarazaga 2009a.

*parallelus* **(Panzer, 1794)** = *venustus* (Fabricius, 1787) nec (Fabricius, 1781). Gaidienė 1993; Pileckis and Monsevičius 1997; Silfverberg 2004.

*picirostris* **(Fabricius, 1787)**. Heyden 1903; Ogyjewicz 1938; Pileckis 1960, 1976a; Mensonienė 1974; Silfverberg 1992, 2004; Pileckis and Monsevičius 1997; Alonso-Zarazaga 2009a.

*polylineatus* **(Germar, 1824)**. Pileckis and Monsevičius 1997; Silfverberg 2004; Alonso-Zarazaga 2009a.

*quinquepunctatus* **(Linnaeus, 1758)**. Mazurowa and Mazur 1939; Pileckis 1960, 1976a; Pileckis and Vengeliauskaitė 1977, 1996; Silfverberg 1992, 2004; Gaidienė 1993; Pileckis et al. 1994b; Pileckis and Monsevičius 1997; Alonso-Zarazaga 2009a.

*schneideri* **(Herbst, 1795)**. Pileckis and Monsevičius 1997; Ferenca 2004; Silfverberg 2004.

[*sharpi* **Tournier, 1873**]. Known in Estonia, Latvia (Silfverberg 2004), northwestern Belarus (Alexandrovitch et al. 1996), Poland (Smreczyński 1972; Wanat and Mokrzycki 2005).

*squamulatus* **Gyllenhal, 1836**= *flavicollis* auct. nec Stephens, 1831. Pileckis 1976a; Silfverberg 1992, 2004; Pileckis and Monsevičius 1997; Alonso-Zarazaga 2009a.

*stephensi* **Schönherr, 1836** = *tomentosus* (Herbst, 1795). Silfverberg 1992, 2004; Monsevičius 1997; Alonso-Zarazaga 2009a.

[*trivialis* **Boheman, 1843** = *kiesenwetteri* Tournier, 1873]. Known in Estonia (Lundberg and Gustafsson, 1995), Denmark (Silfverberg 2004), Poland (Smreczyński 1972; Wanat and Mokrzycki 2005).

*Sibinia* **Germar, 1817**.

*pellucens* **(Scopoli, 1772)**. Mensonienė 1974; Pileckis 1976a; Silfverberg 1992, 2004; Pileckis and Monsevičius 1997; Alonso-Zarazaga 2009a.

*phalerata* **(Gyllenhal, 1836)**. Pileckis and Monsevičius 1997; Silfverberg 2004.

*primita* **(Herbst, 1795)**. Šablevičius 2000a, 2003a.

*pyrrodactyla* **(Marsham, 1802)**= *potentillae* Germar, 1824. Pileckis 1976a; Silfverberg 1992, 2004; Monsevičius 1997; Pileckis and Monsevičius 1997; Šablevičius 2000b; Alonso-Zarazaga 2009a.

*sodalis* **Germar, 1824**. Tamutis 2003; Žiogas and Zolubas 2005.

[*subelliptica* **Desbrochers, 1868**]. Known in Latvia (Telnov et al. 2006), Belarus (Alexandrovitch et al. 1996), Poland (Smreczyński 1972; Wanat and Mokrzycki 2005).

*viscariae* **(Linnaeus, 1761)**. Miländer 1982; Silfverberg 1992, 2004; Monsevičius 1997; Pileckis and Monsevičius 1997; Alonso-Zarazaga 2009a.

**Acalyptini Thomson, 1859**.

*Acalyptus* **Schönherr, 1833**.

*carpini* **(Fabricius, 1787)**. Pileckis 1960, 1976a; Silfverberg 1992, 2004; Gaidienė 1993; Pileckis and Monsevičius 1997; Ferenca 2006b; Ostrauskas and Ferenca 2010.

[*sericeus* **Gyllenhal, 1836**]. Known in Estonia, Denmark, throughout Sweden (Lundberg and Gustafsson 1995), Belarus (Alexandrovitch et al. 1996), Poland (Smreczyński 1972; Wanat and Mokrzycki 2005).

**Anthonomini Thomson, 1859**.

*Anthonomus* **Germar, 1817** = *Furcipus* Desbrochers, 1868.

[*bituberculatus* **Thomson, 1868**]. Known in Estonia, Denmark, southern and central Sweden (Lundberg and Gustafsson 1995), Poland (Smreczyński 1972; Wanat and Mokrzycki 2005).

*brunnipennis* **Curtis, 1840**. Barševskis 2001a; Silfverberg 2004.

*conspersus* **Desbrochers, 1868**. Mastauskis 1970; Ferenca et al. 2002.

*humeralis* **(Panzer, 1794)**. Mastauskis 1970; Mensonienė 1974; Tamutis and Pankevičius 2001.

[*kirschi* **Desbrochers, 1868**]. Known in northwestern Belarus (Alexandrovitch et al. 1996), Poland (Smreczyński 1972; Wanat and Mokrzycki 2005).

*pedicularius* **(Linnaeus, 1758)**. Pileckis 1960, 1976a; Mastauskis 1970; Mensonienė 1981; Silfverberg 1992, 2004; Gaidienė 1993; Pileckis and Monsevičius 1997; Alonso-Zarazaga 2009a.

*phyllocola* **(Herbst, 1795)** = *varians* (Paykull, 1792) nec (Gmelin, 1790). Pileckis 1960, 1976a; Pileckis and Zajančkauskas 1968; Mastauskis 1970; Silfverberg 1992, 2004; Gaidienė 1993; Monsevičius 1997; Pileckis and Monsevičius 1997; Žiogas and Zolubas 2005; Ferenca 2006b; Alonso-Zarazaga 2009a.

*pinivorax* **(Silfverberg, 1977)** = *pubescens* (Paykull, 1792) nec (Fabricius, 1775). Pileckis 1960, 1976a; Pileckis et al. 1968; Mastauskis 1970; Silfverberg 1992, 2004; Pileckis and Monsevičius 1997; Alonso-Zarazaga 2009a.

*piri* **Kollar, 1837**. Pileckis 1959, 1960, 1976a; Mastauskis 1970; Silfverberg 1992, 2004; Pileckis and Monsevičius 1997; Alonso-Zarazaga 2009a.

*pomorum* **(Linnaeus, 1758)**. Ivanauskas and Vailionis 1922; Mastauskis 1925; Ogyjewicz 1929, 1931, 1932, 1938; Pileckis 1960, 1976a; Lešinskas and Pileckis 1967; Zajančkauskas and Pileckis 1968; Mastauskis 1970; Mensonienė 1974, 1981; Pileckis and Vengeliauskaitė 1977, 1996; Silfverberg 1992, 2004; Gaidienė 1993; Pileckis et al. 1994a; Monsevičius 1997; Pileckis and Monsevičius 1997; Šablevičius 2000b; Gliaudys 2001; Šurkus and Gaurilčikienė 2002; Alonso-Zarazaga 2009a; Ostrauskas and Ferenca 2010.

*rectirostris* **(Linnaeus, 1758)**. Heyden 1903; Pileckis 1960, 1976a; Lešinskas and Pileckis 1967; Zajančkauskas and Pileckis 1968; Mastauskis 1970; Pileckis and Vengeliauskaitė 1977, 1996; Silfverberg 1992, 2004; Gaidienė 1993; Pileckis et al. 1994a; Monsevičius 1997; Pileckis and Monsevičius 1997 (*Furcipes*); Gliaudys 2001; Ferenca 2006b; Alonso-Zarazaga 2009a.

*rubi* **(Herbst, 1795)**. Ogijewicz 1929, 1931; Pileckis 1960, 1976a; Lešinskas and Pileckis 1967; Zajančkauskas and Pileckis 1968; Mastauskis 1970; Mensonienė 1974; Pileckis and Vengeliauskaitė 1977, 1996; Silfverberg 1992, 2004; Gaidienė 1993; Pileckis et al. 1994a; Monsevičius 1997; Pileckis and Monsevičius 1997; Šablevičius 2000b; Gliaudys 2001; Vaivilavičius 2008; Alonso-Zarazaga 2009a.

*rufus* **Gyllenhal, 1836**. Bercio and Folwaczny 1979; Silfverberg 1992, 2004; Alonso-Zarazaga 2009a.

[*sorbi* **Germar, 1821**]. Known in Latvia (Telnov 2004), Estonia, Denmark, southern Sweden (Lundberg and Gustafsson 1995), Poland (Smreczyński 1972; Wanat and Mokrzycki 2005).

*ulmi* **(DeGeer, 1775)**. Silfverberg 1992, 2004; Alonso-Zarazaga 2009a.

*undulatus* **Gyllenhal, 1836**. Tamutis and Pankevičius 2001.

*Brachonyx* **Schönherr, 1825**.

*pineti* **(Paykull, 1792)**. Pileckis 1960, 1976a; Pileckis et al. 1968; Silfverberg 1992, 2004; Gaidienė 1993; Žiogas and Gedminas 1994; Monsevičius 1997; Pileckis and Monsevičius 1997; Žiogas and Zolubas 2005; Ferenca 2006b; Alonso-Zarazaga 2009a; Šablevičius 2011.

*Bradybatus* **Germar, 1824**.

*kellneri* **Bach, 1854**. Tenenbaum 1931; Pileckis 1968b,1976a; Silfverberg 1992, 2004; Pileckis and Monsevičius 1997; Ferenca 2004; Alonso-Zarazaga 2009a.

**Curculionini Latreille, 1802**.

*Curculio* **Linnaeus, 1758**.

*betulae* **(Stephens, 1831)** = *cerasorum* Paykull, 1792, nec Fabricius, 1775. Pileckis 1968a, 1976a; Silfverberg 1992, 2004; Pileckis and Monsevičius 1997; Alonso-Zarazaga 2009a.

*glandium* **Marsham, 1802**. Pileckis 1959, 1960, 1976a; Lešinskas and Pileckis 1967; Pileckis et al. 1968; Silfverberg 1992, 2004; Pileckis and Monsevičius 1997; Žiogas 1997; Gliaudys Šablevičius 2000b; 2001, 2011; Alonso-Zarazaga 2009a; Vaivilavičius 2008.

*nucum* **Linnaeus, 1758**. Eichwald 1830; Heyden 1903; Ogyjewicz 1938; Pileckis 1960, 1976a; Lešinskas and Pileckis 1967; Zajančkauskas and Pileckis 1968; Silfverberg 1992, 2004; Gaidienė 1993; Pileckis et al. 1994a; Monsevičius 1997; Pileckis and Monsevičius 1997; Žiogas 1997; Šablevičius 2000b, 2011; Gliaudys 2001; Ferenca 2006b; Alonso-Zarazaga 2009a.

*rubidus* **(Gyllenhal, 1836)**. Miländer 1982; Silfverberg 1992, 2004; Gaidienė 1993; Pileckis and Monsevičius 1997; Alonso-Zarazaga 2009a.

*venosus* **(Gravenhorst, 1807)**. Pileckis 1976a; Silfverberg 1992, 2004; Pileckis and Monsevičius 1997; Alonso-Zarazaga 2009a

*villosus* **Fabricius, 1781**. Pileckis 1959, 1960, 1976a; Mensonienė 1974; Silfverberg 1992, 2004; Gaidienė 1993; Pileckis and Monsevičius 1997; Alonso-Zarazaga 2009a.

*Archarius* **Gistel, 1848**.

*crux* **(Fabricius, 1777)**. Heyden 1903; Pileckis 1960, 1976a; Silfverberg 1992, 2004; Gaidienė 1993; Pileckis and Monsevičius 1997 (*Curculio*); Vaivilavičius 2008; Alonso-Zarazaga 2009a; Šablevičius 2011.

*pyrrhoceras* **(Marsham, 1802)**. Roubal 1910; Pileckis 1959, 1960, 1976a; Silfverberg 1992, 2004; Pileckis and Monsevičius 1997 (*Curculio*); Alonso-Zarazaga 2009a.

*salicivorus* **(Paykull, 1792)**. Heyden 1903; Pileckis 1960, 1976a; Silfverberg 1992, 2004; Gaidienė 1993; Monsevičius 1997; Pileckis and Monsevičius 1997 (*Curculio*); Šablevičius 2000b; Ferenca 2006b; Vaivilavičius 2008; Alonso-Zarazaga 2009a.

**Rhampihini Rafinesque, 1815**.

*Orchestes* **Illiger, 1804**.

*alni* **(Linnaeus, 1758)** = *saltator* (Geoffroy, 1785). Pileckis 1976a; Silfverberg 1992, 2004; Gaidienė 1993; Pileckis and Monsevičius 1997 (*Rhynchaenus*).

*avellanae* **(Donovan, 1797) nec (Paykull, 1792)** = *signifer* (Creutzer, 1799). Pileckis 1962, 1963b, 1976a; Silfverberg 1992, 2004; Pileckis and Monsevičius 1997 (*Rhynchaenus*).

*betuleti* **(Panzer, 1795)** = *rufus* (Schrank, 1781). Pileckis 1976a; Silfverberg 1992, 2004; Pileckis and Monsevičius 1997 (*Rhynchaenus*).

[*fagi* **(Linnaeus, 1758)**]. Known in Latvia (Telnov 2004), Estonia, Denmark, southern Sweden (Lundberg and Gustafsson 1995), Kaliningrad region (Bercio and Folwaczny 1979), Poland (Smreczyński 1976; Wanat and Mokrzycki 2005).

*jota* **(Fabricius, 1787)**. Pileckis 1960, 1976a; Silfverberg 1992, 2004; Gaidienė 1993; Pileckis and Monsevičius 1997 (*Rhynchaenus*); Ferenca 2006b.

*pilosus* **(Fabricius, 1781)**. Miländer 1982; Gaidienė 1993; Pileckis and Monsevičius 1997 (*Rhynchaenus*); Šablevičius 2003a; Silfverberg 2004.

*quercus* **(Linnaeus, 1758)**. Pileckis et al. 1968; Pileckis and Monsevičius 1997 (*Rhynchaenus*); Šablevičius 2000a, 2011; Silfverberg 2004.

*rusci* **(Herbst, 1795)**. Pileckis 1976a; Silfverberg 1992, 2004; Monsevičius 1997; Pileckis and Monsevičius 1997 (*Rhynchaenus*); Šablevičius 2000b.

*testaceus* **(O.F. Müller, 1776)** *= calceatus* (Germar, 1821). # 92. Ogyjewicz 1938; Pileckis 1960, 1976a; Silfverberg 1992, 2004; Pileckis and Monsevičius 1997 (*Rhynchaenus*); Tamutis 2004; Ferenca 2006b; Vaivilavičius 2008.

*Rhynchaenus* **Clairville, 1798**.

*xylostei* **Clairville, 1798** = *lonicerae* Herbst, 1795. Roubal 1910; Pileckis 1960, 1976a; Silfverberg 1992, 2004; Gaidienė 1993; Pileckis and Monsevičius 1997; Šablevičius 2000b, 2011; Ferenca 2006b.

*Pseudorchestes* **Bedel, 1894**.

*cinereus **(Fåhraeus, 1843)**. Pileckis 1976a; Silfverberg 1992, 2004; disproved by Pileckis and Monsevičius (1997) (*Rhynchaenus*).

*pratensis* **(Germar, 1821)**. Pileckis 1968b, 1976a; Zajančkauskas and Pileckis 1968; Silfverberg 1992, 2004; Monsevičius 1997; Pileckis and Monsevičius 1997 (*Rhynchaenus*).

*Tachyerges* **Schönherr, 1826**.

*decoratus* **(Germar, 1821)**. Pileckis 1968b, 1976a; Zajančkauskas and Pileckis 1968; Silfverberg 1992, 2004; Gaidienė 1993; Monsevičius 1997; Pileckis and Monsevičius 1997 (*Rhynchaenus*).

[*pseudostigma* **(Tempère, 1982)**]. Known in Latvia (Telnov 2004), Denmark, Sweden (Lundberg and Gustafsson 1995), Poland (Alonso-Zarazaga 2009a).

*rufitarsis* **(Germar, 1821)**. Pileckis 1976a; Silfverberg 1992, 2004; Pileckis and Monsevičius 1997 (*Rhynchaenus*); Ivinskis et al. 2009.

*salicis* **(Linnaeus, 1758)**. Pileckis 1960, 1976a; Zajančkauskas and Pileckis 1968; Pileckis et al. 1968; Silfverberg 1992, 2004; Gaidienė 1993; Monsevičius 1997; Pileckis and Monsevičius 1997 (*Rhynchaenus*); Šablevičius 2000b, 2011; Vaivilavičius 2008.

*stigma* **(Germar, 1821)**. Pileckis 1960, 1976a; Zajančkauskas and Pileckis 1968; Silfverberg 1992, 2004; Gaidienė 1993; Monsevičius 1997; Pileckis and Monsevičius 1997 (*Rhynchaenus*); Šablevičius 2003b; Vaivilavičius 2008.

*Isochnus* **Thomson, 1859**.

[*angustifrons* **(West, 1916)**]. Known in Estonia, Denmark (Lundberg and Gustafsson 1995), Poland (Smreczyński 1976; Wanat and Mokrzycki 2005).

*foliorum* **(O.F. Müller, 1764)** = *saliceti* (Paykull, 1792). Pileckis 1960, 1976a; Silfverberg 1992, 2004; Gaidienė 1993; Pileckis and Monsevičius 1997 (*Rhynchaenus*).

*sequensi* **(Stierlin, 1894)** = *populicola* (Silfverberg, 1977) = *populi* (Fabricius, 1793) nec (Linnaeus, 1758). Pileckis 1960, 1976a; Lešinskas and Pileckis 1967; Silfverberg 1992, 2004; Gaidienė 1993; Monsevičius 1997; Pileckis and Monsevičius 1997 (*Rhynchaenus*); Šablevičius 2000b; Ferenca 2006b.

*Rhamphus* **Clairville & Schellenberg, 1798**.

[*oxyacanthae* **(Marsham, 1802)**]. Known in Latvia (Telnov 2004), Denmark, southern Sweden (Lundberg and Gustafsson 1995), Poland (Smreczyński 1976; Wanat and Mokrzycki 2005).

*pulicarius* **(Herbst, 1795)**. Pileckis 1968b, 1976a; Zajančkauskas and Pileckis 1968; Silfverberg 1992, 2004; Gaidienė 1993; Monsevičius 1997; Pileckis and Monsevičius 1997; Vaivilavičius 2008; Ostrauskas and Ferenca 2010.

*subaeneus* **(Illiger, 1807)**. Pileckis and Monsevičius 1997; Silfverberg 2004.

**Mecinini Gistel, 1848**.

*Gymnetron* **Schönherr, 1825**.

*beccabungae* **(Linnaeus, 1761)**. Gaidienė 1993; Silfverberg 2004.

*melanarium* **(Germar, 1821)**. Pileckis 1976a; Silfverberg 1992, 2004; Pileckis and Monsevičius 1997; Alonso-Zarazaga 2009a.

[*rostellum* **(Herbst, 1795)**]. Known in Latvia (Telnov 2004), Estonia, Denmark, southern Sweden (Lundberg and Gustafsson 1995), Poland (Smreczyński 1976; Wanat and Mokrzycki 2005).

[*stimulosum* **(Germar, 1821)**]. Known in Latvia (Telnov 2004), Poland (Smreczyński 1976; Wanat and Mokrzycki 2005).

*veronicae* **(Germar, 1821)**. Tamutis and Pankevičius 2001.

*villosulum* **Gyllenhal, 1838**. Pileckis and Monsevičius 1997; Silfverberg 2004.

*Rhinusa* **Stephens, 1829**.

**antirrhini (Paykull, 1800).** Pileckis 1976a; Silfverberg 1992, 2004; Gaidienė 1993; Monsevičius 1997; Pileckis and Monsevičius 1997 (Gymnetron); Alekseev 2008a; Alonso-Zarazaga 2009a.

*collina* **(Gyllenhal, 1813)**. Tamutis and Pankevičius 2001.

[*hispida* **(Brullé, 1832)**]. Known in Denmark, Sweden (Lundberg and Gustafsson 1995), Poland (Smreczyński 1976; Wanat and Mokrzycki 2005).

*linariae* **(Panzer, 1792)**. Barševskis 2001a; Tamutis and Pankevičius 2001; Silfverberg 2004.

*neta* **(Germar, 1821)**. Bercio and Folwaczny 1979; Silfverberg 1992, 2004.

*tetra* **(Fabricius, 1792)**. Pileckis 1960, 1976a; Silfverberg 1992, 2004; Gaidienė 1993; Monsevičius 1997; Pileckis and Monsevičius 1997 (*Gymnetron*).

*thapsicola* **(Germar, 1821)**. Silfverberg 1992, 2004; Alonso-Zarazaga 2009a.

*Mecinus* **Germar, 1821**.

*collaris* **Germar, 1821**. Lentz 1879; Pileckis 1968b, 1976a; Silfverberg 1992, 2004; Gaidienė 1993; Pileckis and Monsevičius 1997.

[*heydeni* **Wencker, 1866**]. Known in Latvia (Telnov 2004), Denmark, southern Sweden (Lundberg and Gustafsson 1995), Belarus (Alexandrovitch et al. 1996), Poland (Smreczyński 1976; Wanat and Mokrzycki 2005).

[*janthinus* **Germar, 1821**]. Known in Latvia (Telnov 2004), Sweden (Lundberg and Gustafsson 1995), Poland (Smreczyński 1976; Wanat and Mokrzycki 2005).

*labilis* **(Herbst, 1795)**. Miländer 1982; Silfverberg 1992, 2004; Monsevičius 1997; Pileckis and Monsevičius 1997; Alonso-Zarazaga 2009a.

*pascuorum* **(Gyllenhal, 1813)**. Pileckis 1968a, 1976a; Silfverberg 1992, 2004; Monsevičius 1997; Pileckis and Monsevičius 1997; Alonso-Zarazaga 2009a.

*pyraster* **(Herbst, 1795)**. Roubal 1910; Mazurowa and Mazur 1939; Pileckis 1960, 1976a; Silfverberg 1992, 2004; Gaidienė 1993; Pileckis and Monsevičius 1997; Ferenca 2006b; Alonso-Zarazaga 2009a.

*Miarus* **Schönherr, 1826**.

*campanulae* **(Linnaeus, 1767)** = *frigidus* Franz, 1947 = *fennicus* Kangas, 1978. # 93. Pileckis 1960, 1976a; Silfverberg 1992, 2004; Gaidienė 1993; Pileckis and Monsevičius 1997; Šablevičius 2000b, 2011; Vaivilavičius 2008; Alonso-Zarazaga 2009a.

*Cleopomiarus* **Pierce, 1919** = *Miaromimus* Solari, 1947.

*distinctus* **(Boheman, 1845)**. Gaidienė 1993; Šablevičius 2000b; Tamutis 2003; Silfverberg 2004.

*graminis* **(Gyllenhal, 1813)**. Gaidienė 1993; Pileckis and Monsevičius 1997 (*Miarus*); Šablevičius 2000b, 2011; Silfverberg 2004.

*micros* **(Germar, 1821)**. Tamutis and Pankevičius 2001; Ferenca 2004; Žiogas and Zolubas 2005.

**Bagoinae** **Thomson, 1859**. # 94.

**Bagoini Thomson, 1859**.

*Bagous* **Germar, 1817**.

*alismatis* **(Marsham, 1802)**. Tamutis and Pankevičius 2001; Silfverberg 2004.

[*argillaceus* **Gyllenhal, 1836**].Known in Estonia, Denmark, southern Sweden (Lundberg and Gustafsson 1995), Poland (Smreczyński 1972; Wanat and Mokrzycki 2005).

*binodulus* **(Herbst, 1795)**. Pileckis 1960, 1976a; Silfverberg 1992, 2004; Gaidienė 1993; Pileckis and Monsevičius 1997; Ferenca 2006b; Alonso-Zarazaga 2009a.

[*brevis* **Gyllenhal, 1836**]. Known in Denmark, Sweden (Lundberg and Gustafsson 1995), Poland (Wanat and Mokrzycki 2005).

[*claudicans* **Boheman, 1845**]. Known in southern Sweden (Lundberg and Gustafsson 1995), Poland (Wanat and Mokrzycki 2005).

[*colligensis* **(Herbst, 1797)**]. Known in Latvia (Vorst et al. 2007), Denmark, Sweden (Lundberg and Gustafsson 1995), throughout Poland (Smreczyński 1972; Wanat and Mokrzycki 2005).

*czwalinai* **Seidlitz, 1891**. Tamutis 2003.

[*diglyptus* **Boheman, 1845** = *curtus* Gyllenhal, 1845 = *brevitarsis* Hansen, 1917]. Known in Estonia, Denmark, Sweden (Lundberg and Gustafsson 1995), Poland (Smreczyński 1972; Wanat and Mokrzycki 2005).

*elegans* **(Fabricius, 1801)**. Gaidienė 1993; Pileckis and Monsevičius 1997 (*Dicranthus*); Silfverberg 2004; Ferenca 2006a.

*frit* **(Herbst, 1795)**. Tamutis et al. 2008.

[*frivaldszkyi* **Tournier, 1874**]. Known in Estonia (Lundberg and Gustafsson 1995), northern Belarus (Alexandrovitch et al. 1996), Poland (Smreczyński 1972; Wanat and Mokrzycki 2005).

*glabirostris* **(Herbst, 1795)**. Tamutis and Pankevičius 2001.

[*limosus* **(Gyllenhal, 1827)**].Known in Estonia, Denmark, Sweden (Lundberg and Gustafsson 1995), Poland (Smreczyński 1972; Wanat and Mokrzycki 2005).

*longitarsis* **Thomson, 1868**. Tamutis 2003.

*lutosus* **(Gyllenhal, 1813)**. Pileckis 1960, 1976a; Silfverberg 1992, 2004; Gaidienė 1993; Pileckis and Monsevičius 1997; Ferenca 2004, 2006b; Ferenca et al. 2006, 2007; Alonso-Zarazaga 2009a.

*lutulentus* **(Gyllenhal, 1813)** = *nigritarsis* Thomson, 1865. Gaidienė 1993; Pileckis and Monsevičius 1997; Šablevičius 2000b; Silfverberg 2004.

[*lutulosus* **(Gyllenhal, 1827)**]. Known in Latvia (Telnov 2004), Estonia, Denmark, Sweden (Lundberg and Gustafsson 1995), Belarus (Alexandrovitch et al. 1996), throughout Poland (Smreczyński 1972; Wanat and Mokrzycki 2005).

[*nodulosus* **Gyllenhal, 1836**]. Known in Estonia, Denmark, Sweden (Lundberg and Gustafsson 1995), Belarus (Alexandrovitch et al. 1996), Poland (Smreczyński 1972; Wanat and Mokrzycki 2005).

[*petro* **(Herbst, 1795)**]. Known in Latvia (Telnov 2004), Estonia, Denmark, Sweden (Lundberg and Gustafsson 1995), Belarus (Alexandrovitch et al. 1996), Poland (Smreczyński 1972; Wanat and Mokrzycki 2005).

[*puncticollis* **Boheman, 1845**]. Known in Latvia (Vorst et al. 2007), Denmark, Sweden (Lundberg and Gustafsson 1995), Belarus (Alexandrovitch et al. 1996), Poland (Smreczyński 1972; Wanat and Mokrzycki 2005).

[*robustus* **Brisout, 1863**]. Known in Denmark, Sweden (Silfverberg 2004), Poland (Smreczyński 1972; Wanat and Mokrzycki 2005).

*subcarinatus* **Gyllenhal, 1813**. Tamutis and Pankevičius 2001.

*tempestivus* **(Herbst, 1795)** = *thomsoni* Bruce, 1968. Tamutis and Pankevičius 2001.

*tubulus* **Caldara and O’Brien, 1994** = *angustus* Silfverberg, 1977, nec Tanner, 1954 = *cylindrus* (Paykull, 1800) nec (Fabricius, 1781). Pileckis and Monsevičius 1997; Silfverberg 2004.

**Baridinae** **Schönherr, 1836**.

**Baridini Schönherr, 1836**.

*Baris* **Germar, 1817**.

*artemisiae* **(Herbst, 1795)**. Pileckis 1960, 1976a; Silfverberg 1992, 2004; Gaidienė 1993; Pileckis and Monsevičius 1997; Šablevičius 2000b; Vaivilavičius 2008.

[*spitzyi* **Hochhuth, 1847**]. Known in Estonia (Silfverberg 2004), Poland Smreczyński 1974; Wanat and Mokrzycki 2005).

*Melanobaris* **Alonso-Zarazaga & Lyal, 1999**.

*carbonaria* **(Boheman, 1836)**. Pileckis 1976a; Pileckis et al. 1983; Silfverberg 1992, 2004; Pileckis and Monsevičius 1997 (*Baris*).

*laticollis* **(Marsham, 1802)**. Pileckis 1960, 1976a; Pileckis et al. 1983; Silfverberg 1992, 2004; Gaidienė 1993; Pileckis and Monsevičius 1997 (*Baris*); Ferenca 2006b.

*Aulacobaris* **Desbrochers, 1892**.

*coerulescens* **(Scopoli, 1763)**. Pileckis 1976a; Silfverberg 1992, 2004; Pileckis and Monsevičius 1997 (*Baris*).

*lepidii* **(Germar, 1824)**. Pileckis 1976a; Silfverberg 1992, 2004; Pileckis and Monsevičius 1997 (*Baris*).

**Apostasimerini Schönherr, 1844**.

*Limnobaris* **Bedel, 1885**.

*pilistriata* **(Stephens, 1831)** = *dolorosa* (Goeze, 1777). Pileckis 1968b, 1976a; Zajančkauskas and Pileckis 1968; Silfverberg 1992, 2004; Gaidienė 1993; Monsevičius 1997; Pileckis and Monsevičius 1997; Ivinskis et al. 2009.

*t-album* **(Linnaeus, 1758)**. Pileckis 1960, 1976a; Zajančkauskas and Pileckis 1968; Silfverberg 1992, 2004; Gaidienė 1993; Monsevičius 1997; Pileckis and Monsevičius 1997; Šablevičius 2000b; Tamutis 2003; Ferenca 2006b; Alonso-Zarazaga 2009a.

**Ceutorhynchinae** **Gistel, 1848**.

**Mononychini LeConte, 1876**.

*Mononychus* **Germar, 1824**.

*punctumalbum* **(Herbst, 1784)**. Pileckis 1960, 1976a; Silfverberg 1992, 2004; Gaidienė 1993; Pileckis and Monsevičius 1997; Šablevičius 2000b; Ferenca 2006b; Vaivilavičius 2008.

**Phytobiini Gistel, 1848**.

*Eubrychius* **Thomson, 1859**.

*velutus* **(Beck, 1817)**. Tamutis and Pankevičius 2001.

*Phytobius* **Schönherr, 1833** = *Litodactylus* Redtenbacher, 1845.

*leucogaster* **(Marsham, 1802)**. Tamutis and Pankevičius 2001; Ferenca et al. 2002.

*Pelenomus* **Thomson, 1859**.

*canaliculatus* **(Fåhraeus, 1843)**. Pileckis 1968b, 1976a; Zajančkauskas and Pileckis 1968; Silfverberg 1992, 2004; Gaidienė 1993; Monsevičius 1997; Pileckis and Monsevičius 1997 (*Phytobius*); Alonso-Zarazaga 2009a.

*commari* **(Herbst, 1795)**. Bercio and Folwaczny 1979; Silfverberg 1992, 2004.

[*olssoni* **(Israelson, 1972)**]. Known in Denmark, southern Sweden (Lundberg and Gustafsson 1995), Poland (Wanat and Mokrzycki 2005).

*quadricorniger* **(Colonnelli, 1986)** = *quadricornis* (Gyllenhal, 1827) nec (Paykull, 1792). Pileckis 1960, 1976a; Silfverberg 1992, 2004; Gaidienė 1993; Pileckis and Monsevičius 1997 (*Phytobius*); Ferenca 2006b.

*quadrituberculatus* **(Fabricius, 1787)**. Pileckis 1968b, 1976a; Zajančkauskas and Pileckis 1968; Silfverberg 1992, 2004; Gaidienė 1993; Monsevičius 1997; Pileckis and Monsevičius 1997 (*Phytobius*); Alonso-Zarazaga 2009a.

*velaris* **(Gyllenhal, 1827)**. Tamutis 2004.

*waltoni* **(Boheman, 1843)**. Gaidienė 1993; Monsevičius 1997; Pileckis and Monsevičius 1997 (*Phytobius*); Silfverberg 2004.

*Neophytobius* **Wagner, 1936**.

*muricatus* **(Brisout, 1867)**. Pileckis 1976a; Silfverberg 1992, 2004; Pileckis and Monsevičius 1997 (*Phytobius*).

*quadrinodosus* **(Gyllenhal, 1813)**. Tamutis and Pankevičius 2001; Ivinskis et al. 2009.

*Rhinoncus* **Schönherr, 1825**.

*albicinctus* **Gyllenhal, 1837**. Tamutis and Pankevičius 2001.

*bruchoides* **(Herbst, 1784)**. Roubal 1910; Pileckis 1960, 1963b, 1976a; Silfverberg 1992, 2004; Gaidienė 1993; Pileckis and Monsevičius 1997; Ferenca 2006b.

*castor* **(Fabricius, 1792)**. Roubal 1910; Pileckis 1960, 1976a; Zajančkauskas and Pileckis 1968; Mensonienė 1974; Silfverberg 1992, 2004; Gaidienė 1993; Monsevičius 1997; Pileckis and Monsevičius 1997; Šablevičius 2000b; Ferenca 2006b.

[*henningsi* **Wagner, 1929**]. Known in northwestern Belarus (Alexandrovitch et al. 1996), Poland (Smreczyński 1974; Wanat and Mokrzycki 2005).

*inconspectus* **(Herbst, 1795)** = *gramineus* (Fabricius, 1793) nec (Gmelin, 1790). Pileckis 1960, 1963b, 1976a; Zajančkauskas and Pileckis 1968; Silfverberg 1992, 2004; Gaidienė 1993; Monsevičius 1997; Pileckis and Monsevičius 1997; Ferenca 2006b.

*periciparius* **(Linnaeus, 1758)**. Pileckis 1968b, 1976a; Zajančkauskas and Pileckis 1968; Silfverberg 1992, 2004; Gaidienė 1993; Monsevičius 1997; Pileckis and Monsevičius 1997; Alonso-Zarazaga 2009a.

*perpendicularis* **(Reich, 1797)**. Silfverberg 1992, 2004; Pileckis and Monsevičius 1997; Šablevičius 2000b; Alonso-Zarazaga 2009a.

*Marmaropus* **Schönherr, 1825**.

*besseri* **Gyllenhal, 1837**. Tamutis and Pankevičius 2001; Ferenca et al. 2002.

**Scleropterini Schultze, 1902**.

*Rutidosoma* **Stephens, 1831**.

[*fallax* **(Otto, 1897)**]. Known in Latvia (Telnov 2004), Denmark, southern Sweden (Lundberg and Gustafsson 1995), Poland (Wanat and Mokrzycki 2005).

*globulus* **(Herbst, 1795)**. Pileckis 1976a; Silfverberg 1992, 2004; Pileckis and Monsevičius 1997.

*Scleropterus* **Schönherr, 1825**.

*serratus* **(Germar, 1824)**. Slavinskas 1982; Silfverberg 1992, 2004; Pileckis and Monsevičius 1997; Šablevičius 2000b; Šablevičius 2003a; Ferenca 2004; Vaivilavičius 2008.

**Cnemogonini Colonnelli, 1979**.

*Auletes* **Dietz, 1896**.

*epilobii* **(Paykull, 1800)**. Šablevičius 2000a, 2001, 2003a, b; Tamutis and Pankevičius 2001; Ferenca et al. 2002; Alonso-Zarazaga 2009a.

**Ceutorhynchini Gistel, 1848**.

*Amalus* **Schönherr, 1825**.

*scortillum* **(Herbst, 1795)** = *haemorrhous* (Herbst, 1795) nec (Gmelin, 1790). Silfverberg 1992, 2004; Ferenca et al. 2002.

*Amalorrhynchus* **Reitter, 1913**.

*melanarius* **(Stephens, 1831)**. Tamutis 2003.

*Poophagus* **Schönherr, 1837**.

*sisymbrii* **(Fabricius, 1776)**. Gaidienė 1993; Monsevičius 1997; Silfverberg 2004.

*Tapeinotus* **Schönherr, 1826**.

*sellatus* **(Fabricius, 1794)**. Silfverberg 1992, 2004; Gaidienė 1993; Monsevičius 1997; Pileckis and Monsevičius 1997; Šablevičius 2000b, 2003b, 2011; Vaivilavičius 2008.

*Coeliodes* **Schönherr, 1837**.

*nana* **(Fabricius, 1787)** = *dryados* (Gmelin, 1790) = *quercus* (Fabricius, 1787) nec (Linnaeus, 1758). Roubal 1910; Pileckis 1959, 1960, 1976a; Silfverberg 1992, 2004; Pileckis and Monsevičius 1997; Ferenca 2006b.

*nigritarsis* **(Hartmann, 1895)**. Pileckis 1968b; 1976a; Zajančkauskas and Pileckis 1968; Silfverberg 1992, 2004; Gaidienė 1993; Monsevičius 1997; Pileckis and Monsevičius 1997.

*ruber* **(Marsham, 1802)**. Pileckis 1962, 1963b, 1976a; Pileckis and Monsevičius 1997; Šablevičius 2000a; Silfverberg 1992, 2004.

*rubicundus* **(Herbst, 1795)**. Pileckis 1960, 1976a; Silfverberg 1992, 2004; Pileckis and Monsevičius 1997; Žiogas and Zolubas 2005; Ferenca 2006b.

*transversealbofasciatus* **(Goeze, 1777)** = *erythroleucos* (Gmelin, 1790) = *cinctus* (Geoffroy, 1785) nec (Drury, 1782). Pileckis 1976a; Silfverberg 1992, 2004; Gaidienė 1993; Pileckis and Monsevičius 1997; Šablevičius 2000a, 2003a.

[*trifasciatus* **Bach, 1854**]. Known in Latvia (Telnov 2004), Estonia (Lundberg and Gustafsson 1995), northwestern Belarus (Alexandrovitch et al. 1996), Poland (Smreczyński 1974; Wanat and Mokrzycki 2005).

*Thamiocolus* **Thomson, 1859**.

*kraatzi* **(Brisout, 1869)**. Tamutis 1996.

[*pubicollis* **(Gyllenhal, 1837)**]. Known in northwestern Belarus (Alexandrovitch et al. 1996), Poland (Smreczyński 1974; Wanat and Mokrzycki 2005).

*sahlbergi* **(R.F. Sahlberg, 1845)**. Tamutis and Pankevičius 2001.

*viduatus* **(Gyllenhal, 1813)**. Tamutis 1996.

*Micrelus* **Thomson, 1859**.

*ericae* **(Gyllenhal, 1813)**. Pileckis 1960, 1976a; Silfverberg 1992, 2004; Pileckis and Monsevičius 1997; Ferenca 2006b; Alonso-Zarazaga 2009a.

*Trichosirocalus* **Colonnelli, 1979** = *Ceuthorhynchidius* auct. nec Jacquelin du Val, 1854.

*barnevillei* **(Grenier, 1866)**. Pileckis and Monsevičius 1997 (*Ceuthorhynchidius*); Silfverberg 2004.

[*horridus* **(Panzer, 1801)**]. Known in northwestern Belarus (Alexandrovitch et al. 1996), northern Poland (Smreczyński 1974; Wanat and Mokrzycki 2005).

[*thalhammeri* **(Schultze, 1906)**]. Known in Denmark, Sweden (Lundberg and Gustafsson 1995).

*troglodytes* **(Fabricius, 1787)**. Pileckis 1976a; Silfverberg 1992, 2004; Pileckis and Monsevičius 1997 (*Ceuthorhynchidius*); Ferenca et al. 2002; Šablevičius 2003a; Alonso-Zarazaga 2009a.

*Zacladus* **Reitter, 1913**.

*geranii* **(Paykull, 1800)** = *affinis* (Paykull, 1792) nec (Schrank, 1781). Gaidienė 1993; Pileckis and Monsevičius 1997; Šablevičius 2000b; Ferenca et al. 2002; Silfverberg 2004; Vaivilavičius 2008; Alonso-Zarazaga 2009a.

*Nedyus* **Schönherr, 1825** = *Cidnorhinus* Thomson, 1869.

*quadrimaculatus* **(Linnaeus, 1758)**. Pileckis 1960, 1976a; Zajančkauskas and Pileckis 1968; Silfverberg 1992, 2004; Gaidienė 1993; Monsevičius 1997; Pileckis and Monsevičius 1997; Šablevičius 2000b, 2011; Ferenca 2006b; Vaivilavičius 2008; Alonso-Zarazaga 2009a.

*Coeliastes* **Weise, 1883**.

*lamii* **(Fabricius, 1792)**. Silfverberg 1992, 2004; Vaivilavičius 2008.

*Sirocalodes* **Voss, 1958**. # 335.

*depressicollis* **(Gyllenhal, 1813)** = *nigrinus* (Marsham, 1802) nec (Herbst, 1795). Tamutis 1996; Pileckis and Monsevičius 1997 (*Ceutorhynchus*); Silfverberg 2004; Alonso-Zarazaga 2009a.

*quercicola* **(Paykull, 1792)**. Pileckis 1976a; Silfverberg 1992, 2004; Tamutis 1996; Pileckis and Monsevičius 1997 (*Ceutorhynchus*).

*Calosirus* **Thomson, 1859**.

*apicalis* **(Gyllenhal, 1827)**. Tamutis 1996.

*terminatus* **(Herbst, 1795)**. Tamutis 1996; Alonso-Zarazaga 2009a.

*Ceutorhynchus* **Germar, 1824** = *Ceuthorhynchidius* Jacquelin du Val, 1854 = *Neosirocalus* Wagner, 1944.

[*alliariae* **Brisout, 1860**]. Known in Denmark, Sweden (Silfverberg 2004), Poland (Smreczyński 1974; Wanat and Mokrzycki 2005).

[*atomus* **Boheman, 1845**]. Known in Denmark, Sweden (Lundberg and Gustafsson 1995), throughout Poland (Smreczyński 1974; Wanat and Mokrzycki 2005).

*barbareae* **Suffrian, 1847**. Tamutis 1996; Ferenca et al. 2002.

*cakilis* **(Hansen, 1917)**. Ferenca and Tamutis 2009.

*chalibaeus* **Germar, 1824**. Silfverberg 1992, 2004; Tamutis 1996; Ferenca et al. 2002; Alonso-Zarazaga 2009a.

*cochleariae* **(Gyllenhal, 1813)**. Pileckis 1960, 1976a; Silfverberg 1992, 2004; Pileckis and Monsevičius 1997; Tamutis 1996; Ferenca 2006b; Alonso-Zarazaga 2009a.

[*constrictus* **(Marsham, 1802)**]. Known in Denmark, southern Sweden (Lundberg and Gustafsson 1995), Belarus (Alexandrovitch et al. 1996), Poland (Smreczyński 1974; Wanat and Mokrzycki 2005).

*dubius* **Brisout, 1883**. Tamutis and Pankevičius 2001; Alonso-Zarazaga 2009a.

*erysimi* **(Fabricius, 1787)**. Roubal 1910; Pileckis 1960, 1976a; Mensonienė 1974; Silfverberg 1992, 2004; Gaidienė 1993; Tamutis 1996; Pileckis and Monsevičius 1997; Vaivilavičius 2008; Alonso-Zarazaga 2009a.

*gallorhenanus* **Hoffmann, 1954**. Tamutis 1996.

*granulicollis* **Thomson, 1865** = *gerhardti* Schultze, 1899. Tamutis 1996.

[*griseus* **Brisout, 1869**]. Known in Denmark, southern Sweden (Lundberg and Gustafsson 1995), Belarus (Alexandrovitch et al. 1996), Poland (Smreczyński 1974; Wanat and Mokrzycki 2005).

*hampei* **Brisout, 1869**. Roubal 1910; Pileckis 1960, 1976a; Silfverberg 1992, 2004; Tamutis 1996; Pileckis and Monsevičius 1997.

*hirtulus* **Germar, 1824**. Silfverberg 1992, 2004; Tamutis 1996.

*ignitus* **(Germar, 1824)**. Tamutis 1996.

[*inaffectatus* **Gyllenhal, 1837**]. Known in Latvia (Telnov 2004), Estonia, Denmark, southern Sweden (Lundberg and Gustafsson 1995), throughut Poland (Smreczyński 1974; Wanat and Mokrzycki 2005).

*napi* **Gyllenhal, 1837**. Pileckis 1960, 1976a; Silfverberg 1992, 2004; Pileckis et al. 1994b; Tamutis 1996; Pileckis and Monsevičius 1997; Šurkus and Gaurilčikienė 2002.

*obstrictus* **(Marsham, 1802)** = *assimilis* (Fabricius, 1792) nec (Paykull, 1792). Heyden 1903; Pileckis 1960, 1976a; Lešinskas and Pileckis 1967; Mensonienė 1974; Pileckis et al. 1983, 1994b; Silfverberg 1992, 2004; Pileckis and Vengeliauskaitė 1996; Tamutis 1996; 2002a; Monsevičius 1997; Pileckis and Monsevičius 1997; Šurkus and Gaurilčikienė 2002; Ferenca 2006b; Alonso-Zarazaga 2009a.

*pallidactylus* **(Marsham, 1802)** = *quadridens* (Panzer, 1795) nec (Fabricius, 1775). Pileckis 1960, 1976a; Lešinskas and Pileckis 1967; Mensonienė 1974; Pileckis and Vengeliauskaitė 1977, 1996; Pileckis et al. 1983, 1994b; Silfverberg 1992, 2004; Tamutis 1996; Pileckis and Monsevičius 1997; Šablevičius 2000b; Gliaudys 2001; Ferenca 2006b; Vaivilavičius 2008; Alonso-Zarazaga 2009a.

*pallipes* **Crotch, 1866** = *minutus* (Reich, 1797) nec (Drury, 1782) = *contractus* (Marsham, 1802) nec (Geffroy, 1785). Pileckis 1960, 1976a; Silfverberg 1992, 2004; Gaidienė 1993; Tamutis 1996; Pileckis and Monsevičius 1997; Alonso-Zarazaga 2009a.

[*parvulus* **Brisout, 1869**]. Known in Denmark (Lundberg and Gustafsson 1995), Poland (Smreczyński 1974; Wanat and Mokrzycki 2005).

*pectoralis* **Weise, 1895**. Tamutis 2004.

*pervicax* **Weise, 1883**. Miländer 1982; Silfverberg 1992, 2004; Pileckis and Monsevičius 1997.

[*picitarsis* **Gyllenhal, 1837**]. Known northwestern Belarus (Alexandrovitch et al. 1996), Poland (Smreczyński 1974; Wanat and Mokrzycki 2005).

*pleurostigma* **(Marsham, 1802)** = *assimilis* (Paykull, 1792) nec (Fabricius, 1792). Pileckis 1960, 1976a; Silfverberg 1992, 2004; Pileckis et al. 1994b; Tamutis 1996; Pileckis and Monsevičius 1997; Šablevičius 2000b; Gliaudys 2001; Ferenca 2006b; Alonso-Zarazaga 2009a.

[*plumbeus* **Brisout, 1869**]. Known in Latvia (Telnov 2004), Estonia (Lundberg and Gustafsson 1995), Belarus (Alexandrovitch et al. 1996), Poland (Smreczyński 1974; Wanat and Mokrzycki 2005). 

[*posthumus* **Germar, 1824**]. Known in Latvia (Telnov 2004), Denmark, southern Sweden (Lundberg and Gustafsson 1995), Poland (Smreczyński 1974; Wanat and Mokrzycki 2005).

*pulvinatus* **Gyllenhal, 1837**. Pileckis 1960, 1976a; Silfverberg 1992, 2004; Gaidienė 1993; Tamutis 1996; Pileckis and Monsevičius 1997; Ferenca 2006b.

[*pumilio* **(Gyllenhal, 1827)**]. Known in Denmark, southern Sweden (Lundberg and Gustafsson 1995), Belarus (Alexandrovitch et al. 1996), Poland (Smreczyński 1974; Wanat and Mokrzycki 2005).

*puncticollis* **Boheman, 1845**. Silfverberg 1992, 2004; Tamutis 1996.

*pyrrhorhynchus* **(Marsham, 1802)**. Silfverberg 1992, 2004.

*querceti* **(Gyllenhal, 1813)**. Tamutis and Pankevičius 2001.

*rapae* **Gyllenhal, 1837**. Silfverberg 1992, 2004; Tamutis 1996.

*rhenanus* **Schultze, 1895**. Tamutis 1996.

[*roberti* **Gyllenhal, 1837**]. Known in southern Sweden (Lundberg and Gustafsson 1995), Belarus (Alexandrovitch et al. 1996), Poland (Smreczyński 1974; Wanat and Mokrzycki 2005).

[*scapularis* **Gyllenhal, 1837**]. Known in Latvia (Vorst et al. 2007), Estonia, Denmark, southern Sweden (Lundberg and Gustafsson 1995), northwestern Belarus (Alexandrovitch et al. 1996), throughout Poland (Smreczyński 1974; Wanat and Mokrzycki 2005).

[*similis* **Brisout, 1869**]. Known in Latvia (Telnov et al. 2006).

*sophiae* **Gyllenhal, 1837**. Pileckis 1976a; Silfverberg 1992, 2004; Tamutis 1996; Pileckis and Monsevičius 1997.

*sulcicollis* **(Paykull, 1800)**. Pileckis 1960, 1976a; Silfverberg 1992, 2004; Tamutis 1996; Pileckis and Monsevičius 1997; Ferenca 2006b; Alonso-Zarazaga 2009a.

*syrites* **Germar, 1824**. Pileckis 1960, 1976a; Silfverberg 1992, 2004; Gaidienė 1993; Tamutis 1996; Pileckis and Monsevičius 1997; Ferenca 2006b.

[*thomsoni* **Kolbe, 1900**].Known in Denmark, Sweden (Lundberg and Gustafsson 1995).

*typhae* **(Herbst, 1795)** = *floralis* (Paykull, 1892) nec (Olivier, 1790). Roubal 1910; Pileckis 1960, 1976a; Silfverberg 1992, 2004; Tamutis 1996; Pileckis and Monsevičius 1997; Alonso-Zarazaga 2009a.

*unguicularis* **Thomson, 1871** = *curvirostris* Schultze, 1898. Tamutis and Pankevičius 2001.

*Oprohinus* **Reitter, 1916**.

*suturalis* **(Fabricius, 1785)**. Pileckis 1960 (*Ceutorhynchus jakovlevi*), 1976a; Pileckis et al. 1983; Gaidienė 1993; Tamutis 1996; Pileckis and Monsevičius 1997 (*Ceutorhynchus*); Duchovskienė 2000; Šablevičius 2000b, 2011; Silfverberg 2004.

*Ethelcus* **Reitter, 1916**.

*denticulatus* **(Schrank, 1781)**. Pileckis and Monsevičius 1997 (*Ceutorhynchus*); Silfverberg 2004.

*Paratelhus* **Dieckmann, 1972**.

*pollinarius* **(Forster, 1771)**. Pileckis 1976a; Silfverberg 1992, 2004; Tamutis 1996; Pileckis and Monsevičius 1997 (*Ceutorhynchus*).

*Stenocarus* **Thomson, 1859**.

[*cardui* **(Herbst, 1784)**]. Known in Denmark, southern Sweden (Lundberg and Gustafsson 1995), throughout Poland (Smreczyński 1974; Wanat and Mokrzycki 2005).

*ruficornis* **(Stephens, 1831)** = *umbrinus* (Gyllenhal, 1827) = *fuliginosus* (Marsham, 1802) nec (Gmelin, 1790). Tamutis and Pankevičius 2001; Tamutis and Ferenca 2006; Ferenca et al. 2006, 2007.

*Glocianus* **Reitter, 1916**.

*distinctus* **(Brisout, 1870)** = *similimus* (Edwards, 1911); *marginatus* (Paykull, 1792) nec (Fabricius, 1775). Pileckis 1960, 1976a; Zajančkauskas and Pileckis 1968; Silfverberg 1992, 2004; Gaidienė 1993; Tamutis 1996; Monsevičius 1997; Pileckis and Monsevičius 1997 (*Ceutorhynchus*); Ferenca 2006b.

[*fennicus* **(Faust, 1895)**]. Known in Estonia, Denmark, throughout Sweden (Lundberg and Gustafsson 1995), northwestern Belarus (Alexandrovitch et al. 1996), Poland (Smreczyński 1974; Wanat and Mokrzycki 2005).

*moelleri* **(Thomson, 1868)**. Tamutis 1996; Ferenca 2006a.

*punctiger* **(R.F. Sahlberg, 1835)**. Pileckis 1960, 1976a; Silfverberg 1992, 2004; Tamutis 1996; Pileckis and Monsevičius 1997 (*Ceutorhynchus*); Ferenca 2006b.

*Datonychus* **Wagner, 1944**.

[*angulosus* **(Boheman, 1845)**]. Known in Denmark, southern Sweden (Lundberg and Gustafsson 1995), Belarus (Alexandrovitch et al. 1996), Poland (Smreczyński 1974; Wanat and Mokrzycki 2005).

*arquata* **(Herbst, 1795)**. Silfverberg 1992, 2004; Tamutis 1996.

*melanostictus* **(Marsham, 1802)**. Silfverberg 1992, 2004; Tamutis 1996.

[*urticae* **(Boheman, 1845)**]. Known in Estonia, Denmark (Lundberg and Gustafsson 1995).

*Microplontus* **Wagner, 1944**.

*campestris* **(Gyllenhal, 1837)**. Silfverberg 1992, 2004; Tamutis 1996.

[*edentulus* **(Schultze, 1897)**]. Known in northwestern Belarus (Alexandrovitch et al. 1996), Poland (Smreczyński 1974; Wanat and Mokrzycki 2005).

*millefolii* **(Schultze, 1897)**. Pileckis 1976a; Silfverberg 1992, 2004; Tamutis 1996; Pileckis and Monsevičius 1997 (*Ceutorhynchus*).

*rugulosus* **(Herbst, 1795)** = *figuratus* Gyllenhal, 1837 = *chrysanthemi* (Germar, 1837). Mensonienė 1974; Silfverberg 1992, 2004; Tamutis 1996; Alonso-Zarazaga 2009a.

*triangulum* **(Boheman, 1845)**. Tamutis 1996.

*Hadroplonthus* **Thomson, 1859**.

*litura* **(Fabricius, 1775)**. Gaidienė 1993; Tamutis 1996; Silfverberg 2004.

*trimaculatus **(Germar, 1824)**. # 95. Tamutis 1996.

*Mogulones* **Reitter, 1916**.

*abbreviatulus* **(Fabricius, 1792)**. Tamutis 1996; Pileckis and Monsevičius 1997 (*Ceutorhynchus*); Silfverberg 2004.

*asperifoliarum* **(Gyllenhal, 1813)**. Tamutis 1996; Pileckis and Monsevičius 1997 (*Ceutorhynchus*); Silfverberg 2004.

*crucifer* **(Pallas, 1781)** = *cruciger* (Herbst, 1784). Pileckis 1960, 1976a; Silfverberg 1992, 2004; Tamutis 1996; Pileckis and Monsevičius 1997 (*Ceutorhynchus*); Ferenca 2006b.

[*euphorbiae* **(Brisout, 1866)**]. Known in Latvia (Telnov 2004), Estonia, Denmark, southern and central Sweden (Lundberg and Gustafsson 1995), Poland (Smreczyński 1974; Wanat and Mokrzycki 2005).

*geographicus* **(Goeze, 1777)**. Miländer 1982; Silfverberg 1992, 2004; Gaidienė 1993; Tamutis 1996; Pileckis and Monsevičius 1997 (*Ceutorhynchus*); Šablevičius 2000a, 2011.

[*javetii* **(Gerhardt, 1867)**]. Known in Estonia, Denmark, southern Sweden (Lundberg and Gustafsson 1995), throughout Poland (Smreczyński 1974; Wanat and Mokrzycki 2005).

*larvatus* **(Schultze, 1897)**. Mensonienė 1974; Pileckis 1976a; Silfverberg 1992, 2004; Tamutis 1996; Pileckis and Monsevičius 1997 (*Ceutorhynchus*).

*pallidicornis* **(Gougelet & Brisout, 1860)**. Tamutis 1996; Vaivilavičius 2008.

[*raphani* **(Fabricius, 1792)** = *simphyti* (Bedel, 1885)]. Known in Belarus (Alexandrovitch et al. 1996), throughout Poland (Smreczyński 1974; Wanat and Mokrzycki 2005).

[*venedicus* **(Weise, 1879)**]. Known in northwestern Belarus (Alexandrovitch et al. 1996), Poland (Smreczyński 1974; Wanat and Mokrzycki 2005).

**Conoderinae** **Schönherr, 1833**.

**Corryssomerini Thomson, 1859**.

*Coryssomerus* **Schönherr, 1825**.

*capucinus* **(Beck, 1817)**. Pileckis and Monsevičius 1997; Ferenca et al. 2002; Silfverberg 2004.

*Euryommatus* **Roger, 1857**.

[*mariae* **Roger, 1857**]. Known in Latvia (Telnov 2004), Poland (Smreczyński 1974; Wanat and Mokrzycki 2005).

**Cossoninae** **Schönherr, 1825**.

**Pentarthrini Lacordaire, 1865**.

*Pentarthrum* **Wollaston, 1854**.

[*huttoni* **Wollaston, 1854**]. Known in Denmark (Lundberg and Gustafsson 1995; Silfverberg 2004), Poland (Wanat and Mokrzycki 2005).

**Cossonini Schönherr, 1825**.

*Cossonus* **Clairville & Schellenberg, 1798**.

*cylindricus* **R.F. Sahlberg, 1835**. Pileckis 1976a; Silfverberg 1992, 2004; Pileckis and Monsevičius 1997; Vaivilavičius 2008.

*linearis* **(Fabricius, 1775)**. Bercio and Folwaczny 1979; Silfverberg 1992, 2004; Alonso-Zarazaga 2009a.

*parallelepipedus* **(Herbst, 1795)**. Pileckis 1960, 1976a; Silfverberg 1992, 2004; Pileckis and Monsevičius 1997; Šablevičius 2001, 2003a; Ehnström et al. 2003.

**Rhyncolini Gistel, 1848**.

*Rhyncolus* **Germar, 1817** = *Eremotes* Wollaston, 1861.

*ater* **(Linnaeus, 1758)** = *chloropus* auct. nec (Linnaeus, 1758). Silfverberg 1992, 2004; Monsevičius 1997; Pileckis and Monsevičius 1997; Šablevičius 2000b, 2003a.

*elongatus* **(Gyllenhal, 1827)**. Pileckis 1976a; Silfverberg 1992, 2004; Monsevičius 1997; Pileckis and Monsevičius 1997; Šablevičius 2003a; Ferenca et al. 2006, 2007.

[*punctatulus* **Boheman, 1838**]. Known in southern Sweden (Lundberg and Gustafsson 1995), Poland (Smreczyński 1972; Wanat and Mokrzycki 2005).

[*reflexus* **Boheman, 1838**]. Known in Latvia (Telnov 2004), Estonia (Lundberg and Gustafsson 1995), Poland (Smreczyński 1972; Wanat and Mokrzycki 2005).

*sculpturatus* **Waltl, 1839** = *nitidipennis* Thomson, 1868. Tamutis and Pankevičius 2001; Ferenca et al. 2006, 2007.

*Phloeophagus* **Schönherr, 1838**.

[*lignarius* **(Marsham, 1802)**]. Known in Latvia (Telnov 2004), Estonia (Silfverberg 2004), Denmark, Sweden (Lundberg and Gustafsson 1995), Belarus (Alexandrovitch et al. 1996), Poland (Smreczyński 1972; Wanat and Mokrzycki 2005).

[*thomsoni* **(Grill, 1896)**]. Known in Estonia (Silfverberg 2004), Denmark, Sweden (Lundberg and Gustafsson 1995), Poland (Smreczyński 1972; Wanat and Mokrzycki 2005).

[*turbatus* **Schönherr, 1845**]. Known in Latvia (Telnov 2004), Estonia, Sweden (Lundberg and Gustafsson 1995), Poland (Smreczyński 1972; Wanat and Mokrzycki 2005).

**Onycholipini Wollaston, 1873**.

*Hexarthrum* **Wollaston, 1860**.

[*exiguum* **Boheman, 1838**]. Known in Belarus (Alexandrovitch et al. 1996), throughout Poland (Smreczyński 1972; Wanat and Mokrzycki 2005).

*Brachytemnus* **Wollaston, 1873**.

*porcatus* **(Germar, 1824)**. Pileckis 1959, 1960, 1976a; Gaidienė 1993; Pileckis and Monsevičius 1997; Silfverberg 2004; Ferenca 2006b; Ferenca et al. 2006, 2007.

*Stereocorynes* **Wollaston, 1873**.

*truncorum* **(Germar, 1824)**. Pileckis 1968a, 1976a; Silfverberg 1992, 2004; Pileckis and Monsevičius 1997; Ferenca et al. 2006, 2007.

*Pselactus* **Broun, 1886**.

*spadix* **(Herbst, 1795)**. Pileckis 1968b, 1976a; Silfverberg 1992, 2004; Gaidienė 1993; Pileckis and Monsevičius 1997.

*Pseudophloeophagus* **Wollaston, 1873** = *Caulotrupodes* Voss, 1955.

*aeneopiceus* **(Boheman, 1845)**. Smreczyński 1972; Pileckis and Monsevičius 1997 (*Caulotrupis***)**; Silfverberg 2004.

**Cryptorhynchinae** **Schönherr, 1825**.

**Cryptorhynchini Schönherr, 1825**.

*Cryptorhynchus* **Illiger, 1807**.

*lapathi* **(Linnaeus, 1758)**. Pileckis 1959, 1960, 1976a; Pileckis et al. 1968; Silfverberg 1992, 2004; Gaidienė 1993; Pileckis and Monsevičius 1997; Šablevičius 2000b; Vaivilavičius 2008; Alonso-Zarazaga 2009a.

*Kyklioacalles* **Stüben, 1999**.

*roboris* **(Curtis, 1834)**. Silfverberg 2004 (*Acalles*).

*Acalles* **Schönherr, 1825**.

*camelus* **(Fabricius, 1792)**. Miländer 1982; Silfverberg 1992, 2004; Pileckis and Monsevičius 1997; Ferenca et al. 2006, 2007; Alonso-Zarazaga 2009a.

*echinatus* **(Germar, 1824)**. Miländer 1982; Silfverberg 1992, 2004; Pileckis and Monsevičius 1997.

*misellus* **Boheman, 1844**. Alonso-Zarazaga 2009a.

*parvulus **Boheman, 1837** = *turbatus* Boheman, 1844. Pileckis 1976a; Silfverberg 1992, 2004; disproved by Pileckis and Monsevičius (1997).

[*ptinoides* **(Marsham, 1802)**]. Known in Denmark, southern Sweden (Lundberg and Gustafsson 1995), Poland (Smreczyński 1972; Wanat and Mokrzycki 2005).

**Cyclominae** **Schönherr, 1826**.

**Rhythirrinini Lacordaire, 1863**.

*Gronops* **Schönherr, 1823**.

[*lunatus* **(Fabricius, 1775)**]. Known in Latvia (Telnov 2004), Denmark, southern Sweden (Lundberg and Gustafsson 1995), throughout Poland (Smreczyński 1968; Wanat and Mokrzycki 2005), Kaliningrad region (Bercio and Folwaczny 1979), Belarus (Alexandrovitch et al. 1996).

*Asperogronops* **Solari, 1940**.

*inaequalis* **(Boheman, 1842)**. Pileckis 1968b, 1970a, 1976a; Mensonienė 1974; Silfverberg 1992, 2004; Pileckis and Monsevičius 1997 (*Gronops*); Ferenca et al. 2002; Šablevičius 2003a.

**Entiminae** **Schönherr, 1823**.

**Otiorhynchini Schönherr, 1826**.

*Dodecastichus* **Stierlin, 1861**

[*pulverulentus* **(Germar, 1824)**]. Known in Poland (Smreczyński 1966; Wanat and Mokrzycki 2005), Kaliningrad region (Alekseev 2002).

*Otiorhynchus* **Germar, 1822**.

*atroapterus* **(DeGeer, 1775)**. Bercio and Folwaczny 1979; Silfverberg 1992, 2004; Alonso-Zarazaga 2009a.

*coecus* **Germar, 1824** = *niger* (Fabricius, 1775). # 96. Žiogas 1997.

[*conspersus* **(Herbst, 1795)**]. Known in Latvia (Telnov 2004), southeastern Poland (Smreczyński 1966; Wanat and Mokrzycki 2005).

[*desertus* **Rosenhauer, 1847**]. Known in Latvia (Telnov 2004), Estonia, Denmark, throughout Sweden (Lundberg and Gustafsson 1995), Poland (Smreczyński 1966; Wanat and Mokrzycki 2005).

*gemmatus **(Scopoli, 1763)**. # 97. Pileckis 1960; Ferenca 2006b.

[*ligneus* **(Olivier, 1807)**]. Known in Denmark, southern Sweden (Lundberg and Gustafsson 1995), Estonia, Latvia, Poland (Alonso-Zarazaga 2009a).

*ligustici* **(Linnaeus, 1758)**. Pileckis 1960, 1976a; Lešinskas and Pileckis 1967; Silfverberg 1992, 2004; Gaidienė 1993; Pileckis and Monsevičius 1997; Ferenca 2006b; Alonso-Zarazaga 2009a.

*nodosus* **(O.F. Müller, 1764)** = *dubius* (Ström, 1783). Heyden 1903; Pileckis 1960, 1976a; Silfverberg 1992, 2004; Pileckis and Monsevičius 1997; Alonso-Zarazaga 2009a.

*orbicularis* **(Herbst, 1795)**. Šablevičius and Ferenca 1995.

*ovatus* **(Linnaeus, 1758)**. Heyden 1903; Roubal 1910; Mazurowa and Mazur 1939; Pileckis 1960, 1976a; Lešinskas and Pileckis 1967; Pileckis et al. 1968; Mensonienė 1974, 1981; Silfverberg 1992, 2004; Gaidienė 1993; Pileckis et al. 1994a; Monsevičius 1997; Pileckis and Monsevičius 1997; Žiogas 1997; Tamutis 1999; Šablevičius 2000b; Gliaudys 2001; Ferenca 2006b; Lynikienė and Gedminas 2006; Ivinskis et al. 2008; Alonso-Zarazaga 2009a.

[*porcatus* **(Herbst, 1795)**]. Known in Denmark, southern Sweden (Lundberg and Gustafsson 1995), Estonia (Silfverberg 2004), Kaliningrad region (Alekseev 2002), Poland (Smreczyński 1966; Wanat and Mokrzycki 2005). 

*raucus* **(Fabricius, 1777)**. Pileckis 1960, 1976a; Mensonienė 1974, 1981; Silfverberg 1992, 2004; Gaidienė 1993; Pileckis and Monsevičius 1997; Gliaudys 2001; Ferenca 2006b; Alonso-Zarazaga 2009a.

*rotundus* **Marseul, 1872**. Ogyjewicz 1938; Pileckis 1959, 1960, 1976a; Silfverberg 1992, 2004; Pileckis and Vengeliauskaitė 1996; Pileckis and Monsevičius 1997; Alonso-Zarazaga 2009a.

[*rugifrons* **(Gyllenhal, 1813)**]. Known in Denmark, Sweden (Lundberg and Gustafsson 1995), Estonia (Silfverberg 2004), Poland (Wanat and Mokrzycki 2005).

[*rugosostriatus* **(Goeze, 1777)**]. Known in Denmark, southern Sweden (Lundberg and Gustafsson 1995), Poland (Wanat and Mokrzycki 2005).

*scaber* **(Linnaeus, 1758)**. Pileckis 1960, 1976a; Lešinskas and Pileckis 1967; Silfverberg 1992, 2004; Gaidienė 1993; Monsevičius 1997; Pileckis and Monsevičius 1997; Žiogas and Zolubas 2005; Ferenca 2006b; Alonso-Zarazaga 2009a.

*singularis* **(Linnaeus, 1767)**. Tenenbaum 1931; Pileckis 1968b, 1976a; Silfverberg 1992, 2004; Pileckis and Monsevičius 1997.

[*smreczynskii* **Cmoluch, 1968**]. Known in Denmark (Silfverberg 2004), Poland (Wanat and Mokrzycki 2005), Belarus (Alonso-Zarazaga 2009a).

*sulcatus* **(Fabricius, 1775)**. Ferenca and Tamutis 2009.

[*tenebricosus* **(Herbst, 1784)** *= lugdunensis* Boheman, 1843 = *fuscipes* (Olivier, 1807)]. Known in Latvia (Telnov 2004), Denmark, Sweden (Lundberg and Gustafsson 1995), Estonia (Silfverberg 2004), Poland (Wanat and Mokrzycki 2005).

*tristis* **(Scopoli, 1763)**. Eichwald 1830; Ogyjewicz 1938; Pileckis 1960, 1976a; Lešinskas and Pileckis 1967; Silfverberg 1992, 2004; Gaidienė 1993; Pileckis and Monsevičius 1997; Šablevičius 2000b; Gliaudys 2001; Alonso-Zarazaga 2009a.

**Peritelini Lacordaire, 1863**.

*Simo* **Dejean, 1821** = *Homorhythmus* Bedel, 1883 = *Peritelus* auct. nec Germar, 1824.

[*hirticornis* **(Herbst, 1795)**]. Known in Denmark, southern Sweden (Lundberg and Gustafsson 1995), eastern Belarus (Alexandrovitch et al. 1996), Poland (Smreczyński 1966; Wanat and Mokrzycki 2005).

**Trachyphloeini Gistel, 1848**.

*Trachyphloeus* **Germar, 1817**.

[*alternans* **Gyllenhal, 1834**]. Known in Denmark, southern Sweden (Lundberg and Gustafsson 1995), Poland (Smreczyński 1966; Wanat and Mokrzycki 2005).

[*angustisetulus* **Hansen, 1915**]. Known in Latvia (Telnov 2004), Denmark, Sweden (Lundberg and Gustafsson 1995), Poland (Wanat and Mokrzycki 2005).

*aristatus* **(Gyllenhal, 1827)**. Pileckis 1960, 1976a; Silfverberg 1992, 2004; Gaidienė 1993; Pileckis and Monsevičius 1997; Ferenca 2006b; Alonso-Zarazaga 2009a.

*bifoveolatus* **(Beck, 1817)**. Pileckis 1960, 1963b, 1970b, 1976a; Mensonienė 1974; Silfverberg 1992, 2004; Gaidienė 1993; Pileckis and Monsevičius 1997; Tamutis 1999; Ferenca 2006b; Alonso-Zarazaga 2009a.

[*digitalis* **(Gyllenhal, 1827)**]. Known in Latvia (Telnov 2004), Estonia, Denmark, southern Sweden (Lundberg and Gustafsson 1995).

[*heymesi* **Hybenthal, 1934**]. Known in Denmark, southern Sweden (Lundberg and Gustafsson 1995), northwestern Belarus (Alexandrovitch et al. 1996), Poland (Wanat and Mokrzycki 2005).

[*rectus* **Thomson, 1865** = *laticollis* auct. nec Boheman, 1843]. Known in Denmark, southern Sweden (Lundberg and Gustafsson 1995).

*scabriculus* **(Linnaeus, 1771)**. Pileckis 1960, 1976a; Silfverberg 1992, 2004; Gaidienė 1993; Pileckis and Monsevičius 1997; Ferenca 2006b; Alonso-Zarazaga 2009a.

*spinimanus* **Germar, 1824**.Alonso-Zarazaga 2009a.

**Omiini Shuckard, 1839**.

*Omiamima* **Silfverberg, 1977** = *Omias* auct. nec Germar, 1817.

*concinna **(Boheman, 1834)**. # 98. Pileckis 1976a; Gaidienė 1993.

[*mollina* **(Boheman, 1834)**]. Known in Denmark, Sweden (Lundberg and Gustafsson 1995), northwestern Belarus (Alexandrovitch et al. 1996), Poland (Smreczyński 1966; Wanat and Mokrzycki 2005).

**Psallidiini Lacordaire, 1863**.

*Psallidium* **Herbst, 1795**.

*maxillosum **(Fabricius, 1792)**. # 99. Eichwald 1830.

**Phyllobiini Schönherr, 1826**.

*Pseudomyllocerus* **Desbrochers, 1872**.

*canescens **(Germar, 1824)**. # 100. Silfverberg 1992, 2004; Pileckis 1960, 1963b, 1976a; Pileckis and Monsevičius 1997(*Phyllobius cinerascens*).

*Phyllobius* **Germar, 1824**.

*arborator* **(Herbst, 1797)** = *psittacinus* Germar, 1824. Roubal 1910; Pileckis 1960, 1976a; Pileckis et al. 1968; Silfverberg 1992, 2004; Gaidienė 1993; Monsevičius 1997; Pileckis and Monsevičius 1997; Šablevičius 2000b; Ferenca 2006b; Vaivilavičius 2008; Alonso-Zarazaga 2009a; Ostrauskas and Ferenca 2010.

*argentatus* **(Linnaeus, 1758)**. Heyden 1903; Roubal 1910; Ogyjewicz 1938; Pileckis 1960, 1976a; Lešinskas and Pileckis 1967; Pileckis et al. 1968, 1994a; Mensonienė 1974, 1981; Silfverberg 1992, 2004; Gaidienė 1993; Monsevičius 1997; Pileckis and Monsevičius 1997; Šablevičius 2000b; Gliaudys 2001; Tamutis and Zolubas 2001; Ferenca 2006b; Vaivilavičius 2008; Alonso-Zarazaga 2009a.

*betulinus* **(Bechstein & Scharfenberg, 1805)** = *betulae* (Fabricius, 1801) nec (Linnaeus, 1758). Pileckis 1960, 1963b; 1976a; Silfverberg 1992, 2004; Pileckis and Monsevičius 1997; Alonso-Zarazaga 2009a.

*brevis* **Gyllenhal, 1834**. Eichwald 1830; Pileckis 1960, 1970b, 1976a; Silfverberg 1992, 2004; Gaidienė 1993; Pileckis and Monsevičius 1997; Ferenca 2006b; Alonso-Zarazaga 2009a.

*fessus* **Boheman, 1843**. Wanat 2005.

*glaucus* **(Scopoli, 1763)** = *calcaratus* (Fabricius, 1792). Heyden 1903; Pileckis 1960, 1976a; Zajančkauskas and Pileckis 1968; Mensonienė 1981; Silfverberg 1992, 2004; Gaidienė 1993; Monsevičius 1997; Pileckis and Monsevičius 1997; Šablevičius 2000b; Ferenca 2006b; Alonso-Zarazaga 2009a.

*maculicornis* **Germar, 1824**. Heyden 1903; Pileckis 1960, 1976a; Mensonienė 1981; Silfverberg 1992, 2004; Gaidienė 1993; Monsevičius 1997; Pileckis and Monsevičius 1997; Šablevičius 2000b; Ferenca 2006b; Vaivilavičius 2008; Alonso-Zarazaga 2009a; Ostrauskas and Ferenca 2010.

*oblongus* **(Linnaeus, 1758)**. Heyden 1903; Roubal 1910; Ogyjewicz 1938; Pileckis 1960, 1976a; Pileckis et al. 1968, 1994a; Mensonienė 1981; Silfverberg 1992, 2004; Gaidienė 1993; Pileckis and Monsevičius 1997; Šablevičius 2000b; Gliaudys 2001; Ferenca 2006b; Alonso-Zarazaga 2009a.

*pomaceus* **Gyllenhal, 1834** = *urticae* (DeGeer, 1775) nec (Scopoli, 1763). Heyden 1903; Ogyjewicz 1938; Pileckis 1960, 1976a; Mensonienė 1974, 1981; Silfverberg 1992, 2004; Gaidienė 1993; Monsevičius 1997; Pileckis and Monsevičius 1997; Šablevičius 2000b; Gliaudys 2001; Ferenca 2006b; Vaivilavičius 2008; Alonso-Zarazaga 2009a.

*pyri* **(Linnaeus, 1758)**. Pileckis 1960, 1976a; Lešinskas and Pileckis 1967; Pileckis et al. 1968, 1994a; Mensonienė 1974, 1981; Silfverberg 1992, 2004; Gaidienė 1993; Monsevičius 1997; Pileckis and Monsevičius 1997; Šablevičius 2000b; Ferenca 2006b; Vaivilavičius 2008; Alonso-Zarazaga 2009a.

*roboretanus* **Gredler, 1882** = *parvulus* (Olivier, 1807) nec (Fabricius, 1792). Pileckis 1960, 1976a; Silfverberg 1992, 2004; Pileckis and Monsevičius 1997; Ferenca 2006b; Alonso-Zarazaga 2009a.

*thalassinus* **Gyllenhal, 1834** = *scutellaris* (Redtenbacher, 1849).Mensonienė 1974, 1981.

[*vespertinus* **(Fabricius, 1792)**]. Known in Latvia (Telnov 2004), Estonia, Denmark, Sweden (Lundberg and Gustafsson 1995), Poland (Wanat and Mokrzycki 2005).

*virideaeris* **(Laicharting, 1781)**. Heyden 1903; Pileckis 1960, 1976a; Silfverberg 1992, 2004; Gaidienė 1993; Monsevičius 1997; Pileckis and Monsevičius 1997; Šablevičius 2000b; Alonso-Zarazaga 2009a.

*viridicollis* **(Fabricius, 1792)**. # 101.Pileckis et al. 1968.

**Polydrusini Schönherr, 1823**.

*Polydrusus* **Germar, 1817**.

*cervinus* **(Linnaeus, 1758)**. Pileckis 1960, 1976a; Lešinskas and Pileckis 1967; Zajančkauskas and Pileckis 1968; Pileckis et al. 1968; Silfverberg 1992, 2004; Gaidienė 1993; Monsevičius 1997; Pileckis and Monsevičius 1997; Šablevičius 2000b; Tamutis and Zolubas 2001; Žiogas and Zolubas 2005; Ferenca 2006b; Alonso-Zarazaga 2009a.

[*confluens* **Stephens, 1831**]. Known in Denmark (Lundberg and Gustafsson 1995), Belarus (Alexandrovitch et al. 1996), throughout Poland (Smreczyński 1966; Wanat and Mokrzycki 2005).

*corruscus* **Germar, 1824**. Heyden 1903; Mazurowa and Mazur 1939; Pileckis 1960, 1976a; Silfverberg 1992, 2004; Gaidienė 1993; Pileckis and Monsevičius 1997; Alonso-Zarazaga 2009a.

*flavipes* **(DeGeer, 1775)**. Pileckis 1976a; Silfverberg 1992, 2004; Pileckis and Monsevičius 1997; Alonso-Zarazaga 2009a.

*formosus* **(Mayer, 1779)** = *sericeus* (Schaller, 1783) nec (Goeze, 1777). Pileckis 1959, 1960, 1976a; Silfverberg 1992, 2004; Gaidienė 1993; Pileckis and Monsevičius 1997.

*fulvicornis* **(Fabricius, 1792)** = *ruficornis* (Bonsdorff, 1785) nec (Linnaeus, 1758). Roubal 1910; Pileckis 1960, 1976a; Lešinskas and Pileckis 1967; Silfverberg 1992, 2004; Gaidienė 1993; Pileckis and Monsevičius 1997; Šablevičius 2000b; Ferenca 2006b; Vaivilavičius 2008; Alonso-Zarazaga 2009a.

*impressifrons* **Gyllenhal, 1834**. Ferenca 2004; Alonso-Zarazaga 2009a.

*mollis* **(Ström, 1768)**. Pileckis 1960, 1976a; Zajančkauskas and Pileckis 1968; Pileckis et al. 1968; Mensonienė 1974, 1981; Silfverberg 1992, 2004; Gaidienė 1993; Monsevičius 1997; Pileckis and Monsevičius 1997; Šablevičius 2000b; Ferenca 2006b; Alonso-Zarazaga 2009a.

*pallidus* **Gyllenhal, 1834** = *atomarius* (Olivier, 1807) nec (Linnaeus, 1761). Pileckis 1976a; Silfverberg 1992, 2004; Pileckis and Monsevičius 1997.

*picus* **(Fabricius, 1792)**. Pileckis 1959, 1960; Silfverberg 1992, 2004; Ferenca 2004; as *P. piceus* F. in (Pileckis 1976a; Pileckis and Monsevičius 1997).

*pilosus* **Gredler, 1866**. Heyden 1903; Pileckis 1960, 1976a; Silfverberg 1992, 2004; Gaidienė 1993; Pileckis and Monsevičius 1997; Ferenca 2006b; Alonso-Zarazaga 2009a.

*pterygomalis* **Boheman, 1840**. Alonso-Zarazaga 2009a.

[*pulchellus* **Stephens, 1831**]. Known in Denmark (Lundberg and Gustafsson 1995), Belarus (Alexandrovitch et al. 1996).

*tereticollis* **(DeGeer, 1775)** = *undatus* (Fabricius, 1781). Heyden 1903; Pileckis 1960, 1976a; Silfverberg 1992, 2004; Gaidienė 1993; Pileckis and Monsevičius 1997; Šablevičius 2003a; Alonso-Zarazaga 2009a.

*Pachyrhinus* **Shönherr, 1823**.

[*mustela* **(Herbst, 1797)**]. Known in western Belarus (Alexandrovitch et al. 1996), throughout Poland (Smreczyński 1966; Wanat and Mokrzycki 2005).

*Liophloeus* **Germar, 1817**.

*tessulatus* **(O.F. Müller, 1776)**. Pileckis 1959, 1960, 1976a; Silfverberg 1992, 2004; Pileckis and Monsevičius 1997; Ferenca 2006b; Vaivilavičius 2008.

**Sciaphilini Sharp, 1891**.

*Eusomus* **Germar, 1824**.

*ovulum* **Germar, 1824**. Mensonienė 1974; Alonso-Zarazaga 2009a.

*Sciaphobus* **Daniel, 1904**.

[*rubi* **(Gyllenhal, 1813)**]. Known in southern Sweden (Lundberg and Gustafsson 1995), Poland (Smreczyński 1966; Wanat and Mokrzycki 2005).

*Sciaphilus* **Schönherr, 1823**.

*asperatus* **(Bonsdorff, 1785)**. Heyden 1903; Pileckis 1960, 1976a; Mensonienė 1974, 1981; Silfverberg 1992, 2004; Gaidienė 1993; Monsevičius 1997; Pileckis and Monsevičius 1997; Ferenca 2006b; Alonso-Zarazaga 2009a.

*Brachysomus* **Schönherr, 1823**.

*echinatus* **(Bonsdorff, 1785)**. Pileckis 1960, 1976a; Mensonienė 1974; Silfverberg 1992, 2004; Gaidienė 1993; Pileckis and Monsevičius 1997; Šablevičius 2000b; Tamutis and Kedienė 2005; Ferenca 2006b; Alonso-Zarazaga 2009a; Ostrauskas and Ferenca 2010.

*Barypeithes* **Jacquelin du Val, 1854**.

*araneiformis* **(Schrank, 1781)**. Alonso-Zarazaga 2009a.

[*chevrolati* **(Boheman, 1834)**]. Known in southern Sweden (Lundberg and Gustafsson 1995), Poland (Smreczyński 1966; Wanat and Mokrzycki 2005).

*mollicomus* **(Ahrens, 1812)**. Mensonienė 1974; Tamutis et al. 2008; Alonso-Zarazaga 2009a.

*pellucidus* **(Boheman, 1834)**. Šablevičius 2004; Alonso-Zarazaga 2009a.

*trichopterus* **(Gautier, 1863)**. Pileckis 1976a; Mensonienė 1981; Silfverberg 1992, 2004; Pileckis and Monsevičius 1997; Tamutis 1999; Tamutis and Kedienė 2005; Alonso-Zarazaga 2009a.

**Brachyderini Schönherr, 1826**.

*Parafoucartia* **F. Solari, 1984**.

[*squamulata* **(Herbst, 1795)**]. Known in southern Sweden (Lundberg and Gustafsson 1995), Poland (Smreczyński 1966; Wanat and Mokrzycki 2005).

*Brachyderes* **Schönherr, 1823**.

*incanus* **(Linnaeus, 1758)**. Mazurowa and Mazur 1939; Pileckis 1960, 1976a; Lešinskas and Pileckis 1967; Pileckis et al. 1968; Silfverberg 1992, 2004; Gaidienė 1993; Monsevičius 1997; Pileckis and Monsevičius 1997; Žiogas 1997; Šablevičius 2000b; Gliaudys 2001; Ferenca 2006b; Lynikienė and Gedminas 2006; Alekseev 2008a; Alonso-Zarazaga 2009a.

*Strophosoma* **Billberg, 1820** = *Neliocarus* Thomson, 1859.

*capitatum* **(DeGeer, 1775)** = *rufipes* Stephens, 1831. Heyden 1903; Roubal 1910; Mazurowa and Mazur 1939; Pileckis 1960, 1976a; Zajančkauskas and Pileckis 1968; Pileckis et al. 1968; Mensonienė 1981; Silfverberg 1992, 2004; Gaidienė 1993; Žiogas and Gedminas 1994; Monsevičius 1997; Pileckis and Monsevičius 1997; Žiogas 1997; Valenta 2000; Šablevičius 2000b; Tamutis and Zolubas 2001; Lynikienė 2003; Ferenca 2006b; Dapkus and Tamutis 2007; Vaivilavičius 2008; Alonso-Zarazaga 2009a.

*faber* **(Herbst, 1785)**. Pileckis 1960, 1970a, b, 1976a; Silfverberg 1992, 2004; Gaidienė 1993; Pileckis and Monsevičius 1997; Ferenca 2006b; Alonso-Zarazaga 2009a.

[*fulvicorne* **Walton, 1846** = *curvipes* Thomson, 1865]. Known in Latvia (Telnov 2004), Denmark, southern Sweden (Lundberg and Gustafsson 1995), Poland (Smreczyński 1966; Wanat and Mokrzycki 2005).

*melanogrammum* **(Forster, 1771)** = *obesus* Thomson, 1865. Pileckis 1959, 1960, 1976a; Pileckis et al. 1968; Mensonienė 1974; Silfverberg 1992, 2004; Pileckis and Monsevičius 1997; Alonso-Zarazaga 2009a.

[*sus* **Stephens, 1831** = *lateralis* (Paykull, 1792) nec (Panzer, 1789)]. Known in Denmark, southern Sweden (Lundberg and Gustafsson 1995), Poland (Smreczyński 1966; Wanat and Mokrzycki 2005).

**Cneorhinini Lacordaire, 1863**.

*Attactagenus* **Tournier, 1876** = *Cneorhinus* auct. nec Schönherr, 1823.

[*plumbeus* **(Marsham, 1802)** = *exaratus* (Marsham, 1802) nec (Gmelin, 1790)]. Known in Denmark, southern Sweden (Lundberg and Gustafsson 1995).

*Philopedon* **Schönherr, 1826**.

*plagiatus* **(Schaller, 1783)**. Pileckis 1960, 1976a; Pileckis et al. 1968; Silfverberg 1992, 2004; Gaidienė 1993; Pileckis and Monsevičius 1997; Gliaudys 2001; Ferenca 2006b; Alonso-Zarazaga 2009a.

**Geonemini Gistel, 1848**.

*Barynotus* **Germar, 1817**.

[*moerens* **(Fabricius, 1792)**]. Known in Latvia (Telnov 2004), Estonia, Denmark, southern Sweden (Lundberg and Gustafsson 1995), Poland (Smreczyński 1966; Wanat and Mokrzycki 2005).

*obscurus* **(Fabricius, 1775)**. Pileckis 1963b, 1976a; Silfverberg 1992, 2004; Gaidienė 1993; Pileckis and Monsevičius 1997; Vaivilavičius 2008; Alonso-Zarazaga 2009a; Ivinskis et al. 2009.

[*squamosus* **Germar, 1824**]. Known in Denmark, Sweden (Lundberg and Gustafsson 1995).

**Tropiphorini Marseul, 1863**.

*Tropiphorus* **Schönherr, 1842**.

*elevatus* **(Herbst, 1795)** = *carinatus* (O.F. Müller, 1776) nec (Linnaeus, 1767). Pileckis 1960, 1976a; Silfverberg 1992, 2004; Gaidienė 1993; Pileckis and Monsevičius 1997; Vaivilavičius 2008; Alonso-Zarazaga 2009a.

*obtusus* **(Bonsdorff, 1785)**. Miländer 1982; Silfverberg 1992, 2004; Pileckis and Monsevičius 1997; Alonso-Zarazaga 2009a.

[*terricola* **(Newman, 1838)** = *tomentosus* (Marsham, 1802) nec (Olivier, 1790)]. Known in Denmark, Sweden (Lundberg and Gustafsson 1995), Poland (Smreczyński 1966; Wanat and Mokrzycki 2005).

**Tanymecini Lacordaire, 1863**.

*Cycloderes* **C.R. Sahlberg, 1823**.

*pilosulus* **(Herbst, 1795)** = *pilosus* (Fabricius, 1792). Pileckis and Monsevičius 1997; Silfverberg 2004; Ferenca 2006b.

*Chlorophanus* **C.R. Sahlberg, 1823**.

*graminicola* **Schönherr, 1832**. Pileckis 1960, 1976a; Silfverberg 1992, 2004; Pileckis and Monsevičius 1997.

*viridis* **(Linnaeus, 1758)**. Eichwald 1830; Heyden 1903; Roubal 1910; Mazurowa and Mazur 1939; Pileckis 1960, 1976a; Lešinskas and Pileckis 1967; Zajančkauskas and Pileckis 1968; Mensonienė 1974; Silfverberg 1992, 2004; Gaidienė 1993; Monsevičius 1997; Pileckis and Monsevičius 1997; Tamutis 1999; Šablevičius 2000b; Gliaudys 2001; Ferenca 2006b.

*Tanymecus* **Germar, 1817**.

*palliatus* **(Fabricius, 1787)**. Pileckis 1960, 1976a; Lešinskas and Pileckis 1967; Mensonienė 1974, 1981; Silfverberg 1992, 2004; Gaidienė 1993; Pileckis and Monsevičius 1997; Ferenca 2006b.

**Sitonini Gistel, 1848**.

*Sitona* **Germar, 1817**.

*ambiguus* **Gyllenhal, 1834** = *lineellus* auct. nec (Bonsdorff, 1785). Pileckis 1960, 1976a; Silfverberg 1992, 2004; Gaidienė 1993; Pileckis and Monsevičius 1997; Ferenca 2006b; Alonso-Zarazaga 2009a.

[*cambricus* **Stephens, 1831**]. Known in Estonia, Denmark (Lundberg and Gustafsson 1995), Poland (Smreczyński 1966; Wanat and Mokrzycki 2005).

[*cinerascens* **Fåhraeus, 1840**]. Known in Latvia (Telnov et al. 2005), Denmark, southern Sweden (Lundberg and Gustafsson 1995), Poland (Smreczyński 1966; Wanat and Mokrzycki 2005).

*cylindricollis* **Fåhraeus, 1840**. Pileckis 1976a; Silfverberg 1992, 2004; Gaidienė 1993; Pileckis and Monsevičius 1997.

*griseus* **(Fabricius, 1775)**. Ogyjewicz 1938; Mazurowa and Mazur 1939; Pileckis 1960, 1976a; Zajančkauskas and Pileckis 1968; Silfverberg 1992, 2004; Gaidienė 1993; Monsevičius 1997; Pileckis and Monsevičius 1997; Šablevičius 2000b, 2011; Gliaudys 2001; Ferenca 2006b; Alekseev 2008a.

*gressorius* **(Fabricius, 1792)**. Šablevičius and Ferenca 1995; Šablevičius 2003a, 2011; Silfverberg 2004.

*hispidulus* **(Fabricius, 1776)**. Pileckis 1960, 1963b, 1976a; Mensonienė 1974; Silfverberg 1992, 2004; Gaidienė 1993; Pileckis and Monsevičius 1997; Ferenca 2006b; Alonso-Zarazaga 2009a.

*humeralis* **Stephens, 1831**. Pileckis 1960, 1976a; Silfverberg 1992, 2004; Gaidienė 1993; Pileckis and Monsevičius 1997; Šablevičius 2000b; Ferenca 2006b; Alonso-Zarazaga 2009a.

[*inops* **Gyllenhal, 1832**]. Known in Latvia (Telnov 2004), Estonia (Silfverberg 1992, 2004), Poland (Smreczyński 1966; Wanat and Mokrzycki 2005).

[*languidus* **Gyllenhal, 1834**]. Known in northwestern Belarus (Alexandrovitch et al. 1996), Poland (Smreczyński 1966; Wanat and Mokrzycki 2005).

*lateralis* **Gyllenhal, 1834** = *ononidis* Sharp, 1866.Alonso-Zarazaga 2009a.

*lepidus* **Gyllenhal, 1834** = *flavescens* (Marsham, 1802) nec (Fabricius, 1787). Pileckis 1960, 1976a; Lešinskas and Pileckis 1967; Silfverberg 1992, 2004; Pileckis and Monsevičius 1997; Tamutis 1999; Ferenca 2006b; Alonso-Zarazaga 2009a.

*lineatus* **(Linnaeus, 1758)**. Eichwald 1830; Heyden 1903; Roubal 1910; Ogyjewicz 1929, 1931, 1932, 1938; Pileckis 1960, 1976a; Lešinskas and Pileckis 1967; Zajančkauskas and Pileckis 1968; Mensonienė 1974; Pileckis and Vengeliauskaitė 1977, 1996; Pileckis et al. 1983, 1994b; Silfverberg 1992, 2004; Gaidienė 1993; Monsevičius 1997; Pileckis and Monsevičius 1997; Tamutis 1999; Šablevičius 2000b, 2011; Šurkus and Gaurilčikienė 2002; Ferenca 2006b; Alonso-Zarazaga 2009a.

*lineellus* **(Bonsdorff, 1785)** = *decipiens* Lindberg, 1933. Pileckis 1960; Palm 1996; Pileckis and Monsevičius 1997; Silfverberg 2004; Alonso-Zarazaga 2009a.

[*longulus* **Gyllenhal, 1834**]. Known in Latvia (Telnov 2004), Estonia (Lundberg and Gustafsson 1995), Belarus (Alexandrovitch et al. 1996), Poland (Smreczyński 1966; Wanat and Mokrzycki 2005).

*macularius* **(Marsham, 1802)** = *crinitus* (Herbst, 1795) nec (Gmelin, 1790). Ogyjewicz 1932, 1938; Pileckis 1960, 1976a; Lešinskas and Pileckis 1967; Pileckis and Vengeliauskaitė 1977, 1996; Pileckis et al. 1983, 1994b; Silfverberg 1992, 2004; Gaidienė 1993; Pileckis and Monsevičius 1997; Šablevičius 2000b; Gliaudys 2001; Ferenca 2006b; Alonso-Zarazaga 2009a.

*puncticollis* **Stephens, 1831**. Pileckis 1960, 1976a; Silfverberg 1992, 2004; Gaidienė 1993; Monsevičius 1997; Pileckis and Monsevičius 1997; Šablevičius 2000b; Ferenca 2006b; Alonso-Zarazaga 2009a.

[*regensteinensis* **(Herbst, 1797)**]. Known in Denmark, southern Sweden (Lundberg and Gustafsson 1995), Poland (Wanat and Mokrzycki 2005).

*striatellus* **Gyllenhal, 1834** = *tibialis* (Herbst, 1795) nec (Sparrman, 1787). Pileckis 1976a; Silfverberg 1992, 2004; Pileckis and Monsevičius 1997; Šablevičius 2000b; Alonso-Zarazaga 2009a.

*sulcifrons* **(Thunberg, 1798)**. Pileckis 1960, 1976a; Zajančkauskas and Pileckis 1968; Silfverberg 1992, 2004; Gaidienė 1993; Monsevičius 1997; Pileckis and Monsevičius 1997; Šablevičius 2000b; Gliaudys 2001; Alonso-Zarazaga 2009a.

*suturalis* **Stephens, 1831**. Pileckis 1960, 1976a; Mensonienė 1974; Silfverberg 1992, 2004; Gaidienė 1993; Pileckis and Monsevičius 1997; Alonso-Zarazaga 2009a.

[*waterhousei* **Walton, 1846**]. Known in Denmark (Lundberg and Gustafsson 1995), Belarus (Alexandrovitch et al. 1996), Poland (Smreczyński 1966; Wanat and Mokrzycki 2005).

**Hyperinae** **Marseul, 1863 (1848)**. # 102.

**Hyperini Marseul, 1863 (1848)**.

*Brachypera* **Capiomont, 1868**.

*dauci* **(Olivier, 1807)** = *fasciculata* (Herbst, 1795) nec (DeGeer, 1775). Pileckis 1960, 1976a; Silfverberg 1992, 2004; Pileckis and Monsevičius 1997 (*Hypera*).

[*vidua* **(Gené, 1837)**]. Known in southern Sweden (Lundberg and Gustafsson 1995).

*zoilus* **(Scopoli, 1763)** = *punctata* (Fabricius, 1775). Pileckis 1960, 1963b, 1976a; Silfverberg 1992, 2004; Gaidienė 1993; Pileckis and Monsevičius 1997 (*Hypera*); Šablevičius 2000b; Ferenca 2006b.

*Hypera* **Germar, 1817**= *Phytonomus* Schönherr, 1823.

*arator* **(Linnaeus, 1758)**. Mazurowa and Mazur 1939; Pileckis 1960, 1976a; Lešinskas and Pileckis 1967; Mensonienė 1974; Silfverberg 1992, 2004; Gaidienė 1993; Monsevičius 1997; Pileckis and Monsevičius 1997; Šablevičius 2000b; Ferenca 2006b.

*arundinis* **(Paykull, 1792)**. Pileckis 1960, 1976a; Zajančkauskas and Pileckis 1968; Silfverberg 1992, 2004; Monsevičius 1997; Pileckis and Monsevičius 1997; Šablevičius 2003a; Ferenca 2006b.

*conmaculata* **(Herbst, 1759)** = *pollux* (Fabricius, 1796) = *adspersa* (Fabricius, 1793) nec (Fabricius, 1775). Pileckis 1960, 1976a; Silfverberg 1992, 2004; Gaidienė 1993; Monsevičius 1997; Pileckis and Monsevičius 1997; Ferenca 2006b.

[*contaminata* **(Herbst, 1795)**].Known in Latvia (Telnov 2004), Poland (Smreczyński 1968; Wanat and Mokrzycki 2005).

[*denominanda* **(Capiomont, 1868)**]. Known in Latvia (Telnov et al. 2006), Denmark, Sweden (Lundberg and Gustafsson 1995), Poland (Wanat and Mokrzycki 2005).

*diversipunctata* **(Schrank, 1798)** = *elongata* (Paykull, 1792) nec (Fabricius, 1775). Pileckis 1960, 1976a; Silfverberg 1992, 2004; Gaidienė 1993; Pileckis and Monsevičius 1997.

[*fornicata* **(Penecke, 1928)**]. Known in Sweden (Lundberg and Gustafsson 1995), Poland (Smreczyński 1968; Wanat and Mokrzycki 2005).

*melancholica* **(Fabricius, 1792)** = *murinus* (Fabricius, 1793) nec (O.F. Müller, 1764). Pileckis 1960, 1976a; Mensonienė 1974; Silfverberg 1992, 2004; Gaidienė 1993; Pileckis and Monsevičius 1997; Ferenca 2006b.

*meles* **(Fabricius, 1792)**. Pileckis 1960, 1976a; Lešinskas and Pileckis 1967; Terechova 1972, 1986; Pileckis and Vengeliauskaitė 1977, 1996; Silfverberg 1992, 2004; Gaidienė 1993; Pileckis et al. 1994b; Pileckis and Monsevičius 1997; Šurkus and Gaurilčikienė 2002; Ferenca 2006b.

*miles* **(Paykull, 1792)** = *pedestris* (Paykull, 1792) nec (Poda, 1761). Ogyjewicz 1938; Pileckis 1960, 1976a; Lešinskas and Pileckis 1967; Zajančkauskas and Pileckis 1968; Silfverberg 1992, 2004; Gaidienė 1993; Monsevičius 1997; Pileckis and Monsevičius 1997; Ferenca 2006b.

*nigrirostris* **(Fabricius, 1775)**. Pileckis 1960, 1976a; Lešinskas and Pileckis 1967; Zajančkauskas and Pileckis 1968; Terechova 1972, 1978; Pileckis and Vengeliauskaitė 1977, 1996; Silfverberg 1992, 2004; Gaidienė 1993; Pileckis et al. 1994b; Monsevičius 1997; Pileckis and Monsevičius 1997; Tamutis 1999; Šablevičius 2000b; Šurkus and Gaurilčikienė 2002; Ferenca 2006b.

*plantaginis* **(DeGeer, 1775)**. Pileckis 1976a; Silfverberg 1992, 2004; Gaidienė 1993; Pileckis and Monsevičius 1997.

*postica* **(Gyllenhal, 1813)** = *variabilis* (Herbst, 1795) nec (Fabricius, 1777). Pileckis 1960, 1976a; Pileckis and Vengeliauskaitė 1977, 1996; Silfverberg 1992, 2004; Gaidienė 1993; Pileckis et al. 1994b; Pileckis and Monsevičius 1997; Ferenca 2006b.

*rumicis* **(Linnaeus, 1758)**. Pileckis 1960, 1976a; Lešinskas and Pileckis 1967; Pileckis et al. 1983, Silfverberg 1992, 2004; Gaidienė 1993; Monsevičius 1997; Pileckis and Monsevičius 1997; Šablevičius 2000b; Gliaudys 2001; Ferenca 2006b.

*venusta* **(Fabricius, 1781)** = *trilineata* (Marsham, 1802). Pileckis 1960, 1976a; Silfverberg 1992, 2004; Gaidienė 1993; Pileckis and Monsevičius 1997.

*viciae* **(Gyllenhal, 1813)**. Pileckis 1976a; Silfverberg 1992, 2004; Gaidienė 1993; Pileckis and Monsevičius 1997.

*Limobius* **Schönherr, 1843**.

*borealis* **(Paykull, 1792)**. Šablevičius 2003a, 2004.

**Lixinae** **Schönherr, 1823**.

**Lixini Schönherr, 1823**.

*Larinus* **Dejean, 1821**.

[*iaceae **(Fabricius, 1775)**]. # 103.Gaidienė 1993.

[*minutus* **Gyllenhal, 1835** = *brevis* Gyllenhal, 1835]. Known in northern Belarus (Alexandrovitch et al. 1996), Poland (Smreczyński 1968; Wanat and Mokrzycki 2005).

[*obtusus* **Gyllenhal, 1835**]. Known in northwestern Belarus (Alexandrovitch et al. 1996), Poland (Wanat and Mokrzycki 2005). 

*planus* **(Fabricius, 1792)** = *carlinae* (Olivier, 1807). Pileckis 1976a; Silfverberg 1992, 2004; Gaidienė 1993; Pileckis and Monsevičius 1997; Šablevičius 2003b.

*sturnus* (**Schaller, 1783)**. Pileckis 1960, 1970b, 1976a; Silfverberg 1992, 2004; Monsevičius 1997; Pileckis and Monsevičius 1997; Šablevičius 2000b, 2011.

*turbinatus* **Gyllenhal, 1835**. Balalaikins et al. 2011.

*Lixus* **Fabricius, 1801**.

*albomarginatus* **Boheman, 1843** = *ascanii* auct. nec (Linnaeus, 1767). Pileckis 1962, 1963b, 1976a; Silfverberg 1992, 2004; Pileckis and Monsevičius 1997.

*bardanae* **(Fabricius, 1787)**. Pileckis 1963b, 1976a; Silfverberg 1992, 2004; Pileckis and Monsevičius 1997; Vaivilavičius 2008.

[*cylindrus* **(Fabricius, 1781)**]. Known in northwestern Belarus (Alexandrovitch et al. 1996), Poland (Wanat and Mokrzycki 2005).

*iridis* **Olivier, 1807**. Pileckis 1960, 1976a; Gaidienė and Ferenca 1992; Silfverberg 1992, 2004; Gaidienė 1993; Pileckis and Monsevičius 1997; Gliaudys 2001; Vaivilavičius 2008; Šablevičius 2011.

*paraplecticus* **(Linnaeus, 1758)**. Eichwald 1830; Pileckis 1960, 1976a; Zajančkauskas and Pileckis 1968; Silfverberg 1992, 2004; Monsevičius 1997; Pileckis and Monsevičius 1997; Gliaudys 2001; Ferenca 2006b; Šablevičius 2011.

*punctiventris* **Boheman, 1835**. Pileckis and Monsevičius 1982, 1997; Šablevičius 2003a; Silfverberg 2004.

**Rhinocyllini Lacordaire, 1863**.

*Rhinocyllus* **Germar, 1819**.

*conicus* **(Frölich, 1792)**. Pileckis 1962, 1963b, 1970a, b, 1976a; Silfverberg 1992, 2004; Gaidienė 1993; Pileckis and Monsevičius 1997; Šablevičius 2000a, b, 2001, 2003a; Vaivilavičius 2008.

**Cleonini Schönherr, 1826**.

*Conorhynchus* **Motschulsky, 1860**.

*hololeucus* **(Pallas, 1781)**. Pileckis 1976a; Silfverberg 1992, 2004.

*Coniocleonus* **Motschulsky, 1860**.

*hollbergii* **(Fåhraeus, 1842)** = *glaucus* (Fabricius, 1787) nec (Scopoli, 1763). Mazurowa and Mazur 1939; Pileckis 1960, 1976a; Lešinskas and Pileckis 1967; Silfverberg 1992, 2004; Gaidienė 1993; Pileckis and Monsevičius 1997.

*nebulosus* **(Linnaeus, 1758)**. Mazurowa and Mazur 1939; Pileckis 1960, 1976a; Silfverberg 1992, 2004; Pileckis and Monsevičius 1997; Ferenca 2006b.

*Bothynoderes* **Schönherr, 1823** = *Chromoderus* Motschulsky, 1860.

*affinis* **(Schrank, 1781)** = *fasciatus* (O.F. Müller, 1776) nec (Scopoli, 1763). Pileckis 1960, 1976a; Lešinskas and Pileckis 1967; Silfverberg 1992, 2004; Gaidienė 1993; Pileckis and Monsevičius 1997; Šablevičius 2003a.

*Pseudocleonus* **Chevrolat, 1873**.

*cinereus* **(Schrank, 1781)**. Pileckis 1976a; Silfverberg 1992, 2004; Pileckis and Monsevičius 1997.

[*grammicus* **(Panzer, 1789)**]. Known in southeastern Sweden (Lundberg and Gustafsson 1995), Poland (Smreczyński 1968; Wanat and Mokrzycki 2005).

*Cyphocleonus* **Motschulsky, 1860**.

*dealbatus* **(Gmelin, 1790)** = *tigrinus* (Panzer, 1789) nec (Geoffroy, 1785). Pileckis 1960; Silfverberg 1992, 2004; Šablevičius and Ferenca 1995; Šablevičius 1995; 2003a; Pileckis and Monsevičius 1997; Gliaudys 2001; Ferenca 2004, 2006b; as *Cyphocleonus nigrinus* Pz. in (Pileckis 1976a).

*trisulcatus* **(Herbst, 1795)**. Gaidienė 1993; Silfverberg 2004.

*Cleonis* **Dejean, 1821**.

*pigra* **(Scopoli, 1763)**. Pileckis 1960, 1976a; Lešinskas and Pileckis 1967; Silfverberg 1992, 2004; Gaidienė 1993; Pileckis and Monsevičius 1997; Šablevičius 2000b, 2011; Gliaudys 2001; Ferenca 2006b.

**Mesoptiliinae** **Lacordaire, 1863**.

**Magdalidini Pascoe, 1870**.

*Magdalis* **Germar, 1817**.

*armigera* **(Geoffroy, 1785)**. Gaidienė and Ferenca 1988; Silfverberg 1992, 2004; Gaidienė 1993; Pileckis and Monsevičius 1997; Šablevičius 2000b; Alonso-Zarazaga 2009a.

*barbicornis* **(Latreille, 1804)** = *mixta* (Desbrochers, 1870). Mensonienė 1981; Tamutis 2003.

*carbonaria* **(Linnaeus, 1758)**. Pileckis 1960, 1976a; Silfverberg 1992, 2004; Gaidienė 1993; Pileckis and Monsevičius 1997; Alonso-Zarazaga 2009a; Ostrauskas and Ferenca 2010.

*caucasica* **(Tournier, 1872)**. Tamutis 2003; Vaivilavičius 2008.

*cerasi* **(Linnaeus, 1758)**. Pileckis 1976a; Silfverberg 1992, 2004; Pileckis and Monsevičius 1997; Alonso-Zarazaga 2009a.

*duplicata* **Germar, 1819** = *weisei* Schreiner, 1882. Pileckis 1963b, 1976a; Silfverberg 1992, 2004; Gaidienė 1993; Pileckis and Monsevičius 1997; Alonso-Zarazaga 2009a.

*exarata* **(Brisout, 1862)**. Tamutis 2004.

*flavicornis* **(Gyllenhal, 1836)**. Pileckis 1976a; Mensonienė 1981; Silfverberg 1992, 2004; Pileckis and Monsevičius 1997; Alonso-Zarazaga 2009a.

*frontalis* **(Gyllenhal, 1827)**. Heyden 1903; Pileckis 1960, 1976a; Zajančkauskas and Pileckis 1968; Silfverberg 1992, 2004; Gaidienė 1993; Monsevičius 1997; Pileckis and Monsevičius 1997; Alonso-Zarazaga 2009a.

*fuscicornis* **(Desbrochers, 1870)** = *quercicola* (Weise, 1872). Pileckis 1960, 1976a; Silfverberg 1992, 2004; Gaidienė 1993; Pileckis and Monsevičius 1997; Ferenca 2006b; Alonso-Zarazaga 2009a.

*linearis* **(Gyllenhal, 1827)**. Silfverberg 1992, 2004; Pileckis and Monsevičius 1997; Alonso-Zarazaga 2009a; Ostrauskas and Ferenca 2010.

*memnonia* **(Gyllenhal, 1837)**. Pileckis 1960, 1976a; Silfverberg 1992, 2004; Pileckis and Monsevičius 1997; Alonso-Zarazaga 2009a.

*nitida* **(Gyllenhal, 1827)**. Gaidienė 1993; Silfverberg 2004.

*nitidipennis* **(Boheman, 1843)**. Pileckis 1960, 1976a; Silfverberg 1992, 2004; Gaidienė 1993; Pileckis and Monsevičius 1997; Alonso-Zarazaga 2009a.

*phlegmatica* **(Herbst, 1797)**. Pileckis and Monsevičius 1997; Silfverberg 2004.

[*rufa* **Germar, 1824**]. Known in Latvia (Silfverberg 2004).

*ruficornis* **(Linnaeus, 1758)**. Ogijewicz 1929, 1931, 1932; Pileckis 1960, 1976a; Lešinskas and Pileckis 1967; Pileckis and Vengeliauskaitė 1977, 1996; Mensonienė 1981; Silfverberg 1992, 2004; Gaidienė 1993; Pileckis and Monsevičius 1997; Alonso-Zarazaga 2009a.

*violacea* **(Linnaeus, 1758)**. Pileckis 1959, 1960, 1976a; Pileckis et al. 1968; Silfverberg 1992, 2004; Gaidienė 1993; Pileckis and Monsevičius 1997; Alonso-Zarazaga 2009a.

**Molytinae** **Schönherr, 1823**.

**Anoplini Bedel, 1884**.

*Anoplus* **Germar, 1820**.

*plantaris* **(Naezén, 1794)**. Heyden 1903; Pileckis 1959, 1960, 1976a; Silfverberg 1992, 2004; Pileckis and Monsevičius 1997; Ferenca 2006b.

*roboris* **Suffrian, 1840**. Miländer 1982; Silfverberg 1992, 2004; Gaidienė 1993; Pileckis and Monsevičius 1997.

*setulosus* **Kirsch, 1870**. Gaidienė 1993; Silfverberg 2004.

**Molytini Schönherr, 1823** = Liparini Latreille, 1828.

*Liparus* **Olivier, 1807**.

*coronatus* **(Goeze, 1777)**. Silfverberg 1992, 2004; Pileckis and Monsevičius 1997; as *L. cornatus* Gz. in (Pileckis 1976a).

*glabirostris* **Küster, 1849**. Šablevičius 1995, 2003a, 2011; Šablevičius and Ferenca 1995; Silfverberg 2004.

*Leiosoma* **Stephens, 1829**.

[*deflexum* **(Panzer, 1795)**]. Known in Denmark, southern Sweden (Lundberg and Gustafsson 1995), Poland (Smreczyński 1968; Wanat and Mokrzycki 2005).

**Lepyrini Kirby, 1837**.

*Lepyrus* **Germar, 1817**.

*capucinus* **(Schaller, 1783)**. Pileckis 1960, 1976a; Silfverberg 1992, 2004; Gaidienė 1993; Pileckis and Monsevičius 1997; Ferenca 2006b.

*palustris* **(Scopoli, 1763)**. Heyden 1903; Pileckis 1960, 1976a; Lešinskas and Pileckis 1967; Mensonienė 1974; Silfverberg 1992, 2004; Gaidienė 1993; Pileckis and Monsevičius 1997; Šablevičius 2000b; Ferenca 2006b.

**Hylobini Kirby, 1837**.

*Hylobius* **Germar, 1817**.

*abietis* **(Linnaeus, 1758)**. Heyden 1903; Ivanauskas and Vailionis 1922; Ogyjewicz 1938; Mazurowa and Mazur 1939; Pileckis 1960, 1976a; Valenta 1965a, 1977, 2000a; Lešinskas and Pileckis 1967; Pileckis et al. 1968; Zajančkauskas and Pileckis 1968; Jakaitis and Valenta 1976; Gavelis and Žiogas 1991; Silfverberg 1992, 2004; Gaidienė 1993; Monsevičius 1997; Pileckis and Monsevičius 1997; Žiogas 1997; Šablevičius 2000b; Gliaudys 2001; Tamutis and Zolubas 2001; Ferenca 2006b; Vaivilavičius 2008; Ivinskis et al. 2008.

*excavatus* **(Laicharting, 1781)** = *piceus* (DeGeer, 1775). Pileckis 1960, 1970a, 1976a; Silfverberg 1992, 2004; Monsevičius 1997; Pileckis and Monsevičius 1997; Žiogas 1997.

*pinastri* **(Gyllenhal, 1813)**. Eichwald 1830; Pileckis 1960, 1976a; Lešinskas and Pileckis 1967; Pileckis et al. 1968; Zajančkauskas and Pileckis 1968; Jakaitis and Valenta 1976; Silfverberg 1992, 2004; Gaidienė 1993; Monsevičius 1997; Pileckis and Monsevičius 1997; Žiogas 1997; Gliaudys 2001; Ferenca 2006b; Ivinskis et al. 2008.

*transversovittatus* **(Goeze, 1777)**. Tamutis and Pankevičius 2001.

**Pissodini Gistel, 1848**.

*Pissodes* **Germar, 1817**.

*castaneus* **(DeGeer, 1775)** = *notatus* (Fabricius, 1787) nec (Bonsdorff, 1785). Pileckis 1960, 1976a; Valenta 1965a, 1977, 2000b; Lešinskas and Pileckis 1967; Pileckis et al. 1968; Jakaitis and Valenta 1976; Silfverberg 1992, 2004; Gaidienė 1993; Monsevičius 1997; Pileckis and Monsevičius 1997; Žiogas 1997; Gliaudys 2001; Ferenca 2006b.

[*gyllenhali* **(R.F. Sahlberg, 1834)**]. Known in Estonia, throughout Sweden (Lundberg and Gustafsson 1995), Denmark, Poland (Alonso-Zarazaga 2009a).

*harcyniae* **(Herbst, 1795)**. Pileckis 1959, 1960, 1976a; Pileckis et al. 1968; Silfverberg 1992, 2004; Pileckis and Monsevičius 1997; Žiogas 1997; Valenta 2000b.

*piceae* **(Illiger, 1807)**. Pileckis 1959, 1960, 1976a; Valenta 1965b; Silfverberg 1992, 2004; Gaidienė 1993; Pileckis and Monsevičius 1997.

*pini* **(Linnaeus, 1758)**. Ogyjewicz 1938; Mazurowa and Mazur 1939; Pileckis 1960, 1976a; Valenta 1965b, 1977, 2000b; Lešinskas and Pileckis 1967; Pileckis et al. 1968; Gavelis and Žiogas; 1991; Silfverberg 1992, 2004; Gaidienė 1993; Žiogas and Gedminas 1994; Monsevičius 1997; Pileckis and Monsevičius 1997; Žiogas 1997; Gliaudys 2001; Gedminas and Lynikienė 2005; Ferenca 2006b; Gedminas et al. 2007.

*piniphilus* **(Herbst, 1797)**. Pileckis 1959, 1960, 1976a; Valenta 1965b, 1977, 2000b; Pileckis et al. 1968; Silfverberg 1992, 2004; Gaidienė 1993; Pileckis and Monsevičius 1997; Žiogas 1997; Gedminas 2005.

*validirostris* **(R.F. Sahlberg, 1834)**. Pileckis 1959, 1960, 1976a; Pileckis et al. 1968; Silfverberg 1992, 2004; Gaidienė 1976a, b, 1993; Pileckis and Monsevičius 1997; Žiogas 1997; Milišauskas 2000; Gliaudys 2001.

**Trachodini Gistel, 1848**.

*Trachodes* **Germar, 1824**.

*hispidus* **(Linnaeus, 1758)**. Miländer 1982; Silfverberg 1992, 2004; Gaidienė 1993; Pileckis and Monsevičius 1997; Ferenca 2004; Gedminas 2005; Ferenca et al. 2006, 2007; Gedminas et al. 2007; Ivinskis et al. 2009.

**Orobitidinae** **Thomson, 1859**.

*Orobitis* **Germar, 1817**.

*cyaneus* **(Linnaeus, 1758)**. Pileckis 1960, 1976a; Silfverberg 1992, 2004; Pileckis and Monsevičius 1997; Ferenca 2006b; Vaivilavičius 2008; Alonso-Zarazaga 2009a; Šablevičius 2011.

**Scolytinae** **Latreille, 1804**. (Scolytidae)

**Hylastini LeConte, 1876**.

*Hylurgops* **LeConte, 1876**.

*glabratus* **(Zetterstedt, 1828)**. Lundberg and Gustafsson 1995, Alonso-Zarazaga 2009a.

*palliatus* **(Gyllenhal, 1813)**. Roubal 1910; Mastauskis 1927; Mastauskis and Pileckis 1959; Pileckis 1960, 1976a; Lešinskas and Pileckis 1967; Pileckis et al. 1968; Valenta and Jakaitis 1972; Jakaitis and Valenta 1976; Silfverberg 1992, 2004; Gaidienė 1993; Monsevičius 1997; Pileckis and Monsevičius 1997; Gedminas et al. 2007; Alonso-Zarazaga 2009a.

*Hylastes* **Erichson, 1836**.

*angustatus* **(Herbst, 1793)** *= scandinavicus* Lekander, 1955. Mastauskis and Pileckis 1959; Pileckis 1960, 1976a; Jakaitis and Valenta 1976; Silfverberg 1992, 2004; Gaidienė 1993; Pileckis and Monsevičius 1997; Ferenca 2006b; Alonso-Zarazaga 2009a.

*ater* **(Paykull, 1800)**. Prüffer 1948; Mastauskis and Pileckis 1959; Pileckis 1960, 1976a; Pileckis et al. 1968; Jakaitis and Valenta 1976; Silfverberg 1992, 2004; Gaidienė 1993; Pileckis and Monsevičius 1997; Žiogas 1997; Valenta 2000b; Žiogas and Zolubas 2005; Ferenca 2006b; Alonso-Zarazaga 2009a.

*attenuatus* **Erichson, 1836**. Mastauskis and Pileckis 1959; Pileckis 1960, 1976a; Silfverberg 1992, 2004; Gaidienė 1993; Pileckis and Monsevičius 1997; Ehnström et al. 2003; Alonso-Zarazaga 2009a.

[*brunneus* **Erichson, 1836** = *aterrimus* Eggers, 1933]. Known in Latvia (Telnov 2004), Estonia, Denmark, throughout Sweden (Lundberg and Gustafsson 1995), Belarus (Alexandrovitch et al. 1996), Poland (Nunberg 1954; Wanat and Mokrzycki 2005).

*cunicularius* **Erichson, 1836** = *starki* (Eggers, 1933). Mastauskis and Pileckis 1959; Pileckis 1960, 1976a; Pileckis et al. 1968; Jakaitis and Valenta 1976; Silfverberg 1992, 2004; Gaidienė 1993; Pileckis and Monsevičius 1997; Žiogas 1997; Tamutis and Zolubas 2001; Alonso-Zarazaga 2009a; Zeniauskas and Gedminas 2010.

*opacus* **Erichson, 1836**. Mastauskis and Pileckis 1959; Pileckis 1960, 1976a; Pileckis et al. 1968; Jakaitis and Valenta 1976; Silfverberg 1992, 2004; Gaidienė 1993; Monsevičius 1997; Pileckis and Monsevičius 1997; Tamutis and Zolubas 2001; Alonso-Zarazaga 2009a.

**Hylesinini Erichson, 1836**.

*Hylastinus* **Bedel, 1888**.

[*obscurus* **(Marsham, 1802)**]. Known in Latvia (Telnov 2004), Denmark, southern Sweden (Lundberg and Gustafsson 1995), Poland (Nunberg 1954; Wanat and Mokrzycki 2005).

*Pteleobius* **Bedel, 1888**.

*vittatus* **(Fabricius, 1787)**. Pileckis 1976a; Silfverberg 1992, 2004; Pileckis and Monsevičius 1997; Alonso-Zarazaga 2009a.

*Hylesinus* **Fabricius, 1801** = *Leperisinus* Reitter, 1913.

*crenatus* **(Fabricius, 1787)**. Mastauskis 1927; Mastauskis and Pileckis 1959; Pileckis 1960, 1976a; Pileckis et al. 1968; Silfverberg 1992, 2004; Gaidienė 1993; Pileckis and Monsevičius 1997; Žiogas 1997; Alonso-Zarazaga 2009a.

*fraxini* **(Panzer, 1779)** = *varius* Fabricius 1775. Mastauskis 1927, 1936; Ogijewicz 1931; Mastauskis and Pileckis 1959; Pileckis 1960, 1976a; Lešinskas and Pileckis 1967; Pileckis et al. 1968; Gavelis and Žiogas 1991; Silfverberg 1992, 2004; Gaidienė 1993; Pileckis and Monsevičius 1997; Žiogas 1997; Valenta 2000b; Šablevičius 2000b, 2011; Gliaudys 2001; Tamutis and Zolubas 2001; Ferenca 2006b; Alonso-Zarazaga 2009a.

[*toranio* **(Danthoine, 1788)** = *oleiperda* (Fabricius, 1792)]. Known in Latvia (Telnov 2004), Denmark, southern Sweden (Lundberg and Gustafsson 1995), Poland (Nunberg 1954; Wanat and Mokrzycki 2005).

*wachtli orni* **Fuchs, 1906**. Tamutis 2003.

**Hylurgini Gistel, 1848**.

*Xylechinus* **Chapuis, 1869**.

[*pilosus* **(Ratzeburg, 1837)**]. Known in Latvia (Telnov 2004), Estonia, Denmark, throughout Sweden (Lundberg and Gustafsson 1995), Belarus (Alexandrovitch et al. 1996), Poland (Nunberg 1954; Wanat and Mokrzycki 2005).

*Hylurgus* **Latreille, 1807**.

*ligniperda* **(Fabricius, 1787)**. Mazurowa and Mazur 1939; Pileckis 1960, 1963b, 1976a; Jakaitis and Valenta 1976; Silfverberg 1992, 2004; Gaidienė 1993; Pileckis and Monsevičius 1997; Ferenca 2006b; Alonso-Zarazaga 2009a.

*Tomicus* **Latreille, 1803** = *Blastophagus* Eichhoff, 1864.

*minor* **(Hartig, 1834)**. Mastauskis 1927; Prüffer 1948; Mastauskis and Pileckis 1959; Pileckis 1960, 1976a; Valenta 1965b, 1977, 2000b; Lešinskas and Pileckis 1967; Pileckis et al. 1968; Gavelis and Žiogas 1991; Silfverberg 1992, 2004; Gaidienė 1976, 1993; Žiogas and Gedminas 1994; Pileckis and Monsevičius 1997; Žiogas 1997; Gliaudys 2001; Gedminas and Lynikienė 2005; Gedminas et al. 2007; Alonso-Zarazaga 2009a.

*piniperda* **(Linnaeus, 1758)**. Mastauskis 1927; Prüffer 1948; Mastauskis and Pileckis 1959; Pileckis 1960, 1976a; Valenta 1965b, 1977, 2000b; Lešinskas and Pileckis 1967; Zajančkauskas and Pileckis 1968; Pileckis et al. 1968; Jakaitis and Valenta 1976; Gavelis and Žiogas 1991; Silfverberg 1992, 2004; Gaidienė 1993; Žiogas and Gedminas 1994; Monsevičius 1997; Pileckis and Monsevičius 1997; Žiogas 1997; Gliaudys 2001; Gedminas and Lynikienė 2005; Ferenca 2006b; Alonso-Zarazaga 2009a.

*Dendroctonus* **Erichson, 1836**.

*micans* **(Kugeann, 1794)**. Mastauskis 1925, 1927; Mastauskis and Pileckis 1959; Pileckis 1960, 1976a; Pileckis et al. 1968; Silfverberg 1992, 2004; Gaidienė 1993; Pileckis and Monsevičius 1997; Žiogas 1997; Šablevičius 2000b, 2004, 2011; Alonso-Zarazaga 2009a.

**Phloeotribini Chapuis, 1869**.

*Phloeotribus* **Latreille, 1796** = *Phthorophloeus* Rey, 1883.

[*rhododactylus* **(Marsham, 1802)**]. Known in Latvia (Telnov 2004), Denmark, southern Sweden (Lundberg and Gustafsson 1995), Kaliningrad region (Alekseev 2005b), Poland (Nunberg 1954; Wanat and Mokrzycki 2005).

*spinulosus* **(Rey, 1883)**. Pileckis and Monsevičius 1997; Silfverberg 2004.

**Polygraphini Chapuis, 1869**.

*Polygraphus* **Erichson, 1836**.

[*grandiclava* **Thomson, 1886**].Known in Denmark, Sweden (Alonso-Zarazaga 2009a), Poland (Nunberg 1954; Wanat and Mokrzycki 2005).

*poligraphus* **(Linnaeus, 1758)**. Vozhev 1892; Mastauskis 1927; Mastauskis and Pileckis 1959; Pileckis 1960, 1963b, 1976a; Valenta 1965b, 2000b; Lešinskas and Pileckis 1967; Pileckis et al. 1968; Valenta and Jakaitis 1972; Silfverberg 1992, 2004; Gaidienė 1993; Pileckis and Monsevičius 1997; Žiogas 1997; Šablevičius 2000b; Gliaudys 2001; Alonso-Zarazaga 2009a; Ostrauskas and Ferenca 2010; Zeniauskas and Gedminas 2010.

[*punctifrons* **Thomson, 1886**].Known in Estonia, Sweden (Lundberg and Gustafsson 1995), Belarus (Alexandrovitch et al. 1996), Poland (Nunberg 1954; Wanat and Mokrzycki 2005).

*subopacus* **Thomson, 1871**. Gedminas et al. 2007.

*Carphoborus* **Eichhoff, 1864**.

[*cholodkovskyi* **Spessivtsteff, 1916**]. Known in Estonia, Sweden (Lundberg and Gustafsson 1995), Belarus (Alexandrovitch et al. 1996), Poland (Nunberg 1954; Wanat and Mokrzycki 2005).

[*minimus* **(Fabricius, 1798)**]. Known in Belarus (Alexandrovitch et al. 1996), Poland (Nunberg 1954; Wanat and Mokrzycki 2005).

**Scolytini Latreille, 1804**.

*Scolytus* **Geoffroy, 1762**.

[*carpini* **(Ratzeburg, 1837)**]. Known in Latvia (Telnov 2004), Estonia, Sweden (Lundberg and Gustafsson 1995), Belarus (Alexandrovitch et al. 1996), Kaliningrad region (Alekseev 2005b), Poland (Nunberg 1954; Wanat and Mokrzycki 2005).

*intricatus* **(Ratzeburg, 1837)**. Mastauskis 1927; Mastauskis and Pileckis 1959; Pileckis 1960, 1976a; Pileckis et al. 1968; Silfverberg 1992, 2004; Pileckis and Monsevičius 1997; Žiogas 1997; Valenta 2000b; Gedminas 2005; Gedminas et al. 2007; Alonso-Zarazaga 2009a.

*laevis* **Chapuis, 1869**. # 104.Pileckis et al. 1968; Žiogas 1997; Valenta 2000b.

*mali* **(Bechstein and Scharfenberg, 1805)**. Mastauskis 1927; Mastauskis and Pileckis 1959; Pileckis 1960, 1976a; Lešinskas and Pileckis 1967; Silfverberg 1992, 2004; Pileckis et al. 1994a; Pileckis and Vengeliauskaitė 1996; Pileckis and Monsevičius 1997; Alonso-Zarazaga 2009a.

*multistriatus* **(Marsham, 1802)**. Mastauskis and Pileckis 1959; Pileckis 1959, 1960, 1976a; Pileckis et al. 1968; Silfverberg 1992, 2004; Pileckis and Monsevičius 1997; Žiogas 1997; Valenta 2000b; Alonso-Zarazaga 2009a.

*pygmaeus* **(Fabricius, 1787)**. Mastauskis and Pileckis 1959; Pileckis 1960, 1976a; Pileckis et al. 1968; Silfverberg 1992, 2004; Pileckis and Monsevičius 1997; Žiogas 1997; Valenta 2000b; Alonso-Zarazaga 2009a.

*ratzeburgi* **Janson, 1856**. Mastauskis 1927; Mastauskis and Pileckis 1959; Pileckis 1960, 1976a; Lešinskas and Pileckis 1967; Pileckis et al. 1968; Gavelis and Žiogas 1991; Silfverberg 1992, 2004; Pileckis and Monsevičius 1997; Žiogas 1997; Valenta 2000b; Šablevičius 2000b, 2011; Gliaudys 2001; Alonso-Zarazaga 2009a.

*rugulosus* **(O.F. Müller, 1818)**. Mastauskis 1927; Mastauskis and Pileckis 1959; Pileckis 1960, 1976a; Pileckis et al. 1994a; Silfverberg 1992, 2004; Pileckis and Monsevičius 1997; Alonso-Zarazaga 2009a; Ostrauskas and Ferenca 2010.

*scolytus* **(Fabricius, 1775)**. Mastauskis 1927; Mastauskis and Pileckis 1959; Pileckis 1960, 1976a; Lešinskas and Pileckis 1967; Pileckis et al. 1968; Silfverberg 1992, 2004; Gaidienė 1993; Pileckis and Monsevičius 1997; Žiogas 1997; Valenta 2000b; Ferenca 2006b; Alonso-Zarazaga 2009a.

[*triarmatus* **(Eggers, 1912)**]. Known in Estonia (Süda 2006), Denmark, southern and middle Sweden (Lundberg and Gustafsson 1995).

**Ipini Bedel, 1888**.

*Pityogenes* **Bedel, 1888**.

*bidentatus* **(Herbst, 1784)**. Prüffer 1948; Mastauskis and Pileckis 1959; Pileckis 1960, 1976a; Lešinskas and Pileckis 1967; Pileckis et al. 1968; Silfverberg 1992, 2004; Pileckis and Monsevičius 1997; Žiogas 1997; Valenta 2000b; Gliaudys 2001; Tamutis and Zolubas 2001; Gedminas 2005; Alonso-Zarazaga 2009a.

*chalcographus* **(Linnaeus, 1761)**. Vozhev 1892; Mastauskis 1927; Prüffer 1948; Mastauskis and Pileckis 1959; Pileckis 1960, 1976a; Valenta 1965b, 2000b; Lešinskas and Pileckis 1967; Pileckis et al. 1968; Zajančkauskas and Pileckis 1968; Valenta and Jakaitis 1972; Gavelis and Žiogas 1991; Silfverberg 1992, 2004; Gaidienė 1993; Monsevičius 1997; Pileckis and Monsevičius 1997; Žiogas 1997; Šablevičius 2000b, 2011; Gliaudys 2001; Tamutis and Zolubas 2001; Gedminas 2005; Gedminas and Lynikienė 2005; Gedminas et al. 2007; Alonso-Zarazaga 2009a; Zeniauskas and Gedminas 2010.

*irkutensis monacensis* **Fuchs, 1911**.Mastauskis and Pileckis 1959; Pileckis 1960, 1963b, 1976a; Silfverberg 1992, 2004; Pileckis and Monsevičius 1997; Alonso-Zarazaga 2009a.

*quadridens* **(Hartig, 1834)**. Prüffer 1948; Mastauskis and Pileckis 1959; Pileckis 1960, 1976a; Valenta 1965b, 1977, 2000b; Lešinskas and Pileckis 1967; Pileckis et al. 1968; Silfverberg 1992, 2004; Gaidienė 1993; Žiogas and Gedminas 1994; Pileckis and Monsevičius 1997; Žiogas 1997; Gedminas 2005; Ferenca 2006b; Alonso-Zarazaga 2009a.

[*trepanatus* **(Nördlinger, 1848)**].Known in Latvia (Telnov 2004), Estonia, Denmark, southern and middle Sweden (Lundberg and Gustafsson 1995), Belarus (Alexandrovitch et al. 1996), Poland (Nunberg 1954; Wanat and Mokrzycki 2005).

*Pityokteines* **Fuchs, 1911**.

*curvidens* **(Germar, 1824)**. Mastauskis and Pileckis 1959; Pileckis 1960, 1963b, 1976a; Silfverberg 1992, 2004; Pileckis and Monsevičius 1997; Alonso-Zarazaga 2009a.

*Orthotomicus* **Ferrari, 1867**.

*erosus* **(Wollaston, 1857)**. Mastauskis 1925, 1927.

*laricis* **(Fabricius, 1792)**. Eichwald 1830; Roubal 1910; Mastauskis 1925, 1927; Mastauskis and Pileckis 1959; Pileckis 1960, 1976a; Jakaitis and Valenta 1976; Silfverberg 1992, 2004; Gaidienė 1993; Monsevičius 1997; Pileckis and Monsevičius 1997; Ferenca 2006b; Alonso-Zarazaga 2009a.

*longicollis* **(Gyllenhal, 1827)**. Gaidienė 1993; Silfverberg 2004.

*proximus* **(Eichhoff, 1868)**. Mazurowa and Mazur 1939; Prüffer 1948; Mastauskis and Pileckis 1959; Pileckis 1960, 1976a; Lešinskas and Pileckis 1967; Pileckis et al. 1968; Silfverberg 1992, 2004; Gaidienė 1993; Pileckis and Monsevičius 1997; Žiogas 1997;Valenta 2000b; Šablevičius 2000b; Tamutis and Zolubas 2001; Ferenca 2006b; Alonso-Zarazaga 2009a.

*starki* **Spessivtseff, 1926**. Jakaitis 1985; Silfverberg 1992, 2004; Pileckis and Monsevičius 1997; Alonso-Zarazaga 2009a.

*suturalis* **(Gyllenhal, 1827)**. Mastauskis 1927; Prüffer 1948; Mastauskis and Pileckis 1959; Pileckis 1960, 1963b, 1976a; Lešinskas and Pileckis 1967; Pileckis et al. 1968; Jakaitis and Valenta 1976; Silfverberg 1992, 2004; Gaidienė 1993; Pileckis and Monsevičius 1997; Žiogas 1997; Tamutis and Zolubas 2001; Alonso-Zarazaga 2009a.

*Ips* **DeGeer, 1775**.

*acuminatus* **(Gyllenhal, 1827)**. Prüffer 1948; Mastauskis and Pileckis 1959; Pileckis 1960, 1976a; Pileckis et al. 1968; Silfverberg 1992, 2004; Pileckis and Monsevičius 1997; Žiogas 1997; Valenta 2000b; Gedminas 2005; Alonso-Zarazaga 2009a.

*amitinus* **(Eichhoff, 1871)**. Vozhev 1892; Mastauskis and Pileckis 1959; Pileckis 1960, 1963b, 1976a; Valenta 1965b, 1977; Silfverberg 1992, 2004; Pileckis and Monsevičius 1997; Alonso-Zarazaga 2009a.

[*cambrae* **(Heer, 1836)** = *subelongatus* Motschulsky, 1860]. Known in Denmark, Sweden (Lundberg and Gustafsson 1995), Poland (Nunberg 1954; Wanat and Mokrzycki 2005).

*duplicatus* **(R.F. Sahlberg, 1836)**. Mastauskis 1925, 1927; Mastauskis and Pileckis 1959; Pileckis 1960, 1976a; Valenta 1965b, 2000b; Pileckis et al. 1968; Valenta and Jakaitis 1972; Silfverberg 1992, 2004; Gaidienė 1993; Pileckis and Monsevičius 1997; Žiogas 1997; Tamutis and Zolubas 2001; Alonso-Zarazaga 2009a.

*sexdentatus* **(Börner, 1776)**. Mastauskis 1925, 1927; Prüffer 1948; Mastauskis and Pileckis 1959; Pileckis 1960, 1976a; Valenta 1965b, 1977, 2000b; Lešinskas and Pileckis 1967; Pileckis et al. 1968; Silfverberg 1992, 2004; Gaidienė 1993; Monsevičius 1997; Pileckis and Monsevičius 1997; Žiogas 1997; Gliaudys 2001; Ferenca 2006b; Alonso-Zarazaga 2009a.

*typographus* **(Linnaeus, 1758)**. Eichwald 1830; Vozhev 1892; Ivanauskas and Vailionis 1922; Mastauskis 1927; Mastauskis and Pileckis 1959; Pileckis 1960, 1976a; Valenta 1965b, 2000b; Lešinskas and Pileckis 1967; Pileckis et al. 1968; Zajančkauskas and Pileckis 1968; Valenta and Jakaitis 1972; Gavelis and Valenta 1976; Gavelis and Žiogas 1991; Silfverberg 1992, 2004; Gaidienė 1993; Monsevičius 1997; Pileckis and Monsevičius 1997; Žiogas 1997; Šablevičius 2000b, 2011; Gliaudys 2001; Tamutis and Zolubas 2001; Ferenca 2006b; Gedminas et al. 2007; Vaivilavičius 2008; Alonso-Zarazaga 2009a; Ostrauskas and Ferenca 2010 Zeniauskas and Gedminas 2010.

**Dryocoetini Lindemann, 1877**.

*Lymantor* **Lövendal, 1889**.

*aceris* **(Lindemann, 1875)**. Stark 1952; Mastauskis and Pileckis 1959; Pileckis 1960, 1976a; Silfverberg 1992, 2004; Pileckis and Monsevičius 1997; Alonso-Zarazaga 2009a.

*coryli* **(Perris, 1853)**. Pileckis et al. 1968; Pileckis 1988; Silfverberg 1992, 2004; Pileckis and Monsevičius 1997; Alonso-Zarazaga 2009a.

*Dryocoetes* **Eichhoff, 1864**.

*alni* **(Georg, 1856)**. Pileckis and Monsevičius 1997; Silfverberg 2004.

*autographus* **(Ratzeburg, 1837)** = *suecicus* Eggers, 1923. Mastauskis and Pileckis 1959; Pileckis 1960, 1963b, 1976a; Valenta and Jakaitis 1972; Jakaitis and Valenta 1976; Silfverberg 1992, 2004; Gaidienė 1993; Monsevičius 1997; Pileckis and Monsevičius 1997; Žiogas and Zolubas 2005; Ferenca 2006b; Alonso-Zarazaga 2009a; Ostrauskas and Ferenca 2010; Zeniauskas and Gedminas 2010.

*hectographus* **Reitter, 1913**. Pileckis 1963b, 1976a; Pileckis et al. 1968; Valenta and Jakaitis 1972; Silfverberg 1992, 2004; Pileckis and Monsevičius 1997; Valenta 2000b; Alonso-Zarazaga 2009a.

*villosus* **(Fabricius, 1792)**. Pileckis and Monsevičius 1997; Silfverberg 2004; Alonso-Zarazaga 2009a.

*Taphrorychus* **Eichhoff, 1878**.

*bicolor* **(Herbst, 1793)**. Silfverberg 1992, 2004; Pileckis and Monsevičius 1997; Alonso-Zarazaga 2009a.

**Crypturgini LeConte, 1876**.

*Crypturgus* **Erichson, 1836**.

*cinereus* **(Herbst, 1793)**. Prüffer 1948; Mastauskis and Pileckis 1959; Pileckis 1960, 1976a; Pileckis et al. 1968; Silfverberg 1992, 2004; Gaidienė 1993; Žiogas and Gedminas 1994; Pileckis and Monsevičius 1997; Tamutis and Zolubas 2001; Gedminas 2005; Ferenca 2006b; Alonso-Zarazaga 2009a; Vaivilavičius 2008; Zeniauskas and Gedminas 2010; Šablevičius 2011.

*hispidulus* **Thomson, 1870**. Monsevičius 1988b, 1997; Silfverberg 1992, 2004; Gaidienė 1993; Pileckis and Monsevičius 1997; Alonso-Zarazaga 2009a.

*pusillus* **(Gyllenhal, 1813)** = *maulei* (Roubal, 1910). Roubal 1910; Mastauskis and Pileckis 1959; Pileckis 1960, 1963b, 1976a; Pileckis et al. 1968; Silfverberg 1992, 2004; Monsevičius 1997; Pileckis and Monsevičius 1997; Alonso-Zarazaga 2009a.

*subcribrosus* **Eggers, 1933**. Roubal 1910; Stark 1952; Mastauskis and Pileckis 1959; Pileckis 1960, 1976a; Silfverberg 1992, 2004; Pileckis and Monsevičius 1997; Alonso-Zarazaga 2009a.

**Xyloterini LeConte, 1876**.

*Trypodendron* **Stephens, 1830** = *Xyloterus* Erichson, 1836.

*domesticum* **(Linnaeus, 1758)**. Mastauskis 1925, 1927; Pileckis et al. 1968; Monsevičius 1988b; Gaidienė and Ferenca 1988; Silfverberg 1992, 2004; Gaidienė 1993; Pileckis and Monsevičius 1997; Alonso-Zarazaga 2009a.

[*laeve* **Eggers, 1939** = *piceum* Strand, 1946 = *proximum* auct. nec (Niijima, 1909)]. Known in Latvia (Telnov 2004), Estonia (Silfverberg 2004), Sweden (Lundberg and Gustafsson 1995).

*lineatum* **(Olivier, 1795)**. Mastauskis 1927; Prüffer 1948; Mastauskis and Pileckis 1959; Pileckis 1960, 1976a; Valenta 1965b, 1977, 2000b; Lešinskas and Pileckis 1967; Pileckis et al. 1968; Gavelis and Žiogas 1991; Silfverberg 1992, 2004; Gaidienė 1993; Monsevičius 1997; Pileckis and Monsevičius 1997; Žiogas 1997; Ferenca 2006b; Vaivilavičius 2008; Alonso-Zarazaga 2009a; Ostrauskas and Ferenca 2010; Šablevičius 2011.

*signatum* **(Fabricius, 1787)**. Mastauskis and Pileckis 1959; Pileckis 1960, 1976a; Pileckis et al. 1968; Silfverberg 1992, 2004; Gaidienė 1993; Monsevičius 1997; Pileckis and Monsevičius 1997; Ferenca 2006b; Alonso-Zarazaga 2009a.

**Xyleborini LeConte, 1876**.

*Xyleborus* **Eichhoff, 1864** = *Anisandrus* Ferrari, 1867.

*cryptographus* **(Ratzeburg, 1837)**. Tamutis and Zolubas 2001; Silfverberg 2004.

*dispar* **(Fabricius, 1792)**. Mastauskis and Pileckis 1959; Pileckis 1960, 1963b, 1976a; Pileckis et al. 1968; Silfverberg 1992, 2004; Gaidienė 1993; Pileckis and Monsevičius 1997; Ferenca 2006b; Alonso-Zarazaga 2009a; Šablevičius 2011.

[*dryographus **(Ratzeburg, 1837)**]. # 105. Ferenca 2006b.

*monographus* **(Fabricius, 1792)**. Pileckis and Monsevičius 1997; Silfverberg 2004.

*Xyleborinus* **Reitter, 1913**.

*saxesenii* **(Ratzeburg, 1837)**. Pileckis and Monsevičius 1997; Tamutis and Zolubas 2001; Silfverberg 2004; Alonso-Zarazaga 2009a.

**Cryphalini Lindemann, 1877**.

*Trypophloeus* **Fairmaire, 1868**.

[*alni* **(Lindemann, 1875)**]. Known in Estonia (Lundberg and Gustafsson 1995), Belarus (Alexandrovitch et al. 1996), Poland (Nunberg 1954; Wanat and Mokrzycki 2005).

*asperatus* **(Gyllenhal, 1813)**. Lindelöw 2010. 

[*bispinulus* **Eggers, 1927**]. Known in Latvia (Telnov 2004), Estonia, Sweden (Lundberg and Gustafsson 1995), Belarus (Alexandrovitch et al. 1996).

*discedens* **Palm, 1950** = *palmi* Hansen, 1955. Lindelöw 2010.

*granulatus granulatus* **(Ratzeburg, 1837)**. Stark 1952; Mastauskis and Pileckis 1959; Pileckis 1960, 1976a; Silfverberg 1992, 2004; Pileckis and Monsevičius 1997; Alonso-Zarazaga 2009a.

*Ernoporicus* **Berger, 1917** = *Ernopocerus* Wood, 1954.

[*caucasicus* **(Lindemann, 1876)**].Known in Denmark, southern Sweden (Lundberg and Gustafsson 1995), Poland (Nunberg 1954; Wanat and Mokrzycki 2005).

[*fagi* **(Fabricius, 1798)**]. Known in Denmark, southern Sweden (Lundberg and Gustafsson 1995), Kaliningrad region (Alekseev 2005b), Poland (Nunberg 1954; Wanat and Mokrzycki 2005).

*Ernoporus* **Thomson, 1859**.

*tiliae* **(Panzer, 1793)**. Pileckis et al. 1968; Tamutis and Zolubas 2001; Silfverberg 2004.

*Cryphalus* **Erichson, 1836**.

*abietis* **(Ratzeburg, 1837)**.Valenta and Jakaitis 1972; Pileckis 1976a; Silfverberg 1992, 2004; Pileckis and Monsevičius 1997; Alonso-Zarazaga 2009a.

*piceae* **(Ratzeburg, 1837)**. Mastauskis and Pileckis 1959; Pileckis 1960, 1976a; Valenta and Jakaitis 1972; Silfverberg 1992, 2004; Pileckis and Monsevičius 1997; Alonso-Zarazaga 2009a.

[*saltuarius* **Weise, 1891**]. Known in Estonia, throughout Sweden (Lundberg and Gustafsson 1995), Belarus (Alexandrovitch et al. 1996), Poland (Nunberg 1954; Wanat and Mokrzycki 2005).

**Cortylini LeConte, 1876**.

*Pityophthorus* **Eichhoff, 1864**.

*glabratus* **Eichhoff, 1878**. Mastauskis and Pileckis 1959; Pileckis 1960, 1976; Silfverberg 1992, 2004; Pileckis and Monsevičius 1997; Alonso-Zarazaga 2009a.

*lichtensteinii* **(Ratzeburg, 1837)**. Prüffer 1948; Mastauskis and Pileckis 1959; Pileckis 1960, 1976a; Silfverberg 1992, 2004; Pileckis and Monsevičius 1997; Ferenca 2004; Gedminas et al. 2007; Alonso-Zarazaga 2009a.

*micrographus* **(Linnaeus, 1758)**. Prüffer 1948; Mastauskis and Pileckis 1959; Pileckis 1960, 1976a; Silfverberg 1992, 2004; Pileckis and Monsevičius 1997; Alonso-Zarazaga 2009a; Šablevičius 2011.

*morosovi* **Spessivtseff, 1926**. Lindelöw 2010.

*pityographus* **(Ratzeburg, 1837)**. Tamutis 2003, 2004.

[*pubescens* **(Marsham, 1802)**]. Known in Denmark, southern Sweden (Lundberg and Gustafsson 1995), Poland (Wanat and Mokrzycki 2005).

[*traeghardhi* **Spessivtseff, 1921**]. Known in Estonia, Sweden (Lundberg and Gustafsson 1995), Belarus (Alexandrovitch et al. 1996), throughout Poland (Nunberg 1954; Wanat and Mokrzycki 2005).

**Platypodinae** **Shuckard, 1839**.

**Platypodini Shuckard, 1839**.

*Platypus* **Herbst, 1793**.

[*cylindrus* **(Fabricius, 1792)**]. Known in Belarus (Alexandrovitch et al. 1996), Poland (Nunberg 1954; Wanat and Mokrzycki 2005).

## Comments

1. Only one record of *Rhysodes sulcatus* (Fabricius, 1787) older than 150 years is known in Lithuania from Tenenbaum’s collections (Ogijewicz 1933; Pileckis 1960), but without explanation this record was not confirmed in later monographs (Pileckis 1976a; Pileckis and Monsevičius 1995).

2. To this day only one record of *Nebria rufescens* (Ström, 1768) older than 50 years is documented in Lithuania (Pileckis 1968b; Pileckis and Monsevičius 1995).

3. To this day only two records of *Calosoma sycophanta* (Linnaeus, 1758) are known in Lithuania (Lentz 1879; Pileckis and Monsevičius 1982). The recent discovery in the territory of Kurtuvėnai Regional Park (Gliaudys 2001) is doubtful and needs further confirmation.

4. *Callisthenes reticulatum* (Fabricius, 1787) is an extinct species in the Kaliningrad region (Alekseev 2008c); not found in Belarus since 1962 (Alexandrovich 1991). The chance to find this species in Lithuania is minimal.

5. *Carabus scheidleri* Panzer, 1799 is noted for Lithuania by Pileckis (1960), but was probably misidentified for *Carabus arcensis* Herbst, 1784, and was not confirmed. *Carabus scheidleri* should be removed from the list of Lithuanian beetles.

6. Only one record of *Pogonus chalceus* (Marsham, 1802) older than 70 years is known in Lithuania from Palionis’ cards (Pileckis 1960; Pileckis and Monsevičius 1995; Ferenca 2006b).

7. *Asaphidion curtum* (Heyden, 1870) was misidentified for *Asaphidion flavipes* (Linnaeus, 1761) and erroneously noted for Lithuania (Tamutis and Ferenca 2006). This species should be removed from the list of Lithuanian beetles. Furthermore *Asaphidion curtum* is known in southern Sweden (Lundberg and Gustafsson 1995), Latvia (Barševskis 2003), Denmark (Vigna Taglianti 2009), so it could be expected in Lithuania.

8. *Odontium foraminosum* (Sturm, 1825), *Ocydromus fluviatilis* (Dejean, 1831) *Ocydromus tibialis* (Duftschmid, 1812), *Ocydromus decorus* (Panzer, 1799), and *Ocydromus modestus* (Fabricius, 1801) have been noted for Lithuania by Marggi et al. (2003b) and Vigna Taglianti (2009). *Odontium foraminosum* is distributed in south European and the southern part of central Europe, so probably these notifications were made in error. *Ocydromus* *fluviatilis* is distributed in the southern and central Europe, northeastern Poland (Burakowski et al. 1973), and southern Belarus (Alexandrovitch et al. 1996), so its notification could be reliable. The other three species, namely, *Ocydromus tibialis*, *Ocydromus decorus*, and *Ocydromus modestus* are usually considered as mountain species and are distributed in the mountain regions of Europe, Caucasus, and Asia Minor (Burakowski et al. 1973). Notifications for countries such as Belarus, Denmark, Latvia, and the Kaliningrad region as well as for Lithuania (Marggi et al. 2003; Vigna Taglianti 2009) is doubtful. We suggest the removal of *Odontium foraminosum*, *Ocydromus tibialis*, *Ocydromus decorus* and *Ocydromus modestus* from the list of Lithuanian beetles.

9. *Sinechostictus elongatus* (Dejean, 1831) is known for Lithuania only from the Palionis cards (Pileckis 1960; Ferenca 2006a) but was not noted later (Pileckis 1976a; Pileckis and Monsevičius 1995). This species is known in southern and central Europe, further to the north in Belgium, the Netherlands, and Germany (Vigna Taglianti 2009). The occurrence of *Sinechostictus elongatus* in Lithuania is very doubtful and its should be exclude from the list of Lithuanian beetles.

10. Only two records, older than 80 years, of *Bembidion quadripustulatum* Audinet-Serville, 1821 are mentioned in Palionis’ cards (Pileckis 1960; Pileckis and Monsevičius 1995; Ferenca 2006b).

11. Trustworthy data on occurrence of *Paratachys bistriatus* (Duftschmid, 1812) in Lithuania still missing. Pileckis and Monsevičius (1995) noted this species for Lithuania in accordance with A. Palionis’ finding in 1938.

12. Reliable data on occurrence of *Amara concinna* Zimmermann, 1832 in Lithuania still missing. (Pileckis (1960, 1976a) and Pileckis and Monsevičius (1995) noted this species for Lithuania in accordance with A. Palionis’ findings, but the authors of this publication did not find any verifying information in Palionis’ cards or collections.

13. Only few old records of *Calathus mollis* (Marsham, 1802)are known in Lithuania (Ogijewicz 1933), all over 80 years old.

14. Only few recordsof *Agonum duftschmidi* J. Schmidt, 1994 are known in Lithuania (Barševskis 2001; Vaivilavičius 2008). This species is very similar in both morphological and ecological characters to *Agonum afrum* (Duftschmid, 1812) and has not been correctly identified in the past.

15. *Chlaenius festivus* (Panzer, 1796) is noted for Lithuania by Kirschenhofer (2003). This species is known in southern Europe, Asia Minor and Caucasus. Distribution in the Lithuanian territory is unlikely and the species should be removed from the list of Lithuanian beetles.

16. The occurrence of *Cryptophonus melancholicus* (Dejean, 1829) in Lithuania has been disproved by Tamutis et al. (2008). This species is known in southern Sweden (Lundberg and Gustafsson 1995) and northern Poland (Burakowski et al. 1974), so it could be expected in Lithuania.

17. *Harpalus atratus* Latreille, 1804 is known in Lithuania probably from the Kaunas T. Ivanauskas’ zoological museum collections (Pileckis 1960; Gaidienė 1993), but was not confirmed (Pileckis 1976a; Pileckis and Monsevičius 1995). Furthermore, the authors of this publication did not find any confirmative information in the collections or in the cards of above-mentioned museum. We suggest the removal of this speies from the list of Lithuanian beetles.

18. Trustworthy data on occurrence of *Harpalus caspius* (Steven, 1806) = *roubali* Schauberger, 1928 in Lithuania still absent. Pileckis and Monsevičius (1995) noted this species for Lithuania for *Harpalus dimidiatus* (look at #19). *Harpalus caspius* is known in central and southern Europe and the Near East, so its occurrence in the territory of Lithuania is very doubtful. We suggest the removal of this species from the list of Lithuanian beetles.

19. *Harpalus* *dimidiatus* (P. Rossi, 1790) has been noted for Lithuania by Pileckis (1960) in accordance with A. Palionis’ findings, but this notification was not confirmed (Pileckis 1976a). The occurrence of this species in Lithuania has been disproved by Pileckis and Monsevičius (1995) on the grounds that the record of this species was not reliable and probably A. Palionis misidentified this species for *Harpalus roubali* Schauberger, 1928. *Harpalus dimidiatus* is distributed in the southern part of western and central Europe.

20. *Anisodactylus signatus* (Panzer, 1797) is known from the catalogue of entomological collections of Kaunas T. Ivanauskas’ zoological museum (Gaidienė 1993), but was not confirmed (Pileckis and Monsevičius 1995). Furthermore, the authors of this publication did not find any confirmative information in the collections or in the cards of mentioned museum and suggest the removal of this species from the list of Lithuanian beetles. However, *Anisodactylus signatus* is known in Latvia (Barševskis 2003), northern Poland (Burakowski et al. 1974), northwestern Belarus (Alexandrovitch et al. 1996) and the Kaliningrad region (Alekseev 2008c), so it could be expected in Lithuania.

21.*Bradycellus csikii* Laczó, 1912 was misidentified for *Bradycellus ruficollis* (Stephens, 1828) and is erroneously noted for Lithuania by Tamutis et al. (2008). We exclude this species from the list of Lithuanian beetles. *Bradycellus csikii* is known in southern Sweden (Lundberg and Gustafsson 1995), northwestern Belarus (Alexandrovitch et al. 1996), northeastern Poland (Aleksandrovich et al. 2003), the Kaliningrad region (Alekseev 2008c) and Denmark (Vigna Taglianti 2009), so it could be expected in Lithuania.

22. *Syntomus ai* Barševskis, 1993 is known only from the type locality in the Kauguri area (Riga Gulf dunes, Jūrmala, Central Latvia). The state of this species is in dispute (Telnov 2004).

23. *Haliplus lineolatus* Mannerheim, 1844 was recorded the first time in Lithuania by Ogijewicz (1933). This species was confirmed by Pileckis (1960), but in the monograph *Lietuvos vabalai* (Pileckis 1976a) *Haliplus lineolatus* was synonymised with *Haliplus wehnckei* Gerh. Later, in the monograph *Lietuvos Fauna. Vabalai* (Pileckis and Monsevičius 1995) both species *Haliplus lineolatus* and *Haliplus wehnckei* are indicated as valid; Sifverberg (1992, 2004) cited only *Haliplus wehnckei* = *Haliplus sibiricus* Motschulsky, 1860 = *Haliplus sahlbergi* Falkenström, 1940 for Lithuania . For more comprehensive information on the presence and distribution of these species in Lithuania, all deposited material should be revised.

24. *Agabus biguttulus* (Thomson, 1867) was misidentified for *Agabus affinis* (Paykull, 1798) and erroneously noted for Lithuania by Gaidienė (1993). This species should be removed from the list of Lithuanian beetles. This species is known in Kaliningrad region (Alekseev 2010a), Estonia, Sweden (Lundberg and Gustafsson 1995), Latvia (Barševskis et al. 2005), Belarus (Alexandrovich et al. 1996) and, Poland (Burakowski et al. 1976). This species could be expected in Lithuania.

25. *Ilybius wasastjernae* (C.R. Sahlberg, 1824) was misidentified for *Agabus sturmii* (Gyllenhal, 1808)and erroneously noted for Lithuania by Monsevičius (1998). This species should be removed from the list of Lithuanian beetles. It is known in Latvia (Barševskis et al. 2005), northern Poland (Burakowski et al. 1976), southern Sweden (Lundberg and Gustafsson 1995), Belarus and Denmark (Nilsson 2009), thus its could be expected in Lithuania.

26. The occurrence of *Laccornis oblongus* (Stephens, 1835) in Lithuania (Viešvilės nature reserve) has been noted only once by Monsevičius (1997), without any detailed information concerning location, habitat and date. But this fact is trustworthy since this species is recorded in all neighbouring territories: Latvia, Belarus, Poland, Kaliningrad region (Telnov 2004; Alexandrovitch et al. 1996; Burakowski et al. 1976; Alekseev 2010a).

27. Sometimes the author of species name *Hydrophorus rufifrons* is wrongly referred to as “Duftschmid, 1805” (Ogijewicz 1933; Burakowski et al. 1976, Pileckis 1960, 1976a; Pileckis and Monsevičius 1995). In fact the author is O.F. Müller, 1776.

28. *Oreodytes sanmarkii* (C.R. Sahlberg, 1826) was recorded for the first time in Lithuania by Pileckis (1976a), but later he suggested to remove this species from the list of Lithuanian beetles on the grounds that the finding was not reliable (Pileckis and Monsevičius 1995).

29. *Stictotarsus (Deronectes) duodecimpustulatus* F., and was mentioned in the first checklist of Lithuanian beetles on the grounds of A. Palionis’ cards (Pileckis 1960), but was not confirmed later.

30. Two species names *Helophorus pumilio* Erichson, 1837 and*Helophorus redtenbacheri* Kuwert, 1885 have been presented as synonyms and noted for Lithuania as single species by Lindeman (1871), (Pileckis (1968b, 1976a) and Pileckis and Monsevičius (1995). In reality there are two valid species (Burakowski et al. 1976; Lundberg and Gustafsson 1995; Silfverberg 1992, 2004; Hansen 2004a, 2009) and only one of them,*Helophorus redtenbacheri* Kuwert, 1885, has been noted for Lithuania by (Silfverberg (1992, 2004) and Hansen (2004a, 2009). Actually trustworthy data on the occurrence of both these species in Lithuania still lacking and we suggest the removal of both species from the list of Lithuanian beetles. Both species are distributed in central and eastern Europe. *Helophorus pumilio* is known in northwestern Poland (Burakowski et al. 1976), recently discovered in Latvia (Telnov et al. 2008) and Denmark (Hansen 2009). *Helophorus redtenbacheri* is known in Poland (Burakowski et al. 1976), southern Sweden (Lundberg and Gustafsson 1995), Estonia and Denmark (Hansen 2009). Hence, both species could be expected in Lithuania.

31. *Hydrochus ignicollis* Motschulsky, 1860 has been known as synonym of *Hydrochus elongatus* (Schaller, 1783) until Angus (1977) who proved the substantive status of these taxa. The situation on the occurrence of these species in Lithuania is still unclear. Only *Hydrochus elongatus* is mentioned in the works of Lithuanian scientists (Ogijewicz 1933; Pileckis 1960, 1976a; Gaidienė 1993; Pileckis and Monsevičius 1995; Monsevičius 1997; Šablevičius 2000b; Ivinskis et al. 2008), but foreign authors proposed that only *Hydrochus ignicollis* lives in Lithuania. In our opinion the occurrence of both species is possible, because the distribution area of both species in Europe is similar.

32.*Anacaena lutescens* (Stephens, 1832) has been known as synonym of *Anacaena limbata* (Fabricius, 1792) until van Berge Henegouwen (1986) proved the substantive status of these both taxa. The occurrence of these species in Lithuania was unclear until the revision of *Anacaena* material collected from Lithuania. In fact the data discussed in the previous publications (Mazurowa and Mazur 1939; Pileckis 1976a; Gaidienė 1993; Pileckis and Monsevičius 1995; Šablevičius 2000; Tamutis and Zolubas 2001; Vaivilavičius 2008) concern *Anacaena lutescens*, where this species has been wrongly noted as *Anacaena limbata* F. Trustworthy data on occurrence of *Anacaena limbata* in Lithuania has been published by Ferenca and Tamutis (2009).

33. The names and their synonyms of three European *Helochares* Mulsant, 1844 species *Helochares lividus* (Forster, 1771), *Helochares griseus* (Fabricius, 1787) and *Helochares obscurus* (O.F. Müller, 1776) were muddled for a long time. The clearest version has been suggested by Hansen (1982). The occurrences of these species in Lithuania are still unclear. The first record of Lithuanian *Helochares* belongs to *Helochares griseus* (Ogijewicz 1933). Most likely the author intended *Helochares obscurus*, the most common species of *Helochares* genus in this region. The same name was found in the Palionis cards, but afterwards this species was presented as a synonym of *Helochares lividus* by Pileckis (1960). In the monograph *Lietuvos vabalai* (Pileckis, 1976a) *Helochares griseus* and *Helochares lividus* have been presented as two valid species. The same situation is repeated in the last monograph *Lietuvos Fauna. Vabalai* (Pileckis and Monsevičius 1995), where, in the text besides *Helochares lividus*, is a note “*Helochares obscurus* = *Helochares griseus*.” In our opinion only one species is found in Lithuania; furthermore, *Helochares lividus* is distributed in southern and central Euorope and further to the north to southern England (Hansen 1982) and central Poland (Burakowski et al. 1976). The occurrence of this species in Lithuania is impossible and we suggest the removal of this species from the list of Lithuanian beetles.

34. Some authors suggested that the species *Enochrus fuscipennis* (Thomson, 1884) was a variation or synonym of *Enochrus quadripunctus* (Herbst, 1797) until Hansen (1987) proved the existence of two valid species. The occurrences of these species in Lithuania are still unclear. Contradictory data are presented in the papers of Lithuanian scientists (Pileckis 1960, 1976a; Gaidienė 1993; Pileckis and Monsevičius 1995).

35. Only one old record of *Plegaderus dissectus* Erichson, 1839, mentioned more than 130 years ago, is known in Lithuania (Lentz 1879).Pileckis and Monsevičius (1995) doubted the occurrence of this species on the grounds that the record of this species was not reliable.

36. Only one old record of the *Ochthebius metallescens* Rosenhauer, 1847, mentioned 77 years ago, is known in Lithuania (Ogijewicz 1933). Pileckis and Monsevičius (1995) doubted the occurrence of this species on the grounds that the record of this species was not reliable. This species so far is not known in the Fennoscandian countries, Belarus, the Kaliningrad region and northern Poland. It is considered as a mountain species, distributed in the mountain regions of southern and central Europe (Burakowski et al. 1976). Its occurrence in Lithuania is unlikely and we suggest the removal of this species from the list of Lithuanian beetles.

37.*Sogda ciliaris* (Thomson, 1874) = *hyperborea* (Strand, 1943) was erroneously noted for Lithuania by Perreau (2004), most likely instead of *Leiodes ciliaris* (W.L.E. Schmidt, 1841). *Sogda ciliaris* is distributed in northern Europe and so far not known in southern Sweden (Lundberg and Gustafsson 1995), Latvia (Telnov 2004) or Estonia (Alonso-Zarazaga 2009a). Its occurrence in Lithuania is unlikely. We suggest the removal of this species from the list of Lithuanian beetles.

38. Some authors considered two *Ptomaphagus* Hellwig, 1795 species, namely *Ptomaphagus sericatus* (Chaudoir, 1845) and *Ptomaphagus medius* (Rey, 1889), to be valid species (Szymczakowski 1961, 1971; Burakowski et al. 1978). Currently these taxa are treated as two subspecies by Zwick 1989, Perreau 2004 and Alonso-Zarazaga 2009a. *Ptomaphagus sericatus* has been noted for Lithuania by (Pileckis (1960, 1976a) and Pileckis and Monsevičius (1995). The nominotypical subspecies *Ptomaphagus sericatus sericatus* (Chaudoir, 1845) is distributed in the southeastern part of Europe, to the north until the central part of Poland, Germany and Belgium (Szymczakowski 1961; Alonso-Zarazaga 2009a), so its occurrence in the territory of Lithuania is doubtful. The other subspecies, *Ptomaphagus sericatus medius* (Rey, 1889), is widely distributed in western and central Europe, known in southern Sweden (Lundberg and Gustafsson 1995), Estonia (Silfverberg 1992, 2004), northern Belarus (Alexandrovitch et al. 1996) and Latvia (Telnov et al. 2008).

39. Trustworthy data on occurrence of *Batrisus formicarius* Aubé, 1833 in Lithuania is still lacking. There are only a few old notes of this species for Lithuania (Łomnicki 1913; Jakobson 1905–1915), which have been referred to later (Pileckis 1968b, 1976a; Pileckis and Monsevičius 1995).

40. *Euplectus bonvouloiri narentinus* Reitter, 1881 is noted for Lithuania by (Jakobson (1905–1915) with a question mark.

41. The trustworthy data on the occurrence of *Plectophloeus nitidus* (Fairmaire, 1857) in Lithuania is still missing. At present only a few old notes are known for Lithuania (Osterloff 1889; Łomnicki 1913, Jakobson 1905–1915). Pileckis and Monsevičius (1995) doubted the occurrence of this species in Lithuania.

42. The trustworthy data on the occurrence of *Amauronyx maerkelii* (Aubé, 1844) in Lithuania is still missing. The first notification of this species for Lithuania was made by Pileckis (1976a), but this fact was later disproved (Pileckis and Monsevičius 1995). This species is known in Denmark, southern Sweden (Lundberg and Gustafsson 1995) and Poland (Burakowski et al. 1978).

43. Some authors treated *Brachygluta sinuata* (Aubé, 1833) as a valid species and noted for Lithuania (Smetana and Besuchet 2004a; Alonso-Zarazaga 2009a). Kurbatov (2007) proposed that only *Ptomaphagus haematica* (Reichenbach, 1816) occurs in the Baltic states. Other authors treated this name as a subspecies of *Ptomaphagus haematica* (Reichenbach, 1816) (Besuchet 1974; Lundberg and Gustafsson 1995, Telnov 2004; Silfverberg 1992, 2004). In fact any trustworthy information on occurrence of *Ptomaphagus sinuata* in Lithuania is missing.

44. Trustworthy data on the occurrence of *Bythinus securiger* (Reichenbach, 1816) in Lithuania is still missing. There is only one old note of this species for Lithuania (Jakobson 1905¬1915), which has been referred to later (Pileckis 1976a; Pileckis and Monsevičius 1995) This species is distributed mainly in central and southern Europe, but so far it is not known in Fennoscandian countries, Belarus or northern Poland.

45. *Bryaxis nodicornis* (Aubé, 1833) is noted for Lithuania by (Jakobson (1905–1915) and wrongly placed in the genus *Euthia* (Scydmaeidae) by Pileckis (1976a). This species was not mentioned later (Pileckis and Monsevičius 1995).

46. *Aleochara morion* Grav. and *Tinotus morion* Grav. were erroneously treated as two valid species by Pileckis (1976a). In fact there is only one species *Tinotus morion* (Gravenhorst, 1802).

47. The two names *Leptusa circellaris* Grav. and *Evanystes circellaris* Grav. have been erroneously treated as two valid species by Pileckis (1976a). In fact there is only one species*Geostiba circellaris* (Gravenhorst, 1802).

48. Trustworthy data on the occurrence of *Taxicera deplanata* Gravenhorst, 1802 in Lithuania is still lacking. The first notification was made by Pileckis (1976a), but Pileckis and Monsevičius (1995) doubted its occurrence . This species so far is not known in the Fennoscandian countries, Belarus, Kaliningrad region, or northern Poland, but distributed in southern parts of central Europe (Benick and Lohse 1974) and further to the north in Belgium more recently (Drugmand and Pontégnie 2002). Its occurrence in Lithuania is impossible and we suggest the removal of this species from the list of Lithuanian beetles.

49. *Amischa analis* Grav. and *Atheta analis* Grav. has been erroneously treated as two valid species by Pileckis (1976a), but in fact its correct taxon is*Amischa analis* (Gravenhorst, 1802).

50. *Cephennium thoracicum* O.F. Müller & Kunze, 1822 is first noted for Lithuania by Osterloff (1889), but Pileckis and Monsevičius (1995) doubted the occurrence of this species.

51. Pileckis (1976a) has wrongly listed *Nevraphes talparum* Lokay, 1920 as a synonym of *Nevraphes rubicundus* (Schaum, 1841). *Nevraphes rubicundus* wasfirst noted for Lithuania by Osterloff (1889), but Pileckis and Monsevičius (1995) doubted the occurrence of this species in Lithuania and suggested that *Nevraphes talparum* was found in Lithuania. The situation on these two species in Lithuania is still unclear.

52. *Stenus gallicus* Fauvel, 1873 is noted for Lithuania (Viešvilės nature rezerve) by Monsevičius (1997), without any detailed information. This species is known in Latvia (Vorst et al. 2007), Denmark, southern Sweden (Lundberg and Gustafsson 1995) and Poland (Burakowski et al. 1979). The notification of Monsevičius is quite trustworthy but it is likely that this species was declared instead of *Stenus excubitor* Erichson, 1839, which is more widely distributed in Europe. It is worth to noting that *Stenus gallicus* is treated as variation of *Stenus excubitor* by some authors (Szujecki 1961; Lohse 1964; Burakowski et al. 1979).

53. *Chilothorax paykulli* Bedel, 1908 = *tessulatus* (Paykull, 1798) nec (Moll, 1782) was erroneously noted for Lithuania by Gaidienė (1993) and Silfverberg (2004), and we suggest its removal from the list of Lithuanian beetles. This species is known in Latvia (Telnov 2004), Estonia, Denmark, southern Sweden (Lundberg and Gustafsson 1995), Poland (Stebnicka 1976) and Belarus (Alexandrovitch et al. 1996), so it could be expected in Lithuania.

54. *Bodilus punctipennis* (Erichson, 1848) is noted for Lithuania by Gaidienė (1993) and Silfverberg (2004), but this notification based on a misindentification for *Agrilinus sordidus* (Fabricius, 1775). *Bodilus punctipennis* must be removed from the list of Lithuanian beetles. This species is distributed in southeastern Europe, northern Africa and the Near East (Alonso-Zarazaga 2009a).

55. The taxonomy and distribution of European species of *Osmoderma* LePeletier & Audinet-Serville, 1828 are well explaned by Audisio et al. (2007).

56. Formerly *Elodes pseudominuta* Klausnitzer, 1971 was treated as a synonym of *Elodes minuta* (Linnaeus, 1767).

57. The occurrence of *Ctenicera cuprea* (Fabricius 1775) in Lithuania has been disproved by (Tamutis et al. 2010). This species is known in Estonia, Sweden (Lundberg and Gustafsson 1995), northern Belarus (Alexandrovitch et al. 1996) and northern Poland (Tarnawski and Buchholz 2008a), so it could be expected in Lithuania.

58. *Cratosilis denticollis* (Schummel, 1844) was noted for Lithuania by Roubal (1910) (Pileckis 1960), but it was not confirmed later (Paleckis 1976a; Pileckis and Monsevičius 1995). This species is usually considered as a mountain species and is distributed in the mountain regions of Europe (Dahlgren 1979). Its occurrence in Lithuania is impossible and we suggest the removal of this species from the list of Lithuanian beetles.

59. Some authors treated *Epuraea fageticola* Audisio, 1991 as the synonym of *Epuraea hilleri* Reitter, 1877 (Silfverberg 2004; Telnov 2004), but according Kirejchuk (1997) there are two valid species.

60. *Epuraea abietina* J.R. Sahlberg, 1889 and *Epuraea florea* Erichson, 1845 were treated as two valid species (Heyden 1903; Roubal 1910; Pileckis 1960, 1976a; Pileckis and Monsevičius 1997).

61. The generic classification of Meligethinae was recently re-organised by Audisio et al. 2009.

62. Pileckis and Monsevičius (1995) doubted the occurrence of *Ipidia sexguttata* (R.F. Sahlberg, 1834) in Lithuania implying that the record of this species was not reliable. This species is distributed in the eastern Europe (Burakowski et al. 1986b); so far is not known in the Fennoscandian and Baltic countries, Kaliningrad region or northern Poland. We suggest the removal of this species from the list of Lithuanian beetles.

63. *Henosepilachna vigintioctomaculata* (Motschulsky, 1857) has been noted for Lithuania by Šablevičius (2000b). This species is mainly distributed in the Far East, westwards to the Amur region (Fosulati 2003–2009) and is not found in the Europe. it is likely that this notification was based on misidentified specimens. This species shoud be removed from the list of Lithuanian beetles.

64. *Melanophthalma transversalis* (Gyllenhal, 1827)has been mentioned twice but as two valid species: *Corticaria hortensis* Motsch. and *Melanopthalma transversalis* Gyll., by Pileckis (1976a).

65. Notification of *Pedinus femoralis* (Linnaeus, 1767) for Lithuania was doubtful (Pileckis and Monsevičius 1997) becouce it was based on unreliably identified larvae. The authors could not confirm this record because the material is lost. This species should be removed from the list of Lithuanian beetles, as it is distributed in southern and southeastern Europe, to the north to southern Poland and Germany (Burakowski et al. 1987; Fattorini 2009).

67. The taxonomical status of *Rhamnusium gracilicorne* Théry, 1895 and *Rh. bicolor* (Schrank, 1781) is still questionable. These two species have been presented as valid species by Pileckis (1976a), Althoff and Danilevsky (1997), Pileckis and Monsevičius (1997), Alexandrovitch et al. (1996), but treated as synonyms by others (Zawadzki 1936; Burakowski et al. 1989; Silfverberg 2004; Telnov 2004; Sama 2009).

68. *Paracorymbia fulva* (DeGeer, 1775) has been noted for Lithuania by Gaidienė (1993). This species must be removed from the list of Lithuanian beetles because this notification is erroneous. The occurrence of this species in Poland and Belarus is also doubted (Althoff and Danilevsky 1997).

69. *Anastrangalia reyi* (Heyden, 1889) is very similar in both morphological and ecological characters to *Anastrangalia dubia* (Scopoli, 1763) and has not been correctly identified in the past. For more comprehensive information on the presence and distribution of these species in Lithuania, all collected material should be revised.

70. *Leptura aurulenta* Fabricius, 1792was misidentified for *Leptura quadrifasciata* Linnaeus, 1758 and wrongly noted for Lithuania by Pileckis (1963b), also later (Pileckis 1970b, 1976a, b, 1979; Pileckis and Monsevičius 1997; Silfverberg 1992, 2004; Alekseev 2007). This species should be removed from the list of Lithuanian beetles. *Leptura aurulenta* is known in southern and central Europe and northern Africa. In the north, the distribution range of *Leptura aurulenta* covers southern and southwestern Poland (Burakowski et al. 1989) and southern Germany (Schoppmann 1989), so its occurrence in Lithuania is very doubtful.

71. *Isotomus comptus* (Mannerheim, 1825) is noted for Lithuania by Gaidienė date, but the authors of current paper did not find any confirmation and suggest the removal this species from the list of Lithuanian beetles. This species is distributed in southeastern Europe (Althoff and Danilevsky 1997).

72. *Isotomus speciosus* (Mannerheim, 1825) has been noted for Lithuania by Pileckis (1959), but disproved later (Pileckis 1960). However this species is noted for Lithuania by Althoff and Danilevsky (1997) and Sama (2009). This species is distributed mainly in southern Europe; in the northern part of central Europe it is very rare, known only in southern Poland (Burakowski et al. 1989).

73. *Clytus rhamni* (Germar, 1817) has been noted for Lithuania by Pileckis (1959), but disproved later (Pileckis 1960). The notifications of some authors that this species occurs in Lithuania are unconfirmed (Silfverberg 2004; Alekseev 2007; Sama 2009). This species is known in Belarus (Alexandrovitch et al. 1996) and Poland (Burakowski et al. 1989).

74. *Acanthocinus reticulatus* (Razoumowsky, 1789) has been noted for Lithuania by Pileckis (1959), but disproved later (Pileckis 1960). This species is known in northeastern Poland (Burakowski et al. 1989) and northwestern Belarus (Alexandrovitch et al. 1996), so it could be expected in Lithuania.

75. *Leiopus femoratus* Fairmaire, 1859 was misidentified for *Leiopus linnei* Wallin, Nylander & Kvamme, 2009 and wrongly noted for Lithuania by Ferenca (2004). This species should be removed from the list of Lithuanian beetles. *Leiopus femoratus* is distributed in southern Europe (France, Italy, Bulgaria), Asia Minor, northern Iran and the Caucasus (Brelih et al. 2006) and its occurrence in Lithuania is not realistic. *Leiopus linnei* is a recently described species from Scandinavian countries (Walinn et al. 2009), noted as very common for Latvia (Telnov et al. 2010), and recently recorded in Belarus and Poland (Gutowski et al. 2010).

76.*Tetrops starkii* Chevrolat, 1859 is very similar to *Tetrops praeusta* (Linnaeus, 1758) and for more comprehensive information about its presence and distribution in Lithuania all collected material of *Tetrops praeusta* should be revised.

77. *Callosobruchus chinensis* (Linnaeus, 1758) is noted for Lithuania by Audisio (2009). This cosmopolitan species is imported into almost all European countries (Audisio 2009), but still not confirmed for Estonia, Latvia or the Kaliningrad region. It was found only once in Lithuania, in souvenirs from Thailand in 2007 (the material is stored in Kaunas T. Ivanauskas zoological museum), but other factors indicating the presence of this species in Lithuania are absent.

78. Two species *Oulema erichsoni* (Suffrian, 1841) and *Oulema septentrionis* (Weise, 1880) were considered as synonyms by some authors (Pileckis 1976a; Pileckis and Monsevičius 1997; Silfverberg 1992, 2004). However these two sibling species are valid and differ by the shapes of the aedeagus apex and flagellum (Bukejs 2010b).

79. *Pilemostoma fastuosa* (Schaller, 1783)was wrongly identified for *Cassida murraea* Linnaeus, 1767and erroneously noted for Lithuania by Šablevičius (2000a). *Pilemostoma fastuosa* should be removed from the list of Lithuanian beetles. However this species is known in Latvia (Telnov 2004), northeastern Poland (Burakowski et al. 1991), Denmark and southern Sweden (Lundberg and Gustafsson 1995), so it could be expected in Lithuania.

80. *Cassida azurea* Fabricius, 1801was wrongly identified for *Cassida sanguinolenta* O.F. Müller, 1776and erroneously noted for Lithuania by Tamutis (2003). *Cassida azurea* should be removed from the list of Lithuanian beetles. This species is known in central Poland (Burakowski et al. 1991) and southern Belarus (Alexandrovitch et al. 1996), so its could be expected in Lithuania.

81. *Chrysolina herbacea* Duftschmid, 1825 was wrongly identified for *Chrysolina varians* (Schaller, 1783) and erroneously noted for Lithuania (Tamutis 2003). The true finding of this species in Lithuania has been recently confirm by Bukejs et al. (2011).

82. *Timarcha tenebricosa* F. is noted for Lithuania in previous publications on Lithanian beetle fauna (Pileckis 1960, 1976a) based on the data of A. Palionis’ cards. Later this record was reidentified to *Timarcha goettingensis* (Linnaeus, 1758) (Pileckis and Monsevičius 1997). In fact trustworthy confirmation on the occurrence of this species in Lithuania is absent.

83. There are four sibling species: *Galerucella nympaeae* (Linnaeus, 1758), *Galerucella aquatica* (Geoffroy 1785), *Galerucella sagittariae* (Gyllenhal, 1713) and *Galerucella kerstensi* Lohse, 1989, which are usually distinguished by their host plants and subtle details of reproductive structures (Lohse 1989; Nokkala and Nokkala 1998; Nesterova 2008). Some authors synonymised these species, introduced them as synonyms or ecological races based on their host plants of *Galerucella nympaeae* (Beenen 2008; Audisio 2009), or as partial synonyms, for example *Galerucella sagittariae* = *Galerucella kerstensi* (Silfverberg 2004), *Galerucella nympaeae* = *Galerucella aquatica* (Burakowski et al. 1990). *Galerucella sagittariae* (Gyllenhal, 1713) is noted for Lithuania by Lundberg and Gustafsson (1995) and *Galerucella sagittariae* (Gyllenhal, 1713) = *Galerucella kerstensi* Lohse, 1989 is noted by (Silfverberg (1992, 2004).

84. *Aphthona flaviceps* Allard, 1859 is noted for Lithuania by Šurkus and Gaurilčikienė (2002). This species is distributed in northern Africa and southern Europe (Burakowski et al. 1991), to the north to France, Republic of Moldova and the Ukraine. Its occurrence in Lithuania is impossible. This species was noted erroneously and should be removed from the list of Lithuanian beetles.

85. *Longitarsus scutellaris* (Rey, 1874) was noted for Poland, Estonia, Finland, Belarus and Latvia in the previous beetle checklists of neighbouring countries (Burakowski et al. 1991; Sifverberg 1992; Lundberg and Gustafsson 1995, Alexandrovitch et al. 1996; Telnov 2004), but later this species was removed from the list of Latvian beetles (Bukejs 2010) as this species was replaced with *Longitarsus lewisii* (Baly, 1874), recorded in Latvia by Sifverberg (2004). We suppose that only *Longitarsus lewisii* could be expected for Lithuania, because *Longitarsus scutellaris* is distributed mainly in southern and southeastern Europe, Turkey and the Caucasus (Borowiec 2007-2010).

86. *Altica engsroemi* J.R. Sahlberg, 1893 and *Altica chamaenerii* H. Lindberg, 1926: the taxonomic status is still in dispute. In the opinion of some scientists, these species are valid (Gruev and Döberl 1997; Kangas and Rutanen 1993; Warchałowski 2003), but others have considered *Altica engstroemi* Sahlb. as a synonym of *Altica aenescens* Weis. or *Altica chamaenerii* Lindb. as a synonym of *Altica brevicollis* Foudr. (Bieńkovski 2004; Lopatin and Nesterova 2005).

87. *Epitrix cucumeris* (Harris, 1851) has been noted for Lithuania by Pileckis and Vengeliauskaitė (1996) but its distributed mainly in North and Central America. It was introduced in the Azores, Madeira and Portugal (Anonymous 2005; Boavida and Germain 2009). This species was noted erroneously and should be removed from the list of Lithuanian beetles.

88. *Bruchela suturalis* (Fabricius, 1793) has been treated as an expected species for Lithuania by Pileckis and Monsevičius (1997), but afterwards, as a result of wrong interpretation, this species was then noted for Lithuania by Silvferberg (2004). In fact *Bruchela suturalis* is still not found in Lithuania.

89. The name *Perapion oblongum* (Gyllenhal, 1827) was considered to be a synonim of *Perapion curtirostre* (Germar, 1817) by some entomologists (Smreczyński 1965; Legalov 2001), but in Europe *Perapion oblongum* is a distinct biological species, diagnostic characters of which have been well-described by Gønget (1997).

90. The taxonomic status of the genus *Tournotaris* Alonso-Zarazaga & Lyal 1999 is well explained by Wanat and Mokrzycki (2005).

91. *Cionus olivieri* Rosenschöld, 1838was misidentified for *Cionus longicollis* Brisout, 1863 and wrongly noted for Lithuania by Tamutis (2003). This species should be removed from the list of Lithuanian beetles. *Cionus olivieri* is known in Latvia (Telnov 2004), Estonia (Silfverberg 2004), northwestern Belarus (Alexandrovitch et al. 1996) and Poland (Smreczyński 1976; Wanat and Mokrzycki 2005), so it could be expected in Lithuania.

92. *Orchestes testaceus* (O.F. Müller, 1776) and *Orchestes calceatus* (Germar, 1821) have been treated as synonyms by Anderson (1989) and Alonso-Zarazaga (2009a). However other authors treated them as two valid species (Lundberg and Gustafsson 1995; Silfverberg 2004; Telnov 2004; Wanat and Mokrzycki 2005). We accepted the first position in this paper.

93. *Miarus fennicus* Kangas 1978 has been presented as distinct species by Lundberg and Gustafsson (1995) and Alonso-Zarazaga (2009a), but *Miarus fennicus* Kangas, 1978was placed as a junior synonym of*Miarus campanulae* (Linnaeus, 1767) by Vahtera and Muona (2006).

94. Generic classification of the subfamily Bagoinae has been tentatively simplified by the authors of a recent revision of Palaearctic species (Caldara and O’Brien 1998). They amalgamated all European generic taxa into one genus *Bagous* divided into a number of monophyletic species groups, not into subgenera.

95. *Hadroplonthus trimaculatus* (Germar, 1824) was identified for *Hadroplonthus litura* (Fabricius, 1775) and wrongly noted for Lithuania by Tamutis (1996). *Hadroplonthus trimaculatus* (Germar, 1824) should be removed from the list of Lithuanian beetles. This species mainly distributed in the southern Europe (Smreczyński 1974; Alonso-Zarazaga 2009a).

96. *Otiorhynchus coecus* Germar, 1824 is noted for Lithuania by Žiogas (1997) as a widespread species in Lithuania. However this notification is still lacking confirmation.

97. *Otiorhynchus gemmatus* (Scopoli, 1763) is noted for Lithuania by Pileckis (1960) according to the data of A. Palionis’ cards, but was not noted in the monographs (Pileckis 1976a; Pileckis and Monsevičius 1997). This species is known in southern and central Europe, northward to Germany, Czech Republic, Slovakia and the Ukraine (Alonso-Zarazaga 2009a). Its occurrence in Lithuania is unlikely and this species should be removed from the list of Lithuanian beetles.

98. *Omiamima concinna* (Boheman 1834) is noted for Lithuania by Pileckis (1976a) and Gaidienė (1993), but has not been confirmed (Pileckis and Monsevičius 1997). It is likely that these records were based on misidentified specimens. This species is known in southern Europe (Alonso-Zarazaga 2009a). The occurrence of *Omiamima concinna* in Lithuania is unlikely and this species should be removed from the list of Lithuanian beetles.

99. *Psallidium maxillosum* (Fabricius, 1792) is noted for Lithuania by Eichwald (1830), but this fact was not confirmed later (Pileckis 1960, 1976a; Pileckis and Monsevičius 1997). This species is known in central and southeastern Europe, to the north to Germany, Poland, Ukraine (Alonso-Zarazaga 2009a); the occurrence in Lithuania is unlikely and this species should be removed from the list of Lithuanian beetles.

100. *Pseudomyllocerus canescens* (Germar, 1824) has been wrongly identified for *Phyllobius betulinus* (Bechstein & Scharfenberg, 1805) and erroneously noted for Lithuania by (Pileckis (1960, 1963b, 1976a) and Pileckis and Monsevičius (1997). *Pseudomyllocerus canescens* (Germar, 1824) is distributed in southern and central Europe, northward to southern Poland (Smreczyński 1965), Germany, Czech Respublic, Slovakia and the Ukraine (Alonso-Zarazaga 2009a), so its occurrence in Lithuania is unlikely. This species should be removed from the list of Lithuanian beetles.

101. *Phyllobius viridicollis* (Fabricius, 1792) is noted as a widespread species in Lithuania (Pileckis et al. 1968); however, this notification is still unconfirmed.

102. We used the classification of the subfamily Hyperinae accepted by Skuhrovec (2008).

103. *Larinus jaceae* (Fabricius, 1775) has been noted for Lithuania by Gaidienė (1993). This species must be removed from the list of Lithuanian beetles because this notification is erroneous. This species is known in Latvia (Telnov 2004), Estonia (Silfverberg 2004) and Poland (Smreczyński 1966; Wanat and Mokrzycki 2005), so its could be expected in Lithuania.

104. *Scolytus laevis* Chapuis, 1869 is noted for Lithuania as a rare species by Pileckis et al. (1968), Žiogas (1997) and Valenta (2000b); however, this notification is still unconfirmed.

105. *Xyleborus dryographus* (Ratzeburg, 1837) is known for Lithuania only from the Palionis cards (Ferenca 2006a), but was not confirmed by other Lithuanian entomolgists. We suggest the removal of this species from the list of Lithuanian beetles because any cofirmation of this record is lacking. This species is known in Sweden (Alonso-Zarazaga 2009a) and Poland (Nunberg 1954; Wanat and Mokrzycki 2005), so it could be expected in Lithuania.
